# A Monograph of *Conostegia* (Melastomataceae, Miconieae)

**DOI:** 10.3897/phytokeys.67.6703

**Published:** 2016-07-20

**Authors:** Ricardo Kriebel

**Affiliations:** 1 Department of Botany, University of Wisconsin-Madison, 430 Lincoln Drive Madison, Wisconsin 53706, USA

**Keywords:** Conostegia, Melastomataceae, monograph, Neotropics

## Abstract

A recent molecular phylogenetic analysis identified a clade containing all species of *Conostegia*, but that also included species of *Clidemia* and *Miconia* nested inside. A taxonomic revision of a more broadly circumscribed *Conostegia* is presented here. In total, 77 species of *Conostegia* are recognized. One species from Ecuador, *Conostegia ortizae* is described as new. Twenty-nine new combinations are proposed for the species of *Clidemia* and *Miconia* that fall inside *Conostegia*. Two new names are proposed for the two species for which the epithet was previously occupied in *Conostegia*. An infrageneric classification of *Conostegia* is proposed recognizing three sections based on the results of the molecular phylogeny. This taxonomic revision includes ample documentation of the anatomy and morphology of most species in the genus, taxonomic descriptions, a dichotomous key, and distribution maps for all species.

## Introduction

*Conostegia* D. Don, a genus in the tribe Miconieae (Melastomataceae), is most famous for the calyptrate calyx of the flowers of its species. The group was revised by Schnell (1996) who recognized 42 species, but this thorough revision unfortunately was never published. A recent molecular phylogenetic analysis based on DNA sequences from four chloroplast and two nuclear ribosomal spacers found *Conostegia* not to be monophyletic but identified a core *Conostegia* clade (Kriebel et al. 2015). This *Conostegia* clade identified contains all species sampled of *Conostegia*, in addition to some species of *Clidemia* D. Don and *Miconia* Ruiz & Pav. most of which are narrowly endemic taxa from Costa Rica and Panama. Ancestral state reconstruction of the calyptra onto the resulting molecular phylogeny of *Conostegia* provided evidence for the multiple origins of this structure albeit with different anatomical characteristics in each origin (Kriebel et al. 2015). The main objective of this revision is to provide a detailed taxonomic account guided by these recent findings. For this purpose, a broadened circumscription of *Conostegia* is here proposed. This revision also includes the proposal of an infrageneric classification, extensive documentation of the anatomy and morphology in the genus, a key for the identification of its species, descriptions, phenological graphs, diagnostic illustrations for most species and distributional maps.

Some authors have suggested that the best solution to the problem of lack of monophyly of many genera of the tribe Miconieae (Michelangeli et al. 2004; Goldenberg et al. 2008; Martin et al. 2008) is to lump all of them into a giant expanded genus *Miconia*, which could then be recognized by the presence of berry fruits (Ionta et al. 2012; Ionta and Judd 2012; Judd and Ionta 2013; Judd and Majure 2013; Majure and Judd 2013a, 2013b). These publications recommended and have started to divide the expanded *Miconia* into sections. Thus, the tribe Miconieae, in their view contains a single genus with about 1800 species divided into an uncertain number of sections. Given that most sections recognized to date are as small as three taxa, it is reasonable to believe the number of sections that will have to be recognized is quite large. This problem also applies with the large number of genera that would have to be created to accommodate all species within Miconieae. The case of *Conostegia* is different in that sampling for phylogenetic and morphological studies include most of its species, something far from happening for a group as large as the Miconieae. Using sections is problematic because taxonomic databases and herbaria usually do not include infrageneric classifications in their organization. These authors argue that lumping all taxa into *Miconia* will result in taxonomic stability because most species were already included in *Miconia*, and that fewer taxonomic changes will have to be made than if separate genera are recognized. An aspect that is less mentioned is how many new names would have to be created to accommodate species in all genera being lumped into a broadly circumscribed *Miconia*. In other words, what is more unstable, to create new combinations or to create altogether new names? For example, including all species of *Conostegia* in *Miconia* would require 30 new names, whereas including its species in an expanded *Conostegia* requires only two new names. I roughly calculated the number of new names required to accomodate all species of the Miconieae in the genus *Miconia* and came up with the number 354 new names. This means that the epithet of those taxa will change all together. It is not clear how to measure taxonomic stability, but I argue that that amount of new names is unnecessary and will result in high nomenclatural instability. The last phylogeny of the Miconieae included two genes for 449 species (Goldenberg et al. 2008), which represents around 25% of the tribe. Greater sampling both of molecular data as well as species would be desirable when so many nomenclatural changes are being considered. If more data results in better supported clades, these could be considered as the genera to recognize. This point was emphasized by Goldenberg and collegues (2008) who cautioned on making major taxonomic realignments since they would be “premature” based on their results. Some authors on the other hand are choosing the alternate route and recognizing genera within the Miconieae such as *Killipia* and *Leandra* s.s. (Posada-Herrera and Mendoza-Cifuentes 2013; Reginato 2014).

The recognition in this study of a broad *Conostegia* is based on the best sampled and only phylogenetic study of *Conostegia* which places all species in a clade within the Miconieae, and since most currently recognized species fall in this clade, and most can easily be recognized by the presence of the calyptra, it is deemed more useful to broaden *Conostegia* than to lump its species in a giant *Miconia*. Lastly, the species of *Clidemia* and *Miconia* that fall within *Conostegia* are almost all endemic to Costa Rica and Panama evidencing the strong and useful geographic component that together with morphology can be used in identifying species of this group.

## Taxonomic history

The idea of separating a group of calyptrate species in the Melastomataceae was first suggested by Bonpland (1806-1816), who intended to group them into the new genus *Calyptres*, but never validly published the name. Subsequently, and apparently following Bonpland’s idea, David Don (1823) formally described the genus *Conostegia* emphasizing the presence of the calyptrate calyx. The name derives from *conus* (meaning ‘cone’) and *tectum* (meaning ‘roof’). Don (1823) included nine species in his concept of *Conostegia* all of which were known as *Melastoma* at the time, namely *Melastoma calyptrata* Desr., *Melastoma extinctorum* Bonpl., *Melastoma glabratum* Sw., *Melastoma montanum* Sw., *Melastoma procera* Sw., *Melastoma xalapense* Bonpl. and the following three species which were undescribed at the time and each followed in Don’s publication: *Melastoma superbum* Bonpl., *Melastoma cucullata* Pav. ex D. Don, and *Melastoma holosericea* Steud. Ex Triana (Schnell 1996). Don (1823) did not choose a type because this was not customary at the time, but he provided a description, diagnosis and discussion of the affinities of *Conostegia* to *Miconia*. Schnell (1996) informally designated *Melastoma procera* as the type of *Conostegia*.

The combinations of Don’s species to *Conostegia* were done by Candolle (1828) but the latter author credited the names in *Conostegia* to Don. Candolle (1828) provided descriptions for sixteen species of *Conostegia* and was one of only two monographers to provide an infrageneric classification in *Conostegia*. He organized the genus into two sections. The first section *Eriostegia* DC. contained only one species that had been described as *Melastoma mutisii* Bonpl. and was proposed as *Conostegia mutisii* (Bonpl.) DC. The globose, hispid and abrupt apex of the calyptra characterized this section. *Melastoma mutisii* was eventually included in the capsular fruited genus *Centronia* (Triana, 1872) and very recently included in *Meriania* (Mendoza-Cifuentes & Fernández-Alonso, 2012). The second section he called *Euconostegia* and characterized it based on the shape of the calyptra, which was abruptly acuminate and also hispid. The next worker to treat *Conostegia* was Naudin (1850) who provided detailed descriptions for 11 species and cited an additional seven names. Most of these taxa are currently recognized in *Conostegia*. Naudin (1850) did not provide an infrageneric classification for *Conostegia*. After Naudin, Triana (1872) treated 23 species of *Conostegia* in his monograph of the family which included detailed descriptions for seven of them. Most of those taxa are also recognized today. Lastly, before a break in the revision of the genus that would last about 100 years, Cogniaux (1891) treated 34 species of *Conostegia*. Later Schnell (1996) provided the only detailed classification within *Conostegia* in which he recognized 42 species and divided them in three subgenera (*Conostegia*, *Lobatostigma* and *Ossaeiformis*) and further divided subgenus *Conostegia* into six sections (*Axiliflora*, *Conostegia*, *Dasystegia*, *Notostegia*, *Parvistigma* and *Tomentostegia*). Unfortunately Schnell’s work was never published. The genus had always been thought of as a morphologically distinct group (Bonpland 1806-1816; Don 1823; Almeda 1990; Schnell 1996) and in one of the few thorough revisions of the taxonomy of genera with terminal inflorescences, Judd and Skean (1991) concluded that *Conostegia* was likely monophyletic. After the work of Schnell (1996), the most comprehensive study that included species of *Conostegia* has been that for the Flora Mesoamericana by Almeda (2009). The latter study followed very closely the work of Schnell (1996).

### Phylogeny and infrageneric classification

**Figure 1.**
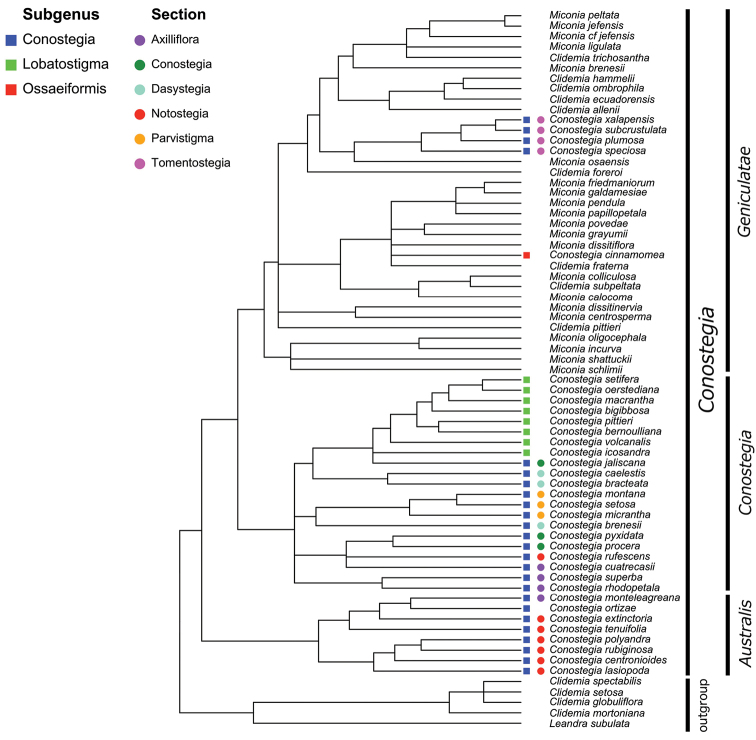
Infrageneric classification in *Conostegia* next to the recently generated molecular phylogenetic hypothesis of *Conostegia*. Colors show the classification proposed by Schnell (1996). To the right of the phylogeny is the sectional classification proposed here. The phylogeny is a slightly modified version of the majority rule consensus tree derived from a Bayesian analysis of DNA sequences from four chloroplast regions and two nuclear ribosomal spacers (Kriebel et al. 2015) pruned to one accession per species.

The most recent hypothesis of relationships in *Conostegia* based on DNA sequences from four chloroplast and two nuclear ribosomal regions, resolved the genus as paraphyletic with species of *Clidemia* and *Miconia* nested inside (Kriebel et al. 2015). Nonetheless, all species of *Conostegia* fall in a major clade regardless of the type of analysis conducted (concatentation vs. concordance). A summary of the relationships within *Conostegia* based on the hypothesis derived from the concatenated analysis is presented in Figure 1. These results also show the paraphyly of Schnell’s (1996) subgenus *Conostegia* since the species of his subgenus *Lobatostigma* form a clade nested inside of it. With respect to Schnell’s sectional classification of subgenus *Conostegia* his groups are not monophyletic except sections *Parvistigma* and *Tomentostegia*. For this reason, the subgeneric classification adopted here includes only sections corresponding to the three major clades in *Conostegia* identified by both concatentation and concordance analyses and proposed as *Conostegia* sections *Australis*, *Conostegia*, and *Geniculatae*, respectively (Fig. 1). Section *Australis* has calyptrate calyces that break at one side, abundant sclereids in the hypanthium, mucilage in the ovary, exserted styles, lack of filament geniculation, and all but one species sampled lack a vascular cylinder in the style. Even then, the cylinder in *Conostegia ortizae* has a single vascular bundle (unlike species in section *Conostegia* which have more). Species in section *Conostegia* are almost unique within the genus in that their styles are not exserted (flowers not herkogamous). This short style is only shared with some populations of *Conostegia monteleagreana* in section *Australis* and the clade composed of *Conostegia osaensis*, *Conostegia plumosa*, *Conostegia speciosa*, *Conostegia subcrustulata* and *Conostegia xalapensis* within section *Geniculatae*. An additional character almost unique to section *Conostegia* and not shared with any of the latter short-styled taxa is the presence of a vascular cylinder within the style. Additional characteristics of section *Conostegia* include the abundant sclereids in the hypanthium and presence of ovary mucilage (shared with section *Australis*). Based on anatomy and morphology, section *Australis* and section *Conostegia* are quite similar. These two sections also share the frequent pleiostemonous condition and the lack of an evident filament geniculation. The two groups differ in that species of section *Conostegia* do not have exserted styles, and all have vascular cylinders in the style whereas species of section *Australis* have exserted style and all but one species lack the vascular cylinder in the style. Lastly, many species in section *Australis* have an “anther shoulder” that species of section *Conostegia* lack. Section *Geniculatae* can be recognized by the lack of calyptra in most of its species. Only the following taxa have a calyptra: *Conostegia cinnamomea*, *Conostegia osaensis*, *Conostegia plumosa*, *Conostegia speciosa*, *Conostegia subcrustulata*, and *Conostegia xalapensis*. The calyptra of these species differs from those of the other two sections in that they lack sclereids. Additional characteristics of section *Geniculatae* include the filament geniculation, the exserted style (in most of its species), small flower size, diplostemonous flowers of most of its species, and the frequently papillose seed testa. Lastly, it is noteworthy that many of the species in this section have leaves that are strongly plinerved and frequently asymmetric. Also, see the Biogeography section for remarks on the marked geographical patterns at the sectional level.

### Chromosome numbers

Chromosome counts have been reported for the following 13 species of *Conostegia*: *Conostegia arborea*, *Conostegia colliculosa*, *Conostegia consimilis*, *Conostegia galdamesiae*, *Clidemia hammelii*, *Conostegia icosandra*, *Conostegia montana*, *Conostegia oerstediana*, *Conostegia setosa*, *Conostegia subcrustulata*, *Conostegia superba*, *Conostegia schlimii*, and *Conostegia xalapensis* (Solt and Wurdack 1980, Almeda and Chuang 1992, Schnell 1996, Almeda 2013). In all reported counts the haploid number was *n*=17, which is believed to be the base number of the tribe Miconieae (Almeda 2013).

### Biogeography

Of the three main clades (sections) of *Conostegia*, the smallest one, *Australis*, is noticeable for its species being primarily South American, i.e., *Conostegia apiculata*, *Conostegia centronioides*, *Conostegia dentata*, *Conostegia extinctoria*, *Conostegia lancifolia*, *Conostegia ortizae* and *Conostegia rubiginosa*. This is almost the only clade to contain species endemic to South America except for *Conostegia ecuadorensis* and *Conostegia foreroi* of section *Geniculatae*. Other species of section *Australis* (e.g. *Conostegia lasiopoda* and *Conostegia tenuifolia*) reach southern Central America and are common in that region, but none of the species in section *Australis* ranges beyond Nicaragua and none are present in the Caribbean. The only species of section *Australis* to reach an oceanic island is *Conostegia lasiopoda*, which occurs on Cocos Island in the Pacific Ocean.

Section *Conostegia* is mostly restricted to Central America and the Caribbean. Roughly speaking there are three distinct areas where endemic species of this section occur. The area with the most endemics is southern Central America (Costa Rica and Panama). The mountains in these two countries include the volcanic ranges in Costa Rica and the Talamanca mountains that start in Costa Rica and end in Panama, harboring endemics such as *Conostegia bigibbosa*, *Conostegia chiriquensis*, *Conostegia fragrantissima*, *Conostegia macrantha*, *Conostegia micrantha*, *Conostegia muriculata*, *Conostegia oerstediana*, *Conostegia pittieri*, *Conostegia rhodopetala*, and *Conostegia vulcanicola*. Some of these species reach their northernmost distribution on volcanoes of Nicaragua and some also reach the lowlands of these three countries. The second area of endemism for species of section *Conostegia* is in northern Central America, both in the mountains of southern Mexico as well as in some lower-elevation and drier valleys. Some of these northernmost endemics include *Conostegia arborea*, *Conostegia caelestis*, and *Conostegia jaliscana*. Lastly the third area of endemism, which could potentially be divided into two, are the Caribbean islands of Hispaniola and Cuba (where the endemic *Conostegia lindenii* grows), and the island of Jamaica where three endemics occur (*Conostegia balbisiana*, *Conostegia procera*, and *Conostegia pyxidata*).

Section *Geniculatae* stands out biogeographically because most of its species (80%) are endemic to the southern Central American countries of Costa Rica and Panama. The rest of the species occur in northern Central America with endemics of that area including *Conostegia fulvostellata*, *Conostegia oligocephala*, and *Conostegia plumosa*. Other species of section *Geniculatae* (e.g. *Conostegia speciosa*, *Conostegia subcrustulata*, and *Conostegia xalapensis*) are more-or-less widespread in Central America reaching South America. Just like in section *Australis*, only one species of section *Geniculatae* (i.e., *Conostegia ombrophila*) reaches an oceanic island, i.e. Cocos Island in the Pacific.

Ongoing work in the tribe Miconieae to obtain a dated phylogeny will provide a time calibrated hypothesis that will enable a thorough biogeographical analysis of *Conostegia*.

### Natural history

#### Herbivory

**Figure 2.**
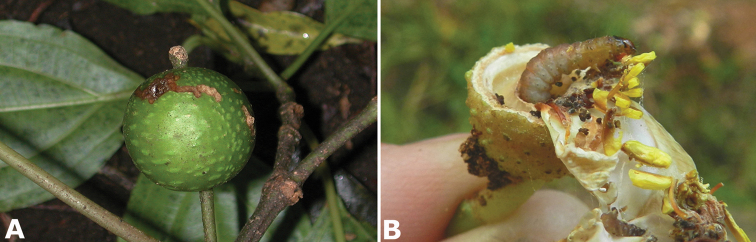
Examples of galls in *Conostegia*. **A** A stem gall of a Lepidopteran in the family Momphidae in *Conostegia oerstediana* **B** A caterpillar of the family Momphidae that apparently can form ovary galls in *Conostegia macrantha*.

**Figure 3.**
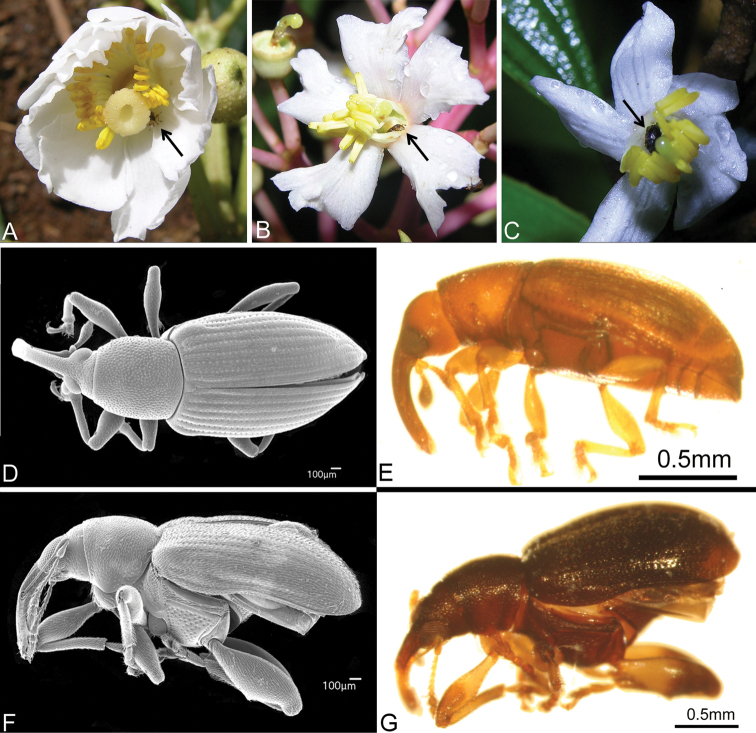
Beetles of the family Curculionidae in *Conostegia* flowers. **A** *Conostegia oerstediana* **B** *Conostegia rhodopetala* **C** *Conostegia xalapensis* **D, F** Scanning electron micrographs of two weevils found in *Conostegia* flowers **E, G** Two weevils from material in spirit.

Many insects especially in the orders Coleoptera, Homoptera, Hymenoptera, and Lepidoptera have been documented interacting with species of *Conostegia*. Herbivory mostly of the leaves of species of *Conostegia* by larvae of Lepidoptera has been well documented in Costa Rica. The data base of lepidopteran herbivores of Janzen and Hallwachs (2013) from northern Costa Rica includes around 4420 records of 134 Lepidoptera species in 84 genera of 27 families reared from species of *Conostegia*. *Conostegia xalapensis* stands out for the large number of species feeding on it. Other lepidopteran species can induce galls such as as species in the family Momphidae (Fig. 2). Hymenopterans have been documented parasitizing larvae in some of these galls, and some have been named after *Conostegia*. *Chrysonotomyia conostegiae* Hansson parasitizes Momphidae larvae in galls of *Conostegia xalapensis* as well as larvae of gall midges (Cecidomyiidae) (Hansson 2004). Another hymenopteran herbivore also named after *Conostegia* is *Allorhogas conostegiae* Marsh and Shaw (Chavarría et al. 2009). This is one of the few phytophagous braconid wasps that has ever been reported (Chavarría et al. 2009). It was reared from the fruits of *Conostegia xalapensis*. During this study, beetles of the family Curculionidae were frequently encountered in flowers of *Conostegia* species. As a result of pickling flowers in the field, several of these beetles remained at the bottom of the pickle jars of different species (Fig. 3). The beetles appear to be of the genus *Phylothrox* sp. (Curculioninae: Acalyptini) (Franz 2006). Other curculionid beetles that have been reared from *Conostegia* include one species of the genus *Anthonomus* from *Conostegia oerstediana* (Chacón-Madrigal et al. 2012). In another family of the beetles, the Chrysomelidae, two species of the genus *Margaridisia* have been found, one in *Conostegia schlimii* and one in *Conostegia xalapensis* (Flowers and Janzen 1997). From these studies it is clear that much is to be learned about *Conostegia* species and their herbivores.

#### Floral biology

Floral longevity in the Melastomataceae has not been thoroughly studied. Stratton (1989) documented floral longevity in Monteverde, Costa Rica, and included nine species of Melastomataceae. He found the mean floral longevity for the family to be 1.24 days. This study included three species of *Conostegia*. This short floral life span is expected in flowers of the family because most are buzz pollinated (Renner 1989). Plants that are buzz pollinated tend to be homogamous (offer pollen reward at the same time they receive it) because the lack of rewards such as nectar inhibit them from being dichogamous. Dichogamous plants such as protandrous ones that reward nectar can change the position of the sexual organs through time and offer a reward in both male and female phases. Because melastomes are generally not dichogamous, their flowers need not live as long as those that change sexual expression.

Floral scent has been noted on several specimen labels of several species of *Conostegia*. To obtain some descriptive statistics on scent production within *Conostegia*, I compiled information from both the Instituto Nacional de Biodiversidad, Costa Rica (INB) and The New York Botanical Garden herbarium for all Melastomataceae. In total, about 52,000 specimens were databased which had label information. Of this total, 389 specimens had some report of fragrance after searching with key words “olor”, “odor”, “scent”, “aroma”, “fragr”, and “cheiro”. Some 60 of the specimens with some report of scent are in the *Conostegia*.

Schnell (1996) noted that some species such as *Conostegia montana* and *Conostegia oerstediana* can have fragrant or odorless populations. Indeed more thorough observations are needed to assess the systematic significance and distribution of floral scent in *Conostegia*. Also, for taxa with scented flowers, it remains to be determined where the scent is being emitted. I have perceived strong pleasant scent emission in flowers of *Conostegia cuatrecasii*, *Conostegia macrantha* and *Conostegia oerstediana*.

#### Reproductive biology

Within *Conostegia*, at least two species are known to be self-compatible, *Conostegia montana* (Tanner, 1982) and *Conostegia oerstediana* (Schnell, 1996) (Table 1). Another species (*Conostegia macrantha*) is reported to be self-incompatible (Renner 1989). The main way in which species within *Conostegia* are thought to avoid self pollination is through herkogamy (Renner 1989). Herkogamy is the spatial separation of sexual parts within flowers and is usually manifested within *Conostegia* by its species having exserted styles. This type of herkogamy falls within the “homomorphic” type of flower in the classification of Webb and Lloyd (1986). Within the homomorphic type they belong to the “ordered” type in which there is usually a single contact between the pollinator with the stigma and stamens. Lastly, within the “ordered” type, flowers with exserted styles fall in the “approach” category in which the stigma is placed forward in the pollinator’s path. Two species, *Conostegia fragrantissima* and *Conostegia pittieri* are unusual in that they appear always to have their style bent opposite the stamens. A third species *Conostegia schlimii* also can have its style bent away from the stamens. Although this position of the style is uncommon in *Conostegia*, it is present in several other groups of Melastomataceae (e.g. Kriebel 2015). The evolution of the giant stigma appears to correspond to a different kind of flower altogether. Taxa with giant stigma tend to have large flowers with spreading petals and with the large stigma probably functioning as a large bullseye for the bee to touch when approaching the flower. This kind of flower fits those termed “dish-blossoms” in the classification of Webb and Lloyd (1986), characterized by having many stamens and a large central stigmatic region. They were considered as having “homomorphic, ordered type” flowers like the others but which may be of the “unordered” type in which the pollinator may contact the stigma and anthers several times and in no particular order. It should be noted that there is a second hypothesis (besides avoiding self pollination and promoting outcrossing) for the evolution of herkogamy (Webb and Lloyd 1986). This second hypothesis proposes that herkogamy evolved as a way to avoid interference between the function of the stamens and that of the style (Webb and Lloyd 1986; Fetscher 2001; Barrett 2002).

**Table 1.** Pollinators and breeding systems in *Conostegia*.

Species	Family	Genus	Species	Compatibility system	Source
*Conostegia bracteata*	Halictidae	*Augochlora*	*Augochlora* sp.	?	R. Kriebel pers. obs.
*Conostegia bracteata*	Apidae	*Euglossa*	*Euglossa* sp.	?	R. Kriebel pers. obs.
*Conostegia cinnamomea*	Apidae	*Melipona*	*Melipona cf costaricensis*	?	R. Kriebel pers. obs.
*Conostegia consimilis*	Halictidae	*Augochlora*	*Augochlora* sp.	?	R. Kriebel pers. obs.
*Conostegia grayumii*	Halictidae	*Augochlora*	*Augochlora* sp.	?	R. Kriebel pers. obs.
*Conostegia macrantha*	?	?	?	SIC	Renner 1989
*Conostegia montana*	?	?	?	SC	Tanner 1980
*Conostegia oerstediana*	Apidae	*Bombus*	*Bombus volluceloides*	SC	R. Kriebel pers. obs.; Schnell 1996
*Conostegia ortizii*	Halictidae	cf *Augochlora*	cf *Augochlora* sp.	?	D. Penneys 1857
*Conostegia pittieri*	Apidae	*Bombus*	*Bombus volluceloides*	?	R. Kriebel pers. obs.
*Conostegia schlimii*	Apidae	*Melipona*	*Melipona cf costaricensis*	?	R. Kriebel pers. obs.
*Conostegia setosa*	Halictidae	*Augochlora*	*Augochlora* sp.	?	R. Kriebel pers. obs.
*Conostegia subcrustulata*	Apidae	*Melipona*	*Melipona cf costaricensis*	?	R. Kriebel pers. obs.
*Conostegia xalapensis*	Apidae	*Melipona*	*Melipona beechei* and *Melipona fasciata*	SC	J. A Reed pers. obs.
*Conostegia* sp.	Apidae	*Melipona*	*Melipona panamica*	?	D. Roubik pers. comm.
*Conostegia* sp.	Halictidae	*Augochlora*	*Augochlora* sp.	?	D. Roubik pers. comm.

All species within section *Conostegia* and a few within section *Geniculatae* have consistently short styles. Flowers that lack a distance between the anthers and the stigma may be called non-herkogamous flowers. Recently the term plesiogamous was proposed for this flower type (Nesom 2012). This term derives from the Greek, *plesios*, meaning near, alluding to the proximity of the stamens and stigma (Nesom 2012). The evolution of plesiogamy has occurred at least twice within *Conostegia* (Kriebel et al. 2015) and is known to have evolved in other clades of Melastomataceae (e.g., Miconia section Hartigia).

**Figure 4.**
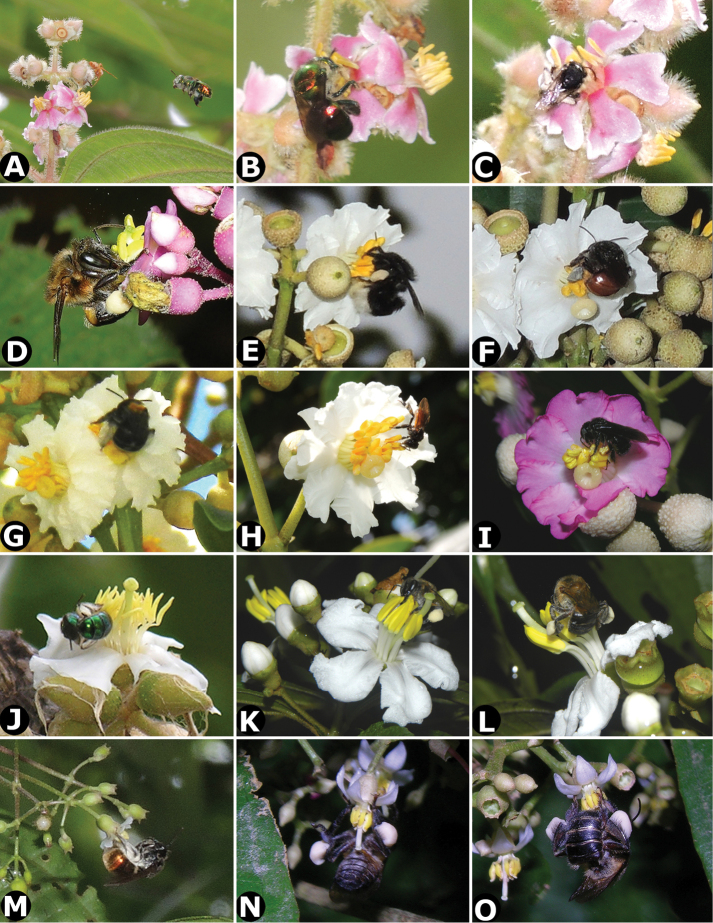
Bee pollinators and pollen robbers in *Conostegia*. **A** Female of the genus *Euglossa* approching an inflorescence of *Conostegia bracteata* **B** *Euglossa* sp. buzzing the flower of *Conostegia bracteata* **C** Unidentified female of a species of Halictidae buzzing the flower of *Conostegia bracteata* **D** Female of *Melipona costaricensis* buzzing flower of *Conostegia subcrustulata* **E** Female of *Bombus volluceloides* buzzing the flower of *Conostegia oerstediana* **F, G** Unidentified large bees buzzing the flowers of *Conostegia oerstediana* **H** A species of *Trigona* robbing pollen from a flower of *Conostegia oerstediana* **I** A species of *Trigona* robbing pollen from a flower of *Conostegia bigibbosa* **J** Unidentified female of a species of Halictidae buzzing the flower of *Conostegia ortizae*. Photograph by Darin Penneys **K, L** Female of *Melipona costaricensis* buzzing flower of *Conostegia schlimii*. Photographs by Reinaldo Aguilar **M** Unidentified female of a species of Halictidae buzzing the flower of *Conostegia grayumii* **N, O** Unidentified female of a species of Halictidae buzzing the flower of *Conostegia cinnamomea*. Photos by the author if not specified.

Little has been published on the pollination of *Conostegia*. Renner (1989) did not report any bee observations for *Conostegia* in her review of melastome reproductive biology. However, because of their poricidal yellow anthers they are all thought to be buzz pollinated, and some observations of buzz pollination are available (Schnell 1996). Throughout the present study, photographs of potential pollinators were gathered in the following Costa Rican localities (province in parenthesis): Escazú (San José), San Miguel Arriba de Grecia (Alajuela), La Selva Biological Station (Heredia), and the Osa Peninsula (Puntarenas). In the larger flowered taxa which tend to occur at higher elevations, bees of the genus *Bombus* as well as an unidentified genus were observed buzzing the flowers of *Conostegia pittieri* and *Conostegia oerstediana*. At lower elevations, bees of the genus *Melipona* were observed buzzing the flowers of *Conostegia cinnamomea*, *Conostegia schlimii*, and *Conostegia subcrustulata*. Also at lower elevations, a species of *Auglochlora* (family Halictidae) was observed buzzing the flowers of *Conostegia bracteata*, *Conostegia consimilis*, *Conostegia grayumii*, and *Conostegia setosa*. Photographs taken in Ecuador by Darin Penneys of a flowering tree of *Conostegia ortizae* also show a halictid bee buzzing the flowers. In the lowlands of Costa Rica, species in the orchid bee genus *Euglossa* have been observed buzzing the flowers of *Conostegia bracteata* and *Conostegia subcrustulata* (Fig. 4, Table 1). During this study it was observed that in small flowered species with exserted styles such as *Conostegia cinnamomea* and *Conostegia grayumii*, the bees cover the stigma with their abdomen before buzzing the flower (Fig. 4). These taxa tend to have their flowers facing downward which appears to force the bees into this position. On the other hand short styled species such as *Conostegia bracteata* and *Conostegia subcrustulata* have their flowers positioned horizontally and the bees grasp the stamen but do not cover the stigma. In large flowered species such as *Conostegia oerstediana*, the flower tends to be either upright or horizontally positioned and the stigma tends to bend downwards potentially being landed on or touched by the abdomen of large bees. Pollen thieves of the genus *Trigona* were also encountered both eating the anthers as well as sticking their tongues into the anther pores of *Conostegia oerstediana* (Fig. 4). Pollen robbing by *Trigona* bees has been well documented in the Melastomataceae (Renner 1983). In summary, the poricidal anther dehiscence, and the observation of only bees visiting flowers of species in all three of the major clades of *Conostegia* suggests that most if not all of the species are buzz pollinated by bees.

#### Dispersers

Seeds of species of *Conostegia* are mostly dispersed by birds (Schnell 1996). Seed dispersal in *Conostegia volcanalis* by the rodents *Peromyscus aztecus* and *Reithrodontomys fulvescens* has also been reported (Vázquez et al. 2004). Two cases of fruit dispersal by bats have also been documented, one by *Hylonycteris underwoodi* dispersing *Conostegia xalapensis* (Castro-Luna and Sosa, 2009) and one by an unidentified species of bat dispersing *Conostegia oerstediana* (Harvey, 2000). Bats are thought not to be important dispersal agents of melastomes (Schnell 1996).

Of the known bird-dispersed species, Harvey (2000) documented sixteen species of birds feeding on *Conostegia oerstediana* and one species feeding on *Conostegia xalapensis* in Monteverde, Costa Rica. Loiselle and Blake (1990) found seeds of *Conostegia subcrustulata* to be the most common in fecal samples of birds in second-growth forest at La Selva, Costa Rica. Loiselle and Blake (1990) also found that red-capped manakins, white collared manakins and scarlet rumped tanagers to have a special preference for the fruits of *Conostegia subcrustulata*. To attract birds dispersers, species of *Conostegia* have purple berries that contrast with the foliage (Schnell 1996). Some species such as *Conostegia monteleagreana* and *Conostegia rhodopetala* have red to pink inflorescence branches that may provide further contrast with the color of the berries. The display of the berries in taxa with terminal inflorescences contrasts with that of taxa with axillary inflorescences. These two different types of fruit display result in different guilds of bird dispersers (Schnell 1996).

Schnell (1996) noted that the presence of berries on individuals of *Conostegia* is infrequent both in herbarium specimens and in the field. He suggested that the reason for this pattern might have to do with optimizing limited resources through selective fruit abortion, as has been documented in many families of flowering plants (Stephenson 1981). Then, the few berries that are actually produced are quickly dispersed.

Schnell (1996) recognized two fruiting patterns in *Conostegia*. In the first one, plants produce berries directly after flowering. This behavior results in the presence of flowers and fruits on the same inflorescence/infructescence. Species with this fruiting type include *Conostegia lasiopoda*, *Conostegia macrantha*, *Conostegia montana*, *Conostegia rhodopetala*, *Conostegia setosa*, and *Conostegia subcrustulata*. The second type of fruiting behavior is that in which fruiting occurs several months after flowering. Examples of species with this second type of fruiting behavior are *Conostegia pittieri* and *Conostegia rufescens*.

Lastly, Schnell (1996) hypothesized that there are three possible advantages gained from bird dispersal: (1) displacement of seeds over a range of distances; (2) improved habitat selection; and (3) enhanced seed germination after passage of seeds through the gut. With respect to the first hypothesis, he found evidence that dispersal distances are modest. This is because retention of seeds by birds of species with small berries and small seeds tends to be short (Levey 1987). As for the second hypothesis of reaching suitable sites, Schnell (1996) could not find support, but noted that little is known about the behavior of birds after fruit ingestion. Lastly, Schnell (1996) found some evidence to suggest that germination is not enhanced by passage of seeds through the birds gut as demonstrated by Ellison et al. (1993).

## Materials and methods

In total about 2000 sheets were studied for this revision from the following herbaria: CAS, CR, INB, NY, PMA, and USCG. In addition, digital images were studied from the following herbaria: BM, COL, F, GH, MO, P, and US. About 665 specimens were collected during the study of the group on several trips to Costa Rica, and one trip each to both Guatemala and Panama. In order to produce maps, many specimens were georeferenced and additional ones identified by experts (mainly Charles Schnell and Frank Almeda) added to improve the distribution maps. The maps and phenology diagrams were made using the R package monographaR (Reginato 2016).

### Leaf and floral anatomy

Leaves and floral buds were fixed in 70% ethanol in the field and at maximum two weeks later were brought into the lab and fixed in formalin-acetic acid-ethanol (FAA; 3.7% formaldehyde; 5% glacial acetic acid; 50% ethanol), vacuum-infiltrated overnight, and then stored in 70% ethanol. For light microscopy, fixed material was dehydrated through an alcohol-toluene series in a Leica TP-1020 automatic tissue processor, and embedded in Paraplast X-tra (Fisher Healthcare, Houston, Texas, USA). The samples were sectioned at 10 µm with an AO Spencer 820 rotary microtome (GMI Inc. Minnesota, USA). Sections were stained with Johansen’s safranin (Johansen 1940) (2% w/v in 50% ethanol) and 0.5% Astra Blue in 2% tartaric acid w/v in distilled water (Maácz and Vágás 1961; Kraus et al. 1998) and mounted in Permount (Fisher Scientific, Pittsburgh, Pennsylvania, USA). Sections were viewed and digitally photographed with a Zeiss Axioplan compound microscope equipped with a Nikon DXM1200C digital camera with ACT-1 software.

### Scanning electron microscopy

To thoroughly document trichomes, floral parts, and seeds, Scanning Electron Microscopy (SEM) was used. For the study of floral parts, flowers collected in the field were brought to the lab and transferred to acetone via an ethanol-acetone series, and then dried by critical point, mounted on aluminum stubs with adhesive tabs (Electron Microscopy Sciences, Hatfield, PA, USA), sputter coated with gold palladium in a Hummer 6.2 sputter coater (Anatech, Springfield, VA, USA), and examined and photographed in a Jeol JSM-5410 LV Scanning Electron Microscope operated at 10 kV. Seeds were cleaned in water prior to sputter coating and SEM.

### Species plates

Both photographs of living plants and dissections of material in spirit are used to illustrate the species treated in this revision. For preserved material, floral structures were photographed under a Nikon SMZ1500 stereoscope equipped with a Nikon DXM1200F camera connected to a computer and using the software ACT-1. The plates were prepared with GIMP (The GNU Image Manipulation Program).

## Results

**Figure 5.**
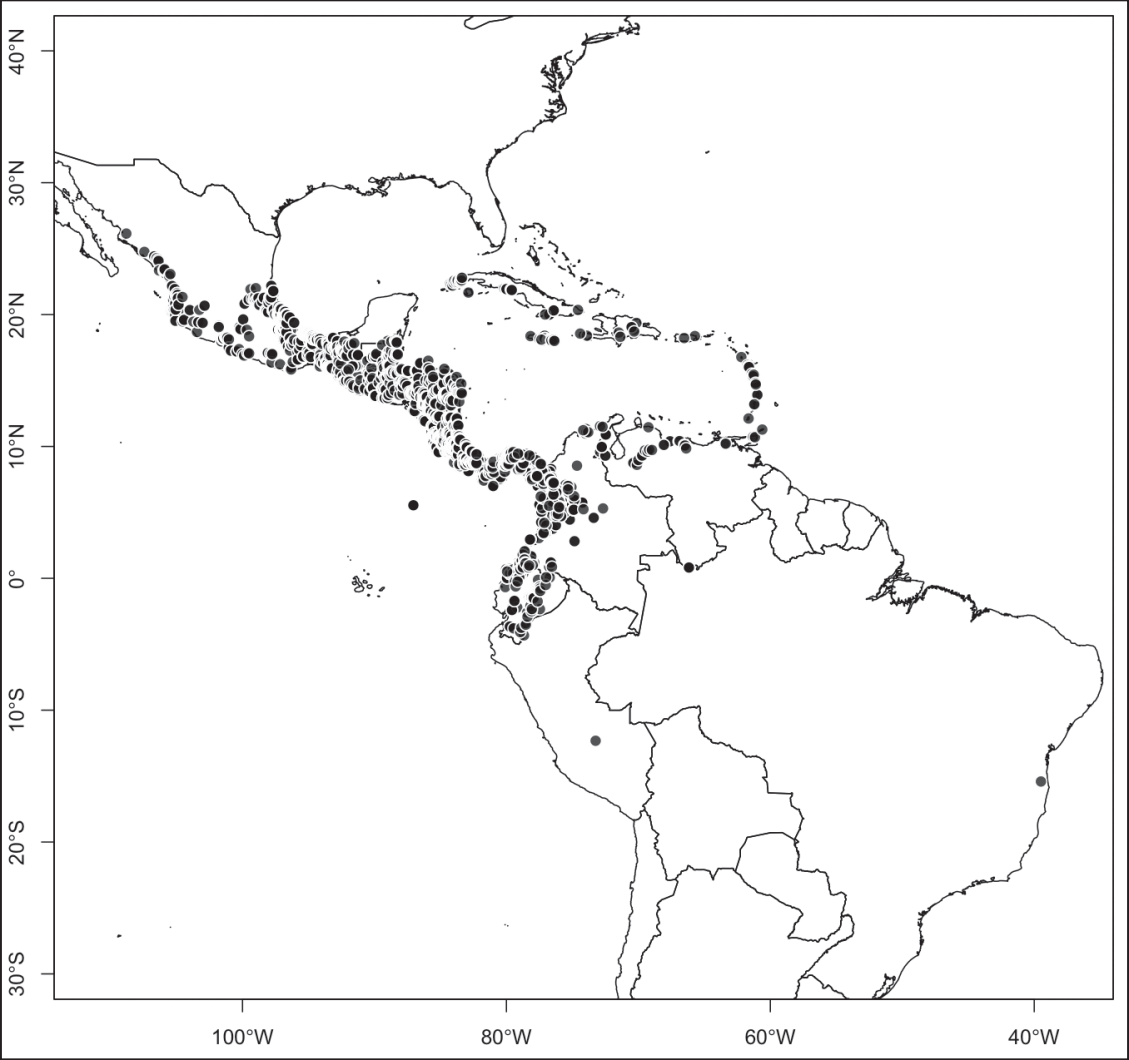
Distribution of *Conostegia*.

**Figure 6.**
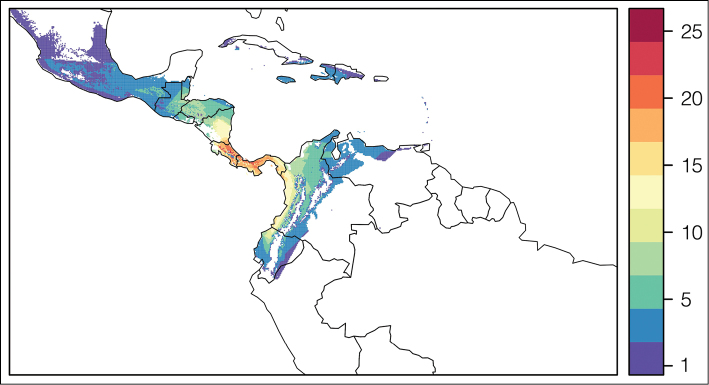
Species density of *Conostegia* per ten square kilometer grid cell size. A couple of known localities of *Conostegia icosandra* from Bahia, Brazil, not shown.

**Figure 7.**
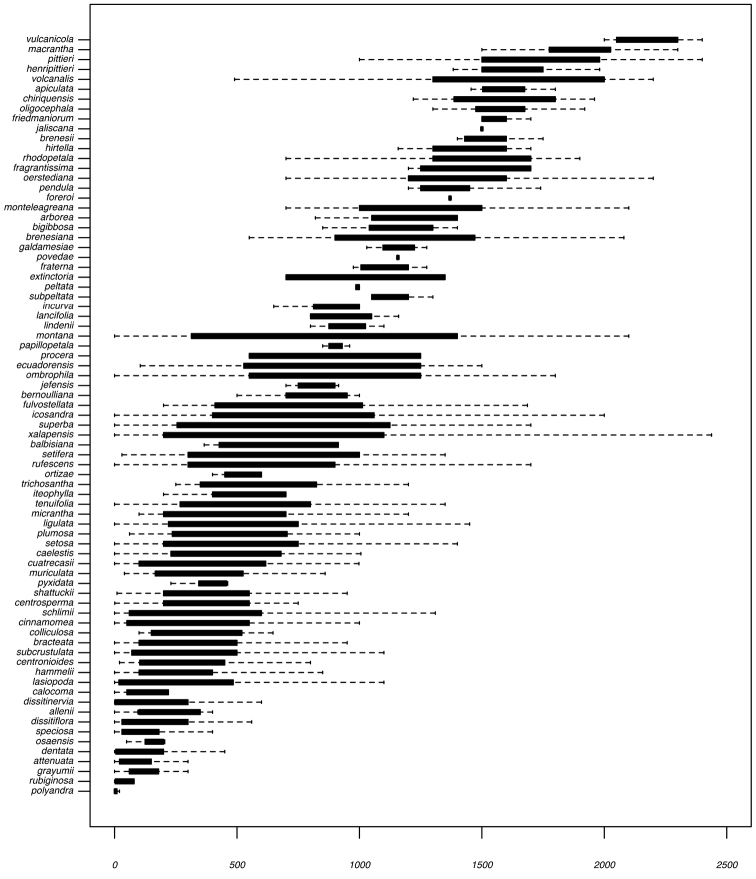
Boxplots of elevation (in meters above sea level) for species of *Conostegia*.

Most species of *Conostegia* are in Central America (Fig. 5) with the highest density being in southern Central America (Fig. 6). I was fortunate to receive material and images of live plants from South American countries as well as the Caribbean Islands, which encompasses the whole range of *Conostegia* except for a disjunct population of *Conostegia icosandra* in Bahia, Brazil. They mostly occur in lowland rainforest and cloud forest habitats. The preferred elevational ranges are shown are shown in Figure 7.

### Flowering phenology

**Figure 8.**
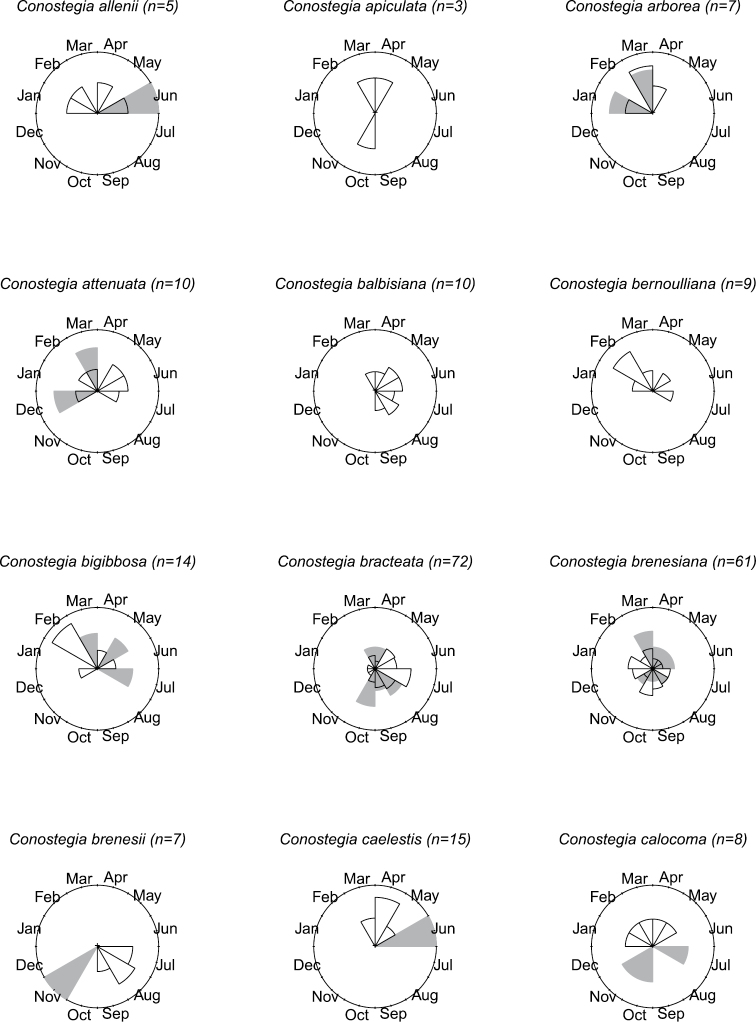
Phenology in species of *Conostegia*. White slices denote flowering specimens and gray slices fruiting specimens. The size of the slice represents the relative amount of specimens from the total which was blooming or fruiting in that month.

**Figure 9.**
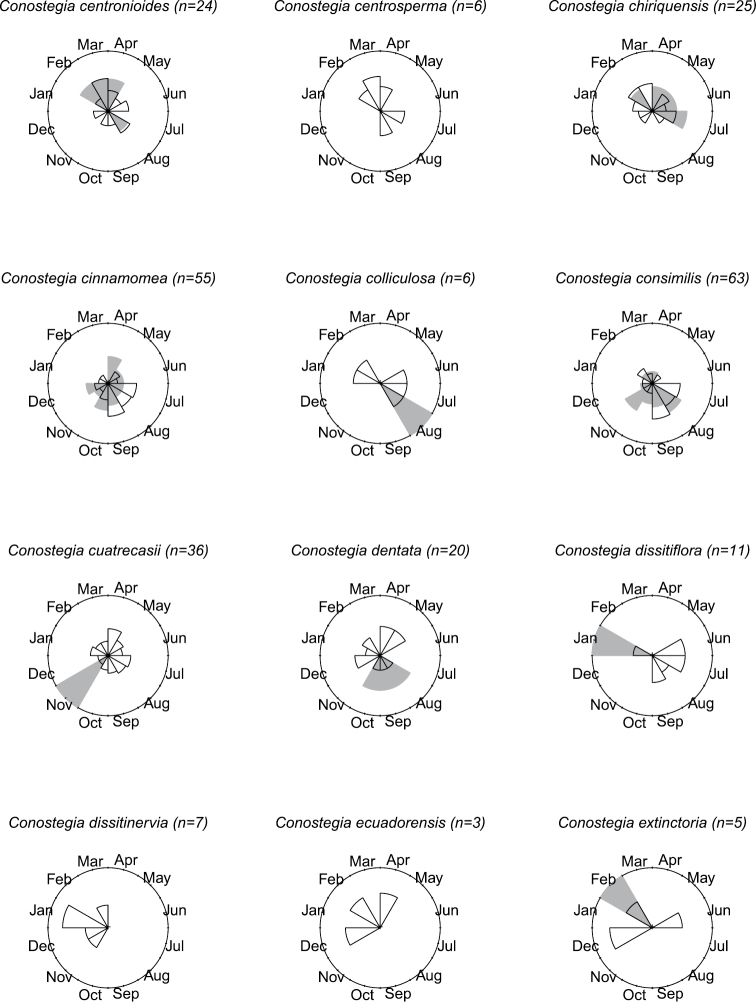
Phenology in species of *Conostegia*. White slices denote flowering specimens and gray slices fruiting specimens. The size of the slice represents the relative amount of specimens from the total which was blooming or fruiting in that month.

**Figure 10.**
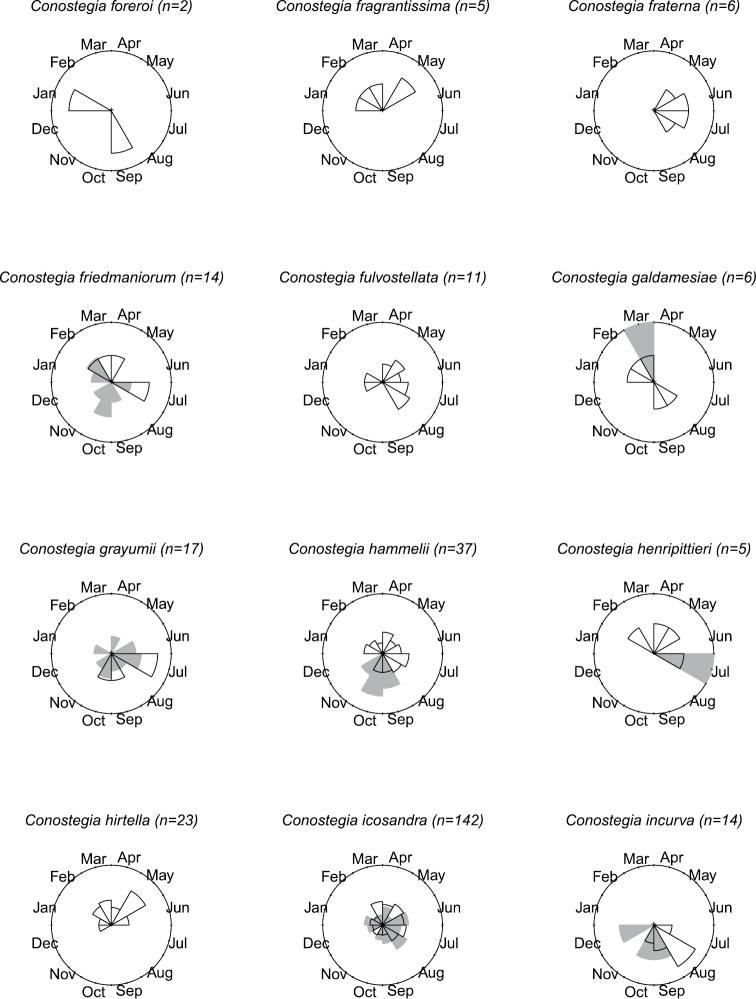
Phenology in species of *Conostegia*. White slices denote flowering specimens and gray slices fruiting specimens. The size of the slice represents the relative amount of specimens from the total which was blooming or fruiting in that month.

**Figure 11.**
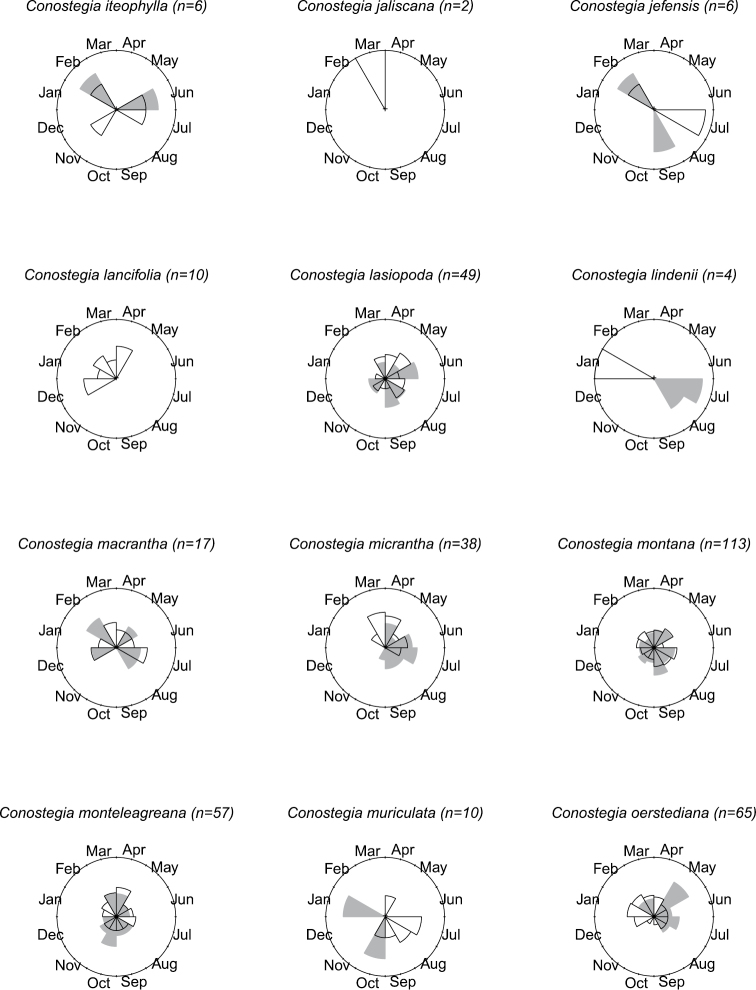
Phenology in species of *Conostegia*. White slices denote flowering specimens and gray slices fruiting specimens. The size of the slice represents the relative amount of specimens from the total which was blooming or fruiting in that month.

**Figure 12.**
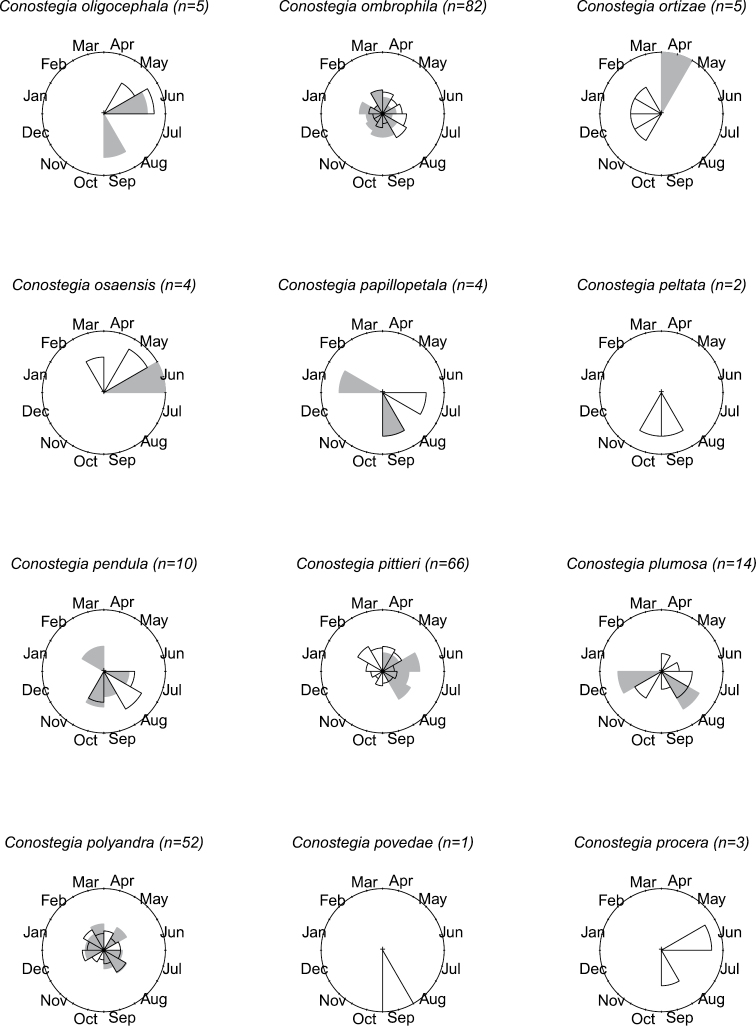
Phenology in species of *Conostegia*. White slices denote flowering specimens and gray slices fruiting specimens. The size of the slice represents the relative amount of specimens from the total which was blooming or fruiting in that month.

**Figure 13.**
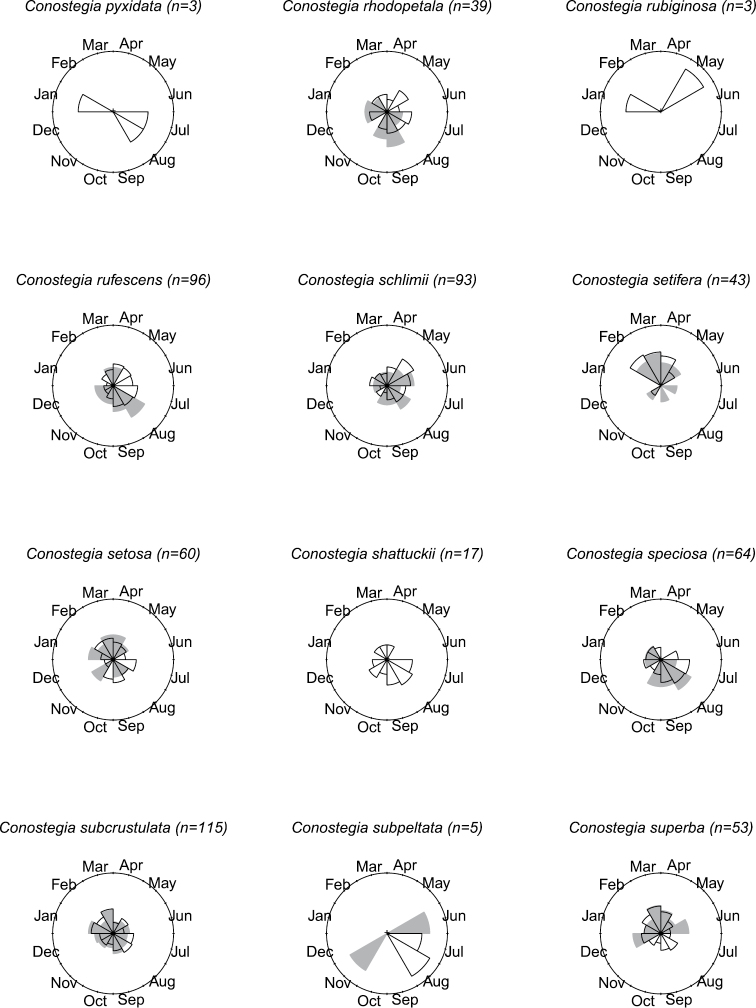
Phenology in species of *Conostegia*. White slices denote flowering specimens and gray slices fruiting specimens. The size of the slice represents the relative amount of specimens from the total which was blooming or fruiting in that month.

**Figure 14.**
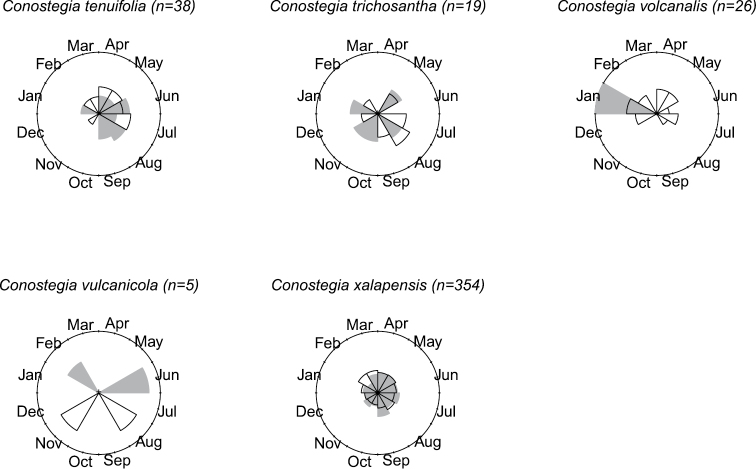
Phenology in species of *Conostegia*. White slices denote flowering specimens and gray slices fruiting specimens. The size of the slice represents the relative amount of specimens from the total which was blooming or fruiting in that month.

The flowering phenology of species of *Conostegia* was tallied using herbarium label data. In total, the flowering time for 1420 specimens was recorded. To best visualize in a comparative manner the phenology of each species, circular histograms were produced (Figs 8–14). This assessment of phenology should be taken with caution since it is based on herbarium specimens which can often be the result of easy accessibility to collecting sites and other collecting biases such as sampling error and good weather conditions. Furthermore, realistic patterns of phenology are obscured for broadly distributed species as well as local patterns, which are best studied at the population level. Schnell (1996) reported a pattern present in species such as *Conostegia bracteata*, *Conostegia montana*, *Conostegia setifera*, and perhaps *Conostegia balbisiana* in which flowering begins around March and finishes in September. Schnell (1996) called this an example of the normal “out-of-season low-frequency flowering” which might be favored in individuals in high light environments. Schnell (1996) also noted the year-long flowering of weedy species and hypothesized that these longer flowering times might be selected for longer fruiting seasons which might permit more colonization. A second possible non-exclusive explanation proposed by Schnell (1996) is that because of their weedy habit they tend to receive more light allowing them more energy for reproduction. Lastly, Schnell (1996) suggested the possibility that there are collection biases and that these habitats are more frequently visited by collectors. In addition, some species show very marked seasonality. For example, *Conostegia caelestis* and *Conostegia osaensis* only flower between March and June. *Conostegia oligocephala* has a similarly narrow pattern being recorded in flower from May to July. *Conostegia brenesii* in the cloud forests of Costa Rica only flowers during the rainy season from July through September. *Conostegia grayumii*, *Conostegia incurva*, *Conostegia pendula*, *Conostegia povedae*, and *Conostegia subpeltata* appear to follow a similar pattern of rainy-season flowering from July through October. Another possible pattern includes *Conostegia allenii* and *Conostegia calocoma* both of which only flower from January through July.

### Habit

Species of *Conostegia* are all terrestrial shrubs or trees. Some of the tallest trees in the tribe Miconieae are found in *Conostegia*, with *Conostegia osaensis* reaching about 25 meters in height. Schnell (1996) noted a bimodal distribution of height classes and growth forms in which species were either large trees able to compete with canopy trees such as (e.g., *Conostegia volcanalis* and *Conostegia rufescens*), or fast-growing shrubs that seldom grow higher than 2–3 m. He further suggested that the shrubby growth habit had evolved more than once. In addition, I have observed *Conostegia rufescens* growing both as a large tree and as a shrub. The latter was observed in Cerro Jefe, Panamá, and in the lower parts of Braulio Carrillo National Park in Costa Rica. In Braulio Carrillo, which harbors forests over a wide elevational range, I also observed *Conostegia rufescens* as a taller tree in the cloud forest. The pattern of tall trees is especially evident in the giant stigma group with a clear independent origin in *Conostegia osaensis* and probably also in *Conostegia schlimii*, which can also become a tall tree. The trunk of some species such as *Conostegia bernoulliana* and *Conostegia oerstediana*, can have flaky bark but in most taxa it is smooth. The smallest species are those previously described in *Clidemia*, such as *Conostegia subpeltata* and *Conostegia trichosantha*, which grow in the understory mostly of cloud forests. Twigs vary from terete and slender to somewhat quadrangular and robust. Particularly the species with thick twigs such as *Conostegia bigibbosa* and *Conostegia macrantha*, tend to have lenticels on the nodes.

### Leaves

Leaves are opposite and generally decussate as is the case in most species in the family. In one species, *Conostegia henripittieri*, they are always strongly anisophyllous. Most species of sections *Australis* and *Conostegia* have leaves with nerved venation and species in section *Geniculatae* tend to be strongly plinerved. Variation in leaf morphology among species is most evident in section *Geniculatae* with one peltate species (*Conostegia peltata*) and a subpeltate species (*Conostegia subpeltata*), as well as species with sessile leaves (*Conostegia dissitiflora*) or strongly attenuate leaf bases (*Conostegia consimilis*). In addition, many species of section *Geniculatae* tend to have asymmetric leaf venation. In *Conostegia grayumii* for example, almost every leaf is asymmetrical at the base.

### Leaf anatomy

**Figure 15.**
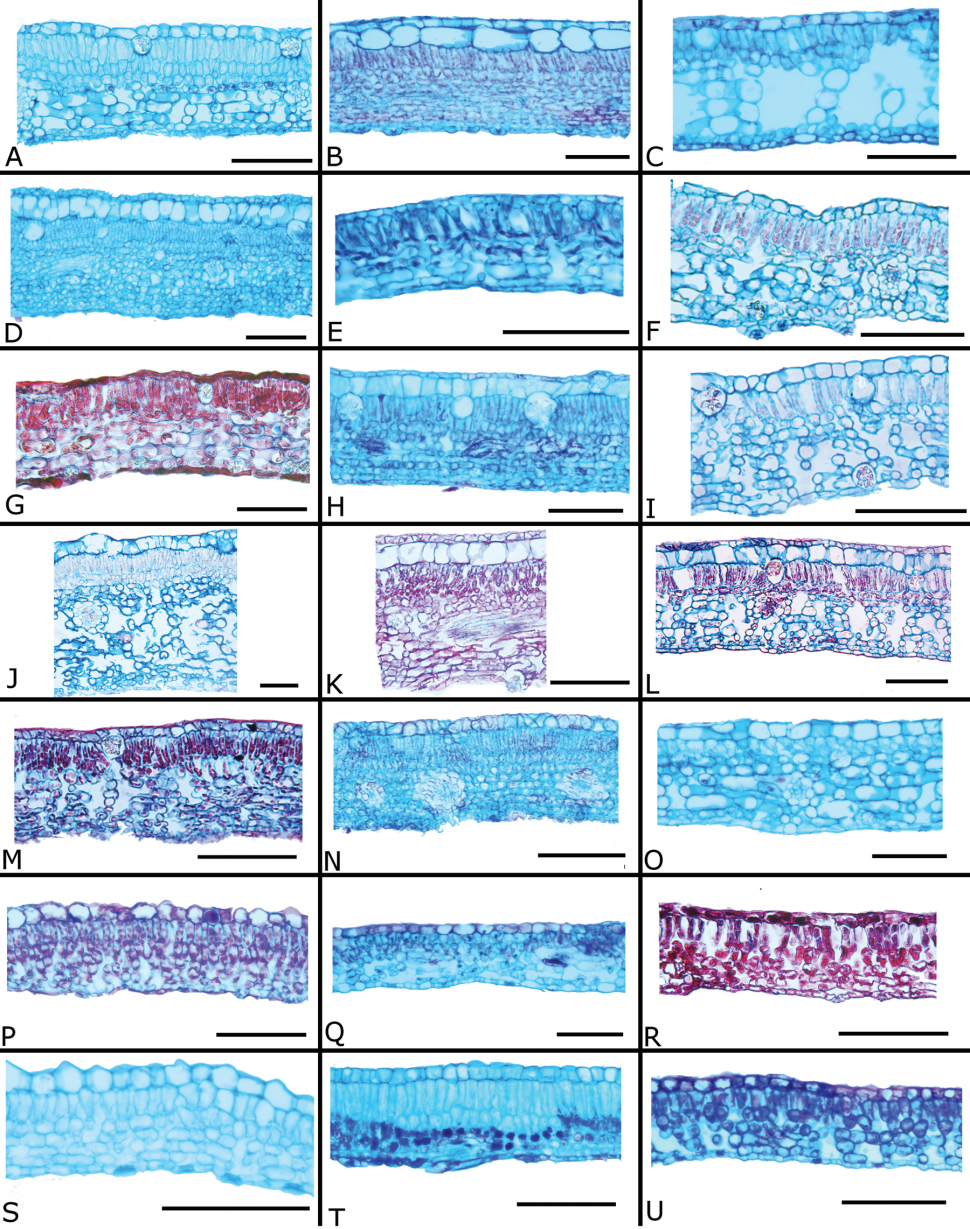
Leaf anatomy in *Conostegia* **A** *Conostegia lasiopoda* (*R. Kriebel 5780*) **B** *Conostegia monteleagreana* (*R. Kriebel s. n.*) **C** *Conostegia tenuifolia* (*R. Kriebel 5773*) **D** *Conostegia bigibbosa* (*R. Kriebel 5771*) **E** *Conostegia bracteata* (*R. Kriebel 5816*). **F** *Conostegia brenesii* (*R. Kriebel 5546*) **G** *Conostegia caelestis* (*R. Kriebel 5617*) **H** *Conostegia bernoulliana* (*R. Kriebel 5772*). **I** *Conostegia montana* (*R. Kriebel 5548*) **J** *Conostegia montana* (*R. Kriebel 5662*) **K** *Conostegia oerstediana* (*R. Kriebel 5627*) **L** *Conostegia pittieri* (*R. Kriebel 5543*) **M** *Conostegia rhodopetala* (*R. Kriebel 5542*). **N** *Conostegia rufescens* (*R. Kriebel 5524*) **O** *Conostegia setosa* (*R. Kriebel 5813*) **P** *Conostegia fraterna* (*R. Kriebel 5774*) **Q** *Clidemia hammelii* (*R. Kriebel 5539*) **R** *Conostegia ombrophila* (*R. Kriebel s.n.*) **S** *Conostegia subcrustulata* (*R. Kriebel 5808*). **T** *Conostegia xalapensis* (*R. Kriebel 5817*) **U** *Conostegia calocoma* (*R. Kriebel s. n.*). Scale bar: 100 µm.

**Figure 16.**
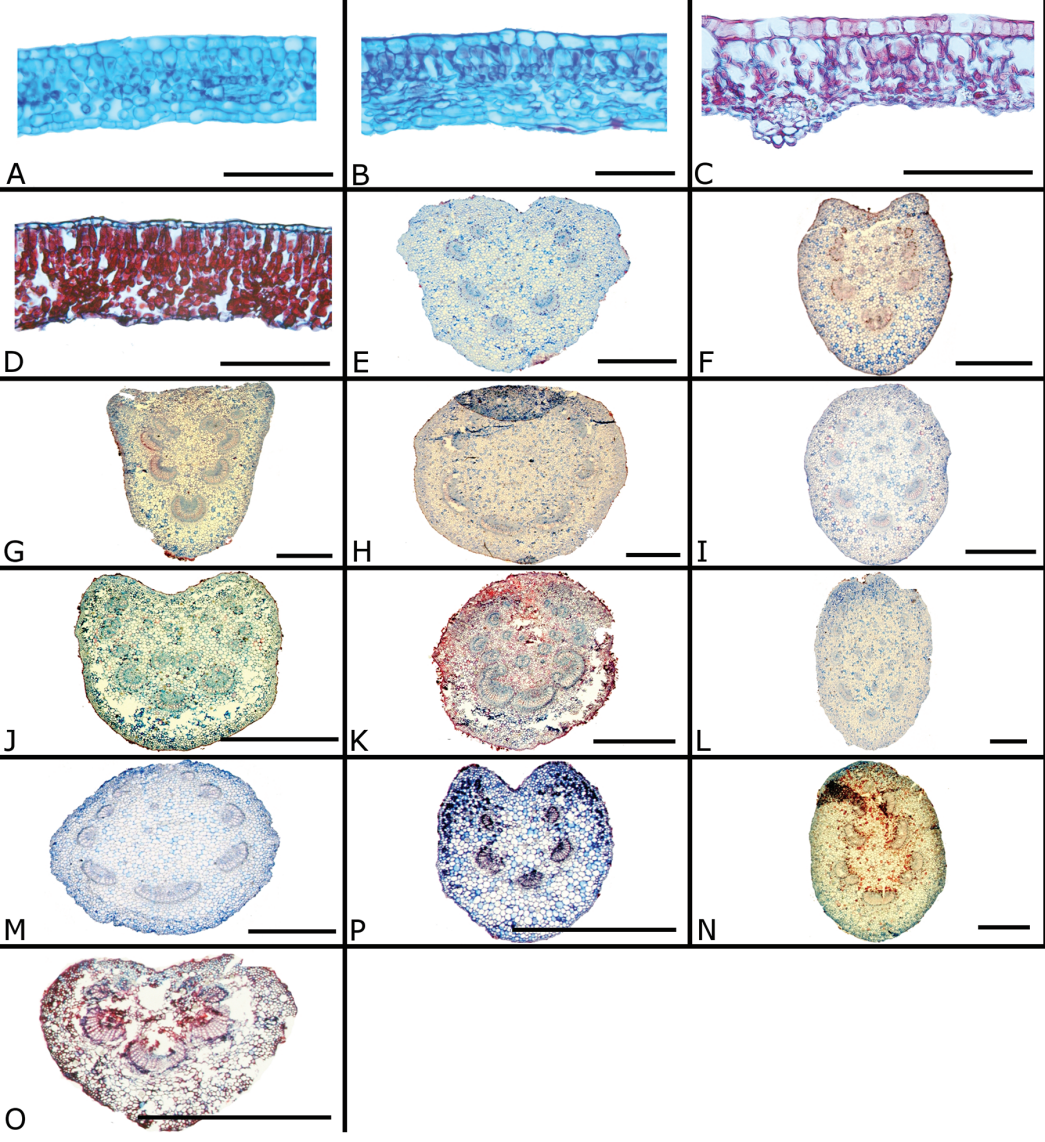
Leaf and petiole anatomy in *Conostegia*. **A** *Conostegia grayumii* (*R. Kriebel 5807*) **B** *Conostegia consimilis* (*R. Kriebel 5811*) **C** *Conostegia oligocephala* (*R. Kriebel 5575*) **D** *Conostegia schlimii* (*R. Kriebel 5776*) **E** *Conostegia lasiopoda* (*R. Kriebel 5780*) **F** *Conostegia tenuifolia* (*R. Kriebel 5773*) **G** *Conostegia monteleagreana* (*R. Kriebel s. n.*) **H** *Conostegia bigibbosa* (*R. Kriebel 5771*). **I** *Conostegia bracteata* (*R. Kriebel 5816*). **J** *Conostegia bernoulliana* (*R. Kriebel 5772*) **K** *Conostegia rufescens* (*R. Kriebel 5524*). **L** *Conostegia setosa* (*R. Kriebel 5813*) **M** *Conostegia subcrustulata* (*R. Kriebel 5808*). **N** *Conostegia grayumii* (*R. Kriebel 5807*) **P** *Conostegia consimilis* (*R. Kriebel 5811*) **O** *Conostegia schlimii* (*R. Kriebel 5776*). Scale bar: 100 μm (**A–D**); 1 mm (**E–O**).

**Figure 17.**
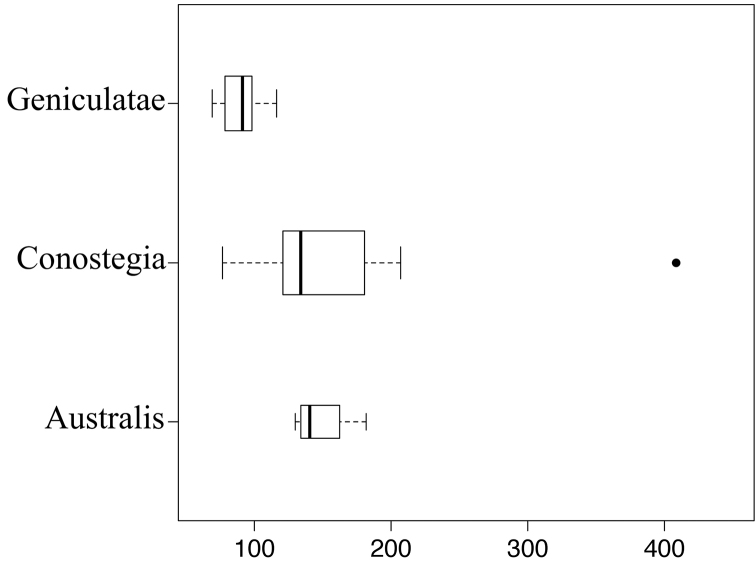
Boxplots of leaf thickness by section of *Conostegia*. The outlier in section *Conostegia* is a population of *Conostegia montana* from Cerro Jefe, Panama. Units in microns.

Thirty leaves of 26 species within *Conostegia* were collected in the field, fixed in FAA, and then sectioned as explained above. Leaves of all *Conostegia* studied are hypostomatic. The cuticle is generally inconspicuous but sometimes can be relatively thick as in *Conostegia rhodopetala* (Fig. 15). The mesophyll is dorsiventral (Figs 15, 16), and druses were present in all species, usually near the adaxial or abaxial leaf epidermis. The size of the druses varies. Some species such *Conostegia tenuifolia* have more space in the mesophyll. Leaf thickness varied but in general leaves were thinner in section *Geniculatae* (Fig. 17). At least two types of coriaceous leaves were found. Populations of *Conostegia montana* from Cerro Jefe, Panama, have a very thick mesophyll and are the most evidently coriaceous leaves encountered in this survey. The other thick-leaved species were found to have a hypodermis one cell layer thick. This hypodermis is found only in *Conostegia montelagreana* in section *Australis* and in the species of the giant stigma clade (species sampled were *Conostegia bigibbosa*, *Conostegia icosandra*, *Conostegia oerstediana*, and *Conostegia pittieri*) where it appears to have evolved in their common ancestor (Fig. 15). Mentink and Baas (1992) reported the presence of a hypodermis in *Conostegia subcructulata* but sections done for this study failed to locate a hypodermis in that this species (Fig. 16). In the Melastomataceae the function of the hypodermis has been suggested to be related to water storage in epiphytic species (Reginato et al. 2009) or protecting the palisade parenchyma against solar radiation in terrestrial species (Ely et al. 2005). The spongy mesophyll was usually not lignified except in some species, such as *Conostegia ombrophila* and *Conostegia schlimii*.

The petioles in *Conostegia bigibbosa* and some Costa Rican and Guatemalan populations of *Conostegia montana* have two protuberances at the apex of the petiole on the abaxial surface. Scanning electron micrographs of these protuberances evidenced a glabrous area with some stomata suggesting that they might be extrafloral nectaries. However, anatomical sections in *Conostegia bigibbosa* did not indicate the presence of carbohydrates. Cross sections of petioles show variation in shape from rounded to somewhat heart shaped, grooved or flat adaxially as in *Conostegia consimilis*, *Conostegia schlimii*, *Conostegia tenuifolia*, and *Conostegia bernoulliana* (Fig. 16). Five to nine amphicribal bundles are present, forming an interrupted arc. The lowermost bundles tend to be larger as in *Conostegia rufescens*, *Conostegia subcrustulata* and *Conostegia schlimii*, (Fig. 16). Smaller bundles are sometimes present inside the primary arc as in *Conostegia bernoulliana*, *Conostegia bracteata*, *Conostegia rufescens*, and *Conostegia tenuifolia* (Fig. 16). Petioles are mostly unlignified but sclereids are present in some species, but besides the sclereids, petioles were mostly unlignified. Some lignification of the petiole was observed in *Conostegia rufescens* and *Conostegia schlimii*.

### Leaf domatia

**Figure 18.**
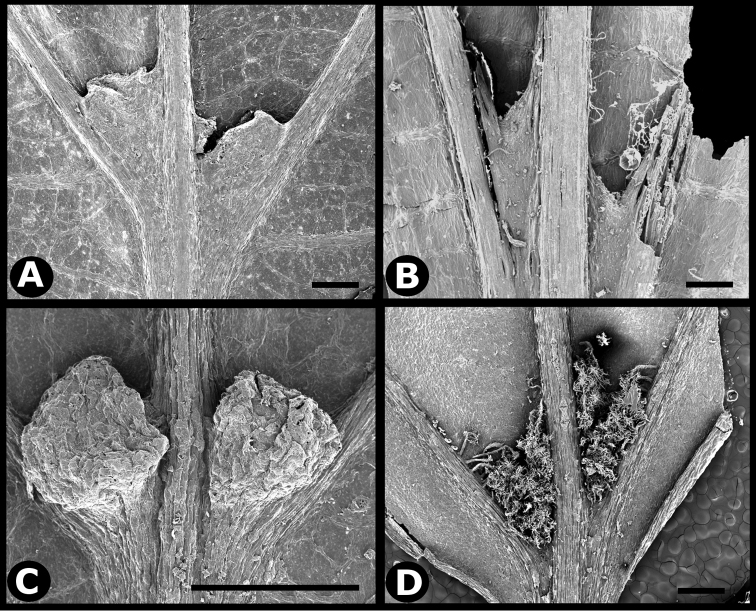
Types of domatia in *Conostegia*. **A, B** examples of pocket domatia **C** example of vesicular pocket domatium **D** example of tuft domatium **A** *Conostegia ecuadorensis* (*J. Betancur 3202*) **B** *Clidemia hammelii* (*L. Acosta 133*). **C** *Conostegia ombrophila* (*R. Kriebel 5730*) **D** *Conostegia procera* (*W. Maxon 8949*). Scale bar: 1 mm.

Three types of domatia occur in *Conostegia*. In two species, *Conostegia dentata* and *Conostegia setosa*, the domatia are of the formicarium type which house ants and are manifested as large swellings at the base of the leaf. The other two types are both mite domatia, present at the base of the leaf on the abaxial side at the point of divergence between the midvein and the primary lateral veins. The general classification of domatia used here follows Jacobs (1966). The first kind of mite domatium is the tuft mite domatium present in *Conostegia procera* (Fig. 18). This species always has these structures, which are densely covered by stipitate stellate to dendritic trichomes. This type of domatium is also seen in some but not all specimens of *Conostegia hirtella*. The second type of mite domatium is the pocket domatium which literally looks like a pocket formed by a membrane (Fig. 18). Some pocket domatia are further called vesicular domatia because they are inflated. Pocket domatia are present in only the clade comprising *Conostegia ecuadorensis*, *Clidemia hammelii*, and *Conostegia ombrophila*. Among the species having pocket domatia, *Conostegia ecuadorensis* is unique in that there are domatia present on both the innermost pair of primary lateral veins as well as on the outermost pair of lateral veins.

### Indument

**Figure 19.**
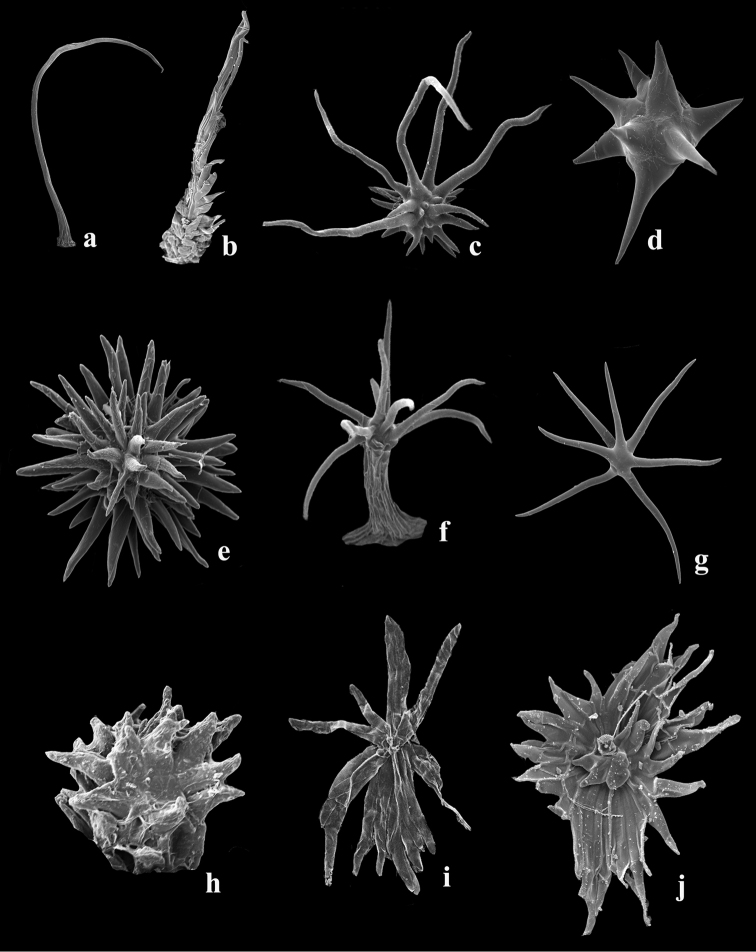
Examples of trichome types in *Conostegia*. **A** *Conostegia dentata* (*J. Cuatrecasas 17668*) **B** *Conostegia bracteata* (*M. Hopkins 22*) **C** *Conostegia ortizae* (*D. Penneys 1857*) **D** *Conostegia superba* (*W. Judd 6521*) **E** *Conostegia extinctoria* (H. David 1227). **F** *Conostegia brenesii* (*R. Kriebel 4907*) **G** *Conostegia subcrustulata* (*L. Williams 27545*) **H** *Conostegia rufescens* (*D. Penneys 1792*) **I** *Conostegia consimilis* (*A. Jiménez 2326*) **J** *Conostegia osaensis* (*R. Aguilar 12890*).

**Figure 20.**
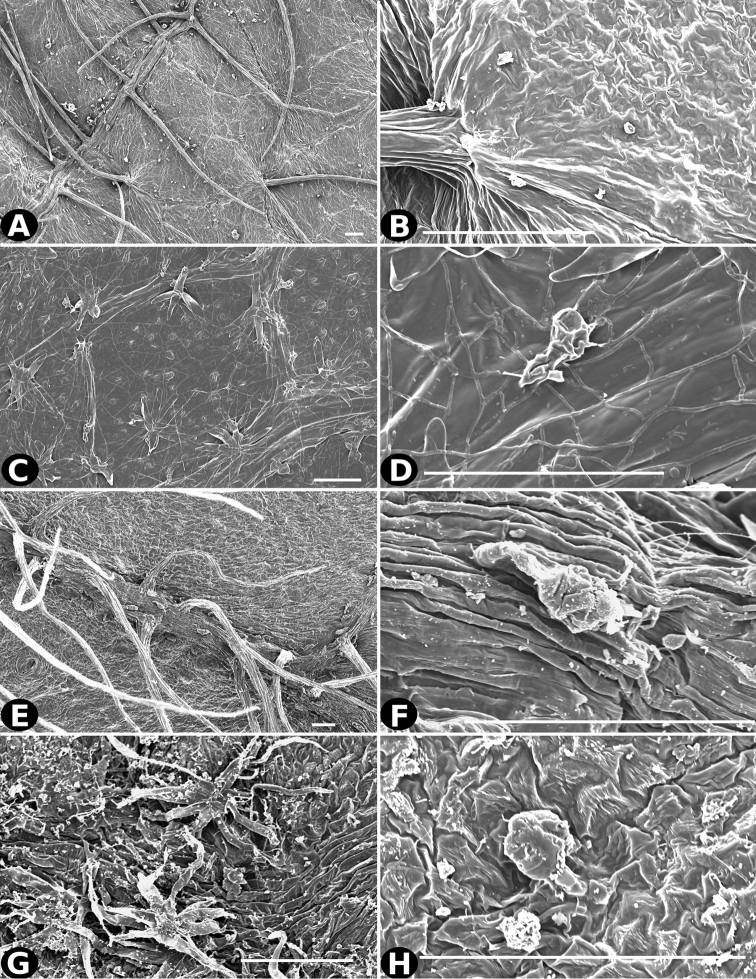
Trichomes in *Conostegia*. **A–B** *Conostegia allenii* (*G. de Nevers 7207*) **C–D** *Conostegia ecuadorensis* (*J. Betancur 3202*) **E–F** *Conostegia foreroi* (*F. Almeda 10336*) **G–H** *Conostegia fraterna* (*R. Kriebel 5774*). Scale bar: 100 µm.

**Figure 21.**
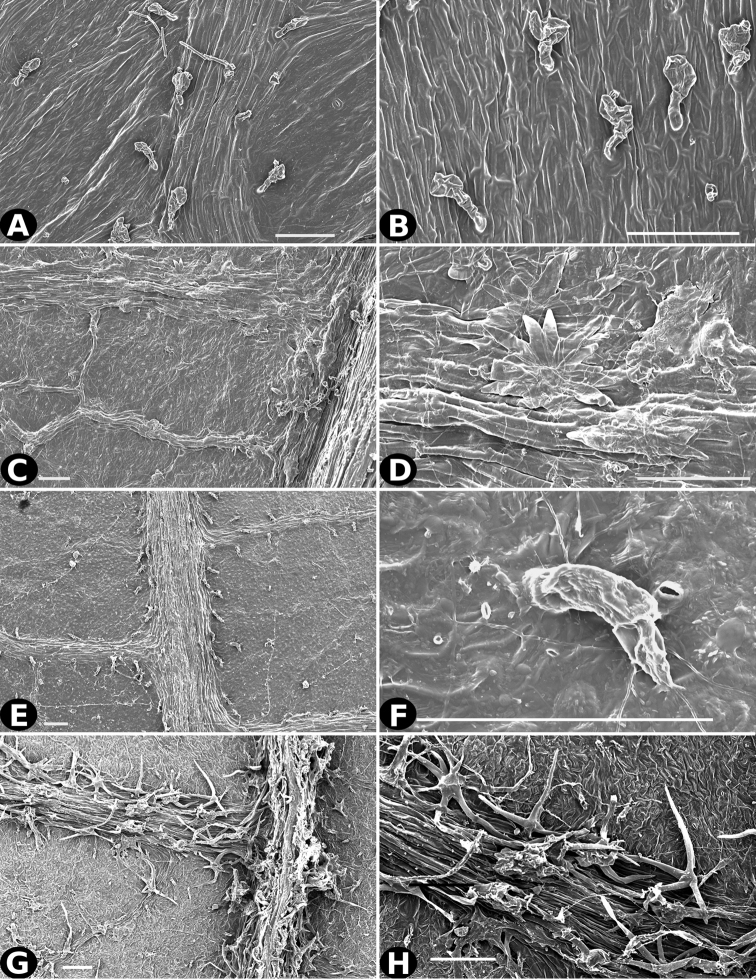
Trichomes in *Conostegia* **A–B** *Clidemia hammelii* (*L. Acosta 133*) **C–D** *Conostegia ombrophila* (*R. Kriebel 5730*) **E–F** *Conostegia henripittieri* (*R. Kriebel 5757*) **G–H** *Conostegia subpeltata* (*R. Kriebel 5347*). Scale bar: 100 µm.

**Figure 22.**
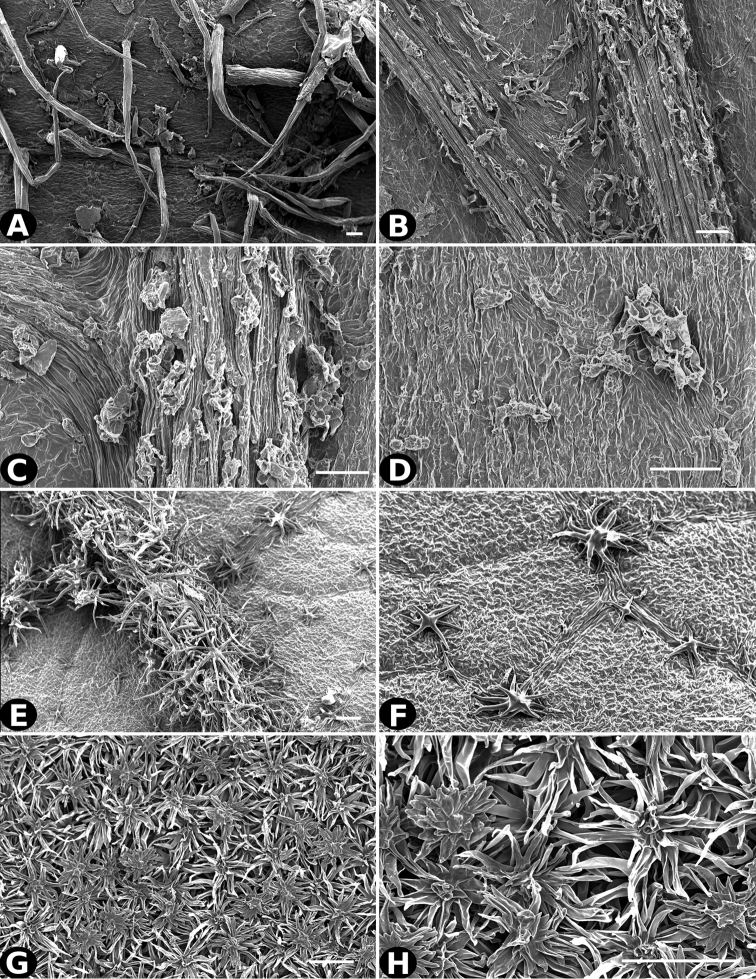
Trichomes in *Conostegia*. **A** *Conostegia trichosantha* (*F. Almeda 6491*). **B** *Conostegia cinnamomea* (*T. Croat 6542*) **C–D** *Conostegia brenesiana* (*J. Taylor 17646*) **E–F** *Conostegia calocoma* (*R. Kriebel 5484*) **G–H** *Conostegia centrosperma* (*R. Kriebel 5690*). Scale bar: 100 µm.

**Figure 23.**
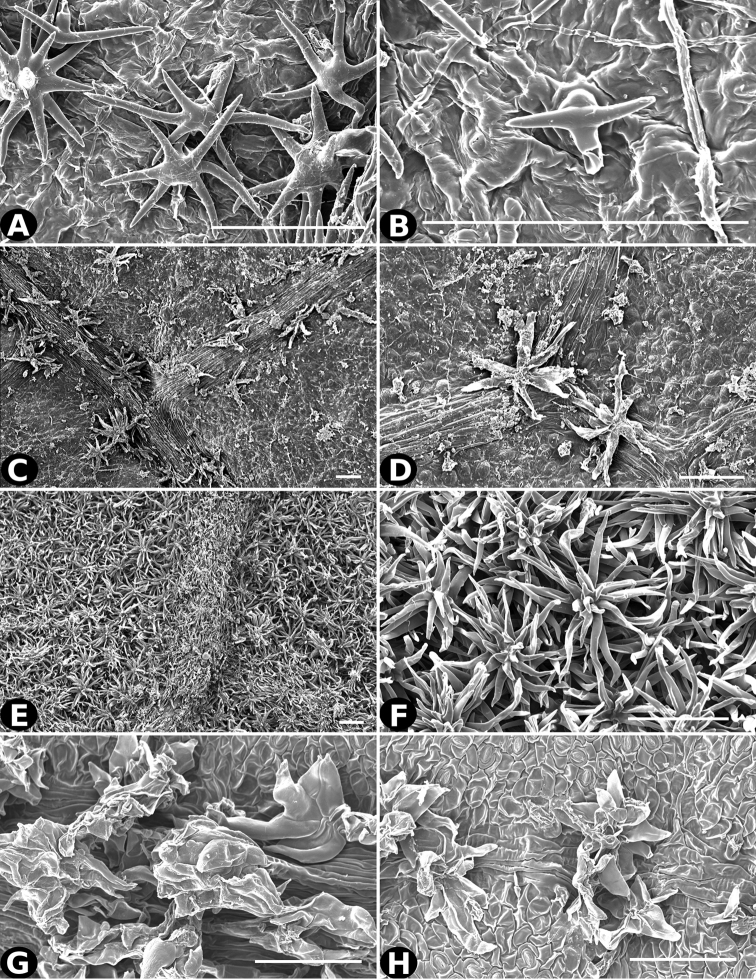
Trichomes in *Conostegia* **A–B** *Conostegia colliculosa* (*R. Kriebel 5721*) **C–D** *Conostegia dissitiflora* (*R. Kriebel 5378*) **E–F** *Conostegia dissitinervia* (*R. Kriebel 5377*) **G–H** *Conostegia friedmaniorum* (*R. Kriebel 5721*). Scale bar: 100 µm.

**Figure 24.**
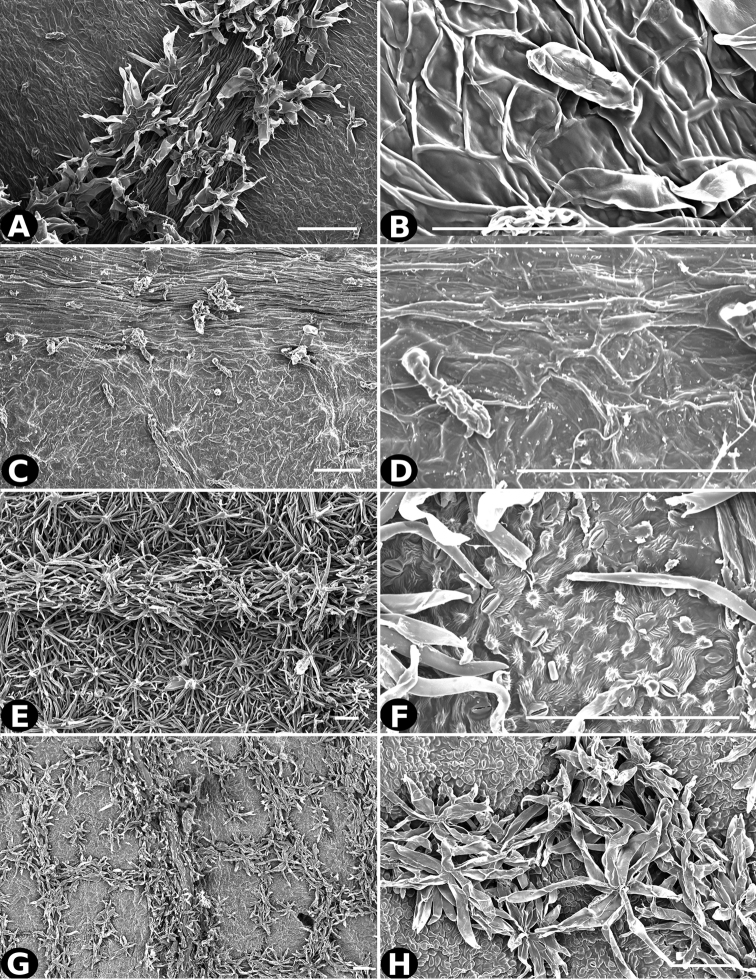
Trichomes in *Conostegia* **A–B** *Conostegia galdamesiae* (*R. Kriebel 5836*) **C–D** *Conostegia grayumii* (*T. McDowell 199*) **E–F** *Conostegia incurva* (*W. Alverson 2747*) **G–H** *Conostegia jefensis* (*R. Kriebel 5680*). Scale bar: 100 µm.

**Figure 25.**
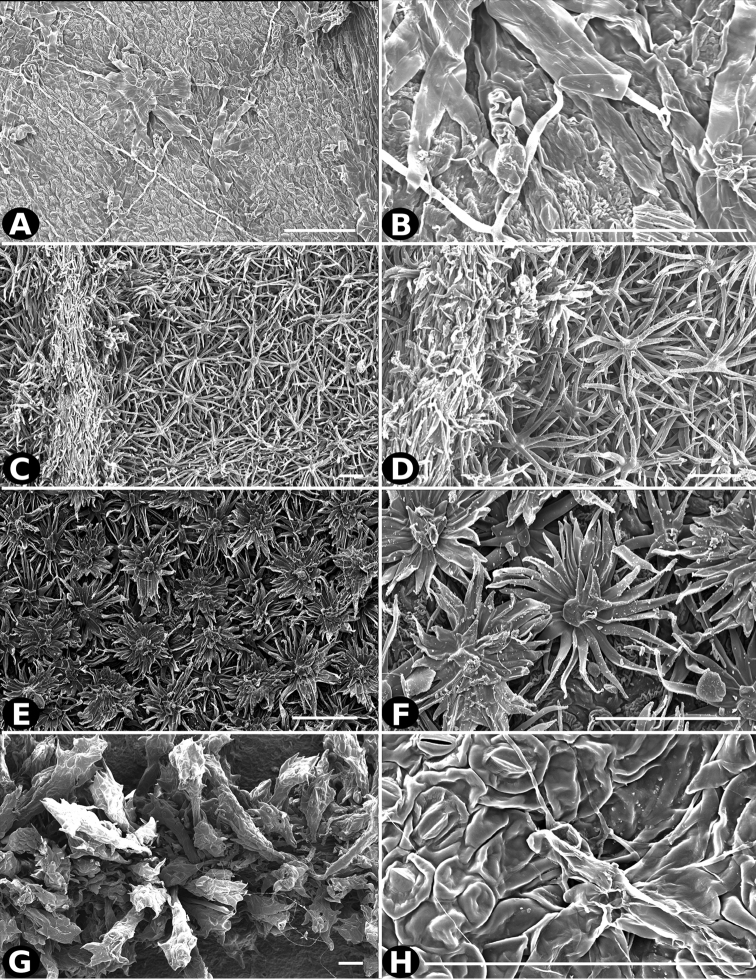
Trichomes in *Conostegia* **A–B** *Conostegia consimilis* (*A. Jimenez 2326*) **C–D** *Conostegia oligocephala* (*R. Kriebel 8575*) **E–F** *Conostegia osaensis* (*R. Aguilar 12890*) **G–H** *Conostegia papillopetala* (*R. Kriebel 5718*). Scale bar: 100 µm.

**Figure 26.**
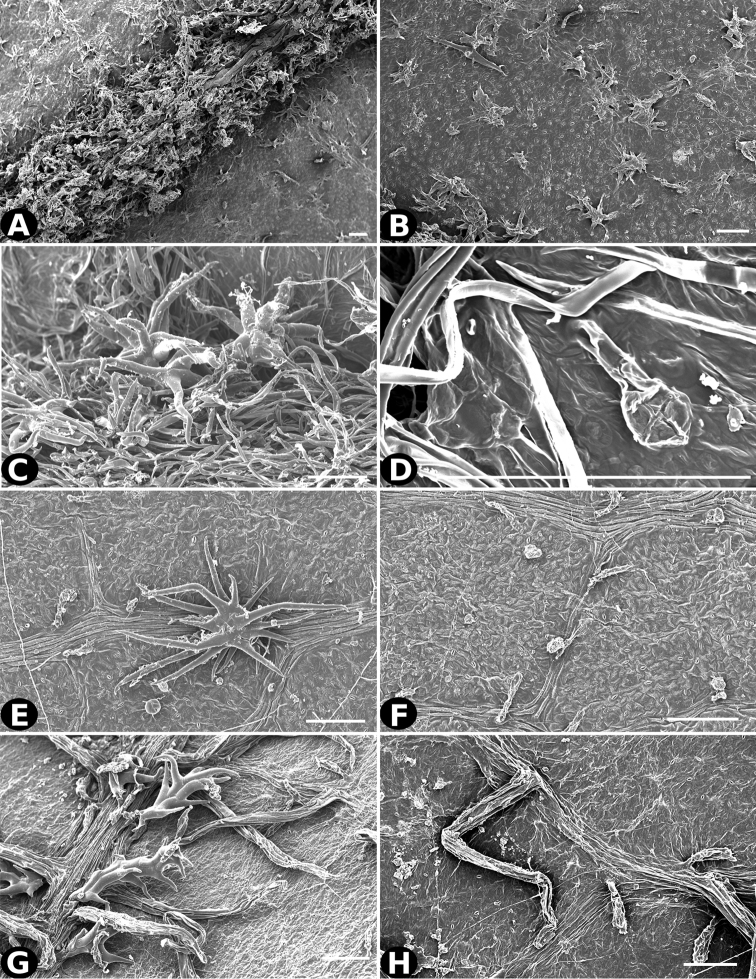
Trichomes in *Conostegia* **A–B** *Conostegia peltata* (*R. Kriebel 5658*) **C–D** *Conostegia povedae* (*F. Oviedo 1908*) **E–F** *Conostegia schlimii* (*T. G. Yuncker 8780*) **G–H** *Conostegia shattuckii* (*R. Kriebel 5688*). Scale bar: 100 µm.

**Figure 27.**
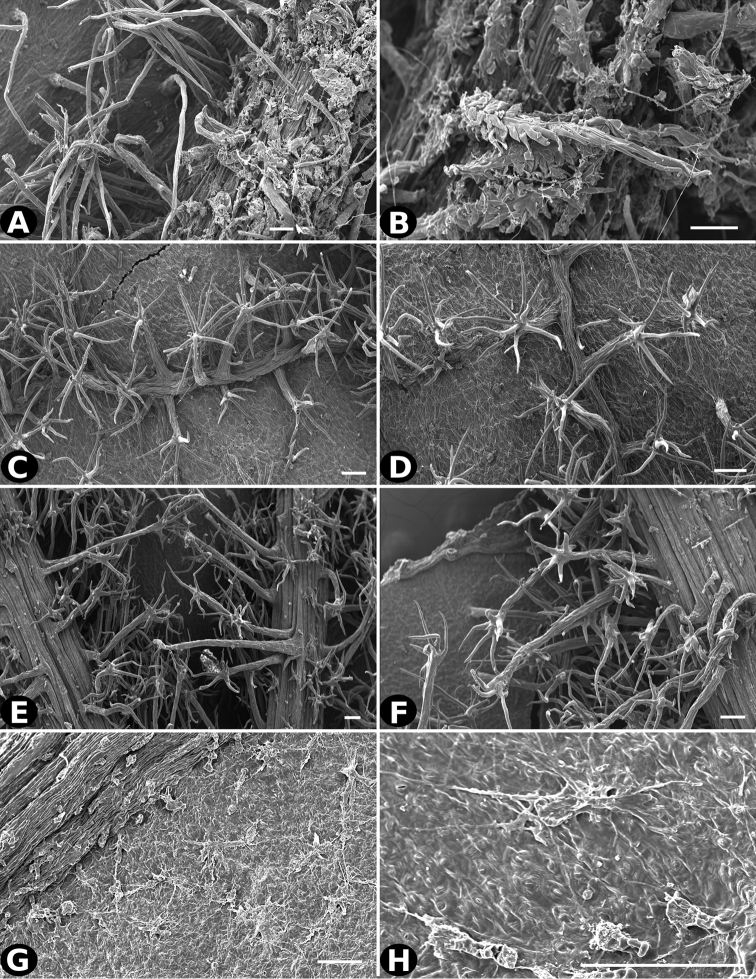
Trichomes in *Conostegia* **A–B** *Conostegia bracteata* (*M. Hopkins 22*) **C–D** *Conostegia brenesii* (*R. Kriebel 4907*) **E–F** *Conostegia caelestis* (*A. Molina 8352*) **G–H** *Conostegia cuatrecasii* (*R. Kriebel 5681*). Scale bar: 100 µm.

**Figure 28.**
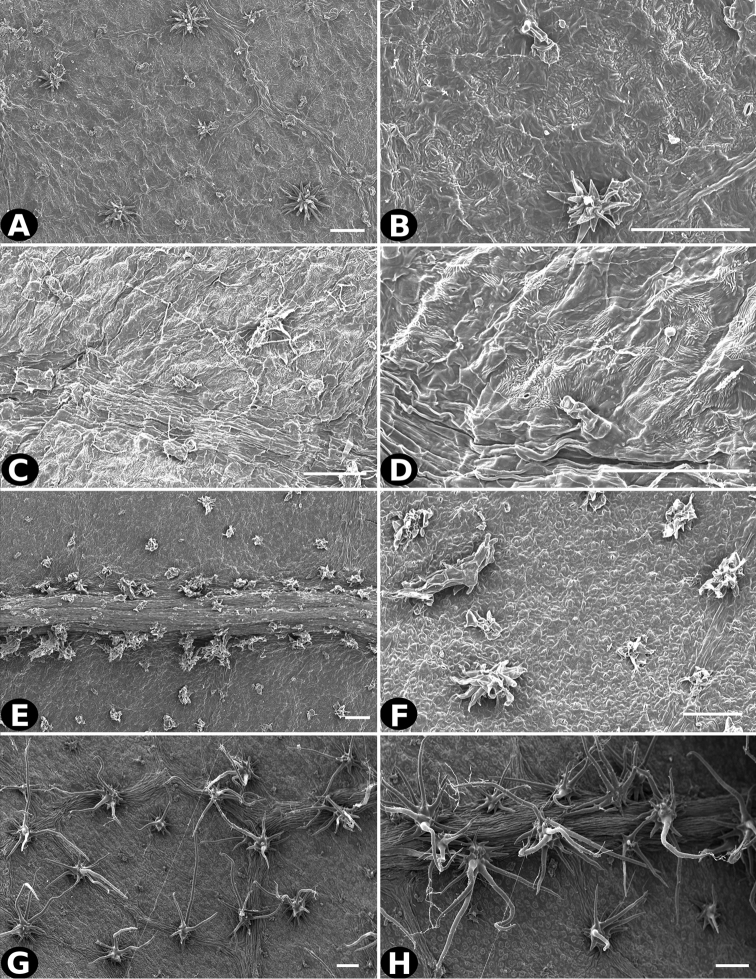
Trichomes in *Conostegia* **A–B** *Conostegia extinctoria* (*H. David 1227*) **C–D** *Conostegia icosandra* (*R. Kriebel 5580*) **E–F** *Conostegia jaliscana* (*F. Almeda 2450*) **G–H** *Conostegia ortizae* (*D. Penneys 1857*). Scale bar: 100 µm.

**Figure 29.**
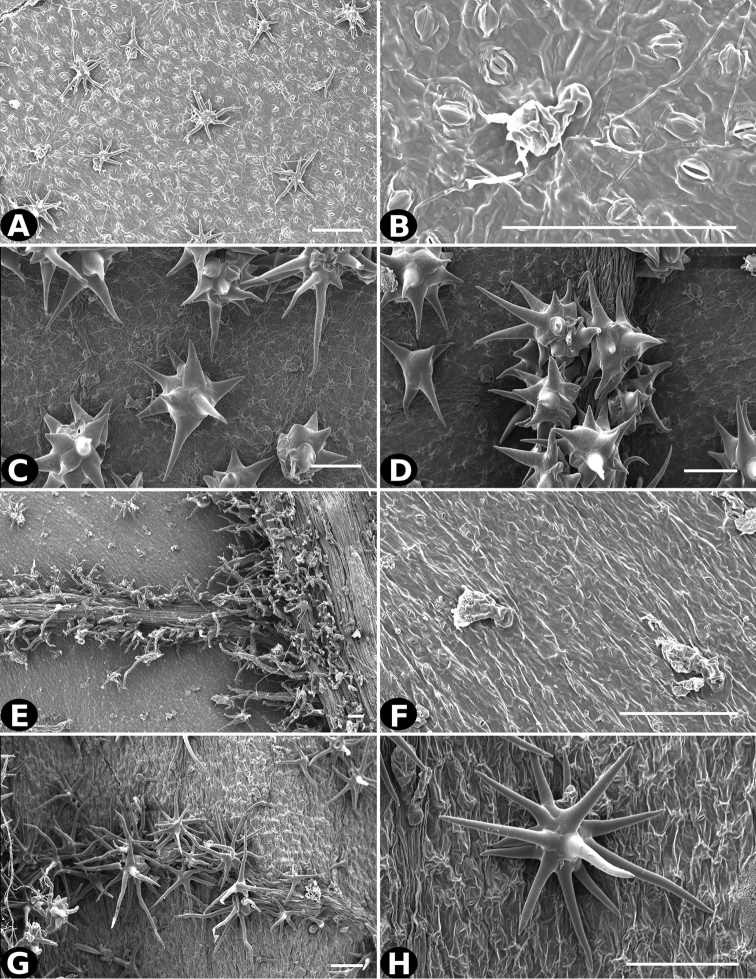
Trichomes in *Conostegia*. **A–B** *Conostegia lasiopoda* (*J. Cuatrecasas 16953*) **C–D** *Conostegia superba* (*W. Judd 6521*) **E–F** *Conostegia macrantha* (*R. Kriebel 5406*) **G–H** *Conostegia micrantha* (*R. Espinoza 1739*). Scale bar: 100 µm.

**Figure 30.**
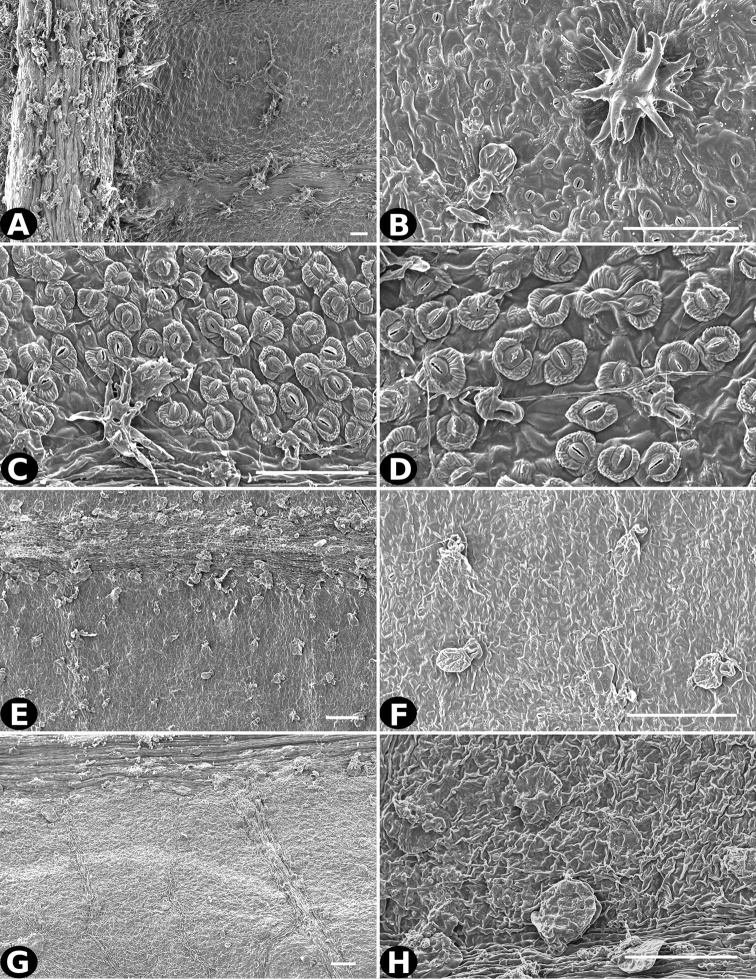
Trichomes in *Conostegia* **A–B** *Conostegia montana* (*R. Kriebel 5662*) **C–D** *Conostegia monteleagreana* (*R. Kriebel 5747*) **E–F** *Conostegia oerstediana* (*R. Kriebel 5408*) **G–H** *Conostegia pittieri* (*R. Kriebel 5400*). Scale bar: 100 µm.

**Figure 31.**
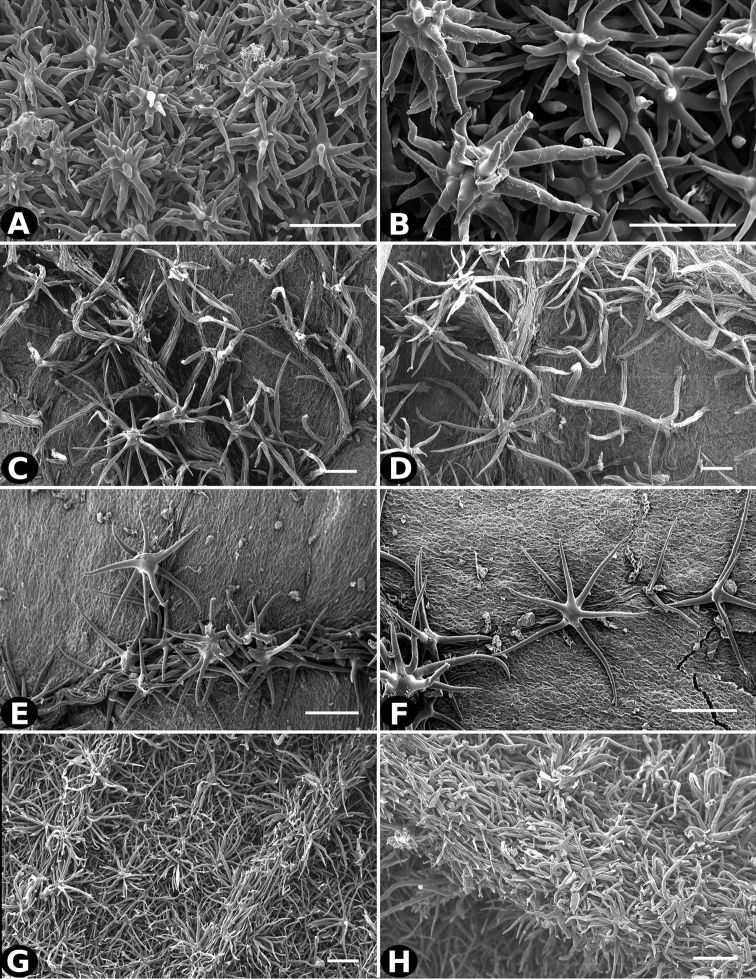
Trichomes in *Conostegia* **A–B** *Conostegia plumosa* (*D.W. Stevens 25247*) **C–D** *Conostegia speciosa* (*S. B. Robbins 6173*) **E–F** *Conostegia subcrustulata* (*L. O. Williams 27545*) **G–H** *Conostegia xalapensis* (*D. Penneys 1758*). Scale bar: 100 µm.

**Figure 32.**
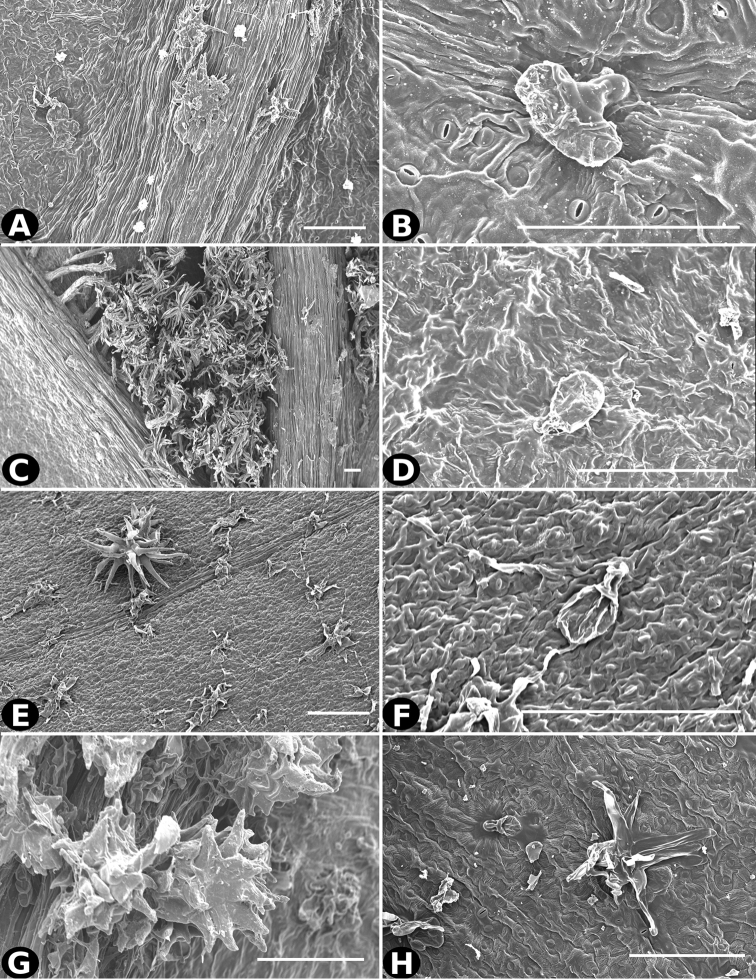
Trichomes in *Conostegia* **A–B** *Conostegia polyandra* (*P. Acevedo 6905*) **C–D** *Conostegia procera* (*W. Maxon 8949*) **E–F** *Conostegia rhodopetala* (*R. Kriebel 5462*) **G** *Conostegia rufescens* (*D. Penneys 1792*) **H** *Conostegia rufescens* (*R. Kriebel 5687*). Scale bar: 100 µm.

**Figure 33.**
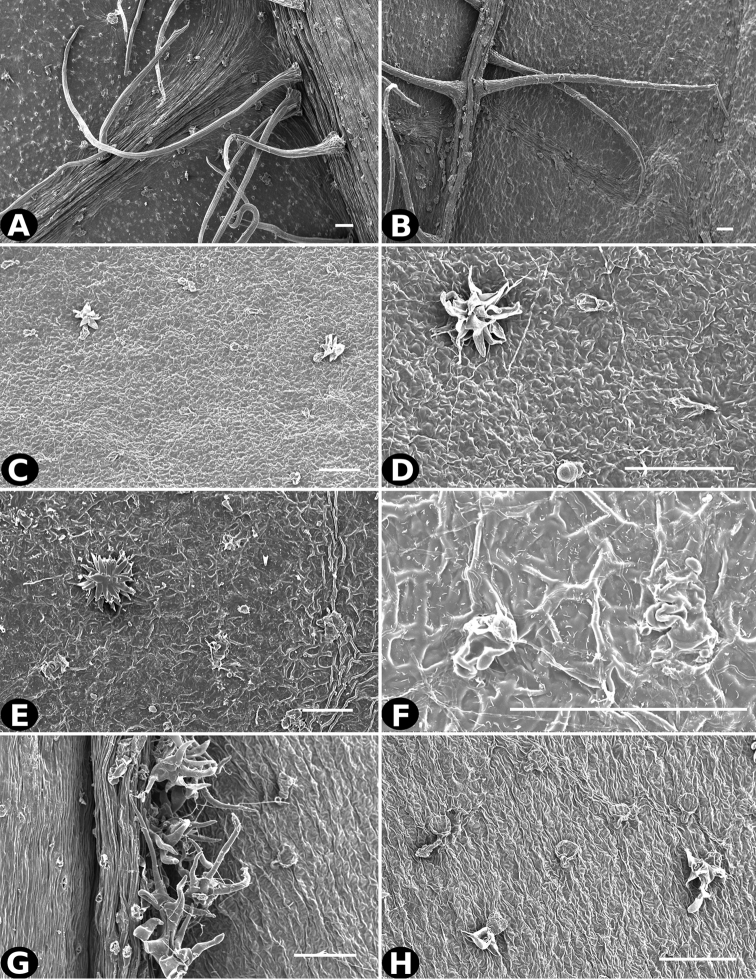
Trichomes in *Conostegia*. **A** *Conostegia cuatrecasii* (*J. Cuatrecasas 17668*). **B** *Conostegia setosa* (*M. Tirado 529*) **C–D** *Conostegia superba* (*R. Kriebel 5582*) **E–F** *Conostegia tenuifolia* (*R. Moran 7950*) **G–H** *Conostegia volcanalis* (*R. Kriebel 5565*). Scale bar: 100 µm.

Trichomes in *Conostegia* are quite diverse and variable (Fig. 19). This may not come as a surprise since trichomes in the Melastomataceae have been said to be the most diverse in the angiosperms (Wurdack 1986). In many cases trichome morphology is easy to describe, such as with simple or lepidote trichomes. Difficulty in describing trichomes arises with the vast variation in dendritic trichomes, and with the gradations from dendritic to stellate. Wurdack (1986) noted that all types in the family are multicellular and, of the 46 trichomes types he recognized, 14 were recorded in species of *Conostegia* sensu Schnell (1996). Schnell (1996) discussed trichomes in *Conostegia* extensively noting that *Conostegia* species tend to have a mixture of trichomes and that variation within a species is extensive. I have noted extreme variation in *Conostegia icosandra*, which can be densely hirsute in the northern part of its range. *Conostegia superba* has also caused confusion because in the mainland specimens tend to be glabrous or almost so, but in the Dominican Republic they can be quite pubescent except for the floral buds. As noted by Schnell (1996), and even now taking the phylogeny into account, it is evident that similar trichomes have evolved independently within *Conostegia*. One example involves the interdependent evolution of stipitate trichomes of *Conostegia brenesii* and *Conostegia caelestis*. Schnell (1996) divided the trichomes of *Conostegia* into the three general groups recognizable based on the work on Wurdack (1986): 1) tiny glands, 2) elongate simple hairs, and 3) a series of eight potentially intergrading kinds of stellate and branching hairs. Schnell (1996) stated that continuous variation among these eight groups made it difficult to describe them. I agree with Schnell and recommend that into the future, a quantitative method should be developed to describe and perhaps discretize these complicated dendritic to stellate trichomes. In the meantime, I have chosen to document the trichomes in a similar way as Wurdack (1986) did, with extensive Scanning Electron Micrographs (Figs 20–33). Apparently all taxa have minute glands on the abaxial leaf surface, and their shape is variable and may prove to be taxonomically informative. Their small size makes them difficult to describe, especially in species with dense pubescence on the leaf abaxial surfaces. To compliment Wurdack’s (1986) initiative for *Conostegia*, I placed special attention on documenting these minute glands as much as possible.

All species with discolorous leaves are in section *Geniculatae*. However, this character has evolved independently a number of times within the section and not all discolorous leaves are the result of the same type of trichome. In some cases such as *Conostegia xalapensis* the dense indument on the abaxial surface is made up of stellate trichomes with long, thin arms, whereas in closely related *Conostegia osaensis* they are lepidote. Many species in section *Geniculatae* have an orange-colored indument of dendritic to stellate trichomes on the stems, especially towards the apex, which are less common in the other sections or if present do not tend not to form a conspicuous orange covering.

### Inflorescence

Inflorescences in *Conostegia* are variable among species as in most clades of Miconieae (Michelangeli et al. 2004). Most species have terminal erect panicles with many flowers, but the inflorescence can be deflexed. In some species previously described in *Miconia* as well as in some species traditionally placed in *Conostegia* such as *Conostegia cinnamomea* and *Conostegia muriculata*, the inflorescence can be evidently deflexed. Axillary or pseudolateral inflorescences tend to have fewer, smaller flowers and are present mainly in those taxa previously described in *Clidemia*. Some small-flowered taxa do have many flowers such as *Conostegia consimilis*. Bracts are usually early deciduous in sections *Australis* and sections *Conostegia* except in species such as *Conostegia monteleagreana* which has persistent bracts subtending the floral glomerules. On the other hand, many species in section *Geniculatae* have bracts and bracteoles that are persistent and fused at the base, forming an inconspicuous nodal collar. Inflorescence branches are particularly thin, wiry, and delicate in several species within section *Geniculatae* such as *Conostegia cinnamomea*, *Conostegia grayumii*, and *Conostegia ombrophila*. It was the presence of this type of inflorescence, as well as the similar bracteoles amongst other characters that prompted Schnell (1996) to state that the similarities between *Conostegia cinnamomea* and *Conostegia brenesiana* are “little short of uncanny, involving almost every character, even to minute details”.

Accessory branches in inflorescences are common in *Conostegia*, especially in taxa with relatively long terminal inflorescences. Although it is tempting to use the presence of these accessory branches in the systematics of the group, they are absent in some specimens, and in such cases it is difficult to tell if they fell off or were not there to begin with. Accessory branches are consistently absent in most species of section *Geniculatae*

Pedicels in *Conostegia* are also variable among species and major clades. In particular, species within section *Conostegia* can have quite long pedicels, as previously noted by Judd and Skean (1991). These pedicels can further elongate as the flowers develop into fruits. Pedicels are absent (flowers sessile) in some species such as *Conostegia monteleagreana* in section *Australis* and *Conostegia colliculosa* and *Conostegia povedae* in section *Geniculatae*. Schnell (1996) noted that in certain species the pedicels are clustered at the end of the inflorescence branches. This clustering is such that the pedicels appear to arise from the axils of other pedicels hence naming these taxa in his section *Axilliflora* Schnell -ined (see figure of *Conostegia superba* for an example). Species in this section do not form a clade as evidenced by the molecular phylogeny where *Conostegia monteleagreana* falls in a different clade than *Conostegia cuatrecasii*, *Conostegia rhodopetala*, and *Conostegia superba* (Fig. 1; see also Kriebel et al. 2015). Nonetheless, Schnell (1996) himself noted that the species of section *Axilliflora* did not sort out into clear groups.

### Flowers

**Figure 34.**
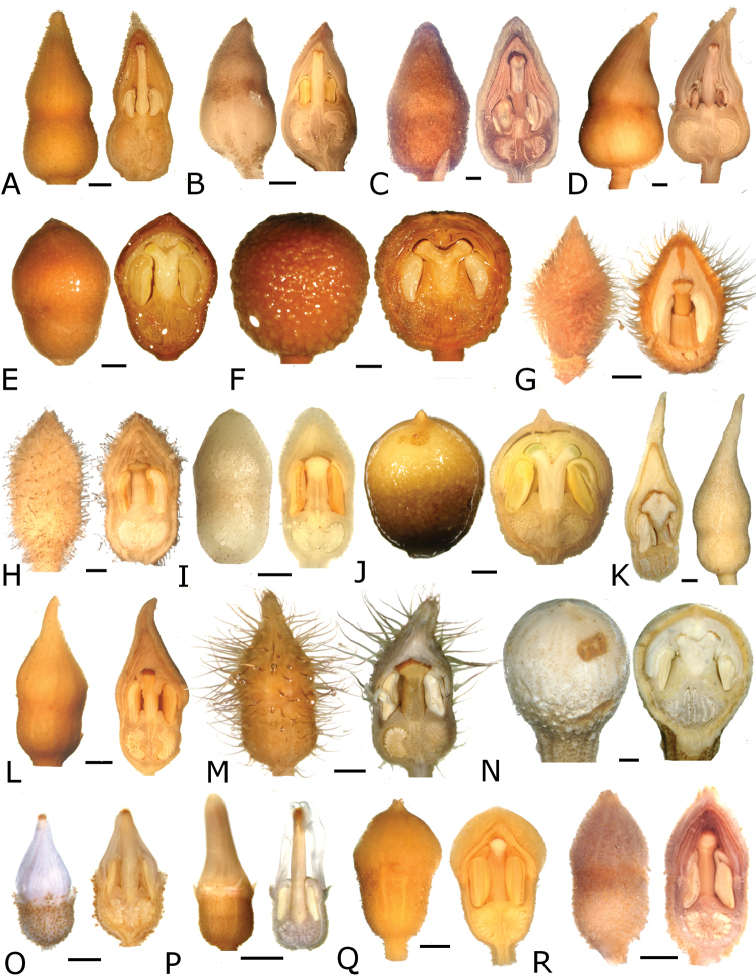
Longitudinal sections of floral buds in *Conostegia*. **A** *Conostegia lasiopoda* (*R. Kriebel 5651*) **B** *Conostegia monteleagreana* (*R. Kriebel 5747*) **C** *Conostegia ortizae* (*D. Penneys 1857*) **D** *Conostegia tenuifolia* (*R. Kriebel 5773*) **E** *Conostegia bernoulliana* (*R. Kriebel 5540*). **F** *Conostegia bigibbosa* (*R. Kriebel 5522*) **G** *Conostegia bracteata* (*R. Kriebel 5806*) **H** *Conostegia brenesii* (*R. Kriebel 5631*) **I** *Conostegia montana* (*R. Kriebel 5593*) **J** *Conostegia oerstediana* (*R. Kriebel 5627*) **K** *Conostegia pittieri* (*R. Kriebel 5400*) **L** *Conostegia rhodopetala* (*R. Kriebel 5542*). **M** *Conostegia setosa* (*R. Kriebel 5731*) **N** *Conostegia volcanalis* (*R. Kriebel 5565*) **O** *Conostegia peltata* (*R. Kriebel 5658*) **P** *Conostegia consimilis* (*R. Kriebel 5726*) **Q** *Conostegia subcrustulata* (*R. Kriebel s.n.*) **R** *Conostegia xalapensis* (*R. Kriebel 5619*). Scale bar: 1 mm.

Floral buds in calyptrate species of *Conostegia* are noticeable because of their calyx tends to fall as a unit at anthesis. Calyptrate calyces have arisen independently at least 15 times just in the Neotropical genera of the family and can be variously shaped (Fig. 34) (Schnell 1996). In the Miconieae, five genera have at least some species with a calyptra namely: *Conostegia*, *Mecranium*, *Miconia*, *Tetrazygia* and *Tococa* (Schnell 1996). Schnell (1996) considered floral buds to be the most useful structures not only to identify species at the generic level but also within *Conostegia*. In general terms their size and shape can be taxonomically useful, as well as the presence of warts or different types of indument on the hypanthium. The apex of the buds can also be helpful for identifying some species. An apiculate calyptra for example, is present in several distinct species from different geographical areas. In South America, the aptly named *Conostegia apiculata* is readily distinguished. In northern Central America, the distinctively apiculate calyptra of *Conostegia arborea* is not easily confused with others; the same is true of *Conostegia pittieri* in the mountains of Costa Rica and western Panama, but in this area there are other species that can have somewhat apiculate calyptras (e.g. *Conostegia tenuifolia* and *Conostegia rhodopetala*).

Phylogenetic and anatomical analyses have revealed that the calyptra evolved at least three times within *Conostegia* (Kriebel et al. 2015). This is a surprise because most workers previously thought the calyptrate calyx was shared by all species in the genus (Judd and Skean 1991; Schnell 1996) even referring to *Conostegia* as monophyletic without phylogenetic analyses of any kind (Judd and Skean 1991). Schnell (1996) did point to the possibility of the independent origin of the calyptra in *Conostegia cinnamomea*, and stated that because these structures had evolved in the family there was possibly a selective force driving their evolution. The adaptive value of the calyptra remains unknown.

**Figure 35.**
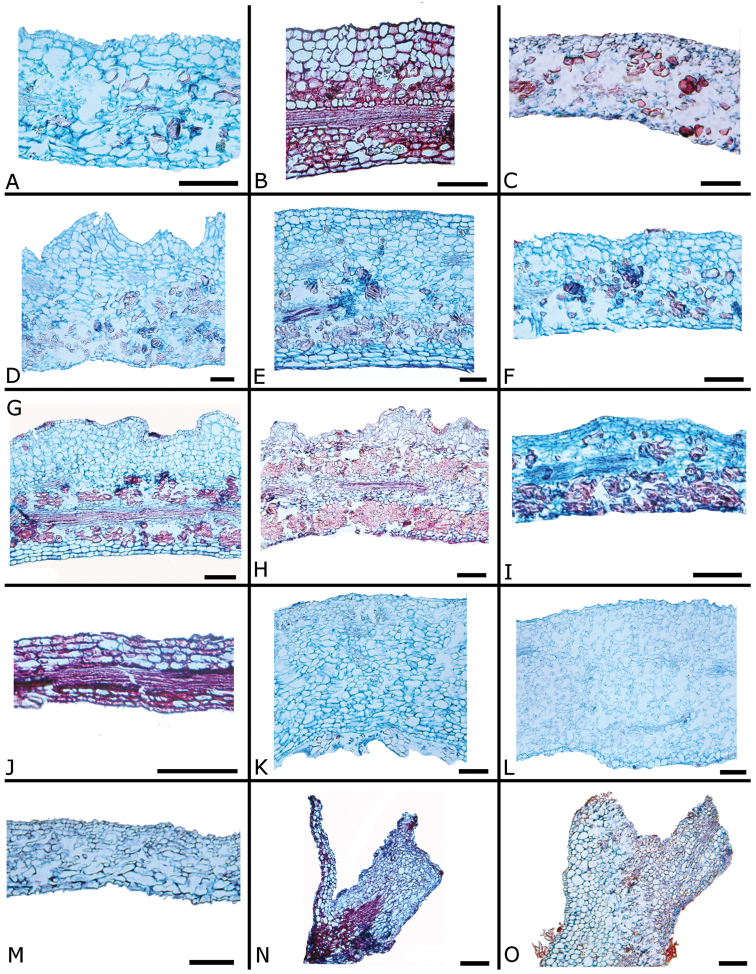
Calyptra and calyx teeth anatomy in *Conostegia*. **A** *Conostegia lasiopoda* (*R. Kriebel* 5651) **B** *Conostegia monteleagreana* (*R. Kriebel 5747*) **C** *Conostegia tenuifolia* (*D. Santamaria 8863*) **D** *Conostegia brenesii* (*R. Kriebel* 5631) **E** *Conostegia icosandra* (*R. Kriebel 5578*) **F** *Conostegia montana* (*R. Kriebel 5544*) **G** *Conostegia oerstediana* (*R. Kriebel 5627*) **H** *Conostegia rufescens* (*R. Kriebel 5635*) **I** *Conostegia superba* (*R. Kriebel 5582*) **J** *Conostegia cinnamomea* (*R. Kriebel 5330*) **K** *Conostegia speciosa* (*R. Kriebel 5677*) **L** *Conostegia subcrustulata* (*R. Kriebel 5653*) **M** *Conostegia xalapensis* (*R. Kriebel 5629*) **N** *Conostegia friedmaniorum* (*R. Kriebel 5641*) **O** *Conostegia schlimii* (*R. Kriebel 5614*). Scale bars: 100 µm.

**Figure 36.**
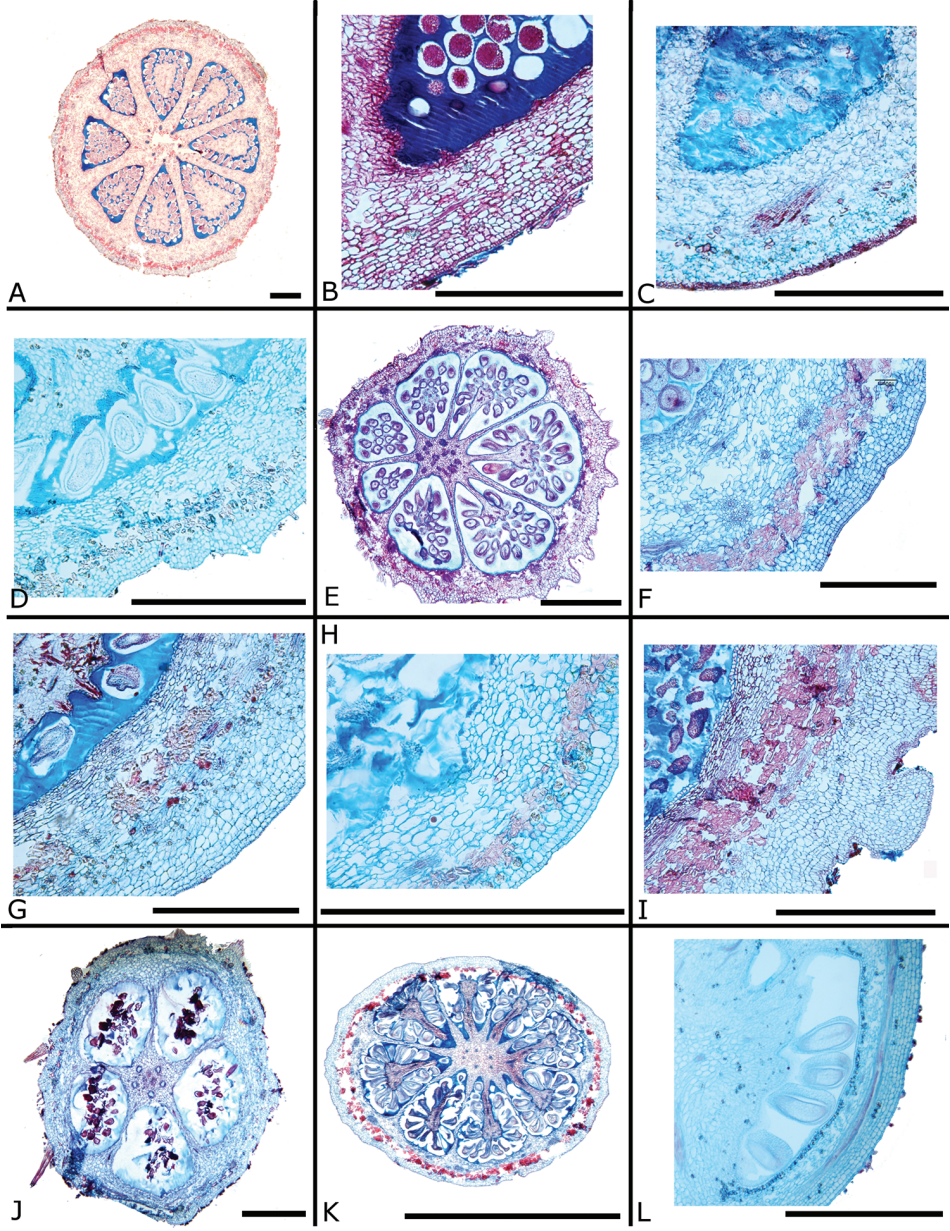
Ovary and hypanthium anatomy in *Conostegia*. **A** *Conostegia lasiopoda* (*R. Kriebel 5651*) **B** *Conostegia monteleagreana* (*R. Kriebel 5747*) **C** *Conostegia tenuifolia* (*D. Santamaria 8863*) **D** *Conostegia brenesii* (*R. Kriebel 5631*) **E** *Conostegia bracteata* (*R. Kriebel 5816*) **F** *Conostegia cuatrecasii* (*R. Kriebel 5681*) **G** *Conostegia icosandra* (*R. Kriebel 5578*) **H** *Conostegia montana* (*R. Kriebel 5544*) **I** *Conostegia oertsediana* (*R. Kriebel 5627*) **J** *Conostegia setosa* (*R. Kriebel 5731*) **K** *Conostegia superba* (*R. Kriebel 5582*) **L** *Conostegia subcrustulata* (*R. Kriebel 5653*). Scale bar: 500 µm.

**Figure 37.**
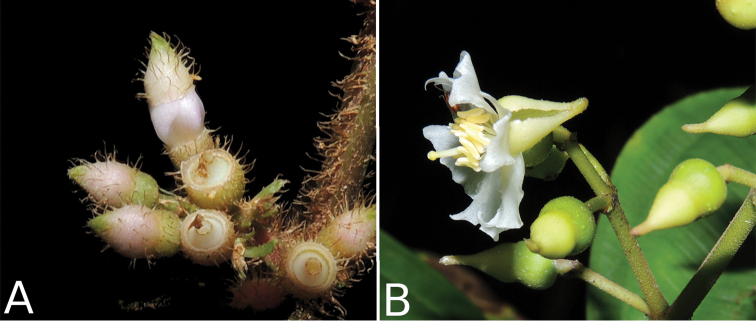
Examples of calyptra dehiscence modes in *Conostegia*. **A** The cleanly circumscissle calyptra of *Conostegia setosa* **B** The calyptra of *Conostegia tenuifolia* ruptured at one side.

The calyptra of species in sections *Australis* and *Conostegia* are very similar in that they have conspicuous sclereids and no calyx teeth nor appendages (Fig. 35). The sclereids for the most part form a layer or two throughout the hypanthium and calyptra (Fig. 36). The main difference in the calyptra of these two sections is that in section *Australis*, the calyptra ruptures at one side and/or breaks into pieces in all species observed in the field (Fig. 37). In section *Conostegia* all evidence points to a cleanly circumscissle calyptra which Judd and Skean (1991) and Schnell (1996) believed was the case in all species of *Conostegia*. Independent origins of the calyptra are also seen in *Conostegia cinnamomea*, which has a very thin calyptra, and in the clade comprised of *Conostegia osaensis*, *Conostegia plumosa*, *Conostegia speciosa*, *Conostegia subcrustulata*, and *Conostegia xalapensis*. The calyptra in this latter clade lacks evident sclereids (Fig. 35). Although most species have a glabrous calyptra on the inside, three closely related species, i.e. *Conostegia plumosa*, *Conostegia speciosa* and *Conostegia xalapensis* tend to have stellate trichomes inside the calyptra.

**Figure 38.**
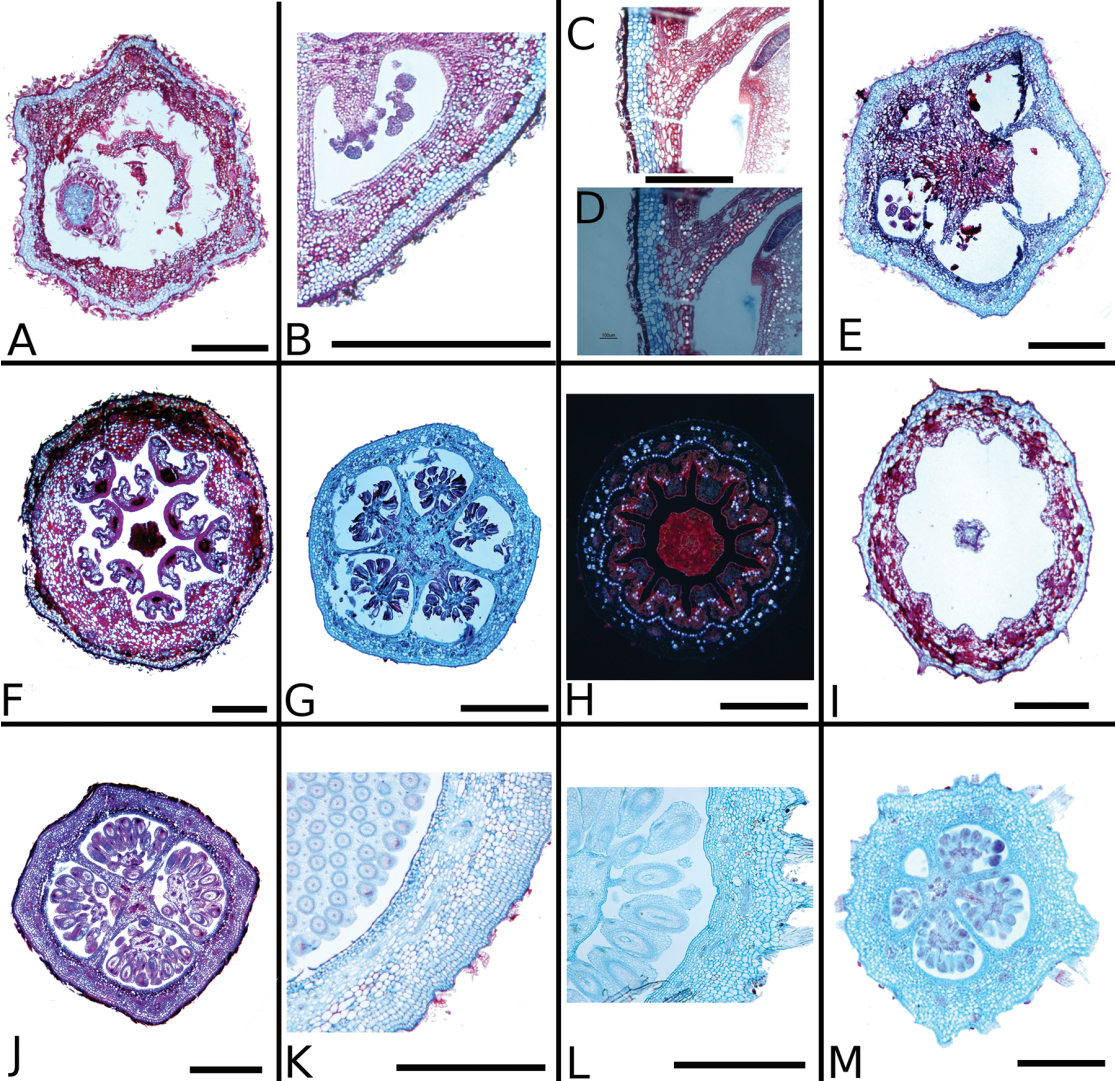
Ovary and hypanthium anatomy in *Conostegia*. **A** *Conostegia centrosperma* (*R. Kriebel 5690*) **B–D** *Conostegia cinnamomea* (*R. Kriebel 5330*) **D** under polarized light **E** *Conostegia dissitinervia* (*R. Kriebel 5377*) **F** *Conostegia fraterna* (*R. Kriebel 5774*) **G–H** *Conostegia grayumii* (*R. Kriebel 5807*) **H** under polarized light **I** *Clidemia hammelii* (*R. Kriebel 5317*) **J** *Conostegia ombrophila* (*R. Kriebel 3120*) **K** *Conostegia schlimii* (*R. Kriebel 5614*) **L** *Conostegia speciosa* (*R. Kriebel 5677*) **M** *Conostegia trichosantha* (*R. Kriebel 5693*). Scale bar: 500 µm except **C** and **D**: 100 µm.

Within section *Geniculatae*, sclereids are mostly absent from the hypanthium and calyx. Some scattered sclereids are seen in the hypanthium of *Conostegia schlimii*. In the floral anatomy of this section there are two general patterns that are absent in sections *Australis* and *Conostegia*. First, in several species of *Geniculatae* there is a lining of druses around the ovary (Fig. 38). Second, several species have a distinctly bicolored anatomy where the ovary tissue stains red, indicating some lignification but the rest of the hypanthium stains blue. In fewer specimens of section *Geniculatae* the hypanthium stains either totally red or entirely blue. For further anatomical differences between the ovaries in section *Geniculatae* and the other two sections *Australis* and *Conostegia*, see below under the Gynoecium section.

**Figure 39.**
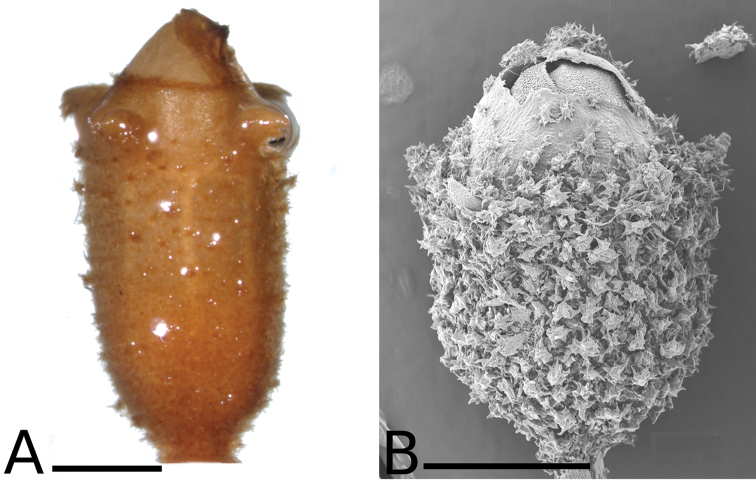
Examples of irregularly rupturing calyces in *Conostegia*. **A** Flower bud from spirit collection of *Conostegia subpeltata* (*R. Kriebel 3643*) **B** Scanning electron micrograph of flower bud of *Conostegia papillopetala* (*R. Kriebel 5718*). Scale bar: 1 mm.

Another unusual characteristic of the calyx of non-calyptrate species in section *Geniculatae* is the presence of an irregularly rupturing calyx in several of them (Fig. 39). This type of calyx is usually translucent. An example of the anatomy of the translucent calyx of *Conostegia friedmaniorum* is presented next to the calyx of *Conostegia schlimii* (a species without a translucent calyx) (Fig. 35). In all cases of species with this type of calyx, because it ruptures, the calyx lobes are irregularly shaped. The calyx teeth in calyptrate taxa are difficult to assess. In sections *Australis* and most calyptrate species of *Geniculatae* they appear to be totally absent and anatomical sections failed to reveal them. In *Conostegia cinnamomea*, anatomical sections did evidence their presence. On the other hand, within the clade of *Conostegia osaensis*, *Conostegia plumosa*, *Conostegia speciosa*, *Conostegia subcrustulata*, and *Conostegia xalapensis* one sees what seem to be inconspicuous calyx teeth in *Conostegia subcrustulata*. Schnell (1996) included the aforementioned clade (except *Conostegia osaensis* which was undescribed at the time) in his section *Tomentostegia*. In his description of that section, Schnell (1996) described the calyx lobes as fused and usually with free teeth or prolonged appendages. Assessing if these teeth or appendages are homologous to calyx teeth is difficult. To complicate things even more, in *Conostegia osaensis*, which is the sister clade, the calyx teeth appear to be at the level of the torus as is common in species of section *Geniculatae* and more generally in most species of the tribe Miconieae.

**Figure 40.**
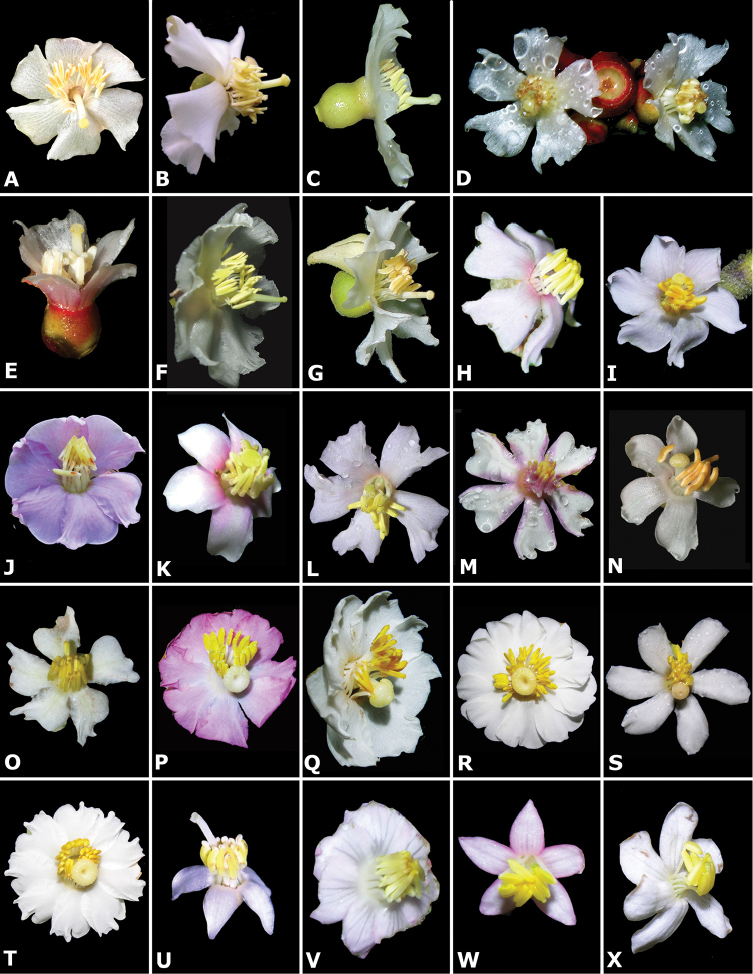
Flowers in Conostegia section Australis (**A–G**), section *Conostegia* (**H–T**), and section *Geniculatae* (**U–X**). **A** *Conostegia centronioides* (*X. Cornejo 8160*) **B** *Conostegia ortizae* (*D. Penneys 1857*) **C** *Conostegia lasiopoda* (*R. Kriebel 5651*) **D** *Conostegia monteleagreana* (*R. Kriebel 5354*). **E** *Conostegia monteleagreana* (*P. Pedraza 1923*) **F** *Conostegia polyandra* (*F. Almeda 10481*) **G** *Conostegia tenuifolia* (*R. Kriebel 5773*). **H** *Conostegia bracteata* (*R. Kriebel 5816*). **I** *Conostegia brenesii* (*R. Kriebel 5631*) **J** *Conostegia cuatrecasii* (*R. Kriebel 5673*) **K** *Conostegia montana* (*R. Kriebel 5446*) **L** *Conostegia rhodopetala* (*R. Kriebel 5542*) **M** *Conostegia rufescens* (*R. Kriebel 5314*) **N** *Conostegia setosa* (*R. Kriebel 5813*) **O** *Conostegia superba* (*R. Kriebel 5582*) **P** *Conostegia bigibbosa* (*R. Kriebel 5522*) **Q** *Conostegia icosandra* (*R. Kriebel 5580*) **R** *Conostegia macrantha* (*R. Kriebel 5406*) **S** *Conostegia oerstediana* (*R. Kriebel 5408*) **T** *Conostegia pittieri* (*R. Kriebel 5543*) **U** *Conostegia cinnamomea* (*R. Kriebel 5330*) **V** *Conostegia speciosa* (*R. Kriebel 5489*) **W** *Conostegia subcrustulata* (*R. Kriebel 5333*). *Conostegia xalapensis* (*R. Kriebel 5555*). Flowers not to scale.

**Figure 41.**
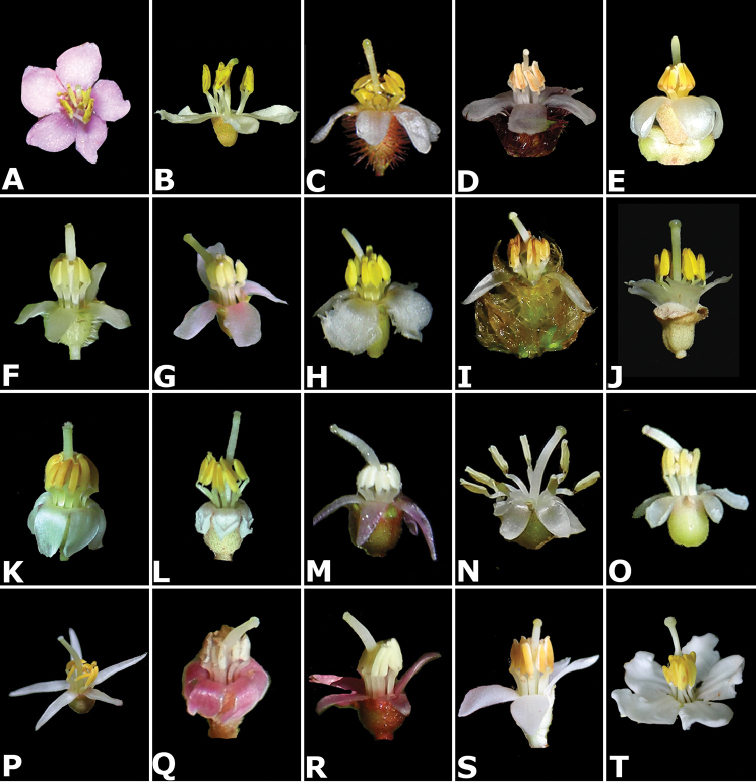
Flowers in Conostegia section Geniculatae. **A** *Conostegia xalapensis* (*R. Kriebel 5619*) **B** *Conostegia osaensis* (*R. Aguilar 10200*) **C** *Conostegia allenii* (*R. Aguilar 13243*) **D** *Conostegia foreroi* (*F. Almeda 10336*) **E** *Conostegia fraterna* (*R. Kriebel 5774*) **F** *Clidemia hammelii* (*R. Kriebel 5737*) **G** *Conostegia ombrophila* (*R. Kriebel 3120*) **H** *Conostegia subpeltata* (*R. Kriebel 5347*) **I** *Conostegia trichosantha* (*R. Kriebel 5693*) **J** *Conostegia centrosperma* (*R. Kriebel 5690*) **K** *Conostegia dissitiflora* (*R. Kriebel 5070*) **L** *Conostegia dissitinervia* (*R. Kriebel 5046*) **M** *Conostegia friedmaniorum* (*R. Kriebel 5641*) **N** *Conostegia galdamesiae* (*R. Kriebel 5736*) **O** *Conostegia grayumii* (*R. Kriebel 5807*) **P** *Conostegia consimilis* (*R. Kriebel 5466*) **Q** *Conostegia papillopetala* (*R. Kriebel 5718*) **R** *Conostegia peltata* (*R. Kriebel 5658*). **S** *Conostegia povedae* (*F. Oviedo 1215*). **T** *Conostegia schlimii* (*R. Kriebel 5095*). Flowers not to scale.

Flowers in the species traditionally treated in *Conostegia* also stand out among genera of Miconieae because most of them are pleiostemonous, meaning that they have more than double the number of stamens than petals (Fig. 40). Other pleiostemonous taxa do exist within the Miconieae, but they are not thought to be closely related to *Conostegia*. There is one pleiostemonous species that has been named in *Conostegia*, *Conostegia inusitata*, which appears not to be closely related to the other named taxa in the genus. This suggestion comes from a nuclear ribosomal DNA sequence (Kriebel, unpublished data) of an undescribed and closely related species to *Conostegia inusitata*, which Schnell (1996) proposed as the new species *Florbella wurdackii*. The genus *Florbella* was proposed by Schnell to accommodate these two species, but it has yet to be published. Inclusion of this DNA sequence in phylogenetic analyses of Miconieae suggests it is more closely related to a clade of mostly Peruvian species of *Miconia* than to species of *Conostegia*. The concept of *Conostegia* included in the present treatment includes many diplostemonous species, particularly those previously described as *Clidemia* and *Miconia*. These diplostemonous taxa are almost completely restricted to section *Geniculatae* (Fig. 41), with a few diplostemonous taxa also found in section *Conostegia* (e.g. *Conostegia setosa*).

### Corolla

**Figure 42.**
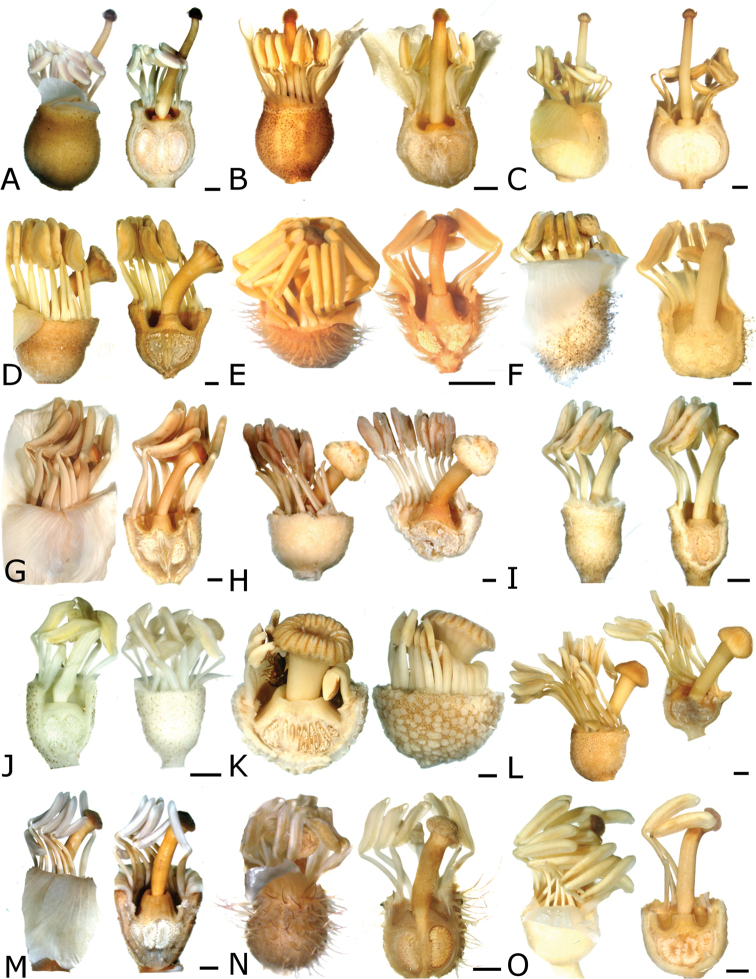
Flowers of *Conostegia* from specimens collected in spirit with a longitudinal section at their side. **A** *Conostegia lasiopoda* (*R. Kriebel 5651*) **B** *Conostegia monteleagreana* (*R. Kriebel 5747*) **C** *Conostegia tenuifolia* (*R. Kriebel 5773*) **D** *Conostegia bernoulliana* (*R. Kriebel 5540*) **E** *Conostegia bracteata* (*R. Kriebel 5816*) **F** *Conostegia brenesii* (*R. Kriebel 5631*). **G** *Conostegia cuatrecasii* (*R. Kriebel 5673*) **H** *Conostegia icosandra* (*R. Kriebel 5580*) **I** *Conostegia montana* (*R. Kriebel 5751*) **J** *Conostegia montana* (*R. Kriebel 5593*) **K** *Conostegia oerstediana* (*R. Kriebel s.n.*) **L** *Conostegia pittieri* (*R. Kriebel 5400*) **M** *Conostegia rufescens* (*R. Kriebel 5314*) **N** *Conostegia setosa* (*R. Kriebel 5731*) **O** *Conostegia superba* (*R. Kriebel 5582*). Scale bar: 1 mm.

**Figure 43.**
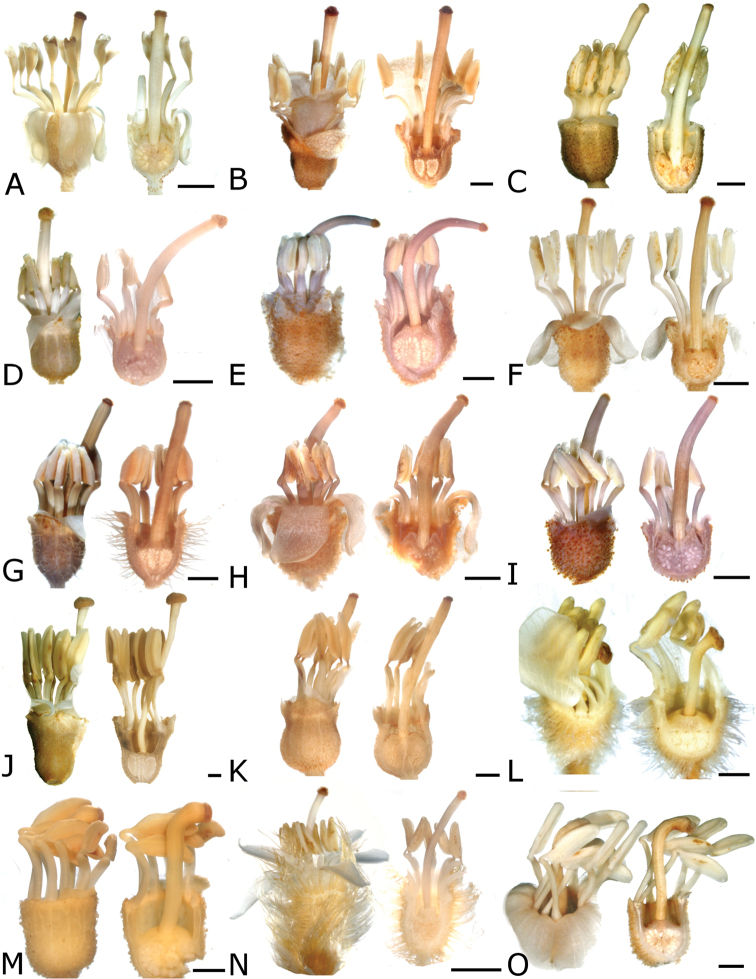
Flowers of *Conostegia* from specimens collected in spirit with a longitudinal section at their side. **A** *Conostegia brenesiana* (*R. Kriebel 3665*) **B** *Conostegia centrosperma* (*R. Kriebel 5690*) **C** *Conostegia cinnamomea* (*R. Kriebel 5330*) **D** *Conostegia consimilis* (*R. Kriebel 5726*) **E** *Conostegia friedmaniorum* (*R. Kriebel 5641*) **F** *Conostegia galdamesiae* (*R. Kriebel 5736*) **G** *Clidemia hammelii* (*R. Kriebel 5737*) **H** *Conostegia papillopetala* (*R. Kriebel 5718*) **I** *Conostegia peltata* (*R. Kriebel 5658*) **J** *Conostegia schlimii* (*R. Kriebel 5614*) **K** *Conostegia shattuckii* (*R. Kriebel 5681*) **L** *Conostegia speciosa* (*R. Kriebel 5677*) **M** *Conostegia subcrustulata* (*R. Kriebel s. n.*) **N** *Conostegia trichosantha* (*R. Kriebel 5693*) **O** *Conostegia xalapensis* (*R. Kriebel 5619*). Scale bar: 1 mm.

**Figure 44.**
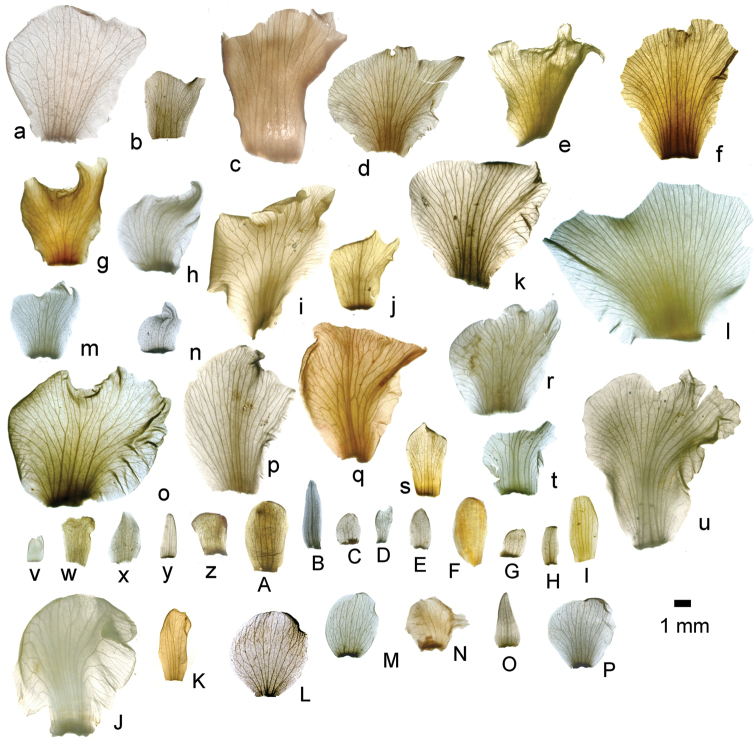
Petals of *Conostegia* to scale. **a** *Conostegia lasiopoda* (*R. Kriebel 5651*) **b** *Conostegia monteleagreana* (*R. Kriebel 5747*) **c** *Conostegia ortizae* (*D. Penneys 1857*) **d** *Conostegia tenuifolia* (*R. Kriebel 5773*) **e** *Conostegia bernoulliana* (*R. Kriebel 5540*) **f** *Conostegia bigibbosa* (*R. Kriebel 5522*) **g** *Conostegia bracteata* (*R. Kriebel 5816*) **h** *Conostegia brenesii* (*R. Kriebel 5631*) **i** *Conostegia cuatrecasii* (*R. Kriebel 5673*) **j** *Conostegia fragrantissima* (*R. Kriebel 3174*) **k** *Conostegia icosandra* (*R. Kriebel 5580*) **l** *Conostegia macrantha* (*R. Kriebel 5406*) **m** *Conostegia montana* (*R. Kriebel 5751*) **n** *Conostegia montana* (*R. Kriebel 5593*) **o** *Conostegia oerstediana* (*R. Kriebel 5627*) **p** *Conostegia pittieri* (*R. Kriebel 5400*) **q** *Conostegia rhodopetala* (*R. Kriebel 5542*) **r** *Conostegia rufescens* (*R. Kriebel 5627*) **s** *Conostegia setosa* (*R. Kriebel 5731*) **t** *Conostegia superba* (*R. Kriebel 5582*) **u** *Conostegia volcanalis* (*R. Kriebel* 5565) **v** *Conostegia brenesiana* (*R. Kriebel 3665*) **w** *Conostegia centrosperma* (*R. Kriebel 5690*) **x** *Conostegia cinnamomea* (*R. Kriebel 5330*) **y** *Conostegia consimilis* (*R. Kriebel 5726*) **z** *Conostegia dissitinervia* (*R. Kriebel 5317*) **A** *Conostegia fraterna* (*R. Kriebel 5774*) **B** *Conostegia friedmaniorum* (*R. Kriebel 5641*) **C** *Conostegia galdamesiae* (*R. Kriebel 5736*) **D** *Conostegia grayumii* (*R. Kriebel 5807*) **E** *Clidemia hammelii* (*R. Kriebel 5737*) **F** *Conostegia ombrophila* (*R. Kriebel 3120*). **G** *Conostegia papillopetala* (*R. Kriebel 5718*) **H** *Conostegia peltata* (*R. Kriebel 5658*) **I** *Conostegia povedae* (*F. Oviedo 231*) **J** *Conostegia schlimii* (*R. Kriebel 5614*) **K** *Conostegia shattuckii* (*R. Kriebel 5681*) **L** *Conostegia speciosa* (*R. Kriebel 5677*) **M** *Conostegia subcrustulata* (*R. Kriebel s.n.*) **N** *Conostegia subpeltata* (*R. Kriebel 3643*) **O** *Conostegia trichosantha* (*R. Kriebel 5693*) **P** *Conostegia xalapensis* (*R. Kriebel 5619*).

The corolla in *Conostegia* varies dramatically in size, shape and number of parts among species (Figs 42, 43) and sometimes also within species. Petal number can range from 4 to 12. Petal shape is quite variable among species and in general they tend to be asymmetric with notable exceptions e.g. in some species in section *Geniculatae*. Many species especially in section *Australis* and section *Conostegia* tend to have strongly asymmetrical petals. The posture of the petals is usually spreading with some species (particularly small-flowered ones) having reflexed petals. Petals are always imbricate in bud and overlap in a counter-clockwise fashion (Schnell 1996). At anthesis petals are usually imbricate in large flowered taxa but the overlap tends to decrease as the flowers get smaller. Petal margins are for the most part entire but in many large-flowered taxa they can have a more membranous texture on one side (Fig. 44). Petal apices are mostly rounded, truncate, or emarginate, and in a few species they can be narrowly rounded to acute. One species (*Conostegia consimilis*) was originally described in the genus *Leandra* because of its acuminate petals. Petal bases are not evidently clawed, except in *Conostegia schlimii*.

**Figure 45.**
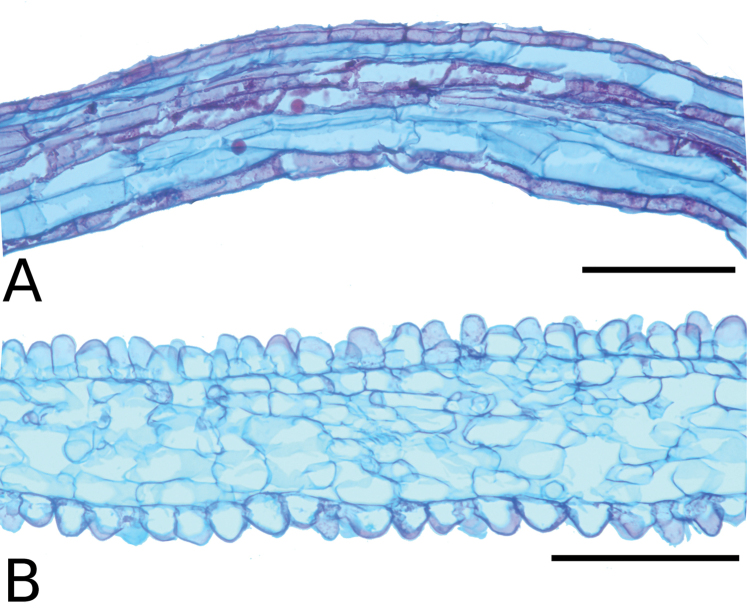
Longitudinal sections of petals in *Conostegia*. **A** Representative section of a species with translucent petals, *Conostegia friedmaniorum* **B** Representative section of a species with white petals, *Conostegia pittieri*. Scale bar: 100 µm.

**Figure 46.**
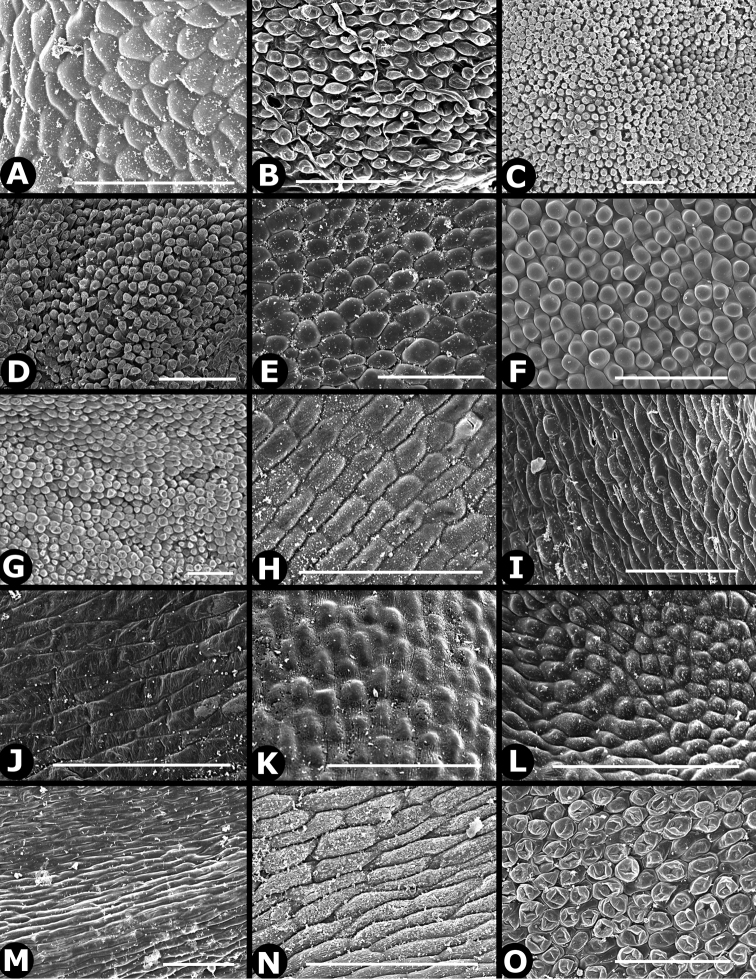
Scanning electron micrographs of *Conostegia* petal surfaces. **A** *Conostegia monteleagreana* (*R. Kriebel 5343*) **B** *Conostegia brenesii* (*R. Kriebel 5631*) **C** *Conostegia bernoulliana* (*R. Kriebel 5540*) **D** *Conostegia montana* (*R. Kriebel 5496*) **E** *Conostegia oerstediana* (*R. Kriebel 5338*) **F** *Conostegia pittieri* (*R. Kriebel 5400*) **G** *Conostegia rhodopetala* (*R. Kriebel 5542*) **H** *Conostegia rufescens* (*R. Kriebel 5314*) **I** *Clidemia hammelii* (*R. Kriebel 5317*) **J** *Conostegia subpeltata* (*R. Kriebel 5347*) **K** *Conostegia cinnamomea* (*R. Kriebel 5330*) **L** *Conostegia dissitinervia* (*R. Kriebel 5377*) **M** *Conostegia friedmaniorum* (*R. Kriebel 5641*) **N** *Conostegia consimilis* (*R. Kriebel 5323*) **O** *Conostegia schlimii* (*R. Kriebel 515*). Scale bar: 100 µm.

White is the most common petal color found in *Conostegia* with a few species having pink to purple petals such as *Conostegia bigibbosa*, *Conostegia cuatrecasii*, and *Conostegia muriculata*. In a few taxa like *Conostegia fragrantissima* the white petals can have a red band at the base. Translucent petals can be found in different clades as well, and although it is tempting to think this might be related to floral size because several small-flowered taxa have them, some species with large petals have translucent petals as well (e.g. *Conostegia lasiopoda*). Most species have glabrous petals but at least one has conspicuously papillose petals (*Conostegia papillopetala*). Petal surfaces tend to be smooth in translucent petaled taxa and with rounded papillose cells in white-flowered species (Fig. 45). Although this difference in petal cells is evident also in micrographs (Fig. 46), intermediates exist and further study is needed to determine their possible systematic utility. In a few species, the petals persist after all other floral parts have fallen (of which *Conostegia pittieri* might be the most notable example).

### Androecium

The androecium in *Conostegia* consists of 8–52 isomorphic stamens. The basic arrangement of the stamens consists of five of them inserted opposite the sepals and five of them opposite the petals like most species in the Melastomataceae. Increase in the number of stamens are common, even predominant in sections *Australis* and *Conostegia*. These increases result in pleiostemony, and were the subject of a recent floral developmental study (Puglisi 2007; Wanntorp et al. 2011) which found two ways in which *Conostegia* species increase in stamen number: 1) by having a large stamen in alternipetalous position with small ones in antipetalous position; or 2) by a process called dedoublement where the stamens split in two (Puglisi 2007; Wanntorp et al. 2011). There appears to be no clear pattern indicating evolutionary relationships, with taxa with either of these developmental pathways being more closely related to another with the opposite pathway.

**Figure 47.**
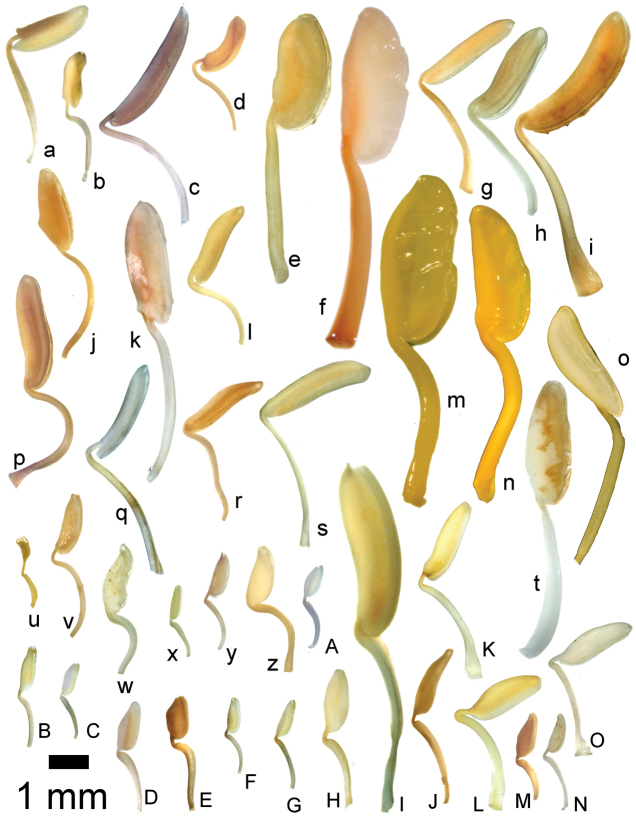
Example of stamens of species in *Conostegia* to scale. **a** *Conostegia lasiopoda* (*R. Kriebel 5651*) **b** *Conostegia monteleagreana* (*R. Kriebel 5747*) **c** *Conostegia ortizae* (*D. Penneys 1857*) **d** *Conostegia tenuifolia* (*R. Kriebel 5773*) **e** *Conostegia bernoulliana* (*R. Kriebel 5540*) **f** *Conostegia bigibbosa* (*R. Kriebel 5522*) **g** *Conostegia bracteata* (*R. Kriebel 5816*) **h** *Conostegia brenesii* (*R. Kriebel 5631*) **i** *Conostegia cuatrecasii* (*R. Kriebel 5673*) **j** *Conostegia fragrantissima* (*R. Kriebel 3174*) **k** *Conostegia icosandra* (*R. Kriebel 5580*) **l** *Conostegia montana* (*R. Kriebel 5544*) **m** *Conostegia macrantha* (*R. Kriebel 5406*) **n** *Conostegia oerstediana* (*R. Kriebel 5338*) **o** *Conostegia pittieri* (*R. Kriebel 5400*) **p** *Conostegia rhodopetala* (*R. Kriebel 5542*) **q** *Conostegia rufescens* (*R. Kriebel 5627*). **r** *Conostegia setosa* (*R. Kriebel 5731*) **s C** *superba* (*R. Kriebel 5582*). **t** *Conostegia volcanalis* (*R. Kriebel 5565*) **u** *Conostegia brenesiana* (*R. Kriebel 3665*). **v** *Conostegia centrosperma* (*R. Kriebel 5690*) **w** *Conostegia cinnamomea* (*R. Kriebel 5330*) **x** *Conostegia consimilis* (*R. Kriebel 5726*) **y** *Conostegia dissitinervia* (*R. Kriebel 5317*) **z** *Conostegia fraterna* (*R. Kriebel 5774*) **A** *Conostegia friedmaniorum* (*R. Kriebel 5641*) **B** *Conostegia galdamesiae* (*R. Kriebel 5736*) **C** *Conostegia grayumii* (*R. Kriebel 5807*) **D** *Clidemia hammelii* (*R. Kriebel 5737*) **E** *Conostegia ombrophila* (*R. Kriebel 3120*). **F** *Conostegia papillopetala* (*R. Kriebel 5718*) **G** *Conostegia peltata* (*R. Kriebel 5658*) **H** *Conostegia povedae* (*F. Oviedo 231*) **I** *Conostegia schlimii* (*R. Kriebel 5614*) **J** *Conostegia shattuckii* (*R. Kriebel 5681*) **K** *Conostegia speciosa* (*R. Kriebel 5677*) **L** *Conostegia subcrustulata* (*R. Kriebel s.n.*) **M** *Conostegia subpeltata* (*R. Kriebel 3643*) **N** *Conostegia trichosantha* (*R. Kriebel 5693*) **O** *Conostegia xalapensis* (*R. Kriebel 5619*).

The posture of the stamens can go from erect, to forming a more or less a 45-degree angle between the filament and the anther (Fig. 47). Species with stamens that form an angle tend to have at least slightly bilaterally symmetric androecia. This condition is common in sections *Australis* and *Conostegia* as well as in the clade composed of *Conostegia osaensis*, *Conostegia plumosa*, *Conostegia speciosa*, *Conostegia subcrustulata*, and *Conostegia xalapensis* within section *Geniculatae*. Bilateral symmetry in *Conostegia* appears also to be related to interactions between the stamen and style during development at least partly the result of the increase in number of stamens. Species in section *Geniculatae*, excluding the clade mentioned above, have stamens that cleanly surround the style and are radially symmetric, yet the flowers of these species might still have slight bilateral symmetry as a result of a gently curving style. In at least two species, *Conostegia fragrantissima* and *Conostegia pittieri*, the stamens are bent away and opposite the style.

**Figure 48.**
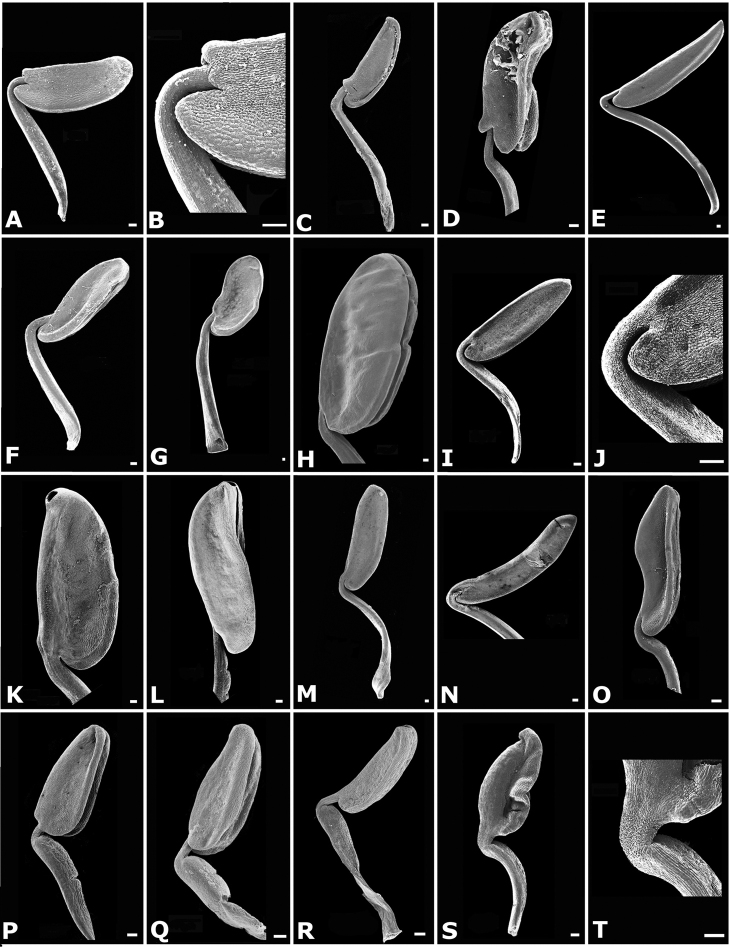
Scanning electron micrographs of the side view of the stamens of *Conostegia*. **A** *Conostegia lasiopoda* (*R. Kriebel 5651*) **B** *Conostegia lasiopoda* close up of anther filament junction (*R. Kriebel 5651*) **C** *Conostegia tenuifolia* (*D. Santamaría 8863*) **D** *Conostegia monteleagreana* (*R. Kriebel 5343*) **E** *Conostegia ortizae* (*D. Penneys 1857*) **F** *Conostegia brenesii* (*R. Kriebel 5631*) **G** *Conostegia icosandra* (*R. Kriebel 5540*) **H** *Conostegia macrantha* (*R. Kriebel 5406*) **I** *Conostegia montana* (*R. Kriebel 5496*) **J** *Conostegia montana* close up of anther filament junction (*R. Kriebel 5496*) **K** *Conostegia oerstediana* (*R. Kriebel 5338*) **L** *Conostegia pittieri* (*R. Kriebel 5400*) **M** *Conostegia rhodopetala* (*R. Kriebel 5542*) **N** *Conostegia rufescens* (*R. Kriebel 5314*) **O** *Conostegia setosa* (*R. Kriebel s.n.*) **P** *Clidemia hammelii* (*R. Kriebel 5317*) **Q** *Conostegia subpeltata* (*R. Kriebel 5347*) **R** *Conostegia trichosantha* (*R. Kriebel 5693*) **S** *Conostegia cinnamomea* (*R. Kriebel 5330*) **T** Close up of anther filament junction in *Conostegia cinnamomea* (*R. Kriebel 5330*). Scale bar: 100 µm.

**Figure 49.**
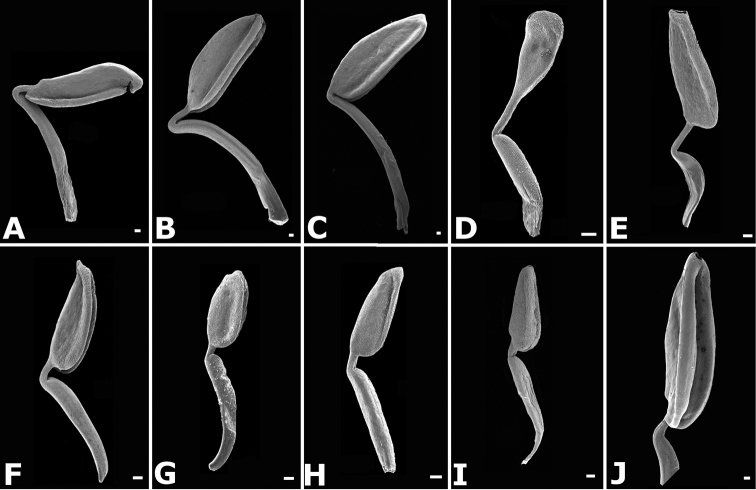
Scanning Electron Micrographs of the side view of the stamens of *Conostegia*. **A** *Conostegia speciosa* (*R. Kriebel 5489*) **B** *Conostegia subcrustulata* (*R. Kriebel s.n.*) **C** *Conostegia xalapensis* (*R. Kriebel 5555*) **D** *Conostegia brenesiana* (*R. Kriebel 3665*) **E** *Conostegia centrosperma* (*R. Kriebel 5690*) **F** *Conostegia dissitinervia* (*R. Kriebel 5377*) **G** *Conostegia friedmaniorum* (*R. Kriebel 5641*) **H** *Conostegia consimilis* (*R. Kriebel 5323*) **I** *Conostegia peltata* (*R. Kriebel 5658*) **J** *Conostegia schlimii* (*R. Kriebel 515*). Scale bar: 100 µm.

The filaments are mostly white to translucent. Sections *Australis* and *Conostegia* lack a clear geniculation towards the apex of the filament, whereas in section *Geniculatae* the geniculation is present in almost every species, except perhaps in *Conostegia fraterna* (Figs 47–49). It is significant that the molecular phylogeny of Kriebel et al. (2015) placed species traditionally recognized in *Conostegia* such as *Conostegia plumosa*, *Conostegia speciosa*, *Conostegia subcrustulata* and *Conostegia xalapensis* in *Geniculatae* and that the latter three species which were studied from field-collected floral material have an evident filament geniculation. This morphological character thus supports their placement within section *Geniculatae*. It has to be considered that some variation does exist. For example, the micrograph of *Conostegia xalapensis* (Fig. 49) does not show the evident geniculation very well whereas the photographs of the pickled stereoscope images of the same specimen do. In general, *Conostegia xalapensis* shows an evident geniculation. Although most species which have filament geniculations present them towards the apex of the filament, in one species, *Conostegia brenesiana*, the geniculation is in the middle of the filament.

**Figure 50.**
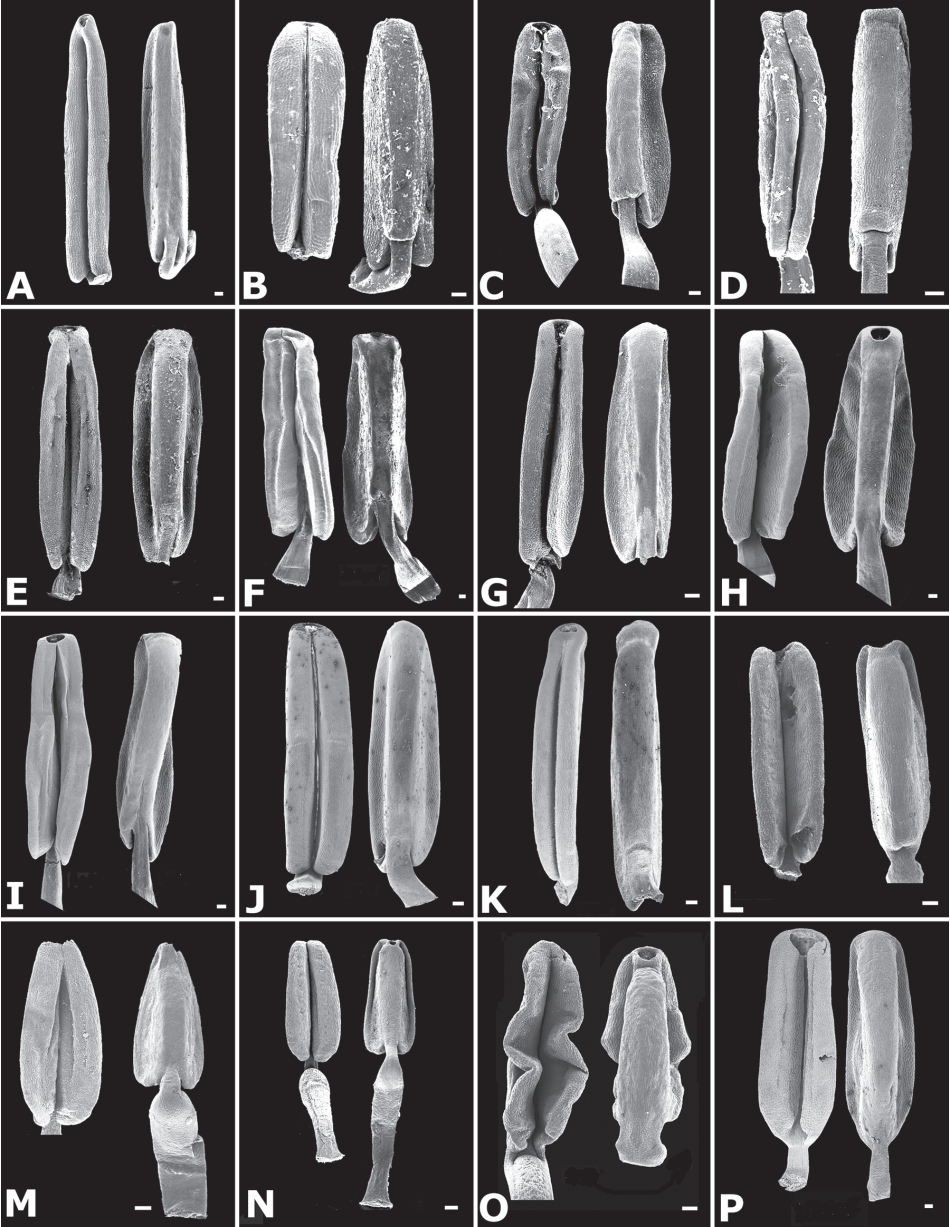
Scanning electron micrographs of the ventral and dorsal view of the stamens in *Conostegia*. **A** *Conostegia ortizae* (*D. Penneys 1857*) **B** *Conostegia lasiopoda* (*R. Kriebel 5651*) **C** *Conostegia monteleagreana* (*R. Kriebel 5343*) **D** *Conostegia tenuifolia* (*D. Santamaria 8863*) **E** *Conostegia brenesii* (*R. Kriebel 5631*) **F** *Conostegia bernoulliana* (*R. Kriebel 5540*) **G** *Conostegia montana* (*R. Kriebel 5496*) **H** *Conostegia oerstediana* (*R. Kriebel 5338*) **I** *Conostegia pittieri* (*R. Kriebel 5400*) **J** *Conostegia rhodopetala* (*R. Kriebel 5542*) **K** *Conostegia rufescens* (*R. Kriebel 5314*) **L** *Clidemia hammelii* (*R. Kriebel 5317*) **M** *Conostegia subpeltata* (*R. Kriebel 5347*) **N** *Conostegia trichosantha* (*R. Kriebel 5693*) **O** *Conostegia cinnamomea* (*R. Kriebel 5330*) **P** *Conostegia subcrustulata* (*R. Kriebel s.n.*). Scale bar: 100 µm.

Anthers in *Conostegia* are almost exclusively yellow to sometimes orange (i.e. *Conostegia fragrantissima*) with some taxa having hues of pink towards the apex in some populations (i.e. *Conostegia rufescens*). In some species particularly in section *Australis* the anthers can be cream colored. Anthers in *Conostegia* lack evident staminal appendages with at the most, some species (e.i., *Conostegia bernoulliana*) having an inconspicuous dorsal bump on the anther connective. The only structure that resembles an anther appendage in *Conostegia* is present in most species of section *Australis*. In these taxa, the anther base is briefly prolonged below the junction of the filament and anther thecae (Fig. 50). This character was recently documented in some species of *Tococa* (Michelangeli 2005) and *Pachyanthus* (Bécquer 2008). The documentation of this type of anther base in *Conostegia* is relevant because it had not been used before in the systematics of the group, and although lost in some species, helps to identify species in section *Australis*.

**Figure 51.**
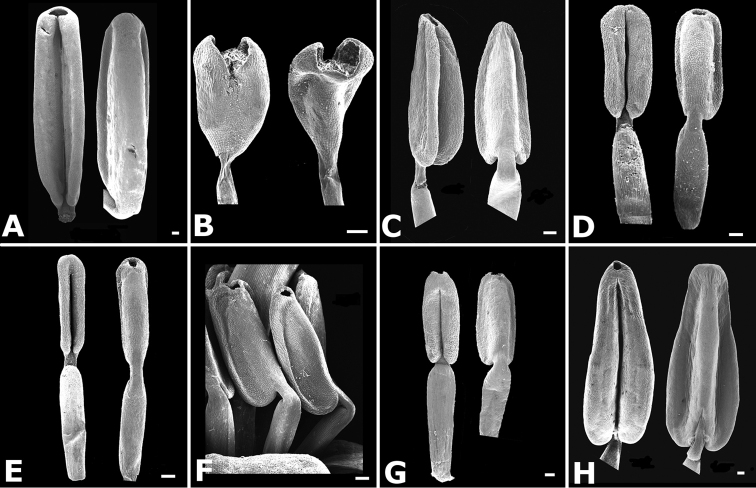
Scanning Electron Micrographs of the ventral and dorsal view of the stamens in *Conostegia*. **A** *Conostegia xalapensis* (*R. Kriebel 5555*) **B** *Conostegia brenesiana* (*R. Kriebel 3665*) **C** *Conostegia dissitinervia* (*R. Kriebel 5377*) **D** *Conostegia friedmaniorum* (*R. Kriebel 5641*) **E** *Conostegia consimilis* (*R. Kriebel 5323*) **F** *Conostegia papillopetala* (*R. Kriebel 5718*) **G** *Conostegia peltata* (*R. Kriebel 5658*) **H** *Conostegia schlimii* (*R. Kriebel 515*). Scale bar: 100 µm.

Anther shape in *Conostegia* varies a lot even when considering only the species treated traditionally in the genus. This is in contrast to what has been previously stated in the literature. In their taxonomic studies of the Miconieae, Judd and Skean (1991) remarked that the non-appendaged anthers characteristic of *Conostegia* are ovoid, tapered to the apex, and open by a small apical pore. These anther characters lead to conclude that the genus did not evolve from within *Miconia*, since members of the latter genus show various modifications of either pore or connective structure. We now know that *Conostegia* is phylogenetically nested within the Miconieae, and furthermore that anthers of its species can be ovoid, oblong, linear, or variations on these shapes (Schnell 1996; Fig. 49). They are definitely not all ovoid. Also, they can be recurved or arcuate making them diffciult to describe. In addition, it is not very useful to say that a small pore characterizes the anthers of *Conostegia*. In fact, most species of Miconieae have small anther pores corresponding to their buzz pollinated syndrome, and for this reason this type of pore was coded as plesiomorphic by Judd and Skean (1991). Even if the size of the pore proves useful in the systematics of *Conostegia*, continuous measurements would have to be employed to determine their utility. The shape of the anther apices and anther pores is quite variable in *Conostegia* and merits further research. This will require careful placement of critically point dried anther apices on stubs and Scanning Electron Microscopy. Some of the variation seen in the pores has to do with the deeply channeled anthers in the ventral surface. In some taxa this deep channel reaches the pore area but in others it does not. *Conostegia brenesiana* stands out because of its very small anther thecae with broad pores which can resemble anthers in Miconia section Cremanium. In most species the pores are terminal to ventrally inclined. Dorsally inclined pores can be found in some species of section *Geniculatae*, and are especially evident in *Conostegia cinnamomea* (Figs 50, 51).

**Figure 52.**
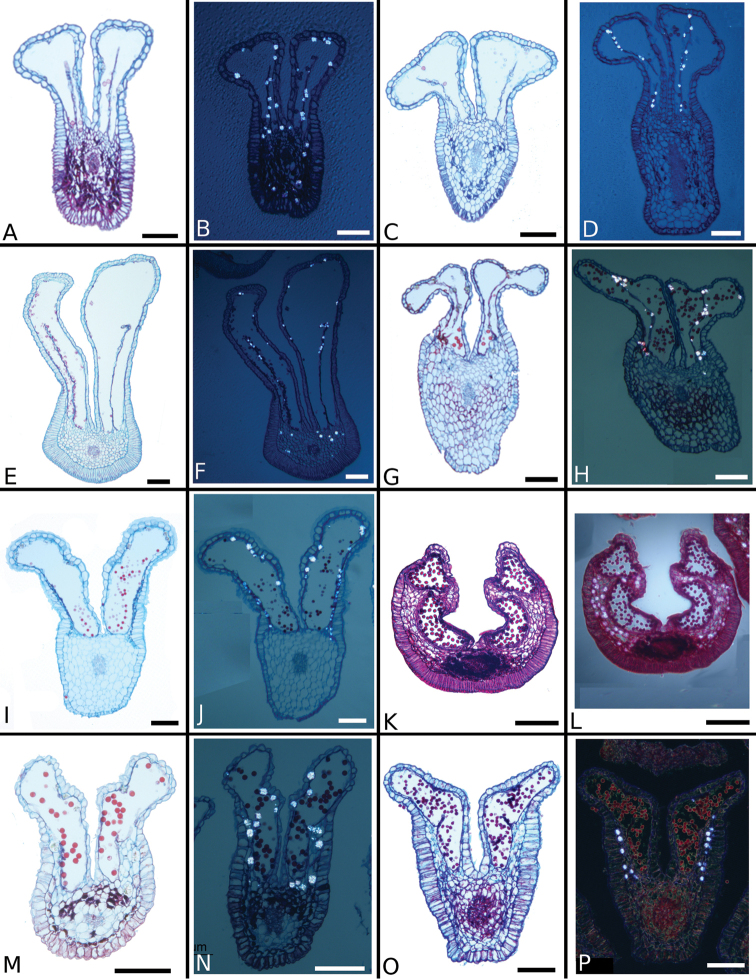
Anther anatomy in *Conostegia*. Each image is a transversal cut of an anther with a photograph under polarized light next to it. **A–H** are examples of anthers with sporangia positioned side by side. **K–P** are examples of anthers with sporangia superposed **A–B** *Conostegia lasiopoda* (*R. Kriebel 5651*) **C–D** *Conostegia tenuifolia* (*D. Santamaría 8863*) **E–F** *Conostegia oerstediana* (*R. Kriebel 5627*) **G–H C** *superba* (*R. Kriebel 5582*) **I–J** *Conostegia subcrustulata* (*R. Kriebel 5653*) **K–L** *Conostegia fraterna* (*R. Kriebel 5774*) **M–N** *Conostegia friedmaniorum* (*R. Kriebel 5641*) **O–P** *Conostegia ombrophila* (*R. Kriebel 3120*). Scale bar: 100 µm.

With respect to the anatomy of the anthers, the sporangia in each anther theca were found to have two general arrangements. In species of section *Australis* and *Conostegia* they tend to be positioned side by side. On the contrary, in section *Geniculatae* they tend to be more-or-less superposed. All anthers were found to have druses. In some species such as *Conostegia lasiopoda* and *Conostegia oerstediana*, the druses are present in the staminal connective, endothecium and anther septum (Fig. 52). In others, they are mostly restricted to the septum like in *Conostegia tenuifolia*. Species in section *Geniculatae* tend to have druses on the proximal half of the outer side of the anthers (as in *Conostegia fraterna*, *Conostegia friedmaniorum*, *Conostegia ombrophila*, and *Conostegia subcrustulata*) (Fig. 52).

**Figure 53.**
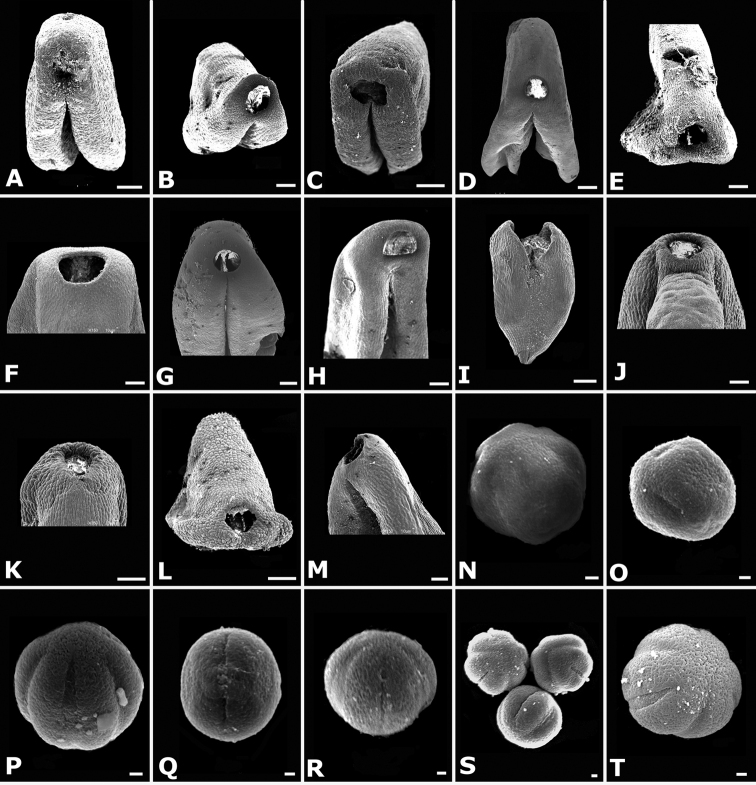
Example of anther pores and pollen in *Conostegia*. **A** *Conostegia lasiopoda* (*R. Kriebel 5651*) **B** *Conostegia ortizae* (*D. Penneys 1857*) **C** *Conostegia tenuifolia* (*D. Santamaría 8863*) **D** *Conostegia bernoulliana* (*R. Kriebel 5540*) **E** *Conostegia brenesii* (*R. Kriebel 5631*) **F** *Conostegia oerstediana* (*R. Kriebel 5338*) **G** *Conostegia rhodopetala* (*R. Kriebel 5542*) **H** *Conostegia rufescens* (*R. Kriebel 5314*) **I** *Conostegia brenesiana* (*R. Kriebel 3665*) **J** *Conostegia cinnamomea* (*R. Kriebel 5330*) **K** *Conostegia consimilis* (*R. Kriebel 5323*) **L** *Conostegia friedmaniorum* (*R. Kriebel 5641*) **M** *Conostegia schlimii* (*R. Kriebel 515*) **N** *Conostegia cinnamomea* (*R. Kriebel 5330*) **O** *Clidemia hammelii* (*R. Kriebel 5317*) **P** *Conostegia schlimii* (*R. Kriebel 515*) **Q** *Conostegia subpeltata* (*R. Kriebel 5347*) **R** *Conostegia macrantha* (*R. Kriebel 5406*) **S** *Conostegia montana* (*R. Kriebel 5496*) **T** *Conostegia oerstediana* (*R. Kriebel 5338*). Scale bar: 100 µm (**A–M**); 10 µm (**N–T**).

Pollen of species in the Melastomataceae has been rarely documented in taxonomic treatments. This may be because the few studies that have been done have revealed little variation (reviewed in Renner 1993). For the species studied, Renner (1993) summarized the pollen as small, tricolporate, radially symmetrical, and isopolar; occasionally grains may have 4, 5 or more colpi, and some grains are dicolporate or heteropolar. Faint to distinct pseudocolpi (or subsidiary colpi, in the terminology of Patel et al. 1984) are usually present. The surface sculpture is smooth and striate to rugulate or rugulate-verrucate. Polyads and tetrads are known from *Tococa spadiciflora* and *Miconia melanotricha* (Patel et al. 1984). In the case of *Conostegia*, Roubik and Moreno (1991) provided the only images known to me of the anatomy of pollen of *Conostegia* species (including *Conostegia shattuckii* as *Miconia shattuckii*). I have found that the pollen characteristics of *Conostegia* agree with Renner’s (1993) general description for the family; this is shown in micrographs provided for several species (Fig. 53).

### Gynoecium

**Figure 54.**
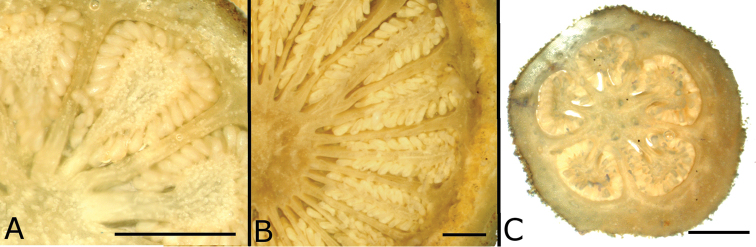
Example of placenta shapes in *Conostegia*. **A** *Conostegia lasiopoda* (*R. Kriebel 5651*) **B** *Conostegia macrantha* (*R. Kriebel 5406*) **C** *Conostegia schlimii* (*R. Kriebel 5614*). Scale bar: 1 mm.

The gynoecium can have from 4 to 25 carpels (Schnell 1996). Placentation is axillary as in most Melastomataceae (Renner 1993). The placenta in most *Conostegia* protrudes into the locule, either in laminar fashion with the ovules attached at the sides, or it is peltate with a clear stipe (Fig. 54). Intermediates are present, and in a few cases (e.g. *Conostegia xalapensis*), the placenta looks like the peltate kind but lacks a clear stipe. Species of sections *Australis* and *Conostegia* have inferior ovaries, and in some species of section *Conostegia* the ovary can be elevated into an evident collar around the style base. Species in these two sections all have glabrous ovary apices. In contrast, species of section *Geniculatae* tend to have ovaries about two thirds inferior and several species have glandular-pubescent ovary apices. A collar around the style base is usually absent in section *Geniculatae*. Species of sections *Australis* and *Conostegia* have a glabrous torus whereas species in section *Geniculatae* can have a glabrous or glandular-puberulent torus. In one species, *Conostegia osaensis*, the torus is beset with lepidote trichomes.

A significant discovery of this study is the deep blue stain recorded in anatomical sections inside the ovary of species in sections *Australis* and *Conostegia* (Fig. 36). On the other hand, this staining is absent in section *Geniculatae*. Staining with Ruthenium red has provided evidence that the substance in question is mucilaginous pectin, and field tests and photographs by Reinaldo Aguilar of with *Conostegia bernoulliana* in the Osa Peninsula have demonstrated that the berries are sticky as would be expected from the presence of mucilage.

**Figure 55.**
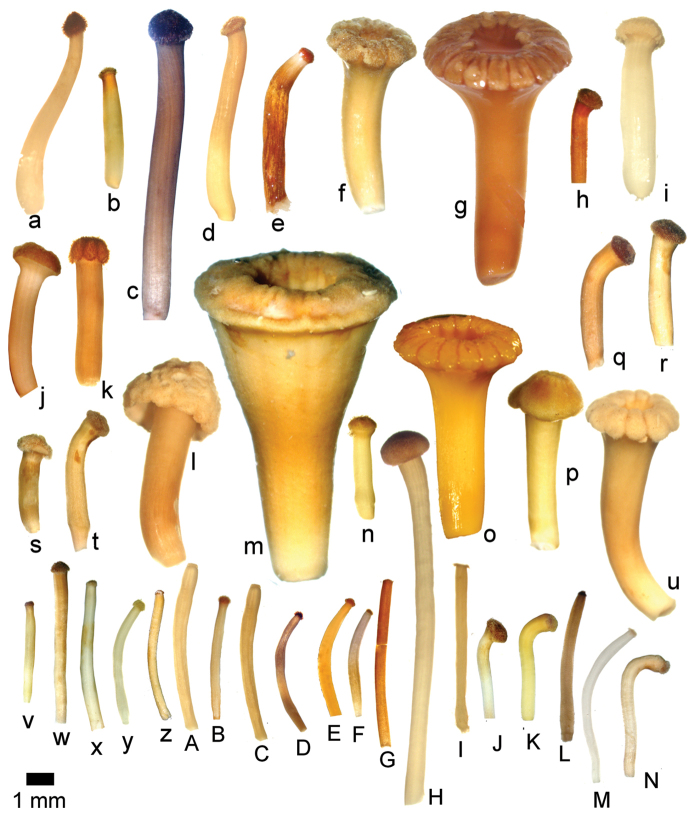
Example of styles of species in *Conostegia* to scale. **a** *Conostegia lasiopoda* (*R. Kriebel 5651*) **b** *Conostegia monteleagreana* (*R. Kriebel 5747*) **c** *Conostegia ortizae* (*D. Penneys 1857*) **d** *Conostegia tenuifolia* (*R. Kriebel 5773*) **e** *Conostegia balbisiana* (*G. Proctor 10285*) **f** *Conostegia bernoulliana* (*R. Kriebel 5540*) **g** *Conostegia bigibbosa* (*R. Kriebel 5522*) **h** *Conostegia bracteata* (*R. Kriebel 5816*) **i** *Conostegia brenesii* (*R. Kriebel 5631*) **j** *Conostegia cuatrecasii* (*R. Kriebel 5673*) **k** *Conostegia fragrantissima* (*R. Kriebel 3174*) **l** *Conostegia icosandra* (*R. Kriebel 5580*) **m** *Conostegia macrantha* (*R. Kriebel 5406*) **n** *Conostegia montana* (*R. Kriebel 5544*) **o** *Conostegia oerstediana* (*R. Kriebel 5338*) **p** *Conostegia pittieri* (*R. Kriebel 5400*) **q** *Conostegia rhodopetala* (*R. Kriebel 5542*) **r** *Conostegia rufescens* (*R. Kriebel 5627*) **s** *Conostegia setosa* (*R. Kriebel 5731*) **t** *Conostegia superba* (*R. Kriebel 5582*) **u** *Conostegia volcanalis* (*R. Kriebel 5565*) **v** *Conostegia brenesiana* (*R. Kriebel 3665*) **w** *Conostegia centrosperma* (*R. Kriebel 5690*) **x** *Conostegia cinnamomea* (*R. Kriebel 5330*) **y** *Conostegia consimilis* (*R. Kriebel 5726*) **z** *Conostegia dissitinervia* (*R. Kriebel 5317*) **A** *Conostegia fraterna* (*R. Kriebel 5774*) **B** *Conostegia galdamesiae* (*R. Kriebel 5736*) **C** *Clidemia hammelii* (*R. Kriebel 5737*) **D** *Conostegia ombrophila* (*R. Kriebel 3120*) **E** *Conostegia papillopetala* (*R. Kriebel 5718*) **F** *Conostegia peltata* (*R. Kriebel 5658*) **G** *Conostegia povedae* (*F. Oviedo 231*) **H** *Conostegia schlimii* (*R. Kriebel 5614*) **I** *Conostegia shattuckii* (*R. Kriebel 5681*) **J** *Conostegia speciosa* (*R. Kriebel 5677*) **K** *Conostegia subcrustulata* (*R. Kriebel s.n*.) **L** *Conostegia subpeltata* (*R. Kriebel 3643*) **M** *Conostegia trichosantha* (*R. Kriebel 5693*) **N** *Conostegia xalapensis* (*R. Kriebel 5619*).

**Figure 56.**
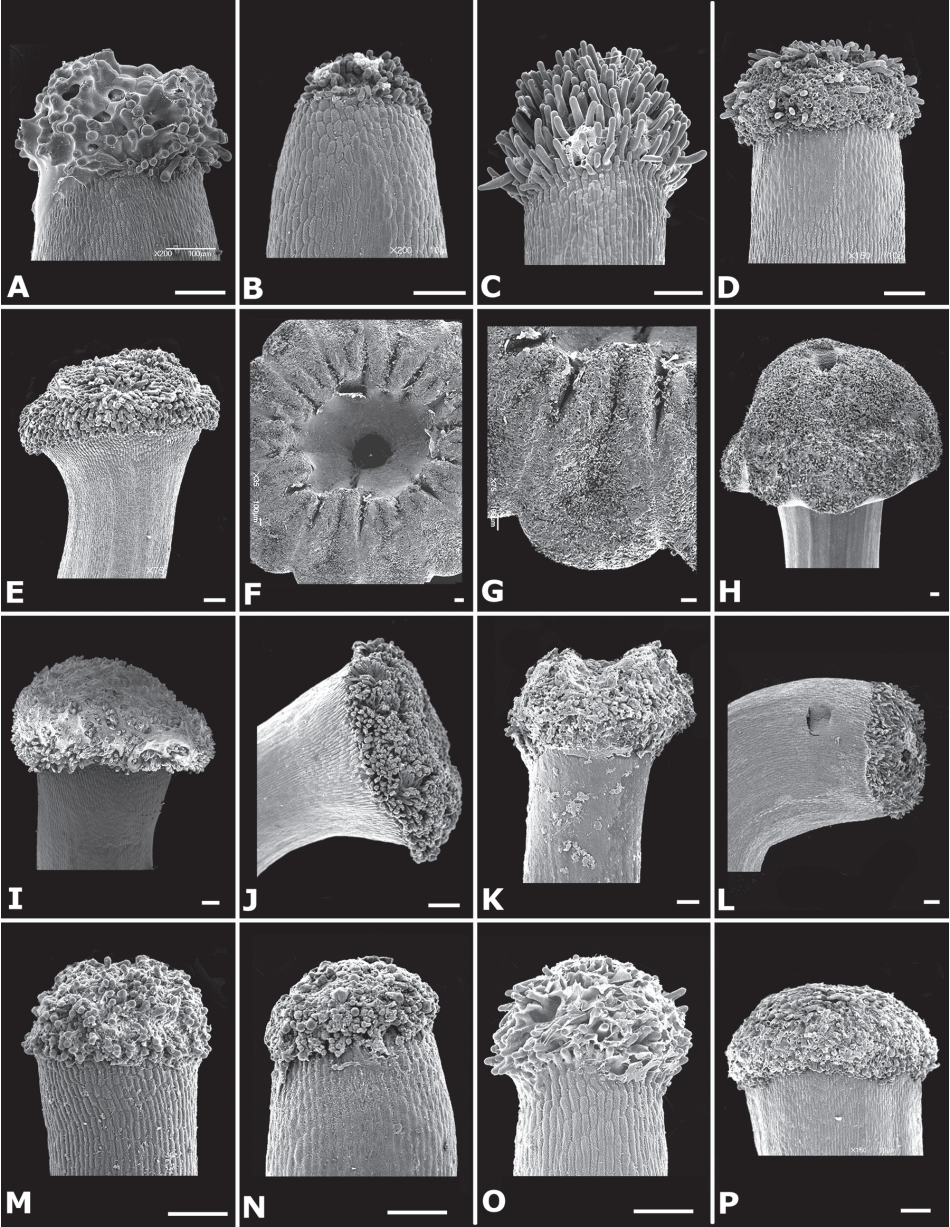
Scanning electron micrographs of stigmas in *Conostegia*. **A** *Clidemia hammelii* (*R. Kriebel 5317*) **B** *Conostegia subpeltata* (*R. Kriebel 5347*) **C** *Conostegia trichosantha* (*R. Kriebel 5693*) **D** *Conostegia cinnamomea* (*R. Kriebel 5330*) **E** *Conostegia montana* (*R. Kriebel 5496*) **F** *Conostegia oerstediana* (*R. Kriebel 5338*) **G** Close up of a stigma lobe of *Conostegia oerstediana* (*R. Kriebel 5338*) **H** *Conostegia pittieri* (*R. Kriebel 5400*) **I** *Conostegia rufescens* (*R. Kriebel 5314*) **J** *Conostegia speciosa* (*R. Kriebel 5489*) **K** *Conostegia tenuifolia* (*D. Santamaría 8863*) **L** *Conostegia xalapensis* (*R. Kriebel 5555*) **M** *Conostegia brenesiana* (*R. Kriebel 3665*) **N** *Conostegia dissitinervia* (*R. Kriebel 5377*) **O** *Conostegia consimilis* (*R. Kriebel 5323*) **P** *Conostegia schlimii* (*R. Kriebel 515*). Scale: 100 µm.

*Conostegia* can be said to have the highest diversity in style and stigma morphology in the whole Melastomataceae (Fig. 55). Most notable is the presence of crateriform lobed stigmas in several species (Figs 55, 56). These crateriform stigmas are unique to a subclade of *Conostegia*, although in some species the crater is reduced to almost absent but the lobes mostly remain present. Besides these obvious outliers, the diversity is still pronounced, ranging from short, stout, straight or apically hooked styles, to long, straight or gently curving styles. Stigmas can be punctiform, truncate, or variously enlarged (Fig. 55). Styles in *Conostegia* can be exserted or not. Biologically, this exertion corresponds to the maintenance of herkogamy (see Reproductive Biology section). Although the posture of the style appears to be taxonomically informative, this posture can vary through time within the same individual in some species. In species with exserted styles, two general trends are noticeable. The first trend, seen in species of section *Australis* as well as *Conostegia schlimii* of section *Geniculatae*, is one in which the flower is either facing upward, or horizontally and thus the style is erect or horizontal. In both these similar postures, the style tends to surve gently towards the apex resulting in a more or less S shape. The other trend in species with exserted styles is almost the norm in species of section *Geniculatae* and involves deflexed styles. It should be noted that in this revision, exemplary flowers of all sections are presented in erect position for convenience (see Figs 40 through 43), but in the taxonomic treatment, images of live flowering plants display the natural posture of the flowers.

There are two main clades where the style is not exserted beyond the stamens (not herkogamous). The first corresponds to section *Conostegia* and the other to the clade comprised of *Conostegia osaensis*, *Conostegia plumosa*, *Conostegia speciosa*, *Conostegia subcrustulata*, and *Conostegia xalapensis*. Aside from the crateriform and or lobed stigma clade, which are derived from section *Conostegia*, all remaining taxa with a short style appear to have either a straight style or one that bends upward below the stigma. Within section *Conostegia*, style posture appears to change in most of its members, particularly in the crateriform or lobed stigma clade. The few observations of species in this group (Stratton 1989; Schnell 1996) suggest their flowers can last about two days. During this time the style bends downward usually blocking numerous stamens. Preliminary experiments (Kriebel unpublished data) show that if the style is removed, the stamens can unveil normally and that if the stamens are removed the style still bends (see photographs in section on Reproductive biology).

**Figure 57.**
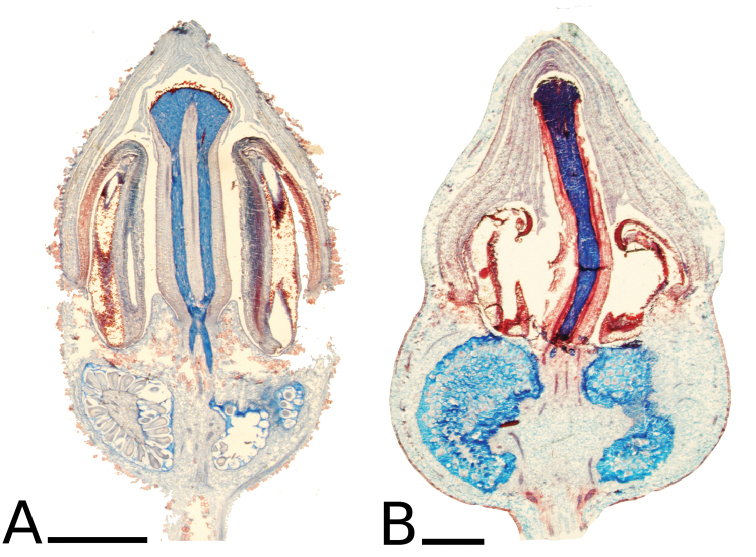
Two examples of longitudinal sections of flowers of *Conostegia*. Note the presence of a stele within the style in **A** *Conostegia superba* (*R. Kriebel 5582*) and the lack of a stele in **B** *Conostegia tenuifolia* (*D. Santamaría 8863*). Scale bar: 1 mm.

**Figure 58.**
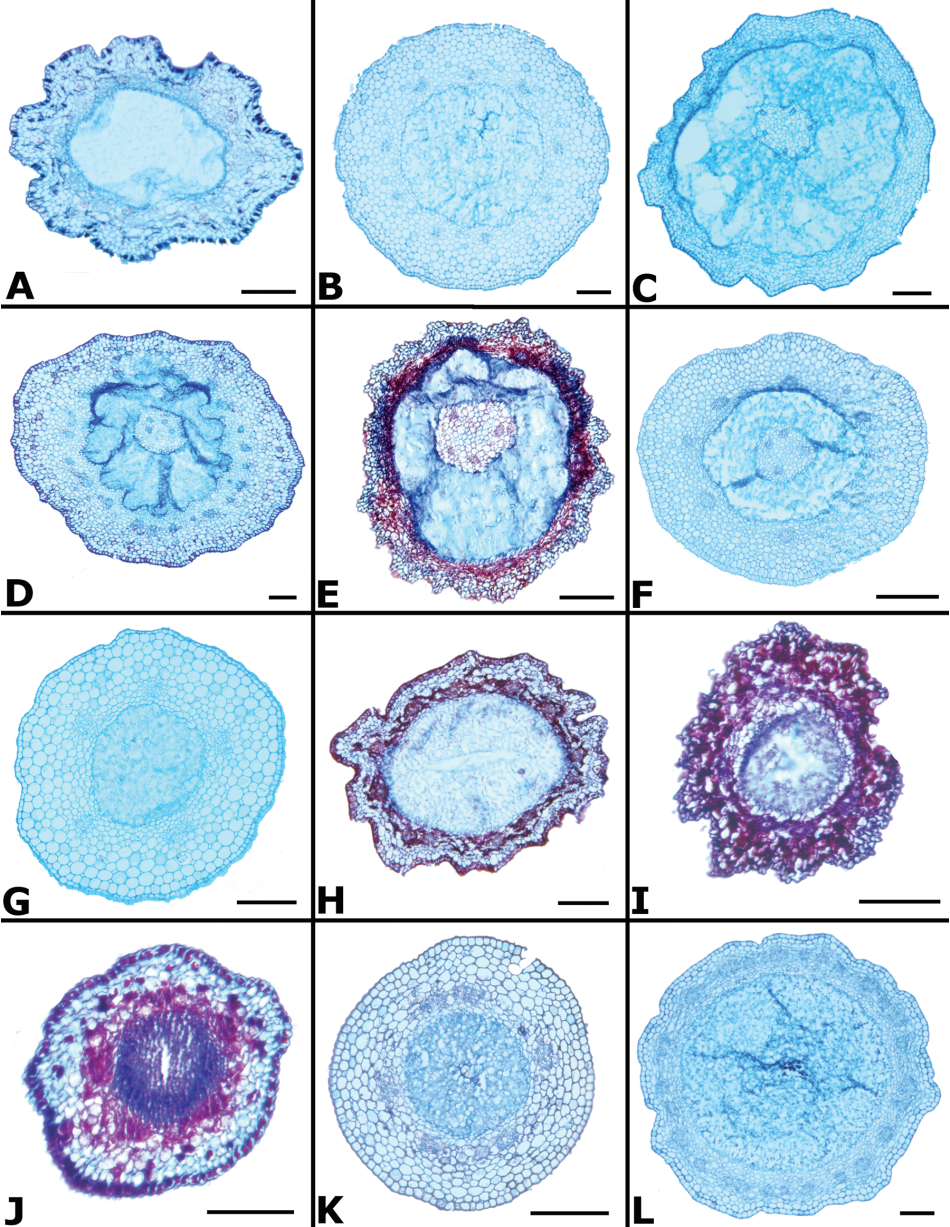
Style transversal anatomy in *Conostegia*. **A** *Conostegia lasiopoda* (*R. Kriebel 5651*) **B** *Conostegia tenuifolia* (*D. Santamaría 8863*) **C** *Conostegia brenesii* (*R. Kriebel 5631*) **D** *Conostegia pittieri* (*R. Kriebel 5543*) **E** *Conostegia setosa* (*R. Kriebel 5731*) **F** *Conostegia superba* (*R. Kriebel 5582*) **G** *Conostegia friedmaniorum* (*R. Kriebel 5641*) **H** *Clidemia hammelii* (*R. Kriebel 5317*) **I** *Conostegia schlimii* (*R. Kriebel 515*) **J** *Conostegia subcrustulata* (*R. Kriebel 5653*) **K** *Conostegia trichosantha* (*R. Kriebel 5693*) **L** *Conostegia xalapensis* (*R. Kriebel 5629*). Scale bar: 100 µm.

In the styles of the *Conostegia* clade, another outstanding aspect involves the presence of a stele within for their entire length (Figs 57, 58). Specifically, section *Conostegia* and convergently *Conostegia ortizae* in section *Australis* present this phenomenon. This stele is unknown in the rest of the Melastomataceae. Schnell (1996) was the first to document the stele in two species which are phenotypically divergent but belong to section *Conostegia*. This character is helpful to confirm the hypothesis, derived by optimizing morphological traits on the molecular phylogeny, that the calyptra and short style seen in the clade composed of *Conostegia osaensis*, *Conostegia plumosa*, *Conostegia speciosa*, *Conostegia subcrustulata*, and *Conostegia xalapensis* must have evolved independently from the similar morphology seen in section *Conostegia*. No species including the ones just mentioned of section *Geniculatae* were found to have a vascular cylinder inside the style. These results also coincide with Schnell’s (1996) observations of a lack of a cylinder in *Conostegia subcrustulata*. The stele found in the style of *Conostegia ortizae*, in general, differs from those in species of section *Conostegia* because it has fewer vascular traces.

### Fruits

**Figure 59.**
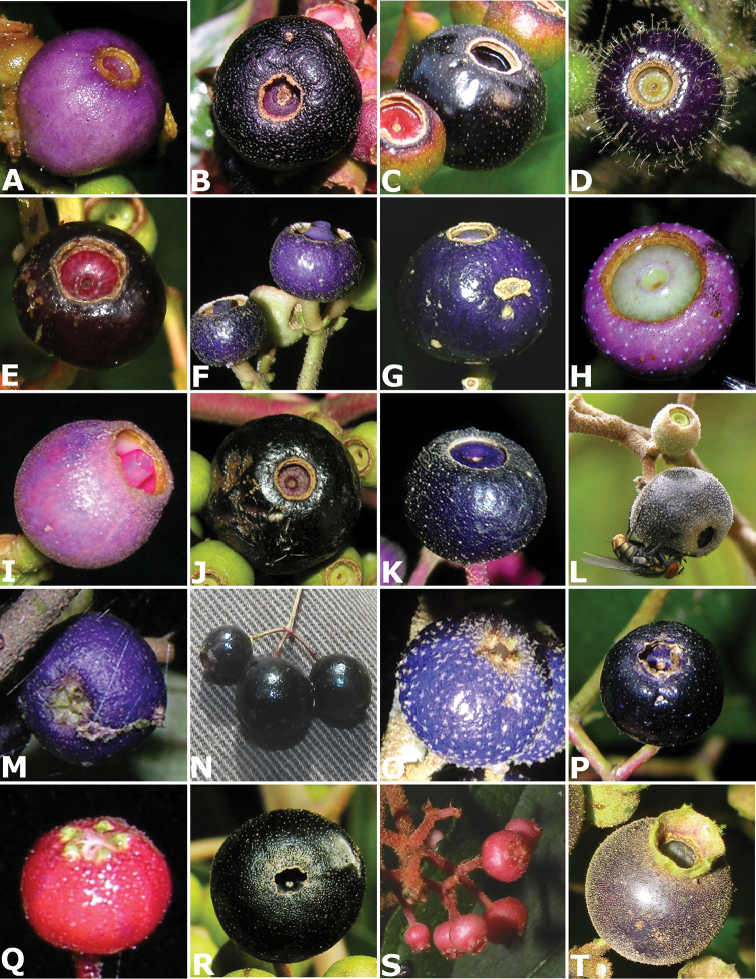
Berries of some species of *Conostegia*. **A** *Conostegia lasiopoda* (*R. Kriebel 5651*) **B** *Conostegia monteleagreana* (*R. Kriebel 5354*) **C** *Conostegia polyandra* (*X. Cornejo 8126*) **D** *Conostegia caelestis* (*R. Kriebel 5588*) **E** *Conostegia cuatrecasii* (*R. Kriebel 5673*) **F** *Conostegia icosandra* (*R. Kriebel 5580*) **G** *Conostegia montana* (*R. Kriebel 5446*) **H** *Conostegia oerstediana* (*R. Kriebel 5338*) **I** *Conostegia rufescens* (*E. Saliceti s.n.*) Photograph by E. Saliceti. **J** *Conostegia superba* (*R. Aguilar 12103*) **K** *Conostegia subcrustulata* (*R. Kriebel 5333*) **L** *Conostegia xalapensis* (*R. Kriebel s. n.*) **M** *Conostegia ombrophila* (*R. Kriebel 5396*) **N** *Conostegia pittieri* (*R. Kriebel 5757*) **O** *Conostegia calocoma* (*R. Kriebel s. n.*) **P** *Conostegia dissitiflora* (*R. Kriebel 5378*) **Q** *Conostegia friedmaniorum* (*R. Kriebel 5497*) **R** *Conostegia osaensis* (*R. Aguilar s. n.*) **S** *Conostegia shattuckii* (*R. Kriebel 5688*) **T** *Conostegia schlimii* (*R. Kriebel 5329*). All photographs by the author except where specified.

Fruits of most species traditionally recognized as *Conostegia* have a truncate apex produced by the dehiscence or breaking of the calyptra. In addition, some have a prominent ovary apex that is retained in the berry and can be highly elevated in fruit (Fig. 59). Fruits of some species such as those of *Conostegia oerstediana* have a pleasant taste.

### Seeds

Almost all species of *Conostegia* have small seeds averaging about 0.5 mm in length and varying in number from hundreds to thousands in a single berry. The most significant deviation from this pattern is observed in the clade comprised of *Conostegia osaensis*, *Conostegia plumosa*, *Conostegia speciosa*, *Conostegia subcrustulata*, and *Conostegia xalapensis*, where seeds are fewer in number and average about double in length.

**Figure 60.**
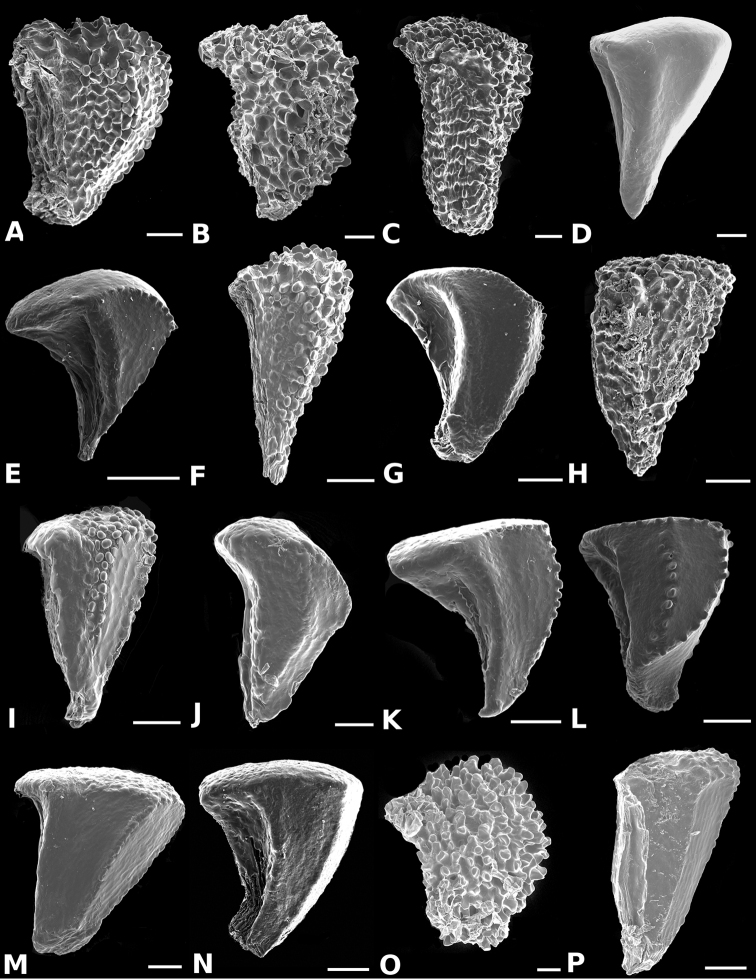
Seeds in *Conostegia*. **A** *Conostegia allenii* (*M. Nepokroeff 722*) **B** *Clidemia hammelii* (*R. Kriebel 1420*) **C** *Conostegia ombrophila* (*R. Kriebel 4887*) **D** *Conostegia pittieri* (*D. Quiroz 800*) **E** *Conostegia trichosantha* (*F. Almeda 6491*) **F** *Conostegia cinnamomea* (*R. Hartman 12257*) **G** *Conostegia brenesiana* (*R. Kriebel 4614*) **H** *Conostegia dissitiflora* (*R. Kriebel 5378*) **I** *Conostegia dissitinervia* (*R. Kriebel 5377*) **J** *Conostegia peltata* (*R. Kriebel 5658*) **K** *Conostegia grayumii* (*M. grayum 1834*) **L** *Conostegia consimilis* (*A. Jiménez 2326*) **M** *Conostegia oligocephala* (*R. Thorne 41087*) **N** *Conostegia pendula* (*D. Santamaría 6672*) **O** *Conostegia calocoma* (*R. Kriebel s.n.*). Scale bar: 100 µm.

**Figure 61.**
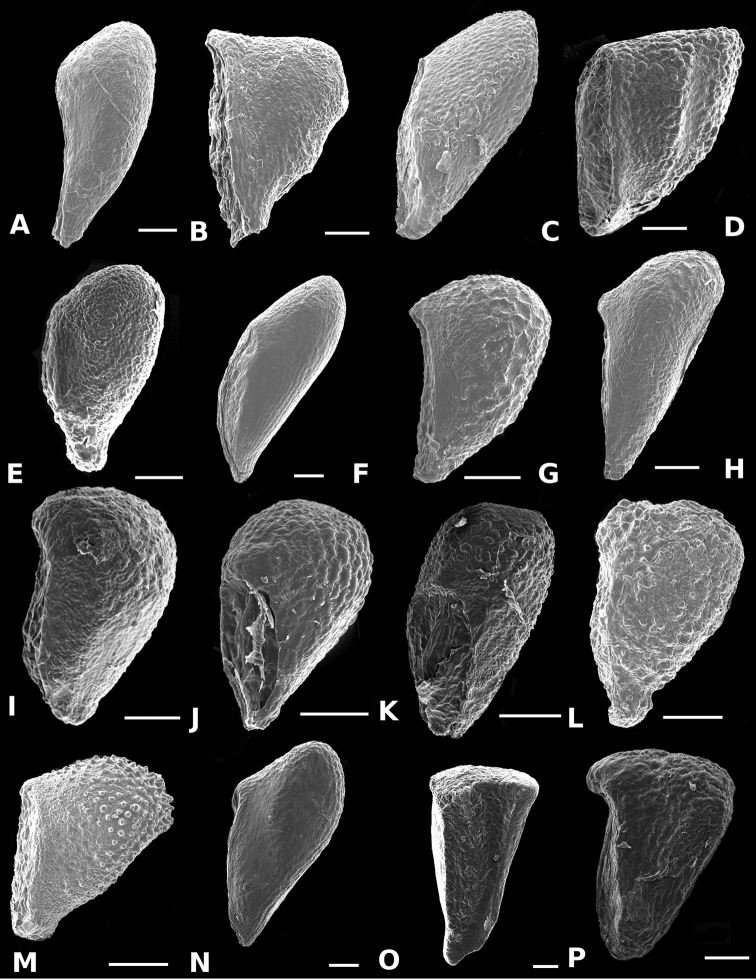
Seeds in *Conostegia*. **A** *Conostegia brenesii* (*A. Tonduz 12580*) **B** *Conostegia caelestis* (*R. Kriebel 5617*) **C** *Conostegia chiriquiensis* (*E. Alfaro 2365*) **D** *Conostegia hirtella* (*A. Molia 3189*) **E** *Conostegia icosandra* (*F. Ventura 20608*) **F** *Conostegia macrantha* (*C. Schnell 1077*) **G** *Conostegia micrantha* (*R. Kriebel 694*) **H** *Conostegia montana* (*W. Stevens 25434*) **I** *Conostegia centronioides* (*E. Little 6215*) **J** *Conostegia extinctoria* (*H. David 1227*) **K** *Conostegia ortizae* (*D. Penneys 1857*) **L** *Conostegia lasiopoda* (*L. Fournier 303*) **M** *Conostegia monteleagreana* (*F. Almeda 6075*) **N** *Conostegia pittieri* (*C. Todzia 2045*) **O** *Conostegia plumosa* (*P. Gentle 9258*) **P** *Conostegia polyandra* (*P. Azevedo 6905*). Scale bar: 100 µm.

**Figure 62.**
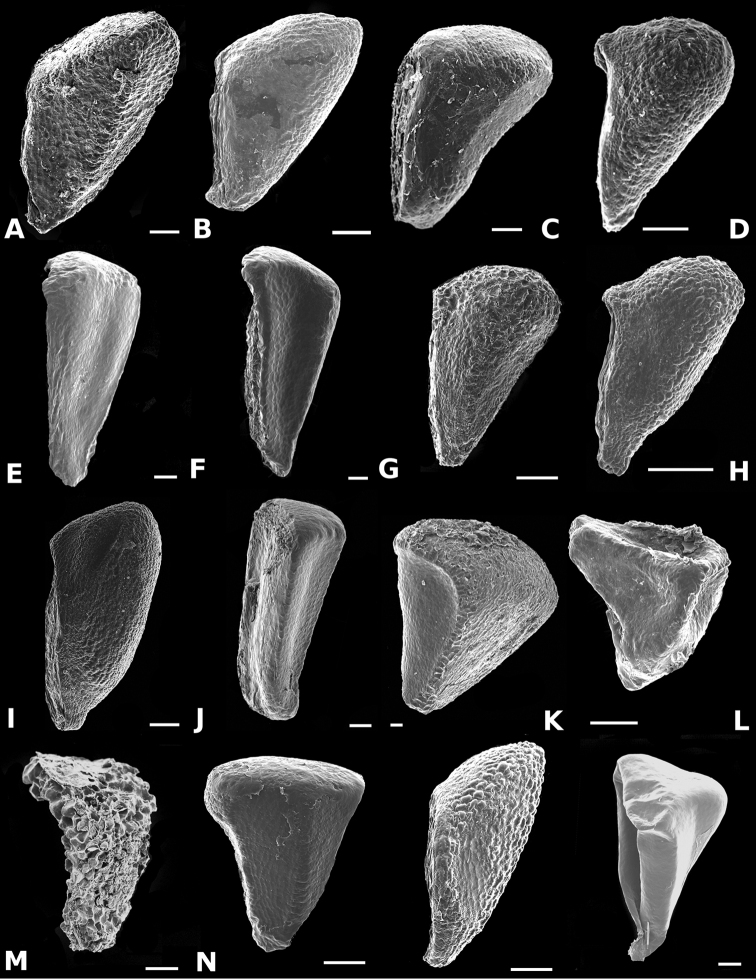
Seeds in *Conostegia*. **A** *Conostegia procera* (*N. Britton 3607*) **B** *Conostegia rhodopetala* (*C. Schnell 1081*) **C** *Conostegia rufescens* (*O. Valverde 13*) **D** *Conostegia setosa* (*D. Solano 1448*) **E** *Conostegia speciosa* (*F. Almeda 2863*) **F** *Conostegia subcrustulata* (*R. Kriebel 5333*) **G** *Conostegia superba* (*J. Steyermark 47891*) **H** *Conostegia tenuifolia* (*A. Rodríguez 11693*) **I** *Conostegia volcanalis* (*E. Matuda 2644*) **J** *Conostegia xalapensis* (*A. Soto 1772*) **K** *Conostegia osaensis* (*R. Aguilar 12228*) **L** *Conostegia papillopetala* (*R. Kriebel 5718*) **M** *Conostegia ecuadorensis* (*S. Stern 328*) **N** *Conostegia balbisiana* (*N. Britton 558*) **O** *Conostegia bracteata* (*E. Killip 12158*) **P** *Conostegia shattuckii* (*R. Kriebel 5688*). Scale bar: 100 µm.

Seeds in *Conostegia* are mostly ovoid except for section *Geniculatae* where several species have pyramidal seeds (Figs 60–62) (Ocampo and Almeda 2013). In some cases like *Conostegia brenesiana* most seeds are pyramidal but ovoid seeds can sometimes be found in the same fruit. Angles are absent except for some species in section *Geniculatae* that can be strongly angled. The lateral symmetrical plane is ovate in sections *Australis* and section *Conostegia* and frequently triangular in section *Geniculatae*. Tubercles on the seed surface are evident in in section *Geniculatae* and in *Conostegia monteleagreana* of section *Australis* but are absent in section *Conostegia*.

The conclusion of this study is that despite the difficulty in coding seed characters, species of section *Australis* and section *Conostegia* have very similar seeds in being ovoid and smooth, whereas there is a lot of variation in seed morphology is in section *Geniculatae*, with ovoid to pyramidal seeds that frequently have angles and tubercle-like cells present just on the angles or covering the whole seed.

## Taxonomic treatment

### Conostegia

Taxon classificationPlantaeMyrtalesMelastomataceae

D. Don

Conostegia D. Don, Mem. Wern. Soc. 4: 316. 1823. Lectotype: Conostegia procera (Sw.) D. Don ex DC. (= Melastoma procera Sw.), designated here.
Synodon Raf., Sylva Tellur. 95. 1838. Lectotype:—Conostegia montana (Sw.) D. Don ex DC.(= Melastoma montana Sw.), designated here.

#### Description.

Small shrubs to medium sized trees with slender and terete to stout and tetragonal branches; branches glabrous to variously pubescent with simple, stellate, stipitate-stellate, lepidote, or highly varying dendritic trichomes, usually the trichomes types not in combination. Twigs with or without nodal lines which can be obscured by the indument. Petiole absent or usually present, in one species (*Conostegia bigibbosa*) and some populations of another (*Conostegia montana*) with two tubercles near the apex abaxially. Leaves subisophyllous to isophyllous or rarely anisophyllous (most markedly in *Conostegia henripittieri*), membranaceous, rarely coriaceous, several species with somewhat leathery texture, particularly the ones with a hypodermis, nerved to strongly plinerved and if the latter, frequently asymmetric, entire to crenate, denticulate or serrate, adaxially usually glabrous, abaxially glabrous to variously pubescent with simple, stellate, stipitate-stellate, lepidote, or highly varying dendritic trichomes, the surface obscured by indument in a few species, all species with tiny glands on the surface, the apex acute to caudate, the base peltate, rounded, cordate, acute or long decurrent, with pouch-like formicaria at the base in two species (*Conostegia dentata* and *Conostegia setosa*), with pocket domatia at the base abaxially in three species (*Conostegia ecuadorensis*, *Clidemia hammelii* and *Conostegia ombrophila*), and with evident tuft domatia in one species (*Conostegia procera*). Inflorescences pseudoaxillary or terminal, erect or deflexed, small dichasia or small to large panicles, few to many flowered, branching at or above the base, in a few cases the branches terminating in bracteate glomerules (i.e. *Conostegia monteleagreana*), bracts and bracteoles deciduous and sometimes appearing absent, or persistent and especially the bracteoles sometimes forming a nodal collar around the inflorescence branches, accessory branches present especielly in taxa with terminal, paniculate inflorescences. Flowers diplostemonous or pleiostemonous, 4-12-merous; calyx calyptrate or not, if calyptrate with or without calyx teeth and the calyptras varying from very thin to very thick, glabrous to pubescent like the hypanthium and with or without sclereids, if the calyx not calyptrate, fused in some species and rupturing irregularly at anthesis into irregular lobes, in non calyptrate species the calyx lobes mostly inconspicuous and similar to the calyx teeth, not conspicuous except in *Conostegia incurva*; petals reflexed or generally spreading, linear-oblong to broadly obovate, the apex acute to rounded, evidently clawed at the base at least on one species (*Conostegia schlimii*), mostly glabrous, in at least one species conspicuously papillose (*Conostegia papillopetala*), translucent to mostly white, in a few species pink or purple, the petals cells on the adaxial surface mostly rounded in species with colored petals and flattened in species with translucent petals. Stamens 8 to ca. 52, arranged neatly around the style in diplostemonous taxa and less neatly but similarly in most pleiostemonous taxa, a few species with the stamens bent to one side of the flower (i.e., *Conostegia fragrantissima* and *Conostegia pittieri*), filaments translucent white to white, with or without an evident geniculation near the apex, the connective not prolonged nor appendaged, the filament anther insertion transitioning smoothly or with a “shoulder” or abrupt step, anthers linear, oblong, elliptic or ovate, apically rounded or acute, basally acute, rounded or sagittate, yellow, less frequently whitish or with pinkish hues, laterally compressed, with druses in the endothecium, the pore oriented totally upward, somewhat ventrally inclined or less frequently dorsally inclined, usually small, broad in one species (*Conostegia brenesiana*), anther sporangia positioned laterally or more or less superposed. Ovary from almost superior to usually totally inferior, 4–25 locular, the placentas within each locule laminar, triangular or peltate, with or without mucilage inside; the style exserted beyond the stamens or not, cylindrical and linear, to shaped as an inverted crateriform cone, when linear, sometimes gently bending to abruptly bending below the stigma, hollow or with a stele within, the stigma lobed or not, papillose. Berries small to large, mostly purple, sweet and usually pleasant to human taste; seeds mostly numerous ad small (ca. 0.5 mm long), few and large in a few species, largest in *Conostegia osaensis* (ca. 1.5 mm long), ovoid to pyramidal, sometimes evidently angular, the testa smooth in most species, tuberculate all over in some, and in fewer still, with the tubercles restricted to the angles.

Species concepts in this revision for the most part follow the morphological species concept as defined by Cronquist (1978) in which species are “the smallest groups that are consistently and persistently distinct, and distinguishable by ordinary means.” It is a problematic concept partly because defining “distinct” is subjective and varies across organisms. Nonetheless, it is the most common species concept applied to plants (McDade 1995), and an effort was made to recognize only species that appear distinct. This author views species as hypotheses that are subject to testing. Thus the reader should be aware that some species may someday prove to be synonymous with others, and some species complexes might prove to be made of cryptic species in need of recognition. Properties of other species concepts are desirable, such as defining species based on monophyly (e.g. the phylogenetic species concept), but the lack of enough representative specimens in the phylogenetic analyses of many species, as well as appropriate molecular markers to ascertain clades (species) with confidence, and inherent biological processes such as incomplete lineage sorting, preclude much of its use in the present treatment. In this monograph when a negative measurement is given for the distance between the anther and the stigma, this means that the anthers are below the stigma for that length.

#### Key to the sections and species of *Conostegia*

**Table:** 

1	Sepals not fused into a calyptra, sometimes rupturing late at anthesis into irregular lobes, calyx teeth present at the level of the torus or slightly above; flowers diplostemonous (sect. *Geniculatae*, pro majore parte)	
2	Leaves discolorous, the abaxial surface white to rusty and concealing the surface	
3	Sepals fused and rupturing at anthesis into irregular lobes	
4	Sepals the same texture as the hypanthium, persisting through flowering; anther apex rounded	***Conostegia centrosperma***
4'	Sepals hyaline and almost not visible during flowering; anther apex acute	***Conostegia dissitinervia***
3'	Sepals not evidently fused at anthesis and rupturing into irregular lobes	
5	Calyx teeth adnate to the calyx lobes, barely discernable and not projecting beyond them; petals papillose	***Conostegia fulvostellata***
5'	Calyx teeth oblong or linear, usually evident and exceeding the calyx lobes; petals glabrous	
6	Abaxial indument rusty colored; calyx teeth usually longer than 2 mm and hooked	***Conostegia incurva***
6'	Abaxial indument white; calyx teeth up to 1.5 mm long and not hooked	***Conostegia oligocephala***
2'	Leaves not discolorous, the abaxial surface visible	
7	Inflorescence axillary or appearing so	
8	Leaves peltate; indument on abaxial leaf surface and hypanthia stellate; flowers 4-merous	***Conostegia subpeltata***
8'	Leaves not peltate; indument on abaxial leaf surface and hypanthia glabrous or stellulate lepidote; flowers 4–5 merous	
9	Leaves and hypanthium glabrous or appearing so	
10	Leaf base decurrent on the petiole and lacking domatia at the base abaxially; inflorescence of pedunculate glomerules with sessile flowers and ovate bracteoles	***Conostegia fraterna***
10'	Leaf base acute and usually with domatia at the base abaxially; inflorescence a laxly branched dichasium with pedicellate flowers and linear to lanceolate bracteoles	
11	Leaves with four domatia located abaxially at the base of the leaf	***Conostegia ecuadorensis***
11'	Leaves lacking, or usually with two domatia located abaxially at the base of the leaf	***Conostegia ombrophila***
9'	Leaves and hypanthium densely pubescent with long simple or dendritic hairs (some populations of *Conostegia allenii* with glabrous leaves)	
12	Petals acuminate at the apex	***Conostegia trichosantha***
12'	Petals rounded at the apex	
13	Leaves with domatia at the base abaxially	***Clidemia hammelii***
13'	Leaves lacking domatia at the base abaxially	
14	Leaves sessile or subsessile	***Conostegia allenii***
14'	Leaves distinctly petiolate	***Conostegia foreroi***
7'	Inflorescence terminal	
15	Leaves peltate	***Conostegia peltata***
15'	Leaves petiolate from the lamina base or sessile, not peltate	
16	Hypanthium densely covered with simple hairs sometimes intermixed with stellate hairs; the leaves with simple hairs on both surfaces or just on the abaxial surface; calyx teeth linear to subulate and 2–4 mm long	***Conostegia allenii***
16'	Hypanthium glabrous to densely covered with stellate and or dendritic hairs; the leaves adaxially glabrous and abaxially glabrescent to densely covered with stellate or dendritic trichomes mostly on the veins; calyx teeth tuberculate to triangular or, if linear, usually less, but up to, 2 mm long	
17	Inflorescences with sessile flowers in bracteate clusters	
18	Leaves sessile and the base decurrent	***Conostegia povedae***
18'	Leaves petiolate, the base obtuse to acute or rounded	
19	Calyx lobes not evidently fused at anthesis and rupturing, ovate to orbicular and mucronate, the same consistency as the hypanthium	***Conostegia colliculosa***
19'	Calyx lobes fused at anthesis, hyaline, and rupturing into irregular lobes	***Conostegia galdamesiae***
17'	Inflorescences with pedicellate flowers not in clusters	
20	Calyx fused in bud and rupturing into irregular hyaline lobes	
21	Flower 4 merous	***Conostegia calocoma***
21'	Flower 5 merous	
22	Leaves subsessile with rounded to cordate bases; plants overall appearing glabrous but bearing minute inconspicuous trichomes almost invisible to the naked eye; stamens alternately two sizes	***Conostegia dissitiflora***
22'	Leaves clearly petiolate or if short petiolate (sometimes in *Conostegia papillopetala*) with acute to decurrent bases; plants evidently pubsecent, especially on the apical nodes and leaf abaxial surface; all stamens mostly of one size	
23	Petals linear oblong; Costa Rican endemics	
24	Leaves 5 plinerved; indument of stem apices of asperous headed hairs only; inflorescence branched at the base	***Conostegia friedmaniorum***
24'	Leaves 7 plinerved; indument of stem apices lanate at least in part; inflorescence branched above the base	***Conostegia pendula***
23'	Petals ovate; Panamanian endemics	
25	Flower regularly arranged at the end of the inflorescence branches and with pink papillose petals	***Conostegia papillopetala***
25'	Flowers usually clustered at the end of the inflorescence branches and with white glabrous petals	***Conostegia galdamesiae***
20'	Calyx not fused in bud and the lobes not rupturing into irregular hyaline lobes, the lobes mostly low and undulate	
26	Flowers large, the petals 9 mm long or more; stigma capitate; ovary totally inferior	***Conostegia schlimii***
26'	Flowers small, the petals usually no more than 5 mm long; stigma truncate to punctiform; ovary at least partly superior	
27	Leaves 5–7 nerved with a broadly rounded to cordate base	***Conostegia shattuckii***
27'	Leaves 5–7 plinerved with rounded to mostly acute to attenuate base	
28	Petals magenta	***Conostegia jefensis***
28'	Petals white	
29	Petals ovate; anthers with broad pores	***Conostegia brenesiana***
29'	Petals linear-oblong; anthers with small pores	
30	Leaves 3 plinerved, the leaf blade 1.7–9.5 × 0.4–1.7 cm	***Conostegia iteophylla***
30'	Leaves 5 plinerved, the leaf blade 4.2–28.4 × 1.4–9.2 cm	***Conostegia consimilis***
1'	Sepals fused into a calyptra, calyx teeth absent, or if present, restricted to the apex of the calyptra; flowers usually pleiostemonous	
31	Calyptra often rupturing at one side or breaking into pieces; style exserted (section *Australis* and *Conostegia cinnamomea*)	
32	Leaf bases with pouch like formicaria; indument of the stems with long simple hairs	***Conostegia dentata***
32'	Leaf bases without pouch like formicaria; indument of stems absent or of stellate, dendritic or asperous hairs	
33	Stem apices and leaf abaxial surface glabrous or nearly so	
34	Flowers in glomerules at the end of inflorescence branches and subtended by evident bracts and bracteoles; stamens usually less, but up to 15	***Conostegia monteleagreana***
34'	Flowers regularly arranged at the end of inflorescence branches and without evident bracts; stamens 16 or more in number	
35	Inflorescence a reflexed and pseudoaxillary panicle; flowers (4-) 5-merous	***Conostegia cinnamomea***
35'	Inflorescence a terminal panicle; flowers usually 6-merous	
36	Leaves with an entire margin and caudate apex	***Conostegia tenuifolia***
36'	Leaves with a serrulate to serrate margin and acuminate apex	
37	Leaves linear to linear elliptic, up to 3.5 cm wide, attenuate at the base; stamens 16–21	***Conostegia attenuata***
37'	Leaves ovate, 2–7.3 cm wide, obtuse to rounded at the base; stamens 26–36	***Conostegia polyandra***
33'	Stem apices and leaf abaxial surfaces evidently pubescent at least on the veins	
38	Leaf base strongly decurrent on the petiole; leaves sessile or nearly so	***Conostegia ortizae***
38'	Leaf base acute to rounded; leaves petiolate	
39	Leaves linear to linear elliptic, up to 3.5 cm wide	***Conostegia attenuata***
39'	Leaves elliptic to ovate, more than 5 cm wide	
40	Petioles setose adaxially; flower buds covered by foliaceous bracteoles	***Conostegia lasiopoda***
40'	Petioles glabrescent or variously pubescent, but not setose adaxially; flower buds not covered by foliaceous bracteoles	
41	Floral buds up to 4(-5.5) mm long; petals up to 5 mm long; style lacking a collar around the base	***Conostegia extinctoria***
41'	Floral buds more than (4.7-)5.5 mm long; petals 7 mm long or more; base of the style enveloped by a collar	
42	Indument on stems evidently stipitate stellate, not rusty colored; stamens more than 30	***Conostegia lancifolia***
42'	Indument on stems sometimes stipitate stellate but not evidently so, rusty colored; stamens up to 24	
43	Flower buds (7.5-) 10–18 mm long, the apex long apiculate	***Conostegia apiculata***
43'	Flower buds 4.7–11 mm long, the apex rounded to short apiculate	
44	Flower buds with the surface sparsely pubescent resulting in a visible surface, constricted below the torus; petioles 1–5 cm long	***Conostegia centronioides***
44'	Flower buds with the surface densely pubescent resulting in a hidden surface, not to slightly constricted below the torus; petioles 0.4–2.5 cm long	***Conostegia rubiginosa***
31'	Calyptra circumscissle and falling as a unit; style shorter or of equal length than the stamens (section *Conostegia* & section *Geniculatae*, pro minore parte)	
45	Stigma lobed and/or with an evident hole in the middle	
46	Flower buds pyriform to ellipsoid, with an attenuate to apiculate apex, longer than wide	
47	Stem apices and leaf abaxial surface slightly pubescent to evidently pubescent; plants from Mexico	
48	Stems and leaf abaxial surfaces furfuraceous	***Conostegia jaliscana***
48'	Stems and leaf abaxial surfaces densely beset with stellate and stalked stellate trichomes	***Conostegia arborea***
47'	Stem apices and leaves glabrous or with scattered stellate trichomes in *Conostegia chiriquensis*; plants from Costa Rica and Panama	
49	Flower buds 5–7 mm long; petals to 5 mm long, white with a red band at the base; anthers to 2.3 mm long	***Conostegia fragrantissima***
49'	Flower buds 7–14 mm long; petals at least 8 mm long, white to less commonly lavender; anthers at least 2.5 mm long	
50	Stigma peltate and with a hole in the middle	***Conostegia pitteri***
50'	Stigma barely expanded and lacking a hole in the middle	***Conostegia chiriquensis***
46'	Flower buds mostly ovoid to spherical with a rounded, mucronate, or broadly acute apex, as long as wide or slightly longer than wide	
51	Stigma capitate, but lacking a large hole in the middle; stigma lobes not conspicuous	***Conostegia icosandra***
51'	Stigma with large hole in the middle; stigma lobes conspicuous	
52	Leaves with two tubercles at the apex of the petiole abaxially; petals pink	***Conostegia bigibbosa***
52'	Leaves lacking tubercles at the apex of the petiole abaxially; petals white	
53'	Floral buds obovoid, constricted near the torus, acute at the apex, the surface smooth; leaves narrowly elliptic	***Conostegia bernoulliana***
53	Floral buds spherical, not constricted near the torus, abruptly mucronate apically, the surface inconspicuously to usually evidently tuberculate; leaves elliptic to usually ovate	
54	Bracteoles replaced by persistent clusters of setae; pedicels up to 3 mm long	***Conostegia setifera***
54'	Bracteoles not consisting of setae, usually early deciduous; pedicels usually more than 3 mm	
55	Style straight; petals apically retuse; leaves stellate pubsecent abaxially	***Conostegia macrantha***
55'	Style curved; petal apex rounded, truncate to emarginate; leaves stellate sometimes stellate pubescent abaxially when young, usually glabrous with age	
56	Leaf margin entire to denticulate; floral buds usually conspicuously tuberculate; Nicaragua to Panama	***Conostegia oerstediana***
56'	Leaf margin undulate dentate; floral buds inconspicuously tuberculate; Guatemala and Mexico	***Conostegia volcanalis***
45'	Stigma not lobed or with a hole in the middle	
57	Leaf bases with pouch like formicaria; indument of the stems with long simple hairs	***Conostegia setosa***
57'	Leaf bases lacking formicaria; indument of the stems various but not of long simple hairs	
58'	Flowers subtended by persistent foliaceous bracts	
59	Stems and leaves glabrous or with inconspicuous trichomes	***Conostegia monteleagreana***
59'	Stems and leaves densely pubescent with simple and somewhat branched trichomes	***Conostegia bracteata***
58'	Flowers not subtended by persistent foliaceous bracts	
60	Leaves with the abaxial surface covered with white to tan trichomes, making the actual surface hidden or almost so	
61	Abaxial leaf surface and hypanthium covered with lepidote trichomes; tree reaching about 25 meters tall; calyptra translucent, less pubescent than the hypanthium; seeds about 1.5 mm long; Endemic to the Osa Peninsula, Costa Rica	***Conostegia osaensis***
61'	Abaxial leaf surface and hypanthium covered with stellate trichomes; tree reaching about 15 meters tall; calyptra tan to whitish colored, not translucent, and as pubsecent as the hypanthium; seeds to about 1 mm long	
62	Calyptra with linear appendages at the apex about 2.5 mm long	***Conostegia plumosa***
62'	Calyptra lacking linear appendages at the apex, at most minute bumps present on the apex	***Conostegia xalapensis***
60'	Leaves with the abaxial surface glabrous or pubescent, if pubescent, never with the surface hidden	
63	Stem apices and leaf abaxial surfaces pubescent	
64	Leaf adaxial surface and floral buds densely hirsute; inflorescence and bud pubescence purple	***Conostegia speciosa***
64'	Leaf adaxial surface glabrous and floral buds glabrous or beset with stellate or dendritic trichomes; inflorescence and bud pubescence rusty or whitish	
65	Leaves 5–9 plinerved; flowers with small appendages on the calyptra apex; floral buds wider above the torus; petals apically slightly mucronate to acute, especially when seen at anthesis; filaments with an evident geniculation; seeds about 1 mm long	***Conostegia subcrustulata***
65'	Leaves 3–5 nerved or plinerved; flowers without small appendages on the calyptra apex; floral buds usually not wider above the torus; petals rounded, emarginate or truncate; filaments without an evident geniculation; seeds about 0.5 mm long	
66	Floral buds, veins on leaf abaxial surface and stem apices rusty with small brown dendritic hairs; petals 7 or more mm long; ovary apex elevated into a conspicuous collar around the style base	***Conostegia rufescens***
66'	Leaf abaxial surface and stem apices sparsely to densely covered with stellate or stipitiate stellate trichomes, floral buds glabrous to sparsely or densely covered with stellate or stipitiate stellate trichomes; petals 6.5 mm long or less; ovary apex usually not elevated into a conspicuous collar around the style base (somewhat evident in *Conostegia superba*)	
67	Stem apices, leaf abaxial surfaces and floral buds densely covered with sessile stellate and/or stipitate stellate trichomes	
68	Leaf and bud indument combining sessile stellate and stipitate stellate trichomes; floral buds with the lobes slightly differentiated at the apex; plants from Cuba and the Dominican Republic	***Conostegia lindenii***
68'	Leaf and bud indument of stipitate stellate trichomes; floral buds with the lobes not differentiated at the apex; plants from Central America	
69	Trees reaching 12 m high; Belize, Guatemala and Honduras	***Conostegia caelestis***
69'	Shrubs rarely reaching 4 m high; Costa Rica	***Conostegia brenesii***
67'	Stem apices and leaf abaxial surfaces sparsely to densely covered with stellate trichomes or sparsely beset with stipitate stellate trichomes, floral buds glabrous or sparsely beset with sessile or less commonly some stipitate stellate trichomes	
70	Leaves 7.1–36 × 2.3–16 cm; inflorescence 7–27.5 cm long, with flowers disposed in umbels terminating the inflorescence branches	***Conostegia superba***
70'	Leaves 3.8–21.5 × 1.2–10.5 cm; inflorescence 2.7–18.1 cm, long with flowers not disposed in umbels terminating the inflorescence branches	
71	Stem apices and abaxial leaf surfaces with sessile and stipitate stellate trichomes	***Conostegia hirtella***
71'	Stem apices and abaxial leaf surfaces with sessile stellate trichomes	
72	Leaves densely stellate pubescent on abaxial leaf surface; lowlands of the Caribbean slope of Nicaragua, Costa Rica and Panama	***Conostegia micrantha***
72'	Leaves with trichomes mostly restricted to the veins; widely distributed	***Conostegia montana***
63'	Stem apices and leaf abaxial surfaces glabrous or with very inconspicuous trichomes	
73	Inflorescence pendant; flowers with purple petals; seeds muriculate	***Conostegia muriculata***
73'	Inflorescence erect; flowers with white or pink petals; seeds smooth	
74	Flowers sessile or on pedicels up to about 1 mm long	***Conostegia montana***
74'	Flowers on pedicels 1.5 mm long or usually longer than 2 mm	
75	Flowers clustered at the end of the inflorescence branches; flower buds up to and usually less than 11 mm long; Southern Mexico (Oaxaca and Veracruz) through Central, and South America, only *Conostegia superba* reaching the Caribbean including Jamaica	
76	Petals 7–12 mm; anthers 4–4.5 mm long; stigma capitate; bracteoles up to 6 mm long; flowers 6–8 merous	***Conostegia cuatrecasii***
76'	Petals to 6 mm, less commonly to up to 8.3 mm; anthers to 3.25 mm long; stigma cylindrical to slightly expanded; bracteoles absent or to about 1 mm long; flowers (4-)5–7 merous	
77	Flower buds acute to slightly apiculate at the apex, not contricted in the middle, usually white; inflorescence rachis usually white	***Conostegia superba***
77'	Flower buds apiculate at the apex, constricted in the middle, usually pink; inflorescence rachis pink	***Conostegia rhodopetala***
75'	Flowers not clustered at the end of the inflorescence branches; flower buds 10 mm long or usually longer; Southwest Mexico (Guerrero and Jalisco) or Jamaica	
78	Leaf margin serrulate and cilate; pedicels 2–5 mm long; plants from Southwest Mexico (Guerrero and Jalisco)	***Conostegia jaliscana***
78'	Leaf margin not serrulate and cilate; pedicels 5–13 mm long; plants from Jamaica	
79	Hypanthium ribbed, at least towards the base; abaxial leaf surfaces with tuft domatia at the base	***Conostegia procera***
79'	Hypanthium not ribbed at the base; abaxial leaf surface lacking tuft domatia at the base	
80	Petals pink	***Conostegia balbisiana***
80'	Petals white	***Conostegia pyxidata***

### Conostegia sect. Conostegia

Taxon classificationPlantaeMyrtalesMelastomataceae

#### Diagnosis.

A mostly Central American and Caribbean group, section *Conostegia* is distinguished by the following combination of characters: calyx calyptrate with the calyptra falling as a unit, lacking calyx teeth altogether, and with conspicuous sclereids internally. Flowers generally pleiostemonous, staminal filaments not evidently geniculate and transitioning smoothly to the anther thecae, style mostly the same length or shorter than the stamens, with a stele within, mucilage inside the ovary, seeds ovoid and smooth.

### Conostegia arborea

Taxon classificationPlantaeMyrtalesMelastomataceae

(Schltdl.) Steud.

Conostegia arborea (Schltdl.) Steud., Nomencl. ed. II. 1: 405. 1841. Melastoma arboreum Schltdl., Linnaea 13: 424. 1839. Type: Mexico. “Inter Tioselo et Jicochimalco”, August 1829, Schiede s.n. (lectotype: GOET!, designated here; isolectotypes: BM!, K!, LE).
Conostegia galeottii Naudin, Ann. Sci. Nat. Bot. ser. 3 16: 107. 1850. Type: Mexico. Veracruz: June-October 1940, H. Galeotti 2917 (holotype: P, isotypes: BR!, K!, NY!).

#### Description.

Trees 2–8 m tall with tetragonal, ridged and swollen stems which are covered with a mixture of sessile stellate and stalked-stellate hairs sometimes intermixed with simple hairs; the nodal line present but mostly obscured by indument. Leaves of a pair equal to somewhat unequal in length. Petiole 1–7 cm. Leaf blades 8–26.9 (-30) × 6.6–11.5 (-15) cm, 3–5 plinerved with the innermost pair of veins diverging from the mid vein in sub alternate to alternate fashion up to 1.5 cm above the base, elliptic, the base acute to obtuse, the apex acute to acuminate, the margins dentate with gentle curves between the well separated teeth, adaxially glabrous, abaxially with a a mixture of sessile stellate and stalked-stellate hairs sometimes intermixed with simple hairs. Inflorescence a terminal panicle 6–15 cm long branched above the base but sometimes appearing branched at the base because of multiple inflorescences arising at opposing meristems at the terminal node, accessory branches present, rachis flattened, bracts early deciduous or absent, bracteoles linear, ca. 2 mm long, early deciduous. Pedicel 3.5–6 mm long. Flowers 7–8(-12) merous, calyptrate. Flower buds 11–16 × 5–9 mm, not constricted in the middle, the base flat, the calyptra apiculate; the hypanthium 6–9 × 6–9 mm, smooth, glabrescent to evidently stellate pubescent. Petals 10–12.5 × 7–8 mm, white, obovate, glabrous, rounded-truncate to emarginate. Stamens 20–28, apparently slightly zygomorphic because the style is bent, the filament 4–5 mm, not evidently geniculate, white, anthers ca. 3 mm, oblong and somewhat recurved, sagittate at the base, the connective thickened, laterally compressed, yellow except for a hugh of rose at the base of thecae dorsally in one specimen, the pore ca 0.15 mm wide. Ovary 10–14 locular, inferior, glabrous, the apex elevated into a collar around the style. Style 5–6 mm, strongly bending downwards, vertical distance from the anther to the stigma ca. -1 mm, horizontal distance ca. 1-1.5 mm; stigma consisting of 10–15 laterally compressed lobes but not evidently crateriform, 2–3 mm wide. Berry 8–9 × 8–9 mm, purple. Seeds ca. 0.6 mm, pyramidal, smooth.

#### Distribution

**Figure 63.**
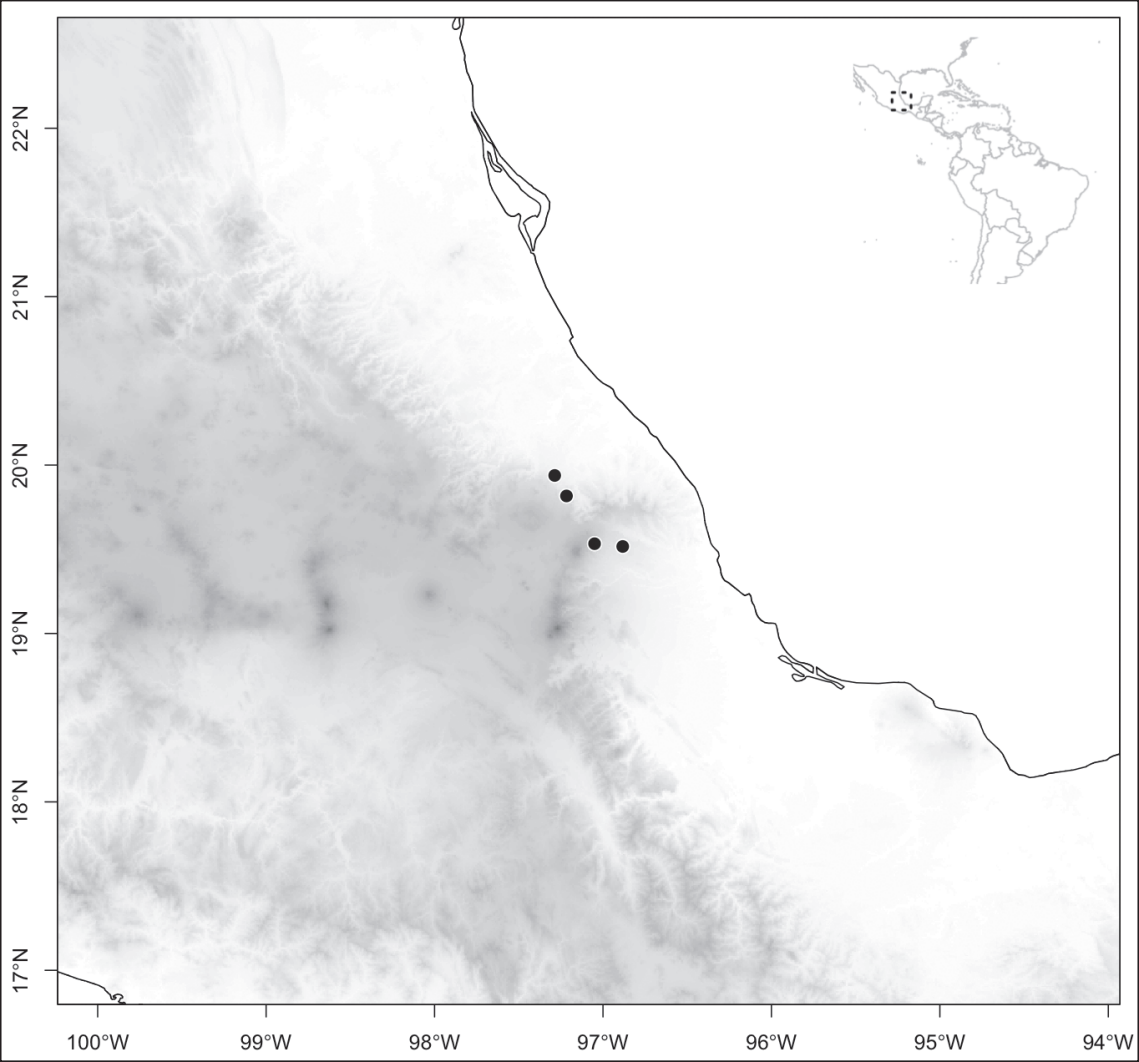
Distribution of *Conostegia arborea*.

(Fig. 63). Puebla and Veracruz, Mexico, 800–1600 m.

*Conostegia arborea* can be recognized by its leaves with dentate margins, abaxial indument of sessile and stipitate stellate hairs and especially by its apiculate calyptra apices. The amount of indument on floral buds is variable. The flowers of *Conostegia arborea* have been reported to have a good fragrance (*Ventura 1141*-NY).

#### Specimens examined.

**MEXICO. Puebla**: Texcaco, Gold 7 (NY); El Reparo, municipio de Hueytamalco, Ventura 415 (IEB, NY). **Veracruz**: 1 km al norte de Rancho Viejo sobre el río Pixquiac, Marquez et al. 849 (NY); along very winding road from Naolinco to Misantla 13 km by road S of turn off to Yecuatla and 6 km by road N of Paz de Enríquez, Mun. Yecuatla, Nee et al. 26343 (NY); near Jalapa, Pringle 8170 (NY); Tatzayanala, Municipio de Atzalan, Ventura 1141 (NY).

### Conostegia balbisiana

Taxon classificationPlantaeMyrtalesMelastomataceae

Ser. ex DC.

Conostegia balbisiana Ser. ex DC., Prodr. 3: 174. 1828. Type: Jamaica. 1822, C. Bertero s.n. (holotype G!).
Conostegia grisebachii Cogn., DC. Monog. Phan. 7: 700. 1891. Type: Jamaica. 1857, W. Marsh 598 (holotype: GOET!; isotype: BR-fide Schnell (1996), K!, TCD!).

#### Description.

Shrubs and trees 2–12 m tall with thick strongly tetragonal glabrous stems; the nodal line present. Leaves of a pair equal to somewhat unequal in length. Petiole 1–6.6 cm. Leaf blades 5–18 × 2.9–9 cm, 3–5 nerved or if 3–5 plinerved, with the innermost pair of primary veins arising up to about 1 cm above the base, ovate, rounded at the base, acute to rounded and short acuminate at the apex, the margin entire, glabrous. Inflorescence a terminal panicle 8.5–19 cm branched well above the base but sometimes appearing branched at the base because of multiple inflorescences arising at opposing meristems at the terminal node, accessory branches apparently absent, bracts early deciduous, bracteoles 1–2 mm long, subulate, deciduous or persistent. Pedicels 7–10 mm. Flowers (5-)6(-7) merous, calyptrate. Flower buds 11–17 × 5.5–7 mm, elliptic pyriform, the base rounded, the apex acuminate and sometimes mucronate, constricted in the middle, hypanthium 4–4.5 × 5.5–7 mm, glabrous. Petals 12–20 × 7–11.5 mm, pink, obtriangular, emarginate, glabrous. Stamens 12–14, 7–9.5 mm long, androecium slightly zygomorphic, the filament 3.5–4.5 mm, not geniculate, apparently white to pink, anthers 2.85–4 × 1–1.5 mm, narrowly elliptic, sagittate at the base, laterally compressed, yellow, thickened dorsally and with a small hump in dried material, the terminal pore ca. 0.1 mm wide. Ovary 6–9 locular, inferior, glabrous, forming a collar around the style base; the style 6–8 mm, bent below the tip, vertical distance from the anthers to the stigma ca. -0.6 – -0.2 mm, horizontal distance apparently very reduced to absent, stigma truncate, 0.5–0.75 mm wide. Berry ca. 6–7 × 8–9 mm, blue-black. Seeds ca. 0.8 mm, obliquely pyramidal, smooth.

#### Distribution

**Figure 64.**
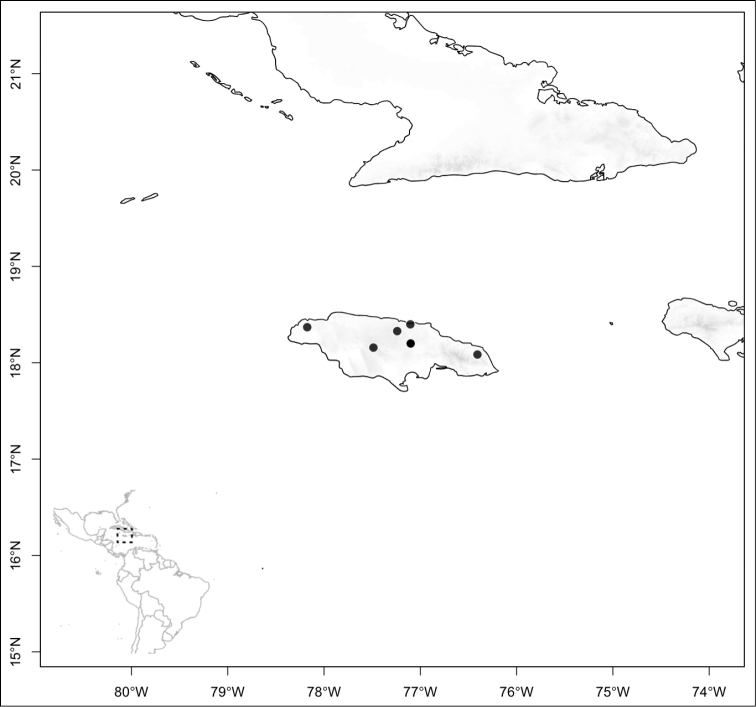
Distribution of *Conostegia balbisiana*.

(Fig. 64). Endemic to Jamaica, 350–950 m elevation.

*Conostegia balbisiana* can be recognized by its leaves which usually have tufted mite domatia on the leaf base abaxially, large pink flowers and linear anthers. Schnell (1996) discussed variation observed in the three Jamaican endemics and struggled with their recognition. Perhaps population genetic studies might help untangle how many species should be recognized within this Jamaican lineage. For the time being, the three species recognized by Schnell (1996), who studied the most specimens of these taxa, are here recognized. The short style characteristic of section *Conostegia* is evident in specimens *Proctor 10432* and *10285* both at NY.

#### Specimens examined.

**JAMAICA. Hanover**: East Slope of Dolphin Head, Proctor 10432 (NY). **Manchester**: Holmwood P.T.C., near Christiana, Proctor 10285 (NY). **Portland**: Woodlands eastern slopes of south end of John Crow Mountains, Harris and Britton 10730 (NY). **St. Andrew**: Near Diamond (Redhills District), Robertson and Wynter s.n. (NY). **St. Ann**: Soho, Harris 12024 (NY). **St. Catherine**: Holly Mount, Mt. Diablo, Harris 8984 (NY); west of Hollymount, Mt. Diablo, Hespenheide 1301 (NY). Ocho Rios and vicinity, Britton and Hollick 2690 (NY). Mandeville, Maxon 2590 (NY).

### Conostegia bernoulliana

Taxon classificationPlantaeMyrtalesMelastomataceae

Cogn.

Fig. 65

Conostegia bernoulliana Cogn., DC. Monog. Phan. 7: 698. 1891. Type: Guatemala. Sarnayara: April 1877, K. Bernoulli & O. Cairo 2884 (lectotype: GOET! (image seen), designated here; isolectotype: K!).
Conostegia sphaerica Triana, Trans. Linn. Soc. London 28: 98. 1872. Type: Mexico. Teotalcingo (probably in Oaxaca): June 1842, Liebmann 2842 (holotype: P, isotypes: BM!, BR, C, F!, K!). Also numbered as Liebmann 18 and Liebmann s.n.

#### Description.

Tree 6–16 m tall with grayish-brown bark peeling in large thick flakes, the somewhat tetragonal and ridged stems in newer branches glabrous or with inconspicuous underdeveloped stellate or dendritic trichomes; the nodal line evident mostly on young nodes. Leaves of a pair equal to somewhat unequal in length. Petioles 0.8–4 cm long. Leaves 5.5–18 × 2–6 cm, 3–5 plinerved, with the innermost diverging from the mid vein 0.5–1.5 cm above the base in opposite or sub opposite fashion, the outermost primary veins usually inconspicuous and resulting in a mostly 3 veined looking leaf, narrowly elliptic, acute at the base, the apex acuminate, the margin entire, glabrous on both surfaces. Inflorescence a terminal panicle 5–9 cm long branching above the base, accessory branches present, bracts to 4 cm, early deciduous absent on most specimens, bracteoles ca. 0.5 mm long, linear, early deciduous and appearing absent on most specimens. Pedicel 3.5–4.5 mm long. Flowers 6–9 merous, calyptrate; flower buds 7.5–9.3 × 5–7 mm, obovoid, rounded at the base, obtuse to rounded and apiculate at the apex, slightly constricted below the calyptra, the hypanthial and calycine portions not or only slightly differentiated, constricted above the torus, hypanthium 4.5–5 × 4.5–5 mm, smooth. Petals 6.75–8 × 6.5–7 mm, white, narrowly obtriangular, spreading, emarginate, glabrous. Stamens 18–25, 7.75–9.25 mm long, radially arranged, the filament 4.75–5.25 mm, lacking a geniculation, white, anthers 3–3.5 × 1.25–1.75 mm, ovoid, laterally compressed, yellow, the base sagittate, the connective thickened dorsally and with a small bump, the pore 0.15 mm wide, terminal or subterminal. Ovary 8–12 locular, inferior, apically glabrous and forming a stylar collar. Style 6–6.5 mm, strongly bending downwards resulting in a evidently zygomorphic flower, vertical distance from the anther to the stigma ca. -1 mm, horizontal distance ca. 1–1.5 mm; stigma crateriform, consisting of 8–12 laterally compressed lobes, 3–3.3 mm wide. Berry 6–8 × 6–8 mm, when dry; seeds not seen.

**Figure 65.**
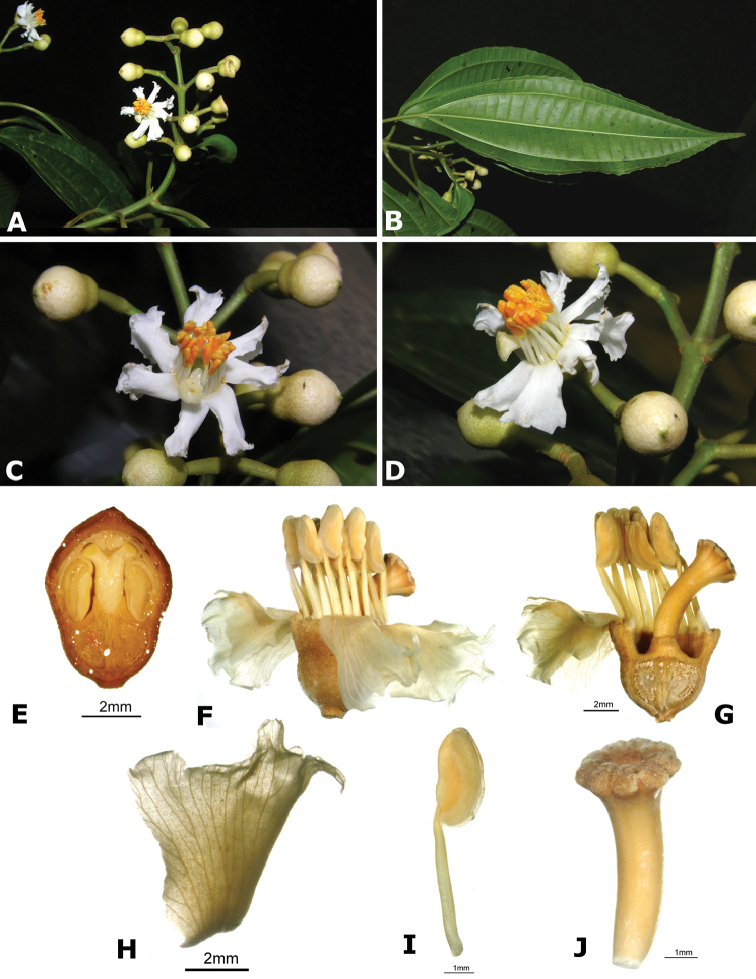
*Conostegia bernoulliana*. **A** Habit and inflorescence **B** Leaf abaxial surface **C** Frontal view of flower **D** Lateral view of the flower **E** Longitudinal section of flower bud **F** Lateral view of the flower from pickled material **G** Dissection of lateral view of the flower from pickled material **H** Petal **I** Stamen **J** Style. Photos of specimen vouchered *R. Kriebel 5578*.

#### Distribution

**Figure 66.**
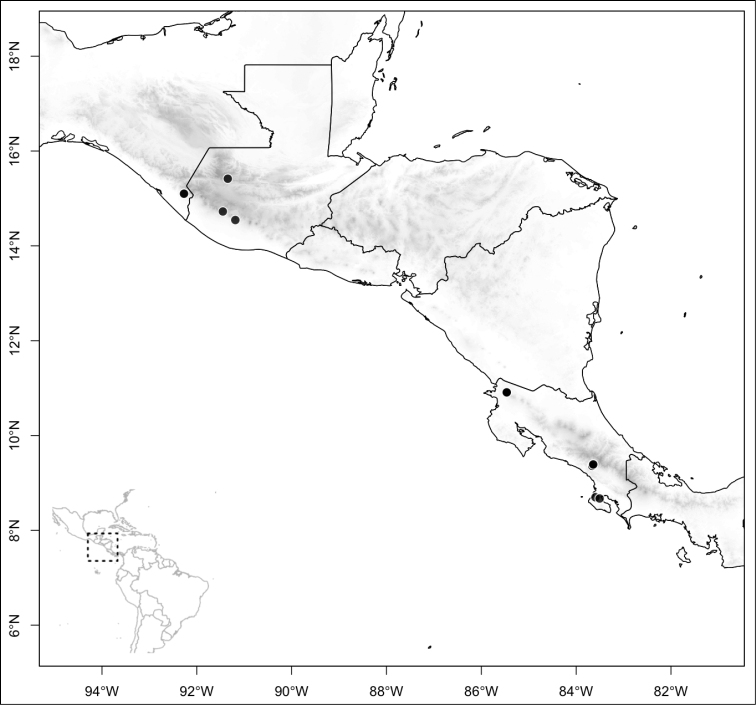
Distribution of *Conostegia bernoulliana*.

(Fig. 66). Mexico, Guatemala and on the pacific slope of the Costa Rica cordilleras, reaching the lowlands of the Osa Península, 50–1500 m in elevation.

*Conostegia bernoulliana* was synonymized under *Conostegia icosandra* by Schnell (1996) and Almeda (2009). I have chosen to recognize *Conostegia bernoulliana* after studying populations in the field of *Conostegia icosandra* that correspond with the type and one that corresponds to *Conostegia bernoulliana* in addition to many herbarium specimens of *Conostegia icosandra*. The main differences between the two species are the presence of indument in the stems and leaves, the broader 3–5 plinerved leaves, the persistent ovate bracteoles and non crateriform stigma of *Conostegia icosandra*. In contrast *Conostegia bernoulliana* has glabrous stems and leaves, the latter narrow and usually three plinerved, bracteoles very early deciduous to apparently lacking, and an evidently crateriform stigma. Schnell (1996) noted that the other syntype, *Wendland 545* (GOET), pertains to *Conostegia oerstediana* Berg ex Triana.

#### Specimens examined.

**MEXICO. Chiapas**: Finca Mexiquito, Purpus 6785 (MO, NY).

**GUATEMALA. Huehuetenango**: vicinity of Maxbal about 17 miles north of Barillas, Sierra de los Cuchumatanes, between Maxbal and lake to the southeast, Steyermark 48726 (NY). **Sololá**: south-facing slopes of Volcán Atitlán above Finca Mocá, Steyermark 47935 (NY). **Suchitepéquez**: Finca Mocá, Hunnewell 14756 (NY); Finca Moca, Skutch 2068 (NY); southern lower slopes of Volcán Zunil, vicinity of Finca Las Nubes, along Quebrada Chita, east of Pueblo Nuevo, Steyermark 35407 (NY).

**COSTA RICA. Guanacaste**: Liberia, P.N. Guanacaste, cuenca del Tempisque, Sector Cacao, Acosta et al. 1166 (CR, INB, MO, NY). **Puntarenas**: Distrito Sierpe, Reserva Forestal Golfo Dulce, Rincón, cerca de Banegas, Los Charcos, 1 km. al Este del centro del pueblo Banegas Estación Biológica Los Charcos de Osa, Aguilar 10855 (NY); camino a Rancho Quemado, Kriebel et al. 5540 (INB). **San José**: Río San Isidro, Jiménez 3840 (NY); Perez Zeledón, Vicinity of El General, Skutch 2604, 4159 (MO, NY).

### Conostegia bigibbosa

Taxon classificationPlantaeMyrtalesMelastomataceae

Cogn.

Fig. 67

Conostegia bigibbosa Cogn., Bull. Soc. Roy. Bot. Belg. 30: 252. 1892. Type: Costa Rica. San José: Dans la forest a General, 800 m, February 1891, A. Tonduz 3793 (holotype BR!; isotypes BR! (2), US!). Cited as Pittier 3793 in the original description.

#### Description.

Trees 3–15 m tall with tetragonal and ridged, glabrous to sparsely furfuraceous-puberulent stems; the nodal line conspicuous and frequently elevated, with conspicuous lenticels abaxially. Leaves of a pair equal to somewhat unequal in length. Petioles 2.7–6.5 cm, with paired projections on the abaxial surfaces at the petiole/laminar junction. Leaf blades 17–29 × 9.2–20 cm, 5–7 nerved, elliptic to elliptic ovate, obtuse to broadly rounded, abruptly acuminate, the margins undulate-denticulate, adaxially glabrous,abaxially glabrous or sparsely furfuraceous-puberulent. Inflorescence a terminal panicle 13–22 cm long branching well above the base, with accessory branches, bracts not seen, bracteoles subulate, 1–2 mm long, deciduous. Pedicel 4–10 mm long. Flowers 8 merous, calyptrate. Flower buds 7–9.2 × 6.4–9.6 mm, white, globose, rounded to flattened at the base, rounded to slightly flattened at the apex, the hypanthial and calycine portions undifferentiated, the hypanthium 5–6 × 8–9 mm, glabrescent and tuberculate. Petals 7–11.25 × 7–11 mm, pink, broadly obovate, rotate at anthesis, rounded-emarginate, glabrous. Stamens 22–26, 9–10 mm, radially arranged around the style but frequently secondarily zygomorphic because the stamens that are below the side that the style bends get stuck below the downward bent style and giant stigma, the filament 5–5.5 mm long, not geniculate, white, anthers 4–4.5 × 1.5–1.6 mm, elliptic, laterally compressed, the base sagittate, yellow, the pore 0.2–0.35 mm, terminal to slightly dorsally inclined. Ovary 15–17 locular, inferior, fluted into a glabrous dome. Style 7–8.5 mm long, bending downwards, vertical distance from the anther to the stigma ca.-0.5 – -0.25 mm, horizontal distance ca. 1 mm; stigma crateriform and lobed, 5.5–6 mm wide. Berry and seeds not seen.

**Figure 67.**
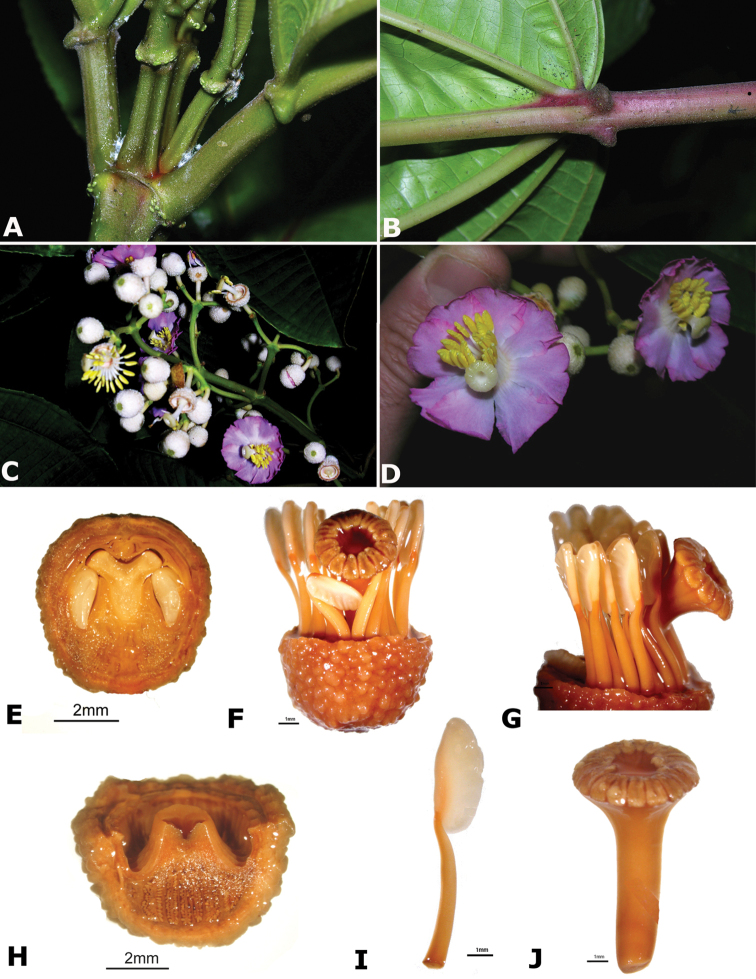
*Conostegia bigibbosa*. **A** Lenticellate internode **B** Tubercles on the base of the abaxial leaf surface **C** Inflorescence **D** Flowers at anthesis **E** Longitudinal section of a flower bud **F** Ventral view of a pickled flower with the petals removed and showing a stamen stuck under the stigma **G** Lateral view of a pickled flower with the petals removed **H** Longitudinal section of a hypanthium **I** Stamen **J** Style. Photos of specimen vouchered *R. Kriebel 5522*.

#### Distribution

**Figure 68.**
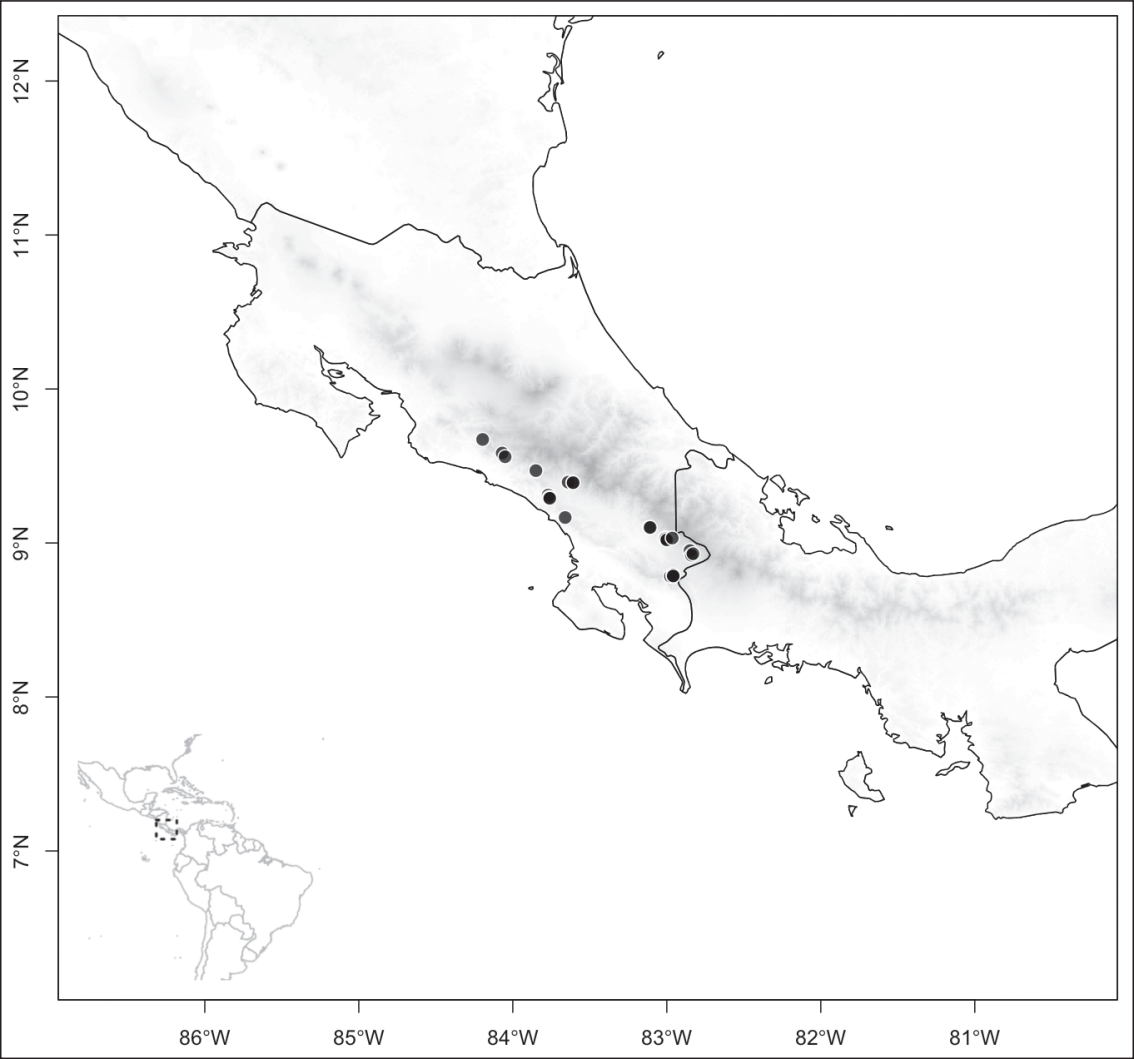
Distribution of *Conostegia bigibbosa*.

(Fig. 68). Endemic to the pacific side of the Cordillera de Talamanca in Costa Rica, 800–1800 m elevation.

*Conostegia bigibbosa* was synonimized under *Conostegia oerstediana* by Schnell (1996) and subsequently recognized by Almeda (2009). I here recognize it based on geographic distribution and morphological characters. *Conostegia bigibbosa* always has pink petals as well as two tubercles on the petiole apex which makes it unmistakable. At one point, Schnell annotated it as a subspecies of *Conostegia oerstediana* which is allopatric and has white petals and lacks the two tubercles at the apex of the petiole. Schnell (1996) carried out some crossing experiments between *Conostegia bigibbosa* and *Conostegia oerstediana* and *Conostegia montana*. He noted that fruit development was initiated when pollinating *Conostegia oerstediana* with pollen of *Conostegia bigibbosa* but not with pollen of *Conostegia montana* or when left unpollinated. Unfortunately he could not finish his experiment because the material was vandalized. The two species are not sympatric however and maintain the petal color difference as well as the petiole tubercle difference in natural populations.

#### Specimens examined.

**COSTA RICA. Puntarenas**: Finca Las Alturas NW of lechería, Almeda et al. 6697 (CAS, CR, NY); E.B. Las Cruces Trailhead to río Java, Boyle et al. 6265 (CR, NY). **San José**: Cantón de Pérez Zeledón, Fila Costeña, Fila Tinamastes, en la Fila del Farallón, Hammel et al. 20145 (INB, CR, NY); Aserrí, Quebrada Lajas, ca. 2.5 km al noroeste de Altos el Aguacate, Kriebel et al. 3975 (INB); Pérez Zeledon, Bajo Bonitas, Kriebel et al. 5522 (INB); Vicinity of El General, Skutch 2623 (NY).

### Conostegia bracteata

Taxon classificationPlantaeMyrtalesMelastomataceae

Triana

Fig. 69

Conostegia bracteata Triana, Jour. Bot. 4: 209. 1867. Type: Nicaragua. Chontales: 12 September 1867, B. Seemann 36 (holotype: K!; isotypes: BM!, EM, LE; BR).

#### Description.

Shrub to small tree 1.75–4.6 m tall with terete or nearly terete stems that are moderately to densely covered with an indument of simple or little branched hairs; the nodal line present but usually covered by indument. Leaves at a node equal to unequal in length. Petiole 0.4–2.1 cm. Leaf blades 5.5–18.5 × 2–7.2 cm, 3–5 nerved or slightly plinerved, narrowly elliptic to oblanceolate or narrowly obovate, the base attenuate to obtuse, the apex acuminate, margin entire to dentate, adaxially setose with spreading smooth hairs 1–2 mm long, abaxially moderately setose on the actual surface below with a mixture of smooth, barbed and stellulate hairs on the elevated primaries. Inflorescence a terminal panicle 3.5–9 cm long branched above the base, accessory branches present, the inflorescence rachis moderately to densely covered with an indument of simple or little branched hairs, elliptic to oblong, bracteoles 6–8 × 2–2.5 mm, persistent. Flowers sessile, (5-) 6 (-7) merous, calyptrate, buds 6–8 mm long, pyriform to ovoid or broadly ellipsoid, rounded at the base, short apiculate at the apex, the calyptra and hypanthium little differentiated, the hypanthium 3.5–3.75 × 3.5–4 mm moderately to densely covered with an indument of simple or little branched hairs. Petals 6–7 × 4–5 mm, white or pink, obtriangular, spreading, emarginate apically, glabrous on both surfaces, spreading. Stamens (12-)14–18, 5–6.5 mm, slightly zygomorphic, the filament 2.5–2.75 mm, white, anthers 2.5–2.75 × 0.5–0.75 mm, linear and somewhat laterally compressed, yellow, the base sagittate, the pore 0.1–0.16 mm, subterminal to ventral. Ovary 6–7 locular, inferior, fluted into a glabrous collar around the style. Style 3.5–4.25 mm, slightly curving upward, vertical distance between the anther and stigma ca. -1 mm, horizontal distance absent; stigma capitate, 1–1.5 mm wide. Berry 6–8 × 6–8 mm, dark purple-black. Seeds 0.4–0.6 mm, pyramidal, smooth.

**Figure 69.**
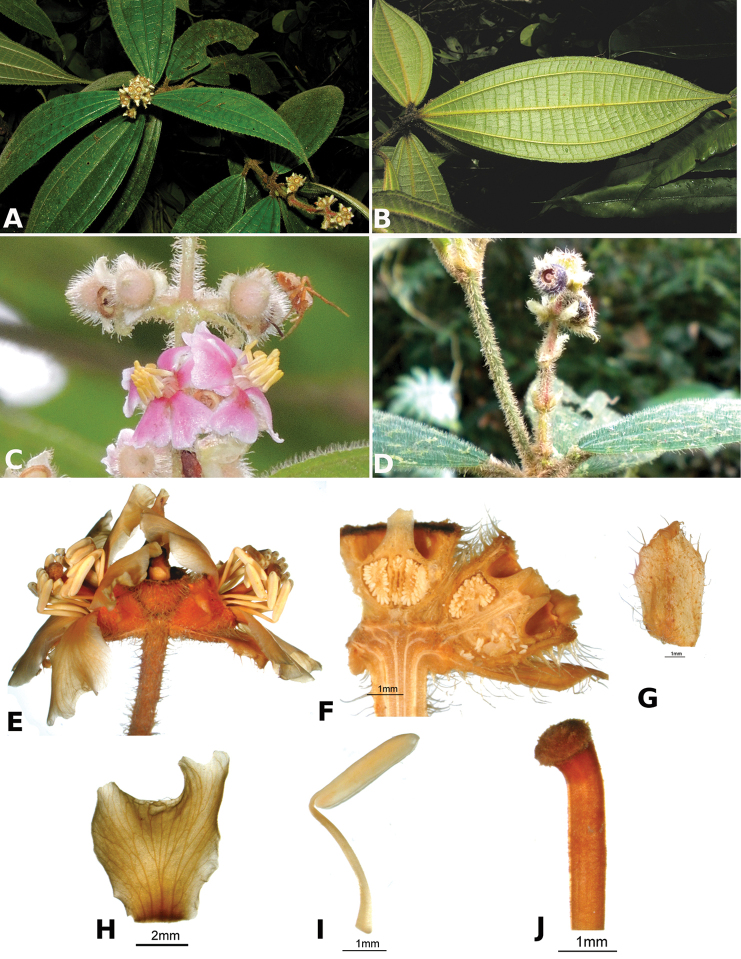
*Conostegia bracteata*. **A** Habit **B** Leaf abaxial surface **C** Flowers at anthesis **D** Infuctescence **E** Pickled flowers at anthesis **F** Lomgitudinal section of hypanthia of flowers at anthesis **G** Bract **H** Petal **I** Stamen **J** Style. Photos of specimen vouchered *R. Kriebel 5806*.

#### Distribution

**Figure 70.**
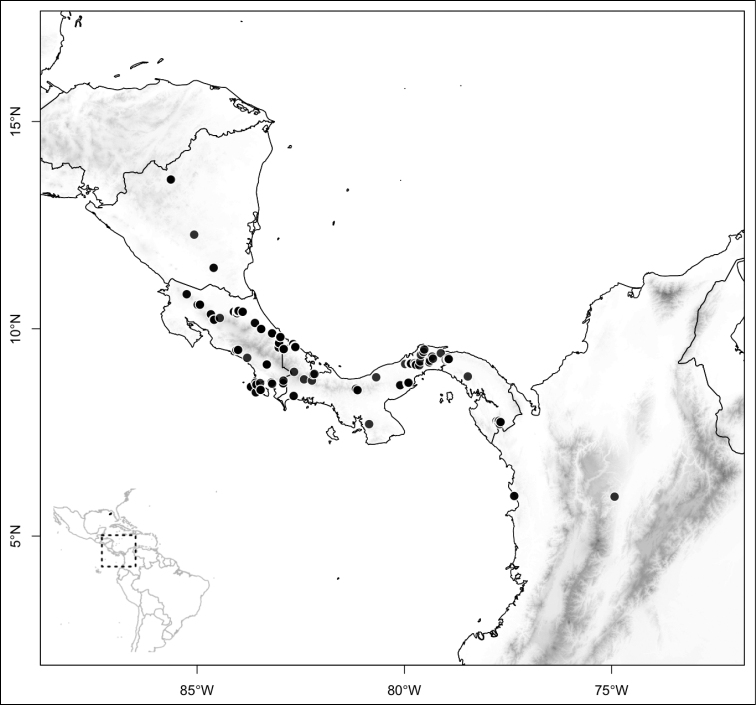
Distribution of *Conostegia bracteata*.

(Fig. 70). Nicaragua to Colombia, 0–1150 m in elevation.

*Conostegia bracteata* is one of the most distinctive species in the genus based on the presence of a hirsute indument on most parts of the plant including adaxial leaf surface, and large, persistent bracts subtending the sessile flowers. Flowers have been observed to be buzzed by several types of bees including Euglossines (Fig. 4). Specimens of this species dry red when treated with ethanol. Schnell (1996) argued that *Conostegia bracteata* provided an excellent example of convergent evolution between this species and *Miconia barbinervis* and *Miconia cuneata* in their “pubescence, foliage, and the general aspect of flowers, bracts and inflorescences”. These species look a lot alike in their leaf shape and pubescence but not in their flowers. *Conostegia bracteata* occurs sympatrically with *Miconia barbinervis* in some localities like at La Selva Biological Statation. They can easily be distinguished on the basis of the stipitate stellate trichomes present in *Miconia barbinervis* and simple trichomes in *Conostegia bracteata*.

#### Specimens examined.

**NICARAGUA. Chontales**: Jinotega (fide Schnell): Cerro San Pedro, Comarca Kilambe, Sandino 183 (MO).

**COSTA RICA. Limón**: R.B. Hitoy Cerere, siguiendo el sendero el Espavel hasta la cima de un cerro innominado, González et al. 3309 (INB, NY). **Heredia**: La Selva, OTS field station, La Sura trail at 1070 m, Hopkins 22 (NY); Sarapiquí, sendero Tres ríos y lindero el Peje, Kriebel et al. 3606 (INB, NY). **Puntarenas**: Osa, Sierpe, Reserva Forestal Golfo Dulce, Rancho Quemado, camino a Chiqueron en la parte mas alta de la fila al Sur de Rancho Quemado, Aguilar 6704 (NY); Golfito, P. N. Corcovado, Estación Agujas, Cerro Rincón, Mora et al. 721 (INB, MO, NY).

**PANAMA. Bocas del Toro**: Duwebdulup Peak No. of Río Teribe across from Quebrada Huron, Kirkbride and Duke 585 (MO, NY); Above Chiriqui Grande 10 road-miles from continental divide and c. 2 miles along road to east, McPherson 12837 (MO, NY). **Canal Zone**: Zetek trail 550, Croat 6628 (MO, NY); Madden Forest, Las Cruces Trail, Gentry 1380 (NY). **Darién**: 0–2 mi. E. of Tres Bocas along shortest headwater of Río Coasi, Kirkbride and Duke 1194 (MO, NY); Coasi-Cana Trail on Cerro Campamiento E. of Tres Bocas headwater of Río Coasi, Kirkbride and Duke 1252 (MO, NY); Vicinity of Cana, Stern et al. 514 (NY). **Panamá**: 2.5 m N of Goofy Lake on road to Cerro Azul, Croat 11533 (MO, NY); Along highway near top of Cerro Campana, Croat 12089 (MO, NY); 10 miles from highway on road to Cerro Jefe, Croat 15186 (MO, NY); Cerro Azul, Dwyer 2083 (MO, NY); Trail into forest 4.8 miles north of highway just west of El Llano, Gentry 5077 (MO, NY); Between Frijoles and Monte Lirio, Killip 12158 (NY); Parque Nacional Chagres, Sendero hacia Cerro Jefe que sale de la urbanización Altos de Cerro Azul, Kriebel and Burke 5683 (PMA, NY).

**COLOMBIA. Antioquia**: vicinity of Planta Providencia, 26 km S & 23 km W of Zaragoza, in valley of Río Anorí between Anorí and Dos Bocas, Denslow 2312 (US-not seen).

### Conostegia brenesii

Taxon classificationPlantaeMyrtalesMelastomataceae

Standl.

Fig. 71

Conostegia brenesii Standl., Field Mus. Publ. Bot. 18: 801. 1938. Type: Costa Rica. Alajuela: La Palma de San Ramón, 1275–1300 m, 7 August 1927, A. Brenes 5577 (holotype: F!, isotypes: CR!, NY!). Note: at least one sheet bearing this number (also at NY) is of Conostegia montana (Sw) Don ex DC. Schnell (1996).

#### Description.

Shrubs to small trees 1.5–4 m tall with tetragonal young stems that soon become terete and which are densely covered with stipitate-stellate trichomes; the nodal line inconspicuous to absent. Leaves of a pair equal to somewhat unequal in length. Petioles 0.6–4.9 cm. Leaf blades 5–13.2 × 2.5–6.5 cm, 5 nerved or more commonly slightly plinerved, with the innermost pair of veins arising up to 1.5 cm above the base in opposite to sub opposite fashion, elliptic to elliptic-ovate, the base acute or obtuse, the apex acute and acuminate, the margin denticulate or entire, densely hirsute with rigid hairs on both surfaces. Inflorescence a terminal panicle 4.8–9.6 cm long branched above the base but sometimes appearing branched at the base because of multiple inflorescences arising at opposing meristems at the terminal node, accessory branches absent, the rachis greenish-purple to purple, densely covered with stipitate stellate trichomes, the bracts absent or very early deciduous, bracteoles 1–4 mm, deciduous. Flowers 5–6 merous, calyptrate, flower buds 5.2–7.51 × 2.4–4 mm, rounded at the base, acute apically, the calycine and hypanthium portions weekly differentiated, slightly constricted at the torus; hypanthium 3–3.5 × 2.75–3.25 mm, covered with stipitate-stellate trichomes. Petals 5–6.5 × 6–6.25 mm, pink, light violet or white, obovate, spreading, rounded and emarginate, glabrous. Stamens 11–15, 6.25–7.25 mm, slightly zygomorphic, forming a 45 degree angle, the filament 3.75–4.25 mm, not evidently geniculate, white, anthers 2.5–3 × 0.5–1 mm, linear-oblong and somewhat recurved, laterally compressed, the base sagittate, yellow, the pore 0.1–0.2 mm, terminal. Ovary 6 (-7)-locular, inferior, glabrous and elevated into a collar around the base of the style. Style 5.5–6.5 mm, bending below the stigma, distance between the anther and the stigma -0.1 – -0.3 mm, horizontal distance absent; stigma subcapitate, 1.4–1.6 mm wide. Almost mature berry 5–6 × 5–6, probably purple at maturity like its close relatives. Seeds 0.4–0.6 mm, pyramidal, smooth.

**Figure 71.**
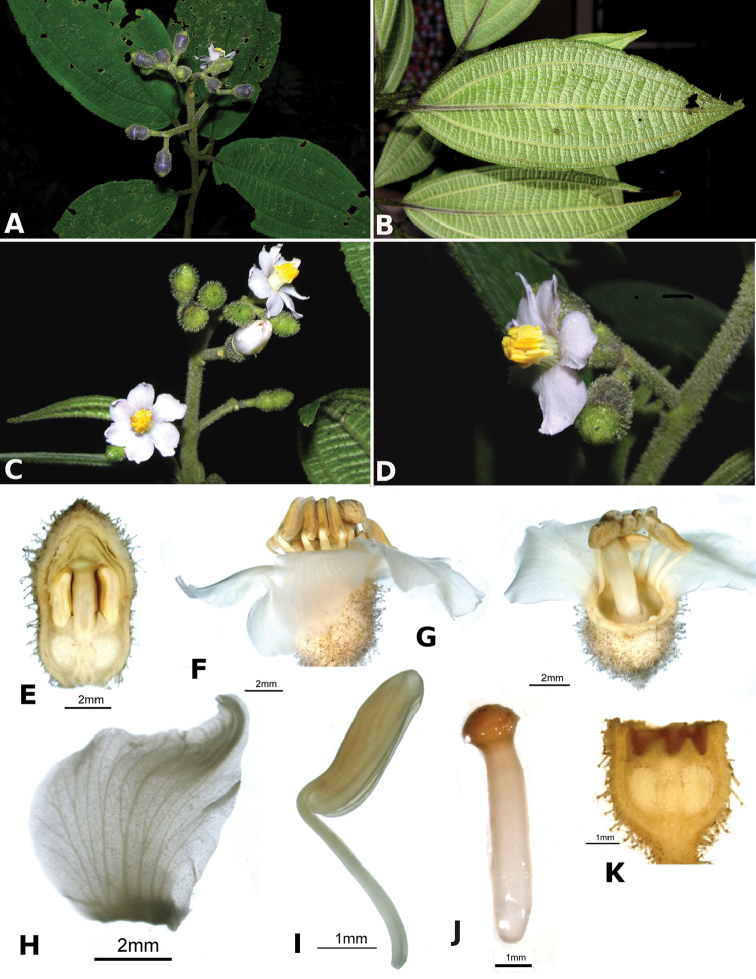
*Conostegia brenesii*. **A** Branch with inflorescence **B** Leaf abaxial surface **C** Inflorescence **D** Flower at anthesis **E** Longitudinal section of flower bud **F** Pickled flower at anthesis **G** Pickled flower at anthesis with half of the petals and stamens removed **H** Petal **I** Stamen **J** Style **K** Longitudinal section of the hypanthium. Photos of specimen vouchered *R. Kriebel 5631*.

#### Distribution

**Figure 72.**
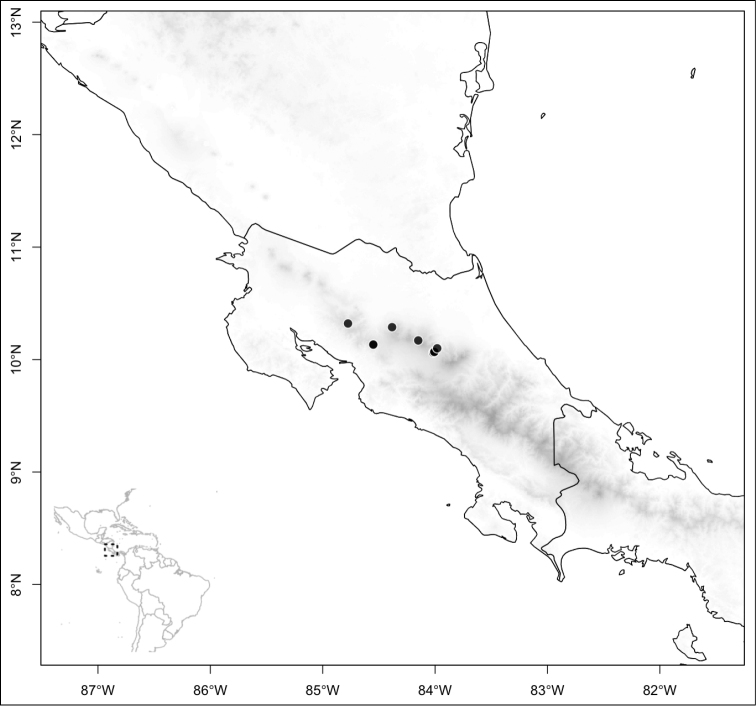
Distribution of *Conostegia brenesii*.

(Fig. 72). Endemic to cloud forests on the Caribbean slope of the Central and Tilaran cordilleras in Costa Rica, 1100–1750 m in elevation.

*Conostegia brenesii* is a very distinctive and narrow endemic of middle elevation cloud forests in Costa Rica. It can be easily distinguished by its dense indument of stipitate stellate hairs on all plant parts. Because of its dense indument of stipitate stellate hairs it is similar to *Conostegia caelestis* which is allopatric occurring in northern Central America. In addition, *Conostegia brenesii* tends to be a shrubby species whereas *Conostegia caelestis* tends to be a larger tree. The flowering time differs with *Conostegia caelestis* flowering in the first half of the year and *Conostegia brenesii* flowering in the second half of the year consistently from July to September. The molecular phylogeny does not place these species as sister taxa, which suggests convergent evolution in the dense stipitate stellate indument (Fig. 1). *Conostegia brenesii* falls sister to the *Conostegia montana*-*Conostegia setosa* complex in the molecular phylogeny.

#### Specimens examined.

**COSTA RICA. Alajuela**: Zapote, San Carlos, Caribe watershed, Smith 1102 (NY); La Palma de San Ramón, Brenes 5633 (CR, NY); San Carlos, P. N. Juan Castro Blanco, entrando por San Vicente faldas del Cerro Platanar, Rodríguez et al. 6050 (INB, NY); Vara Blanca de Sarapiquí, north slope of the Central Cordillera, Skutch 3161 (NY); Forests of La Palma, Tonduz 12580 (NY). **Puntarenas**: R. B. Monteverde, Haber 352 (CR). **San José**: Cuenca del Sarapiquí, Braulio Carrillo, cerca de el túnel, Kriebel 4907, 5631 (INB).

### Conostegia caelestis

Taxon classificationPlantaeMyrtalesMelastomataceae

Standl.

Fig. 73

Conostegia caelestis Standl., Field Mus. Nat. Hist. Publ. Bot. Series. 4: 318. 1929. Type: British Honduras (= Belize). Big Creek: Mullins river road, 15 m, 8 March 1929, W. Schipp 63 (holotype: F!, isotypes: A, BM, CAS!, GH, MICH, MO!, NY!, S, UC, US!).
Conostegia hondurensis Standl. ex Yuncker, Field Mus. Nat. Hist. Publ. Bot. Series. 9: 322. 1940. Type: Honduras. Atlántida: bank of Danto river, slopes of Mt. Cangrejal, vicinity of La Ceiba, 300 m, 6 August 1938, T. Yuncker, J. Koepper, & K. Wagner 8818 (holotype: F!, isotypes: BM, GH, K!, MO!, NY!, US!).

#### Description.

Small trees 2–12 m tall with tetragonal stems that soon become terete and are covered with simple and mostly stellate-stipitate trichomes to 1.5 mm long; the nodal line present but slight. Leaves of a pair equal to somewhat unequal in length. Petioles 0.6–3.8 cm long. Leaves 5.5–26 × 2–7.8 cm long, 3–5 nerved or 3–5 plinerved, if plinerved, the innermost pair of primary veins arising up to 1 cm above the in opposite or subopposite fashion, elliptical to obovate, the base acute or cuneate, the apex acute to acuminate, the margin entire to serrate, adaxial surface short-setose with simple bristles and sometimes stipitate stellate trichomes on the mid vein, abaxially covered with stipitate stellate hairs. Inflorescence a terminal panicle 3.9–9 cm long branched above the base, accessory branches absent, bracts linear, up to 5 mm long, deciduous, bracteoles 2–10 mm long, deciduous. Flowers 5–6 merous, calyptrate. Flower buds 5.8–7.6 × 2.9–3.5 mm, pyriform oblong, the base flat to rounded, the apex acute to apiculate, not constricted, hypanthium 3–3.25 × 3–3.25 mm, covered with stipitate stellate hairs. Petals 6–10 × 6.5–7 mm, white, obovate, not observed at anthesis, glabrous, 3 lobed. Stamens (14-)16–18, slightly zygomorphic, the filament 2.5–3 mm, not geniculate, anthers 2–2.5 × 0.3–0.6 mm, linear, reportedly white, somewhat laterally compressed, the base sagittate, the pore terminal, 0.1–0.15 mm. Ovary 7–10 locular, inferior, forming a glabrous collar around the base of the style. Style 3.5–4 mm, bent near the tip, vertical distance from the stigma to the anthers ca. -0.6 mm, horizontal distance absent; stigma subcapitate, 0.9–1.1 mm wide. Berry 6–7.5 × 6–7.5 mm, purple-black. Seeds 0.33–0.5 mm, pyramidal, smooth.

**Figure 73.**
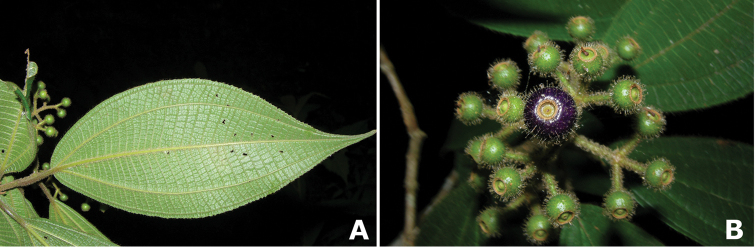
*Conostegia caelestis*. **A** Leaf abaxial surface. **B** Infructescence. Photos of specimen vouchered *R. Kriebel 5588*.

#### Distribution

**Figure 74.**
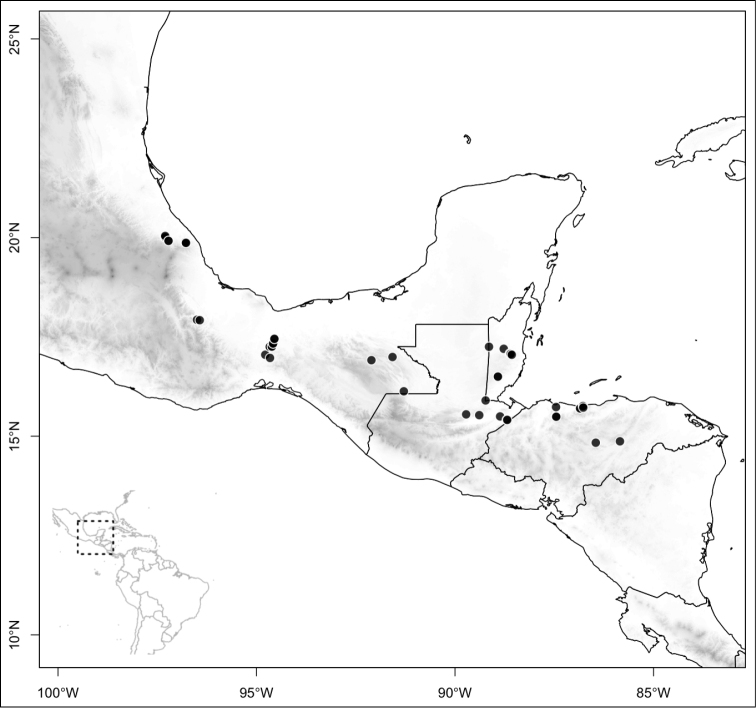
Distribution of *Conostegia caelestis*.

(Fig. 74). Known from Mexico through Guatemala and Belize to Honduras from sea level to 1000 m in elevation.

*Conostegia caelestis* is easily distinguished from its congeners on the basis of its dense indument of stipitate stellate hairs especially on the leaf abaxial surface, inflorescence and hypanthia. As Schnell (1996) noted, this species has a very definite flowering time and is never fruiting at the same time that it is flowering, suggesting a long maturation time for the berries. *Conostegia caelestis* shares the dense indument of stipitate stellate hairs with *Conostegia brenesii* but they are not closely related. See further discussion of their differences under *Conostegia brenesii*.

#### Specimens examined.

**MEXICO. Chiapas**: west end of Laguna Ocotal Grande, Municipio de Ocosingo, Breedlove 15700 (NY).

**BELIZE. El Cayo District**: Humming Bird Highway, Gentle 8633 (MO, NY).

**GUATEMALA. Alta Verapaz**: Sebol in high forest about 2 km east, Contreras 4587 (NY). **Izabal**: Vicinity of Lago Izabal 1–2 km south of village of Izabal, Jones et al. 3014 (NY); Sierra Caral, Quebrada atravesada por el sendero al noreste de la casa de investigadores, hacia la Finca Bonanza, Kriebel et al. 5588, 5617 (NY, USCG). HONDURAS. **Atlantida**: along Río Danta road to La Presa vicinity of La Ceiba, Molina 20822 (NY). **Olancho**: Quebrada Catacamas cerca de la presa en Montaña Peña Blanca, Molina 8352 (NY); Lancetilla mountain, Molina and Molina 25611 (NY).

### Conostegia chiriquensis

Taxon classificationPlantaeMyrtalesMelastomataceae

Gleason

Conostegia chiriquensis Gleason in R. E. Woodson, Jr. and R. W. Schery, (Eds), Flora of Panama. Ann. Missouri Bot. Gard. 45: 203–304. 1941. Type: Panama. Chiriquí: Vicinity of Finca Lérida, 1750 m. elev., 7–11 July 1940, R. Woodson & R. Schery 376 (holotype: NY!, isotype: GH!).

#### Description.

Trees 4–20 m tall and with tetragonal and ridged stems that are glabrous or sometimes with scattered sessile stellate trichomes; the nodal line present. Leaves of a pair equal to somewhat unequal in length. Petioles 1–4.8 cm long. Leaf blades 6.2–16.5 × 3.2–7.7 cm, 3–5 nerved or slightly plinerved, elliptic, base obtuse to rounded, apex obtuse to acute and short acuminate, the margin entire to denticulate, essentially glabrous on both surfaces. Inflorescence terminal, 5.7–13.5 cm long branched above the base but sometimes appearing branched at the base because of multiple inflorescences arising at opposing meristems at the terminal node, accessory branches present, bracts absent or very early deciduous, the bracteoles 1–5 mm, deciduous. Pedicels 3–6 mm. Flowers 7–11 merous, calyptrate. Floral buds 7.2–13 × 3.2–7.6 mm, mostly ellipsoid pyriform, constricted below the middle, the base flat to rounded, apiculate at the apex. Petals 10–12 × 5–6 mm, white to pale lavender, obtriangular, spreading, the apex rounded-truncate to emarginate, glabrous. Stamens 14–24, 8.5–10 mm long, androecium zygomorphic, the filament 4.25–5.25 mm, white but apparently turning red on some specimens perhaps when old, anthers 3.25–4.5 × 0.1–0.2 mm, subulate and slightly recurved, sagittate at the base, yellow except for a hugh of rose at the base of thecae dorsally in one specimen, the pore ventral-terminal, ca. 0.3 mm wide. Ovary 6–12 locular, inferior, glabrous and lacking a distinct apical collar. Style 9–12 mm, bent away from the stamens, vertical and horizontal distance not assessed, stigma barely expanded, made of lobes that are almost non discernible, ca. 1–2 mm wide. Berry 6–7 × 6–7 mm, blue-black or purple. Seeds 0.5–0.65 mm, pyramidal and smooth.

#### Distribution

**Figure 75.**
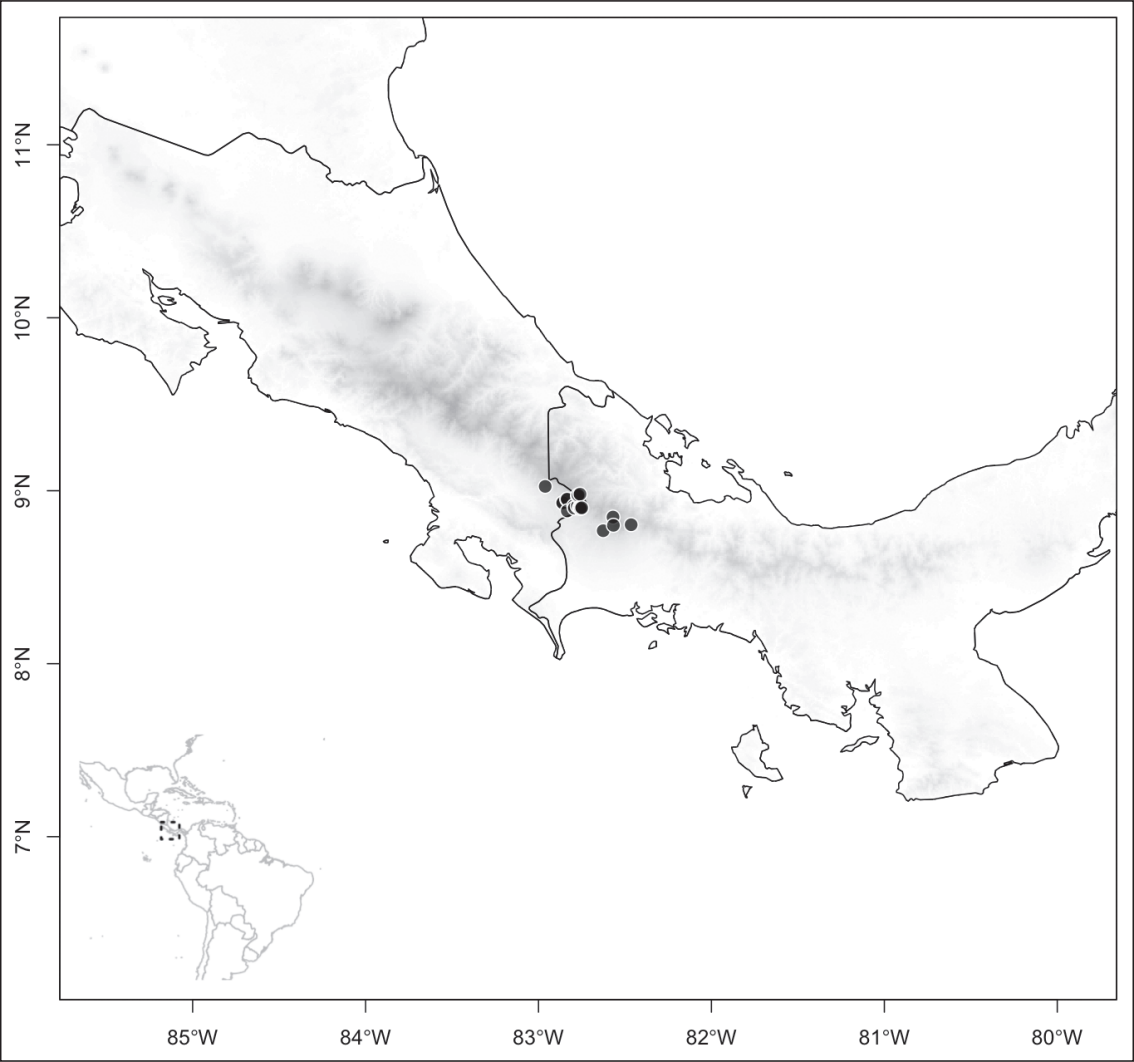
Distribution of *Conostegia chiriquensis*.

(Fig. 75). Endemic to cloud forests in Costa Rica and Panama from 1000–2100 m. In Panama restricted to peaks near the Canal Area as well as Volcan Chiriquí. In Costa Rica common in Las Tablas Protected Zone on the Costa Rica-Panama border.

*Conostegia chiriquensis* is similar and possibly closely related to *Conostegia pittieri*, especially in their glabrescence, apiculate calpytras and style lacking a conspicuous crater in the middle. *Conostegia chiriquensis* differs most notably in the more slender style that is not capitate like in *Conostegia pittieri*. Schnell (1996) noted that the petals and particularly the style are persistent in *Conostegia chiriquensis* and this differs from *Conostegia pittieri* where only the petals tend to persist. In the protologue Gleason compared this species to *Conostegia rhodopetala* and *Conostegia pittieri* (Gleason, 1941). *Conostegia rhodopetala* can have an apiculate calyptra but has a noticeably short style lacking lobes. In Schnell’s (1996) key *Conostegia chiriquensis* is included in the group of species with large stigmas mostly because of the presence of lobes since it is barely expanded. Almeda noted “Gardenia like fragrance on flowers of this species (*Almeda et al. 6599*-CAS, NY). Schnell (1996) reports the infestation of anthers in unopened flower buds by gall wasps on the specimen *Woodson and Schery 479* (NY).

#### Specimens examined.

**COSTA RICA. Puntarenas**: Lumber road along Fila Tigre S and E of Las Alturas between Río Cotón and Río Quebrada Nochebuena, Almeda et al. 6597, 6599 (CAS, NY); Cantón de Coto Brus, Z. P. Las Tablas, Est. Biológica Las Alturas, Alfaro 2365 (INB, NY); Cantón de Coto Brus, Z. P. Las Tablas, Hacienda La Amistad, Zona La Neblina, Solano et al. 752 (INB, NY).

**PANAMA. Chiriquí**: Along the rock road to Lago del Volcán Barú and due SW of El Hato del Volcán, Almeda et al. 6205 (NY); Trail from Paso Ancho to Monte Liro upper valley of Río Chririquí Viejo, Allen 1480 (NY); R. Chiriquí Viejo valley, in Bambita Woods, White 47 (NY); Valley of the upper Río Chiriquí Viejo, White 324 (NY); west of Aserradero Cerro de Punta, Stern and Chambers 64 (NY); Vicinity of Callejón Seco, Volcán de Chiriquí, Woodson and Schery 479 (NY).

### Conostegia cuatrecasii

Taxon classificationPlantaeMyrtalesMelastomataceae

Gleason

Fig. 76

Conostegia cuatrecasii Gleason, Bull. Torrey Bot. Club 72: 473. 1945. Type: Colombia. Depto. del Valle: Silva, Río Cajambre, Costa del Pacífico, 5–80 m, 5–15 May 1944, J. Cuatrecasas 17612 (holotype: NY!, isotype: F!).

#### Description.

Shrubs to small trees 2–12 m tall with strongly tetragonal stems that are glabrous or with scattered sessile stellate trichomes; the nodal line inconspicuous or evident as a whitish line, not elevated. Leaves of a pair equal to somewhat unequal in length. Petioles 0.9–6 cm. Leaf blades 11–25.7 × 5–12.9 cm, 3–5 plinerved, with the innermost pair of vein arising just above the base in opposite or sub alternate fashion, elliptic to ovate, base acute to rounded, apex acuminate, margin entire to denticulate, glabrous on both surfaces. Inflorescence a terminal panicle 6–25 cm long branched above the base base but sometimes appearing branched at the base because of multiple inflorescences arising at opposing meristems at the terminal node, accessory branches absent or present, the bracts absent or early deciduous, bracteoles to 6 mm long, subulate, usually persistent at anthesis and deciduous in fruit. Pedicel 5–8 mm, lengthening in fruit. Flowers 6–8 merous, calyptrate. Floral buds 6–11 × 3–7 mm, slightly constricted at the torus, the base flat, the apex acuminate; the hypanthium 5–6 × 5–5.5 mm. Petals 7–12 × 7–11.5 mm, pink to lilac or white, obovate, spreading, apically emarginate, glabrous, spreading, the margin entire to undulate. Stamens 12–15(-17), 9–10 mm long, slightly zygomorphic, the filament 5–5.5 mm, white, anthers 4–4.5 × 0.8–1.2 mm, linear-oblong and recurved, somewhat laterally compressed, sagittate at the base, yellow, the pore 0.12–0.13 mm, slightly ventrally inclined. Ovary (6-)7–9(-11) locular, inferior, the apex glabrous and elevated into a pronounced collar around the style base. Style 4–6.7 mm, bent upward below the stigma, distance between the anther and the stigma -2 – -0.5 mm, stigma broadly capitate, 0.3–0.5 mm wide. Berry 8–9 × 8–9 mm, purple-black. Seeds ca. 0.4 mm long, triangular in profile view, smooth.

**Figure 76.**
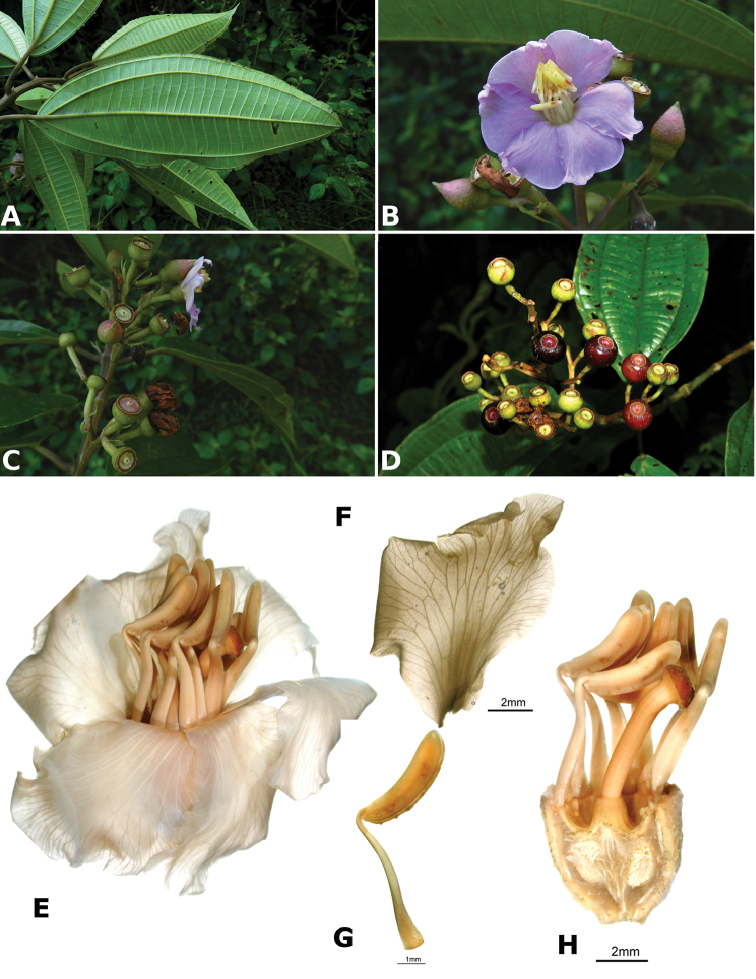
*Conostegia cuatrecasii*. **A** Leaf abaxial surface **B** Frontal view of flower at anthesis **C** Inflorescence with side view of flower at anthesis **D** Infructescence **E** Pickled flower at anthesis **F** Petal **G** Stamen **H** Longitudinal section of flower at anthesis with petals removed. Photos of specimen vouchered *R. Kriebel and Burke 5673*.

#### Distribution

**Figure 77.**
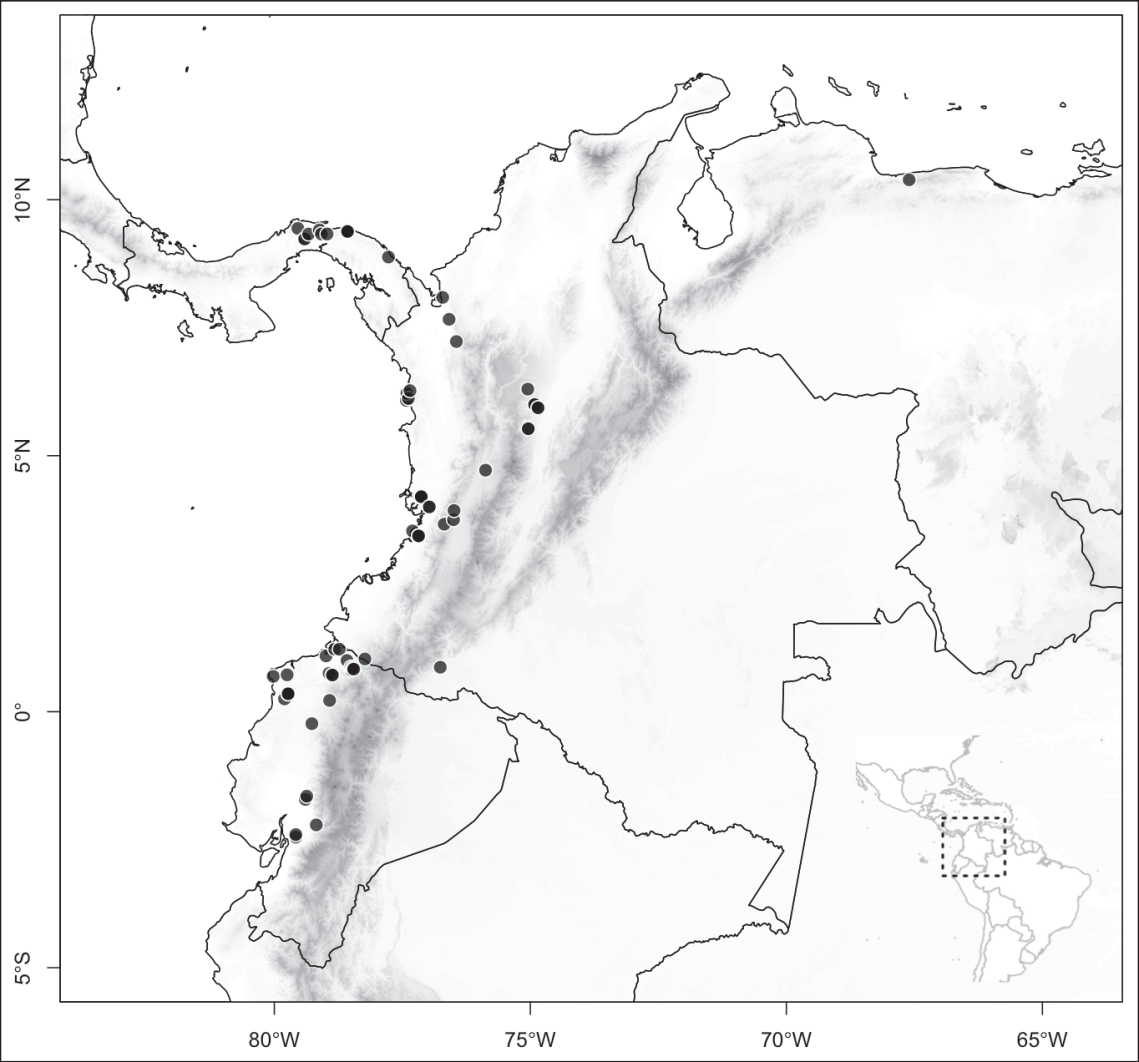
Distribution of *Conostegia cuatrecasii*.

(Fig. 77). Ranging from Panama to Colombia, Venezuela and Ecuador, and the coastal range of Venezuela, 0–1000 m in elevation.

This species is particularly distinctive when found in the field with its large lavender flowers. Herbarium specimens with flower buds on the other hand can be hard to separate from some populations of *Conostegia superba*. In general both can be separated on the basis of the larger flowers with usually lavender petals and more floral parts in *Conostegia cuatrecasii* (versus smaller flowers with white petals in *Conostegia superba*). Also, *Conostegia cuatrecasii* tend to have a more markedly acute calyptra apex than *Conostegia superba*. Schnell (1996) further separated the two stating that the stigma in *Conostegia cuatrecasii* was lobed but I did not observe lobes in a specimen collected in spirit in Cerro Jefe, Panama. Also, Schnell (1996) stated the floral buds in *Conostegia superba* are clearly constricted but I did not observe constriction in the floral buds of *Conostegia superba* in Izabal, Guatemala. Good fragrance has been reported in the flowers of this species (*Kriebel and Burke 5681*-NY).

#### Specimens examined.

**PANAMA. Panamá**: P. N. Chagres, sendero hacia Cerro Jefe, Kriebel and Burke 5681 (NY, PMA).

**COLOMBIA. Antioquia**: Rio Chigorodo, Forest on Quebrada Congo 11 km east of Chigorodo 40 km south of Turbo, Haught 4713 (NY); In damp forest on ridge west of Quebrada Mercedes east of Turbo, Haught 4973 (NY). **Chocó**: Río San Juan margen derecha, Quebrada del Taparal, Cuatrecasas 21468 (NY); Banks of Quebrada Togoromá, Killip and Cuatrecasas 39089 (NY); Rio Mutata tributary of Río El Valle between base of Alto de Buey and mouth of river, Gentry and Fallen 17283 (MO, NY). **El Valle**: Cordillera Occidental, vertiente occidental, hoya del río Anchicayá ado derecho bosques entre Pavas y Miramar, Cuatrecasas 14386 (NY); Cordillera Occidental, vertiente occidental, Hoya del Río Digua lado izquierdo, Piedra de Moler, Cuatrecasas 15142 (NY); Costa del Pacífico, río Cajambre, Barco, Cuatrecasas 17001 (NY); Costa del Pacífico, río Cajambre, Quebrada de Ordonez, Cuatrecasas 17273 (NY); Río Digua Valley between La Elsa and Río Blanco, Killip 34712 (NY).

**ECUADOR. Bolívar**: Trip to Bucay 87 km e. of Eloy Alfaro in walk of 5 km up trail n.e. in foothills along pipeline to intake of Guayaquil water supply, Little 6736 (NY). **Esmeraldas**: Quininde Cantón, Bilsa Biological Station, Mache mountains 35 km W of Quinindé 5 km W of Santa Isabel, Clark and Troya 680 (NY); Carretera Lita-Alto Tambo-La Punta, Gudiño and Moran 1286 (MO, NY); Río San Miguel one hour upstream from San Miguel de Cayapas, Holm-Nielsen et al. 25494 (MO, NY); Rio Onzole on estero Chontaduro, Játiva and Epling 1103 (NY); near Borbon, Játiva and Epling 2203 (NY). **Los Rios**: Hacienda Clementina, Samama, Harling 284 (NY); Hacienda Clementina, Cerro Samama, Cornejo and Yoza 8188 (NY). **Imbabura**: Lita, Palacios 12231 (NY). **Manabí**: Pedernales, Reserva Ecológica Mache-Chindul, Comunidad Ambache (vía marginal de la costa-Chindul), Clark et al. 4229 (MO, NY). **Pichincha**: Distrito Metropolitano de Quito, carretera Mashpi-Los Bancos, Ulloa et al. 1935 (MO, NY).

**VENEZUELA** (fide Schnell). **Carabobo**: Mpio. Autónomo Mora, cuenca del río Moron, Díaz 153 (US).

### Conostegia fragrantissima

Taxon classificationPlantaeMyrtalesMelastomataceae

Almeda

Fig. 78

Conostegia fragrantissima Almeda, Proc. Calif. Acad. Sci. 46: 327. 1990. Type: Panama. Bocas del Toro: Fortuna Dam area, along continental divide bordering Chiriqui province, 1200-1300 m, 10 March 1988, F. Almeda, T. Daniel, & G. McPherson 6064 (holotype: CAS!, isotypes: MO!, US, AAU, BM, BR, CR!, DUKE, F, MEXU, PMA, TEX, NY!).

#### Description.

Shrubs to small trees 4–11 m tall with apically tetragonal glabrous stems; the nodal line present. Leaves of a pair equal to somewhat unequal in length. Petioles 0.7–3 cm long. Leaf blades 3.5–10 × 1.2–4 cm, 3–5 plinerved, with the innermost pair of veins diverging from the mid vein up to 5 mm above the blade base, mostly elliptic, glabrous adaxially, glabrous or with some scattered minute trichomes abaxially, the base acute, the apex acuminate to caudate acuminate, margin entire. Inflorescence a terminal panicle 3–10 cm long branched well above the base, accessory branches apparently absent, the rachis glabrous or with some minute furfuraceous lepidote hairs, bracts apparently early deciduous, not observed, bracteoles 0.5–2 mm, narrowly triangular to subulate, early deciduous. Pedicel 2–7 mm long. Flowers 6–7 merous, calyptrate. Flower buds 5–7 × 2–3.25 mm, oblong-ellipsoid, the base rounded, the apex acute to apiculate, slightly constricted below the calyptra; the hypanthium 2.75–3.25 × 3.5–4 mm, glabrous or sparsely furfuraceous lepidote. Petals 4.5–5 × 4–4.5 mm, white with a red band at the base, obovate, reflexed to slightly spreading, glabrous, the apex three lobed. Stamens 14–19, 4–5.5 mm long, zygomorphic resulting from their bending all to one side, the filament 2.5–3.5 mm, white, non geniculate, anthers 1.5–2.3 × 0.65–0.75 mm, linear-oblong, sagittate at the base, yellow-orange, the connective thickened and forming a slight hump, the pore ventrally inclined, 0.25 mm wide. Ovary 6–7 locular, inferior, the apex glabrous and lacking an elevated collar. Style 5–5.5 mm, bending opposite the stamens, vertical distance between the anther and the stigma absent, horizontal distance 2–2.3 mm, the stigma capitellate, with 6 or 7 lobes, 1.4–1.5 mm wide. Berry 3 × 3.5–4 mm, purple black. Seeds 0.5–0.75 mm, oblong or narrowly pyramidate, smooth.

**Figure 78.**
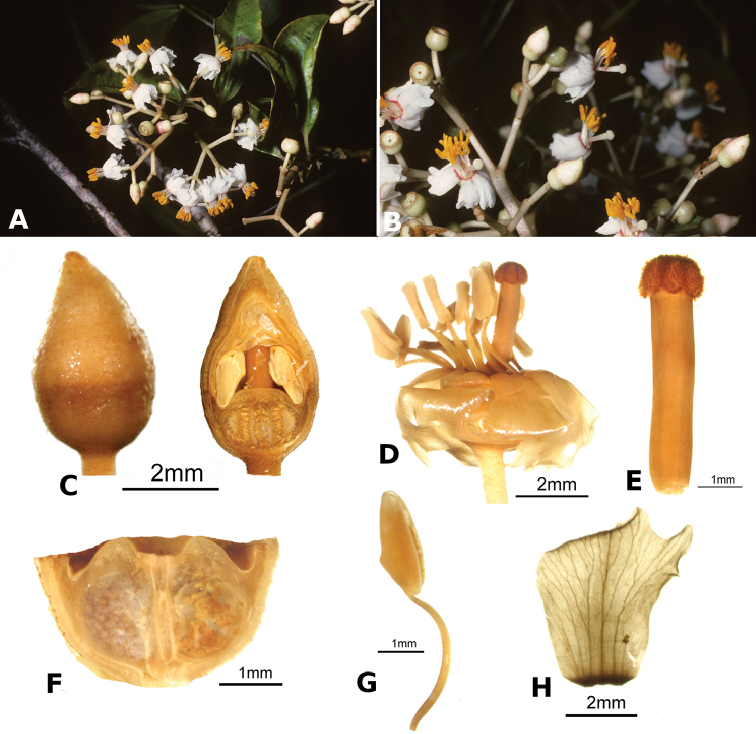
*Conostegia fragrantissima*. **A** Inflorescence **B** Close up of flowers at anthesis **C** Flowers bud with longitudinal section to the side **D** Pickled flower at anthesis **E** Style **F** Longitudinal section of the hypanthium **G** Stamen **H** Petal. Photos A-B of specimen vouchered *F. Almeda 6040* (CAS) and taken by Frank Almeda **C–H** of specimen vouchered *R. Kriebel and D. Solano 3174*.

#### Distribution

**Figure 79.**
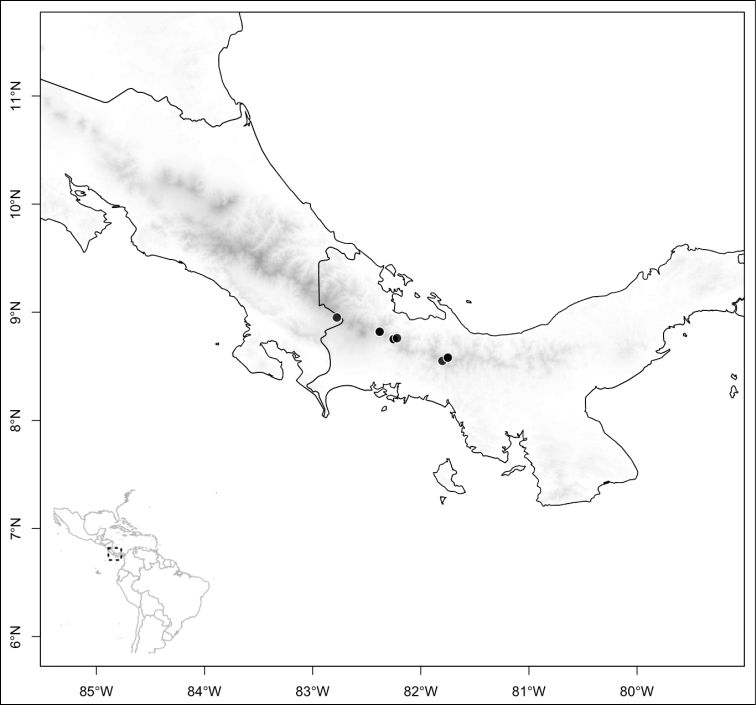
Distribution of *Conostegia fragrantissima*.

(Fig. 79). Known from south eastern Costa Rica and west Panama, 1200–1700 m elevation.

Schnell (1996) and Almeda (2009) synonimized *Conostegia fragrantissima* under *Conostegia montana*. Despite the heterogeneity present in *Conostegia montana*, there was no stigma lobes in several living populations studied, or was there an evident style declination as is present in *Conostegia fragrantissima*. My observations contrast with Schnell’s because he stated most populations of *Conostegia montana* do present a lobed stigma and a declinate style. Although Schnell (1996) makes a good point in stating that collectors tend to overlook fragrance, *Conostegia montana* is one of the most frequently collected species of *Conostegia* and none of the specimens studied cited floral fragrance. On the other hand, of the only six specimens of *Conostegia fragrantissima*, one describes evident fragrance (*Almeda et al. 6040*-CAS, MO, NY). The stigma lobes present in *Conostegia fragrantissima* as well as the declinate style (Fig. 78) also suggest a possible relationship to *Conostegia pittieri*. Unfortunately this species was not included in the phylogeny of *Conostegia* and thus its close relatives remain unknown.

#### Specimens examined.

**COSTARICA. Puntarenas**: Coto Brus, Z. P. Las Tablas, sendero Las Tablas camino a Cotoncito, Kriebel and Solano 3174 (INB, NY).

**PANAMA. Chiriquí**: SE slopes and summit of Cerro Pate Macho, trail between Rio Palo Alto, 4 km NE of Boquete, Sytsma et al. 4884 (MO, NY).

### Conostegia hirtella

Taxon classificationPlantaeMyrtalesMelastomataceae

Cogn

Conostegia hirtella Cogn. in J. D. Smith, Bot. Gaz 16: 4. 1891. Type: Guatemala. Alta Verapaz: Pansamala, 1170 m, May 1887, H. von Tuerckheim 1233 (holotype: BR; isotypes: BR, GH, K!, NY!, P (fide Almeda in Schnell (1996)), PH!, US!).
Conostegia gleasoniana Standl. & Steyerm., Field Mus. Publ. Bot. 22: 361. 1940. Type: Guatemala. Alta Verapaz: Damp forest, region of Cocola, NE of Carcha,1200 m, 2 April 1938, P. Standley 70317 (F!).

#### Description.

Shrubs to small trees 1.8–10 m tall with tetragonal stems becoming terete with age and that are finely puberulent with minute sessile stellate and stipitate-stellate trichomes; the nodal line present but slight. Leaves of a pair equal to somewhat unequal in length. Petioles 0.8–3.2 cm long. Leaves 6.5–16.1 × 1.75–5.7 cm, 3–5 nerved or if plinerved, with the innermost pair of primary veins diverging from the mid vein up to 2 cm above the base in opposite to sub opposite fashion, adaxially glabrous, abaxially with stipitate stellate hairs mostly on the primary veins and with minute stellate trichomes on higher order veins, some specimens with evident pocket domatia at the base abaxially, narrowly ovate to narrowly elliptic, acute at the base and apex, the margins undulate-dentate to entire. Inflorescence terminal panicle 4.3–11.8 cm long branched above the base but sometimes appearing branched at the base because of multiple inflorescences arising at opposing meristems at the terminal node, accessory branches present, bracts and bracteoles 0.6–4 mm long, subulate to linear-lanceolate, deciduous. Pedicels 2–5 mm long. Flowers 5–6 merous, calyptrate. Flower bud ca. 4–6 × 2.5–3.5 mm, obovate, obtuse or rounded at base, acute to short-apiculate at the apex, not constricted, the hypanthium 2.5–3 × 2.5–3 mm, smooth. Petals ca. 5 × 4 mm, white or pink, spathulate, emarginate, glabrous. Stamens 13–17, 3–4 mm long, the filament ca. 1.5–2 mm, white, anthers 1.8–2.0 × 0.4–0.5 mm, linear-oblong, straight or slightly recurved, laterally compressed, thickened dorsally, yellow, the pore ca. 0.2 mm, terminal. Ovary 5–6 locular, inferior, apically glabrous and forming a collar around the style base. Style ca. 3 mm, straight distance between the anther and the stigma -0.5 – -0.1 mm; stigma punctiform, ca. 0.6 mm wide wide. Berry ca. 5 × 5 mm, dark purple. Seeds 0.4–0.6 mm long, obovoid, angulate or not, essentially smooth but frequently with the periclinal walls elevated to give a roughened look.

#### Distribution

**Figure 80.**
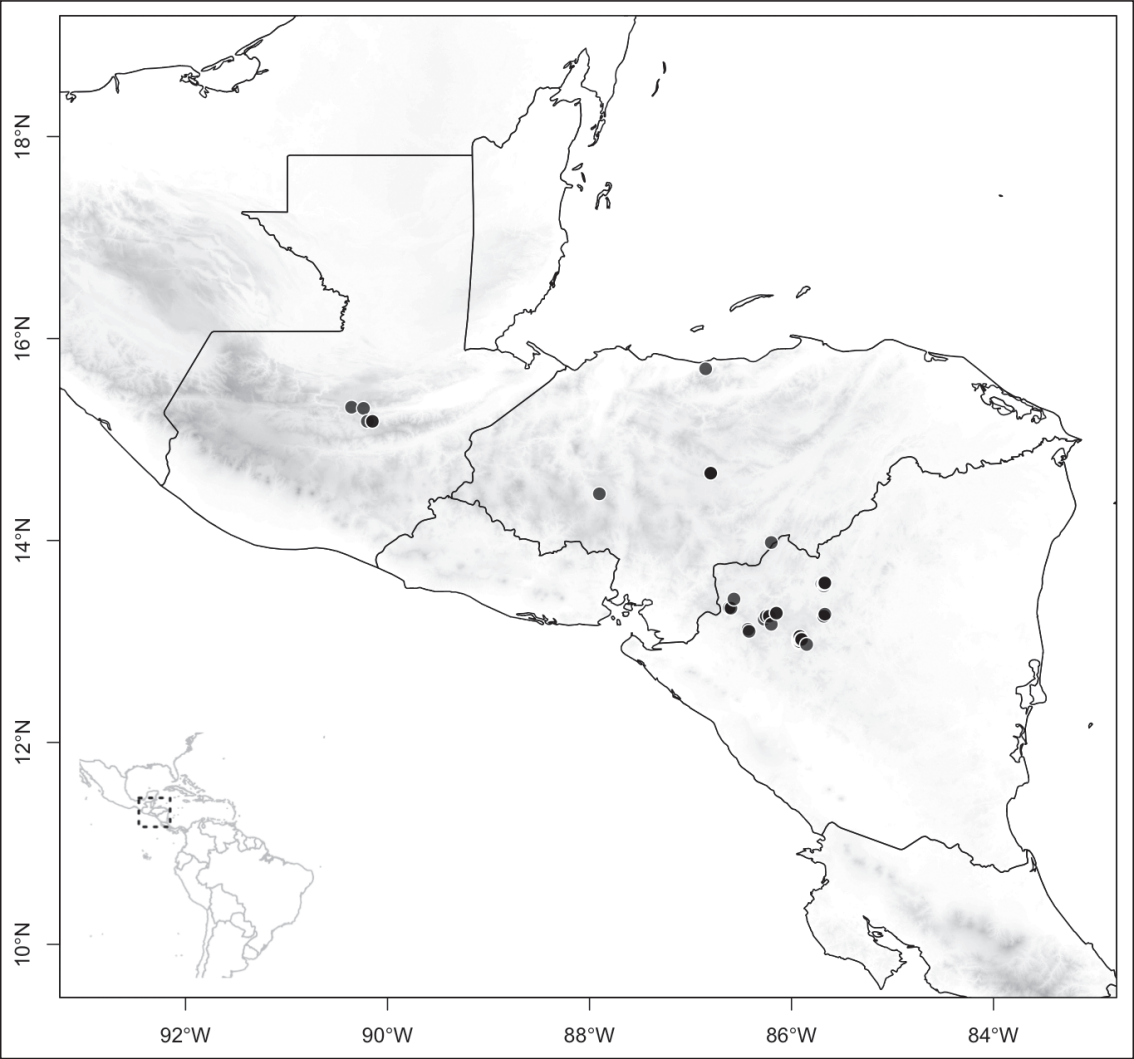
Distribution of *Conostegia hirtella*.

(Fig. 80). Known from Guatemala, Honduras and Nicaragua, 700–1700 m.

*Conostegia hirtella* is quite similar to *Conostegia montana*, a very variable species. It is recognized here on the basis of the presence of stipitate stellate hairs which *Conostegia montana* lacks. Schnell (1996) considered *Conostegia hirtella* to be close to *Conostegia caelestis* on the basis of those stipitate stellate trichomes. The much sparser pubescence in *Conostegia hirtella* compared to the dense indument in all the plant in *Conostegia caelestis* makes it diffcult to confuse them. Unfortunately *Conostegia hirtella* was not included in the molecular phylogeny to further help elucidate its close relatives.

#### Specimens examined.

**GUATEMALA. Alta Verapaz**: Bosque mixto de Chamal, margenes del Río Cobán, Molina 12142 (NY); Mountains east of Tactic on road to Tamahú, Standley 71305 (NY); Wet forest near Tactic, above the bridge across Río Frío, Standley 90285 (NY).

**HONDURAS. Comayagua**: Bosque nuboso de Cordillera de Misoco o Volcán de Guaimaca entre los Departamentos de Olancho y Morazán, Molina 3189 (NY); Bosque Pino-Liquidambar de Montana, la Choca, Cordillera Comayagua, cerca de Coyocutena, Molina 7120 (NY). **Morazán**: Bosque de nubes del Volcán de Guaimaca, Cordillera de Misoco, Molina 6094 (NY).

**NICARAGUA. Estelí**: Dept. of Estelí on the border with Madriz, Cerro Pataste, Neill 121 (NY); on border with Madriz, Cerro Pataste, Neill 128 (NY). **Matagalpa**: Cut over cloud forest area El Porvenir, Cordillera Central de Nicaragua, Molina 20522 (MO, NY); Cut over cloud forest of El Picacho east of Sant María de Ostuma, Cordillera Dariense, Molina and Molina 30522 (NY); Cut over cloud forest area road to Aranjuez, Cordillera Central de Nicaragua, Williams 20143 (NY); road to La Fundadora, cloud forest area north of Sta. María de Ostuma, Cordillera Central de Nicaragua, Williams 24858, 24926 (MO, NY).

### Conostegia icosandra

Taxon classificationPlantaeMyrtalesMelastomataceae

(Swartz in Wikstr.) Urban

Fig. 81

Conostegia icosandra (Swartz in Wikstr.) Urban, Rep. Sp. Nov. 17: 404. 1921. Melastoma icosandrum Swartz in Wikstrom, Kongl. Vetensk. Akad. Handl. 64. 1827. Type: O. Swartz s.n. (not seen; application of name based on Urban’s treatment). Lectotype: Guadeloupe: J. Forsstrom s.n. (designated by Howard and Kellogg, J. Arnold Arb. 67: 244. 1986): S!).
Conostegia subhirsuta DC, Prodr. 3: 174. 1828. Lectotype (designated here): Cuba. Havana: 1825, J. de la Ossa s. n. (G!). Additional syntypes: Guadeloupe. Richard s.n. (G!, photograph of P specimen at IJ (Schnell 1996)).
Conostegia mexicana Cogn., DC. Monog. Phan. 7: 707. 1891. Lectotype (designated here): Mexico. Monte Pelado, July 1840–1849, H. Galeotti 2963 (BR!); Mexico. Huatusco, 1335 m, August 1888, Comisión Geografico-Exploradora 401 (BR). Later homonym of Conostegia mexicana (Bonpl.) Ser. ex DC, Prodr. 3: 175. 1828 (see “Excluded taxa or uncertain names” section for further discussion of this name).
Conostegia icosandra var. crenata Urban, Rep. Sp. Nov. 22: 222. 1926. Type: Cuba. Oriente: Arroyo Jimenez, Sierra Maestra, 600–700 m, Ekman 14783 (holotype: S!; isotype: NY!). Conostegia icosandra subsp. crenata (Urban) Borhidi and Muniz, Bot. Kozelem. 58: 176. 1971.
Conostegia lundellii Gleason, Publ. Carnegie lnst. Wash. 522: 348. 1940. Type: British Honduras (= Belize). El Cayo District: San Augustín, Mountain Pine Ridge, July–August 1936, C. Lundell 6587 (holotype NY!, isotypes MICH!, NY!).

#### Description.

Shrubs to trees 1–15.3 m tall with somewhat tetragonal and ridged stems that are glabrescent to hirsute with sessile and stipitate stellate as well as branching hairs; the nodal line present. Leaves of a pair equal to somewhat unequal in length. Petioles 1–6.9 cm long. Leaves 4.6–25.2 × 1.5–11 cm, 3–5 plinerved, with the innermost diverging from the midvein just above the blade base in opposite or alternate fashion, elliptic to ovate, acute to rounded at the base, apex acute to acuminate, the margin entire to denticulate, adaxially generally glabrous, abaxially glabrous to densely hirsute with sessile or stipitate stellate and branching trichomes. Inflorescence a terminal panicle, 2.8–18 cm long branched above the base but sometimes appearing branched at the base because of multiple inflorescences arising at opposing meristems at the terminal node, accessory branches absent or present, the axis glabrous to hirsute with sessile and stipitate stellate, bracts linear to elliptic, up to 1.5 cm long, persistent or deciduous, bracteoles 1–10 mm long, oblong to ovate, mostly persistent. Pedicel 1–10 mm long. Flowers (5-)6–9(-11) merous, calyptrate. Floral buds 5–11 × 4.5–8.5 mm, rounded at the base, obtuse to rounded and apiculate at the apex, not constricted, the hypanthial and ca-lycine portions not or only slightly differentiated; the hypanthium 3.5–4.5 × 5–5.5 mm, glabrous to puberulent. Petals 7–8 × 7–8 mm, white, broadly obovate, spreading and rotate, emarginate, glabrous. Stamens (17)19–26 (30), 6.5–9 mm long, somewhat zygomorphic, the filament 4.5–5 mm, white, anthers 3.27–3.75 × 1.5–2 mm, oblong, laterally compressed, the base sagittate, yellow, the pore 0.1–0.15 mm, terminal or subterminal. Ovary 8–15 locular, inferior, apically glabrous and forming a stylar collar. Style 5.5–6 mm, slightly to strongly bent, vertical distance from the anther to the stigma ca. -0.25, horizontal distance 0.5–1.5 mm; stigma capitate, consisting of 8–15 lobes that are difficult to distinguish, not crateriform, the stigma 3–3.5 mm wide. Berry 9–12 × 7–10 mm, blue-black or purple. Seeds 0.4–0.8 mm long, obovoid, smooth.

**Figure 81.**
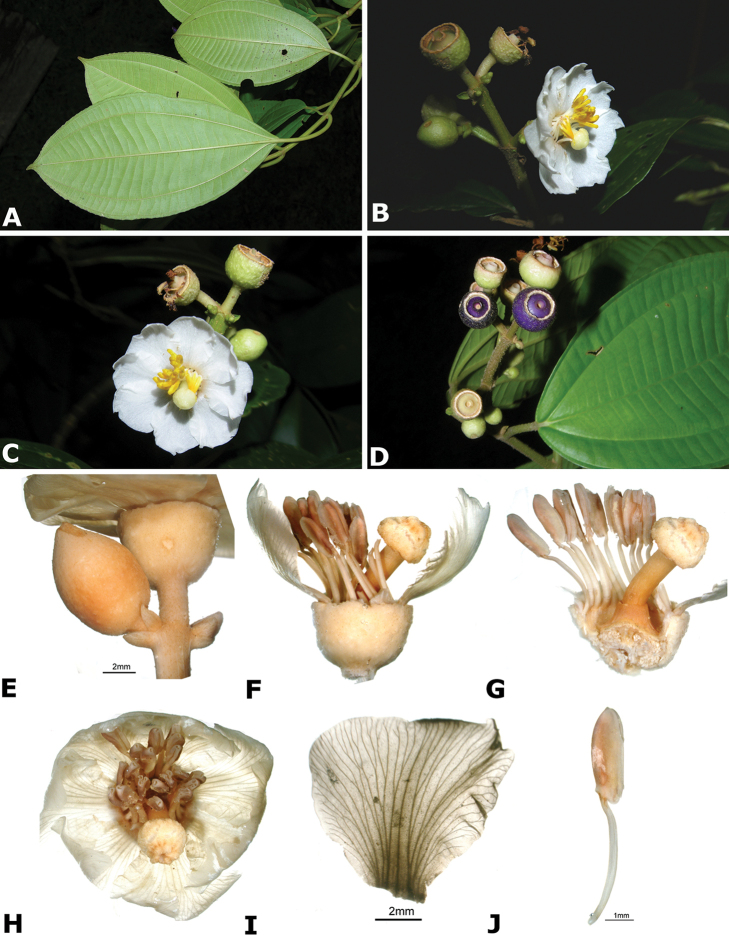
*Conostegia icosandra*. **A** Leaf abaxial folia surface **B** Side view of flower at anthesis **C** Frontal view of flower at anthesis **D** Infructescence **E** Flower bud with detail of bracteoles at the base **F** Pickled flower at anthesis **G** Longitudinal section of flower at anthesis **H** Frontal view of flower at anthesis **I** Petal **J** Stamen. Photos of specimen vouchered *R. Kriebel 5578* (flowers) and *R. Kriebel 5580* (fruits).

#### Distribution

**Figure 82.**
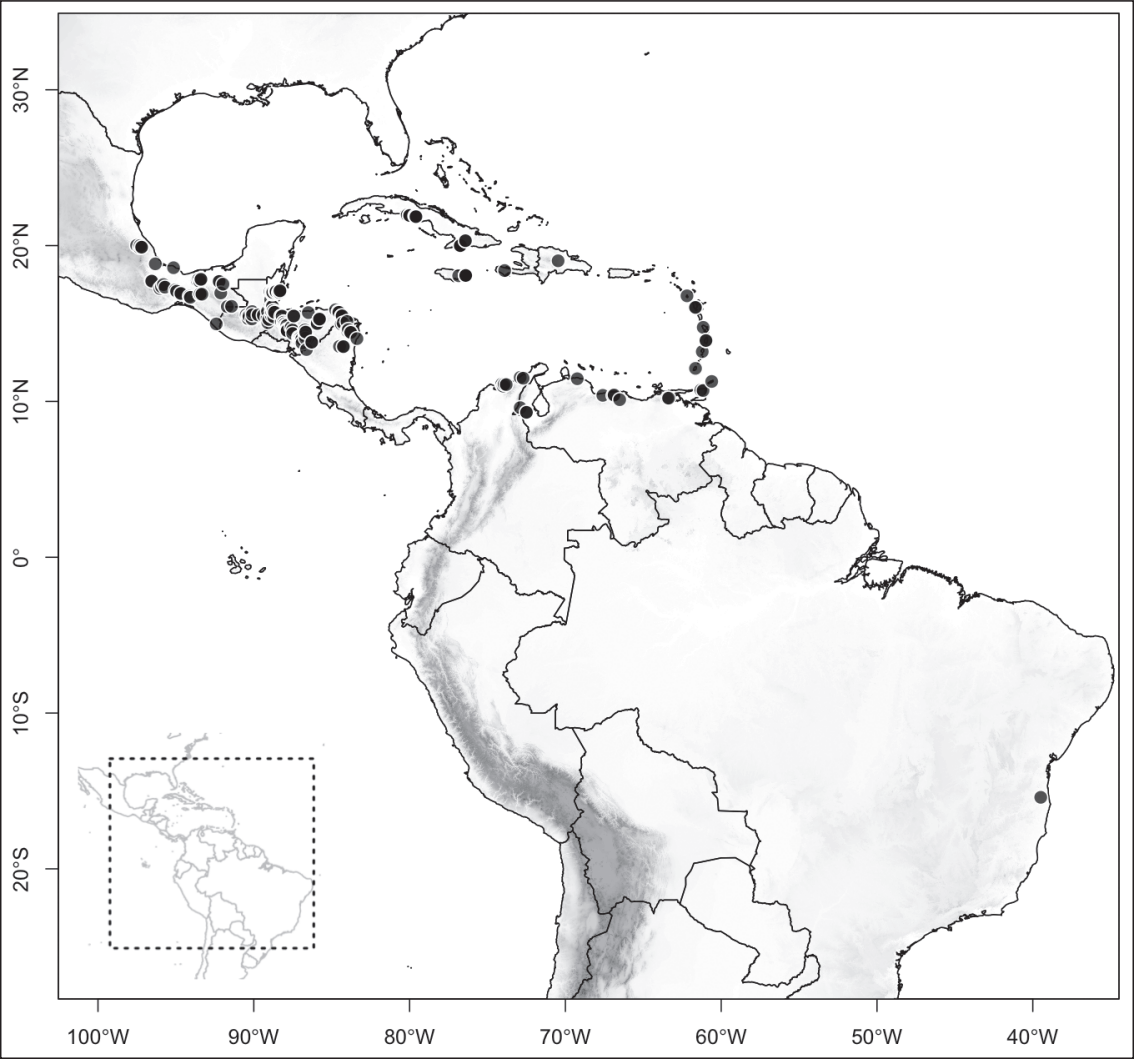
Distribution of *Conostegia icosandra*.

(Fig. 82). Ranging from Mexico throughout Central America and the Caribbean, reaching the coast of Venezuela, sea level to 1500 (-2000) m.

*Conostegia icosandra* is variable in the amount of indument and degree of dentition of the leaf margin. Despite this variation, the species has usually been circumscribed as having conspicuous persistent bracteoles (Fig. 81). These bracteoles are evident in populations mostly north of Nicaragua but in other populations of this species which were synonimized in Schnell (1996) and Almeda (2009) under *Conostegia icosandra*, the plants are glabrous, the leaves narrower and the bracteoles missing altogether. These specimens match the name *Conostegia bernoulliana* which is here considered a distinct species. In addition, the style in *Conostegia icosandra* can lack the large central hole that is evident in *Conostegia bernoulliana*. The distinction of these two taxa is relevant for understanding the evolution of crateriform stigmas since the true *Conostegia icosandra* falls as sister to the rest of the lobed stigma clade, suggesting the crateriform stigma was possibly not lost in *Conostegia icosandra* whereas it was probably lost in *Conostegia pittieri*. The specimen *Davidse*, *González and León 18607* (NY) from Zulia, Venezuela, unlike any other specimens studied has extremely narrow leaves.

#### Specimens examined.

**CUBA. Oriente**: Sierra Maestra, Pinar de Papagayo, Ekman 9273 (NY); Loma del Gato, Cobre Range, Clemente 1823, León 10054 (NY). **Santa Clara**: Trinidad Mountains, Aguacate, Britton and Wilson 5377 (NY); Southern Oriente and Pico Turquino, High Maestra, Ekman 10944 (NY); Pinar de la Caridad, Southeast of Yara, Ekman 14689 (NY); Buenos Aires, Trinidad Hills, Jack 6844 (NY); Loma Las Divisiones, Banao Mts., León 7864 (NY); Sierra de Gavilanes, Sanoti-Spiritus Mts., León and Clement 6616 (NY); Lomas de Banao, Luna 172 (NY); Sevilla Estate near Santiago, Jiquarito, Sierra Maestra, Taylor 501 (NY); Vicinity of La Sabana, Buenos Aires, Trinidad Mountains, Smith et al. 3384 (NY). Las Villas: Buenos Aires, Hills East of Cienfuegos, León and Jack 13934 (NY).

**GUADALOUPE. Basse Terre**: Mosciu district, south of La Citerne, Proctor 20129 (NY). Gran Savane, Duss 3472 (NY); along road from Bains Jaunes to Soufriere, Howard 19773 (NY); Dr. Madiana s. n. (NY); Grand Etang, Martin and Hus 488 (NY).

**HAITI. Massif de la Hotte**: Depts. Sud-Grand Anse límite, zona rural “Gerard”, 18 km Norte de Camp Perrin, en la carretera a Beaumont y Jérémie, Zanoni et al. 25696 (NY).

**JAMAICA. Portland**: East slope of the John Crow Mts. 1.5–2 miles southwest of Ecclesdown, Proctor 9975 (NY); about 1.5–2 miles southwest of Ecclesdown, Yuncker 18537 (NY); Vicinity of Moody’s Gap, Britton 3388 (NY); John Crow Mountains, Britton 4181 (NY); Woodland eastern slopes of south end of John Crow Mountains, Harris and Britton 10721 (NY). **St. Andrew**: Coopers Hill, Red Hills, Proctor 8483 (NY). **St. Thomas**: Cuna Cuna Gap, Britton 4050 (NY).

**MARTINIQUE.** Sta. Marie, Duss 917 (NY); Hauteux de Case-Pelote, Petons du Cartel, Duss 4626 (NY); along road from Morne Rouge to Mt. Pelée, Holdridge 465 (NY); Sieber 119, 466 (NY).

**MONTSERRAT.** Pond Mountain, Shafer 683 (NY).

**SAINT LUCIA.** Forest reserve between Qilesse and Morne Troumasse, Howard 11666 (NY); 1.5 miles south west of Millet on the Millet River, Slane 137 (NY).

**SAINT VINCENT.** Upper Richmond Valley, Smith 489 (NY).

**TOBAGO.** Roxborough-Parlatuvier road, 8th-9th mileports, Sandwith 1924 (NY).

**MEXICO. Chiapas**: 18–20 km north of Ocozocoautla along road to Mal Paso, Municipio of Ocozocoautla de Espinoza, Breedlove and Thorne 21011 (CAS, MO, NY); between Colonia Francisco I. Madero and Colonia A. Lopez Mateos, Breedlove 50594 (CAS, NY); Loc. a 3 km al este de Tziscao en el parque natural lagos de Montebello, Cabrera, Mendez and Cabrera 2957 (NY); Avalinero, Palenque, Matuda 3601 (NY); Finca Mexiquito, Purpus 6785 (MO, NY). **Oaxaca**: Juchitan to the east of Sarabia, MacDougall s.n. (NY); Palomares, Juchitán, MacDougall 88 (NY); Distrito Choapam, Yaveo, Mexia 9141; Sierra de Juárez, Paray 127 (NY); San Juan Teotalcingo and Santiago Choapam District of Choapam, Schultes 568 (NY); Between Monte Negro de Lalana and San Juan Lalana District of Choapam, Schultes and Ro 796 (NY). **Puebla**: Moist roadside soil near Ocostoc below Teziutlan, Sharp 45823 (NY); Atecocomo, municipio de Cuetzalan, Ventura 1115 (NY). **Tabasco**: Km. 4 de la Est. Chontalpa hacia Malpaso, carretera Huimanguillo Malpaso, Cowan et al. 2545 (NY); Km 31.9 de la desviación de Huimanguillo hacia Fco. Rueda, Cowan 3329 (MO, NY); Achotal, Balancan, Matuda 3068 (NY); Carretera Huimanguillo-Francisco Rueda, 8.5 km, Ventura 20608 (MO, NY). **Veracruz**: Minatlitlán, Mell s.n. (NY); About 6 km by air S of Tlapacoyan on road to Altotonga, Mun. Tlapacoyan, Nee and Diggs 24874 (NY); Vicinity of La Calavera 10 km N of Altotonga (13 km by road) on road to Tlapacoyan, Nee and Diggs 24891 (NY); Jaltipan, Orcutt 6515 (NY); Zacuapan, Purpus 7510 (NY).

**BELIZE. Cayo**: Kinlocks Camp Road, Balick 3345 (MO, NY); Along Hummingbird Highway south of Belmopan between 30–38 mi., Croat 24837 (MO, NY); Mountain Pine Ridge, San Agustin, Lundell 6587 (NY); Near Río On along road to Augustine, Nee et al. 46782 (MO, NY); Mountain Pine Ridge on main road to Augustine, Ratter 5168 (NY). **Stann Creek**: in broken cohune ridge near Carasow Hill, Gentle 8212 (NY); MI 32 Hummingbird HWY, Margaret Creek Village, Holmes 4613 (NY); Mullins River Road, Schipp 145 (MO, NY). **Toledo**: 1.4 km on S turnooff ”Farmer’s Road” 10km W of Punta Gorda Town, Arvigo 969 (NY); in edge in wooded island Condemn Branch Pine Ridge, Gentle 5283 (NY); ca. 3 km W of coast of Punta Gorda, Nee et al. 46934 (MO, NY).

**GUATEMALA. Alta Verapaz**: Tucuru, Finca de la Concepción, Boeke and Utzschneider 2932 (NY); Chacirociha, Finca Seaway, Hatch 193 (NY); entre San Pedro Carchá y Sacoyoú, Molina and Molina 12124 (NY); large swamp just east of Tactic, Steyermark 43988, 92629 (NY); Saquija 43 km. northeast of Cobán, Standley 70110 (NY); von Turckheim 2236 (NY). **Izabal**: 1–2 km south of Izabal, Jones 3015 (F, NY); South shore of Lake Izabal east of village of Izabal at sea level, Jones 3143 (NY); San Tomas de Castilla on coastline road to Las Pavas, Marshall et al. 332 (MO, NY); Vicinity of Quiriguá, Standley 23924 (NY). **Quiché**: Finca Chaila, “Zona Reyna”, Skutch 1809 (NY).

**HONDURAS. Comayagua**: 14 km SE of Taulabé, Davidse and Pohl 2228 (NY); Matorrales en colinas rocosas de los alrededores de La Libertad, Molina 7074 (NY); Bosque de Montania La Choca en Cordillera de Comayagua cerca de Coyocutena, Molina 8129 (NY). **Cortés**: Río Amapa on road to Lake Yojoa, Howard 587 (NY); Matorrales húmedos de Peña Blanca, nacimiento del Río Lindo, Molina 6777 (NY); bosque de pino-roble entre Cofradia y Cusuco, Molina 7294 (NY); Bosque lluvioso entre Agua Azul y Pito Solo Lago de Yojoa, Molina 7331 (NY); Montaña La Cumbre caserío las Piñitas, Molina 10522 (NY); Matorrales y bosque mixto de Cascada El Chorrito 16 kms. al S.O. de Siguatepeque, Molina 10901 (NY); Montaña San Idalfonso entre Cusuco y San Isirdo, Molina 11544 (NY); above la Misión, Molina 12341 (NY). **Yoró**: Ocotales en sabanas pedrejosas de Piedra Colorada, Molina 6886 (NY). **El Paraíso**: Montaña Teupasenti entre El Junquillo y Teupasenti, Molina 11866 (NY); Matorrales Quebrada El Coyol, Sierra El Chile entre El Junquillo y El Robledal, Molina 14158 (NY); Bosque mixto Quebrada Tapahuasca, Molina 14658 (NY). Gracias a Dios: Mocorón 60 km SO de Puerto Lempira, Rivas 131 (NY); Alrededores de Mocorón 60 km al SO de Puerto Lempira, Torres 136 (NY). **Intibucá**: Cordillera Opalaca, between Calaveras and Pela Naríz road to La Esperanza, Molina 22600 (NY). **Morazán**: entre la Pirámide y Zambrano, Molina 11030 (MO, NY); carretera a Maraita, Molina 13781 (NY); along Agua Amarilla creek 5 kms. north of El Zamorano, Molina 26269 (NY); Region of Agua Amarilla above El Zamorano, Standley et al. 5074 (NY); Agua amarilla, Williams and Molina 10142, 12156 (MO, NY). **Siguatepeque**: near El Achote, hills above the plains of Siguatepeque, Yuncker et al. 5822, 5825, 5909 (MO, NY).

**NICARAGUA. Nueva Segovia**: in gorge on Cerro Mogoton, Atwood and Neill AN15 (MO, NY); San Mateo, 16 millas al sur de Tronquera cerca de Río Wawa, Molina 15075 (MO, NY); frecuente a orillas de Río Leicus cerca del campo de aviación de Tronquera 35 kms S. O. de Waspan, Molina 15174 (MO, NY).

**BRAZIL: Bahia**: Camaça, km la 2 da estrada do lado L de Camaça, dos Santos 1340 (NY).

**COLOMBIA. Guajira**: Municipio Riohacha, Sierra Nevada de Santa Marta, sector de Cenuá, Dueñas 1779 (COL). **Magdalena**: Cincinati, Giacometto 138 (NY); along quebrada in S portion of finca Reflejo, Kirkbride 2146 (NY); Around San Andrés de la Sierra, western slope of Cordillera de Santa Marta, Pittier 1684 (NY); Plants of Santa Marta above Agua Dulce, Smith 765 (NY).

**TRINIDAD. Arima**: Cumaca Roadover Morn Croix, Adams 14331 (NY); Arima-Blanchisseuse Road, Kalloo B.1056 (NY); St. Pat’s, Arima Valley, Snow and Fleming 310 (NY); Arima Valley, Snow DS-H (NY); Blanchiseusses River, Snow DS-D (NY).

**VENEZUELA. Falcón**: Cerro Santa Ana, ascensión del lado sur desde el pueblo de Santa Ana, Steyermark and Braun 94654 (NY). **Miranda**: Los Guayabitos (arriba de Baruta), Aristeguieta 2880 (NY); Bernardi s. n. (NY). **Monagas**: Cuenca del Río Caripe al E de Caripe vía Las Margaritas, Quebrada Pajaral 3 km al E de Escuela Rural El Aguacate, 11.2 Km al E del puente sobre el Río Colorado, Michelangeli and Alford 612 (NY); P. G. Pacanilla, Parque Nacional Guatopo, Quebrada al inmediatamente al S de P.G.P Macanilla, 9 km S de Los Alpes, Michelangeli and Michelangeli 803 (NY); Montaña de Aguacate, along Quebrada de Pajarral, tributary to Río Caripe, northeast of Alto de Aguacate, between Caripe and Caripito, Steyermark 62200 (NY). **Zulia**: Distrito Colón, 3 km E of the Río de Oro settlement on the Río de Oro, Davidse et al. 18607 (NY).

### Conostegia jaliscana

Taxon classificationPlantaeMyrtalesMelastomataceae

Standl.

Conostegia jaliscana Standl., Field Mus. Publ. Bot. 4: 245. 1929. Type: Mexico. Jalisco: Streamside, Arroyo de los Hornos, Hacienda del Ototal, E of San Sebastian, Sierra Madre, 1500 m, 5 March 1927, Y. Mexía 1819 (holotype: F!, isotypes: A!, BM!, C, CAS!, GH!, LA, MICH, MO!, NY!, S!, TEX!, UC, US!).

#### Description.

Shrubs 2–4 m tall with young tetragonal stems which become terete with age that are glabrescent to finely and sparsely furfuraceous; the nodal line present. Leaves of a pair equal to unequal in length. Petioles 0.4–1.2 cm. Leaf blades 6–17 × 2–6 cm, 3–5 plinerved, with the innermost pair of veins diverging from the mid vein 1–2 cm above the base, elliptic, the base acute, the apex acute to acuminate, adaxially glabrous or sparsely ciliate, abaxially pubescent with stellate trichomes on the veins, the margins serrulate and ciliate. Inflorescence a terminal panicle 3–7 cm long branched above the base but sometimes appearing branched at the base because of multiple inflorescences arising at opposing meristems at the terminal node, accessory branches apparently absent, branches flattened; bracteoles linear to ovate, 2 mm long, early deciduous and appearing absent. Pedicels 2–5 mm. Flowers (5-)6–7 merous, calyptrate. Floral buds 10–15 × 4–7.1 mm, slightly ovoid to elliptic, subacute or rounded at the base, acute to acuminate at the apex, slightly constricted below the middle, the calyptra and base weakly differentiated; hypanthium 4.5–5.5 × 5–6 mm, glabrescent with inconspicuous sessile stellate hairs. Petals ca. 7–8 × 5–6 mm, white, broadly spatulate, probably spreading, glabrous. Stamens 15–20, 7 mm long, the filaments 3–4 mm long, white, anthers 3.5–4 mm long, linear-subulate, recurved, yellow, the pore not observed, terminal. Ovary 6–7 locular, inferior, glabrous, and elevated into a collar around the style base. Style 4–6 mm, curving and not widening below the stigma, bent beneath the tip, apparently no vertical or horizontal distance between the anthers and the stigma; stigma subcapitate, ca 1 mm wide. Mature berries not seen.

#### Distribution

**Figure 83.**
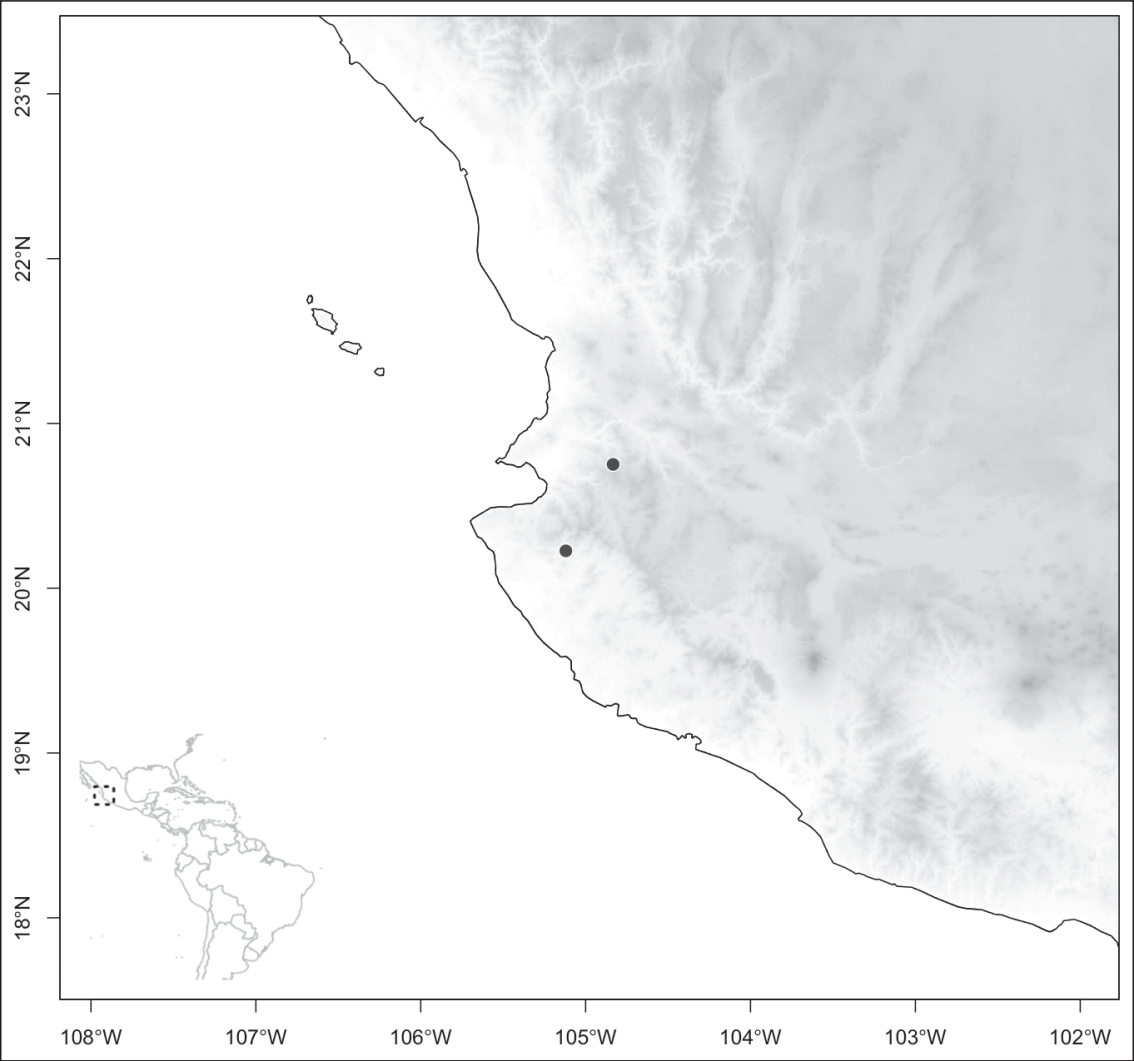
Distribution of *Conostegia jaliscana*.

(Fig. 83). Endemic to Mexico where it occurs in the Sierra Madre in the states of Jalisco and Guerrero from 700–1600 m in elevation.

*Conostegia jaliscana* is a rare species reported usually from alongside streams. It has few-flowered inflorescences and relatively long and acute to acuminate floral buds. Schnell (1996) placed *Conostegia jaliscana* with the three Jamaican endemic in his section *Conostegia* but also hinted to the possibility of *Conostegia jaliscana* being closely related to species in his subgenus *Lobatostigma*. This latter hypothesis is the one supported in the molecular phylogeny.

#### Specimens examined.

**MEXICO. Guerrero** (fide Schnell): in Sierra Madre del Sur, 20 mi S of Chilpancingo on the Acapulco Highway, Smith M73 (TEX). **Jalisco**: ca. 27.8 mi sse of Puerto Vallarta on a new dirt road to silver mine, left hand turnoff hwy 200 toward Manzanillo, Almeda 2540 (CAS).

### Conostegia lindenii

Taxon classificationPlantaeMyrtalesMelastomataceae

Cogn.

Fig. 84

Conostegia lindenii Cogn, DC. Monog. Phan. 7: 705. 1891. Type: Cuba. La Guinea, 600 m, no date, J. Linden 2204 (holotype: LE, isotype: BR!).
Conostegia lomensis Urban, Fedde Rep. Sp. Nov. 17: 161. 1921. Type. Dominican Republic. Santo Domingo: Barahona, La Loma, 1000 m, September 1911, M. Fuertes 1028 (holotype: A!).
Conostegia furfuracea Urban & Ekman, Arkiv. Bot. 23A (11): 15. 1931. Type: DOMINICAN REPUBLIC. Santo Domingo: Duarte, Cordillera Septentrional, Loma Quita-Espuela, c. 700 m, 25 April 1929, E. Ekman H12273 (holotype: S!, isotypes: A!, GH!, NY!, US!).

#### Description.

Shrubs or small trees to 6 m tall, stems tetragonal but soon terete and densely pubescent with sessile and stipitate stellate hairs; the nodal line present yet slight. Leaves of a pair equal to subequal in length. Petiole 0.8–2.5 cm long. Leaf blade 6–18 × 2–6 cm, 3–5 nerved, narrowly ovate to elliptic or ovate, acute to rounded at the base, acute to acuminate or rarely obtuse at the apex, the margins entire or obscurely denticulate, adaxially glabrous, abaxially densely covered with stellate and stipitate trichomes. Inflorescence a terminal panicle 6–12 cm long branched above the base but sometimes appearing branched at the base because of multiple inflorescences arising at opposing meristems at the terminal node, accessory branches apparently absent; bracts and bracteoles to 3 mm long, linear, early deciduous. Pedicels 2–7 mm long, covered with stellate and branching trichomes. Flowers (4-)5(-6) merous, calyptrate. Floral buds 8–9 × 3–4 mm, pyriform to lachrimiform, truncate or rounded at the base, narrowly acute and acuminate at the apex, the apex with somewhat discernible lobes, the calyptra well differentiated from the hypanthium; hypanthium 4–5 × 3.5–4 mm, campanunlate, sparsely to densely beset with stellate hairs and minute brown glands. Petals ca. 5 mm long, white, obtriangular, spreading to a little reflexed, rounded apically, glabrous. Stamens 9–14, 3–3.5 mm long, radially arranged around the style but apparently becoming zygomorphic becasuse of the style bending to one side below the stigma, the filament ca. 1.5 mm long, white, lacking a geniculation, anthers 1.5–2 mm, linear and slightly recurved, yellow, the pore ca. 0.1 mm, terminal. Ovary 5–7 locular, inferior, apically glabrous and forming a low collar around the style base. Style 2–3 mm long, bent below the stigma, vertical and horizontal distance from the stigma to the anther absent; stigma truncate, ca. 1 mm wide. Berry 5 × 5 mm, blue black. Seeds 0.4–0.5 mm, ovoid, smooth.

**Figure 84.**
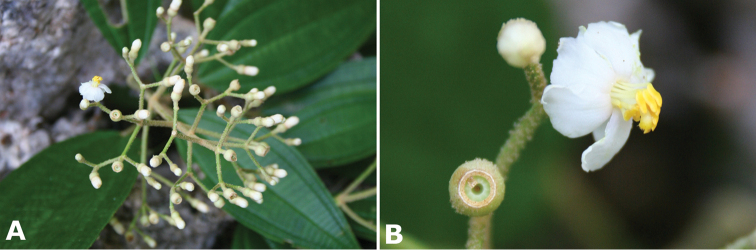
*Conostegia lindenii*. **A** Inflorescence **B** Close up of flower. Photos by Eldis Bécquer.

#### Distribution

**Figure 85.**
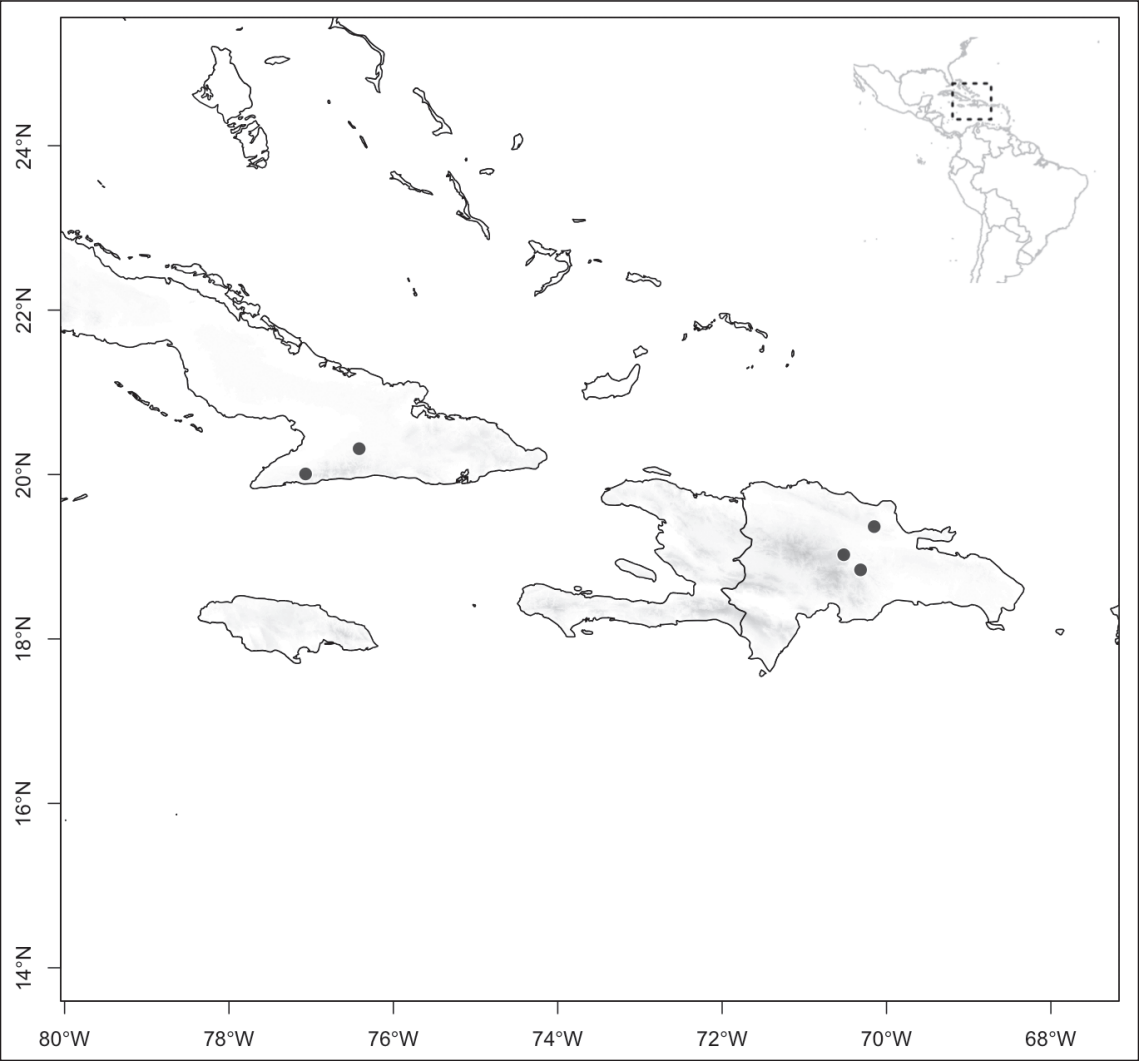
Distribution of *Conostegia lindenii*.

(Fig. 85). Cuba and the Dominican Republic, 750–1300 m elevation.

Note that in Dominican Republic a pubescent morphotype of *Conostegia superba* has been collected in the same place. They are similar but when looking at the flower buds, *Conostegia lindenii* has pubescent buds with the lobes somewhat evident in the calyptra apex. In *Conostegia superba* on the other hand, the flower bud and calyptra are glabrous and the lobes not discernible at all.

#### Specimens examined.

**CUBA. Oriente**: Loma del Gato and vicinity Cobre Range of Sierra Maestra, Edmond 84 (NY); Sierra Maestra, El Gigante on the high ridge, on Río Guisa, Ekman 16084 (NY).

**DOMINICAN REPUBLIC. Monseñor Nouel**: Road up to Alto Casabito ca. 8 km W of jct. with Highway Duarte on road from Bonao to Constanza, Judd et al. 6521 (NY); Firme de Banilejo Piedra Blanca, Liogier and Liogier 19940 (NY).

### Conostegia macrantha

Taxon classificationPlantaeMyrtalesMelastomataceae

O. Berg ex Triana

Fig. 86

Conostegia macrantha O. Berg ex Triana, Trans. Linn. Soc. London 28: 97. 1872. Type: Costa Rica. San José: Candelaria, no date, A. Oersted 12 (lectotype: BR!, designated here; isolectotypes (fide Schnell (1996): C, GH, as well as a photo of a lost specimen from B at F!).

#### Description.

Trees 3.5–15 m tall with thick tetragonal and ridged stems that are glabrous or furfuraceous on new growth with sessile stellate trichomes; the nodal line conspicuous and sometimes setulose in young branches, lenticellate abaxially. Petioles 1–7 cm long. Leaves of a pair equal to somewhat unequal in length. Leaf blades 6.7–30 × 2.3–15 cm, 5 nerved or slightly plinerved, ovate to elliptic, the base acute to obtuse, the apex obtuse and acute to acuminate, the margin entire or denticulate, adaxially glabrous, abaxially with branched and stellate hairs on the veins. Inflorescence a terminal panicle 6–21.2 cm long branched above the base but sometimes appearing branched at the base because of multiple inflorescences arising at opposing meristems at the terminal node, accessory branches present, rachis tetragonal, accessory branches present, bracts early deciduous or absent, the rachis glabrous or furfuraceous with sessile stellate trichomes these sometimes minute and inconspicuous, bracteoles linear, ca. 2 mm long, deciduous. Pedicels 4–15 mm, frequently nodding. Flowers 7–10 merous, calyptrate. Floral buds 8–15 × 7–13.5 mm, spherical, the base rounded, the apex rounded and mucronate, not constricted, the hypanthial and calycine portions little differentiated; hypanthium 9.5–10 × 13–14 mm, glabrous or beset with small sessile stellate trichomes, strongly tuberculate. Petals 9.5–16 × 12–14.5 mm, white, obovate, spreading, the apex retuse, glabrous. Stamens 28–45, 9–11 mm long, radially arranged around the style, occasionally secondarily zygomorphic resulting from some stamen getting stuck below the stigma, the filament 5.25–5.7 mm, white, anthers 4.5–5 × 1.75–2.25 mm, oval, yellow, the base sagittate, strongly laterally compressed, the pore 0.2–0.3 mm, terminal. Ovary 18–25 locular, inferior, the apex glabrous and forming a collar around the style; style 7–8 mm long, usually straight but sometimes slightly curving, distance from the anther to the stigma ca. -2 – 0 mm, stigma crateriform, consisting of 18–25 laterally compressed lobes, 6.5–8 mm wide. Berry 14–18 × 10–12 mm, purple. Seeds 0.5–1 mm long, narrowly ellipsoid, the testa smooth.

**Figure 86.**
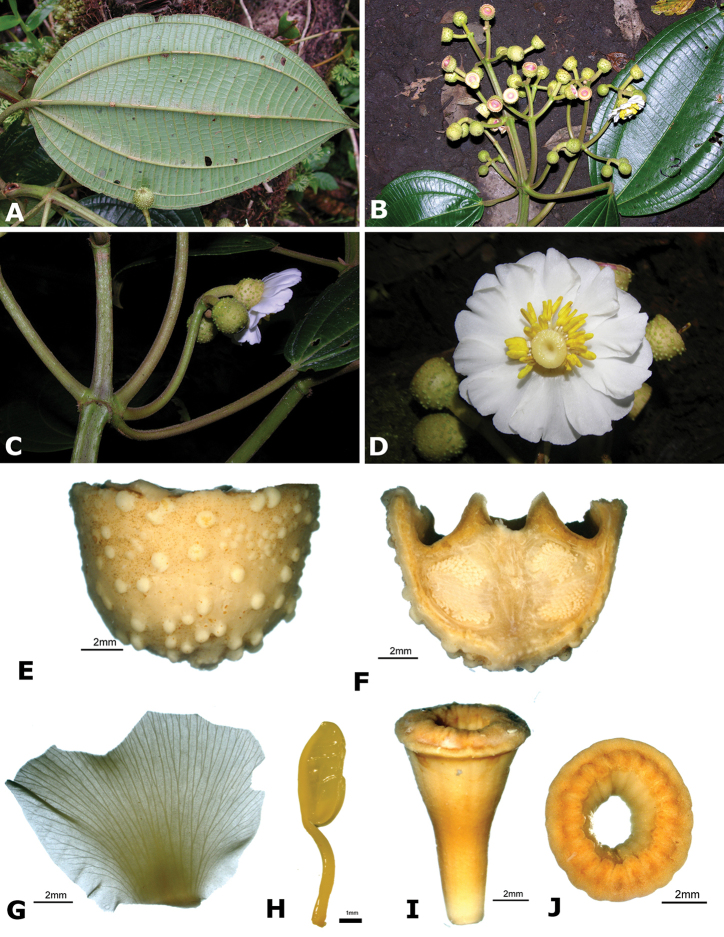
*Conostegia macrantha*. **A** Leaf abaxial foliar surface **B** Inflorescence **C** Lateral view of flower at anthesis **D** Frontal view of flower at anthesis **E** Close up of the surface of the hypanthium **F** Longitudinal section of a hypanthium of a flower at anthesis with its parts removed **G** Petal **H** Stamen **I** Style **J** Stigma. Photos of specimen vouchered *R. Kriebel 5406*.

#### Distribution

**Figure 87.**
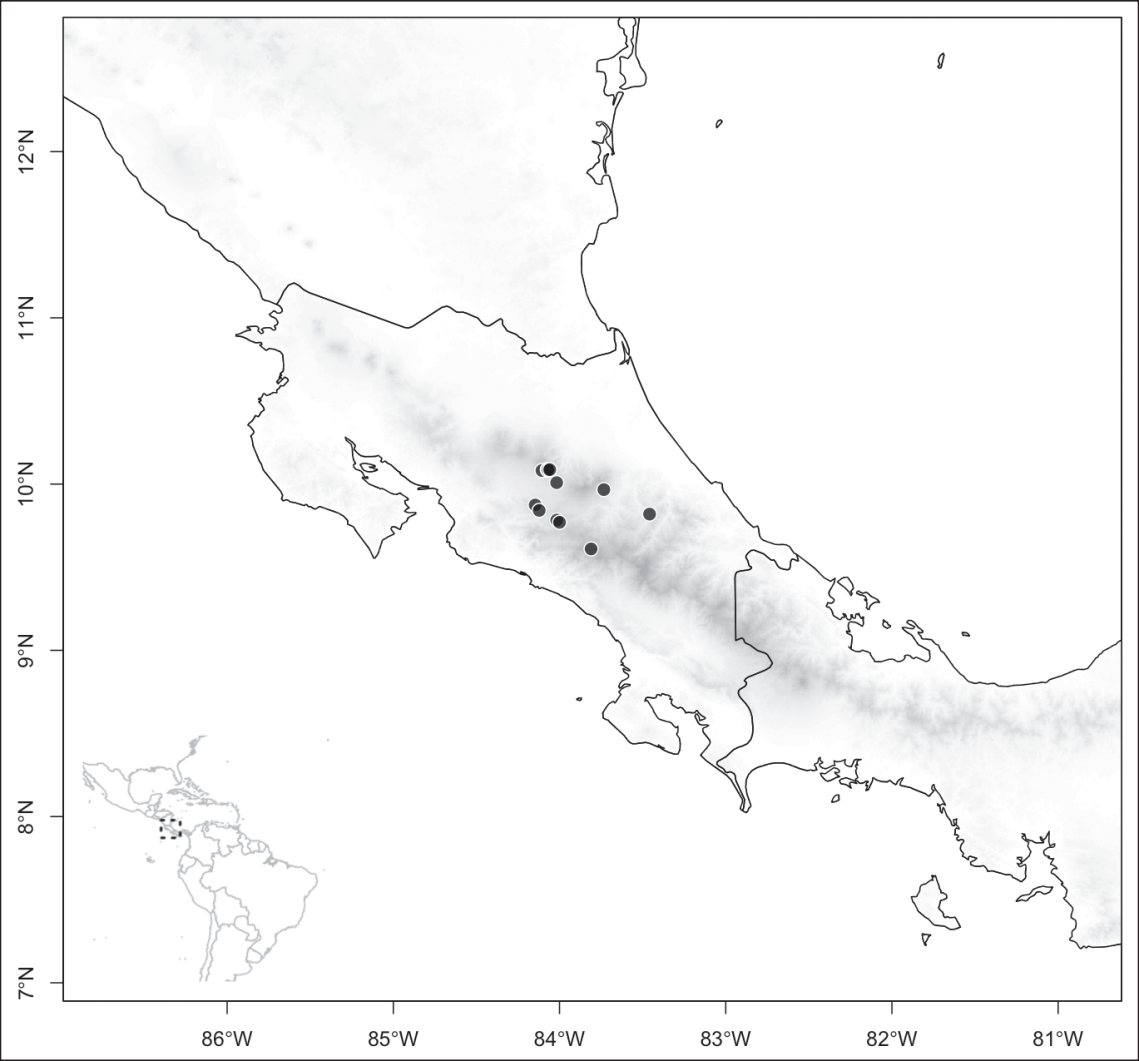
Distribution of *Conostegia macrantha*.

(Fig. 87). Endemic to Costa Rica, 1300–3000 m elevation.

*Conostegia macrantha* can be recognized by its stout branches, stellate indument on abaxial leaf surface, and large flowers with crateriform, straight styles, and retuse petals. Flowers of this species have a good fragrance (i.e., *Chavarría 817*, *Jiménez 626*-both at MO). This species is also reported from northern Panama by Schnell (1996) but I have not seen any Panamanian specimens. The few specimens from Panama that resemble *Conostegia macrantha* are glabrous, the reason why I did not include them in the distribution of this species presented here. Schnell (1996) noted that the species is especially common in the volcanoes surrounding the central valley of Costa Rica and is mostly restricted to that area. Specimens collected near Barva and Poas volcano for this study have the typical straight styles of *Conostegia macrantha*. Schnell (1996) noted some specimens from Vara Blanca showed evidence of introgression from *Conostegia oerstediana* in their smaller flowers.

#### Specimens examined.

**COSTA RICA. Alajuela**: Near Continental Divide, vicinity of Vara Blanca, Chrysler 5348 (NY); Alfaro Ruiz hills above laguna, Smith 10075 (NY). **Cartago**: Volcán Turrialba on road between Lechería La Central and La Trinidad, Schnell 1077 (MO, NY); south slope of Turrialba Volcano above Santa Cruz, Williams 19677 (NY); near La Sierra, about 25 km south of Cartago, Cordillera de Talamanca, Williams et al. 28022 (NY). **Heredia**: Sacramento, Finca Ingrid Steinvorth, Kriebel 5406 (INB, NY); near Río Las Vueltas N.E. of Volcán Barba, Lent 2644 (CR, NY). **San José**: San Cristóbal Norte, Desamparados, Antonio 706 (CR, NY); Property of Hacienda Forestales above Cascajal, Lumer 1301 (NY); NE of Coronado, Chrysler 5348 (NY); Riviera de los Arcángeles, Escazú, Pittier 13053 (NY).

### Conostegia micrantha

Taxon classificationPlantaeMyrtalesMelastomataceae

Standl.

Fig. 88

Conostegia micrantha Standl., Field Mus. Nat. Hist, Bot. Series 4: 246. 1929. Type: Panama. Bocas del Toro: Buena Vista Camp on Chiriquí Trail, Almirante, 400 m, January–March 1928, G. Cooper 578 (holotype F!, isotype NY!).

#### Description.

Shrubs to trees 1.5–10 m tall with tetragonal to terete stem which are densely tomentose with sessile stellate hairs; the nodal line present yet slight. Leaves of a pair equal to subequal in length. Petioles 0.5–6 cm long. Leaves 5.7–21.5 × 2–10.5 cm, 3–5 nerved or slightly plinerved, ovate-elliptic to ovate, the base acute to obtuse, the apex acute to acuminate, the margins entire or remotely denticulate, the adaxial surface glabrous, the abaxial surface densely tomentose with sessile stellate hairs. Inflorescence a terminal panicle 5–12.5 cm long branched above the base but sometimes appearing branched at the base because of multiple inflorescences arising at opposing meristems at the terminal node, accessory branches present or absent; bracteoles to 3 mm long, linear, early deciduous. Pedicels 0.5–3 mm long. Flowers (4-)5(-6) merous, calyptrate. Floral buds 2.5–6.5 × 1.5–3.5 mm, obovoid pyriform, the base rounded, the apex rounded to acute or or short apiculate, slightly constricted below the calyptra; the hypanthium 3–3.25 × 2.35–2.85 mm, with scattered stellate trichomes. Petals 3.5–5.25 × 2.5–3 mm, totally white with or white with pink or violet bases, oblong or broadly ovate, spreading to somewhat reflexed, glabrous, entire. Stamens mostly 12–18, 3.5–5.5 mm long, slightly zygomorphic, the filament 1.5–2.5 mm, white, 1.8–2.5 × 0.5–0.75 mm, linear to slightly sinuous, cream to yellow, the pore ca. 0.1 mm, subterminal and slightly ventrally inclined. Ovary 5–7 locular, inferior, glabrous, lacking an elevated collar around the style base. Style 3–4 mm, straight but bending slightly towards the apex, vertical distance between the anther and the stigma ca. -0.5 mm, horizontal distance absent; stigma capitellate, 1–1.5 mm wide. Berry 5–6 × 5–6 mm, purple black. Seeds 0.3–0.6 mm long, pyramidal, the testa smooth to slightly foveolate.

**Figure 88.**
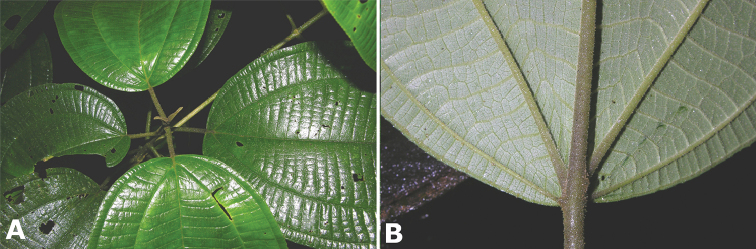
*Conostegia micrantha*. **A** Branch apex **B** Abaxial view of leaf base. Photos of specimen vouchered *R. Kriebel 5553*.

#### Distribution

**Figure 89.**
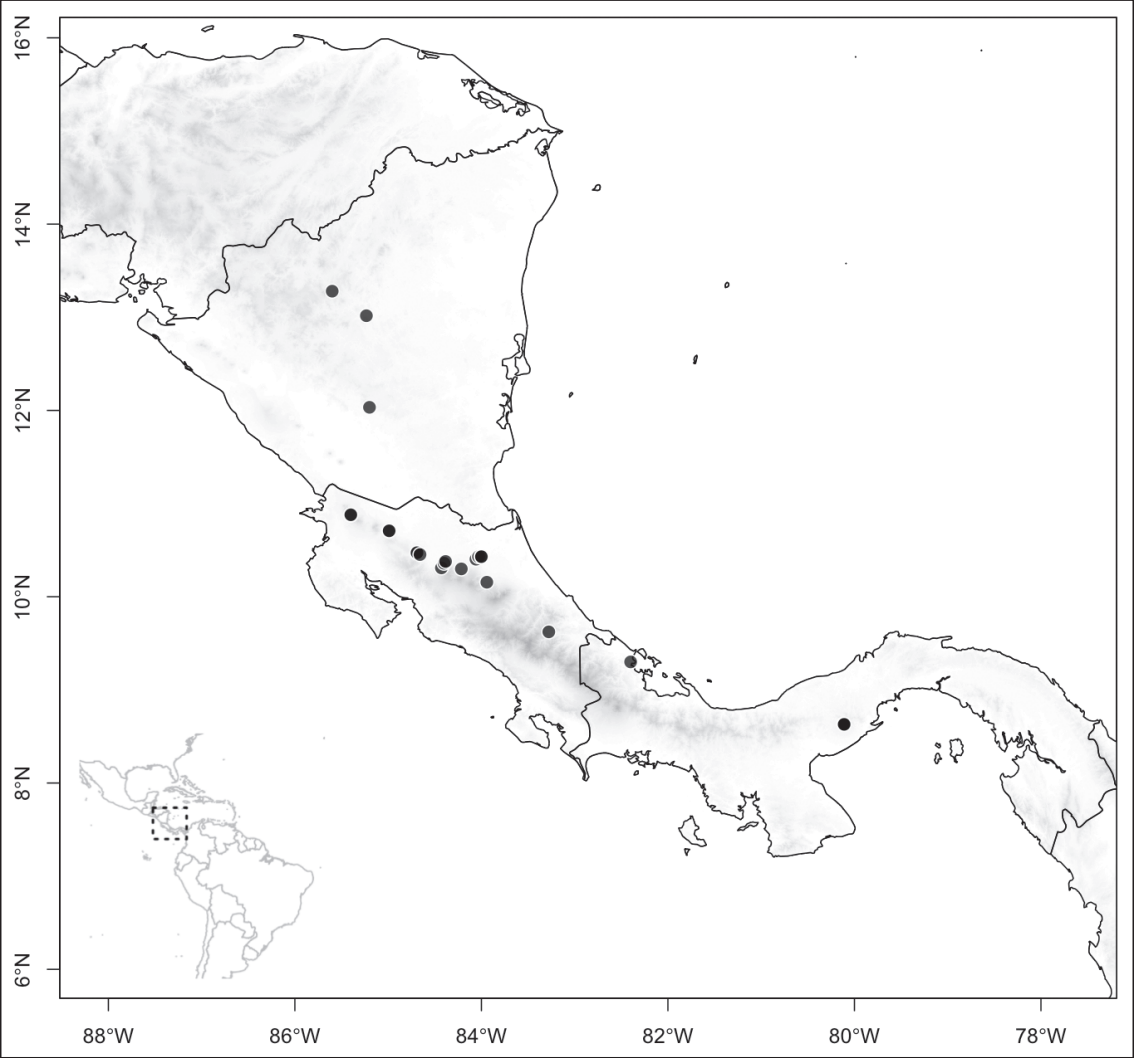
Distribution of *Conostegia micrantha*.

(Fig. 89). Nicaragua to Panama and in Ecuador, 100–1200 m elevation.

*Conostegia micrantha* is very similar to *Conostegia montana* from which it can de distinguished on the basis of the dense indument of stellate trichomes on the stem apices, the abaxial surface of the leaves, and the inflorescence. Some populations of *Conostegia montana* on the Caribbean islands as well as in Central American highlands can have stellate trichomes, complicating their distinction. When sympatric, such as in La Selva Biological Station in Costa Rica, *Conostegia montana* is glabrous and has narrower leaves. Schnell (1996) discussed the possibility that this species is derived from *Conostegia montana*. This is indeed a possibility supported also by the molecular phylogeny where *Conostegia micrantha* falls in a clade of several specimens of *Conostegia montana*. The question does remain as to whether *Conostegia montana* should be considered a single species.

#### Specimens examined.

**NICARAGUA. Río San Juan**: in tall forest near Río San Juan at ‘‘El Relos” ca. midpoint between El Castillo and Delta de San Juan, Bunting and Licht 771 (NY). **Jinotega**: Cordillera Isabelia near Río Bote Comarca de Bocaycito 117 km from Matagalpa, Neill 7170 (NY).

**COSTA RICA. Alajuela**: Cataratas de San Ramón, Brenes 13654 (CR, NY); Forested stream edge and cleared slopes below the Methodist Rural Center Quebrada Marín, About 7 km east of Ciudad Quesada, Burger and Stolze 4990 (CR, F, NY); Upala, San Cristobal, Sendero toma de agua, Espinoza 1739 (INB, NY); R. V. S. Bosque Alegre, Laguna Hule, Kriebel and Larraguivel 694 (INB, NY); Woods N.E. base of Arenal Volcano 5 km W of Fortuna San Carlos, Lent 2524 (NY); San Carlos, Fortuna, R. B. Arenal Mundo Aventura, Rodríguez 8715 (INB, NY); Bijagua, Parque Nacional Volcán Tenorio, estación Pilón, Santamaría and Azofeifa 1104 (INB, NY); Villa Quesada, San Carlos, Smith 2508 (CR, NY); P. N. Tenorio, Sector Pilón, Río Celeste, Desviación hacia la catarata, Vargas and Villalobos 1243 (INB, NY); Near Artezalea and Methodist Rural Center about 8 km N.E. of Villa Quesada, Williams 17200 (NY). **Cartago**: entre Pavones y Chitaria, Turrialba, Jiménez 734 (CR, NY). **Heredia**: Finca La Selva, the OTS Field Station on the Río Puerto Viejo just E of its junction with the Río Sarapiquí, Southern boundary, SW corner, Folsom 9525 (INB, NY); Cantón de Sarapiquí, entre Bijagual y Magsasay, Bosque ripario sobre el río Mojón, Rodríguez et al. 3110 (INB, NY). **Limón**: Pococí, P.N. Braulio Carrillo, Estación Quebrada González, entre sendero El Ceibo y sendero Botarrama, Rodríguez et al. 5076 (INB, NY); Talamanca, Bratsi ca. 1 km NW de Laguna Dabagri, Rodríguez et al. 11296 (INB, NY).

**PANAMA. Bocas del Toro**: Región of Almirante, Buena Vista camp on Chiriquí trail, Cooper 619 (NY). **Coclé**: n. side Gaital above El Valle, Darwin 2780 (NY); El Valle de Antón, Trailside woods near La Mesa about 5 miles north of El Valle, Wilbur and Luteyn 11721 (NY).

**ECUADOR** (fide Schnell). **Esmeraldas**: near Río Palavi Awá encampment, Hoover et al. 3741 (MO). **Los Ríos or Pichincha**: Montañas de Ila, on road from Patricia Pilar to 24 de Mayo, Dodson et al. 8673 (MO).

### Conostegia montana

Taxon classificationPlantaeMyrtalesMelastomataceae

(Swartz) D. Don ex DC

Figs 90 , 91

Conostegia montana (Swartz) D. Don ex DC, Prodr. 3: 175. 1828. Melastoma montana Swartz, Prodr. Veg. Ind. Occ. 69. 1788. Type: Jamaica. no date, O. Swartz s.n. (holotype: S!)
Melastoma calyptrata Desr. in Lam. Encycl. Meth. Bot. 4: 51. 1797. Type: Antilles, 177 in herb. Surian (v.s. Apud. D. de Jussieu) (not seen: Cogniaux’s monograph cites a Richard specimen at P, apparently the type, of which there is a photograph in IJ; the latter has served to authenticate the name).
Conostegia alpina Macfad., Fl. Jamaica 2: 72. 1850; nom. inval.
Conostegia calyptrata (Desr.) DC, Prodr. 3: 174. 1828.
Conostegia cooperi Cogn, DC. Mon. Phan. 7: 705. 1891. Type: Costa Rica: Cartago: 1500 m, 1888, J. Cooper 290 (distributed by Donnell Smith as 5740) (holotype: BR; isotypes: A, CAS!, F, GH!, K!, M!, US!).
Conostegia petiolata Gleason, Brittonia 1: 184. 1932. Type: British Guiana (= Guyana). Demerara, no date, Parker s.n. (holotype: K!).
Conostegia multiflora Gleason, Bull. Torrey Bot. Club 66: 416. 1939. Type: Ecuador. Esmeraldas: Playa Rica, Parroquia de Concepción, 105 m, 7 December 1936, Y. Mexía 8409 (holotype: NY!; isotypes: BM, F!, GH, K!, MO, NY!, S!, UC!, US!).

#### Description.

Shrubs to small trees 2–11 m tall with apically tetragonal stems that are glabrous or pubescent with stellate hairs or occasionally furfuraceous with sessile and stipitate stellate hairs; the nodal line present yet slight. Leaves of a pair equal to unequal in length. Petioles 0.3–6.7 cm long, sometimes bearing to small tubercles abaxially at the lamina/petiole junction. Leaf blades 3.8–21.3 × 1.2–9.6 cm, 3–5 nerved or slightly plinerved, narrowly ovate to ovate, elliptic or obovate, glabrous adaxially, glabrous or with some scattered stellulate hairs abaxially. The base acute to rounded, the apex acute to acuminate, the margin entire to serrate. Inflorescence a terminal panicle 2.7–18.1 cm long branched above the base but sometimes appearing branched at the base because of multiple inflorescences arising at opposing meristems at the terminal node, rarely a spike, accessory branches infrequent, the rachis glabrous or with some stellulate hairs, sometimes strongly flattened, subulate, lanceolate or slightly ovate, bracteoles 0.5–1 × 0.25–0.5 mm, early deciduous or persistent. Flowers sessile or more commonly with pedicels to 1mm long. Flowers (4-)5(-7) merous, calyptrate. Flower buds 4–7(-10) × 2.25–4.3 mm, variable in shape, mostly obovoid to ellipsoid, the base rounded, the apex acute to apiculate, slightly constricted below the calyptra; the hypanthium 2.85–3.25 × 4–4.5 mm, glabrous or with few scattered stellate trichomes. Petals 4.5–5.25 × 4–4.25 mm, white to pink or lilac, oblong or obovate, spreading to somewhat reflexed, the apex rounded to emarginate, glabrous. Stamens (9-)12–16(-19), slightly zygomorphic, the filament 2.5–3.5 mm, white, 1.5–2.5 × 0.5–0.75 mm, yellow, the pore 0.1–0.25 mm wide. Ovary (4-)5–7(-8) locular, inferior, the apex glabrous and elevated into a collar around the style. Style 3.25–3.75 mm, straight and curving towards the apex, vertical distance between the anther and the stigma -2 – -0.5 mm, horizontal distance absent, stigma truncate to capitellate, 1.3–1.6 mm wide. Berry 4.5–6 × 4.5–6 mm, purple black. Seeds 0.6 mm, pyramidal, smooth.

**Figure 90.**
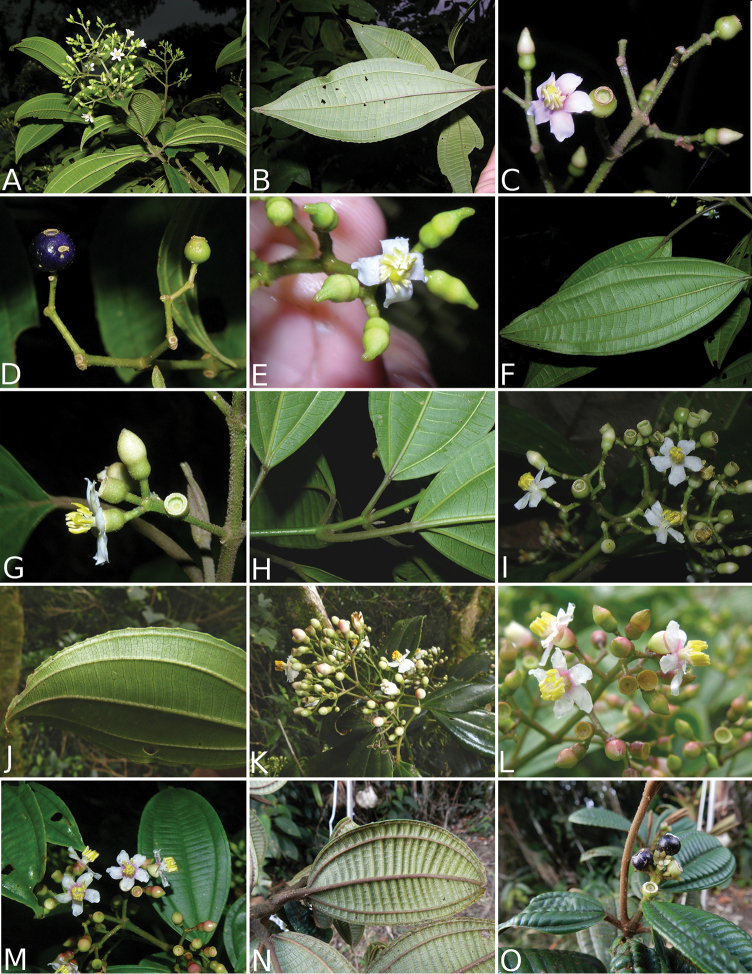
Morphological variation in *Conostegia montana*. **A** Fertile branch **B** Abaxial leaf surface **C** Flower and floral buds **D** Fruit **E** Flower and floral buds **F** Abaxial leaf surface **G** Flower and floral buds **H** Branch showing leaf bases (note tubercles towards the apex of the petioles) **I** Inflorescence **J** Abaxial leaf surface (note serrate margin) **K** Inflorescence **L** Flower and floral buds **M** Inflorescence **N** Leaf abaxial surface (note coriaceous leaves) **O** Infructescence **A–D** from specimen vouchered *R. Kriebel 5446* **E** from specimen vouchered *R. Kriebel 5544* **F–G** from specimen vouchered *R. Kriebel 5446* **H–I** from specimen vouchered *R. Kriebel 5593* **J–K** from specimen vouchered *R. Kriebel 5751* **L–M** from specimen vouchered *R. Kriebel 4895* **N–O** from specimen vouchered *R. Kriebel 5662*.

**Figure 91.**
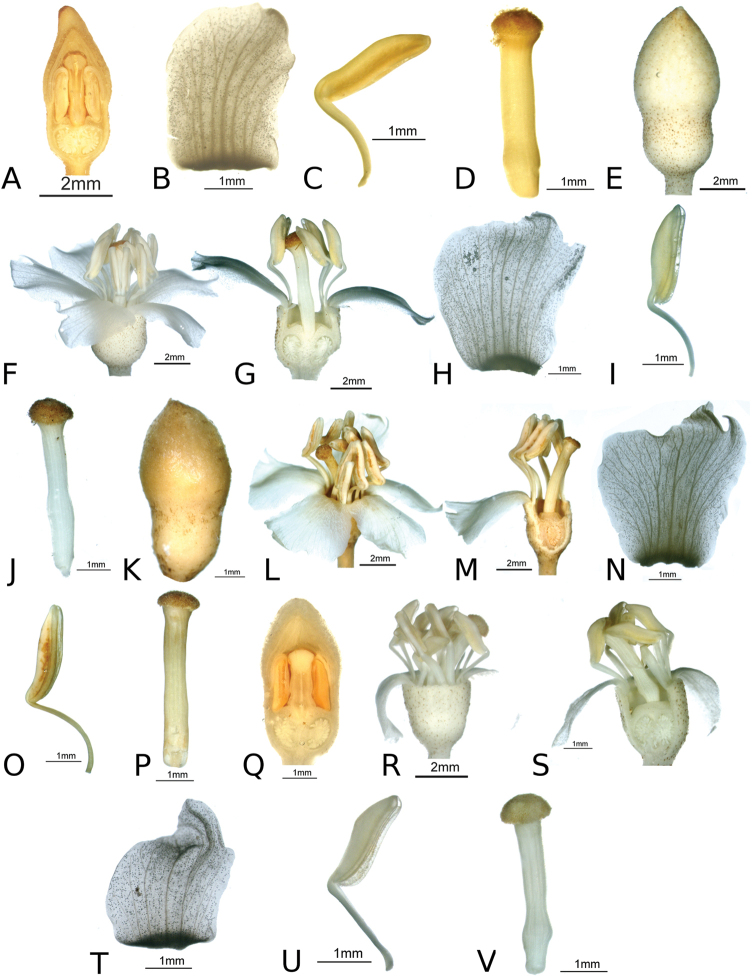
*Conostegia montana*. **A** Longitudinal cut of a flower bud **B** Petal **C** Stamen, lateral view **D** Style **E** Flower bud **F** Flower **G** Longitudinal cut of a flower **H** Petal **I** Stamen, lateral view **J** Style **K** Flower bud **L** Flower **M** Longitudinal cut of a flower **N** Petal **O** Stamen, lateral view **P** Style **Q** Longitudinal cut of a flower bud **R** Flower **S** Longitudinal cut of a flower **T** Petal **U** Stamen, lateral view **V** Style. **A–D** from specimen vouchered *R. Kriebel 5544* **E–J** from specimen vouchered *R. Kriebel 5568*. **K–P** from specimen vouchered **R** *Kriebel 5751* from specimen vouchered *R. Kriebel 5544* **Q–V** from specimen vouchered *R. Kriebel 5593*.

#### Distribution

**Figure 92.**
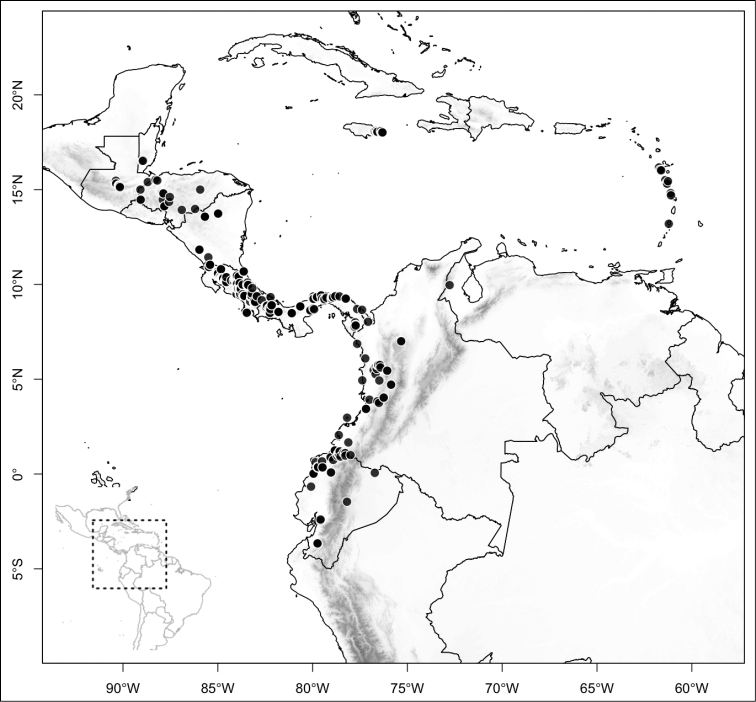
Distribution of *Conostegia montana*.

(Fig. 92). Lesser Antilles, Jamaica, in Mesoamerica from Mexico (Chiapas) to Panama (except Belize and El Salvador), western Colombia, northern Ecuador, and coastal Venezuela, at 0–2100 m in elevation.

Among the variation of this species there are populations in the mountains of Guatemala and Costa Rica that have two small knob-like structures at the apex of the petioles. In general this species tends to have some pubescence but never as dense and with as well defined stellate trichomes as typical *Conostegia micrantha*. Other populations such as the one at La Selva Biological Station in Costa Rica are glabrous and symmpatric with typical *Conostegia micrantha*. Other variants include leaves with conspicuously serrate margins such as a population in Cerro Hornito in Panama, and one population in Cerro Jefe, also in Panama, has very coriaceous leaves. For similarities to *Conostegia vulcanicola*, see discussion under the latter. The molecular phylogeny (Kriebel et al. 2015) including several specimens of different phenotypes of *Conostegia montana* from Guatemala, Costa Rica, Panama, and Jamaica revealed a clade with good support which included all the latter accessions, but also included *Conostegia micrantha* and *Conostegia setosa* nested within them. This suggests *Conostegia montana* is actually paraphyletic indicating this species complex is in need of more work. One species that is recognized in this revision and which Schnell (1996) considered a synonym of *Conostegia montana* is *Conostegia fragrantissima*. For a discussion of the differences between both, see the discussion under the latter species. Schnell (1996) clarified the precedence of the Swartz name *Melastoma montana* over *Melastoma calyptrata*.

#### Specimens examined.

**DOMINICA. Roseau**: Laudat, Beard 1467 (NY); Near the Fresh Water Lake on southeast side of Morne Micotrin and along old road on side of the mountain 1–1/2 miles east of Laudat, Chambers 2563 (MO NY); Castle Bruce Road and Trace, Cowan 1622 (NY); Lloyd 188 (NY); **St. John.** Morne Diablotin on NW ridge, Webster 13325 (MO, NY); Sylvania, Rainforest bordering Imperial Road, Hodge 3972 (NY); Proctor 95 (NY); Lower slopes of Morne Plat Pays above Bellevue along trail to Grand Bay, Wilbur et al. 7879 (NY).

**GUADALOUPE. Basse Terre**: Grand Etang, Barrier 2404 (NY); loc. cit., Martin and Gus 489 (NY); Forest above Bains Jaunes, Howard 19386 (NY); Forest near les Mamelles, Howard 19778 (NY).

**JAMAICA. Portland**: Hillside Cura Cura Gap, Britton 3527 (NY); North side of Cuna Cuna Pass, Harris 10652 (NY); 0.5 mile north of Hardwar Gap, Port Royal Mts, Proctor 9346 (NY). **St. Andrew**: Blue Mountains, along the road from Newcastle to Catherine’s Peak, Skean and Slantis 1872 (MO, NY); Blue Mountains, Hollywell Gardens, along the ”Waterfall Trail” originating at the rental cabins, Skean and Slantis 1903 (NY). **St. George**: Vicinity of Cinchona, between Moodee’s Gap and Vinegar Hill, Britton 163, 221 (NY); Below Vinegar Hill, Harris 6336 (NY). **St. Thomas**: Saint Thomas and Portland Parish boundary, Blue Mountains, Trail from just below Blue Mountain Peak (Middle Peak) to Portland Gap, Judd 5288 (NY); Portland Gap, Proctor 8204, 9597 (NY); West slope of Blue Mt. Peak, Proctor 9443 (NY); West slopes of Blue Mountain Peak between Portland Gap and the top, Skean and Slantis 1919 (NY).

**MARTINIQUE. Saint-Pierre**: Near L’Alma, Bailey and Bailey 284 (NY); Valle du Carbet St. Denis, Duss 108924 (NY).

**ST. VINCENT.** Valley of north fork of Cumberland River, Morton 5519 (MO, NY); Smith and Smith 994 (NY).

**MEXICO** (fide Schnell). **Chiapas**: km 18 Col. Cuahutemoc trinitaria Chiapas, Shilom Ton 8196 (MO).

**GUATEMALA. Alta Verapaz**: Wet forest near Tactic above the bridge across Río Frío, Standley 90312 (NY); Large swamp east of Tactic, Standley 92640 (NY). **Baja Verapaz**: Entrada al Biotopo Universitario para la Conservación del Quetzal, Kriebel et al. 5568 (NY, USCG); Biotopo del Quetzal WNW of Purulhá, Stevens et al. 25434 (MO, NY). **Izabal**: Sierra Caral, sendero al sur de la casa de investigadores hacia la cima de la Sierra Caral, mitad del camino, Kriebel et al. 5593 (NY, USCG).

**HONDURAS. Comayagua**: Bosque de pino-liquidambar de Montaña El Cedral Cordillera Montecillos, Molina 7202 (NY). **Cortés**: frecuente en el bosque nebuloso de Cusuco, Montaña Idalfonso, norte de Cofradía, Molina 7256, 8252 (NY). **Intibucá**: Bosque abierto de El Cedral 20 kms al suroeste de Siguatepeque camino a Jesus de Otoro, Molina 6150 (NY). **La Paz**: Cut over cloud forest of Montaña Verde on Cordillera Guajiquiro, Molina 24374 (NY). **Ocotepeque**: a 35 km al NE de Nvo Ocotepeque camino a San Pedro Sula, Martínez and Téllez 12986 (MO, NY).

**COSTA RICA. Alajuela**: La Palma de San Ramón, Brenes 5577, 5647, 6282 (CR, NY); Guatuso, P.N. Volcán Tenorio, Cuenca del Río Frío, Alto Masís, Chaves and Muñoz 335 (INB, NY); Finca La Paz, San Ramón, Kriebel 1476 (INB, NY); Parque Nacional Volcán Tenorio, Estación El Pilón, Sendero hacia Cerro Montezuma, Kriebel et al. 5496 (INB, NY); Vara Blanca de Sarapiquí, north slope of Central Cordillera, Skutch 3314 (NY). **Cartago**: approximately 2 km E of Peñas Blancas bordering the Río Naranjo, Almeda and Nakai 3958 (CR, NY); P.N. Tapantí, alrededores de la estación, Kriebel 5352 (INB, NY); Kiri Lodge sendero a la catarata, Kriebel et al. 5466 (INB, NY); south slope of Turrialba Volcano above Santa Cruz, Williams 19662 (NY). Heredia: Z. P. La Selva, camino al lindero sur, Kriebel et al. 3984 (INB, NY). **Limón**: Parque Internacional La Amistad, Camp 2, ridge above camp, Monro and Santamaria 5540 (INB, NY); Talamanca, P.I. La Amistad, colectando en transecto 2 y alrededores, Solano 4165 (INB, NY); Valle de la Estrella, Fila Matama, Cerca de 11 km SW del pueblo de Aguas Zarcas, Sitio Helechal, Solano et al. 4759 (INB, NY). **Puntarenas**: Monteverde cloud forest reserve, Lumer 1025, 1101 (CR, NY); Buenos Aires Sabanas Esperanza y Bosques Aledaños, Santamaría et al. 4407 (INB, NY). **San José**: Pérez Zeledón, Río Nuevo, Savegre Abajo, Finca de Julio Mena, Estrada et al. 2194 (CR, NY); Río Quebradas a orillas de la carretera Interamericana camino a Pérez Zeledón, Kriebel and Hammel 3338 (INB, NY).

**PANAMA. Bocas del Toro**: Robalo Trail Northern slopes of Cerro Horqueta, Allen 4942, 5006 (NY); along road to Chiriquí Grande c. 10 road miles from continental divide and about 2 miles along road east of highway, McPherson 10834 (MO, NY). **Canal Zone**: Militar Reserva Fuerta Sherman and adjacent canal zone, road S-1 between Gatun and Pina, Liesner 1360 (MO, NY); Premontane wet forest along road S1 5.5 km W of Gatun Dam, Nee and Hale 9692 (NY). **Chiriquí**: Quebrada Velo Vcty. Finca Lerida, Allen 4684 (NY); heavily forested slope above the Río Caldera beyond Bajo Mono in the vecinity of Boquete, Wilbur et al. 11075 (MO, NY). **Colón**: Carretera hacia el poblado de Piñas, Galdames and Guillén 3317, 3320 (MO, NY); Santa Rita Ridge 2-3 miles from Transisthmian Hwy, Gentry 1860 (NY). **Darién**: Coasi-Cana trail on Cerro Campamento E. of Tres Bocas headwater of Río Coasi, Kirkbride and Duke 1253 (MO, NY). **Panamá**: between peaks of Cerro Trinidad saddle on SE slope, Kirkbride and Duke 1643 (MO, NY); El Llano-Cartí road about 4.6 miles north of junction with Pan-American Highway, trail to east, McPherson 12514 (MO, NY). **San Blas**: El Llano-Carti road near Nusagandi along Sendero Nusagandi, c. 11 road-miles from Pan-American Highway, McPherson 12731 (MO, NY). **Veraguas**: Serranía de Tute, Aranda et al. 2672 (NY); above Santa Fé on slopes of Cerro Tute below Agricultural School, Gentry 6217 (MO, NY).

**COLOMBIA. Antioquia**: Municipio Campamento, 6 km NO del pueblo en la vía a Mina Las Brisas, Callejas et al. (MO, NY). **Chocó**: cerca del río Atrato en los alrededores de Quibdó, Araque and Barkley 129 (NY); Rio Munguido afluente del rio Atrato, alrededores de Altagracia, Forero et al. 1504 (MO, NY); Top of Serranía del Darien ca. due east of Unguia, Gentry et al. 16790 (MO, NY); Top of Serranía del Darien exactly on the frontier with Panama, N.E. of Cerro Mali, Gentry et al. 16982 (MO, NY); North ridge of Alto de Buey east-southwest of El Valle, Gentry and Fallen 17381 (NY); 7 km W. of Tutenendo on road to Quibdo, Gentry and Fallen 17591 (MO, NY). **El Valle**: Cordillera Occidental, vertiente occidental, Hoya del río Digua lado izquierdo, Piedra de Moler, Cuatrecasas 14983, 15100 (NY); Costa del Pacífico, río Yurumanguí, El Aguacate, Cuatrecasas 16146 (NY); Río Calima (región del Chocó), entre Herradura de Ordónez y Peña de Campotriste, Cuatrecasas 16633, 16689 (NY); Costa del Pacífico, río Cajambre, Barco, Cuatrecasas 16950, 17247 (NY); Punta Magdalena, Haught 5589 (NY). **Gorgona Island**: “Sr. George” Expedition 705 (NY). Nariño: Barbacoas, Alston 8490 (NY).

**ECUADOR. Esmeraldas**: San José km 321 along railroad from Ibarra to San Lorenzo, Boom 1325 (MO, NY); The Mache-Chindul Ecological Reserve, Bilsa Biological Station, Mache mountains 35 km W of Quinindé, collected on Ramon Loor’s property, Clark 3088 (NY); Bilsa Biological Station, Reserva Ecologica Mache-Chindul 40 km NW of Quinindé, Loma de los Guerrilleros, Permanent plot 2, Clark 4054 (MO, NY); Territorio Indígena Awá, Mataje village, Neil et al. 12498 (MO, NY); Reserva Biologica Bilsa, sendero Amarillo, Stern and Tepe 392 (NY). **Pichincha**: Carretera Quito-Puerto Quito km 113, Betancourt 139 (NY); Reserva de ENDESA km 113 along Quito-Pto. Quito rd, Luteyn and Borchsenius 13343 (NY).

**VENEZUELA** (fide Schnell). **Zulia**: San José de los Altos, Sierra Perijá, Delascio and Benkowsky 2952 (US).

### Conostegia muriculata

Taxon classificationPlantaeMyrtalesMelastomataceae

Almeda

Fig. 93

Conostegia muriculata Almeda, Proc. Calif. Acad. Sci. 46: 330. 1990. Type: Panama. Bocas del Toro: above Chiriqui Grande 10 road-miles from continental divide and 2 mi along road to E (0855'N 8210'W, 300 m), 6 August 1988, *G. McPherson 12836* (holotype: CAS!, isotypes: CR!, DUKE, MO!, PMA, US!).

#### Description.

Shrubs to small trees to 3.5 m tall with slightly to evidently tetragonal stems towards the apex, essentially glabrous or with inconspicuous scales; the nodal line present. Leaves of a pair somewhat unequal in size. Petioles 1.7–7.5 cm long. Leaf blade 18–27 × 6–13.5 cm, 3–5 nerved, elliptic to elliptic-ovate, the base acute or obtuse, the apex acute to abruptly acuminate, the margin entire, both surfaces glabrous but inconspicuously glandular puncticulate. Inflorescence a terminal, elongated and deflexed or arching panicle 8–30 cm long branching well above the base, accessory branches absent or present, rachis glabrous, bracts and bracteoles to 1 mm long, linear, early deciduous. Pedicels 1.5–2.5 mm. Flowers 5 merous, pyriform, calyptrate. Flower buds 5–9 × 3–4.5 mm, the base rounded to obtuse, the apex acute to acuminate, constricted below the calyptra, the calyptra and hypanthium differentiated; the hypanthium 2–2.5 × 2.75–3.15 mm, campanulate, glabrous and inconspicuously glandular puncticulate. Petals 5–7 × 4.5–6 mm, white to lavender, obovate, glabrous, emarginate. Stamens (9-)10, 4.5–5.5 mm long, slightly zygomorphic, the filament ca. 3 mm, white, anthers 2.25–2.75 × 0.6–0.9 mm, linear-oblong, yellow or pale yellow, the pore ca. 0.1 mm, subterminal and ventrally inclined. Ovary 5–6(-7), inferior, apically glabrous and lacking a collar around the style base. Style 3–3.5 mm, straight and bending below the tip, vertical and horizontal distance from the anther to the stigma ca. -1.25 – -0.5 mm, stigma capitate, 1–1.5 mm wide. Berry 5–6 × 5–6 mm, dark purple. Seeds 0.4–0.6 mm, ovoid to pyramidal, the testa muriculate.

**Figure 93.**
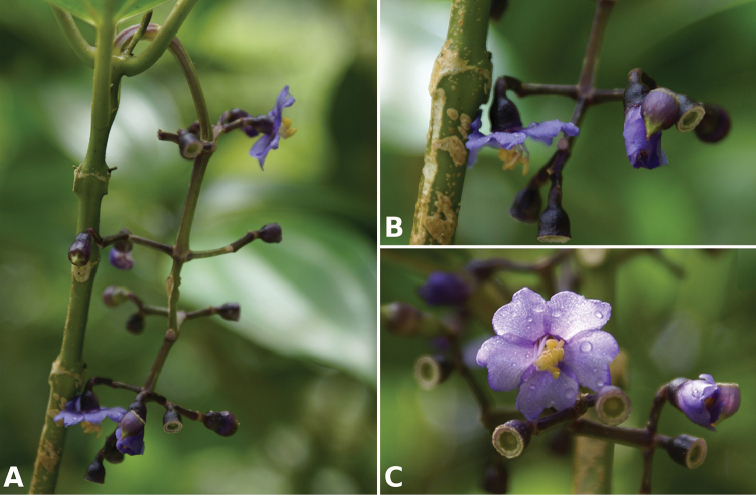
*Conostegia muriculata*. **A** Inflorescence **B** Lateral view of flower **C** Frontal view of flower. Photographs by Laurencio Martínez.

#### Distribution

**Figure 94.**
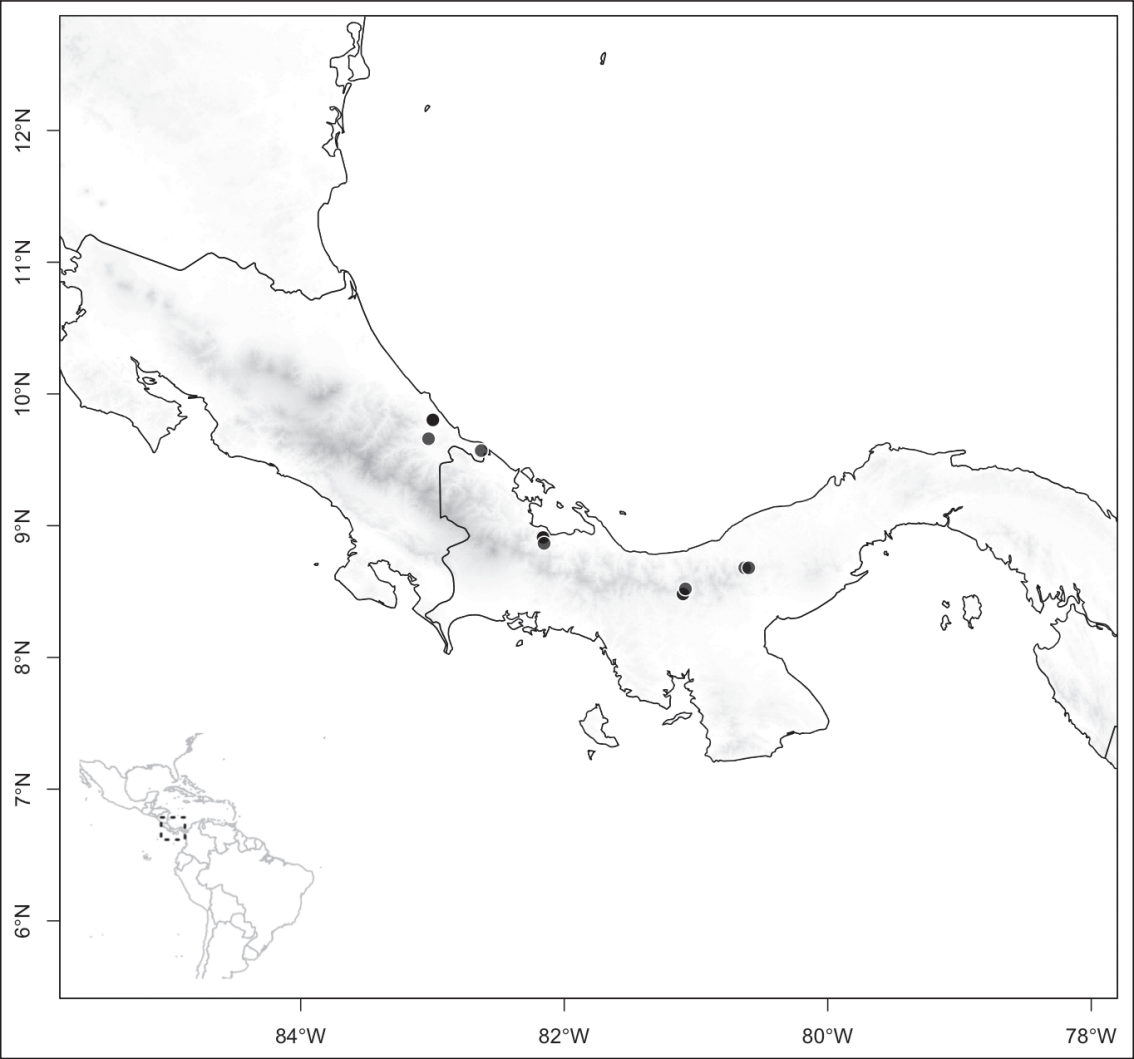
Distribution of *Conostegia muriculata*.

(Fig. 94). Endemic to the Caribbean slope of south eastern Costa Rica and northern Panama, 40–1200 m in elevation.

*Conostegia muriculata* is a distinctive species recognized by being overall glabrous, pendant inflorescence, purple petals and muriculate seeds. Unfortunately the phylogenetic placement of this species remains unknown. Because of its small flower buds and being almost totally glabrous, it likely belongs in the *Conostegia montana* complex in section *Conostegia*. As in all species in section *Conostegia*, the style is short (Fig. 93). Schnell (1996) mentions the fact that several specimen labels point to an understory habitat and suggests it is probably a shade tolerant species.

#### Specimens examined.

**COSTA RICA. Limón**: Sixaola, San Miguel, Finca Albergue ASACODE, Quesada 356 (CR, NY).

**PANAMA. Bocas del Toro**: above Chiriquí Grande on a side road about 10 road miles below the Continental Divide about 2.5 miles east on that road, Almeda et al. 6328 (CAS, CR, MO, NY). **Veraguas**: Distrito de Santa Fé, Serranía de Tute, Galdames et al. 3122 (MO, NY).

### Conostegia oerstediana

Taxon classificationPlantaeMyrtalesMelastomataceae

O. Berg ex Triana

Fig. 95

Conostegia oerstediana O. Berg ex Triana, Trans. Linn. Soc. 28: 98. 1872. Type: Costa Rica. Naranjo, no date, A. Oersted 11 (holotype: C; isotypes: BR!, K!, GH).

#### Description.

Trees 4.5–18 m tall with whitish flaky bark and tetragonal and ridged stems that are glabrous or with sessile stellate trichomes, these usually minute and inconspicuous; the nodal line present but frequently inconspicuous, lenticels frequent at the nodes. Leaves of a pair equal to somewhat unequal in length. Petiole 1.3–9 cm. Leaf blade 5.2–25 × 2.9–15 cm, 3–5-plinerved, with the innermost diverging from the mid vein just above the blade or rarely up to 3 cm above the base in opposite or alternate fashion, ovate or elliptic, thick, the base acute to rounded, the apex rounded and abruptly acute to acuminate, the margin entire to denticulate, glabrous on both surfaces. Inflorescence a terminal panicle 6–18 cm long branched above the base but sometimes appearing branched at the base because of multiple inflorescences arising at opposing meristems at the terminal node, bracts absent or if present elliptic to ovate and up to 3 cm long, accessory branches present, the rachis glabrous, bracts apparently lacking and bracteoles early deciduous. Pedicels 1–8 mm. Flowers 6–10 merous (mostly 8), calyptrate. Flower buds 5–12 × 5–10 mm, spherical, the base rounded to truncate, the apex rounded to truncate and usually short apiculate, not constricted and undifferentiated; the hypanthium 5–5.5 × 7–8.25 mm, smooth to more frequently tuberculate, glabrous and frequently evidently white. Petals 7–11 × 7–11 mm, white, obovate, spreading and overlapping, emarginate, glabrous, spreading and overlapping, entire. Stamens (20-)24–28(-36), 7–9 mm long, radial but appearing bilateral because stamens often get stuck below the stigma, the filament 4–5 mm, white, anthers 3–4 × 1–1.5 mm, elliptical to linear-oblong, yellow, strongly laterally compressed, the base sagittate, the pore 0.2–0.3 mm, terminal. Ovary 14–20 locular, inferior, apically glabrous. Style 5.75–8 mm, bending downward to ca. 45 degrees throughout anthesis and protruding below the anthers, vertical distance from the anthers to the stigma ca. 0 mm, horizontal distance ca. -2–0; stigma crateriform, consisting of 14–21 laterally compressed lobes, 4–6 mm wide. Berry 11–14 × 8–10 mm, blue-black or purple-black. Seeds 0.6–0.8 mm, pyramidal, the testa smooth.

**Figure 95.**
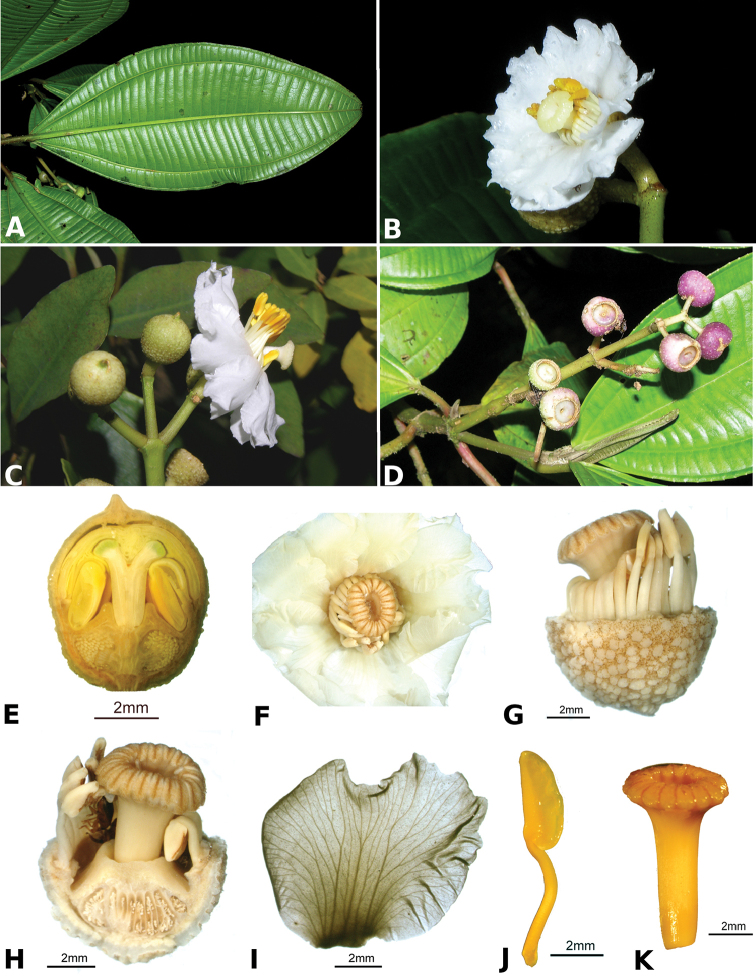
*Conostegia oerstediana*. **A** Leaf abaxial surface **B** Flower at anthesis showing many stamens stuck below the stigma **C** Lateral view of a flower showing bent style **D** Infructescence **E** Longitudinal section of flower bud **F** View of pickled flower from above **G** Lateral view of pickled flower with petals removed **H** Longitudinal section of a flower at anthesis **I** Petal **J** Stamen **K** Style **A** and **D** from specimen vouchered *R. Kriebel 5633* **B, C, E–K** from specimen vouchered *R. Kriebel 5627*.

#### Distribution

**Figure 96.**
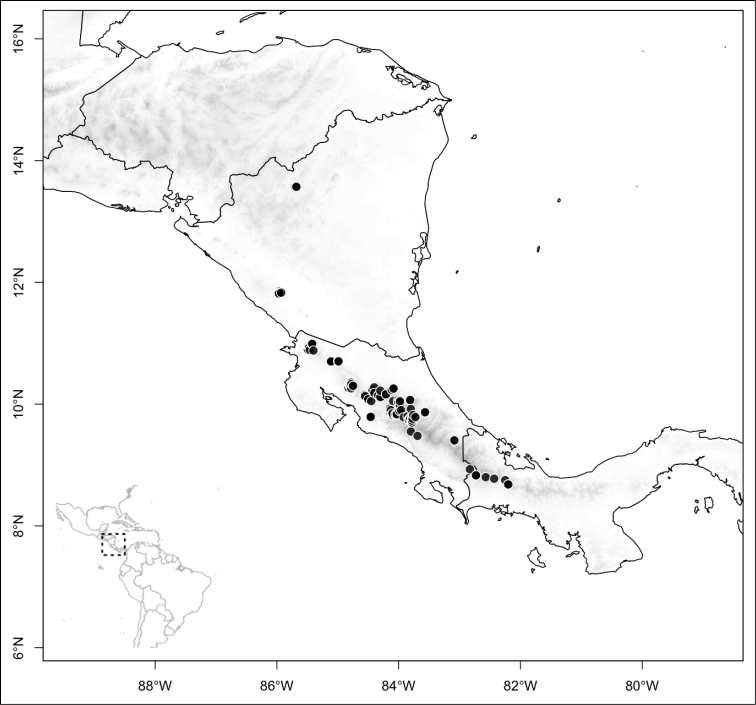
Distribution of *Conostegia oerstediana*.

(Fig. 96). Nicaragua to west Panama where it occurs in the mountains from (550)1000–2400 m elevation.

Specimens from Chiriquí have been annotated as *Conostegia macrantha* (*White 194*, *Penneys and Olmos 1738*-both at NY). I have included these specimens in the circumscription of *Conostegia oerstediana* on the basis of their glabrous leaves as well as on the description of the style in the second above mentioned specimen where the style was described as curving downward whereas in *Conostegia macrantha* it is straight. Most populations have tubercles on the hypanthium but in some populations these are reduced or lacking. When the tubercles are lacking, *Conostegia oerstediana* looks similar to *Conostegia bernoulliana*. The latter lacks the spherical floral buds of *Conostegia oerstediana* and instead has a constriction in the middle of the bud. Also, the leaves in *Conostegia bernoulliana* are narrower. *Conostegia oerstediana* is also similar to *Conostegia bigibbosa*. The latter name was considered a synonym of *Conostegia oerstediana*. See the discussion under *Conostegia bigibbosa* for the differences between the two species. See also Schnell (1996) for details on crossing experiments between *Conostegia oerstediana* and close relatives. Flowers of this species have good fragrance which has been reported on several specimens (e.i., *N. Zamora 5843*, *5844*, *5845*-INB, MO). These flowers have been observed to be visited by *Bombus volluceloides* and other large unidentified bees. Berries of this species have a good taste. Schnell (1996) notes the almost exclusivity of growing on volcanic soils of this species and Umaña Dodero (1988) documented peak flowering in December for populations in Grecia, Costa Rica. *Phoradendron chrysocladon* A. Gray has been reported as a parasite of *Conostegia oerstediana* (Braby and Nishida, 2010).

#### Specimens examined.

**NICARAGUA. Granada**: in forest on Mombacho Volcano, Williams 20009, 20039 (MO, NY).

**COSTA RICA. Alajuela**: Los Angeles de Heredia, Brenes 14646 (CR, NY); Los Angeles de San Ramón, Brenes 168, 3820, 6093, 6711, 13630 (CR, NY); El Socorro de San Ramón (Haut de la Palma), Brenes 5242 (CR, NY); San Miguel Arriba de Grecia, Finca Arnold Haehner, Kriebel 5408 (INB, NY); Zarcero, Upper edge tropical zone, Pacific watershed edge of woodland, Smith 7 (NY); near Tapezco River, Cordillera Central, about 10 km north of Zarcero, Williams et al. 28927 (NY). **Cartago**: Paraíso, Parque Nacional Tapantí Macizo de La Muerte, a orilla de la calle el cruce de Río Humo al tunnel, Acosta and Ramírez 572 (INB, MO, NY); a 20 metros del río Grande de Orosi, Tapantí, Jiménez 1596 (CR, NY); Paraíso, Parque Nacional Tapantí Macizo de La Muerte, Alrededores de la estación, Kriebel 5338 (INB, NY); Woods beside Río Sombrero, 1.5 km S. of Muneco, Lent 1904 (NY); P. N. Tapantí, 500 meters after Rangers Station, Vargas and Villalobos 3827 (INB, MO, NY). **Heredia**: Collected along the slopes of Cerro Zurquí above the Río Para Blanco, Taylor 17631 (NY). **Puntarenas**: Cordillera de Tilarán, Road to San Luís about 3 km downslope from the Monteverde Cloud Forest Reserve Station, Almeda and Daniel 7179 (CAS, NY); Monteverde San Luis Cerro Chomogo, Kriebel et al. 5515 (INB, NY); Monteverde property of John Campbell, Lumer 1013 (NY). **San José**: Vicinity of Altos Tablazo about 10 km. W of Tablon and SE of Higuito, Almeda et al. 2841 (CR, NY); Old cart road to Limon, 5.6 km N of San Jeronimo, near La Palma, Hill et al. 17791 (CR); Acosta, Salvaje, Kriebel 5416 (INB, NY); Alto La Palma, Lumer 1341 (NY); La Palma, Tonduz 7427 (NY); Heavily pastured slopes between Volcán Barba and Irazú about 5 miles northeast of San Vicente, Wilbur and Stone 9666 (CR, NY); in mountains near Tarbaca about 15 kms south of San José, Williams 19466 (NY).

**PANAMA. Chiriquí**: Vicinity of Gualaca ca 8 mi from Planes de Hornito, La Fortuna on road to damsite, Antonio 5157 (MO, NY); Fortuna Dam region, along Quebrada Arena near continental divide, McPherson 8723 (NY); above Boquete near Parque Nacional Volcan Barú, along Río Caldera in Alto Chiquero, Penneys and Olmos 1738 (NY); Distrito de Bugaba, Santa Clara, Hartmann’s finca, van der Werff and Herrera 7091 (MO, NY).

### Conostegia pittieri

Taxon classificationPlantaeMyrtalesMelastomataceae

Cogn. Ex T. Durand

Fig. 97

Conostegia pittieri Cogn. Ex T. Durand, Bull. Soc. Roy. Bot. Belg. 27: 176. 1888. Type: Costa Rica. Alto del Roble, Massif du Barba, 1800–2000 m, 6 July 1888, H. Pittier 212 (holotype: BR; isotypes: F!, US!).
Conostegia donnell-smithii Cogn., DC. Monog. Phan. 7: 700. 1891. Type: Costa Rica. Cartago, 4000 ft., 1888, J. Cooper 327 (holotype BR; isotypes BR, F, GH!, NY!, US!). Schnell (1996) notes: Some sheets of this number have been distributed by John Donnell Smith under his number 5471, but since the latter was also used for Cooper 334 (US) care must be exercised in determining isotypes. The Donnell Smith number at least on the GH specimen is 5741 and not 5471.
Conostegia pittieri Cogn. ex Durand var. brevifolia Cogn., DC. Monog. Phan. 7: 704. 1891. Type: Costa Rica. Rio Segundo, Massif du Barba, 2000 m, Tonduz 1732 (lectotype BM!, designated here; isolectotypes BR, M!, US!). Other syntypes: Costa Rica. Rancho Flores, versant S du Massif du Barba, 2050 m, Costa Rica, Pittier 290 (BR, US!). The latter collection is labeled Pittier and Tonduz 290 in US, a discrepancy common with the exsiccatae of these men and apparently due to erroneous use of the printed labels bearing both their names. Handwritten label with both names were never seen, nor evidence that the two collected in the same place at the same time (Schnell 1996).

#### Description.

Trees 2–18 m tall with tetragonal stems when young which become terete with age and glabrous; the nodal line present. Leaves of a pair equal to somewhat unequal in length. Petioles 0.5–4.3 cm. Leaf blades 3.5–18.5 × 1.6–6.1 cm, 3–5 nerved or generally 3–5 plinerved, with the innermost diverging from the mid vein just above the blade base in opposite or alternate fashion, elliptic to ovate, the base acute to decurrent on the petiole, the apex abruptly acute to acuminate, the margin entire to undulate-denticulate. Inflorescence a terminal panicle 4–12.1 cm long branching above the base, bracts and bracteoles to 3 mm, linear, deciduous. Pedicels 4–11 mm long. Flowers (5-) 6–8 (-10) merous, calyptrate, floral buds 7–14 × 2.75–6.75 mm, pyriform or ellipsoid, the base rounded, the apex apiculate, slightly constricted in the middle; the hypanthium 3.25–3.75 × 3.75–4.25 mm, glabrous and smooth. Petals 8–14 × 5–9 mm, white, obovate, rotate, rounded-truncate to emarginate, glabrous on both surfaces, rotate, entire, persistent after the stamens and style have fallen. Stamens 14–23, 7–9 mm, opposing the style resulting in zygomorphy, the filament 5.5–6.5 mm, white, anthers 2.5–3.5 × 0.75–1.25 mm, linear and slightly recurved, the base sagittate, yellow, not conspicuously compressed, the pore 0.2–0.26 mm, terminal. Ovary 7–12 locular, inferior. Style 6–6.5 mm, bending to one side of the flower, opposite the stamens, lacking a basal collar, vertical distance between the anthers and the stigma absent, horizontal distance 0.5–3 mm; stigma peltate, consisting of 7–11(-12) laterally compressed lobes, 3–3.5 mm wide. Berry ca. 1 × 1 cm, purple black. Seeds 0.5–0.7 mm, ovoid, the testa smooth.

**Figure 97.**
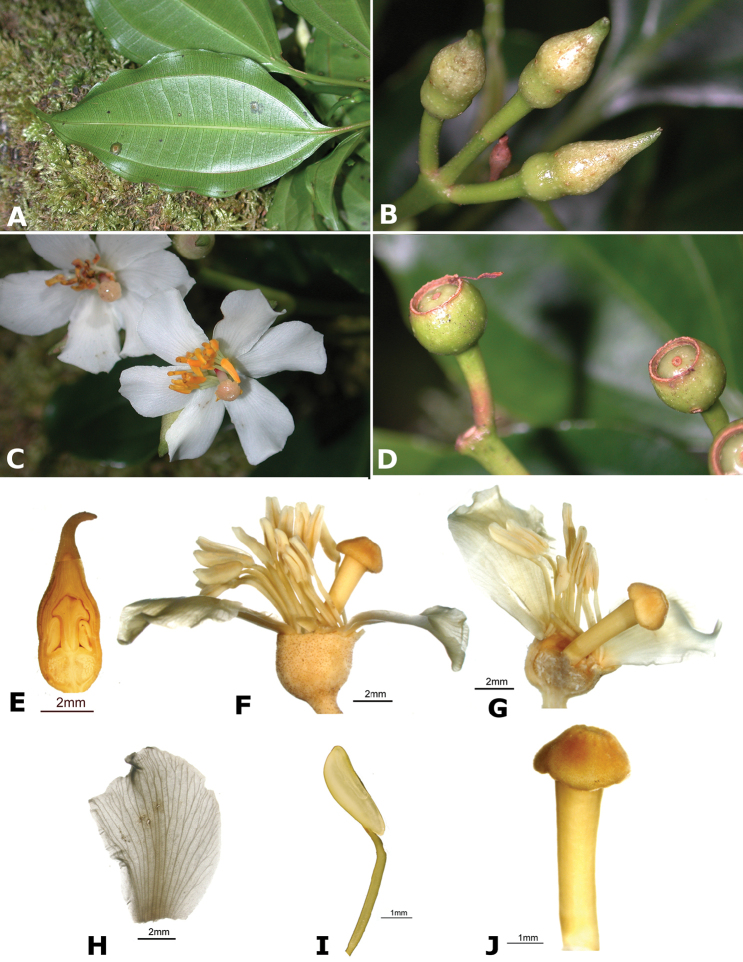
*Conostegia pittieri*. **A** Leaf abaxial **B** Flower buds **C** Flower at anthesis **D** Immature fruit **E** Longitudinal section of a floral bud **F** Pickled flower at anthesis **G** Longitudinal section of a flower at anthesis **H** Petal **I** Stamen **J** Style. Photos of specimen vouchered *R. Kriebel 5400*.

#### Distribution

**Figure 98.**
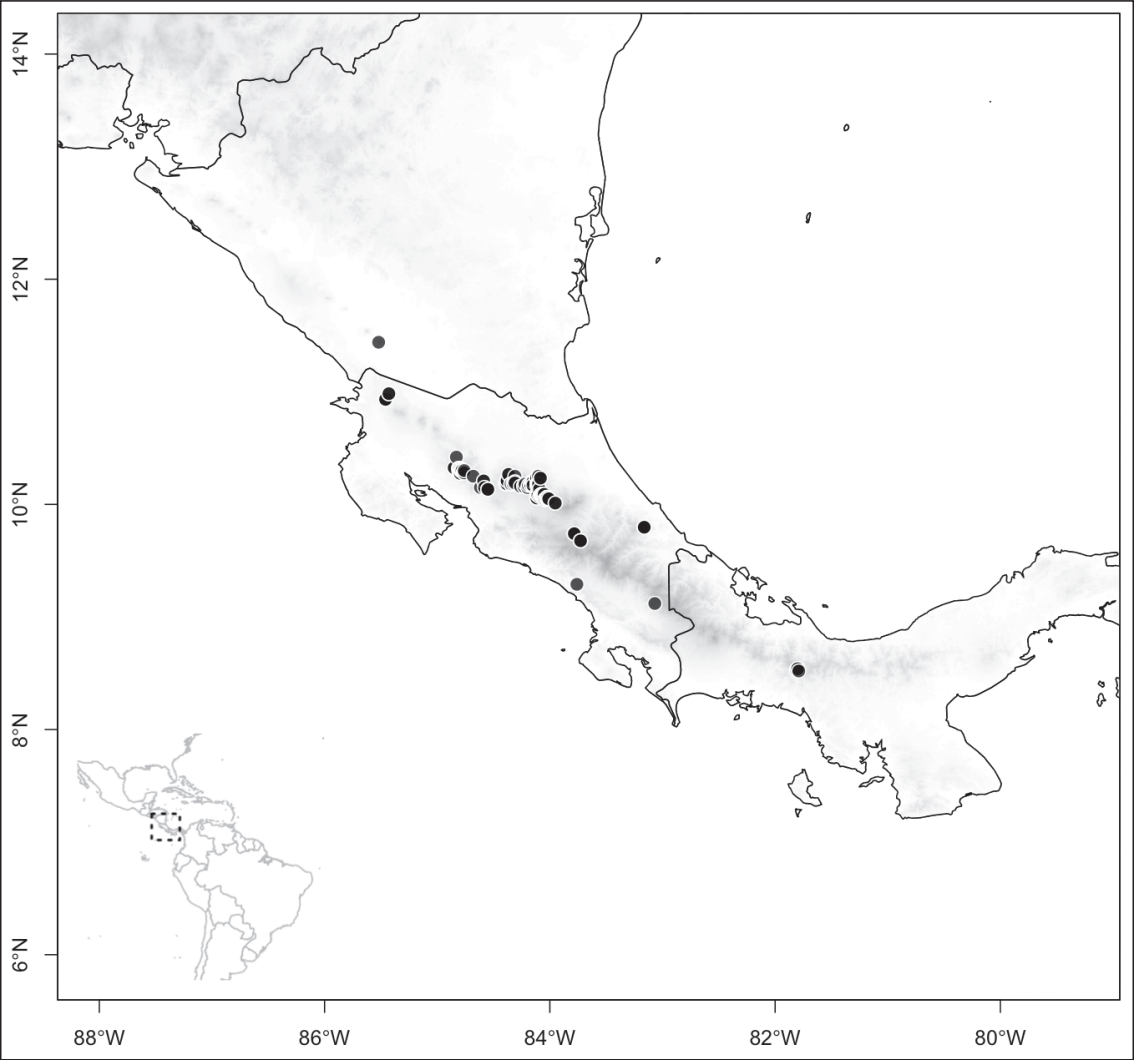
Distribution of *Conostegia pittieri*.

(Fig. 98). In cloud forests from southern Nicaragua through central and northern Costa Rica to northwestern Panama, (500-)1000–2400 m.

*Conostegia pittieri* is one of the few species of *Conostegia* that retains its petals once the stamens and style have fallen. The functional significance of petal retention, if any, might have to do with continuing to make inflorescences attractive (Bertin 1982).The similar species *Conostegia chiriquensis* also retains the petals and the style after the stamens have fallen. See the discussion under the latter species for differences between *Conostegia chiriquensis* and *Conostegia pittieri*. Flowers of *Conostegia pittieri* have been observed to be buzzed by female *Bombus volluceloides* (pers. obs.). As Schnell (1996) mentioned, this species can be quite abundant in pastures and second growth but is rare inside the forest where it occurs mainly in riparian habitats. Individuals within the forest produce much less flowers (pers. obs.).

#### Specimens examined.

**NICARAGUA** (fide Schnell). **Rivas**: Volcán Maderas, Isla Ometepe, Moreno 19760 (MO).

**COSTA RICA. Alajuela**: Upala, Bijagua, P. N. Tenorio, Aguilar 6452 (NY); Palmira de Naranjo, Brenes 3519 (CR, NY); Cerros de San Antonio de San Ramón, Brenes 5664 (NY); La Palma de San Ramón, Brenes 5528, 6787, 6807 (NY); Posada Volcán Póas, Viento Fresco, Vara Blanca, Kriebel and Solano 3266 (INB, NY); Alto Palomo 9 km W of top of Poás Volcano, Lent 1655 (CR, NY); Remnant cloud forest on mountains of Cordillera Central about 2 kms east of Zarcero, Molina et al. 17060 (NY); Cordillera Central near Palmira about 5 kms east of Zarcero, Molina et al. 17769 (NY); Fraijanes Volcán Poás, on road from San Pedro de Poás to Vara Blanca, Schnell 1084 (CR, NY); Palmira (Canton de Alfaro Ruiz), Smith 40 (NY). **Heredia**: Wet secondary forest about 15 m high with many epiphytes with frequent wind and rain from the Caribbean, Rio Vueltas (Upper Río Patria), eastern slope of Volcan Barba near Continental Divide, near Finca Montecristo, Barringer and Christenson 3412 (F, NY); Sacramento, Finca Ingrid Steinvorth, Kriebel 5400 (INB, NY); 5 km down a jackknife turn to the east off of Highway 9, 3.3 km north of Vara Blanca, Luteyn 685 (MO, NY); Vara Blanca de Sarapiquí north slope of Central Cordillera between Póas and Barba volcanoes, Skutch 3415, 3563 (MO, NY); entre Porosatí y Sacramento, Solano et al. 2696 (INB, NY); Collected along the slopes of Cerro Zurquí above Río Para Blanco, Taylor 17481 (NY); Monte de la Cruz, above Finca Monte Cristo, Todzia and Moran 2045 (CR, NY); Forets du Barba, Tonduz 1947 (NY); woods on steep bank of stream about 7 miles northeast of Barba on Route 114, on flank of Volcán Barba, Wilbur and Teeri 13681 (MO, NY). **Puntarenas**: near the Continental Divide about 2 to 5 km east and southeast of Monteverde, Burger and Gentry 8728 (F, NY). **San José**: Vázquez de Coronado, P. N. Braulio Carrillo, Cuenca del Sarapiquí, Santa Elena, Acosta and Ramírez 442 (INB, MO, NY). **PANAMA. Chiriquí**: Gualaca-Chiriqui Grande road, 4.5 mi N of middle of bridge over Fortuna Lake, just S of Bocas del Toro border, Croat 66710 (MO); Fortuna Dam, trail from highway near forestry nursery down to Rio Hornito, McPherson 12498 (MO); Cerro Colorado 50 km N of San Felix on continental divide, Mori and Dressler 7803 (MO).

### Conostegia procera

Taxon classificationPlantaeMyrtalesMelastomataceae

(Swartz) D. Don ex DC

Conostegia procera (Swartz) D. Don ex DC., Prodr. 3: 174. 1828. Melastoma procera Swartz, Prodr. Veg. Ind. Occ. 68. 1788. Type Jamaica. no date, O. Swartz s.n. (lectotype: S!, designated here; isolectotype: BM!).

#### Description.

Trees to about 7 m tall with subtetragonal, stems stellate pubescent apically, glabrous to glabrescent with age; the nodal line present. Leaves of a pair equal to somewhat unequal in length. Petiole 1–5 cm. Leaf blades 4–15.5 × 3.1–7 cm, 3-nerved, elliptic, the base obtuse, the apex obtuse, acute or acuminate, the margin entire or weakly crenulate-dentate near the apex, the adaxial surface glabrous, the abaxial surface glabrous except for tuft domatia of stipitate branching hairs present at the base of the leaf and minute puncticulate glands throughout the surface. Inflorescence a terminal panicle 5–17.7 cm long branched above the base, rachis flattened accessory branches absent or present, bracts and bracteoles linear, 2–3 mm long, deciduous or appearing absent. Pedicels 5–12 mm. Flowers 6 merous, calyptrate. Floral buds 11–18 × 5–7 mm, elliptic pyriform, the base rounded, apex acuminate and apiculate, slightly constricted in the middle, the hypanthium 4–5 × 4.5–5.25 mm, glabrous and ribbed. Petals 10–14 × 6–9 mm, white or pinkish white, obtriangular, spreading, emarginate, glabrous. Stamens (17-)18(-20), 6–7.5 mm long, the filaments 3.5–4 mm long, reportedly yellow, anthers 3.5–4 × 0.5–1 mm, linear, yellow, slightly laterally compressed, the pore ca. 0.2 mm wide, terminal. Ovary (5-)6(-7) locular, inferior, the apex glabrous and elevated into a conspicuous collar around the style. Style 7–8 mm long, bending below the stigma, vertical and horizontal distance from the anthers to the stigma absent; stigma, punctate, ca. 0.5 mm wide. Dry berry 7–9 × 7–9 mm. Seeds 0.5–0.75 mm, ovoid to pyramidal, the testa angulate and smooth to slightly roughened.

#### Distribution

**Figure 99.**
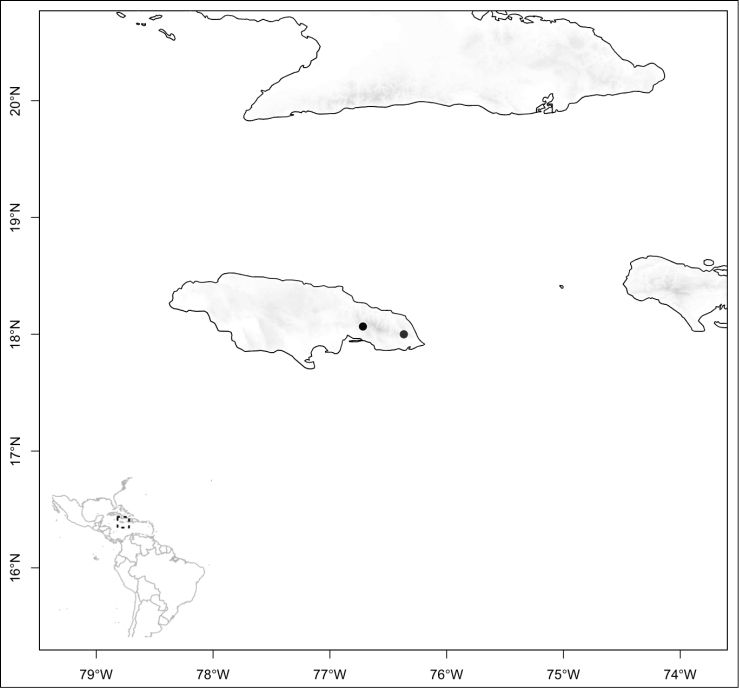
Distribution of *Conostegia procera*.

(Fig. 99). Endemic to Jamaica, 500–1300 m in elevation.

*Conostegia procera* can be recognized by its leaves with mite domatia, usually white flowers, and ribbed hypanthium. Schnell (1996) found this species does not overlap in flowering time with the other Jamaican endemics. Schnell (1996) thought *Conostegia procera* highly resembled *Conostegia jaliscana* and placed it in a section together with the rest of Jamaican endemics. The molecular phylogeny places both Jamaican endemics sampled (including *Conostegia procera*) forming a well supported clade and belonging to a clade that also includes *Conostegia rufescens* and *Conostegia cuatrecasii*. *Conostegia jaliscana* on the other hand falls with species of the giant stigma clade, which is centered in Central America.

#### Specimens examined.

**JAMAICA. St. Andrew**: Southwest slope of Mt. Horeb above Hardwar Gap, Proctor 10321 (NY). **St. Thomas**: Hillside, Mansfield, Britton 3607 (NY); Mountain trail between House Hill and Cuna Cuna Gap, Maxon 8949 (NY).

### Conostegia pyxidata

Taxon classificationPlantaeMyrtalesMelastomataceae

Proctor

Conostegia pyxidata Proctor, Bull. Inst. Jam. Sci. Series 16: 38. Pl. 15, p. 39. 1967. Type: Jamaica. Portland Parish: East slope of John Crow Mts, 1.5–2.5 miles southwest of Ecclesdown, 1500–2500 ft, 11 August 1956, G. R. Proctor 10468 (lectotype: NY!, designated here; isolectotypes: IJ, LIL!).
Conostegia subprocera Proctor, Bull. Inst. Jam. Sci. ser. 16: 38. Pl. 16, p.40. 1967. Type: Jamaica. Portland Parish: east slope of John Crow Mts, c. 1-1.5 miles southwest of Ecclesdown, 1500 ft., 6 August 1954, G. R. Proctor 9229 (holotype: IJ).

#### Description.

Shrubs to small trees 1–5 m tall with subtetragonal stems that become terete and that are densely beset with simple roughened to dendritic trichomes near the tips, a small layer of inconspicuous underdeveloped dendritic trichomes also present, glabrescent to glabrous with age; the nodal line present, covered or not by indument on young branches. Leaves of a pair equal to somewhat unequal in length. Petiole 0.5–3.5 cm, adaxially pubescent like the stem apices. Leaf blade 4–13 × 2–5 cm, 3-nerved, oblong, linear to elliptic or ovate to obovate, the base acute to rounded, the apex rounded to acute or acuminate, the margin entire, the adaxial surface glabrous, the abaxial surface essentially glabrous, sometimes with short stipitate and sessile stellate trichomes on the mid vein and some secondary veins and inconspicuously glandular puncticulate throughout. Inflorescence a terminal panicle 3–8.5 cm long branched above the base, accessory branches apparently absent, bracts and bracteoles 0.5–2 mm, subulate, deciduous. Pedicel 7–13 mm. Flowers 5–7 merous, calyptrate. Floral buds 11–20 mm long, ovoid, the base obtuse to rounded, the apex acute and mucronate, not constricted, the hypanthium 6–7 × 6–7 mm, smooth and glabrous. Petals 6–17 mm long, white. Stamens 11–18, 6–8 mm long, the filaments 3.4–4.4 mm long, white, lacking a conspicuous geniculation, anthers 3–4 × 0.8–1 mm, subulate, recurved at the tip, yellow, somewhat laterally compressed, the pore ca. 0.1 mm wide, terminal. Ovary 5–7 locular, inferior, apically glabrous and forming a collar around the style base. Style ca. 8–9 mm, straight to slightly bent at the tip, vertical distance from the anther to the stigma -0–1 mm, horizontal distance absent, the stigma truncate, ca. 04–0.5 mm wide. Berry ca. 10 × 10 mm, red at first but turning purple black with maturity. Seeds ca 0.8 mm, pyramidal, smooth.

#### Distribution

**Figure 100.**
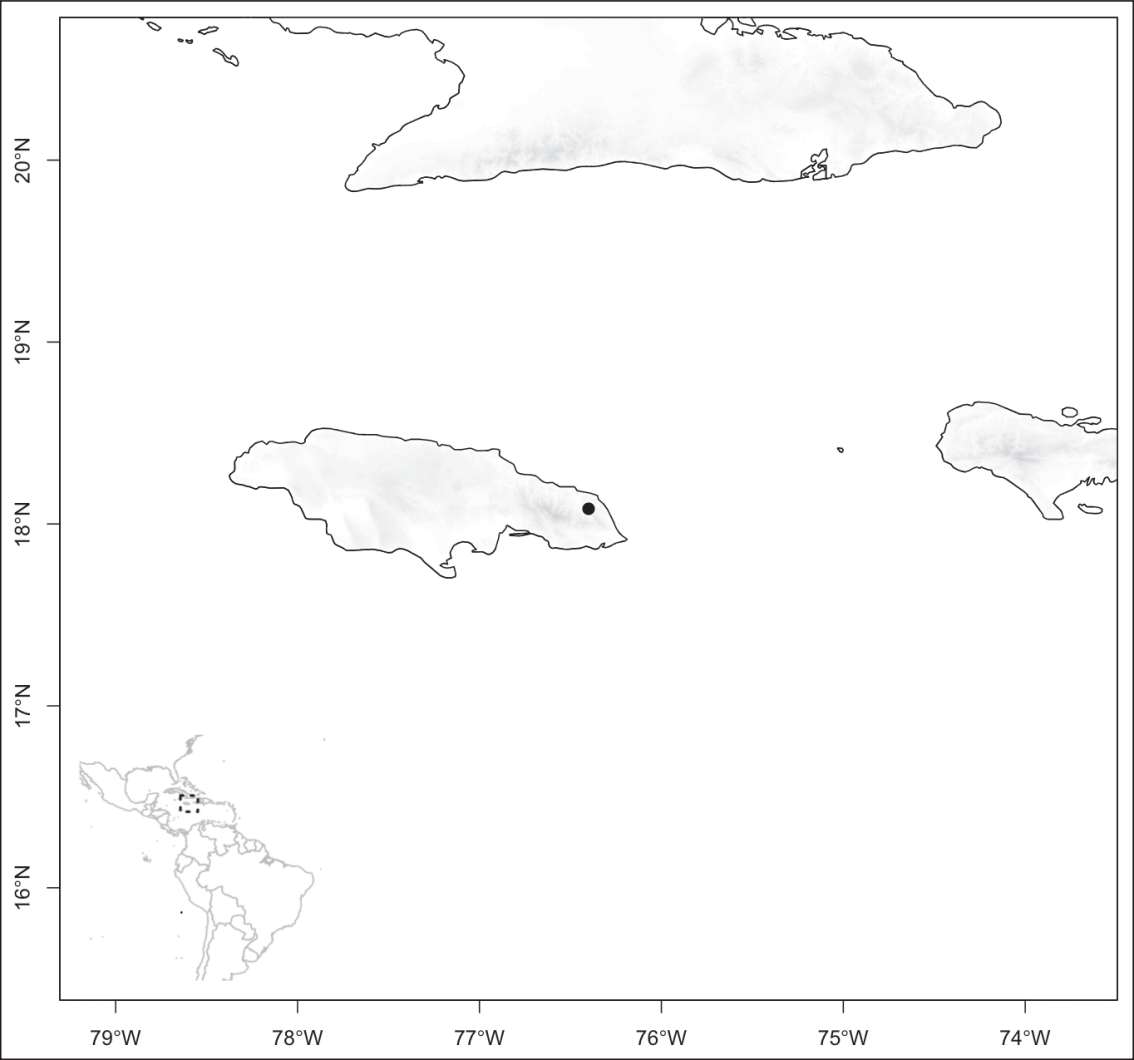
Distribution of *Conostegia pyxidata*.

(Fig. 100). Endemic to Jamaica, 200–1100 m in elevation.

Schnell (1996) discusses at length that *Conostegia pyxidata* is very similar to *Conostegia balbisiana* and *Conostegia procera*. He chose to maintain the species on a “borderline” decision and gave three reasons for keeping the three taxa as distinct: 1) some morphological differences in a small area; 2) the disjunct range of *Conostegia balbisiana*; and 3) differences in flowering time between the more similar species *Conostegia procera* and *Conostegia pyxidata*, and *Conostegia balbisiana* overlapping somewhat in flowering time with the other two species, but having larger flowers with different stamen morphology. Schnell (1996) argued that these morphological and phenological differences suggested the presence of biological isolation and a diverging lineage. For the time being I have chosen to follow Schnell’s reasoning for recognizing three taxa. Schnell (1996) also suggested the possibility of introgression from *Conostegia rufescens*. The type of *Conostegia subprocera* was not studied but Schnell (1996) cited this name as a synonym of *Conostegia pyxidata*.

#### Specimens examined.

**JAMAICA. Portland**: east slope of the John Crow Mts 1.5–2 miles southwest of Ecclesdown, Proctor 9814 (NY); east foothills of John Crow Mts Along Ecclesdown road ca. 2 miles N of Ecclesdown, Skean and Slantis 1864 (MO, NY).

### Conostegia rhodopetala

Taxon classificationPlantaeMyrtalesMelastomataceae

Donn. Smith

Fig. 101

Conostegia rhodopetala Donn. Smith, Bot. Gaz. 42: 295. 1906. Type: Costa Rica. San José: La Palma de San José, 1500–1700 m, 22 May 1898, Tonduz 12347 (lectotype: NY!, designated here; isolectotypes: BR!, F!, US!). Other syntypes: COSTA RICA. San José: La Palma de San José, 1500–1700 m, A. Tonduz 9702 (BR!(2), NY!, US!), H. Pittier 10169 (BR!(2), M!, US!); Heredia: San Isidro, Rio de Las Lajas,1500 m, H. Pittier 14022 (F!, NY!, US!).

#### Description.

Trees 3–12 m tall with tetragonal glabrous stems that sometimes have inconspicuous dendritic trichomes particularly noticeable when dry; the nodal line present but faint. Leaves of a pair equal to somewhat unequal in length. Petiole 1–7.1 cm. Leaf blades 7–23.1 × 3–9.8 cm, 3–5 plinerved, with the innermost pair of primary veins diverging from the mid vein 0.5–1 cm above the base in opposite or sub opposite fashion, elliptic to oblong or elliptic ovate, the base acute or obtuse, the apex acute to caudate, the margin entire, glabrous on both surfaces (except some specimens with tiny dendritic trichomes abaxially). Inflorescence a terminal panicle 11–25.7 cm long branched above the base but sometimes appearing branched at the base because of multiple inflorescences arising at opposing meristems at the terminal node, accessory branches present, rachis pink; bracts and bracteoles apparently lacking. Pedicel 2–15 mm. Flowers (5-)6(-7) merous, calyptrate. Floral buds 5–11 × 2.5–5 mm, obovoid pyriform, the base obtuse, the apex apiculate, slightly constricted in the middle, the hypanthium 3.5–4 × 4–4.25 mm, campanulate glabrous. Petals 5–7(-8.3) × 6–8 mm, pink or rarely white, obovate, spreading, the apex emarginate, glabrous. Stamens 12–15(-17), 4.5–6.5 mm long, slightly zygomorphic, the filaments 2.25–3.75 mm, white, anthers 2.5–3.25 × 0.75–1 mm, elliptic to oblong, sagittate at the base, somewhat laterally compressed, yellow, the pore around 0.1 mm wide, terminal to slightly ventrally inclined. Ovary (5-) 6 (-7) locular, inferior, the apex glabrous, forming a collar around the style. Style 4.5–5 mm, bent below the stigma, vertical distance between anther pore and stigma ca. -1 – -0.5 mm, horizontal distance ca. 0–1mm, stigma slightly expanded, 0.75–1 mm wide. Berry 6–7 × 6–7 mm, purple-black. Seeds 0.3–0.55 mm, ovoid to pyramidal, the testa smooth.

**Figure 101.**
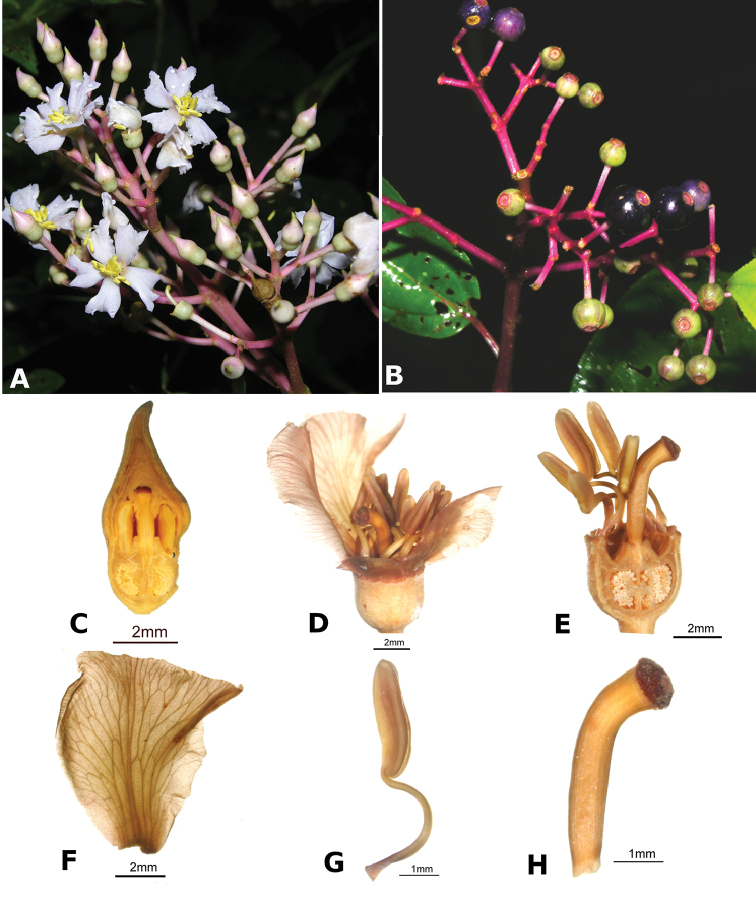
*Conostegia rhodopetala*. **A** Inflorescence **B** Infrutescence **C** Longitudinal section of a flower bud **D** Pickled flower collected at late anthesis **E** Longitudinal section of a flower at late anthesis with petals removed **F** Petal **G** Stamen **H** Style. Photos **A, C–H** of specimen vouchered *R. Kriebel 5542* **B** from specimen vouchered *R. Kriebel 5462*.

#### Distribution

**Figure 102.**
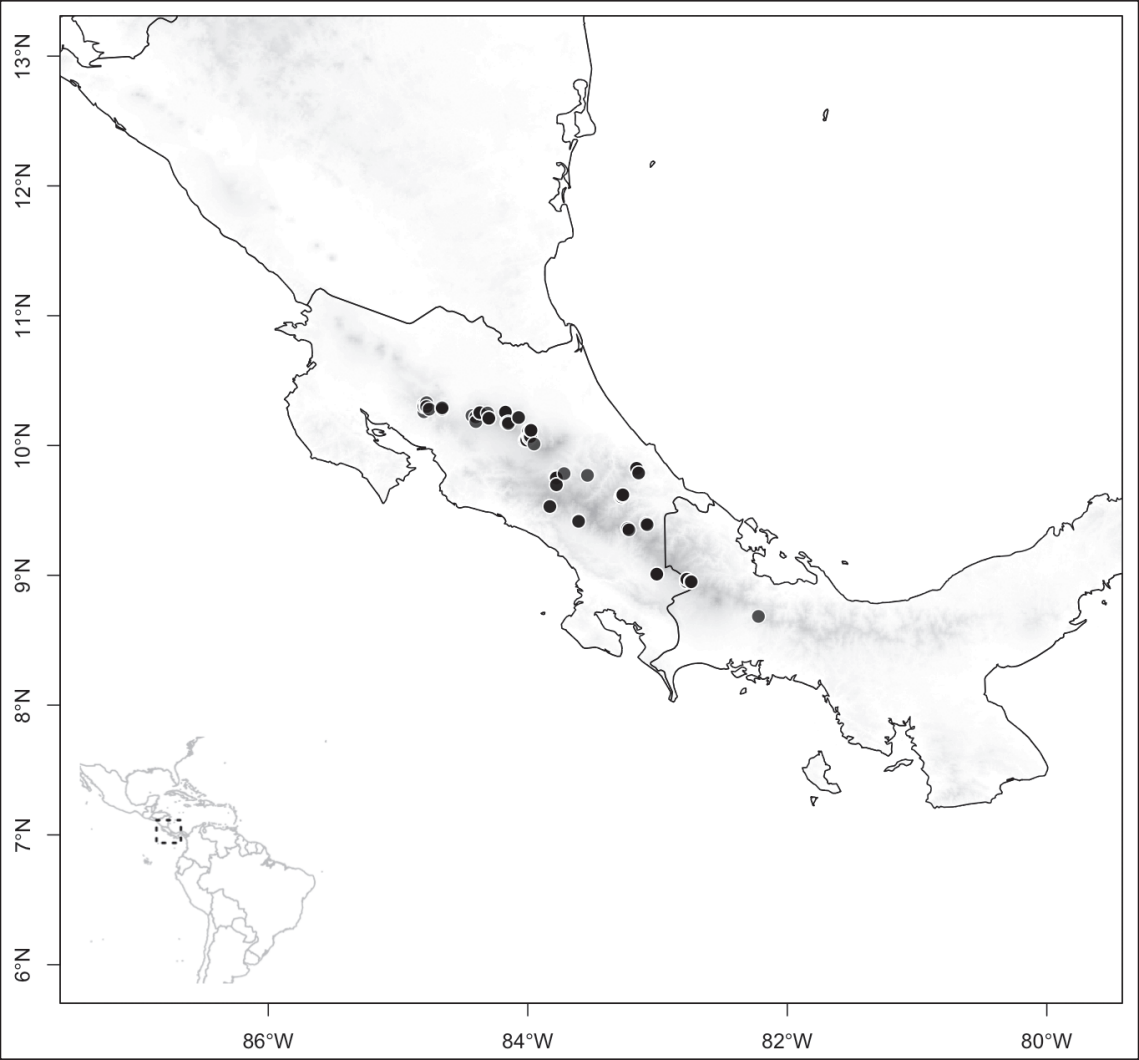
Distribution of *Conostegia rhodopetala*.

(Fig. 102). Cloud forests in Costa Rica reaching western Panama, 700–1900 m in elevation.

*Conostegia rhodopetala* can be distinguished by being almost entirely glabrous, having pink inflorescences and floral buds, apiculate floral buds and pink petals. It is similar to *Conostegia superba*, which as Almeda (2009) recently mentioned has wider leaves and white petals. Schnell (1996) noted a delay in of up to 5–6 months in fruit ripening and a much shorter fruiting than flowering season.

#### Specimens examined.

**COSTA RICA. Alajuela**: San Ramón, Reserva Monteverde, Sendero El Camino, Haber and Zuchowski 12398 (INB, NY); San Carlos, La Tigra, entre la Fila divisoria de agua (Rincón de Cedral) y cerro Bekom, Herrera et al. 8999 (CR, MO, NY); vicinity of La Palma, Maxon 445 (NY); Zapote de San Carlos, Smith 381 (NY); Guadalupe de Zarcero, cantón de Alfaro Ruiz, Smith 702 (NY); P. N. Juan Castro Blanco. Cuenca alta del río La Vieja. Colectando a orillas del río y en la fila, Solano et al. 2667 (INB, NY). **Cartago**: Jiménez, Pejibaye, Turrialba, Refugio El Copal, Sendero Galbula, Kriebel et al. 5462 (INB, NY); Volcan Turrialba on road from La Trinidad to Lechería La Central, Schnell 1076 (NY). **Heredia**: Vara Blanca on road to Cariblanco, Schnell 1081 (MO, NY); Vara Blanca de Sarapiquí north slope of Central Cordillera, Skutch 3224 (NY). **Puntarenas**: Coto Brus, Sendero Las Tablas camino a Cotoncito, Kriebel and Solano 3176 (INB, NY). **San José**: Moravia, P.N. Braulio Carrillo, Cuenca del Sarapiquí, Camino al Bajo de la Hondura, Acosta and Ramírez 389 (INB, NY); Woods high above Río Cascajal 3 km N.E. of Cascajal, Lent 2177 (NY); Vicinity of El General, Skutch 2989 (MO, NY).

**PANAMA. Chiriquí**: Sendero Rio Hornito, Kriebel and Burke 5760 (PMA, NY).

### Conostegia rufescens

Taxon classificationPlantaeMyrtalesMelastomataceae

Naudin

Fig. 103

Conostegia rufescens Naudin, Ann. Sc. Nat. Bot. ser. 3 16: 108. 1850. Type: Jamaica. no date, W. Purdie s.n. (Schnell (1996) cited holotype at P “fide Almeda pers. com.” but annotated that of K as the holotype; isotypes: GH!, K!, fragment BR).
Conostegia formosa Macfad., Fl. Jamaica 2: 70. 1850; nom. inval.
Conostegia puberula Cogn., DC Monog. Phan. 7: 703. 1891. Type: NICARAGUA. Chontales: no date, B. Seemann 30 (lectotype: BM!, designated here; isolectotypes: BR, K!, LE, W). Additional syntype: Costa Rica. Naranjo, Wendland s.n. (GOET).
Conostegia hotteana Urban & Ekman, Ark. Bot. 22a 17: 29. 1929. Type: Haiti. Massif de la Hotte, western group, Dame-Marie, Montagniac, 500 m, Ekman 10324 (holotype: S!; isotypes: A!, C, IJ, NY!, US!).
Conostegia affinis Urban, Arkiv. Bot. 22a 17: 29. 1929. Type: Haiti. Massif de la Hotte, western group, Jeremie, near La Source Chaude, E. Ekman 10264 (holotype: S!).

#### Description.

Shrubs to trees 1.5–20 m tall with tetragonal to terete slightly ridged stems that are usually densely covered with small brown dendritic trichomes sometimes intermixed with sessile stellate and stalked-stellate trichomes, sometimes glabrescent; the nodal line present (sometimes obscured by indument). Leaves of a pair equal to somewhat unequal in length. Petioles 0.4–5 cm, occasionally densely setose adaxially. Leaf blades 8–27 × 3–10.5 cm, 3–5 nerved or if plinerved, with the innermost diverging from the mid vein up to about 1 cm above the base in opposite or sub opposite or alternate fashion, elliptic, the base obtuse to acute and sometimes decurrent on the petiole, the apex acute, acuminate or short-caudate, the margin entire to denticulate, the adaxial foliar surface essentially glabrous, the abaxial surface densely or lightly furfuraceous or puberulent with mealy brown stellate or branching trichomes, sessile or short stipitate, thick bodied and short branched. Inflorescence a terminal panicle 5–23 cm branched well above the base but sometimes appearing branched at the base because of multiple inflorescences arising at opposing meristems at the terminal node and with the flowers frequently clustered at the end of the branches, accessory branches absent or present, the rachis pubsecent with brown stellate and branching trichomes, bracts early deciduous or absent, the bracteoles to 3 mm, deciduous. Pedicel 1–3 mm. Flowers 7–8(-12) merous, calyptrate. Floral buds 5.5–12.75 × 3–7 mm, broadly pyriform, the base and apex obtuse to acute, slightly constricted below the torus, the hypanthium 3.5–4.5 × 4–5 mm, ferrugineous. Petals 7–11 × 5–7.5 mm, white or pink, obovate, spreading, rounded-truncate to emarginate, glabrous. Stamens 20–28, 6–7 mm, slightly zygomorphic, the filaments 3.5–5.25 mm, white, anthers 2.75–3.5 × 0.5–0.75 mm, linear and often recurved, yellow or rarely pink or yellow with a pink tip, the base sagittate, somewhat laterally compressed, the pore ca. 0.1 mm, subterminal. Ovary 10–14 locular, inferior, apically glabrous and with a conspicuous collar around the style base. Style 4–7 mm long, straight or slightly bending just below the stigma, distance from the anthers to the stigma ca. -1.5–0 mm, horizontal ca. distance 0–2 mm; stigma capitate, 1.25–75 mm wide. Berry 9–15 × 9–15 mm, blue-black to purple. Seeds 0.5–0.7 mm long, obliquely pyramidal.

**Figure 103.**
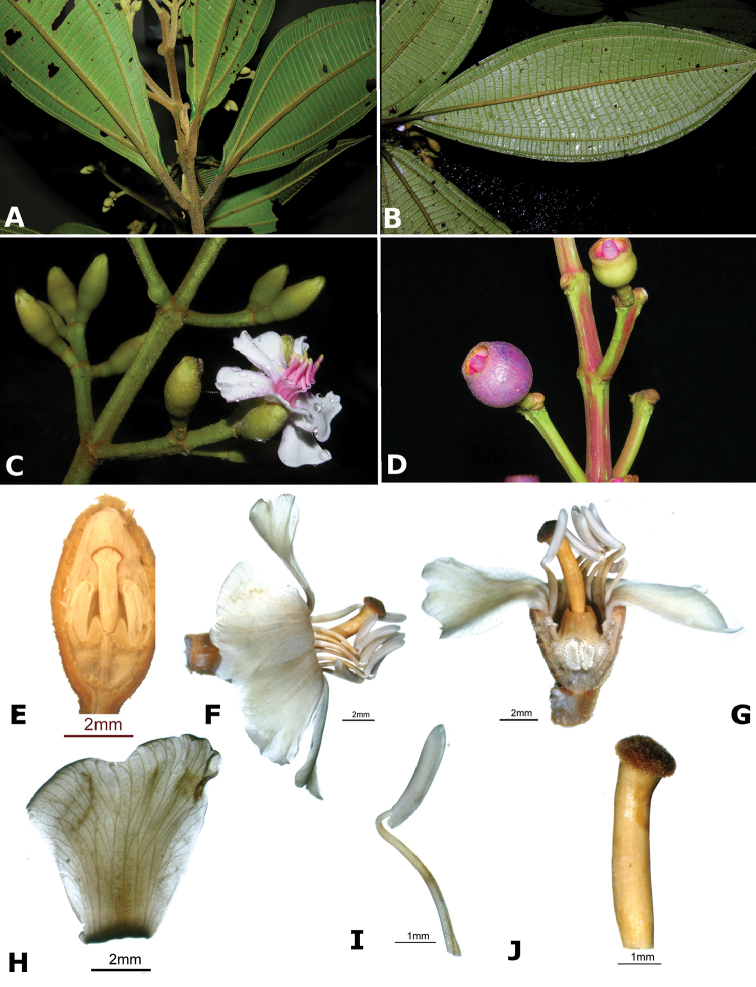
*Conostegia rufescens*. **A** Habit and morphotype with revolute leaf base **B** Leaf abaxial surface **C** Flower at anthesis **D** Berry **E** Longitudinal section of a flower bud **F** Pickled flower at anthesis **G** Longitudinal section of a flower at anthesis **H** Petal **I** Stamen **J** Style. Photograph **A** from voucher *R. Kriebel 5635* **B, C** from *R. Kriebel 5314* **D** by E. Salicetti **E–J** from *R. Kriebel 5687*.

#### Distribution

**Figure 104.**
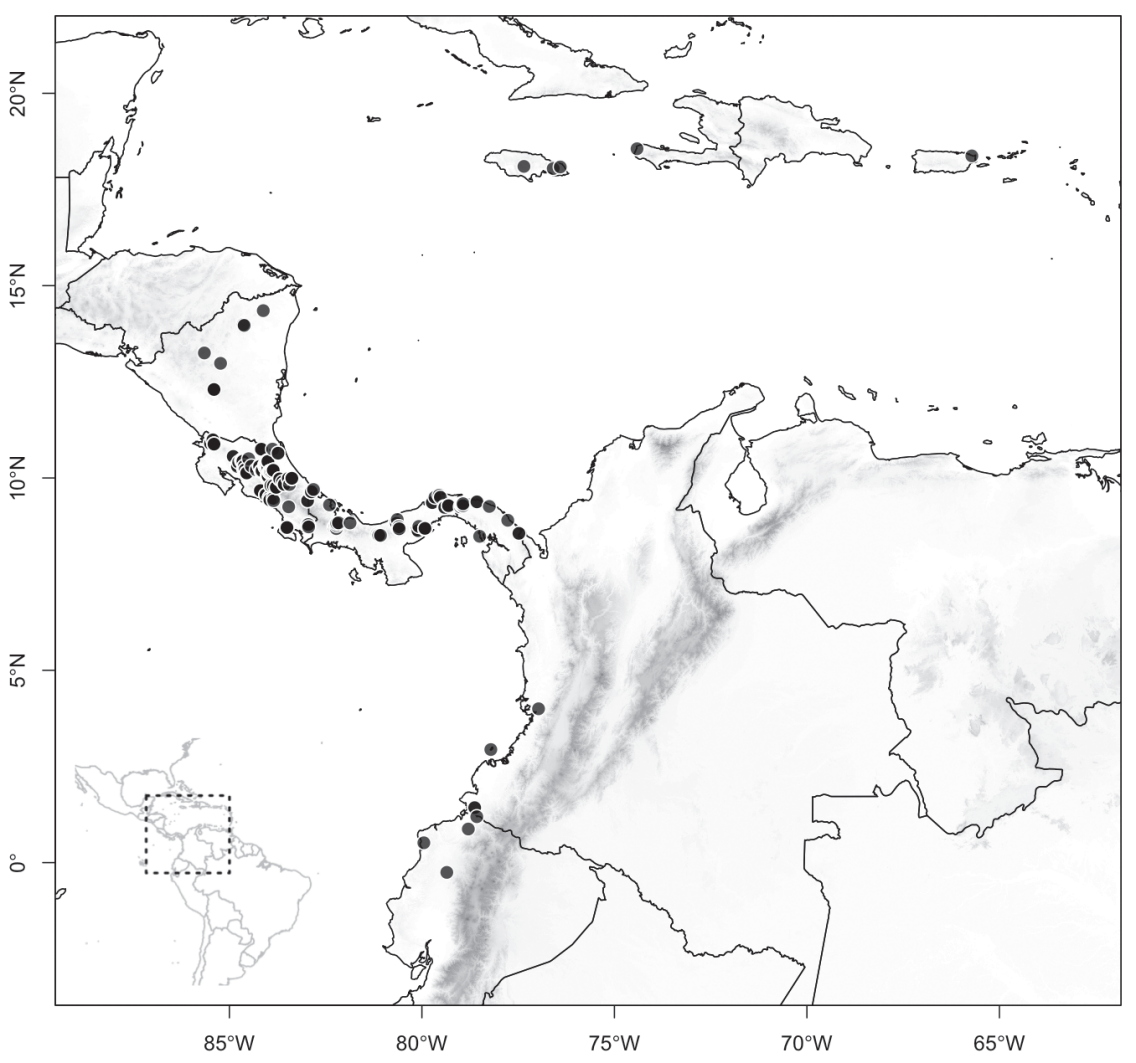
Distribution of *Conostegia rufescens*.

(Fig. 104). In the mainland ranging from Nicaragua through Costa Rica and Panama to Colombia and Ecuador, in the Caribbean known from Dominican Republic, Jamaica, Haiti, and Puerto Rico, from sea level to 1700 m elevation.

*Conostegia rufescens* can be recognized by its dense but short indument of mainly small brown dendritic trichomes covering floral buds and veins on the abaxial leaf surface. This species is variable in habit, the amount of indument on the leaves and the shape of the leaf base. In Costa Rica for example, one can find shrubs to small trees in Braulio Carrillo National Park at 500 meters elevation with acute leaf bases and in the same park at 1500 meters tall trees with decurrent leaf bases. *Conostegia rufescens* can be easily confused with *Conostegia centronioides* and *Conostegia rubiginosa* on the basis of the rusty indument. The latter two species differ from *Conostegia rufescens* in their exserted styles. Schnell (1996) noted limited local flowering seasons and differentiation between population in flowering time due to local adaptation. Schnell (1996) also studied a population in Alto La Palma, Costa Rica, and noted brief and concentrated flowering peaks, more so than he observed in other species such as *Conostegia macrantha*, *Conostegia montana*, *Conostegia oerstediana*, and *Conostegia rhodopetala*. Lastly, Schnell (1996) noted the earlier flowering season of populations at lower elevations and a later flowering season for more higher elevation populations. *Conostegia formosa* Macfad. from Jamaica was considered by Schnell (1996) as conspecific with *Conostegia rufescens* based on Macfadyen’s description. Schnell (1996) argued that if Macfadyen’s treatment were accepted, *Conostegia formosa* would be the oldest name available.

#### Specimens examined.

**HAITI** (fide Schnell). Riviere Glace, Holdridge 2120 (MICH, US).

**JAMAICA. Portland**: Vicinity of Moody’s Gap, Britton 3392 (NY). **Westmoreland**: Copse Mt. Woods c. 1 mile southwest of Rat Trap, Proctor 21476 (NY).

**PUERTO RICO. Luquillo**: Northeastern Luquillo Mts. Rd. 191, Woodbury s.n. (NY).

**NICARAGUA** (fide Schnell). **Jinotega**: Cordillera Isabelia, Macizos de Peñas Blancas, Neill 7180 (GH). Matagalpa: N & NW sides of Cerro Musun above Salto Grande de Q. Negra, Río Bilampi, Neill 1797 (MO). **Zelaya**: 20 km O de Awas Tingi, S. de Rio Wawa, Little and Delvis 23354 (US).

**COSTA RICA. Alajuela**: La Palma de San Ramón, Brenes 4401, 5688, 6283, 16203 (CR, NY); Cerro de “La Muralla” de San Ramón, Brenes 5688 (NY); San Carlos, Fortuna, R. B. Arenal, Mundo Aventura, Rodríguez 8606, 8830 (INB, NY); Villa Quesada, Cantón de San Carlos, Smith 2556 (NY); 3.5 km west of Fortuna, 2.5 km northeast of New Volcan Arenal along sloping base, Taylor and Taylor 11625 (NY); La Palma, Tonduz 12434 (NY). **Cartago**: P.N. Tapantí, sendero Oropéndula, ca. 10 mfrom river, Penneys and Blanco 1792 (FLAS, NY). **Limón**: Quebrada González, sendero Las Palmas, Kriebel 1422, 5314 (INB, NY); 6 miles inland from mouth of Estrella River, Stork 4614 (NY); Talamanca, Fila Carbón, Buena Vista, Finca Corredor Biológico, Valverde 13 (CR, NY). **Puntarenas**: Forested slopes east of Las Cruces and 5 to 6 km south of San Vito on and around the property of Mr. Robt. Wilson, Burger and Matta 4470 (F, NY); R.B. Monteverde, Cordillera de Tilarán, Pacific slope wet forest road to Continental divide, Haber 11514 (INB, MO, NY). **San José**: Tarrazú, San Lorenzo, Estribaciones sureste de cerro Toro, Estrada et al. 684 (CR, NY); Límite del P. N. Braulio Carrillo, 2 Km después del peaje hacia Guápiles, Kriebel 1938 (INB, NY); Pérez Zeledón, Río Nuevo, El Brujo, 0.5 km NO del Andaribel del Brujo, Rodríguez et al. 6974 (INB, CR, NY); San Isidro de Dota, albergue Tinamú, Santamaría and Morales 813 (INB, NY).

**PANAMA. Bocas del Toro**: Cricamola Valley, Cooper 486 (NY); Buena Vista Camp on Chiriquí trail, Cooper 581 (NY); Along road towards Chiriquí Grande along trail leaving road, McPherson 12553 (MO, NY). **Coclé**: Vicinity of La Mesa, Croat 13325 (F, MO, NY); La Mesa, Gentry 5636 (MO, NY); Vicinity of La Mesa above El Valle, Gentry 7427 (MO, NY). **Veraguas**: Along road on Pacific slope 1-3 km above Escuela Agricola Alto Piedra, Croat 25996 (NY); Area between La Junta and Limón, 5 hours walk north of Alto Calvario, Folsom 5877 (MO, NY). **Colón**: Around Dos Bocas, Río Fató valley, Pittier 4218 (NY).

**COLOMBIA. Nariño**: Espriella, Tumaco, Romero-Castañeda 2801 (NY). **Pichincha**: 20 km W of Santo Domingo de los Colorados, Cazalet 5110 (NY).

**ECUADOR. Esmeraldas**: Parroquia Mataje, Reserva Etnica Awá, Centro Mataje, Aulestia et al. 442 (MO, NY, QCNE); Parroquia de Concepción, bank of Río Santiago, above Playa Rica, Mexia 8471 (NY); Eloy Alfaro, Reserva Ecológica Cotacachi-Cayapas, Parroquia Luis Vargas Torres, Río Santiago, Estero Angostura, Tirado et al. 647 (MO, NY, QCNE).

### Conostegia setifera

Taxon classificationPlantaeMyrtalesMelastomataceae

Standl.

Fig. 105

Conostegia setifera Standl., Field Mus. Nat. Hist., Bot. ser. 18: 805. 1938. Type: Costa Rica. Alajuela: Camino de la Finca Johanson, Los Angeles de San Ramón, 15 March 1928, A. Brenes 6041 (holotype: F!, isotypes: CR!, NY!).

#### Description.

Trees 4–12 m tall with tetragonal and ridged stems that are sparsely to densely setose with stramineous hairs 2–5 mm long, with or sometimes replaced by a dense but inconspicuous puberulent understory; the nodal line present and bearing coarse setae. Leaves of a pair equal to somewhat unequal in length. Petiole 1.8–7.8 cm. Leaf blades 8–27 × 4–13.5 cm, 3–5 plinerved, with the innermost pair of veins arising 0.5–2 cm above the base in opposite to mostly alternate fashion, elliptic to obovate, the base acute or obtuse, the apex acute to obtuse or rounded, the margin denticulate and often ciliate, the adaxial surface glabrous, the abaxial surface glabrous except for some pubescence on the nerves. Inflorescence a terminal panicle 4–17.3 cm long with flowers frequently congested at the end of small branches, branching above the base, accessory branches present, the rachis covered mostly with stellate trichomes, bracts to 3 cm long, linear or setulose, deciduous or if setulose persistent, bracteoles replaced by clusters of setae subtending the buds, the setae persistent. Pedicels to 3 mm. Flowers 7–9 merous, calyptrate. Floral buds 6–10 × 6–10.5 mm, globose, the base rounded to truncate, the apex rounded and short-mucronate, not constricted, hypanthium 4.5–5 × 6–8 mm, stellate pubescent and tuberculate. Petals 8–11 × 8–11 mm, white, obovoid, spreading, apically emarginate, glabrous. Stamens 26–31, 6–8 mm long, radial to slightly bilateral resulting from stamens getting stuck below the stigma, the filaments 3.5–4.5 mm long, white, anthers 2.5–3.5 × ca. 1 mm, elliptic, the base sagittate, laterally compressed, yellow, the pore ca. 0.15 mm wide, terminal. Ovary 13–18 locular, inferior, apically glabrous and forming a collar around the style. Style ca. 5 mm long, bending downwards during anthesis and protruding below the anthers, vertical distance frome the anthers to the stigma ca. -2 – 0 mm, horizontal distance ca. 0.5–2 mm; stigma capitate, consisting of 13–18 laterally compressed lobes, ca. 4 mm wide. Berry 10–12 × 10–12 mm, purple black. Seeds ellipsoid, the testa smooth.

**Figure 105.**
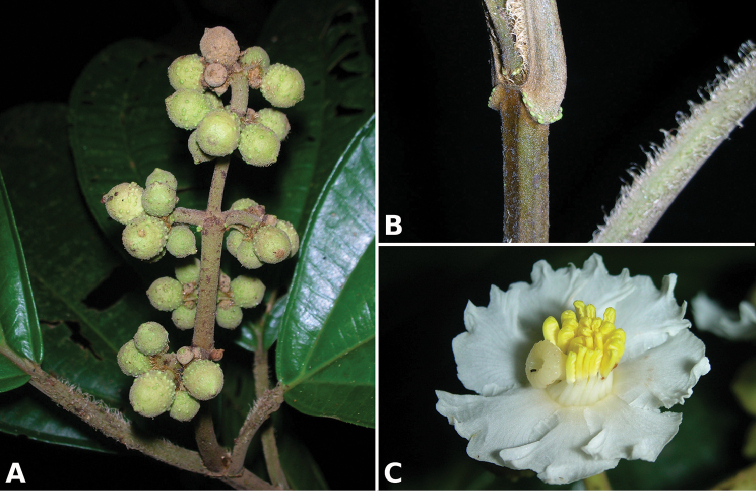
*Conostegia setifera*. **A** Inflorescence **B** Lenticellate node **C** Flower at anthesis. Photos by E. Salicetti.

#### Distribution

**Figure 106.**
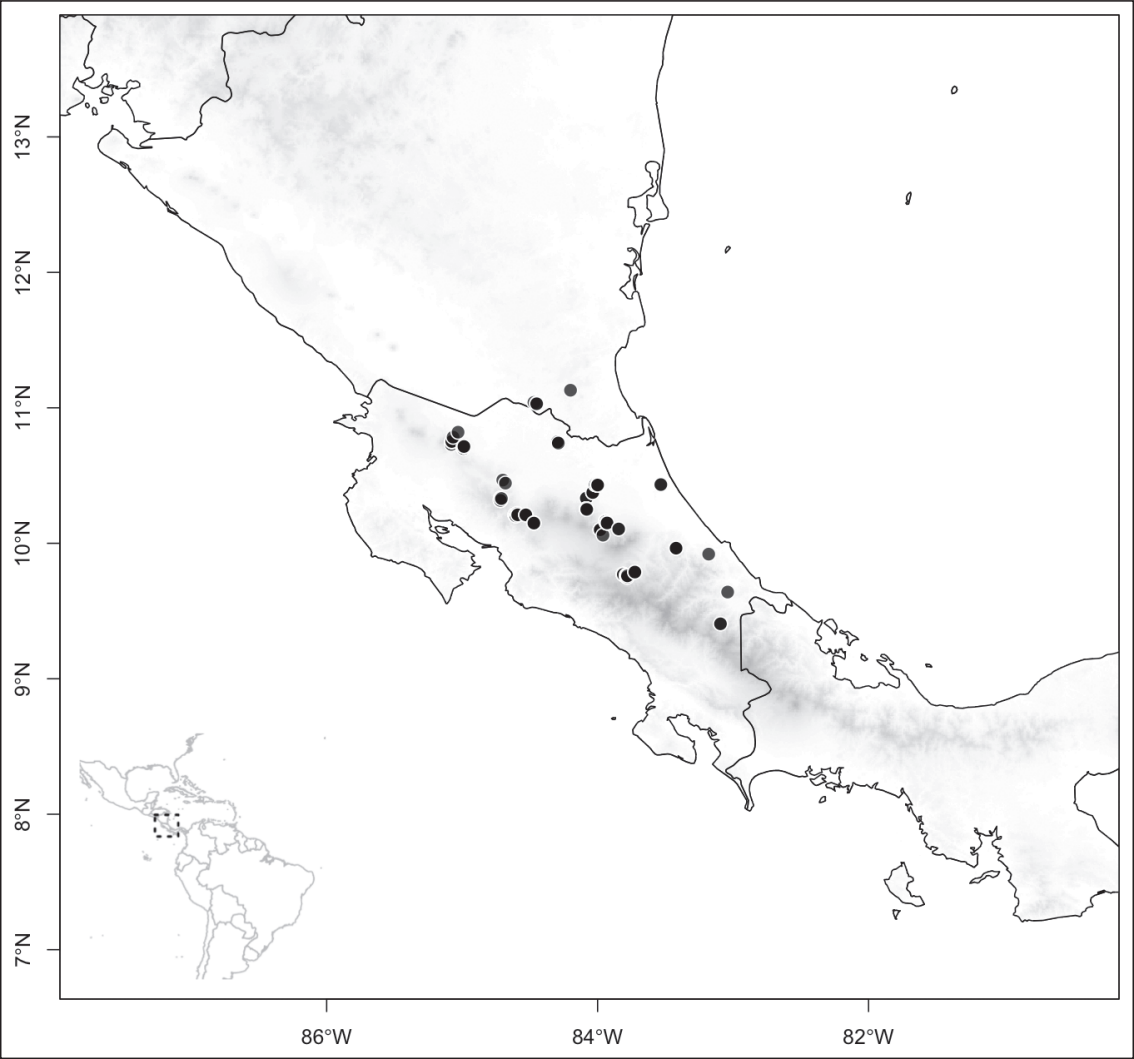
Distribution of *Conostegia setifera*.

(Fig. 106). Ranging from Nicaragua to Costa Rica, 0–1350 m elevation.

*Conostegia setifera* is distinguished by its setose petiole adaxial surface, setose bracteoles and sessile to subsessile, spherical flower buds. Specimens of *Conostegia setifera* have been confused with *Conostegia lasiopoda* based on the setose indument on the petioles. One way to differentiate them with infertile material is that *Conostegia lasiopoda* is consistently basinerved whereas *Conostegia setifera* is plinerved. With fertile material they are unmistakable since *Conostegia lasiopoda* has a long exserted style with a capitate stigma whereas *Conostegia setifera* has a large crateriform lobed stigma, and its style is not exserted. This species was reported from Panama from the specimen *Mori et al. 3850* (at MO and reportedly at WIS) in both Schnell (1996) and Almeda (2009) but this specimen was also cited as *Conostegia dentata* in both of the latter publications. I have not been able to locate the specimen *Mori et al. 3850* to confirm its identification. This species is usually encountered in the forest understory and Schnell (1996) noted that within this habitat, only well lit branches produce flowers.

#### Specimens examined.

**NICARAGUA** (fide Schnell). **Río San Juan**: near Caño Chontaleño, 20 km NE of El Castillo, Neil and Vincelli 3519 (MO); 1 km E de Río Sábalos, Moreno 23190 (MO); boca del Sábalo, camino a Buena Vista, Moreno 25625 (MO).

**COSTA RICA. Alajuela**: Los Angeles de San Ramón, Brenes 13582 (CR, NY); Cataratas de San Ramón, Brenes 13652 (CR, NY); Eastern slopes of Volcán Miravalles west of Bijagua near the Río Zapote, Burger et al. 11709 (CR, NY); Guatuso, P. N. Volcán Tenorio, Cuenca del Río Frío, El Pilón, Chaves and Muñoz 436 (CR, INB, NY); San Ramón, Villa Blanca, González 680 (INB, NY); N side of Volcan Arenal, Lent et al. 3311 (NY); along trail from macadamia village SE around base of Cerro Chato to Catarata de Fortuna, Smith 10900 (CR, NY). **Heredia**: Finca La Selva, the OTS Field Station on the Río Puerto Viejo just E of its junction with the Río Sarapiquí, Holdridge trail 1800 m line, Folsom 9139 (MO, NY); along Río Peje about .5 km SW of back end of Vargas property, aprox. in the area where an imaginary line drawn between Magsasay (colonia penal) and Puerto Viejo de Sarapiquí would cross the Río Peje, Hammel 11216 (MO, NY). **Limón**: Reserva Biológica Hitoy Cerere, rainforest along the sloping banks of Río Cerere from the Reserve Station to the big waterfall, Almeda et al. 6841 (CR, MO, NY); Hacienda Tapezco-Hda. La Suerte 29 air km W of Tortuguero, Davidson 6968 (NY). **San José**: Sendero La Montura, entre Estación Quebrada González y Estación Zurquí, Rodríguez et al., 5799 (INB, NY).

### Conostegia setosa

Taxon classificationPlantaeMyrtalesMelastomataceae

Triana

Fig. 107

Conostegia setosa Triana, Trans. Linn. Soc. London 28: 99. 1872. Type: Colombia. Chocó: Cordillera Occidental, between Tuquerres and Barbacoas, 1851–1857, J. Triana 3940 (holotype: BM!, isotypes: BR!, K!, P (fide Almeda in Schnell 1996), W).
Cryptophysa setosa Standl. & J. F. Macbr., Field Mus. Publ. Bot. 4: 244. 1929. Type: Panama. Bocas del Toro: Buena Vista, Almirante, January-March 1928, G. Cooper 219 (holotype: F!, isotype: NY!). Conostegia hirsuta Gleason, Phytologia 3: 359. 1959. Non Conostegia setosa Triana.

#### Description.

Shrubs to less commonly small trees 0.9–1.5(-3) m tall with terete to a somewhat tetragonal stems that are covered with long smooth spreading hairs and a sparse and inconspicuous ground layer of brown lepidote hairs; the nodal line obscured and covered by the setae as the rest of the node and internode. Leaves of a pair equal to unequal in length. Petiole 0.2–3.9 cm. Leaf blades 7.6–35.5 × 3.22–13.5 cm, usually clustered at the apex of the branches, 5–7 plinerved, with the innermost pair of primary veins diverging from the mid vein up to about 4 cm above the base usually after the formicarium mostly opposite fashion, elliptic to obovate, the base acute and attenuate or rounded and with formicarium 1.5–3 cm long entirely on the leaf blade when the base is decurrent ot half of the formicarium on the petiole when not, the apex acute to abruptly acuminate, the margin denticulate to dentate, setose on both surfaces. Inflorescence a terminal panicle 3.4–16.3 cm long branched above the base but sometimes appearing branched at the base because of multiple inflorescences arising at opposing meristems at the terminal node, accessory branches present, the rachis setose with green or red trichomes, bracts subtending the nodes up to 3 cm long, persistent or deciduous, bracteoles up to 1 cm long, linear, persistent. Pedicel 0.5–3 mm. Flowers (4-)5(-6) merous, obovate to pyriform, calyptrate, the floral buds 4–7 × 2–4 mm, the base rounded, the apex apiculate, slightly constricted; the hypanthium 2.35–3.5 × 2–3 mm, setose with green or red trichomes and tiny brownish glands to rarely glabrescent. Petals 6–7 × 4–5 mm, white to pale pink, broadly obovate, spreading, eventually closing and persisting closed, emarginate, glabrous. Stamens (13-)15(-17), 4–5.5 mm long, radially arranged but sometimes bilaterally symmetric or asymmetric apparently from interactions with the style, the filament 2.45–2.75 mm long, white, anthers 2.25–2.75 × 0.5–0.75 mm, linear and sinuous, laterally compressed, the base sagittate, yellow, the pore ca. 0.15 mm wide, ventro terminal. Ovary (4-)5(-7) locular, inferior, apically glabrous and forming a low collar around the style. Style 4–5 mm long, straight and just slightly curved upward apically, vertical distance of the anther pore to the stigma -2 – 0 mm, horizontal distance absent; stigma capitellate to subcapitate, 1–1.5 mm wide. Berry 5–6 × 5–6 mm, dark purple to black. Seeds 0.3–0.5 mm, ovoid, the testa smooth.

**Figure 107.**
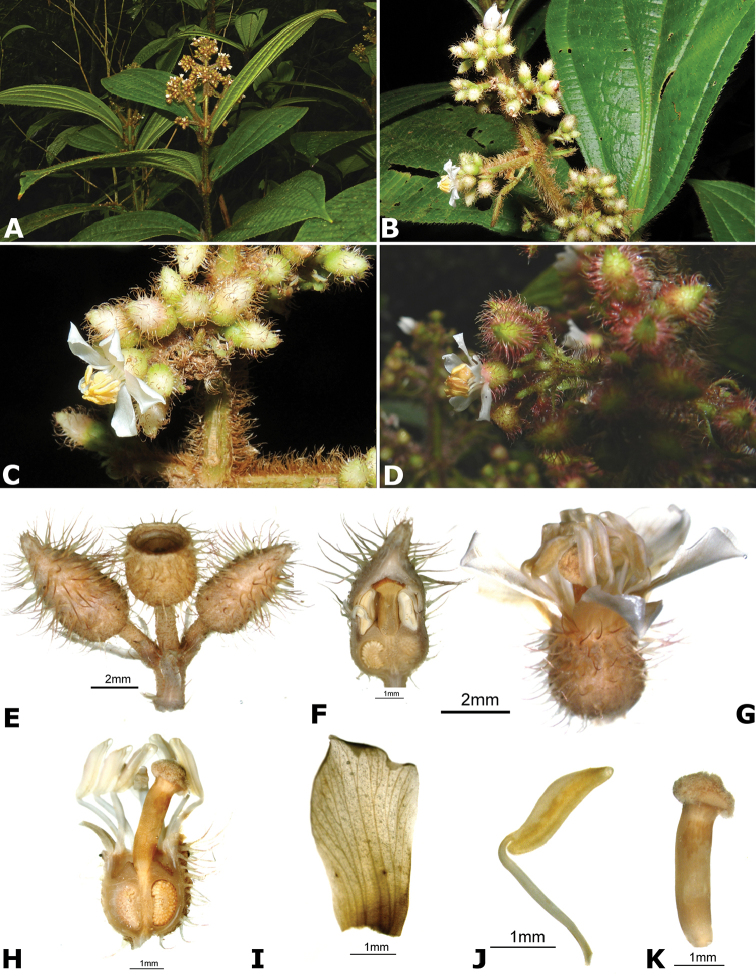
*Conostegia setosa*. **A** Habit **B** Inflorescence and leaf base showing formicaria **C** Close up of flowers **D** Close up of morphotype with red indument **E** Flower buds and maturing fruit in the middle **F** Longitudinal section of a flower bud **G** Pickled flowers at anthesis **H** Longitudinal section of a flower at anthesis **I** Petal **J** Stamen **K** Style. Photos **A, D–K** of specimen voucher *R. Kriebel 5731* **B–C** from *R. Kriebel s. n*.

#### Distribution

**Figure 108.**
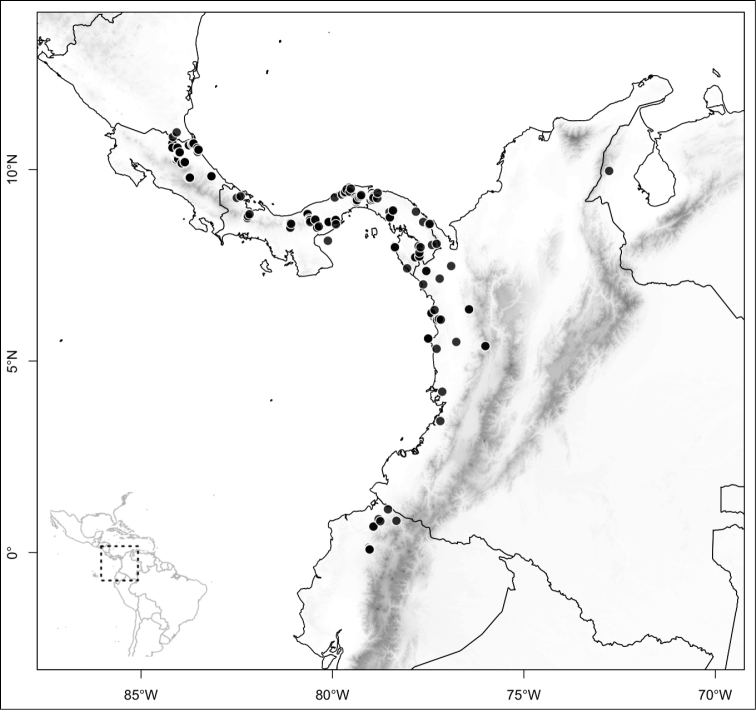
Distribution of *Conostegia setosa*.

(Fig. 108). From Nicaragua through Costa Rica and Panama to Colombia and Ecuador, 0–1400 m elevation.

This is one of the most distinctive species of *Conostegia* because of the densely setose indument on most parts and the presence of pouch formicaria at the base of the leaf. Only one additional species has this kind of structure within *Conostegia* and that is *Conostegia dentata*. The latter taxon differs from *Conostegia setosa* in its reduced inflorescences, larger flowers, and exserted styles. Two morphotypes exist in *Conostegia setosa*, with typical plants having almost sessile leaves with mostly acute to attenuate bases in which the formicarium is almost all on the lamina. On the other hand plants described by Standley and Macbride as *Cyprophysa setosa* and given the new name of *Conostegia hirsuta* by Gleason have long petioles with the formicarium placed half on the petiole and half on the lamina. Schnell (1996) considered these two morphotypes to be the same species because he saw intermediate morphologies. The latter author also observed no geographic pattern but noted the similarity between plants from the same locality. During the course of this study plants of both morphotypes were collected in the same locality in Santa Fé, Veraguas, Panama (short petiolate leaves with acute to attenuate bases in *Kriebel and Burke 5731*, petiolate leaves with rounded bases in *Kriebel and Burke 5712*- both at NY, PMA).

Schnell (1996) studied the phenology of this species in detail and observed one or two flowers opening everyday for two to three months. He hypothesized that this behavior might result in a greater degree of outcrossing by forcing the bees to forage to other plants in these large clonal populations. He further noted that bee species that visit *Conostegia setosa* are non traplining opportunistic species (Schnell 1996). I have observed one of these opportunistic bees in the Halictidae family visiting *Conostegia setosa* at La Selva, Costa Rica. Perhaps as expected for a species that flowers over a long period of time, fruiting is also spread through large periods of time (Schnell 1996).

Alonso (1998) studied populations of *Conostegia setosa* in Costa Rica and Panama and observed some populations in Colombia and Ecuador. She found that in the southern part of it distribution, *Conostegia setosa* was inhabited by more specialized ants and one obligate inhabitant, *Pheidole melastomae*. The latter inhabitant was most common in South America so Alonso (1998) hypothesized that perhaps this pattern is due to the fact that because *Conostegia setosa* is bird dispersed, plants have dispersed more rapidly than their obligate ant inhabitant *Pheidole melastomae*. In general, Alonso (1998) found a lot of variation in the ant inhabitants of *Conostegia setosa*.

#### Specimens examined.

**COSTA RICA. Alajuela**: San Carlos, Boca Tapada, Laguna de Lagarto Lodge, Solano 894, 1448 (INB, NY). **Cartago**: Jiménez, Pejibaye, Reserva El Copal, Kriebel 2474 (INB); Jiménez, Pejibaye, Refugio de Vida Silvestre La Marta, Kriebel 4484 (INB); **Heredia**: La Selva, Sendero Holdridge a Parcelas, Kriebel 3565 (INB). **Limón**: North end of Tortuguero National Park and near the Boca de las Lagunas de Tortuguero, Burger and Antonio 11274 (CR, F, NY); Between Cerro Jacrón and Cerro Bitárkara, Trail between “Sitio Rangalle and Cuen”, Hazlett 5125 (NY); Pococí, R. B. Bosque Lluvioso, Sendero derecho, Vargas et al. 3539 (INB, NY).

**PANAMA. Coclé**: 7 km from Llano Grande on road to Coclesito near Continental Divide, Antonio 1365 (NY); Vicinity of La Mesa, N of El Valle de Antón, along steep slopes above water reservoirs, ca. 1 km W of road between Finca Mandarinas and Finca Furlong, Croat 67169 (MO, NY); Parque Nacional Omar Torrijos, Sendero Cuerpo de Paz, Penneys and Blanco 1760 (FLAS, NY). **Darién**: 0-2 mi. E of Tres Bocas along shortest headwater of Río Coasi, Kirkbride and Duke 1170 (MO, NY); Mannene to the mouth of Río Coasi, Kirkbride and Bristan 1492 (MO, NY); Ensenada Guayabo, between Punta Guayabo Grande and Punta Guayabo Chiquita, Stern and Chambers 178 (NY). **Panamá**: On Atlantic side Llano-Carti Road, 12 miles from Pan-American Highway, Antonio 3308 (MO, NY); Road to Carti (San Blas), 19 km north of El Llano, Busey 894 (MO, NY); Near summit of Cerro Camapan, Croat 22814 (MO, NY); Cerro Azul, D’Arcy and D’Arcy 6233 (MO, NY); Cerro Jefe, Dwyer and Gentry 10257 (MO, NY); On trails radiating from end of road which passes Campana water tank near Cerro Campana, Kirkbride and Hayden 311 (MO, NY); along El Llano Carti-Tupile road, 12 mi above Pan-Am Hwy, Liesner 1132 (MO, NY); Forest 10 miles north of Highway 1 towards Cerro Jefe, Luteyn 1323 (MO, NY); Cerro Campana, Sendero La Cruz, near the summit, Penneys and Blanco 1678 (FLAS, NY).

**COLOMBIA. Chocó**: North ridge of Alto de Buey, above Dos Bocas del Río Mutatá, tributary of Río El Valle, ESE of El Valle, Gentry and Fallen 17413 (MO, NY); Nuquí, Alto de Buey, von Sneidern 7 (NY). **El Valle**: Cordillera Occidental, vertiente occidental, hoya del río Anchicayá, lado derecho, bosques entre Pavas y Miramar, Cuatrecasas 14380 (NY); Costa del Pacífico, río Cajambre, Silva, Cuatrecasas 17555 (NY).

**ECUADOR. Esmeraldas**: Eloy Alfaro, Reserva Ecológica Cotacachi-Cayapas, Parroquia Luis Vargas Torres, Río Santiago, estero Pote, Tirado et al. 529 (MO, NY). **Pichincha**: Carretera Quito-Puerto Quito km 113, Betancourt 82, 111, 166, 169, 218 (NY); Carretera Quito-Puerto Quito km 113, 10 km al Norte de la carretera principal, Freire 1060 (NY); Reserva Forestal ENDESA, Río Silanche: “Corporación Forestal Juan Manuel Durini”, km 113 de la carretera Quito-Pto. Quito, faldas occidentales a 10 km al Norte de la carretera principal, Jaramillo 5202, 6412 (NY).

**VENEZUELA** (fide Schnell). **Zulia**: Caño Helena, Sierra Perijá, Delascio and Benkowsky 3191 (US).

### Conostegia superba

Taxon classificationPlantaeMyrtalesMelastomataceae

Naudin

Fig. 109

Conostegia superba Naudin, Ann. Sci. Nat. Bot. ser. 3. 16: 108. 1850. Type: Jamaica. Wilson s.n. (holotype: P, isotype: K!; Schnell (1996) cites a fragment in F).
Conostegia macrophylla Naudin, Ann. Sc. Nat. ser 3 16: 112. 1850. Type: Mexico. “In montibus Mex. prope Oaxaca and Chinantla”, 700 m, April–November 1840, H. Galeotti 2941 (isotype: BR!).
Conostegia alternifolia Macfad., Fl. Jamaica 2: 71. 1850; nom. inval.
Conostegia clidemioides Wright ex Grisebach, Cat. PI. Cuba 98. 1866. Type: Cuba. La Perla, eastern Cuba, 1861, C. Wright 2503 (holotype: GOET!; isotypes: BM!, BR, GH!, K!, LE, MO!, S!).
Conostegia poeppigii Cogn., Mart. Fl. Bras. 14(4): 211. 1886. Type: Peru. Provo Maynas, Poeppig s. n. (lectotype: LE, designated here; isolectotype: ?BR). Other syntype: Colares, Provo Para, Brazil, June 1832, Poeppig s. n. (W). “The Brazilian syntype is sterile and cannot be identified with certainty” Schnell (1996).
Conostegia purpusii Brandegee, Univ. Calif. Publ. Bot. 6: 57. 1914. Type: Mexico. Chiapas: Finca Mexiquita, July 1913, C. Purpus 6784 (holotype: UC!; isotypes: A, BM!, CAS!, F!, GH, MO!, NY!, US). “This collection is mixed with a species of Miconia in many herbaria.” Schnell (1996)
Conostegia pentaneura Standl., Field Mus. Publ. Bot. 8: 146. 1930. Type: Honduras. Lancetilla valley near Tela, 100 m, 8 August 1929, F. Salvoza 875 (holotype: A!, photograph: GH).
Miconia bailloni Gomez, Anal. Hist. Nat. Madrid 23: 69. 1894. Nom. inval. (no specimen cited)

#### Description.

Shrubs to small trees 1–8 m, tetragonal and sulcate stems that are glabrous or beset with sessile stellate trichomes or densely covered with stipitate stellate trichomes; the nodal line present but inconspicuous. Leaves of a pair equal to somewhat unequal in length. Petiole 0.7–10.8 cm. Leaf blades 7.1–36 × 2.3–16 cm, 5-plinerved, with the innermost pair of primary veins diverging from the mid vein up to 1.5 cm above the base in opposite to sub opposite fashion, narrowly ovate to broadly ovate, or oblong-elliptic, the base acute to rounded, the apex acute to obtuse and acuminate, the margin entire, undulate-ciliate, or dentate, the adaxial surface glabrous or with simple hairs in young leaves, the abaxial surface glabrous or glabrescent with sessile stellate trichomes, to evidently pubescent with stipitate stellate trichomes especially on the veins. Inflorescence a terminal panicle with the flowers disposed in umbels terminating the inflorescence branches, 7–27.5 cm long and branching above the base but sometimes appearing branched at the base because of multiple inflorescences arising at opposing meristems at the terminal node, accessory branches present, rachis often reddish, sul-cate, bracts and bracteoles 1 mm or less, early deciduous. Pedicels thick, 1.5–5.5 mm long. Flowers (4-)5–7 merous, oblong to obovate-pyriform, calyptrate, floral buds 5–9 × 2.5–5 mm, the base rounded, the apex acute to slightly apiculate, slightly to not constricted at the middle, the hypanthium 2.5–3 × 2.25–2.75 mm, glabrous. Petals 4–6.5 × 3.5–6 mm, white or less commonly pink, obovate or obtriangular, spreading at anthesis, emarginate, glabrous. Stamens (10-)14–16(-17), anthers 5–7.5 mm long, slightly zygomorphic, the filament 3–4.25 mm long, not geniculate, white, anthers 2–3.25 × 0.5–1 mm, linear-oblong, slightly recurved, laterally compressed, briefly sagittate at the base, yellow, the pore 0.1–0.15 mm wide, subterminal and ventrally inclined. Ovary (4-)5–6(-9) locular, inferior, apically glabrous and forming a collar around the style. Style 3–5.25 mm, enveloped at the base by a collar, straight or slightly bent towards the tip, vertical distance between the anther pore and the stigma ca -1 – 0 mm, horizontal distance absent, the stigma subcapitate, 1–1.5 mm wide. Berry 6–9 × 6–9 mm, purple-black. Seeds 0.4–0.6 mm long, narrowly pyramidal, the testa smooth.

**Figure 109.**
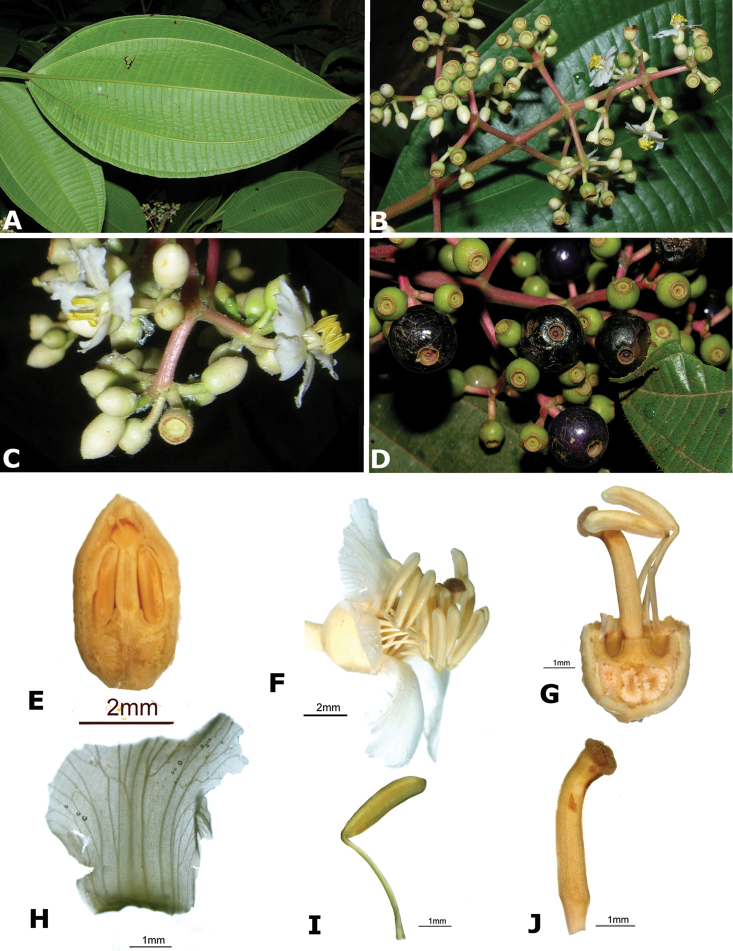
*Conostegia superba*. **A** Leaf abaxial surface **B** Inflorescence **C** Close up of the flower **D** Infructescence **E** Longitudinal section of a flower bud **F** Pickled flower at anthesis **G** Longitudinal section of a flower at anthesis wit petals and most stamens removed **H** Petal I Stamen **J** Style. Photos of **A–C** and **E–J** from specimen vouchered *R. Kriebel 5582* **D** taken by Reinaldo Aguilar.

#### Distribution

**Figure 110.**
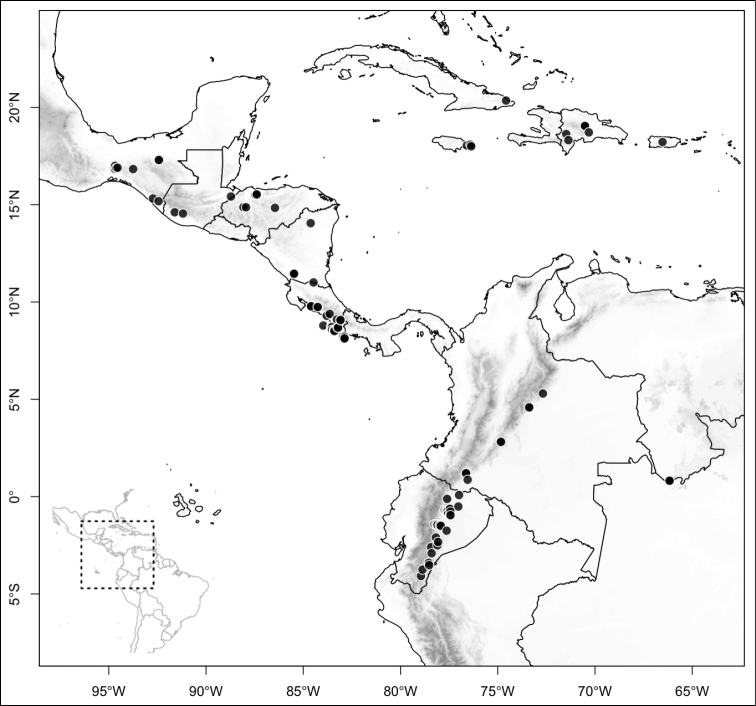
Distribution of *Conostegia superba*.

(Fig. 110). In the mainland from Mexico through most of Central America, restricted to the Pacific coast of Costa Rica and in Panama to the western portion. In South America in Colombia, Ecuador, and Venezuela. Schnell (1996) also reports this species from Peru, 0–1700 m in elevation.

The recent molecular phylogeny of *Conostegia* included three samples of *Conostegia superba*. One, from the Dominican Republic, the second from Guatemala, and the third from Ecuador. Two of the samples, the one from Guatemala and the one from the Dominican Republic fell sister to each other in a clade that also includes *Conostegia bracteata* and *Conostegia caelestis*. The third specimen from Ecuador, falls sister to *Conostegia rhodopetala*. On the one hand, this confirms the position of Schnell (1996) of treating *Conostegia clidemioides* as a synonym of *Conostegia superba* but on the other hand underlines the species delimitation problems between *Conostegia superba* and *Conostegia rhodopetala*. Also, although genetically *Conostegia cuatrecasii* appears to be a distinctive species based on the Panamanian and Ecuadorian samples included in the phylogeny, morphologically the populations of pink flowers of *Conostegia superba* can resemble *Conostegia cuatrecasii*. Schnell (1996) notes more limited flowering seasons in local populations than are reflected by specimens. A Brazilian infertile specimen which corresponds to a syntype of *Conostegia poeppigii* cannot be positively identified (Schnell 1996) and is thus excluded from the distribution circumscription until it is collected again. Schnell (1996) noted that some isotypes of *Conostegia purpusii* are mixed with a species of *Miconia* in many herbaria.

#### Specimens examined.

**CUBA. Oriente**: El Yunque, Ekman 3970 (NY); La Prenda, Hioram and Maurel 4776 (NY).

**DOMINICAN REPUBLIC. Barahona**: Sierra del Bahoruco, Municipio Paraíso La Víbora, Clase, Montilla and Schuber 4383 (NY); Fuertes 969 (NY). **Bonao**: subida al Casabito, Liogier 30328 (NY). **Monseñor Nouel**: Road up to Alto Casabito, ca. 8 km W of jct. with Highway Duarte on road from Bonao to Constanza, Judd and McDowell 6523 (NY); Cordillera Central on road to Alto Casabito ca. 2.8 km W on highway from Autopista Duarte N of Bonao to El Rio and Constanza, Judd et al. 8218 (NY); Cordillera Central, “Zumbador”, 1.5 km al sudeste de Juan Aldian, en la confluencia de los rios Zumbador and Calle Estrecha, Zanoni et al. 31550 (NY).

**PUERTO RICO. Cordillera Central**: Toro Negro Forest, in Vereda del Bolo, Vives 138 (NY).

**JAMAICA. Portland**: Upper Swift River Study Site of Ecological Survey, Blue Mt. Multipurpose Project near Mossman’s Peak, Bretting 61, 254 (NY); North side of Cuna Cuna Pass, Harris and Britton 10560 (NY); Foothills of the John Crow Mts. Along road between Fair Prospect and Hartford and Ecclesdown, ca. 3.9-4 mi SE of jct. with Rt. A-4 (coastal road), Judd 5327 (NY). **St. Thomas**: Banks Devil’s River, Britton 3582 (NY); near Milepost 18 southeast of Bowden Pen. upper valley of the Río Grande, Proctor 26603 (NY).

**MEXICO** (fide Schnell). **Chiapas**: crest of ridge 3 km E of Francisco Madero, NE of Cintalapa, Breedlove 38701 (MO). **Oaxaca**: entre Puerto Eligio y Comaltepec, km 149 entre Tuxtepec a Oaxaca S. Juarez, Martinez Calderon 388 (GH, MICH, UC, US). **Veracruz**: Lado SE de Laguna Catemaco, arriba de Rio Cuetzalapan, Beaman 5165 (GH).

**GUATEMALA. Izabal**: Sierra Caral, camino de terracería cerca de la entrada a la Finca La Firmeza, desde Morales, Kriebel et al. 5582 (USCG, NY). **Sololá**: south-facing slopes of Volcán Atitlán above Finca Mocá, Steyermark 47891 (NY).

**HONDURAS. Cortés**: entre Agua Azul y Pito Solo Lago de Yojoa, Molina 7326 (NY).

**NICARAGUA** (fide Schnell). **Rivas**: Isla Ometepe, Volcán Maderas, Hacienda La Argentina, Robleto 845 (MO). Zelaya: near Bil Tingnia, 6 km NW of Bonanza, 150 m, Neill 4000 (M).

**COSTA RICA. Puntarenas**: about 5 km. west of Rincón de Osa, Osa Península, Burger and Gentry 9006 (CR, NY); Buenos Aires, Potrero Grande, Sabanas Helechales, bosques y potreros aledaños al camino, Santamaría et al. 4194 (NY). **San José**: about 1.8 km north of Platanillo on the road to Dominical, Almeda and Nakai 4123 (CR, MO, NY); Basin of El General, Skutch 4842 (CR, NY).

**PANAMA.** (fide Schnell). **Chiriquí**: Burica Peninsula, 4-9 mi S of Puerto Armuelles, Croat 22101 (MO).

**COLOMBIA. Putumayo**: Municipio Mocoa, corregimiento de San Antonio, vereda Alto Campucana, finca La Mariposa, Vertiente Amazónica de Colombia, Betancourt et al. 4957 (NY); Umbría, Klug 1909 (NY).

**ECUADOR. Morona-Santiago**: Centro Shuar Yukutais 8 km SW of Sucua, Andrade 571 (NY); small ravine ca. 7 km N of Limón, Moran et al. 7613 (NY). **Napo**: Puyo, in rastrojo, Asplund 18907 (NY); Mera in forest on shore of Río Pastaza, Asplund 19128 (NY); Hacienda San Antonio del Barón von Humboldt 2 km al NE de Mera, Baker et al. 5370 (MO, NY); Hacienda San Antonia von Humboldt 2 km al NE de Mera, Baker et al. 5501 (NY); Near El Topo along trail to La Gloria, Valley of the río Pastaza and adjacent uplands, Camp 2403 (NY); Loreto, Faldas del Volcán Sumaco, al oeste de Avila Viejo, Bloque 19 línea sísmica 8, Compania Triton, Freire and Cerda 136 (MO, NY); Chaco rastrojal, Harling 3888 (NY); Río Napo between Coca (Puerto Francisco de Orellana) and Armenia Vieja, Harling and Andersson 11978 (NY); Santa Rosa at Rio Napo, Lugo 173 (MO, NY); between Banios and Mera, Mexia 6967 (NY); ca. 50 km NE of Baeza Cascada de San Rafael along Río Quijos, Moran et al. 7561 (NY); Hacienda San Antonio del Baron von Humboldt, 2 km al NE de Mera, Neill et al. 5869 (MO, NY); Hda. San Francisco below Banos, Penland and Summers 279 (NY); Vicinity of Puyo, Eastern foothills of the Andes, Skutch 4518 (NY); carretera Hollin-Loreto-Coca, en las orillas del Río Hollin, Zak and Jaramillo 3140 (NY). **Tungurahua**: Rio Negro, Asplund 18366 (NY); Colonia Mexico 4 km de Topo, Lugo 648 (NY). **Zamora-Chinchipe**: Road La Saquea-Yacuambi 1 km N Chapintza, Harling and Anderson 23885 (NY).

**PERU** (fide Schnell). **Cuzco**: Mapitunari valley, 5-7 km from Hda. Luisiana and the Apurimac river, Cordillera Villacabamba, Madison 10066-7 (NA, US). **Huanaco**: Tingo Maria, Asplund 12992 (US).

**VENEZUELA. Amazonas**: Trail S from Cerro Neblina camp 5, Gentry and Stein 46530 (NY); Cerro Neblina Campamento 5 N base of Pico Cardenas, Gentry and Stein 46650 (NY); Departamento Río Negro, Cerro de La Neblina Camp V Valley north base of Pico Cardona, Liesner and Stannard 16893 (MO, NY).

### Conostegia volcanalis

Taxon classificationPlantaeMyrtalesMelastomataceae

Standl. & Steyermark

Fig. 111

Conostegia volcanalis Standl. & Steyermark, Field Mus. Nat. Hist., Bot. sere 23: 136. 1944. Type: Guatemala. Quetzaltenango: Damp forest, Chiquihuite, 1410 m, 8 March 1939, P. Standley 68152 (holotype: F!, isotypes: A!, NY!).

#### Description.

Trees 2–20 m tall with tetragonal and ridged branches that are generally sparsely to copiously covered with a mixture of caducous, sessile stellate and stalked-stellate hairs; nodal line present. Leaves of a pair equal to somewhat unequal in length. Petioles 0.7–7 cm. Leaf blades 6–32 × 2.6–20 cm, 3–5 plinerved, with the innermost pair of primary veins diverging 1–3.5 cm from the mid vein in opposite to alternate fashion, ovate to elliptic, the base acute or obtuse, the apex acute or obtuse and short acuminate, the margin undulate dentate, the adaxial surface glabrous or glabrescent with sessile or stipitate trichomes which are branching or stellate,the abaxial surface with with sessile or stipitate trichomes which are branching or stellate especially on the veins. Inflorescence a terminal panicle 3.7–16 cm long branching above the base but sometimes appearing branched at the base because of multiple inflorescences arising at opposing meristems at the terminal node, accessory branches present or absent, the rachis glabrescent with few scattered stellate trichomes, bracts and bracteoles to 5 mm long, linear, early deciduous. Pedicel 1.5–15 mm. the hypanthium 2.25–3 × 2.5–3 mm, smooth and mostly glabrous. Flowers 6–10(-12) merous, calyptrate. Floral buds 6–14 × 4–9 mm, spherical, the base rounded or flattened, the apex obtuse to flattened and apiculate, not constricted. Petals 7.5–15 × 4.5–10 mm, white, obovate, spreading, rounded-truncate to emarginate, glabrous. Stamens18–30, 7–8 mm long, radially arranged, to slightly bilateral apparently because of the downward bending style, the filaments 3.75–4.5 mm, white, lacking a geniculation, anthers 2.75–4 × 1–1.25 mm, oblong, straight or recurved, laterally compressed, yellow, the pore 0.1–0.3 mm wide, terminal. Ovary 9–16 locular, inferior, apically glabrous and forming a collar around the style. Style ca. 7 mm long, curving downward, vertical distance from the anthers to the stigma ca. -0.5 – -0.25 mm, horizontal distance ca. 1–2 mm; stigma crateriform, consisting of 9–16 laterally compressed lobes, ca 3–4 mm wide. Berry 10–13 × 8–10 mm, blue-black or purple. Seeds 0.5–0.75 mm, obliquely pyramidal, the testa smooth.

**Figure 111.**
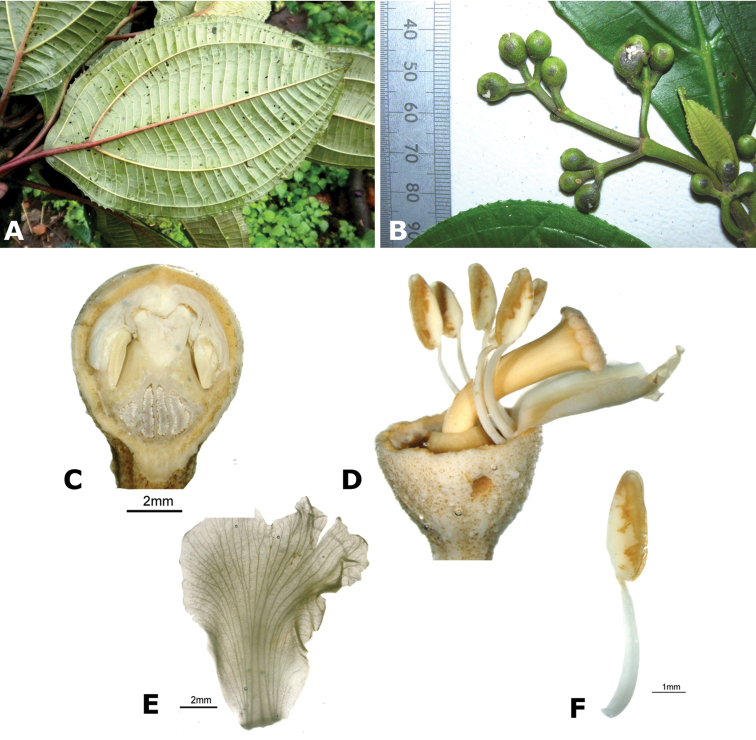
*Conostegia volcanalis*. **A** Leaf abaxial surface **B** Inflorescence with flower buds **C** Longitudinal section of a flower bud **D** Lateral view of a flower at late anthesis **E** Petal **F** Stamen. Photos from specimen vouchered *R. Kriebel 5565*.

#### Distribution

**Figure 112.**
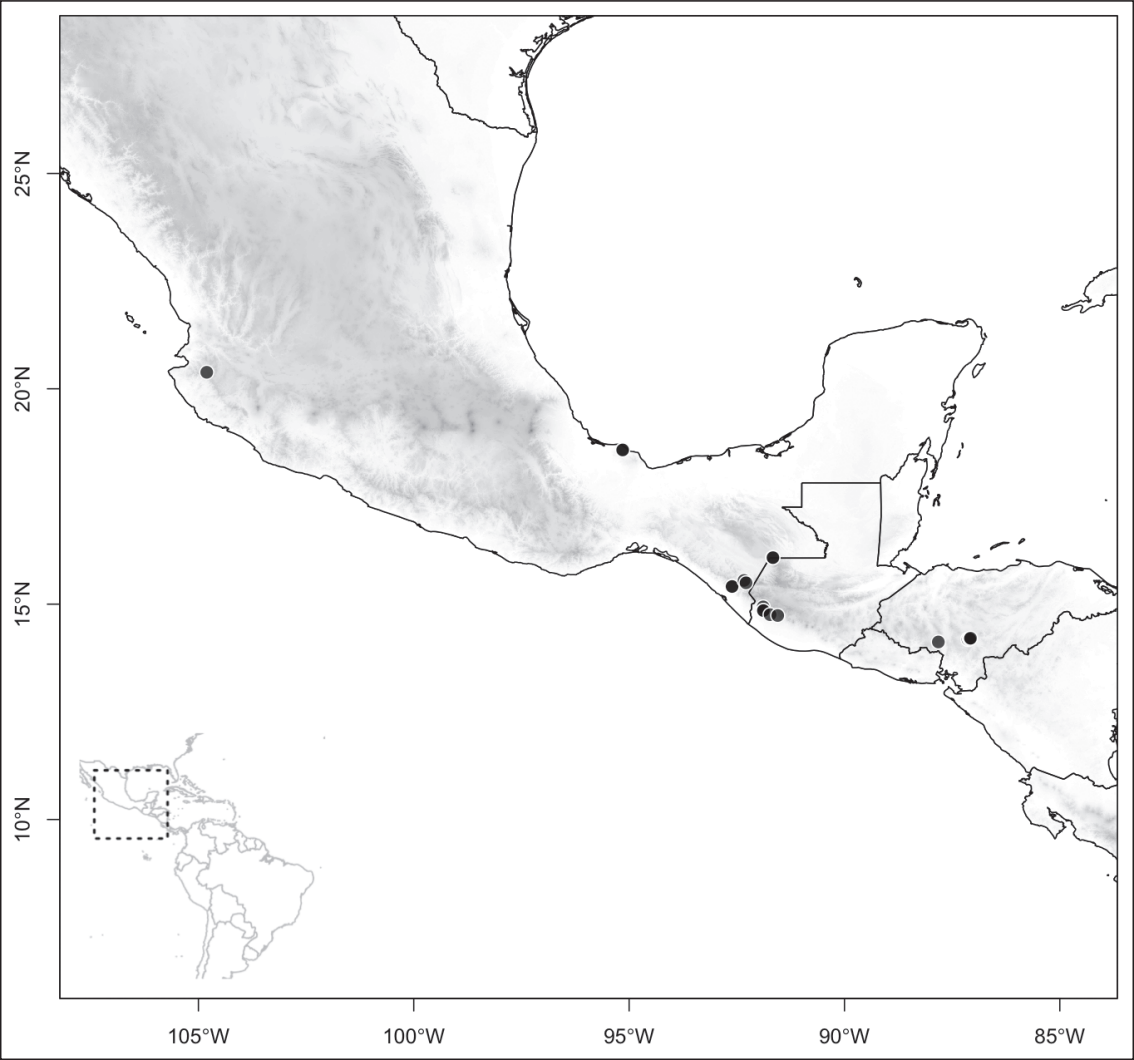
Distribution of *Conostegia volcanalis*.

(Fig. 112). From Mexico through Guatemala to Honduras, 500–2200 m in elevation.

In general, *Conostegia volcanalis* can be recognized on the basis of its mostly spherical flower buds and broad leaves with undulate dentate margins. Schnell (1996) discussed this species as having “three well defined allopatric races”. These morphotypes were considered on the “borderline” of deserving species status (Schnell 1996). The three morphotypes differed in their distribution, habitat preference, indument density, and floral part size. The first morphotype is found in Guerrero and Jalisco and (Schnell 1996) noted that as *Conostegia jaliscana*. These plants prefer streamsides in pine forests. The leaves are is more pubescent and have larger floral parts. These trees flower January through April. The second morphotype recognized by Schnell (1996) occurs in cloud forests of Chiapas, San Marcos and Quetzaltenango in Mexico. The plants from this morphotype are larger trees with less pubescent leaves and flowering November through March. This morphotype is reminiscent of *Conostegia icosandra* and had been described as *Conostegia sphaerica* Triana. The third race occurs in cloud forests of central Guatemala, Honduras, El Salvador and Nicaragua. This morphotype Schnell (1996) characterized as being like morphotype one in pubescence but morphotype two in inflorescence structure. The southernmost population within the third morphotype occurs in Costa Rica and Panama and was described as *Conostegia orbeliana* Almeda. Very few specimens have been collected of this species and they tend to look different than *Conostegia volcanalis* because of their few-flowered inflorescences. When it was only known from Panama, Schnell (1996) noted its flowering time as in January, differing from populations from Guatemala to Nicaragua that flower May through October. Very few specimens are known of this species and Costa Rican specimens remeniscent of *Conostegia orbeliana* have been collected. One of them, was flowering in August, undermining the possible phenological gaps between these populations or possible species. I have chosen not to include *Conostegia orbeliana* under the synonymy of *Conostegia volcanalis* not only because they are allopatric, but also because it might also be a variant of for example *Conostegia oerstediana* or *Conostegia macrantha* which occur in nearby areas. In general, *Conostegia volcanalis* is a complex in need of study to assess habitat and phenological specialization. Perhaps more species can be recognized within this complex in the future.

#### Specimens examined.

**MEXICO. Chiapas**: southwest side of Cerro Mozotal 11 km northwest of the junction of the road to Motozintla along the road to El Porvenir and Siltepec. Municipio de Motozintla de Mendoza, Breedlove and Almeda 58087 (CAS, NY); on the ridge above Siltepec on the road to Huixtla. Municipio de Siltepec, Breedlove 31999 (CAS, NY); La Trinitaria, east of Laguna Tzikaw, Monte Bello National Park, Breedlove 35147 (CAS, NY); Laguna Salina Montceristo, Matuda 2049 (NY); Mt. Ovando, Matuda 2100, 2644 (MO, NY). **Jalisco**: Talpa de Allende, km 12.8 (8 mi) en el camino de La Cuesta hacia Talpa, Cowan and Nieves 4745 (NY); San Sebastián Arroyo de Santa Gertrudis, Mexia 1532 (NY); San Sebastián, Arroyo Monte Oscuro, Mexia 1647 (NY). **Guerrero**: San Antonio-Buenos Aires, Montes de Oca, Hinton 14026 (NY); Chilacayote, Mina, Hinton 14188 (NY). **Veracruz**: Soteapan, Mapa 21.0/58.5, La Azufrera ca. 18 km al E de Lago Catemaco entre Bastonal y Cerro Campanario, Beaman 6108 (NY); San Andrés Tuxtla, Mapa 23.5/55.0, Cerro Vigia al lado E de Volcán San Martín Tuxtla, Beaman and Alvarez 6277 (IE, MO, NY).

**EL SALVADOR** (fide Schnell). **Santa Ana**: Hacienda Montecristo, Metapán, Winkler s.n. (F).

**GUATEMALA. Quetzaltenango**: Above Mujuliá, between San Martin Chile Verde and Colomba, Standley 85672 (NY); slopes and ridges between Quebrada Chicharro and Montania Chicharro on southeast facing slopes of Volcán Santa María, Steyermark 34333 (NY). **San Marcos**: Finca Nueva Granada, Montaña de Vista Hermosa, Kriebel et al. 5565, 5566 (NY, USCG); wet mountain forest near Aldea Fraternidad, between San Rafael Pie de la Cuesta and Palo Gordo west facing slope of the Sierra Madre Mountains, Williams et. al. 25661 (NY).

**HONDURAS. La Paz**: Bosque mixto de Cordillera Guajiquiro 5 kms a Sabanetas, Molina and Molina 12906 (NY). **Morazán**: Mt. San Juancito, Glassman 1986 (NY); Peña Blanca, Montaña La Tigra, Molina 11056, 14495, 25739 (NY); in cloud forest on Mt. Leyuca, Williams 10013 (NY).

### Conostegia vulcanicola

Taxon classificationPlantaeMyrtalesMelastomataceae

Donn. Sm.

Conostegia vulcanicola Donn. Sm., Bot. Gaz. 42: 294. 1906. Type: Costa Rica. Forets de l’Achiote, volcán de Poás, 2200 m, November 1896, Tonduz 10840 (lectotype: NY!, designated here; isolectotypes: BR[2]!, US!). Other syntypes: Costa Rica. Forets de l’Achiote, volcán de Póas, 2100 m elevation, A. Tonduz 10836 (BR, M, US [Schnell (1996) notes that “this number is mixed with Miconia tonduzii Cogn. at BR and US; at CR it includes no Conostegia material]), 10840 (BR [2], NY, US)”.

#### Description.

Shrubs or small trees 3 to 5 m tall with flattened stems that become terete with age and are densely puberulent with sessile stellate trichomes; the nodal line present yet slight. Leaves of a pair equal to somewhat unequal in length. Petioles 1–3.8 cm. Leaf blades 5–15 × 2–7.1 cm, 5-plinerved, with the innermost pair of primary veins diverging from ca. 1 cm from the blade base the midvein in opposite to subopposite fashion, elliptic-ovate to elliptic, the base acute, the apex acute to acuminate,the margin entire or ciliate, the adaxial and abaxial foliar surfaces covered with small stellate hairs. Inflorescence a terminal panicle 3–10 cm, accessory branches apparently absent, the rachis with small stellate hairs, linear, the bracteoles to 3 mm, deciduous. Pedicel 5–20 mm. Flowers 5–6 merous, calyptrate. Floral buds 4–7 × 2–3.5 mm, oblong-pyriform, the base rounded, the apex acute to short apiculate, slightly constricted below the calyptra. Petals 5–7 mm long, white or pinkish, broadly obovate, spreading, glabrous. Stamens 10–12 (-13), 4–5 mm long, possibly slightly bilateral, the filaments ca. 2.5 mm, white, anthers ca. 2 mm long, linear oblong, yellow, the pore ca. 0.2 mm, terminal. Ovary 4–5-locular, inferior, apically glabrous and forming a low collar around the style. Style 3–5 mm, bending below the stigma, apparently no vertical or horizontal distance between the anthers and the stigma, truncate or subcapitate. Berries 5–7 × 5–7 mm, purple-black. Seeds ca. 0.7 mm long, obliquely pyramidal, the testa smooth.

#### Distribution

**Figure 113.**
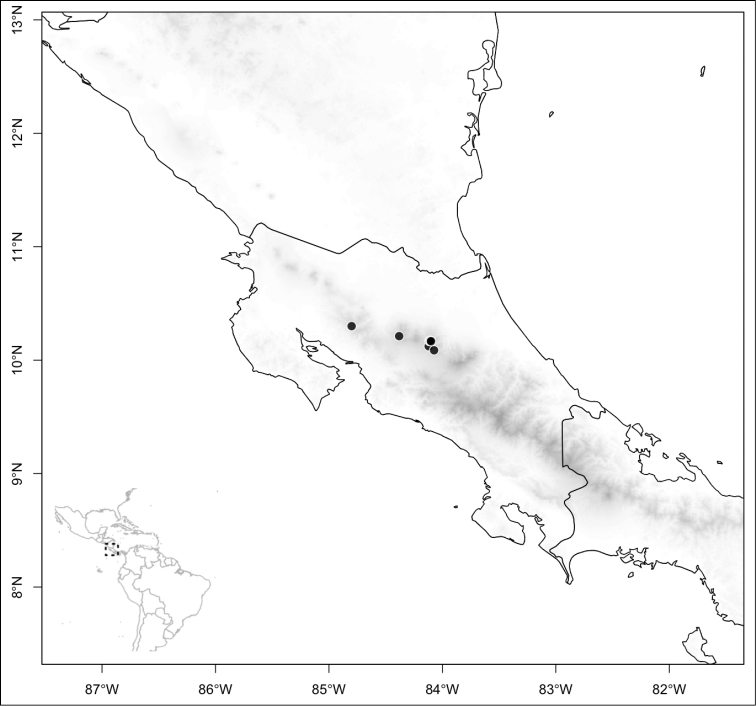
Distribution of *Conostegia vulcanicola*.

(Fig. 113). Costa Rica and reported from Panama by Schnell (1996), 1550–2400 m in elevation.

Considering the lengthy discussions provided by Schnell (1996) in problematic species, it is interesting he did not discuss anything under *Conostegia vulcanicola*, although he did mention that *Conostegia micrantha* and *Conostegia vulcanicola* were probably derived from *Conostegia montana*. I have gone to several volcanoes in Costa Rica looking for *Conostegia vulcanicola* and I found populations that resemble the type specimen except mostly for the reddish hairs on the newest vegetative growth. In general, these specimens also resemble and I have annotated as *Conostegia montana*. The isolectotype of *Conostegia vulcanicola* was previously identified as *Conostegia montana* apparently by A. Tonduz and another specimen identified by H. A. Gleason as *Conostegia montana* was annotated as *Conostegia vulcanicola* by Schnell. The main differences between the two taxa at this time are the indument mentioned above as well as leaf venation. *Conostegia vulcanicola* tends to have plinerved leaves whereas *Conostegia montana* lack the reddish indument and usually has nerved leaves. Further investigation into this species complex is needed.

#### Specimens examined.

**COSTA RICA. Alajuela**: Palmira, Alfaro Ruiz, Smith 1038 (NY).

### Conostegia sect. Australis

Taxon classificationPlantaeMyrtalesMelastomataceae

Kriebel sect. nov.

urn:lsid:ipni.org:names:77156240-1

#### Diagnosis.

A mostly South American group distinguished by the following combination of characters: pleiostemonous flowers, calyx calyptrate, lacking calyx teeth altogether, and with conspicuous sclereids internally exserted styles, stamens lacking a filament geniculation but frequently bearing an evident distinction between the filament and the anther, exserted style, most species lacking a stele inside the style, mucilage inside the ovary present, and seeds ovoid and smooth.

#### Type.

*Conostegia lasiopoda* Benth.

### Conostegia apiculata

Taxon classificationPlantaeMyrtalesMelastomataceae

Wurdack

Fig. 114

Conostegia apiculata Wurdack, Brittonia 9: 103. 1957. Type: Colombia. Nariño: Curcuele, just above San Miguel, 8 km below Piedrancha, valley of Rio Guabo, 1550 m, 1 October 1943, F. Fosberg 21084 (holotype: US!, isotype: NY!).

#### Description.

Small tree to 7 m tall (one label had this information) with tetragonal and ridged young stems that are covered with a rusty indument of sessile stellate trichomes intermixed with dendritic trichomes which have a thick axis and appear stipitate stellate; nodal line absent and/or covered by indument. Leaves equal or unequal at each node. Petiole 0.8–3.5 cm. Leaf blades 8–22 × 2.-8 cm, 3–5-plinerved, with the innermost pair of veins arising just above the blade base or up to 1 cm above the base in opposite to alternate fashion, narrowly elliptic to elliptic, the base acute, the apex acuminate to caudate, entire to inconspicuously denticulate, glabrous adaxially, with small stellate trichomes and branched trichomes mostly on the veins abaxially. Inflorescence a terminal panicle 4–11 cm long, accessory branches absent, branches rusty from the indumenta like the stems, bracteoles linear to lanceolate, 3–10 mm long, persisting but ultimately deciduous. Flowers mostly 6 merous, calyptrate. Flower buds (7.5-) 10–18 × 4–8 mm, slightly constricted in the middle, flattened at the base and apiculate at the apex, the calycine and hypanthial portions weakly differentiated, the hypanthium 4.5–6.25 × 5–6.5 mm, covered with stellate hairs. Petals 8–11 × 6–8.8 mm, white, obovate to obovate-spatulate, emarginate, and glabrous. Stamens 18–24, ca 6–8 mm long, the filament 3–4.5 mm long, white, without an abrupt geniculation but curving below the anthers, anthers 3–3.5 × 0.5–0.7 mm, linear, slightly recurved, the anther connective thickened but without an evident shoulder, yellow, the pore ca. 0.14 mm wide. Ovary mostly 6 locular, glabrous, inferior and elevated into a small collar around the style base. Style ca. 7.5–11 mm, vertical distance from the anther to the stigma ca. 4 mm, horizontal distance absent; stigma capitate, 1.4–1.6 mm wide. Berry not seen.

**Figure 114.**
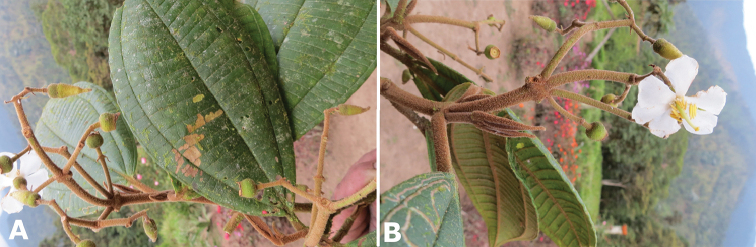
*Conostegia apiculata*. **A** Flowering branch showing and apiculate flower bud **B** Flowering branch showing flower at anthesis. Note exserted style. Photographs taken by Germán Toasa and vouchered *Toasa et al. 11350*.

#### Distribution

**Figure 115.**
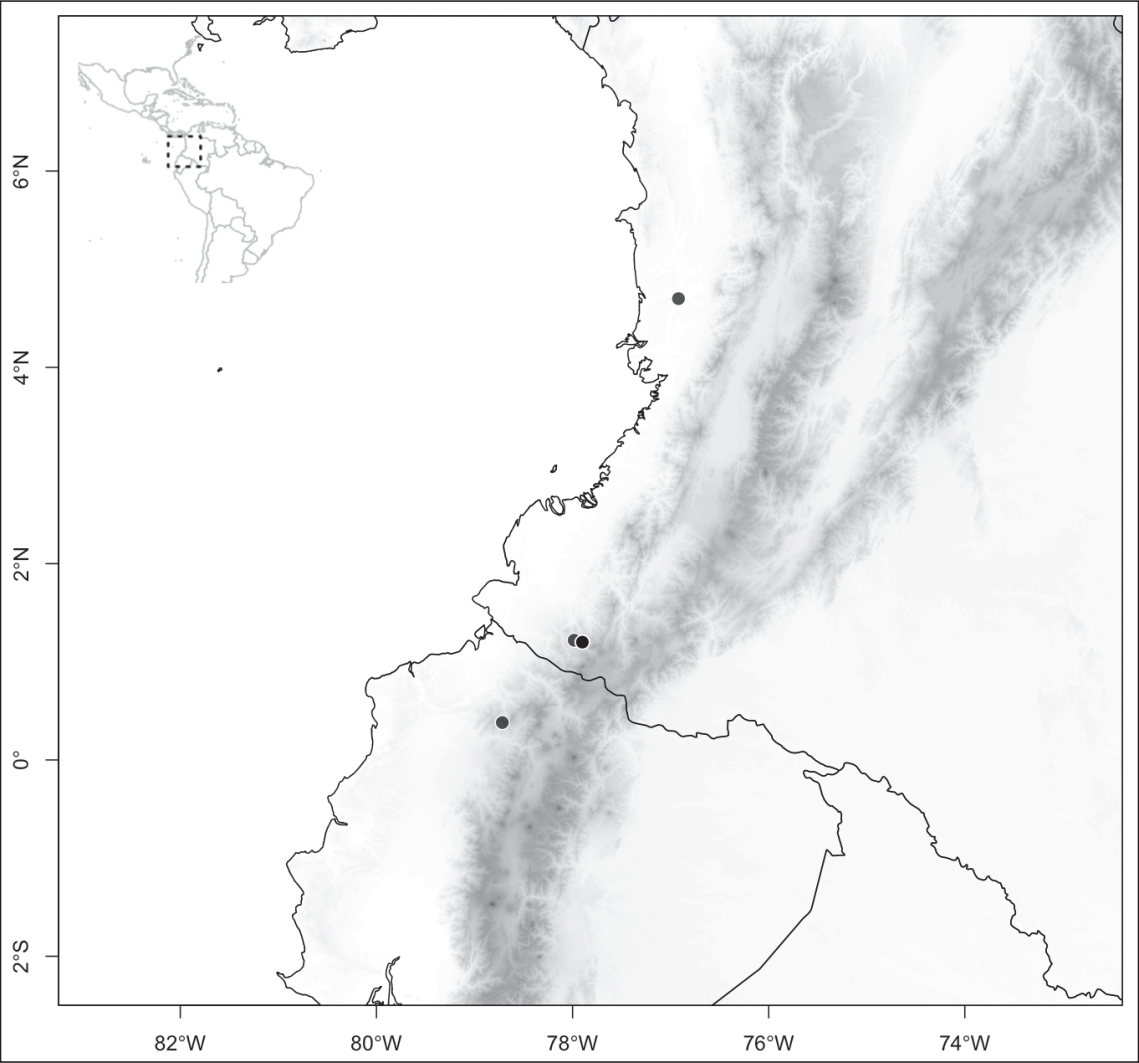
Distribution of *Conostegia apiculata*.

(Fig. 115). Known from few collections in the Pacific coast of Colombia to Central Ecuador, 600–1800 m in elevation.

This rare species can be recognized based on its indument of stellate hairs, long apiculate calyptras, and stout, exserted styles. The drawing made by Wurdack deposited at NY shows the lack of a filament geniculation in this species. Floral measurements made of an Ecuadorian specimen (*Toasa et al. 11350*-NY) show longer styles at least in that population than in Wurdack’s drawing.

#### Specimens examined.

**COLOMBIA. Chocó**: Alrededores de Noanamá, Forero et al. 4568 (COL, MO). **Nariño**: Ricuarte, Reserva Natural La Planada, Agudelo et al. 2963 (COL).

**ECUADOR. Imbabura**: Brillasol, Toasa et al. 11350 (NY).

### Conostegia attenuata

Taxon classificationPlantaeMyrtalesMelastomataceae

Triana

Conostegia attenuata Triana, Trans. Linn. Soc. London 28: 98. 1872. Type: Colombia. Secus flumen Patia prov. Barbacoas Novae Granatae, Triana 3941 (holotype: BM!; isotypes: BR, COL!, GH, K!, P).
Conostegia attenuata var. peruviana MacBride, Field Mus. Publ. Bot. 13: 341. 1941. Type: Ecuador. Esmeraldas: below Playa Rica, forested river bank, Parroquia de Concepción,100 m, 12 December 1936, Y. Mexía 8485 (lectotype: NY! designated here; isolectotypes: BM, F, GR, MO!, NA, S, UC, US!).

#### Description.

Shrub to small tree 0.5–3 m tall with terete glabrescent stems that are beset with an inconspicuous indument of minute sessile stellate or branching trichomes; the nodal line present but inconspicuous. Leaves equal or unequal at each node. Petiole 0.5–2.4 cm. Leaf blades 2.5–15 × 1–3.5 cm, 3-nerved, linear to elliptic, the base attenuate, the apex acuminate, adaxially glabrous and inconspicuously glandular puncticulate, abaxially with small stellate trichomes mostly on the primary and higher order veins, entire to more commonly denticulate. Inflorescence terminal panicles 3.5–7.9 cm long branching above the base, but sometimes appearing branched at the base because of multiple inflorescences arising at opposing meristems at the terminal node, accessory branches present or absent, the rachis glabrescent with few scattered stellate trichomes, bracts and bracteoles 1–5 mm long, linear, persistent. Flowers (5-)6–8 merous, calyptrate. Flower buds 3.9–8.6 × 2.3–4.6 mm, not to slightly constricted in the middle, pyriform to obovate, flattened at the base, acute to short apiculate apically, the calycine and hypanthial portions weakly differentiated, the hypanthium 3–4.5 × 3–5 mm, inconspicuously stellate. Petals 5–9 × 4–7.2 mm, white or pinkish, broadly obovate-spatulate, glabrous, the apex emarginate. Stamens 16–21, 4.1–5.5 mm long, the filament 2.3–3 mm long, anthers 1.8–2.5 × mm, linear or narrowly oblong, straight to slightly recurved, laterally flattened, the base sagittate, with a small bump on the anther connective dorsally at the filament insertion, yellow, the pore ca. 0.1 mm wide. Ovary (4-)5–6 locular, glabrous, inferior and elevated into a conspicuous collar around the style base. Style 4.7–5.7 mm long, vertical distance from the anther to the stigma ca. 1.5 mm, horizontal distance absent; stigma capitate, 0.6–1 mm wide. Berry 4–5 × 4–5 mm, dark purple to black. Seeds ca. 0.4 mm long, pyramidal, the testa smooth.

#### Distribution

**Figure 116.**
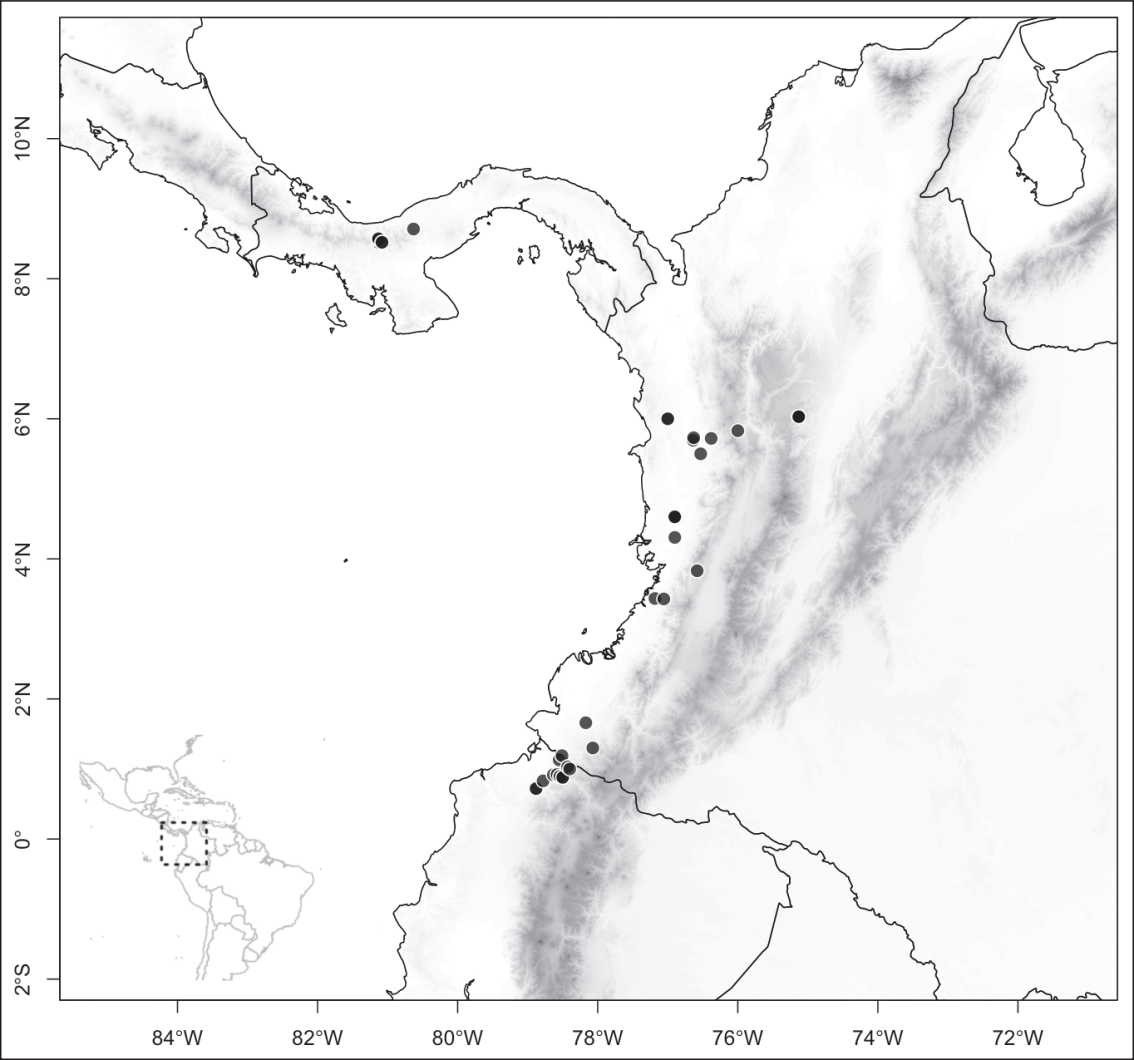
Distribution of *Conostegia attenuata*.

(Fig. 116). Colombia to northern Ecuador, 0–600 m in elevation. Also reported from Panama by Schnell (1996). Conostegia attenuata var. peruviana is known only from a single infertile specimen in Peru and cannot be positively identified at this time. Thus, Peru is excluded in the present distribution of the species.

Floral measurements of Schnell are quite small in comparison to those of Gleason. The latter author measured three specimens of which the drawings are deposited at NY. With regards to staminal morphology, Gleason also noted in his drawings that “thecae are prolonged below the insertion of the filament. Connective minutely gibbous on the back just above apex the filament.” This morphological feature is common and only present in section *Australis*. Schnell (1996) and Almeda (2009) reported *Conostegia attenuata* from Costa Rica. The Costa Rican specimens cited by these authors (*Skutch 2734*, *4941*-NY) differ quite a bit from typical *Conostegia attenuata* material. The main differences are that the Costa Rican material is glabrous to inconspicuously lepidote, has plinerved leaves, and most notably has short styles. The typical *Conostegia attenuata* has stellate trichomes on the branch apices and floral buds, nerved leaves and exserted styles. Both specimens were also annotated as a probable new species on the NY specimens by Schnell. Given that they are highly reminiscent to *Conostegia montana* and have not been recollected, I consider them a narrow leaf variant of *Conostegia montana* for the time being.

#### Specimens examined.

**COLOMBIA. Chocó**: Carretera Medellín-Quibdó, Adelante de Ciudad Bolívar, Km. 171 río La Playa, Forero, Jaramillo and McElroy 1082 (NY); Quibdó, Quebrada La Platina, I Cuatrecasas 2700 (F, MO, NY). **El Valle**: Costa del Pacífico, Río Yurumanguí, entre Isla de Golondro y La Amargura, Cuatrecasas 16063 (NY); Río Calima (región del Chocó), Quebrada de La Brea, Cuatrecasas 21265 (NY); Costa del Pacífico, río Cajambre, Barco, Cuatrecasas 17170 (NY); Buenaventura, Quebrada San Joachim, Schultes and Villareal 7310 (NY). **Nariño**: Quebrada La Toma, Rio Telembi betw. Rio Pimbi and Rio Cuembí, Ewan 16858 (NY).

**ECUADOR.** Carchi Border area between Prov. Carchi and Esmeraldas about 20 km past Lita on road Lita-Alto Tambo, van der Werff et al. 12000, 12018 (NY). **Esmeraldas**: Río San Juan and Río Camumbi, primary tall forest at Tobar Donoso junction of Río San Juan, Játiva and Epling 1119 (NY); Parroquia de Concepcion below Playa Rica, Mexia 8485 (NY); San Lorenzo, Reserva Indígena Awá, Cañon del Río Mira 10 km al oeste de Alto Tambo, Comunidad “La Unión”, Rubio et al. 1173 (MO, NY).

### Conostegia centronioides

Taxon classificationPlantaeMyrtalesMelastomataceae

Markgraf

Fig. 117

Conostegia centronioides Markgraf, Notizbl. Bot. Gart. Berlin-Dahlem 14: 25–44. 1938. Type: Ecuador. San Carlos de los Colorados, West-Ecuador, 120 m, 7 September 1935, H. Schultze-Rhonhof 1898 (B, destroyed). Neotype (designated here): 20 km W. of Santo Domingo de los Colorados, 300 m, Pichincha, Ecuador, Cazalet and Pennington 5017 (holoneotype: NY!, isoneotypes: K, US!).

#### Description.

Shrubs to small trees 2–11 m tall with flattened or tetragonal that are finely and densely pubescent with tiny sessile stellate trichomes; the nodal line present yet slight. Leaves equal to unequal at each node. Petiole 1–5 cm long. Leaves 5–21 × 1.9–10 cm, 3–5 nerved or if 3–5 plinerved, with the innermost pair of primary veins diverging from the mid vein up to about to about 2 cm above the base, elliptic, the base acute to obtuse, the apex abruptly acuminate, usually conspicuously undulate denticulate, adaxially glabrous except for inconspicuous stellate trichomes when young, abaxially stellate pubescent with small trichomes on the veins to almost glabrous. Inflorescence terminal a panicle 3.7–22 cm long branching above the base but sometimes appearing branched at the base because of multiple inflorescences arising at opposing meristems at the terminal node, accessory branches present, rachis pubescent with small stellate trichomes; bracts and bracteoles 0.5–2 mm, linear to lanceolate, usually deciduous. Flowers 5–6(-7) merous, calyptrate, the calyptra apparently consistently breaking in pieces. Flower buds 4.7–11 × 2.5–6.5, pyriform, the base flat to rounded, the apex rounded to short apiculate, the calycine and hypanthium portions weekly differentiated, constricted below the calyptra; the hypanthium 3–6 × 3–6 mm, pubescent with rusty stellate hairs. Petals 6–9 × 6–9 mm, white to translucent white, obtriangular, glabrous, emarginate to three lobed apically. Stamens 15–24, 4.8–7 mm long, slightly zygomorphic, the filament 3–4 mm, white, not geniculate, anthers 1.8–3, linear-oblong, pale yellow, laterally compressed, dorsally thickened, basally sagittate, the pore ca. 0.1 mm wide, terminal. Ovary 5–6(-7), inferior, glabrous, the apex forming a conspicuous collar around the style base; style ca. 6–8 mm, straight to gently curving near the apex, vertical distance from the anther to the stigma 1.5–3 mm, horizontal distance absent, the stigma capitate, ca. 1 mm wide. Berry 6–7 × 6–7 mm, purple. Seeds 0.4–0.5 mm long, ovoid and smooth.

**Figure 117.**
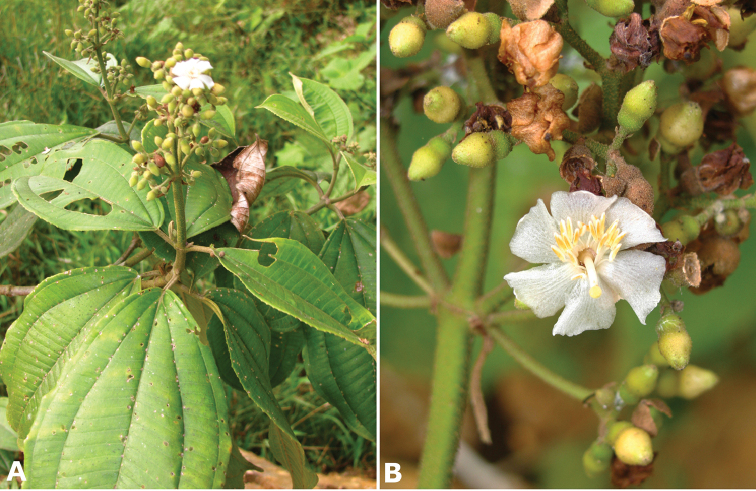
*Conostegia centronioides*. **A** Fertile branch **B** Close up of the flower. Photographs taken by Xavier Cornejo and vouchered *X. Cornejo 8160*.

#### Distribution

**Figure 118.**
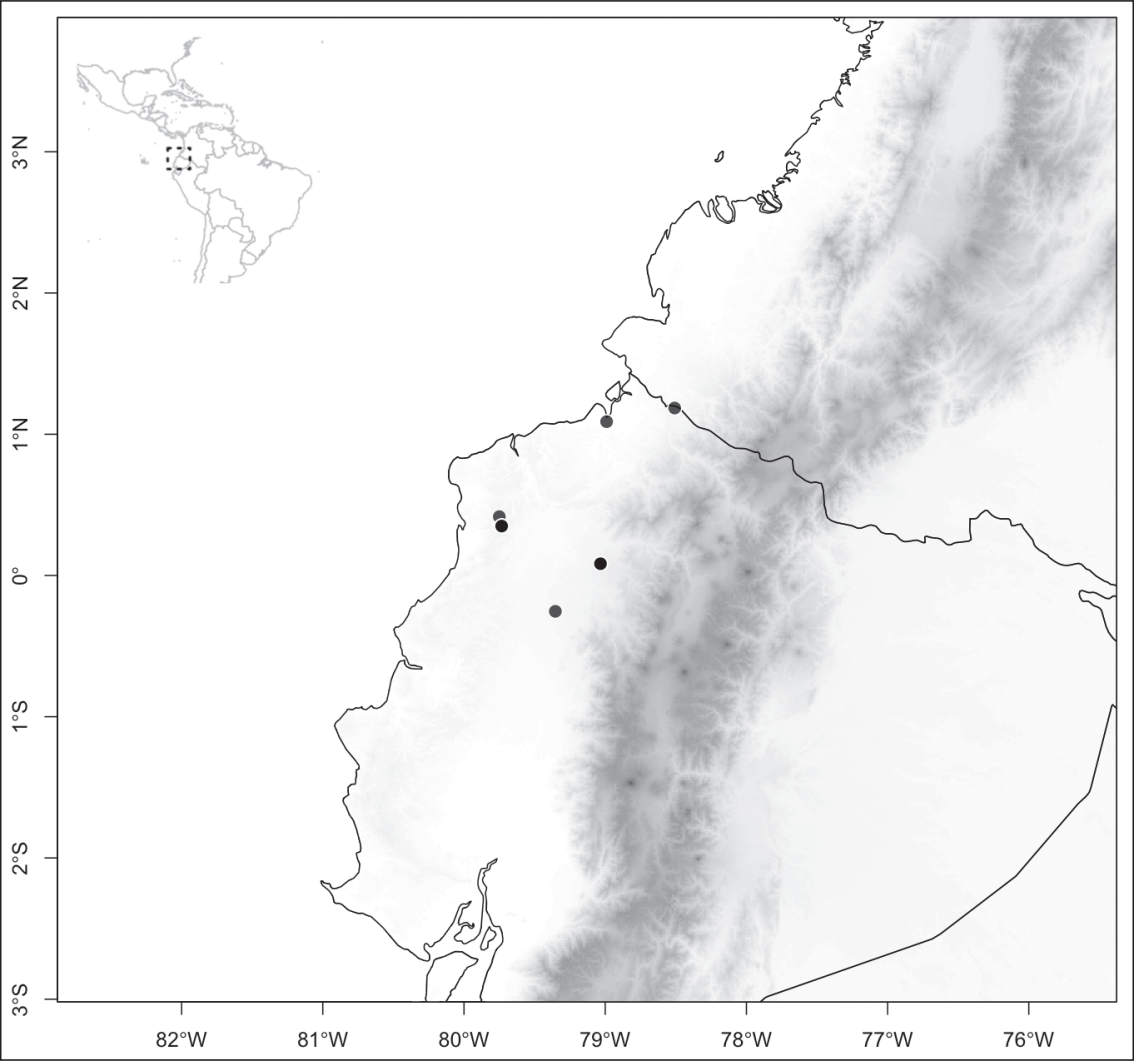
Distribution of *Conostegia centronioides*.

(Fig. 118). Mostly in northwestern Ecuador and probably overlooked in south-western Colombia, 20–900 m in elevation.

Schnell (1996) reported that the holotype of this species (as well as the isotypes which had not been distributed) were lost at Berlin. As mentioned by Schnell (1996), the neotype chosen here (the same chosen by Schnell’s unpublished work) fits the original description. *Conostegia centronioiodes* is quite variable in the sizes of leaves, inflorescences and flower buds as well as in the flower bud apex which can be more or less rounded to short apiculate. Perhaps because of its small flower buds, this species has been confused with *Conostegia montana*. An easy way to tell these species apart in bloom is that *Conostegia montana* has a short style whereas *Conostegia centronioides* has a long style. The same character can be used to separate *Conostegia centronioides* from *Conostegia rufescens*, another species with which it gets confused. Lastly, *Conostegia centronioides* is similar to *Conostegia lasiopoda*, particularly when in the latter the large bracts that cover the flower buds have fallen off. *Conostegia lasiopoda* tends to have conspicuously setose adaxial petiole surfaces which aid in its distinction from *Conostegia centronioides*.

#### Specimens examined.

**ECUADOR. Carchi**: above San Marcos de los Coaiqueres on trail towards Gualpí Bajo, Ollgaard et al. 57324 (MO, NY); Tulcan Cantón, Parroquia Tobar Donoso, Sector Sabalera, Reserva Indígena Awá, Bosque primario Noreste Casa Comunal, Tipaz, Zuleta and Guanga 1333 (NY). **Esmeraldas**: 2-4 km SE of San Lorenzo along railroad track, Boom 2529 (MO, NY); Quininde Cantón, Bilsa Biological Station, Montanias de Mache 35 km W of Quinindé, 5 km W of Santa Isabel, Clark and Watt 755 (NY); Quininde, Bilsa Biological Station, Reserva Ecológica Mache-Chindul, 35 km W of Quinindé, Clark and Pallis 4871 (NY); near San Jose Mission, Cayapa River, Játiva and Epling 1038 (NY); near Playa Grande, Cayapa River, Játiva and Epling 2090 (NY); Across Rio Quininde w about 2 km, Little 6215 (NY); Borbón, Little 21045 (NY); Parroquia de Concepción, Playa Rica, Mexia 8435 (NY); Eloy Alfaro, San Miguel, Río Cayapas, Propiedad del Sr. Miguel Chapiro, Parcela Permanente 07 y alrededores, Palacios and Tirado 11101 (NY). **Pichincha**: Carretera Quito-Puerto Quito Km 113, 10 Km al Norte de la carretera principal, Betancourt 129, 154, 173, 223 (NY); 20 km W of Santo Domingo de los Colorados, Cazalet and Pennington 5017 (NY).

### Conostegia dentata

Taxon classificationPlantaeMyrtalesMelastomataceae

Triana

Conostegia dentata Triana, Trans. Linn. Soc. London 28: 99. 1872. Type: Colombia. Chocó: J. Triana 4113 (not seen-see discussion for details on the type).
Conostegia hispida Gleason, Bull. Torrey Bot. Club 66: 415. 1939. Type: Ecuador. Esmeraldas: Playa Rica, Parroquia de Concepción, 105 m, 10 December 1936, Y. Mexía 8430 (holotype: NY!, isotypes: BM, F, GH, MO!, NA, S, UC, US!).

#### Description.

Shrub to small tree 1.3–8 m with sub-terete stems that are densely setose with simple bristles up to 4 mm long; the nodal line hard to see and covered with setae as the rest of the node and internode. Leaves at a node equal to sub equal in size. Petiole 0.5–4.9 cm long. Leaves 11.9–35 × 5–12.9 cm, 3–5 plinerved, with the innermost pair of veins arising up to about 3 cm above the base and diverging mostly in opposite or sub opposite fashion from the mid vein, obovate to nearly elliptical, the base acute to decurrent on the petiole and with paired formicaria ca. 2–4 cm long on the leaf surface or extending to the petiole, the apex rounded to obtuse and abruptly acuminate, the margin dentate, undulate-dentate or denticulate, adaxially sparsely setose, abaxially setose. Inflorescence a terminal compact panicle 1.5–4(-7) cm long, inflorescence rachis obscured by the dense setose indument; bracts and bracteoles 1.5–4 mm. Pedicel ca. 2 mm long, obscured by the indument. Flowers (5-)6(-8) merous, calyptrate. Flower buds ca. 9–15 × 5–7 mm, narrowly ovate not constricted about the middle, rounded a the base, acute and long attenuate at the apex, not constricted in the middle, the calycine and hypanthial portions undifferentiated; the hypanthium 5–6.25 × 5.5–7.5 mm, densely hirsute with trichomes with swollen bases. Petals ca. 9–13 × 9–12 mm, white or pink, obtriangular, spreading, glabrous, the apex emarginate. Stamens 19–30, ca. 7–8 mm long, their posture at anthesis not seen, the filament 4–5 mm long, lacking a conspicuous geniculation, anther 2.8–3.5 mm, linear-oblong, recurved near the base, yellow, laterally compressed, the connective thickened and with a small bump dorsally, the pore ca. 0.1 mm, subterminal and slightly ventrally inclined. Ovary 6–10 locular, inferior, the apex glabrous and forming a collar around the style base. Style 6.5–7.5 mm long, gently curved, vertical distance from the anthers to the stigma ca. 1–2 mm, horizontal distance absent, the stigma sub capitate, ca. 1.3 mm wide. Berry ca. 7 × 7 mm when dry. Seeds not seen.

#### Distribution

**Figure 119.**
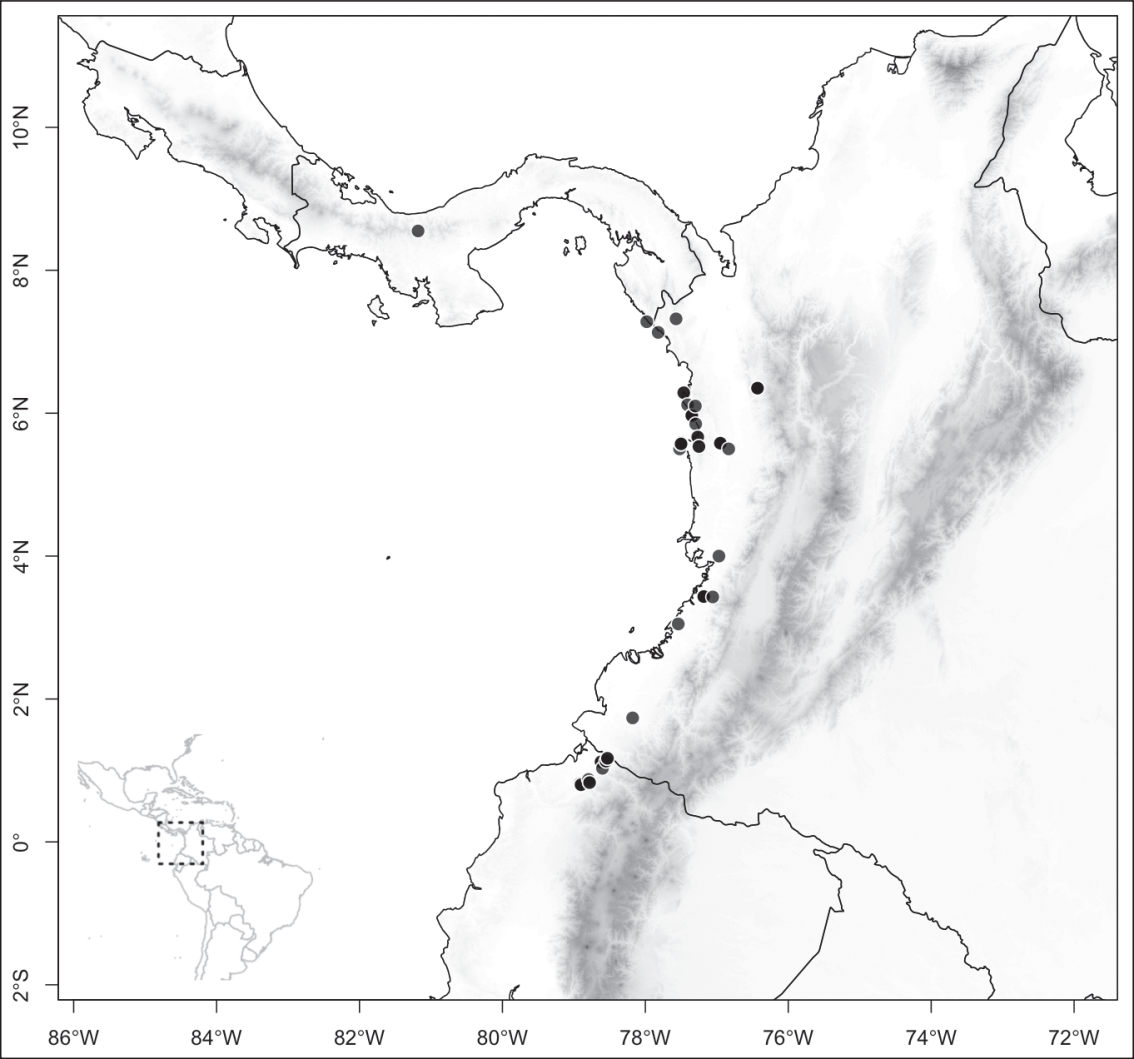
Distribution of *Conostegia dentata*.

(Fig. 119). Ranging from Panama to Colombia and Ecuador on the Pacific coast, 0–680 m in elevation.

*Conostegia dentata* is easily recognized because of its setose indument on stems, inflorescences and flower buds, leaves with formicaria at the base and dentate margins, and compact inflorescences. The study of flowers in herbarium specimens revealed an exserted style as is typical of species of section *Australis*. Almeda (2009) was unable to locate the type specimen *Triana 4113* of this species at BM and when checking Triana’s herbarium list noted that that number is stated to be from Antioquia instead of Chocó, but no genus or species are stated. Almeda did locate *Triana 4112* which corresponds to *Conostegia dentata* but that specimen is from Barbacoas province instead of Chocó province where *Triana 4113* was cited to have been collected. I agree with Schnell (1996) that the original description leaves little doubt on its identity namely because of the description of the setose indument, congested inflorescences and long petals. Because the setae of the fruiting hypanthia are white instead of brown as in the rest if the plant, Schnell (1996) hypothesized that this color difference might function to attract dispersal agents.

#### Specimens examined.

**PANAMA** (fide Schnell). **Darién**: Cocalita near the Colombian border on the Pacific side, Dwyer 4395 (BR, GH, UD); Atlantic slope NW of Santa Fe, 11 km from Escuela Agricola Alto de Piedra, in valley of Rio Dos Bocas, Mori and Kallunki 3850 (US).

**COLOMBIA. Cauca**: Costa del Pacífico, río Micay, orilla derecha, en Caliche, Cuatrecasas 14190 (NY). **Chocó**: Mun. Nuqui, Corregimiento Termales, Quebrada Piedra Piedra, Acevedo-Rodríguez, Callejas and Churchill 6800 (NY); Río Mutatá tributary of Río El Valle between base of Alto de Buey and mouth of river, Gentry and Fallen 17474 (MO, NY). **El Valle**: Costa del Pacífico, río Cajambre, Barco, Cuatrecasas 17143 (NY, US); Costa del Pacífico, río Cajambre, San Isidro, Cuatrecasas 17280 (NY); Costa del Pacífico, río Cajambre, Silva, Cuatrecasas 17668 (NY); Colorado, north shore of Buenaventura Bay, Killip 38772 (NY). **Nariño**: Quebrada Mongon at Camp Mongon, Río Telembi above Barbacoas, Ewan 16877 (NY).

**ECUADOR. Esmeraldas**: Eloy Alfaro, Reserva Ecológica Cotacachi-Cayapas, Río Santiago, Tirado 571 (MO, NY).

### Conostegia extinctoria

Taxon classificationPlantaeMyrtalesMelastomataceae

(Bonpl.) D. Don ex DC.

Fig. 120

Conostegia extinctoria (Bonpl.) D. Don ex DC., Prodr. 3: 174. 1828.
Melastoma extinctoria Bonpl., Melast. 133, t. 57. 1806–1816. Type: Colombia. Mariquita, royaume de Santa Fe, A. Bonpland 1719 (fide Almeda in Schnell, 1996, holotype P; isotype P!).
Conostegia pulverulenta Naudin, Ann. Sci. Nat. ser. 3, 16: 110. 1850. Type: In America meridionali?; loco nec collectore cognitis., collector unknown (holotype: P!).

#### Description.

Shrub to small tree 2.5–7(-12.5) m tall with flattened stems that are furfuraceous on new growth with stellate trichomes; the nodal line present yet slight. Leaves at a node equal to sub equal in size. Petiole 0.7–2.8 cm long. Leaves 6–24.8 × 3–10.1 cm, 5 plinerved, with the innermost pair of veins diverging up to 2.5 cm above the base mostly in sub opposite fashion, elliptic to narrowly ovate, the base subacute to obtuse, acute or acuminate at the apex, the margins remotely undulate to denticulate, adaxially glabrous or glabrescent, abaxially with numerous underdeveloped stellate trichomes. Inflorescence a terminal panicle 6–12.3 cm long branched above the base, accessory branches present, the rachis covered with stout stellate or dendritic trichomes. Pedicel 2–4.4 mm. Flowers (4-)5–6(-7) merous, calyptrate. Floral buds 3–5.5 × 3.75–4.25, oblong-pyriform, acute at the base, acute and apiculate at the apex; hypanthium ca. 3–4 × 3.25–4.25 mm, not constricted in the middle, stellate pubescent, the calyptra and hypanthium not differentiated. Petals 4–5 × 3–3.5 mm, white, broadly obovate, spreading, glabrous, rounded with a lobe to one side of the flower. Stamens (16)27–36, 4.75–6 mm long, apparently slightly zygomorphic, the filament 2.5–3 mm, white, anthers 2.25–2.75 × 0.25–0.75 mm, linear, white to yellowish, not evidently sagittate at the base, the pore 0.1–0.12 mm, ventro-terminal. Ovary (5-)6–7(-8) locular, inferior, glabrous. Style 7–8 mm, straight, lacking a prominent a collar at the base, vertical distance from the anthers to the stigma ca. 1–1.5 mm, horizontal distance absent; stigma subcapitate, 0.75–1 mm wide. Berry 4–5 × 4–5 mm, purple-black. Seeds 0.3–0.4 mm, ovoid, the testa smooth to a little roughened.

**Figure 120.**
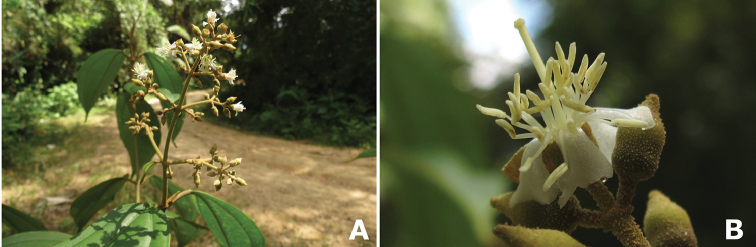
*Conostegia extinctoria*. **A** Fertile branch **B** Close up of the flower. Photographs by M. A. Buitrago Aristizabal of specimen vouchered *J. M. Posada 308*.

#### Distribution

**Figure 121.**
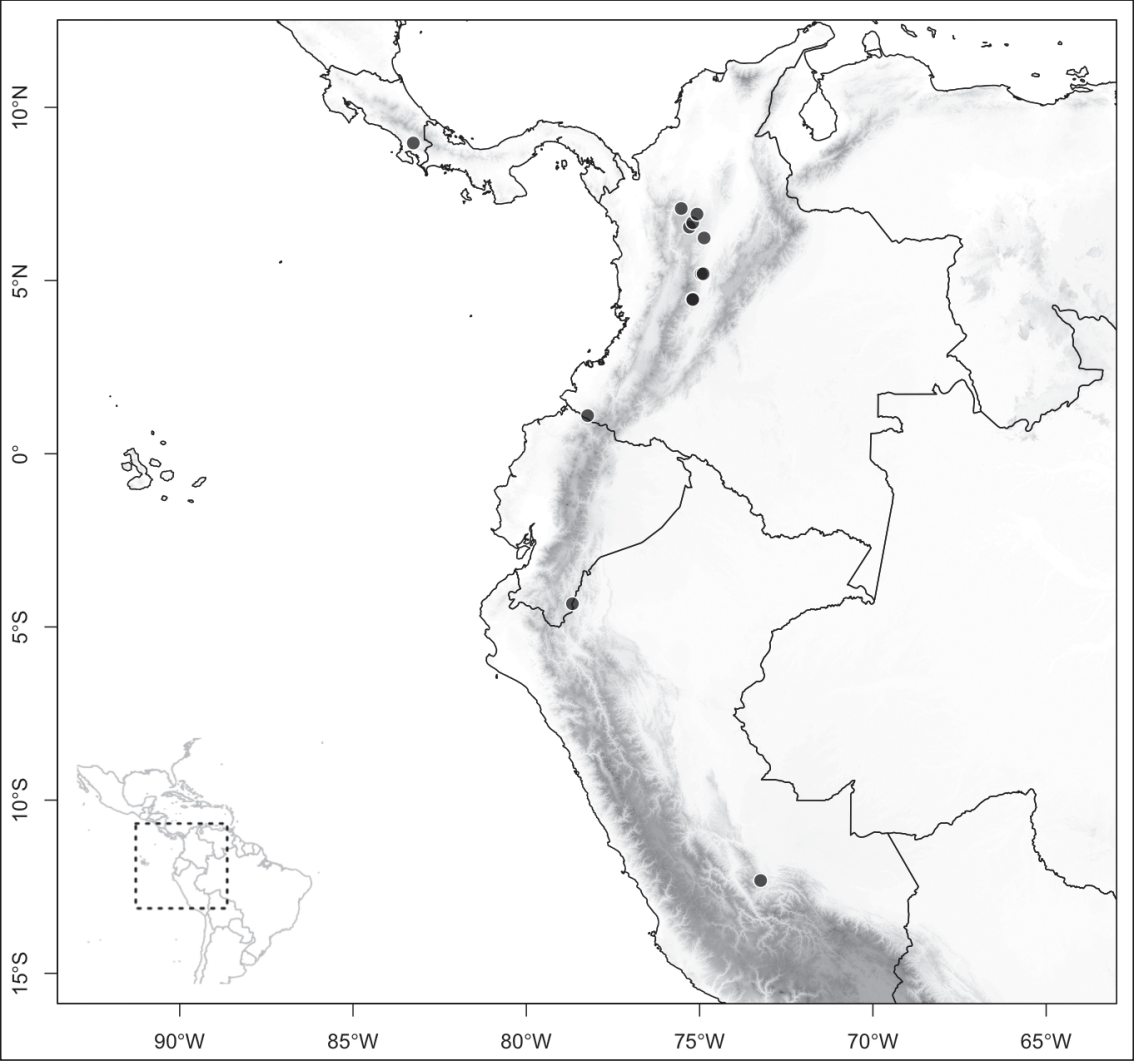
Distribution of *Conostegia extinctoria*.

(Fig. 121). From southern Costa Rica to Colombia, with a disjunct population in the Cordillera de Vilcabamba in Peru, 700–1350 m in elevation. The inclusion of the Peruvian locality is based on a specimen cited by Schnell (1996).

*Conostegia extinctoria* is one of the smallest flowered species in section *Australis*. The style of this species is exserted as in other species of section *Australis* (Fig. 120). Schnell (1996) commented on the variation within this species, in particular in the number of floral parts. As in other species in this section, some specimens (e.i. *Callejas et al. 8926*-NY) suggest the calyptra is not cleanly circumscissle and instead ruptures at anthesis.

#### Specimens examined.

**COSTA RICA. Puntarenas**: forets du Boruca, Tonduz 4965 (US).

**COLOMBIA. Antioquia**: Municipio Anori, Proyecto hidroeléctrico Porce III, Vargas et al. 1581 (COL); Municipio de San Carlos, Corregimiento El Jordán, Trocha “Los Planes”, entre carretera Alto de Samaná-Quebrada La Villa, Embalse Punchiná, Velásquez 266 (NY); Municipio Gomez Plata, 10–15 km en la vía Barbosa-Porce-Amalfi N de Barbosa en límites con el Municipio de Yolombó a orillas del Río Medellín, Callejas et al. 8926 (MO, NY); Municipio de Amalfi, Finca la Picardía, sector el Coco, David, Rivas and Hernández 1227 (NY). **Tolima**: Mariquita, en La Parroquia, Uribe 2766 (NY); Ibague, cerca de la Escuela de Agronomía, Uribe 4957 (COL).

**PERU** (fide Schnell). **Cuzco**: La Convención, east side of Rio Apurimac, across from Hacienda Louisiana, Cordillera Vilcabamba, Madison 10001–70 (NA).

### Conostegia lancifolia

Taxon classificationPlantaeMyrtalesMelastomataceae

(Markgraf) C.E. Schnell ex Kriebel comb. et stat. nov.

urn:lsid:ipni.org:names:77156241-1

Conostegia lancifolia (Markgraf) C.E. Schnell ex Kriebel. Basionym: Conostegia centronioides var. lancifolia Markgraf, Notizbl. Bot. Gart. Berlin-Dahlem 15: 377. 1941. Type: Ecuador. Mera: 1000 m, 8 November 1938, H. Schultze-Rhonhof 2966 (B, destroyed). Neotype designated here: Ecuador. Pastaza: between Mera and Moravia, 1000 m, 16 December 1955, E. Asplund 18868 (holoneotype: NY!).

#### Description.

Shrubs or small trees 1–5 m tall with tetragonal to rounded stems which are covered predominantly by stipitate stellate trichomes; nodal line present, somtimes obscured by indument. Leaves at a node equal to unequal in size. Petiole 0.4–1.8 cm. Leaf blades 5–17 × 2–6 cm, 3–5 slightly plinerved, elliptic to lanceolate, rounded to subacute at the base, the apex and acuminate, margins entire to undulate-denticulate, glabrous adaxially, pubescent mainly on the primary veins abaxially with indument like that of the stems. Inflorescence a terminal panicle 4–9 cm long branched above the base but sometimes appearing branched at the base because of multiple inflorescences arising at opposing meristems at the terminal node, accessory branches absent or present. Bracts and bracteoles 1.5–3 mm long, linear. Pedicels 1–3 mm long. Flowers 7–9 merous, calyptrate. Floral buds 6–9.5 × 3–6.5 mm, ovoid to obovoid, slightly constricted below the calyptra, the hypanthium and calyptra undifferentiated; hypanthium 3–3.5 × 3.75–4.25 mm, pubescent with sessile and/or stipitate stellate trichomes. Petals 8–10 × 5–6 mm, white, obtriangular, posture not seen, glabrous on both surfaces, emarginate apically. Stamens 30–42, 7–8 mm long, the filament 4–4.5 mm, apparently white, anthers 3–3.5 × 0.5–0.75 mm, linear subulate, pale yellow (in rehydrated specimen), the pore ca. 0.18 mm, terminal to slightly ventrally inclined. Ovary 11–12-locular, inferior, glabrous and forming a collar around the style base; style ca. 7.5–8 mm, gently bending from the base, glabrous, the style appears to be exserted but difficult to assess in the rehydrated material as well as if it is bent opposite the stamens or not, stigma capitate, ca. 2 mm wide. Mature berry not seen.

#### Distribution

**Figure 122.**
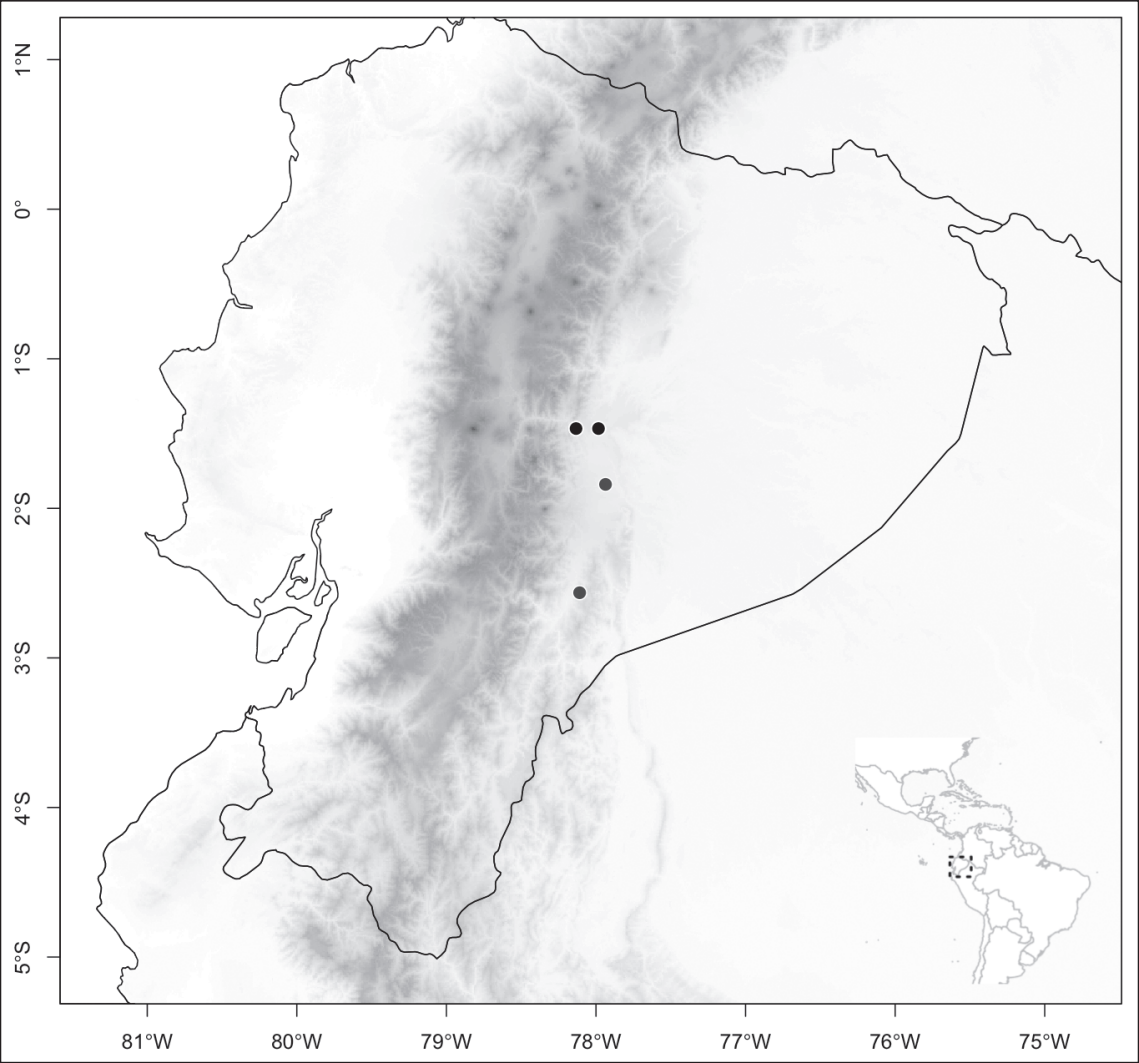
Distribution of *Conostegia lancifolia*.

(Fig. 122). Southeastern foothills of the Andes in Ecuador at 800–1200 m in elevation.

Schnell (1996) noted that as like for *Conostegia centronoides*, the holotype of Conostegia centronoides var. lancifolia was lost during the war in Berlin. I concur with Schnell (1996) in that the description of the latter including stipitate stellate hairs and narrowly ovate leaves matches topotypical material which Schnell cited as the neotype (*E. Asplund 18868*). For this reason and because the work of Schnell (1996) wasa not published, the neotypification is made here. Schnell (1996) included in his circumscription of *Conostegia lancifolia* the decurrent leaved and large leaf blade plants here referred to as *Conostegia ortizae*. Schnell pointed out that if intermediates between the two morphotypes did not arise they might as well be called subspecies or species. Considering they are easy to distinguish they are here proposed as different species.

#### Specimens examined.

**ECUADOR. Morona-Santiago**: 2-4 km N of Arapicos, Lugo 5957 (NY); along Río Palora 2-5 km downstream from Arapicos, Lugo 5990, 6047 (NY). **Napo-Pastaza**: Mera, Asplund 19348 (NY); between Mera and Moravia, Asplund 18868 (NY); Mera, Harling 3697, 3701 (NY); Mera, Isidro Ayora, Harling 19723 (NY); Río Tigre, in the vicinity of Mera, Lugo 853 (NY); Puyo, Prescott 384 (NY).

### Conostegia lasiopoda

Taxon classificationPlantaeMyrtalesMelastomataceae

Benth.

Fig. 123

Conostegia lasiopoda Benth., Bot. Voy. Sulphur 96. 1844. Type: Costa Rica. Puntarenas: Cocos Island, no date, *Barclay s.n.* (K!).
Conostegia trianaei Cogn., DC. Monog. Phan. 7: 702. 1891. Type: Colombia. Chocó: 1851–1857, J. Triana 3943 (holotype: BR!, isotypes: BM!, NY!, K!, US!, W).
Conostegia sororia Standl., Field Mus. Nat. Hist., Bot. sere 22: 161. 1940. Type: Panama. Darién: Cana-Cuasi trail, Rio Cuasi (camp I), Chepigna, 250 m, 7 March 1940, M. and R. Terry 1414 (holotype: F!, isotypes: A!, MO!).

#### Description.

Shrubs to small trees 1.5–6 m tall with stems that are first tetragonal then terete and setose with appressed single bristles 1–2 mm long and many minute sessile stellate hairs; nodal line present yet slight. Leaves at a node equal to subequal in size. Petioles 0.7–3.6 cm long, densely hirsute adaxially. Leaves 8.3–30 × 3.6–11.7 cm, 3–5 nerved, ovate to obovate, cuneate to obtuse at the base, the apex acute to rounded and attenuate to abruptly acuminate,the margins entire to undulate-dentate, the adaxial surface glabrous, slightly concave and with deeply impressed tertiary venation, abaxially covered only with a layer of these tiny sessile stellae. Inflorescence terminal, 5–19.1 cm long, accessory branches absent or present, the branches subtended by deciduous, setose linear bracteoles 2–20 mm long, the clusters of flowers subtended by puberulent foliose, persistent to deciduous bracteoles 2–7 mm long. Pedicels of 0.5–2.0 mm. Flowers (5-)6(-8) merous, calyptrate. Floral buds 4–9 × 2–5 mm, ovate to oblong-pyriform, the base rounded, the apex acuminate, constricted below the middle, the upper and lower portions undifferentiated, the hypanthium 3.5–4 × 3.25–3.5 mm, pubescent. Petals 7–8 × 7–8 mm, translucent white, obtriangular, spreading, glabrous, and slightly asymmetrical apically. Stamens 17–25, 4–6 mm, slightly zygomorphic apparently from the movement of the style, the filament 2.5–3 mm, white, anthers 1.5–2 × 0.5–1 mm, linear-oblong, straight or recurved, light yellow., the pore terminal, less than 0.1 mm. Ovary 6–9 locular, inferior, apically glabrous and forming a low collar around the style. Style 5–8 mm, straight for most of its length and bending gently near the apex, vertical distance from the anther to the stigma 2–3 mm, the stigma capitate, 1–1.25 mm wide. Berry 8–10 × 8–10 mm, light purple to purple-black. Seeds 0.3–0.5 mm long, ovoid, the testa smooth to roughened.

**Figure 123.**
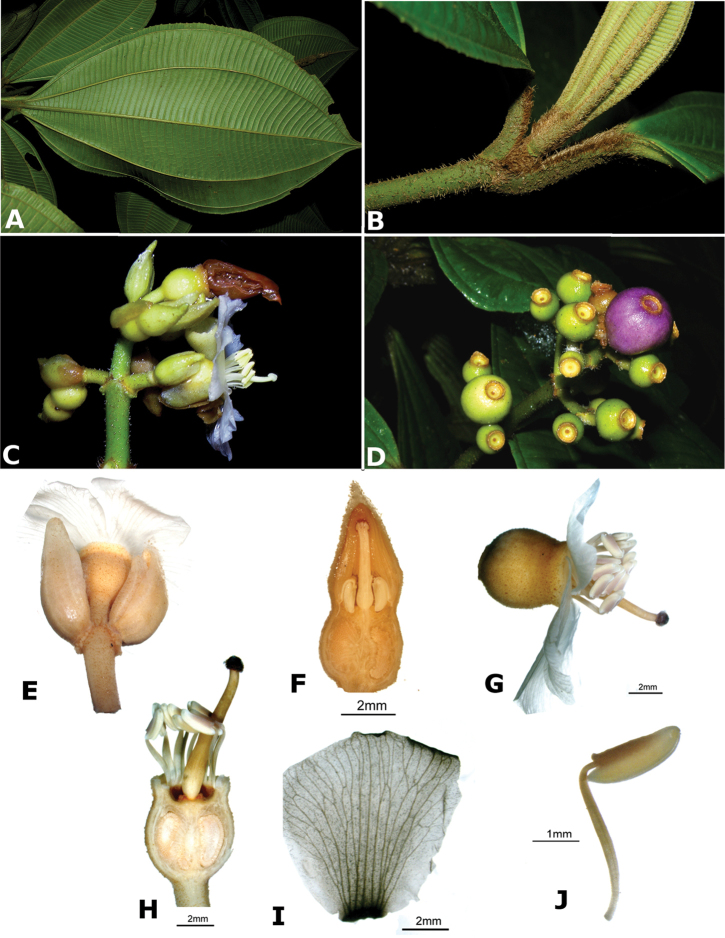
*Conostegia lasiopoda*. **A** Leaf abaxial surface **B** Internode **C** Lateral view of the flower **D** Fruit **E** Inflorescence branch showing bracteoles covering flower buds **F** Longitudinal section of a flower bud **G** Flower **H** Longitudinal section of a flower with petals removed **I** Petal **J** Stamen Photographs **A, B** by Reinaldo Aguilar and vouchered *R. Aguilar 11265* **C–J** vouchered from specimen *R. Kriebel 5651*.

#### Distribution

**Figure 124.**
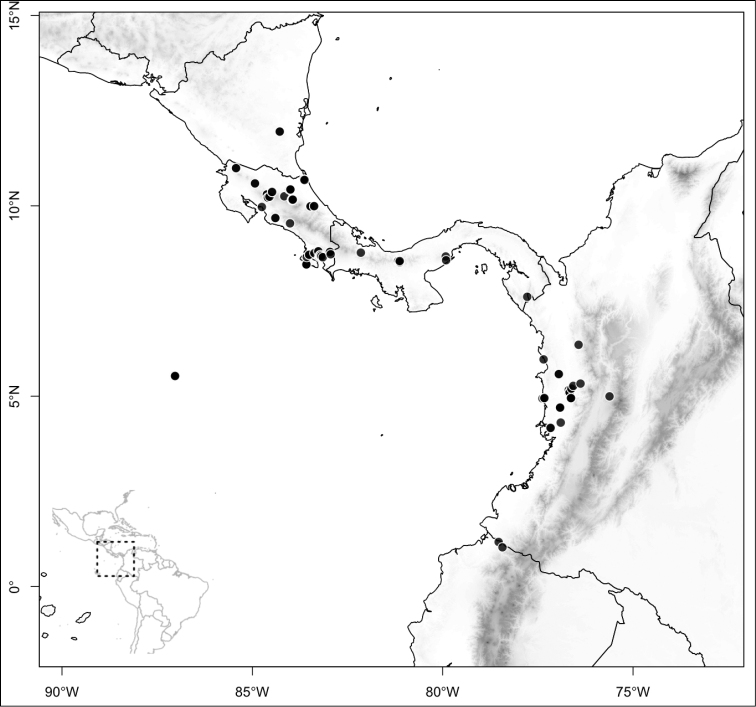
Distribution of *Conostegia lasiopoda*.

(Fig. 124). Distributed in southeastern Nicaragua, wet forests of Costa Rica, somewhat restricted in Panama to mostly western Colombia, with a few specimens collected in northwestern Ecuador, also in Cocos Island, from sea level to 2100 m in elevation.

*Conostegia lasiopoda* is usually a quite distinctive species. The leaf veins are elevated and arise exactly at the base (“perfectly nerved”), the petioles are setose adaxially and the flowers have foliaceous bracts covering them. When these bracts fall, distinguishing *Conostegia lasiopoda* from *Conostegia centronioides* can be difficult. The latter tends to be at least somewhat plinerved and lack the adaxial setose petioles. *Conostegia rubiginosa* is also similar but lacks the foliaceous bracts covering the floral bracts and the indument in flower bud is more evident. Also, *Conostegia rubiginosa* tends to have smaller coriaceous leaves. The stamens of *Conostegia lasiopoda* have a conspicuous anther shoulder (Figs 49, 123) which has not been confirmed in *Conostegia centronioides* or *Conostegia rubiginosa* and the style is exserted (Fig. 123) like the latter two taxa and as usual in section *Australis*. Lastly, the calyptra has been observed to rupture into pieces at anthesis (pers. obs. and photographs by Reinaldo Aguilar).

#### Specimens examined.

**NICARAGUA** (fide Schnell). **Zelaya**: Salto La Oropendula, Rio Rama, Stevens 8962 (MO).

**COSTA RICA. Alajuela**: San Ramón, R. B. Manuel Alberto Brenes, Estación Río San Lorenzo, Kriebel 902 (INB); **Limón**: Pococí, P.N. Braulio Carrillo, Estación Quebrada González, Kriebel 203 (INB); Matina, P. N. Barbilla, Cuenca del Matina, Sector Colonia Puriscaleña, Sendero Cerro Azul hasta Río Surubres, Mora 1162 (INB, MO, NY). **Puntarenas**: R.F. Golfo Dulce, Serranías de Golfito, Estación Río Bonito por la Fila que va a Cerro La Gamba, Aguilar and Albertín 5115 (NY); Distrito, Sierpe, Reserva Forestal Golfo Dulce, Mogos 1 km antes de llegar a la entrada del Porvenir a 16 km. de Chacarita, Aguilar 11265 (NY); E.B. Las Cruces, downhill from the greenhouses along the trail to the río Java, Boyle et al. 6435 (NY); along stream leading into Chatham Bay, Cocos Island, Fournier 303 (CR, NY); Bahía Wafer aguas arriba del Río Genio, González 1200 (INB, NY); Reserva Forestal innominada 2 K al norte de la entrada de Chacarita por la carretera interamericana, González 3594 (INB, NY); Estación La Gamba, Kriebel 1078 (INB).

**PANAMA. Panamá**: Sendero de Interpretación, 1 km al este del Campamento de los guardabosques de INRENARE, Correa and Montenegro 11122 (NY). **Veraguas**: Parque Nacional Santa Fé, aproximadamente de 3 a 6 km pasando la Escuela Agricola Alto de Piedra, Kriebel and Burke 5722 (NY, PMA).

**COLOMBIA. Antioquia**: P.N. Natural Las Orquideas, Vereda Venados Abajo, Pedraza et al. 2323 (NY). **Chocó**: Río San Juan, cercanías de Palestina, Cuatrecasas 16938 (NY); area of Baudó, on right bank of river Baudó about 12.5 km upstream of estuary, near estuary of Quebrada Carpio, 1 km upstream of camp site, Quebrada Angueradó, Fuchs and Zanella 21770 (MO, NY); At Río Iro on Hwy S from Istmina, Juncosa 2484 (MO, NY). **El Valle**: Río Calima (región del Chocó), entre Malaguita y Palestina, Cuatrecasas 21331 (NY).

**ECUADOR. Esmeraldas**: San Lorenzo cantón Ricuarte, Reserva Indígena Awá, Tipaz et al. 2107 (MO, NY).

### Conostegia monteleagreana

Taxon classificationPlantaeMyrtalesMelastomataceae

Cogn.

Fig. 125

Conostegia monteleagreana Cogn., DC. Monog. Phan. 7: 1189. 1891. Type: Costa Rica. Bord de la route a Carrillo, versant Atlantique, 300 m, 5 December 1890, H. Pittier 2539 (holotype: BR!; isotype: US!). (One of the US sheets is labeled Tonduz 2539 but is clearly of the same collection, fide Schnell (1996)).

#### Description.

Shrubs 1–2 m tall with tetragonal stems that are mostly glabrous with inconspicuous and scattered sessile stellate trichomes; the nodal line present yet slight. Leaves at a node equal to subequal in size. Petioles 0.2–2 cm, sometimes with large lenticels abaxially. Leaf blades 7.6–13 × 2.1–8 cm, 3-nerved to more frequently plinerved, if plinerved with the innermost pair of veins diverging up to 2 cm above the base in opposite or subopposite fashion, narrowly elliptic to elliptic-ovate, the base acute or obtuse and often short-decurrent, the apex acute to acuminate, the margin entire or remotely denticulate, essentially glabrous on both surfaces. Inflorescence a terminal panicle with few internodes on the main axis and each branch with the flowers agglomerated at the apex, 2.1–8 cm long, accessory branches apparently present as additional flowers in the glomerules, with green to red branches that can be heavily lenticellate, mostly glabrous, the foliaceous bracts 1–1.5 cm subtending the glomerules, each glomerule enveloped by ovate persistent bracteoles 5–9 mm long. Flowers sessile or with pedicels to 1 mm long. Flowers (4-)5(-7) merous, calyptrate. Floral buds 5–7 × 2.5–4 mm, oblong-pyriform, the base rounded, the apex apiculate, constricted about the middle, the calyptra and hypanthium weakly differentiated; the hypanthium 4–4.5 × 3.5–4 mm, essentially glabrous. Petals 4–5 × 4–4.25 mm, translucent white, obovate, spreading, glabrous, apically asymmetrical, closing after anthesis. Stamens (10-)13(-15), 3–4.5 mm long, radially arranged around the style, the filament 2–2.5 mm, white, anthers 1.5–2 × 0.4–0.6 mm, trapezoidal, whitish to pale yellow, forming a conspicuous anther shoulder at the filament anther junction, the pore ca. 0.1 mm, terminal. Ovary 6–9 locular, inferior, glabrous, forming a low collar around the style base. Style 4–5 mm, mostly straight, vertical distance between the anther and the stigma 0–1 mm, horizontal distance absent, stigma punctiform, 0.75–1 mm wide. Berry 5–7 × 5–7 mm, dark purple to black. Seeds 0.25–0.4 mm, pyriform, the testa minutely tuberculate.

**Figure 125.**
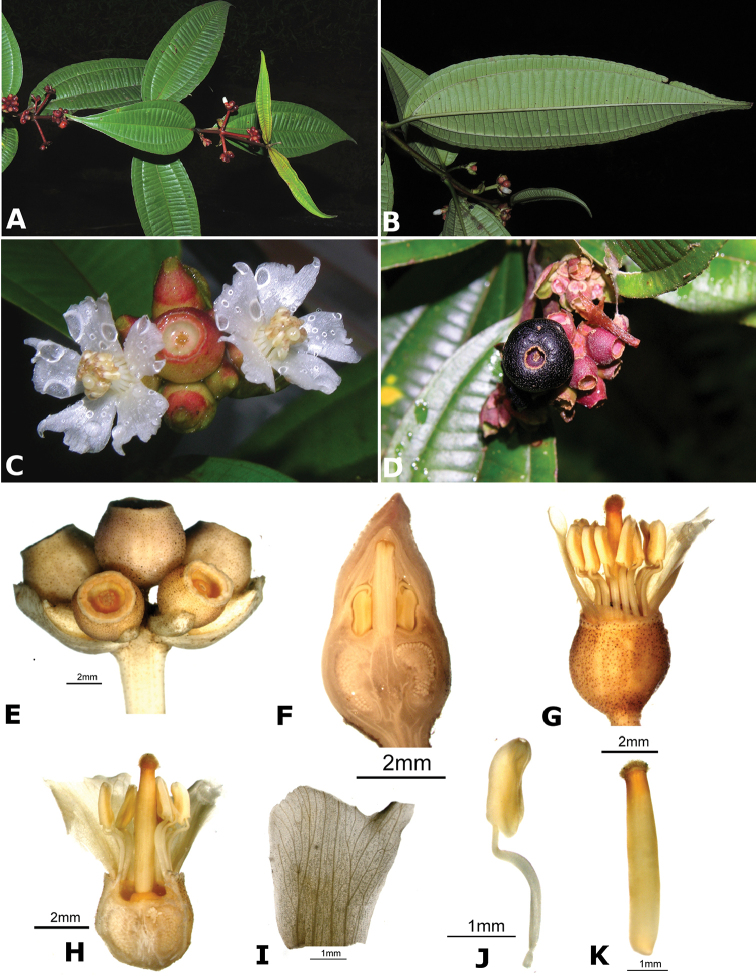
*Conostegia monteleagreana*. **A** Habit **B** Leaf abaxial surface **C** Close up of flowers at anthesis **D** Infructescence **E** Pickled inflorescence showing bracts subtending fruiting glomerule **F** Longitudinal section of a flower bud **G** Pickled flower **H** Longitudinal section of a flower at anthesis **I** Petal **J** Stamen **K** Style. Photographs **A–D** vouchered *R. Kriebel 5354* **E–K** vouchered *R. Kriebel s.n*.

#### Distribution

**Figure 126.**
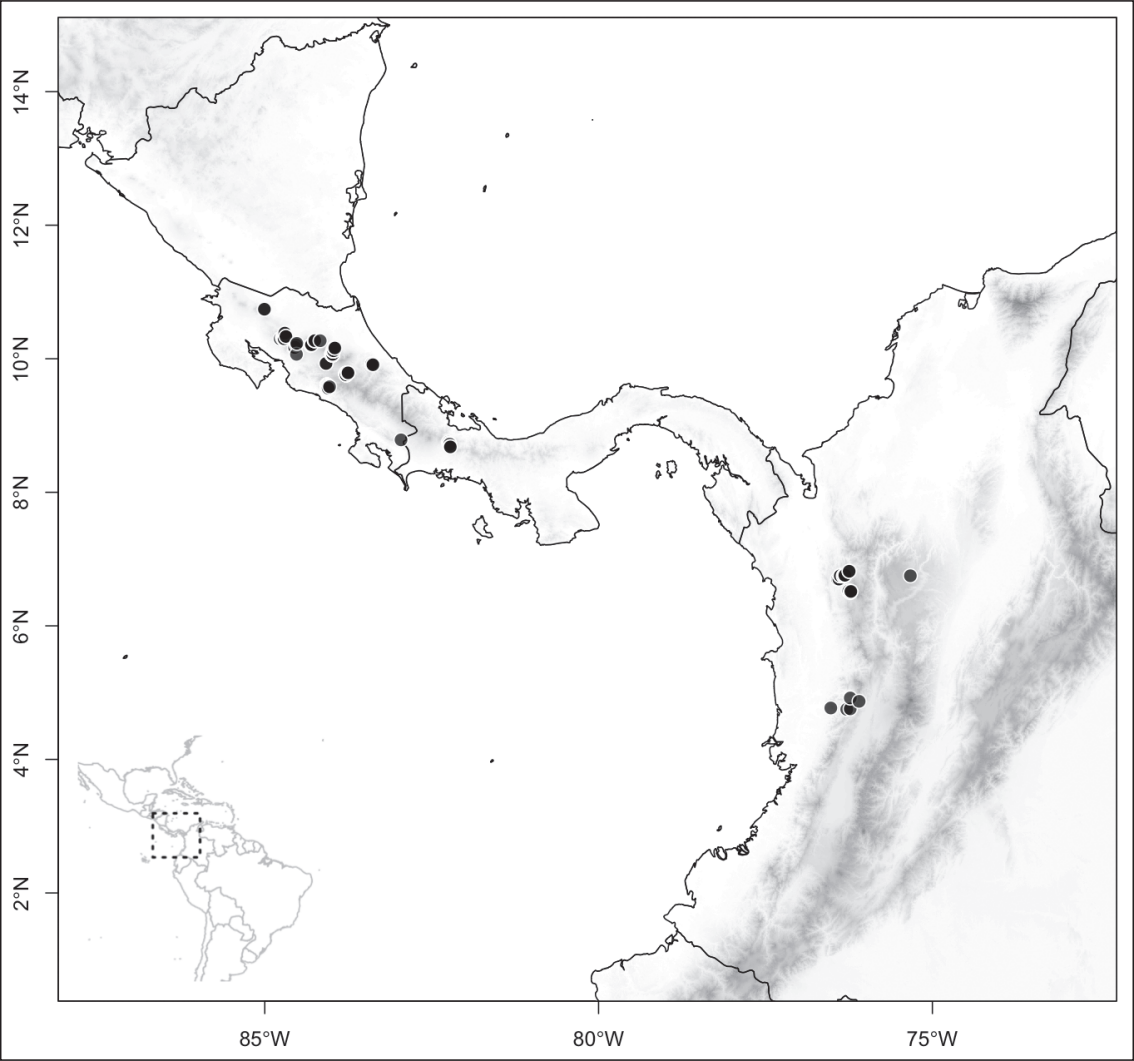
Distribution of *Conostegia monteleagreana*.

(Fig. 126). From Costa Rica to western Panama and then in the Cordillera Occidental of Colombia mostly in cloud forests from 500–2100 m in elevation.

*Conostegia monteleagreana* is easy to distinguish because of the foliaceous bracts that subtend the glomerulate inflorescences. The seeds of this species are also distinctive because of their particular tubercles. It is the only species in section *Australis* to have this testa ornamentation. This species has two morphotypes, one in Colombia with larger leaves and inflorescences than the one present in Costa Rica and Panama. It also appears to vary in the degree of herkogamy. The distinction between the insertion of the anther and filament (“anther shoulder”) is very conspicuous as in other species of section *Australis*. Schnell (1996) states that the name is a misspelling of the last name Montealegre.

#### Specimens examined.

**COSTA RICA. Alajuela**: about 2–7 km SE of Cataratas de San Ramon, Almeda et al. 4306 (CR, MO, NY); Los Angeles de San Ramón, Brenes 6103 (CR, NY); Piedades cerca de San Ramón, Brenes 4256 (CR, NY); Finca La Paz, San Ramón, Kriebel 1475 (INB, NY); Pueblo Nuevo San Carlos, Smith 1909 (CR, NY). **Cartago**: Selva entre Quebrada Selva y Río Taus, Morales 12915 (INB, NY); P.N. Tapantí, a lrededores de la estación, Kriebel 5354 (INB, NY); Cantón de Paraíso, cuenca del Reventazón, Orosí, entrada a Tausito aprox. 3 km después del cruce hacia la Estación Tapantí, Rodríguez et al. 3241 (INB, MO, NY). **San José**: Vázquez de Coronado, P.N. Braulio Carrillo, Sendero de la Ventana al Bajo de la Hondura, Acosta and Ramírez 515 (INB, MO NY); Tarrazú, San Lorenzo, estribaciones sureste de Cerro Toro, Estrada et al. 701 (CR, MO, NY).

**PANAMA. Bocas del Toro**: Fortuna Dam Area along continental divide trail bordering Chiriquí Province, Almeda 6075 (CAS, NY, PMA). **Chiriquí**: comú n al lado de la carretera cerca de la estacion de STRI, Kriebel and Burke 5747 (NY, PMA); Near Lago de Fortuna. Km 43 from Chiriquí on road to Chiriquí Grande, Penneys and Blanco 1719 (FLAS, NY).

**COLOMBIA. Antioquia**: Mun. Frontino, Corregimiento Nutibara, Región Murí, Acevedo-Rodríguez 1264 (NY); Mcpio. Frontino, Corrig. Nutibara, región de Murrí, Altos de Cuevas, Luteyn et al. 11724 (MO, NY); Valle/Chocó border area, Mpio. El Cairo, Correg. Boquerón, Vereda Las Amarillas, Serranía de los Paraguas, ca. 21–25 km beyond El Cairo, Luteyn and Giraldo 12682 (NY); Urrao, Corregimiento La Encarnación, vereda Calles, P. N. Natural Las Orquídeas, en el camino de la cabana a la quebrada El Agudelo, Pedraza 1983 (NY). **Chocó**: Along road between San José del Palmar and Cartago at Vereda San Antonio between San José del Palmar and jct. in road to El Cairo, Croat 56664 (MO, NY); Cerro del Torra vertiente nororiental abajo del helipuerto, Silverstone et al. 1144 (COL, NY). **El Valle**: Cordillera Occidental vertiente occidental, Hoya del río Digua, Quebrada de San Juan abajo de Queremal, Cuatrecasas 22727 (NY).

### Conostegia ortizae

Taxon classificationPlantaeMyrtalesMelastomataceae

Kriebel sp. nov.

urn:lsid:ipni.org:names:77156242-1

Fig. 127

#### Type.

Ecuador. Napo: Estación Biológica Jatun Sacha, Sendero Río Napo, 14 November 2005, D. Penneys 1857 (holotype NY!; isotype: FLAS, MO).

#### Description.

Shrubs or small trees to 3–6 m tall with flattened and grooved stems that are covered with an orangish mixture of long and shorter-branching stellate trichomes, the long branching ones graceful and sessile, sometimes also becoming dendritic; the nodal line present yet slight. Leaves at a node equal to subequal in size. Petiole 0–1.5 cm, grooved above, usually entirely covered by the decurrent leaf base. Leaf blades 17–37 × 8–15 cm, 3–5 plinerved, with the innermost pair of veins diverging above the base in fashion, broadly elliptic to obovate, decurrent at the base, the apex long-caudate up to 3.5 cm long, margins entire to undulate-denticulate, glabrous adaxially, abaxially with indument like that of the stem. Inflorescence a terminal panicle 4–13 cm long with accessory branches; bracteoles 2–3 mm long, linear, tending to persist. Pedicels 1–2 mm. The hypanthium 3–4 × 4.5–5 mm, pubescent with long-and shorter-branching stellate trichomes. Flowers 6–8 merous, calyptrate; floral buds 7–11.5 mm, not constricted below the calyptra. Petals 10.5–12 × 8.25–8.75 mm, white, obtriangular, spreading, glabrous on both surfaces. Stamens (26-)34–52, slightly zygomorphic, the filament 4–4.5 mm, white, anthers 3.5–4 × 0.5–0.75 mm, linear subulate, pale yellow, the pore ca. 0.15 mm, slightly ventrally inclined. Ovary 12–13 locular, inferior; style ca. 11–12 mm long mm, straight but gently bending from the base, glabrous, distance from the anther to the stigma ca. 3.5–4 mm, stigma subcapitate, 1.4–1.7 mm wide. Mature berry ca. 8–9 × 8–9 mm when dry.

**Figure 127.**
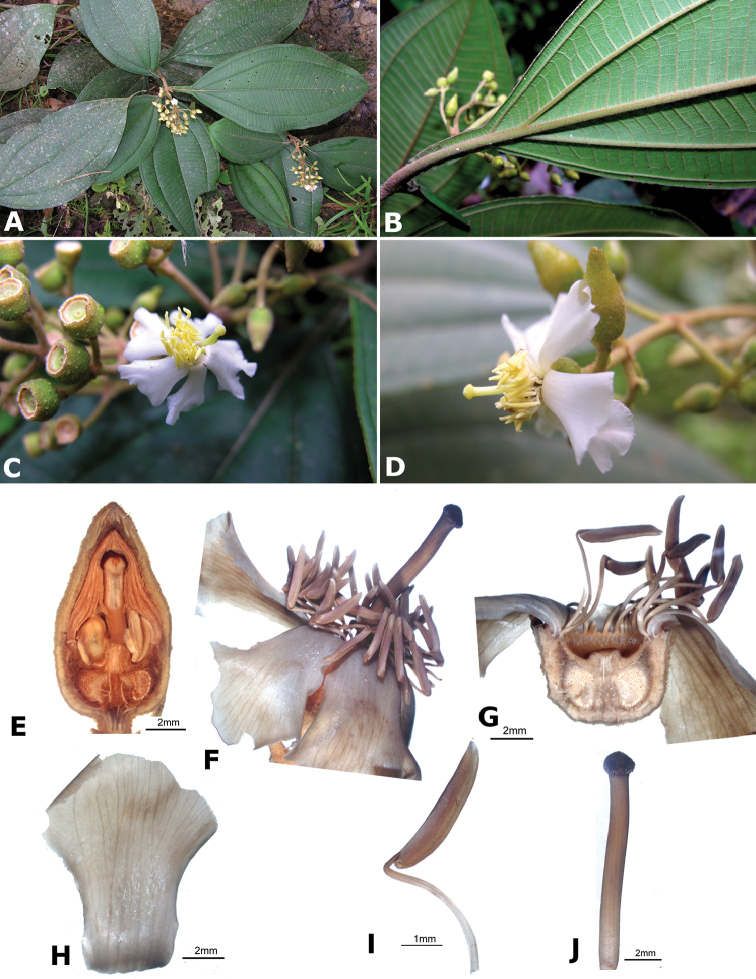
*Conostegia ortizae*. **A** Fertile branch **B** Abaxial surface of leaf showing decurrent base **C** Close up of the frontal view of the flower **D** Close up of the lateral view of the flower. Note exserted style **E** Longitudinal section of a flower bud **F** Pickled flower at anthesis **G** Longitudinal section of a flower at anthesis with the style removed **H** Petal **I** Stamen **J** Style. Photographs taken by Darin Penneys and vouchered *D. Penneys 1857*.

#### Distribution

**Figure 128.**
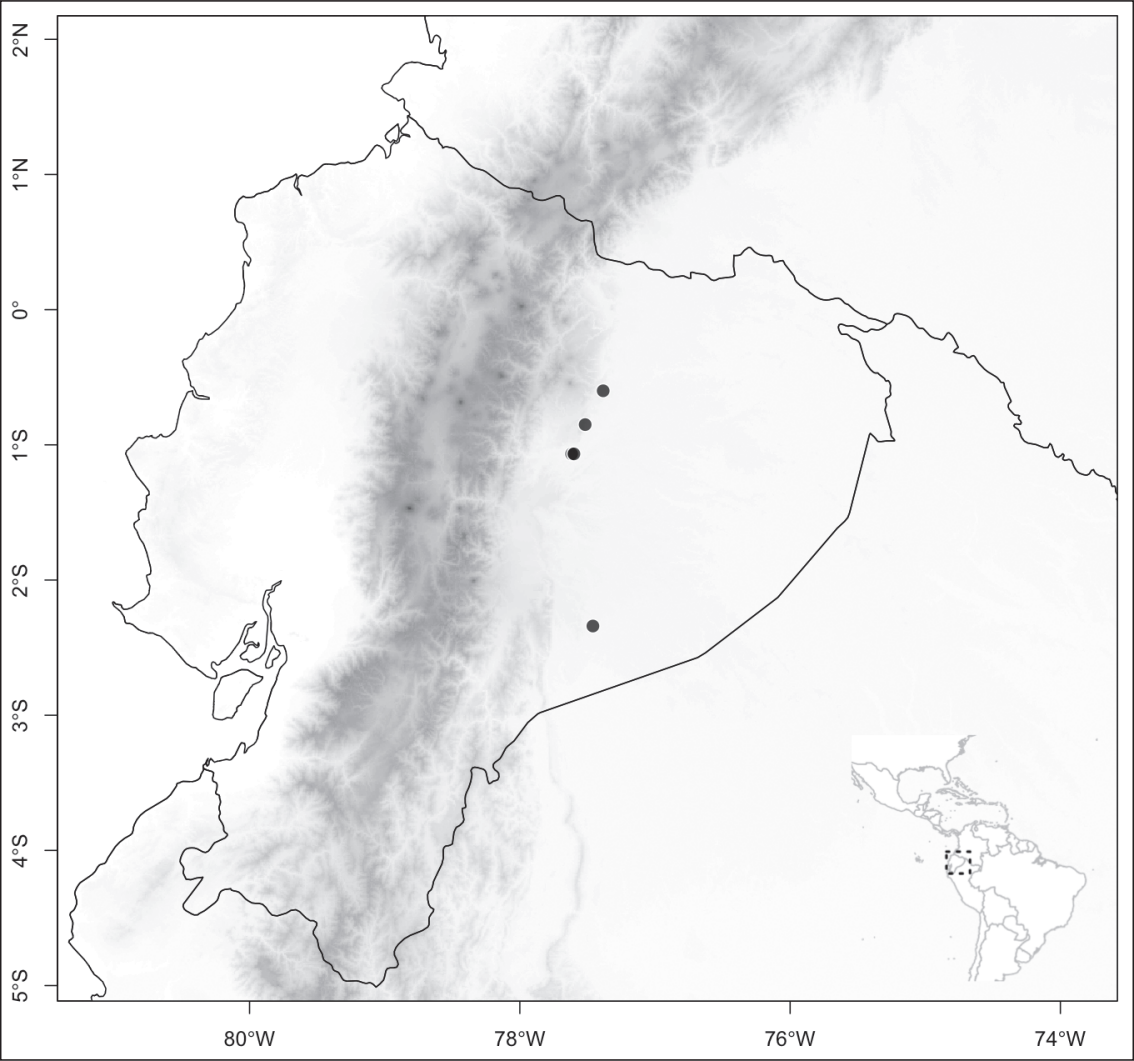
Distribution of *Conostegia ortizae*.

(Fig. 128). Northeastern foothills of the Ecuatorian Andes with one more southern population in Ecuador, at 250–1250 m in elevation.

*Conostegia ortizae* can be easily recognized by its large sessile leaves with an evidently decurrent leaf base and strongly plinerved venation. Of the species for which the anatomy of the style has been studied, this is the only one in section *Australis* to have a stele within the style.

#### Etymology.

This species is dedicated to the Ecuadorian biologist Patricia Ortiz who tragically passed away in her second home of Monteverde, Puntarenas, Costa Rica. Pati was one of the best and most passionate naturalists I have had the fortune to meet.

#### Specimens examined.

**ECUADOR. Napo**: Cantón Archidona, North bank Río Suno, 15 km NW of Loreto, 8 km W of El Progreso, Neill et al. 9153 (MO, NY); Estación Biológica Jatun Sacha, Río Napo 8 km al E de Misahualli, Palacios 2510 (MO, NY); Archidona, Parque Nacional Galeras, a 1.5 km de la comunidad Santa Rosa de Arapino, faldas cordillera de Galeras, Bloque 19 línea sísmica 28, Compania Triton, Vargas and Grefa 943 (MO, NY). **Santiago-Zamora**: Taisha, Río Guaguayme, Cazalet and Pennington 7545 (NY).

### Conostegia polyandra

Taxon classificationPlantaeMyrtalesMelastomataceae

Benth.

Fig. 129

Conostegia polyandra Benth., Bot. Voy. Sulph. 96, pl. 35. 1844. Type: Colombia. San Pedro, 1841, R. Hinds s.n. (lectotype: K!, isotypes: GH!, LE, fragment BR).
Miconia rupicola Gleason, Bull. Torrey Bot. Club 52: 383. 1925. Type Colombia. EL Valle: exposed cliffs, Buenaventura, 0–10 m elev., 5–10 October 1922, E. Killip 11685 (holotype: NY!; isotypes: GH!, US!).

#### Description.

Small trees 1–13.6 m tall with flattened stems that become terete with age and are sparsely to densely puberulent with sessile stellate hairs; the nodal line present yet slight. Leaves at a node equal to subequal in size. Petiole 0.4–3.5 cm long. Leaf blade 4–14 × 2–7.3 cm, 3–5 nerved, usually elliptic to elliptic ovate, the base rounded to obtuse, the apex acute or short acuminate, the margin serrulate and ciliate, the adaxial surface glabrous, the abaxial surface sparsely to densely puberulent with sessile stellate hairs and minute white rounded and roughened secretions. Inflorescence a terminal panicle 3–15 cm long, accessory branches absent or less frequently present, the rachis with sessile stellate and stalked stellate hairs, the bracteoles 1–8 mm long, linear to ovate, persistent or deciduous. Pedicel 1–5 mm long. Flowers 5–8-merous, calyptrate, floral buds 6.5–10 × 3.25–6.25 mm, elliptic-pyriform, the base obtuse to rounded, the apex apiculate, slightly constricted below the calyptra, the calyptra and hypanthium not differentiated; the hypanthium 3.5–4 × 5.25–5.75 mm, stellate puberulent. Petals 7–9.5 × 5.25–9 mm, translucent white to white or pink, obtriangular to obovoid, spreading, glabrous, apically slightly bilobed to emarginate. Stamens 26–36, 5–7 mm long, radially arranged around the style, the filaments 3–4 mm, white, without an evident geniculation, anthers 2–3 × 0.4–0.6 mm, yellow, the pore ca. 0.25 mm. Ovary 6–8 locular, inferior, apically glabrous, forming a very low to absent collar around the style base. Style 8–10 mm, basically straight but slightly bending along its length, vertical the distance from the anthers to the stigma ca. 2.5–3.5 mm, horizontal distance absent, stigma capitellate, 0.5–0.75 mm wide. Berry 6–8 × 6–8 mm, dark purple. Seeds 0.45–0.7 mm long, obliquely pyramidal and somewhat angulate, the testa smooth to roughened.

**Figure 129.**
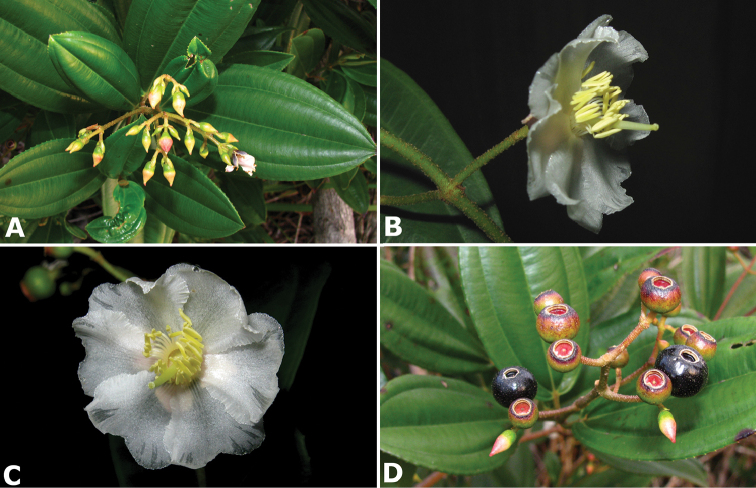
*Conostegia polyandra*. **A** Habit and inflorescence with flower buds **B** Close up of the lateral view of the flower **C** Close up of the frontal view of the flower **D** Infructescence. Photographs **A, D** taken by Xavier Cornejo and vouchered *X. Cornejo 8126*, photographs **B, C** taken by Frank Almeda and vouchered *F.Almeda 10481*.

#### Distribution

**Figure 130.**
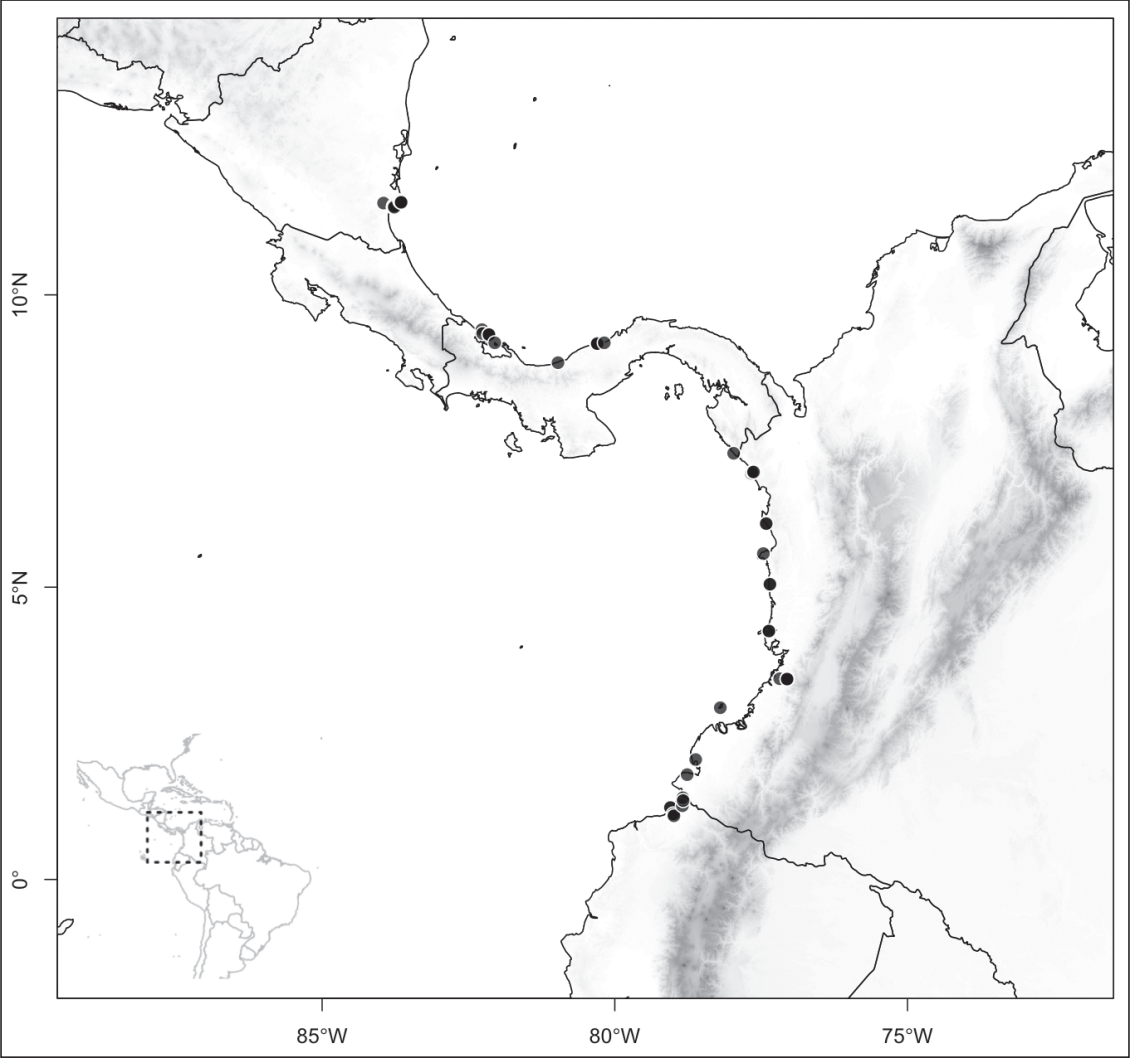
Distribution of *Conostegia polyandra*.

(Fig. 130). On the Caribbean coast of Nicaragua, both coasts of Panama and on the Pacific coast of Colombia and Ecuador and a disjunct population in Peru, sea level to 200(-600) m elevation.

*Conostegia polyandra* has white secretions on the leaf abaxially. Since this species grows in mangrove habitats, these secretions are probably salt. This is the only species of Neotropical Melastomataceae known to grow in mangroves. It can further be recognized by its 3–4 nerved leaves which tend to have a consistent elliptic shape and serrulate and ciliate margins. Flowers in this species are also noticeable for their great number of stamens and strongly exserted styles. Schnell (1996) and Almeda (2009) commented on the phenological patterns of this species citing its continuous flowering and fruiting throughout the year. In addition these authors stated that the this phenological behavior coupled with few large seeds per fruit might help explain its weedy tendencies. I note here that the specimens I studied did not have particularly few large seeds per fruit. Schnell (1996) suggests *Conostegia polyandra* is aggressive and somewhat weedy, reminiscent of *Conostegia speciosa* and *Conostegia xalapensis*. Its apparent absence in coastal areas in the Caribbean of Costa Rica might be explained by its replacement in these areas by *Conostegia xalapensis* (Schnell 1996). *Duke and Idrobo 11567* (MO, NY) reported the berries as “quite edible”.

#### Specimens examined.

**NICARAGUA** (fide Schnell). **Zelaya**: Monkey Point, Stevens 20003 (MO).

**PANAMA. Bocas del Toro**: Island of Bocas del Toro, along stream below dam at Bocas, Durkee 71 (MO, NY); Isla Colón, Wedel 75 (NY); Isla Colón, Southwest of Bocas at Maccaw Hills, Wedel 531 (NY); Old Bank Island, vicinity of Chiriquí Lagoon, Wedel 1876 (MO, NY). **Colón**: Miguel de la Borda, Croat 10053 (MO, NY).

**COLOMBIA. Cauca**: Buenaventura, Pacific Coastal Zone, Pittier 1507 (NY). **Chocó**: Municipio de Nuquí, corregimiento Termales, entre Jobe y Arusi, bosque cerca a las cabañas Pijibá, Costa Pacífica colombiana, estribaciones de la serranía del Baudó, Cabo Corrientes, Betancur et al. 6044 (NY); Strand flora between Camp Curiche and Q. Changame, 3.7 miles S. of Camp Curiche, Duke and Idrobo 11567 (MO, NY); Costa del Pacífico, Coredó, Fernandez 367 (NY); area Baudó, about 3 km north of Rio Baudó, on Pacific coast, Fuchs and Zanela 22133 (MO, NY); Pizarro, von Sneidern 4878, 4888 (NY). **El Valle**: Corregimiento Termales, coastal zone between quebrada Piedra Piedra and Rio Terco, Acevedo-Rodríguez et al. 6905 (MO, NY); Buenaventura, Alston 8639 (NY); Costa del Pacífico, río Yurumanguí, Cuatrecasas 15890 (NY); El Valle, Gentry and Fallen 17234 (MO, NY); Rio Cajambre, Haught 5352 (NY); North shore of Buenaventura Bay, Playa Basán, Killip and Cuatrecasas 38690 (NY). **Nariño**: Tumaco, Dryander 2610 (NY); South end of Gorgona Island, Killip and Garcias 33098 (NY); Gorgonilla Island, Killip and Garcia 33112 (NY).

**ECUADOR. Esmeraldas**: Limones-Borbón, 5 km before Borbón, Holm-Nielsen et al. 26028 (MO, NY); San Lorenzo, Játiva and Epling 754 (NY); Santiago Estuary at Lagartera near La Tolita, Játiva and Epling 1175 (MO, NY); La Guayacana near Pichangal, Játiva and Epling 2112 (NY).

**PERU** (fide Schnell). Without locality, Maclean s.n. (GH).

### Conostegia rubiginosa

Taxon classificationPlantaeMyrtalesMelastomataceae

Gleason

Fig. 131

Conostegia rubiginosa Gleason, Bull. Torrey Bot. Club 72: 473. 1945. Type: Colombia. El Valle: Quebrada de Guapecito, rio Cajambre, Costa del Pacifico, 0–5 m, 16 May 1944, J. Cuatrecasas 17700 (holotype: NY!, isotypes: F!, US!).

#### Description.

Shrubs to trees 3–6 m tall with tetragonal to terete and slightly ridged stems that are covered with a mixture of sessile stellate and stalked-stellate hairs sometimes intermixed with simple hairs or sometimes glabrescent and becoming glabrous with age; the nodal line present (sometimes obscured by indument). Leaves at a node equal to subequal in size. Petioles 0.4–2.5 cm. Leaf blades 5.5–25 × 3–9 cm, 3–5 nerved or less frequently plinerved, if plinerved with the innermost veins diverging from the midvein up to ca. 1 cm above the base in opposite or alternate fashion, elliptic to ovate, the base obtuse to acute, acuminate to caudate, the margin entire to denticulate, the adaxial foliar surface essentially glabrous, the abaxial surface densely pubescent with rusty brown stellate and branching trichomes, sessile or short stipitate, especially on the main veins and lightly pubescent to glabrescent on the surface. Inflorescence a terminal panicle 5.5–13 cm long, accessory branches present or absent, the rachis pubsecent with brown stellate and branching trichomes, the bracteoles to 4 mm long, lanceolate to ovate, persistent or caducous. Flowers sessile or with pedicels up to 2 mm long, 6–7 merous, calyptrate. Floral buds 5.5–11 × 3.7–5.5 mm, obovoid, the base flat to rounded, and apex broadly acute, slightly to not constricted near the torus; the hypanthium 4.5–6 × 4.5–6 mm, rusty brown pubescent and sometimes sparsely tuberculate. Petals 7–9 × 6–8.5 mm, translucent white, obovate, spreading, glabrous, asymmetrical. Stamens 15–24, 5.5–6.5 mm, slightly zygomorphic, the filaments 2.75–3.25 mm, white, anthers 2.5–3 × 0.5–0.75 mm, linear to elliptic, laterally compressed and with an anther shoulder, yellow, the pore ca. 0.1 mm, subterminal and slightly ventrally inclined. Ovary 6–7 locular, inferior, apically glabrous and with a low collar around the style base. Style 6–7 mm long, straight to bending sideways, vertical distance from the anthers to the stigma 1.75–2.5 mm, horizontal distance 0–1 mm; stigma capitate, ca. 1 mm wide. Berry and seeds not seen.

**Figure 131.**
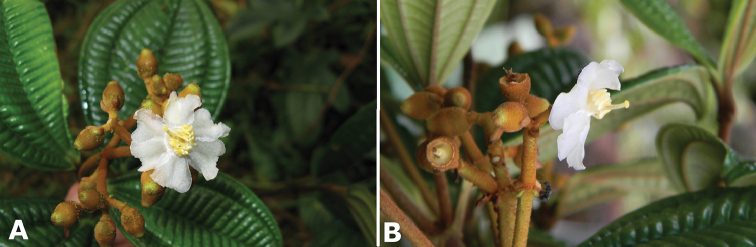
*Conostegia rubiginosa*. **A** Inflorescence and frontal view of a flower **B** Lateral view of the flower. Note exserted style. Photographs taken by Frank Almeda and vouchered *F. Almeda 10426*.

#### Distribution

**Figure 132.**
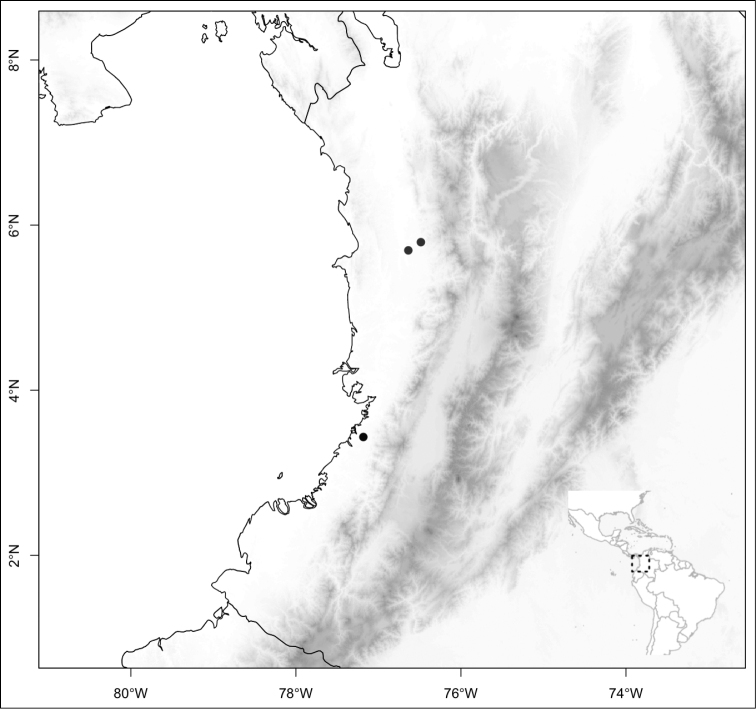
Distribution of *Conostegia rubiginosa*.

(Fig. 132). West coast of Colombia, sea level to 100 m elevation.

For the few specimens studied, *Conostegia rubiginosa* is quite variable in leaf size and consistency. *Conostegia rubiginosa* can be confused with *Conostegia centronioides*, *Conostegia lasiopoda* and *Conostegia rufescens*. From *Conostegia centronioides* it can be distinguish by the larger flowers without a constriction in the hypanthium, and denser reddish indument. From *Conostegia rufescens* it can be distinguished by its exserted style. For differences between this species and *Conostegia lasiopoda*, see the discussion under the latter.

#### Specimens examined.

**COLOMBIA. Chocó**: Municipio de Atrato, Corregiemiento Doña Josefa, road between Quibdo and Municipio de Atrato (formerly Yuto), Almeda et al. 10426 (CAS); Carretera Quibdó-Guayabal, Forero and Jaramillo 2783 (COL). **El Valle**: Costa del Pacífico, río Cajambre, Silva, Cuatrecasas 17538 (NY).

### Conostegia tenuifolia

Taxon classificationPlantaeMyrtalesMelastomataceae

Donnell Smith

Fig. 133

Conostegia tenuifolia Donnell Smith, Bot. Gaz. 27: 334. 1899. Type: Costa Rica. Limón: April 1896, J. Smith 6571 (lectotype: BR!, designated here; isolectotypes: BM, GH, US!). Other syntypes: Costa Rica. Limón: Río Verde, Llanuras de Santa Clara, Costa Rica, 250 m, May 1896, J. Smith 6574 (BR!, F!, K! (fide Almeda), US); forets de Shiroles, Talamanca, Costa Rica, ca.100 m, February 1895, A. Tonduz 9339 (BR!, NY!, US!).

#### Description.

Shrub or small tree 1.5–4 (-8) m tall with young tetragonal stems that then become terete and which are glabrescent with some minute stellate trichomes scattered throughout; the nodal line present yet slight. Leaves at a node equal to subequal in size. Petioles 0.3–3 cm. Leaf blades 5.9–19 × 2.1–9 cm, 3–5 plinerved, with the innermost pair of primary veins diverging from the midvein up to 1.5 cm above the base in opposite to alternate fashion, the secondaries obscure, elliptic to obovate, the base acute ro rounded, the apex obtuse to acute and caudate, the margin entire, the adaxial surface glabrous, the abaxial surface glabrous except for tiny stellate hairs on the veins. Inflorescence a terminal few flowered panicle 2.2–8 cm long, accessory branches absent or if present frequently reduced to single flowers the rachis glabrescent with minute stellate hairs, the bracteoles up to 1mm long, linear to ovate, deciduous. Pedicels 1–2.2 mm. Flowers (5-)6(-7) merous, calyptrate. Floral buds 6–9 × 3–5 mm, globose, wider below the torus, the base truncate to rounded, the apex apiculate, constricted in the middle, the hypanthium 4–5 × 4–5 mm, sparsely stellate with minute trichomes. Petals 6–8.25 × 6–7.25 mm, translucent white, obtriangular, spreading at anthesis, apically asymmetrical, glabrous. Stamens 18–24, 3.5–5 mm long, radial to slightly bilateral apparently from interactions with the style, the filament 2–3 mm long, white, anthers 1–2 × 0.5–0.75 mm, oblong, forming an anther shoulder at the filament anther junction, yellow, the pore 0.1–0.2 mm, terminal. Ovary (6-)8–12 locular, inferior, apically glabrous and forming a low inconspicuous collar around the style. Style 5–6 mm long, straight with the stigma turning slightly upward, vertical distance from the anther pore to the stigma ca. 3–3.5 mm, horizontal distance absent; stigma capitellate, 1–1.25 mm wide. Berry 5–7 × 5–7 mm, purple black. Seeds 0.25–0.45 mm long, ovoid, the testa smooth.

**Figure 133.**
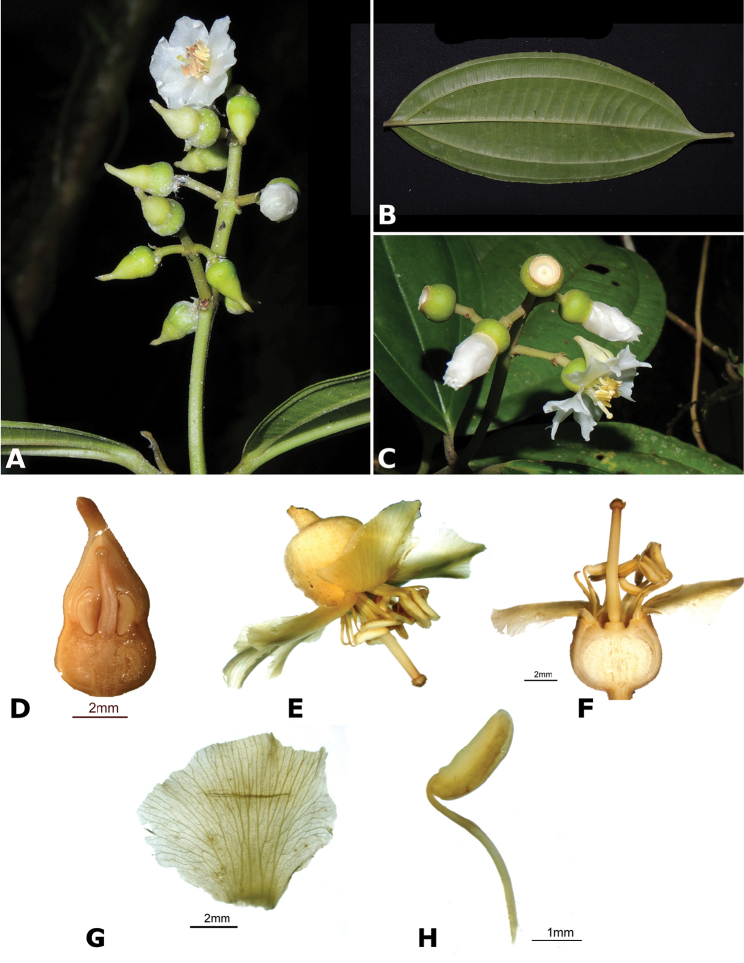
*Conostegia tenuifolia*. **A** Inflorescence **B** Leaf abaxial surface **C** Lateral view of a flower. Note exserted style **D** Longitudinal section of a flower bud **E** Pickled flower **F** Longitudinal section of a pickled flower **G** Petal **H** Stamen. Photographs from specimen vouchered *R. Kriebel 5773*.

#### Distribution

**Figure 134.**
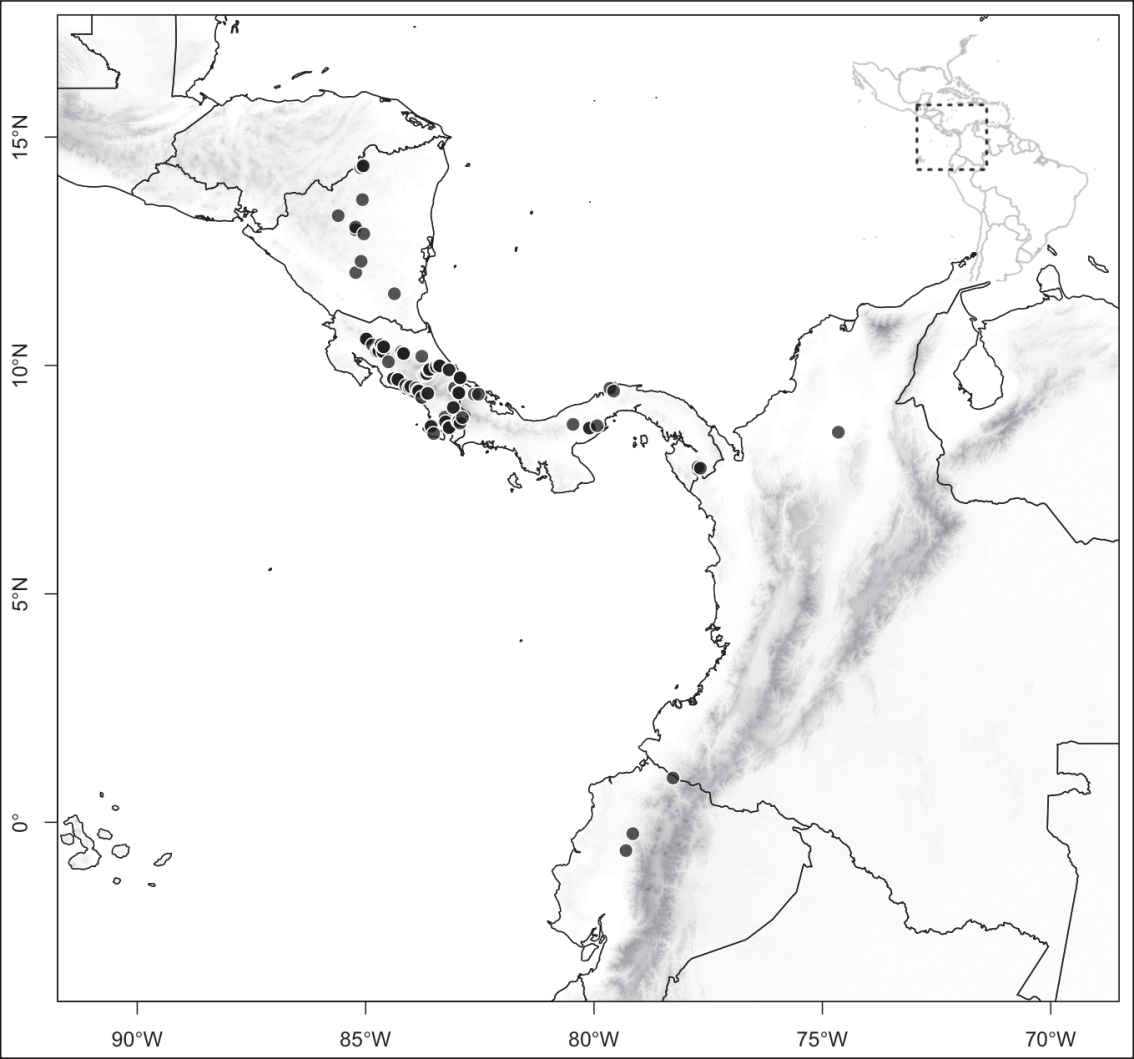
Distribution of *Conostegia tenuifolia*.

(Fig. 134). Nicaragua, Costa Rica, Panama, Colombia and reaching Ecuador, most common below 1000 m in elevation but reaching 1600 m.

This species is easy to recognize because of its glabrosity, plinerved leaves with entire margins and caudate apices, few flowered inflorescences, apiculate calyptra, and exserted styles with a capitate stigma. In addition, *Conostegia tenuifolia* has an evident anther shoulder. The calyptra of *Conostegia tenuifolia* has been observed to rupture longitudinally at one site in San Vito de Coto Brus, Puntarenas, Costa Rica (Fig. 133). The specimens from Peru (*Kayap 955* and *Berlin 723*, both at NY), resemble this species, but more flowering specimens are required to assess their identity.

#### Specimens examined.

**NICARAGUA** (fide Schnell). **Chontales**: 4 km NW Santo Domingo, Grijalva and Soza 3739 (MO). **Jinotega**: Comarca de Bocayito, 117 km from Matagalpa, Cordillera Isabelia, near Rio Bote, Neil 7171 (GH). Matagalpa: NW del Cerro Musun, sobre el filo de la montana, a partir trocha a Paylo, Araguistain and Moreno 2580 (MO). **Zelaya**: Cerro EI Escobin, 4 km from Colonia Serrano, Sandino 3368 (MO).

**COSTA RICA. Alajuela**: R.V.S. Bosque Alegre, Laguna Hule, Kriebel and Larraguivel 667 (INB); San Carlos, Fortuna, R. B. Arenal Mundo Aventura, Rodríguez 8979 (INB, NY); San Ramón, Peñas Blancas, Chachagua, Finca Propiedad de la Asociación de Desarrollo, Alrededores de Quebrada Chachagita en cercanías del pueblo, Rodríguez 11693 (INB, NY). **Cartago**: Jiménez, Tausito, Río Tausito, Santamaría and Morales 4343 (INB, NY); rain forest area near Pavones about 15 kms east of Turrialba, Williams 19704 (NY). **Limón**: Matina, P. N. Barbilla Sector Colonia Puriscaleña, sendero Cerro Azul hasta Río Surubres, Mora 1168 (INB, MO, NY). **Puntarenas**: Coto Brus, Jardín Botanico Wilson, Kriebel 3215 (INB); loc cit. Moran and Hernandez 7950 (NY). **San José**: Pérez Zeledon, Basin of El General, Skutch 5102 (CR, MO, NY).

**PANAMA. Bocas del Toro**: Río Teribe, between Quebrada Huron and Quebrada Schlunjik, Kirkbride and Duke 470 (NY). Darién: 0–2 miles E of Tres Bocas along the shortest headwater of Río Coasi, Kirkbride and Duke 1189 (NY).

**COLOMBIA. Antioquia** (fide Schnell): Mpio. Gómez Plata, 10–15 km en la via Barboza Porce-Amalfi, N de Barboza, Río Medellin, límites Mpio. Yolombó, Callejas et al. (US). Boyacá: Region of Mt. Chapon, extreme western part of Dept. Boyaca northwest of Bogotá, Lawrence 340 (NY).

**ECUADOR** (fide Schnell). **Carchí**: trail to Pailon encampment, Gualpi Chico area of Awá reserve, Hoover et al. 3617 (MO). **Pichincha**: Cantón Santo Domingo, 12 km E of Patricia Pilar, Dodson et al. 7243 (MO).

### Conostegia sect. Geniculatae

Taxon classificationPlantaeMyrtalesMelastomataceae

Kriebel sect. nov.

urn:lsid:ipni.org:names:77156243-1

#### Diagnosis.

Leaves frequently conspicuously plinerved, calyx not calyptrate or the few species with a calyptra then the latter lacking sclereids. Several species have fused calyces in bud that rupture irregularly. Flowers mostly diplostemonous and herkogamous, with the stamens bearing a conspicuous geniculation towards the apex, anthers radially arranged around the exserted style except for the calyptrate species which have short styles. These short styled species resemble species of section *Conostegia* but lack a stele in their internal anatomy. Species in this section are almost entirely endemic to Costa Rica and Panama with a few species reaching South America and a few others northern Central America. Only *Conostegia xalapensis* reaches the Caribbean.

#### Type.

*Conostegia cinnamomea* (Beurl.) Wurdack

### Conostegia allenii

Taxon classificationPlantaeMyrtalesMelastomataceae

(Almeda) Kriebel comb. nov.

urn:lsid:ipni.org:names:77156244-1

Fig. 135

Conostegia allenii (Almeda) Kriebel. Basionym: Clidemia allenii Almeda, Proc. Calif. Acad. Sci. Series 4, 55(4): 90, f. 1. 2004. Type: Costa Rica. Puntarenas: Cantón de Osa, Golfo Dulce Area in the vicinity of Esquinas Experiment Station at sea level, 16 April 1949, P. H. Allen 5265 (holotype: CAS!, isotypes: AAU, CR, INB!, MEXU, MO, US!).

#### Description.

Shrub to small trees 1.5–5 m tall with terete to flattened internodes that are moderately to copiously covered with smooth spreading hairs 1.5–3 mm long, sometimes underlain with a sparse to moderate understory of sessile stellate and stipitate-stellate hairs; the nodal line not evident. Leaves at a node equal to somewhat unequal in length. Petiole absent or up to 1.2 cm long. Leaf blades 3.5–23 × 1.8–10 cm, 5–7 plinerved with the innermost pair of primary veins diverging from the midvein 0.7–1.5 cm above the base and arising in alternate fashion, elliptic to elliptic-ovate, oblique and rounded, acuminate to attenuate, entire and ciliate varying to obscurely denticulate distally, adaxially moderately covered with spreading hairs 1–2.5 mm long to nearly glabrous, abaxially moderately to sparingly covered with spreading simple hairs 1–3 mm long on the median vein and innermost primaries and sparsely underlain with sessile stellate and stipitate-stellate hairs varying to glabrate. Inflorescence a pseudolateral modified dichasium 2.6–9 cm long sometimes divaricately branched from the base, accessory branches absent, the branches reddish green to red, moderately to copiously covered with smooth spreading hairs, sometimes underlain with a sparse to moderate understory of sessile stellate and stipitate-stellate hairs, bracteoles 1–6 mm long, subulate to narrowly triangular, persistent. Pedicel 0.5–1 mm long. Flowers 5 merous, calyx not calyptrate. Floral buds 3.75–4.25 × 3–3.5 mm, cupulate to campanulate, the hypanthium 3–4 × 2.5–3.5 mm, moderately to sparingly covered with spreading hairs 1–3 mm long and sparsely underlain with deciduous sessile stellate and stipitate-stellate hairs; calyx tube 0.5 mm long, calyx lobes hyaline, rounded-triangular, 0.25–1.5 mm long, calyx teeth linear to subulate, 2–4 mm long. Petals 4.5–5 × 1–2 mm, white or reportedly pink, oblong to oblong-ovate, reflexed, glabrous, truncate or rounded. Stamens 10, radially arranged around the style, the filament 2–2.5 mm long with a geniculation near the apex, yellow, anthers 1.5–2 × 0.5 mm, linear-oblong, yellow, the pore ca. 0.1 mm, terminal and slightly ventrally inclined. Ovary 5-locular, 2/3 inferior, apex elevated into a low ringlike collar with or without smooth hairs that surround the stylar scar. Style ca. 7 mm long, straight to slightly curving, vertical distance from the anther apex to the stigma ca. 1.5–2.5 mm, horizontal distance absent, stigma punctiform, ca. 0.25 wide. Berry 5 × 5 mm, purple black. Seeds ca. 0.5 mm long, more or less triangular in profile, angulate and somewhat muriculate to papillate on the convex face.

**Figure 135.**
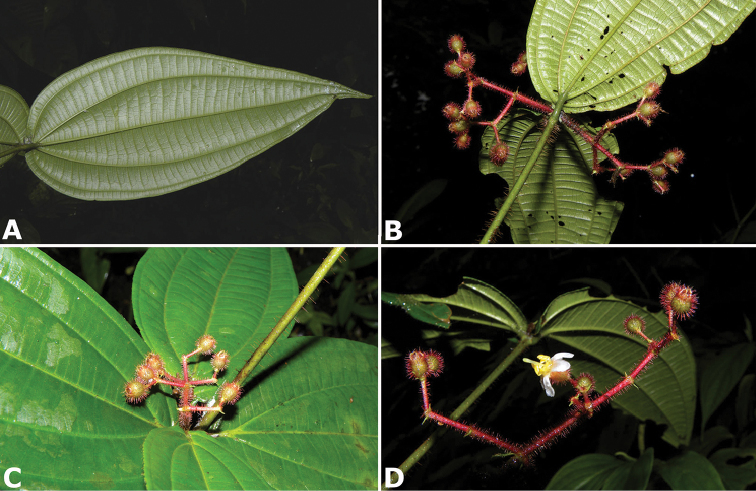
*Conostegia allenii*. **A** Leaf abaxial surface **B** Leaf base abaxial surface and infructescence **C** Leaf adaxial surface and infructescence **D** Close up of the flower. Photographs taken by Reinaldo Aguilar and vouchered *R. Aguilar 13243*.

#### Distribution

**Figure 136.**
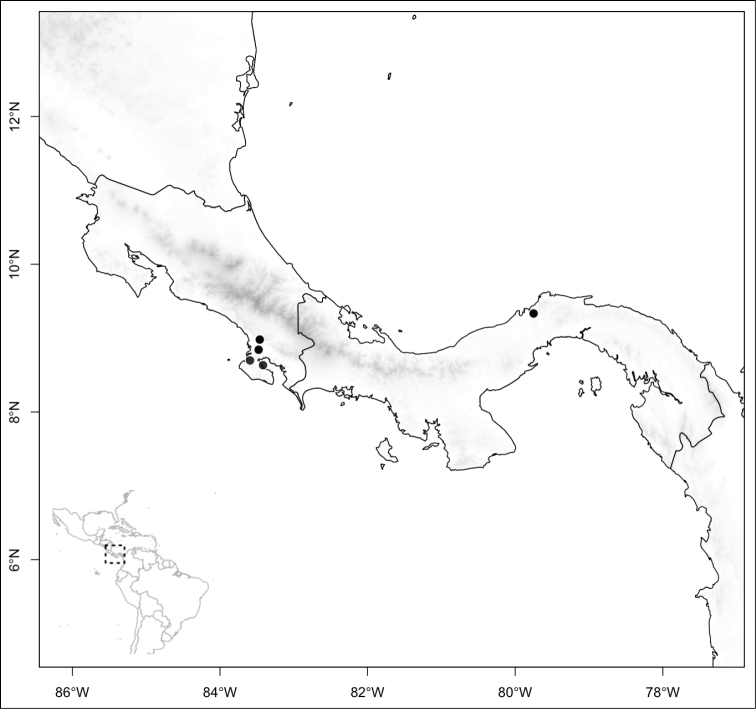
Distribution of *Conostegia allenii*.

(Fig. 136). Primary forest understory in the Golfo Dulce and Osa Peninsula region in southern Costa Rica, and a population in Panama on the Santa Rita Ridge, from sea level to 400 m elevation.

*Conostegia allenii* can be distinguished by its sessile to subsessile leaves that usually have oblique bases, pseudolateral inflorescences, and reddish hirsute indument on inflorescence branches and hypanthium. In the protologue Almeda (2004) mentioned the specimen *Quesada 591* (INB) from Rincón de Osa as somewhat unusual in having glabrous adaxial foliar surfaces. An additional specimen from this locality (*Aguilar 13243*-NY) also has glabrous leaves adaxially. The latter specimen was accompanied by photographs of the flowering plant (Fig. 135) which evidenced the white petals of the flowers. Other Costa Rican specimens reportedly have pink flowers, and the only other report of white petals in this species are the only known Panamanian specimen cited above. *Conostegia allenii* represents a strong case of vegetative convergence with taxa such as *Clidemia costaricensis* and *Clidemia petiolaris*. Almeda (2004) compared *Conostegia allenii* to both of these taxa in the protologue because of their similar indument and inflorescence.

#### Specimens examined.

**COSTA RICA. Puntarenas**: Rancho Quemado, camino a Drake, parte mas elevada del camino, Aguilar 13243 (NY); Sendero por el acueducto de Sierpe, Gonzalez 3586 (INB); Bosque primario a la par de la carretera Interamericana 3 km N de Chacarita, Nepokroeff and Hammel 722 (CAS, CR); Cantón de Osa, Rancho Quemado, Rincón, Quesada 591 (INB, MO).

**PANAMA. Colón**: Santa Rita ridge, km 21.2, de Nevers 7207 (CAS).

### Conostegia brenesiana

Taxon classificationPlantaeMyrtalesMelastomataceae

Kriebel nom. nov.

urn:lsid:ipni.org:names:77156245-1

Fig. 137

Conostegia brenesiana Kriebel. Based on: Miconia brenesii Standl, Field Mus. Nat. Hist, Bot. Ser. 18: 816. 1938. Type: Costa Rica. Alajuela: Santiago de San Ramón, January 1937, A. Brenes 21981 (holotype: F!, isotypes: CR!).

#### Description.

Small trees 1.5–10 m tall with rounded-quadrate stems that are moderately to sparsely ferrugineous scurfy or stellulate-puberulent, to almost glabrous; the nodal line present but inconspicuous. Leaves at a node equal to unequal in length. Petioles 0.4–2 cm. Leaf blades 4–14.4 × 1.2–4.7 cm, 3–5 plinerved, with the innermost pair of primary veins diverging from the midvein 0.4–2 cm above the base in opposite or generally alternate fashion, elliptic, the base acute to obtuse and typically asymmetrical, the apex attenuate to acuminate, the margin entire to distally crenulate, the adaxial surface glabrous, the abaxial syrface glabrous on the surface and scurfy puberulent to glabrous on the veins, frequently with white dots irregularly spread on the lamina. Inflorescence a terminal panicle 2.8–7.5 cm long branching at or above the base, the rachis glabrous to scurfy puberulent, the branches very thin, the bracteoles 0.5–1 mm, narrowly triangular to subulate, persistent and fused basallly forming a shallow inconspicuous nodal collar or elevated ridge. Pedicels 0.5–2 mm. Flowers 5 merous, not calyptrate, floral buds 1.5–3.75 × 0.9–2.0 mm, the hypanthuim 1.5–2 × 1.25–1.75 mm, scurfy puberulent, calyx tube ca. 0.1 mm long, the calyx lobes depressed undulate, 0.5 × 1 mm, the calyx teeth bluntly triangular, 0.25 mm long, equaling or barely exceeding the calyx lobes. Petals 1.5–2.5 × 1–1.5 mm, white, oblong, glabrous reflexed, emarginate. Stamens 10, 2–2.5 mm long, actinomorphic, the filaments 1.25–1.75 mm, with a geniculation near the middle, white, anthers 0.5–1 × 0.25–0.55 mm, cuneate in outline and widest at the apex, white or yellow, the connective prolonged briefly below thecae, the pore subterminal ca. 0.3 mm wide, dorsally inclined. Ovary 5 locular, inferior, glabrous or scurfy puberulent apically. Style 3.25–4 mm long, straight, vertical distance from stamens to stigma ca. 0.5–1 mm, horizontal distance absent, the stigma punctiform to truncate, ca. 0.35 wide. Berry 4.25–5.25 × 4.25–5.25 mm, purple black. Seeds 0.34–0.5 mm long, more or less pyramidal, frequently asymmetrical.

**Figure 137.**
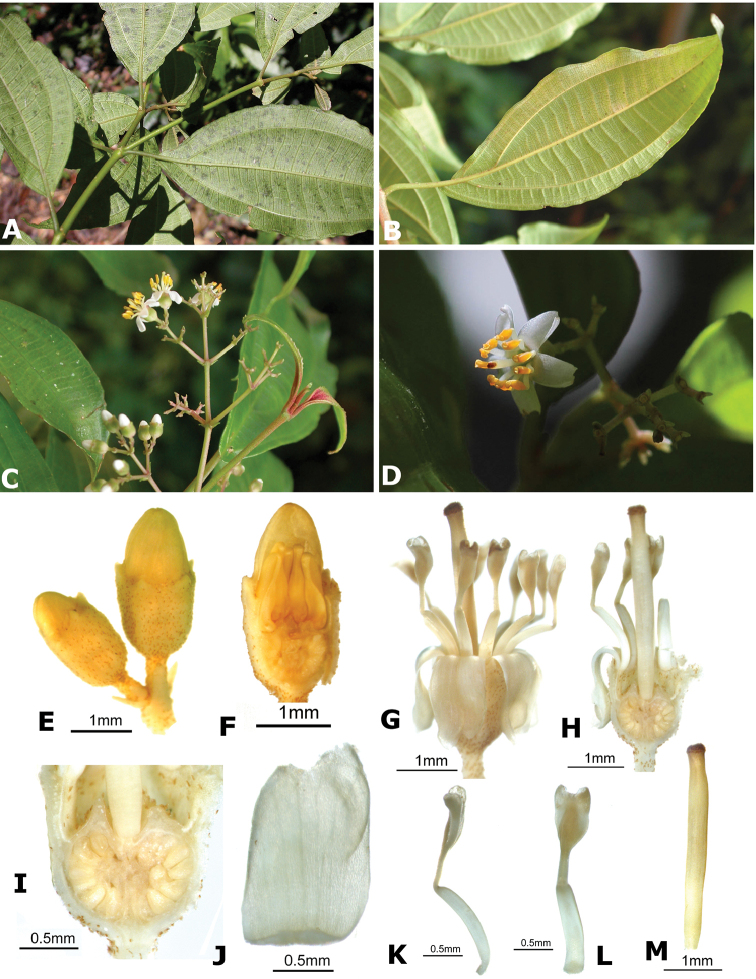
*Conostegia brenesiana*. **A** Branch showing plinerved leaf venation with slightly asymmetrical venation **B** Leaf abaxial surface showing strongly asymmetrical leaf venation **C** Inflorescence **D** Close up of the flower **E** Flower buds and subtending bracteoles **F** Longitudal section of a flower bud with the style removed **G** Pickled flower **H** Longitudal section of a flower **I** Close up of the ovary apex. Note brown glands in the inner hypanthium wall and ovary apex **J** Petal **K** Stamen side view **L** Stamen ventral view **M** Style. Photographs **A–D** taken by Kenji Nishida **E–M** from photographs of specimen vouchered *R. Kriebel 3665*.

#### Distribution

**Figure 138.**
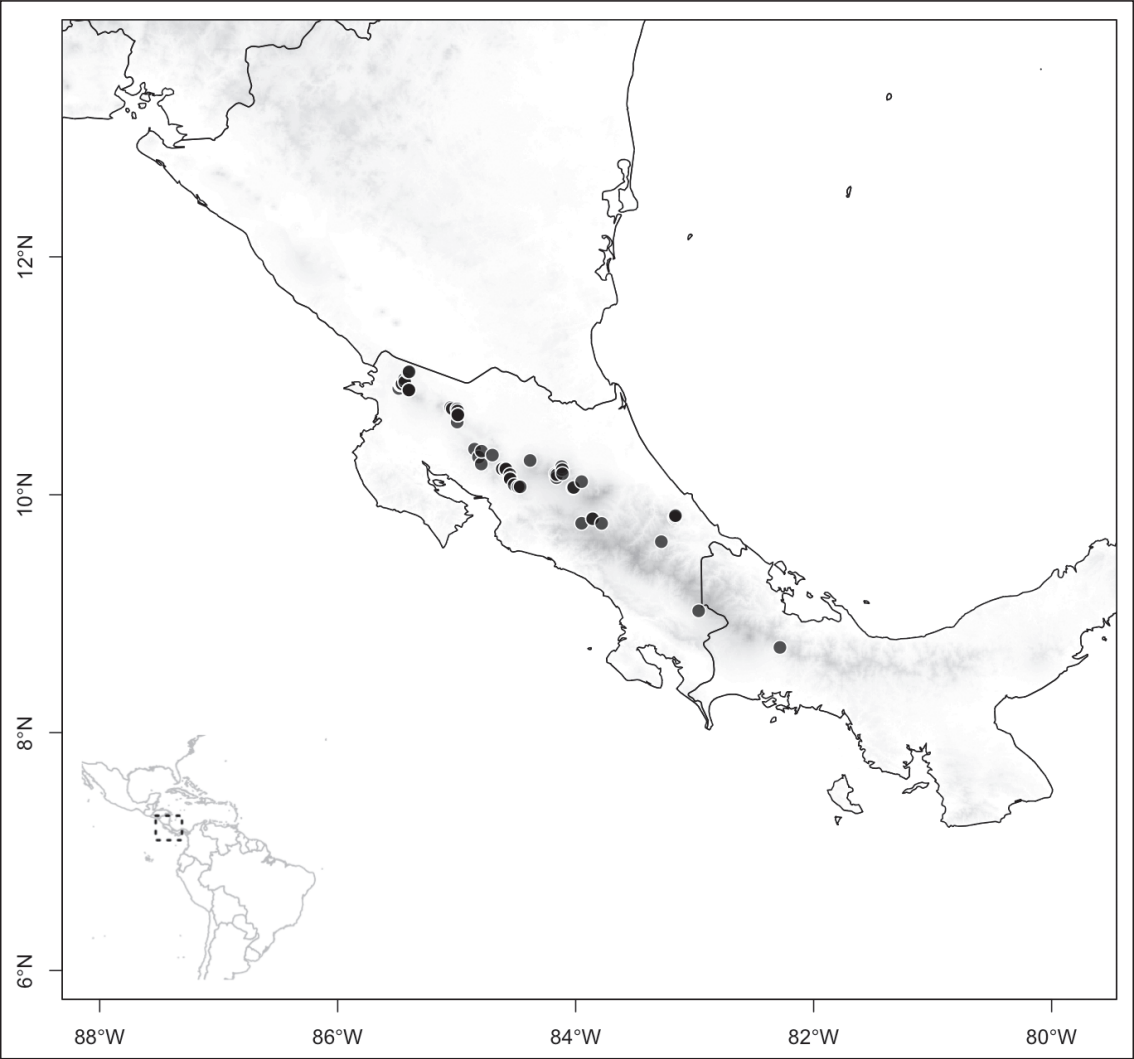
Distribution of *Conostegia brenesiana*.

(Fig. 138). Endemic to cloud forests in Costa Rica from 550–2100 m elevation.

The epithet *brenesiana* was chosen for this species because *brenesii* is preempted by *Conostegia brenesii* Standl. *Conostegia brenesiana* can be recognized because of its almost glabrous vegetative parts, plinerved leaves frequently with asymmetric venation, wiry inflorescences with persistent bracteoles and small five merous flowers. In addition, this species has the widest anther pores in *Conostegia* and some of the smallest anthers. Almeda (2009) reports the ovary of this species as glabrous. The specimen I studied from spirit had small glands suggesting intraspecific variation. Further observations should be made in this species considering it is also variable in anther color. Some specimens report the flowers as totally white while others report the anthers as yellow. An interesting aspect of this variation concerns the possible transition of pollination systems in this species. *Conostegia brenesiana* inhabits cloud forests mainly at middle elevation but spanning a broad range. An interesting hypothesis to test is if the more white flowers occur at higher elevations where the buzz pollinated species tend to decline. The species already presents the broad anther pores which in themselves hint to a transition that might be happening. One specimen from Cerro Hornito in western Panama (*Kriebel and Burke 5752*) might represent this species but the inflorescence is too immature to tell.

#### Specimens examined.

**COSTA RICA. Alajuela**: La Palma de San Ramón, Brenes 5232, 6768 (CR, NY); Los Angeles (Silencia) de San Ramon, Brenes 17081 (CR, NY); San Pedro near San Ramon, Brenes 5052 (NY); Cerro de San Rafael de San Ramon, Brenes 22414 (CR, NY); Reserva forestal San Ramón, slope above and in the valley of the Río San Lorencito, Burger et al. 12130 (CR, MO, NY); Guatuso, P.N. Volcán Tenorio, Cuenca del Río Frío, Alto Masis, Chaves and Muñoz 361 (CR, INB, MO, NY); Estación Río San Lorenzo, Kriebel 931 (INB); Finca La Paz, San Ramón, Kriebel 1477 (INB, NY); Alfaro Ruiz, P. N. Juan Castro Blanco, Cuenca alta del Río La Vieja, Solano and Cerling 2696 (INB, NY). **Cartago**: Kiri Lodge, Sendero el Colibrí, Kriebel 3665 (INB, MO, NY). **Guanacaste**: Estación Biológica Cacao, Trail to Cacao summit aprox. 1 km from station alongside trail, Boyle 7203 (INB, NY); P.N. Guanacaste, Sector Cacao, Kriebel et al. 879 (INB); Estación Pitilla, sobre la fila al Cerro Orosilito, Robles et al. 2815 (INB, MO). **Heredia**: Santa Bárbara, Posada Volcán Poás, Kriebel 1255 (INB, NY); Vara Blanca de Sarapiquí north slope of Central Cordillera between Poás and Barba volcanoes, Skutch 3455 (CR, NY); 9 km NO de Varablanca hacia finca Murillo, Proyecto ALAS, Solano, González and Santamaría 2134 (INB, NY). **Puntarenas**: Cordillera de Tilarán, San Luis, Río Veracruz, Monteverde, Fuentes 690 (CR, MO). **San José**: San Jerónimo de Moravia, 2 km NE del peaje, en el límite con el P. N. Braulio Carrillo, Kriebel 1113 (INB) forest near Río Hondura, Lent 1352 (CR, MO, NY); P. N. Braulio Carrillo, Faldas del cerro junto al Río Zurquí, Sector Santa Elena, Vargas and Castillo 3473 (INB, NY).

### Conostegia calocoma

Taxon classificationPlantaeMyrtalesMelastomataceae

(Almeda) Kriebel comb. nov.

urn:lsid:ipni.org:names:77156246-1

Fig. 139

Conostegia calocoma (Almeda) Kriebel. Basionym: Miconia calocoma Almeda, Proc. Calif. Acad. Sci. 46(5): 144. 1989. Type: Costa Rica. Heredia: Finca La Selva, OTS Field Station on Río Puerto Viejo just E of its junction with Río Sarapiquí, SE corner, elev. 100 m, 17 April 1981, J. Folsom 9776 (holotype: CAS!, isotype: DUKE).

#### Description.

Shrub to small tree 2.5–5 m tall with terete stems which are covered with a rusty mixture of stellate and stipitate stellate trichomes; the nodal line covered by indument. Petiole 0.4–1.4 cm. Leaf blade 4.5–19.5 × 2.5–9 cm, 5(-7) plinerved, with the inner pair of primary nerves diverging from the mid vein 0.4–1.5 cm above the blade base in opposite, sub opposite or alternate fashion, elliptic to elliptic ovate, the base typically obtuse to rounded but varying to asymmetrical and briefly decurrent, the apex acuminate, the margin undulate to undulate-dentate, the adaxial surface sparingly stellate to glabrous, abaxially copiously stellate on the elevated primary veins with a sparser cover on the transverse secondary and higher order veins. Inflorescence a terminal, erect or deflexed panicle 2–8 cm long, branched above the base, with flowers in terminal congested glomerules, accessory branches absent; bracteoles 0.5–1.5 × 0.25–5 mm, linear-oblong, persistent. Pedicel 0.5 mm long. Flowers 4-merous, not calyptrate but flower bud closed and crowned by an apiculum ca. 0.25 mm long that ruptures at anthesis into 2 to 4 deltoid and hyaline lobes mostly 1 × 1–1.5 m the calyx teeth linear-oblong, 1 mm long, the hypanthium 2–3.75 × 2–2.5 mm, densely stellate pubescent. Petals 3–4 × 1.5–2.5 mm, white, obovate to oblong-obovate, apparently reflexed at anthesis, glabrous, rounded to emarginated apically. Stamens 8, 3–3.5 mm, actinomorphic, the filaments 1.5–2 mm, with a geniculation near the apex, white, anthers 1–1.5 × 0.5–0.75 mm, linear-oblong, laterally compressed, the connective not prolonged nor appendaged, yellow, the pore ca. 0.17 mm, omewhat ventrally inclined. Ovary 4-locular, 3/4 inferior, the apex glabrous and not forming a collar around the style base. Style 5–7 mm long, exserted and straight to slightly curving towards the apex, vertical distance from the anthers to the stigma ca. 1.5–2 mm, horizontal distance absent; stigma punctiform, 0.25–0.5 mm wide. Berry 6–8 × 6–8 mm, purple black. Seeds ca. 1 mm long, obovoid to pyriform, angulate and with a densely tuberculate testa.

**Figure 139.**
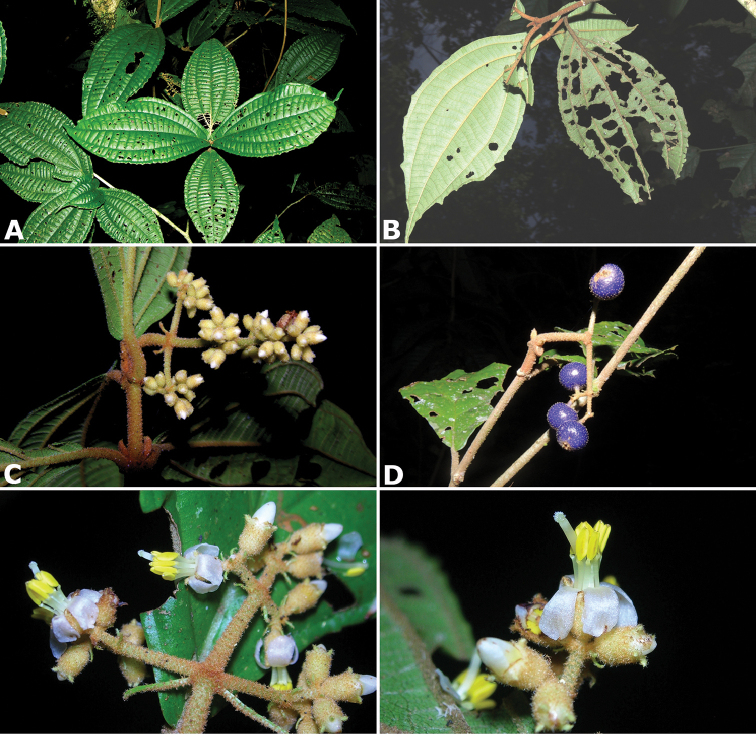
*Conostegia calocoma*. **A** Branch of immature plant **B** Abaxial surface of a new leaf and older leaf. Note herbivory on older leaf **C** Inflorescence with flower buds **D** Infructescence. Photos **A, C** of specimen vouchered *R. Kriebel 5484*, and **B, D** from spirit material at NY vouchered *R. Kriebel s.n*. Photos **E, F** by Enrique Salicetti.

#### Distribution

**Figure 140.**
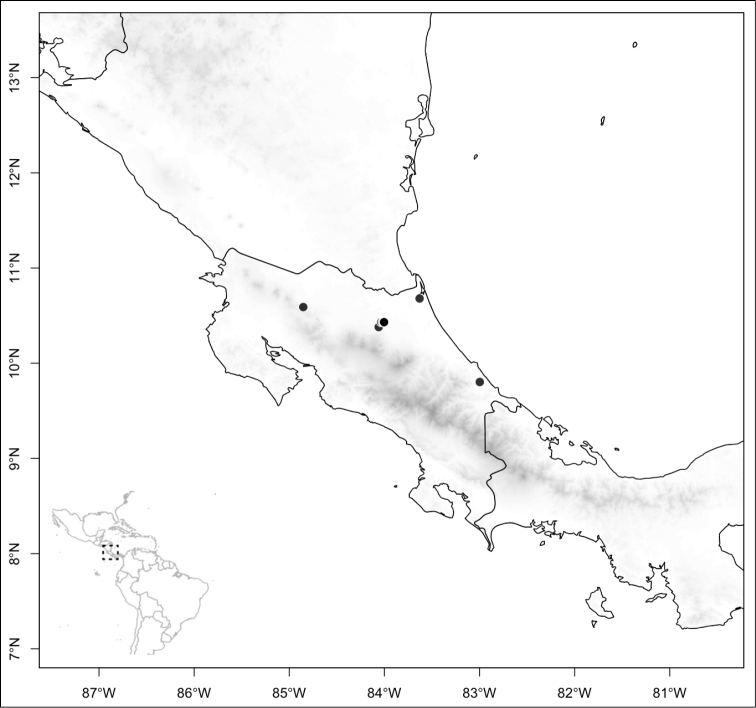
Distribution of *Conostegia calocoma*.

(Fig. 140). Endemic to Caribbean lowlands and foothills of Costa Rica, from sea level to 600 m in elevation. To be expected in the south eastern lowlands of Nicaragua.

*Conostegia calocoma* can be recognized on the basis of its rusty stellate pubescence, short petiolate leaves with plinerved venation and undulate margin, fused floral buds in which the calyx ruptures irregularly, 4 merous flowers, and bright yellow anthers. The molecular phylogenetic study revealed strong support for a clade comprised of *Conostegia calocoma* as sister to the species pair *Conostegia colliculosa* and *Conostegia subpeltata*. A high quality line drawing was provided in the protologue of this species (Almeda 1989a).

#### Specimens examined.

**COSTA RICA. Alajuela**: Guatuso, La Cabanga (Sector Cabanga), Finca de José Martínez (parche grande después de cruzar dos quebradas), Kriebel et al. 5484 (INB, NY). **Heredia**: Estación Biológica La Selva, camino al lindero sur, Kriebel 4006 (INB, NY). **Limón**: en Playón sombreado y con pedrones del Río Lari, La Quebrada Alto Lari, Jiménez 1910 (CR, NY); San Rafael de Pandora, Rodríguez 861 (CAS, INB); Cerro Coronel, E of Laguna Danto, Stevens 23730 (CAS, MO).

### Conostegia centrosperma

Taxon classificationPlantaeMyrtalesMelastomataceae

(Almeda) Kriebel comb. nov.

urn:lsid:ipni.org:names:77156247-1

Fig. 141

Conostegia centrosperma (Almeda) Kriebel. Basionym: Miconia centrosperma Almeda, Brittonia 35(1): 42, f. 1. 1983. Type: Panama. Panamá: along newly cut road from El Llano to Carti-Tupile, 3 mi above Pan-Am Highway, elev. 200 m, 13 March 1973, R. L. Liesner 702 (holotype: CAS!; isotypes: MO!, US).

#### Description.

Small trees 3–7 m tall with flattened and two edged stems when young that become terete with age and which are reddish brown and densely stellulate-lepidote on young branches; the nodal line nodal line evident. Leaves at a node equal to subequal in length. Petiole 0.5–2 cm. Leaf blades 5.25–12.5 × 1.5–4.5 cm, 3-plinerved, with the inner pair of primary nerves diverging from the mid vein 0.4–3 cm above the blade base in opposite, subopposite or alternate fashion, elliptic to elliptic lanceolate, the base acute to decurrent on the petiole, the apex acuminate, the margin entire or slightly denticulate, the adaxial surface glabrous, the abaxial surface reddish brown and densely stellulate-lepidote. Inflorescence a terminal little branched panicle 1.6–4.8 cm long with the branches terminating in three sessile-flowered clusters, accessory branches mostly absent, bracteoles 3–4 mm long, linear-oblong, early deciduous. Floral buds ca. 4–5 × 2–3 mm, campanulate to urceolate. Flowers 5-merous, not calyptrate, but with the calyx lobes fused in bud and rupturing at anthesis (with the style sticking out initially) into irregular persistent lobes ca. 2 mm long, the calyx teeth obsolete or evident as blunt protuberances at or near the torus, the hypanthium 2–3 × 2–3 mm, densely reddish brown and densely stellulate-lepidote. Petals 3.75–4.25 × 2–2.5 mm, white, oblong-obovate, spreading, glabrous, truncate to asymmetrical apically. Stamens 10, 4.5–5.25 mm long, radially arranged around the style, the filament 2.5–3.25 mm, with a geniculation near the apex, white, anthers 1.5–2 × 0.5–0.75 mm, linear oblong, yellow, laterally compressed, the pore ca. 0.2 mm, terminal. Ovary 5-locular, inferior, costate apically and forming a setose collar around the style. Style 6–6.5 mm, exserted and straight, vertical distance from the anther to the stigma ca. 1.75–2 mm from the stigma, horizontal distance absent, the stigma truncate to slightly expanded, 0.65–0.85 mm wide. Berry 3–4 × 3–4 mm, purple black. Seeds ca 0.5 mm, cuneate, angularly ridged with a conspicuous spur on the distal truncate surface.

**Figure 141.**
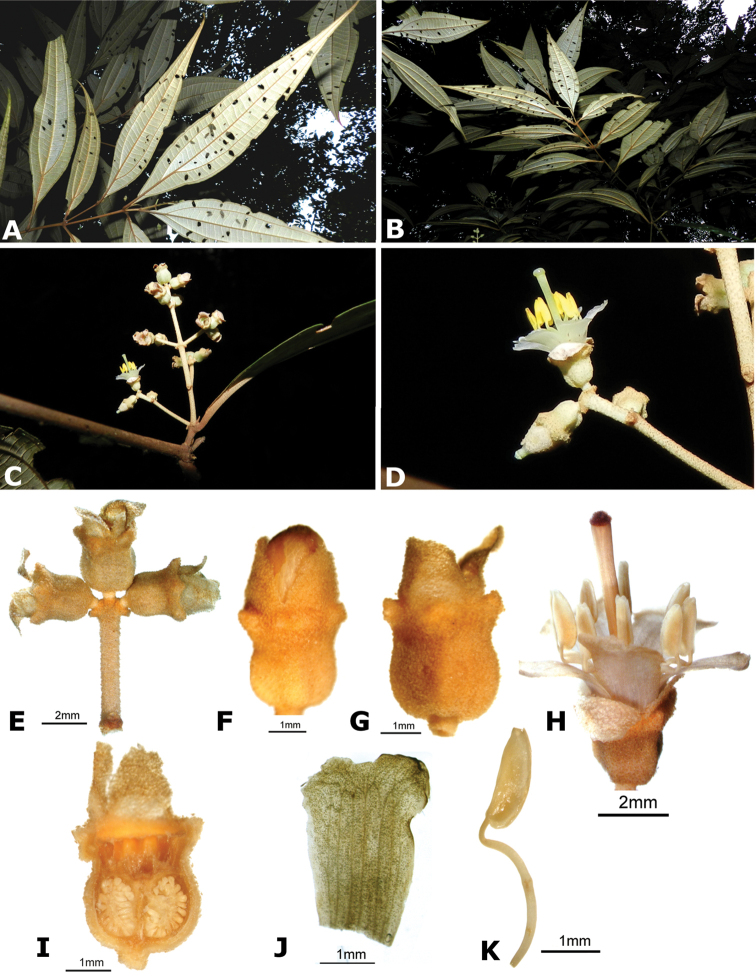
*Conostegia centrosperma* **A–B** Infertile branches showing whitish abaxial indument **C** Inflorescence **D** Close up of flower bud and flower **E** Inflorescence branch **F** Flower bud opening **G** Hypanthium showing irregularly rupturing calyx **H** Pickled flower at anthesis **I** Longitudinal section of flower at anthesis with parts removed showing rim around stylar scar and irregularly rupturing calyx lobes from the inside **J** Petal **K** Stamen. Photos of specimen vouchered *R. Kriebel 5690*.

#### Distribution

**Figure 142.**
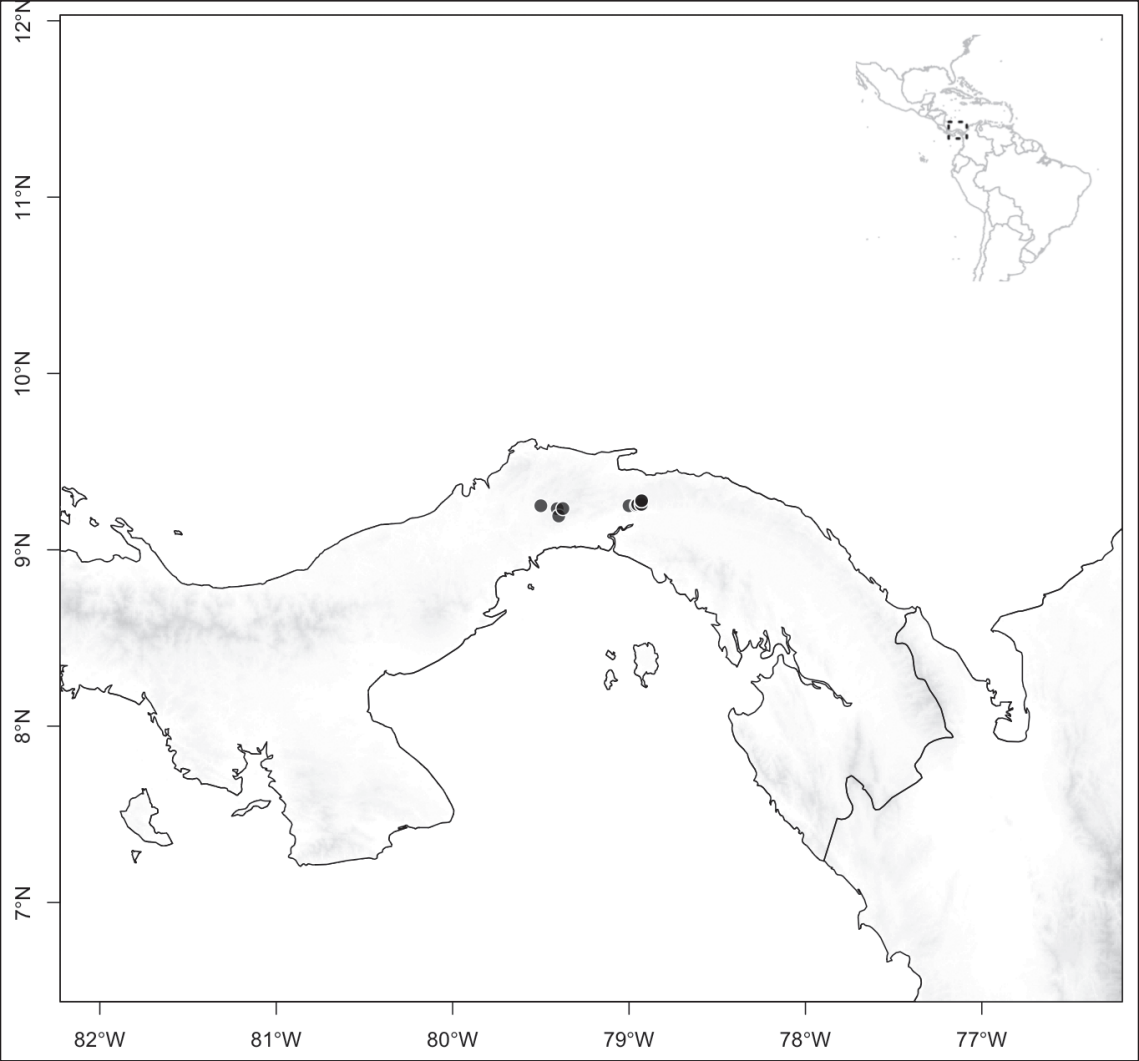
Distribution of *Conostegia centrosperma*.

(Fig. 142). Endemic to central Panama where it has been collected in the foothills of Cerro Jefe and El Llano Cartí Road, 200–750 m elevation.

Almeda (1983) compared *Conostegia centrosperma* to *Conostegia xalapensis* in the protologue, a species from which *Conostegia centrosperma* differs by its non-calyptrate calyx, more lepidote than stellate indument, exserted style, and setose rim around the style base. Almeda (1983) also compared *Conostegia centrosperma* to *Conostegia fulvostellata* and *Conostegia oligocephala* which interestingly also belong to section *Geniculatae*. These two species differ from *Conostegia centrosperma* in their non rupturing calyx and stellate indument. When Almeda described *Conostegia centrosperma*, *Conostegia dissitinervia* had not been described. When *Conostegia dissitinervia* was described (Kriebel et al. 2005) its possible relationship to *Conostegia centrosperma* was discussed and both taxa were separated in a dichotomous key. These two species form a strongly supported sister pair in the molecular phylogenetic analyses. They can be distinguished by the larger leaves with stellate indument abaxially and longer inflorescences in *Conostegia dissitinervia*. In addition to the hyaline calyx of the latter when rupturing, does not leave the evident calyx lobe pieces of *Conostegia centrosperma*. Lastly, *Conostegia dissitinervia* lacks the setae around the style base present in *Conostegia centrosperma* and has an apiculate anther apex that *Conostegia centrosperma* lacks.

#### Specimens examined.

**PANAMA. Panamá**: P.N. Chagres, sendero El Mono, adentro de la urbanización Altos de Cerro Azul, Kriebel and Burke 5690 (NY, PMA); vicinity of Cerro Jefe, McPherson 10004 (CAS); El Llano Cartí Road, 8.5 km from Inter-American Hwy, Mori et al. 4546, 5142 (CAS).

### Conostegia cinnamomea

Taxon classificationPlantaeMyrtalesMelastomataceae

(Beurl.) Wurdack

Fig. 143

Conostegia cinnamomea (Beurl.) Wurdack, Phytologia 38: 287. 1978. Miconia cinnamomea Beurl., Svensk. Vet. Handl. 1854: 131. 1854. Type: Panama. Portobello, April 1826, J. Billberg 271 (holotype: S!). Oxymeris cinnamomea (Beurl.) Triana, Trans. Linn. Soc. Bot. 28: 94. 1872. Leandra cinnamomea (Beurl.) Cogn. Mart. Fl. Bras. 14(4): 77. 1886.
Conostegia micromeris Standl., Contr. Arnold Arb. 5: 117, pl. 15. 1933. Type: Panama. shore of cove west of Drayton House, Barro Colorado Island, Canal Zone, 6 February 1932, R. Woodworth and P. Vestal 602 (holotype: F!, isotypes: A!, LE, MO!).
Conostegia haughtii Gleason, Phytologia 2: 429. 1948. Type: Colombia. Antioquia: on Quebrada Isaias, east of Turbo, 5 July 1946, O. Haught 4939 (holotype: NY!, isotypes: F, K!, P[fide Almeda], S!, US!).

#### Description.

Shrubs to small tress 1.5–6 m tall with terete, sometimes slightly rectangular stems especially towards the apex which are moderately to densely covered on new growth with short dendritic hairs; the nodal line present yet slight. Leaves at a node equal to unequal in length. Petioles absent or to 1.3 cm long. Leaves 4.5–20.1 × 1.9–6.9 cm long, 5-plinerved, with the innermost pair of veins arising up to 4 cm above the blade base in opposite or more commonly strongly alternate fashion, elliptic, acute to cuneate, attenuate to acuminate, margin entire to crenate, glabrous adaxially, abaxially glabrous or with a few scattered stellate furfuraceous trichomes on the main veins. Inflorescence a terminal reflexed and pseudoaxillary panicle 2.6–7.6 cm long branching at the base, accessory branches absent, bracteoles lanceolate, to about 2 mm, persistent. Pedicel 0.5–1 mm. Flowers (4-)5 merous, calyptrate. Flower buds 2.3–4 × 1.1–1.5 mm, elliptic to slightly pyriform, rounded at the base, apiculate at the apex, calyx teeth present but minute and not discernable to the naked eye, the calycine and hypanthium differentiated with the calyptra tending to dry white, slightly to not constricted below the calyptra; the hypanthium 2–2.5 × 2–2.5 mm, glabrescent. Petals 3–4.25 × 1.75–2.5 mm, white, turning pink and closing with age, narrowly ovate, reflexed, glabrous, apically acute. Stamens (8-)10, 3.25–4.25 mm long, radially arranged around the style, the filament 1.75–2.25 mm, with a geniculation near the apex, white, anthers 1.5–2 × 0.5–0.75 mm, oblong, yellow, ventrally wrinkled, the pore 0.18–0.2 mm, dorsally inclined. Ovary 5 locular, inferior, apically glabrous and not forming a collar around the style base. Style 5.75–6.25 mm, straight to gently curving, vertical distance from the anther apex to the stigma 1.5–2 mm, horizontal distance absent, stigma punctiform, 0.35- 0.45 mm wide. Berry 4–5 × 4–5 mm, blue-black. Seeds 0.4–0.5(-0.8) mm, roughly pyramidal, the testa tuberculate.

**Figure 143.**
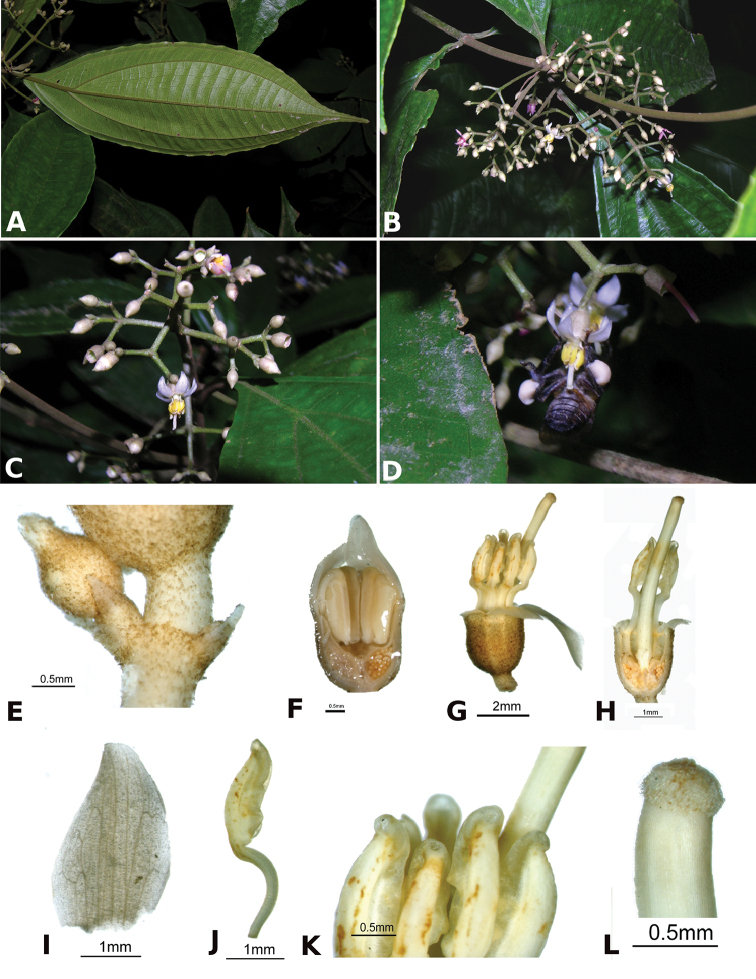
*Conostegia cinnamomea*. **A** Leaf abaxial surface **B** Inflorescence **C** Close up of flower **D** Close up of flower being grasped and about to be buzzed by a Melipona bee **E** Detail of flower bud subtended by bracteoles **F** Longitudinal section of a flower bud with the calyptra ad style removed **G** Pickled flower with some petals removed **H** Longitudinal section of a pickled flower **I** Petal **J** Stamen **K** Close up of the stamens showing dorsally inclined pore **L** Close up of the stigma. Photos of specimen vouchered *R. Kriebel 5330*.

#### Distribution

**Figure 144.**
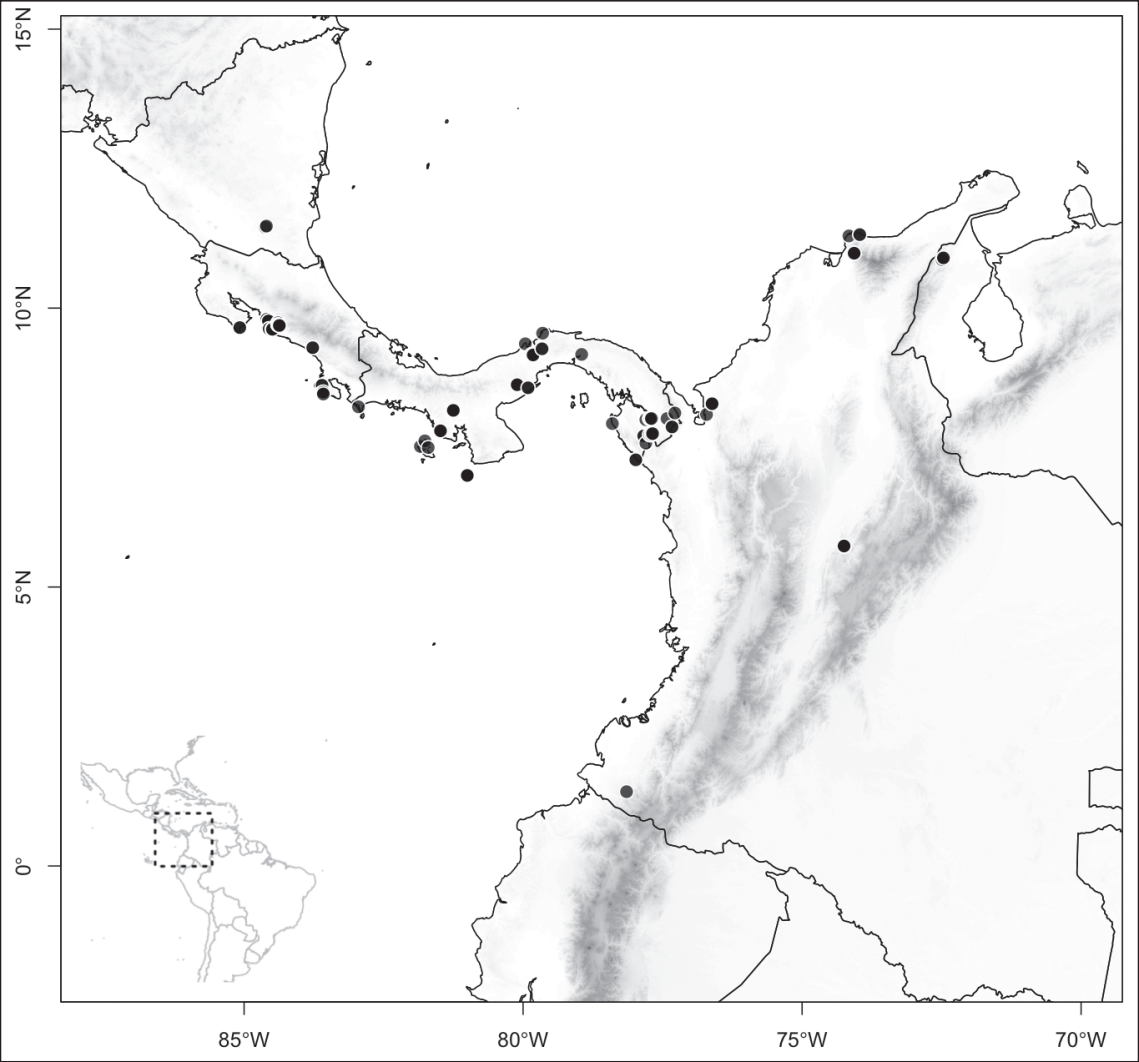
Distribution of *Conostegia cinnamomea*.

(Fig. 144). Nicaragua, Costa Rica, Panama, Colombia and northern Venezuela, from sea level to 1450 m elevation.

*Conostegia cinnamomea* is easily recognized its glabrous appearance, evidently plinerved leaves, deflexed inflorescences and calyptrate calyx. Its bracteoles are also helpful when flowers are lacking because they are persistent and form a nodal collar around the inflorescence branches. The style of this species is exserted and one of the bees observed to visit its flowers, *Melipona costaricensis*, curls its abdomen over the style before buzzing the flowers (Fig. 143). This same behaviour was also observed by another small halictid bee in a species with similar floral morphology, *Conostegia grayumii*. It is possible most of the species in section *Geniculatae* are buzzed in a similar way since they have floral morphology very similar to the above mentioned taxa. The anther pore in this species is perhaps the most strongly dorsally inclined in any species of the genus.

#### Specimens examined.

**NICARAGUA** (fide Schnell). **Zelaya**: between Toro Bayo and Esquipulas, drainage of the rivers Jícaro and Esquipulas, Shank and Molina 4615 (GH).

**COSTA RICA. Puntarenas**: Golfito, P. N. Corcovado, Estación Sirena, Sendero Espaveles, Aguilar 4980 (INB, MO). **San José**: Puriscal, La Cangreja, alrededores senderos de finca de Fundación Ecotrópica, Kriebel et al. 4283 (INB, NY); Puriscal, camino entre Puriscal y P. N. La Cangreja, Kriebel 5330 (INB, NY).

**PANAMA. Canal Zone**: Barro Colorado Island, Barbour Lathrop Trail 250, Croat 6542 (MO, NY). Coclé: cloud forest on slopes of Cerro Pilon near El Valle, Duke 12170 (NY). **Darién**: between Paya and Palo de los Letras, Duke and Kirkbride 14022 (NY); Lower slopes of alturas de Nique along Río Coasi, Hartman 12257 (MO, NY); Mannene to the mouth of Río Coasi, Kirkbride and Bristan 1560 (NY); 0–2 mi. E. of Tres Bocas along shortest headwater of Río Coasi, Kirkbride and Duke 1201 (MO, NY).

**COLOMBIA. Magdalena**: in forest along trail from Pueblito to Playa Brava, Parque Nacional Tayrona, Kirkbride 2585 (NY); Alto Río Frío Cabeceras del Río Congo, Ciudad Antigua, Madriñán and Barbosa 486 (NY).

**VENEZUELA. Zulia**: Dtto. Mara, cuenca del río Guasare, alrededores del Destacamento Guasare 1 (La Yolanda) en las laderas del cerro ca. 5 km al SSE del Destacamento entre el Caño Indio y la fila arriba de su orilla izquierda, Bunting et al. 12711 (NY); Distrito Mara, semi-evergreen riverine forest along dry creek bed of Caño Indio between Hacienda Caño Azul and base of Cerro Yolanda 15 km southwest of rancho 505 south of and tributary of Rio Guazare, Steyermark et al. 122652 (MO, NY).

### Conostegia colliculosa

Taxon classificationPlantaeMyrtalesMelastomataceae

(Almeda) Kriebel comb. nov.

urn:lsid:ipni.org:names:77156248-1

Fig. 145

Conostegia colliculosa (Almeda) Kriebel. Basionym: Miconia colliculosa Almeda, Proc. Calif. Acad. Sci, ser. 4, Series 4, 52(4): 33, f. 1. 2000. Type: Costa Rica. Limón: Cantón de Talamanca Amubri. Camino entre Amubri y Soki, Siguiendo el Río Nabri hacia Alto Soki, 929'50"N, 8259'10"W, elev. 150 m, 3 July 1989, G. Herrera 3129 (holotype: CAS!, isotypes: CR!, INB!, MEXU, MO!).

#### Description.

Shrub to small trees 1.5–6 m tall with terete branches that are apically densely covered with brown penicillate-stellate hairs, older branches glabrous; the nodal line present yet slight and covered by indument. Leaves at a node equal to subequal in length. Petiole 0.4–1.5 cm. Leaf blades 9.5–20.5 × 4.2–10.6 cm, 5(-7) plinerved, with the inner pairs of primary nerves diverging from the mid vein up to 2 cm above the base in sub opposite or alternate fashion, elliptic-ovate, the base obtuse to rounded and frequently oblique, slightly decurrent on the petiole, the apex acuminate, the margin undulate-denticulate to almost entire, the adaxial surface glabrous at maturity, the abaxial surface with penicillate stellate hairs on the elevated primary veins and stellate hairs on the secondary and higher order veins. Inflorescence a terminal panicle with ultimate branches termi-nating in simple dichasia appearing pseudolateral from elongation of lateral branches, sometimes deflexed, 3.5–8 cm long, branching above the base, accessory branches absent, densely covered with brown penicillate-stellate and /or coarse dendritic hairs, bracteoles 1–4 mm × 0.25–1 mm, linear-oblong, persistent. Pedicel lacking or to 0.25 mm long. Flowers 5-merous, calyx not calyptrate. Flower buds not seen; the hypanthium 2–2.5 × 1.75–2.25 mm, densely covered with brown penicillate-stellate and/or coarse dendritic hairs; the calyx tube 0.5 mm long, the calyx lobes ovate to suborbicular, often mucronate at the apex, stellate pubescent on both surfaces, the calyx teeth subulate, 1.5–2 mm long. Petals 3–4 × 1.5–2 mm, white, oblong-obovate, petal posture uncertain, the apex mostly rounded, glabrous. Stamens 10, ca. 2.5–3.5 mm long, radially arranged at anthesis, the filament 1–2 mm, with a geniculation near the apex, white, anthers 1–1.5 × 0.5–0.75 mm, elliptic, yellow, the pore 0.1 mm wide, dorsally inclined. Ovary 5-locular, inferior, costate apically and with a lobed glandular puberulent crown around the style base. Style 3.15–3.45 mm, exserted and straight to slightly curving, vertical distance from the anther to the stigma 1.45–1.65 mm, horizontal distance absent, stigma punctiform, 0.25–04 mm wide. Berry 4–5 × 4–5 mm, not seen at maturity. Seeds 0.5 mm long, triangular in outline, testa colliculose.

**Figure 145.**
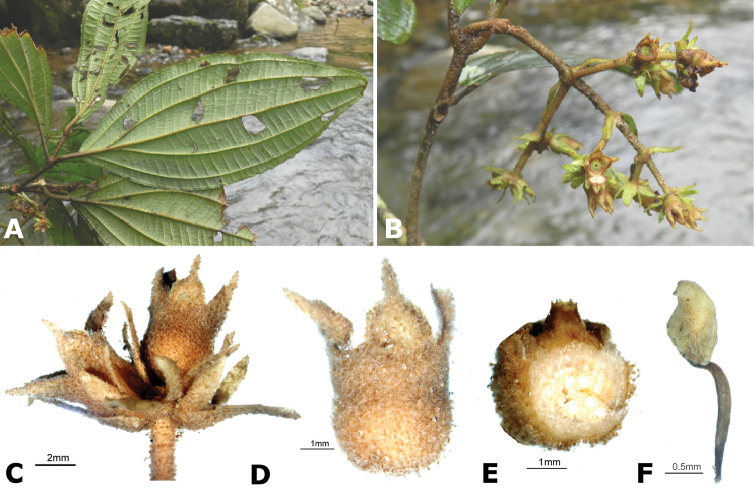
*Conostegia colliculosa*. **A** Leaf abaxial surface **B** Infructescence **C** Detail of the apex of an inflorescence branch showing bracts and bracteoles subtending sessile flowers **D** External view of the hypanthium **E** Apex of the ovary **F** Dried stamen. Photos of specimen vouchered *R Kriebel and J. Burke 5751*.

#### Distribution

**Figure 146.**
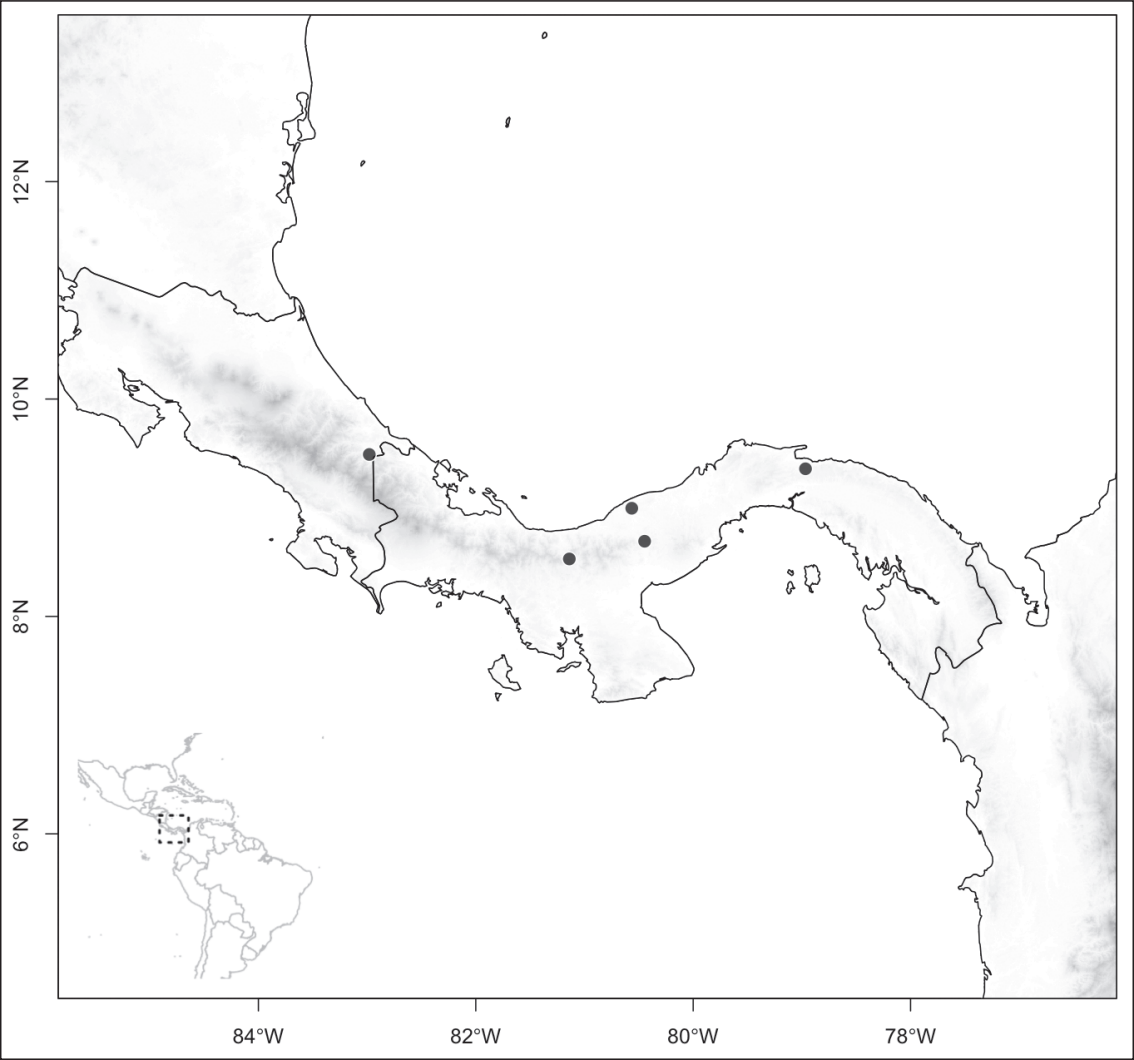
Distribution of *Conostegia colliculosa*.

(Fig. 146). Restricted to the Caribbean slope of Cost Rica and Panama, 150–650 m in elevation.

This rare species was assigned to Miconia section Ambyarrhena by Almeda (2000). The drawing in the protologue and study of the type specimen confirmed that the style is exserted and that the anthers are yellow, like most of its relatives, including *Conostegia calocoma* and *Conostegia subpeltata*. With the latter two species it also shares an irregularly rupturing calyx but in the case of *Conostegia colliculosa* the lobes are thicker and the calyx teeth longer. In addition, *Conostegia colliculosa* has five merous sessile flowers disposed in bracteate clusters whereas *Conostegia calocoma* and *Conostegia subpeltata* have four merous flowers that are not clustered in the inflorescences. *Conostegia supeltata* is further distinguished from *Conostegia colliculosa* in having subpeltate leaves and axillary inflorescences. Almeda (2000) suggested the relationship with *Conostegia calocoma*.

#### Specimens examined.

**PANAMA. Coclé**: El Santisimo, Wong 15–270 (CAS). **Colón**: Cedro Hueco, Mendieta 11–30 (CAS). SAN BLAS: headwaters of Río Nergala, along continental divide, de Nevers and Herrera 4514 (NY). **Veraguas**: P. N. Santa Fé, aprox. 3 a 6 km pasando la Escuela Agrícola Alto d e Piedra, Kriebel and Burke 5751 (NY, PMA).

### Conostegia consimilis

Taxon classificationPlantaeMyrtalesMelastomataceae

(Gleason) Kriebel comb. nov.

urn:lsid:ipni.org:names:77156249-1

Fig. 147

Conostegia consimilis (Gleason) Kriebel, comb. nov. Basionym: Leandra consimilis Gleason, Ann. Missouri Bot. Gard. 45(3): 268. 1958. Type: Panama. Panamá: Las Minas, 9 January 1941, P. Allen 2702 (holotype: NY!).
Miconia ligulata Almeda, Proc. Calif. Acad. Sci. 46(9): 216. 1989.

#### Description.

Small tree 1.5–6m tall with somewhat tetragonal and ridged stems in newer branches that are moderately to densely brown scurfy with dendritic or pinoid hairs; the nodal line present. Leaves at a node equal to unequal in length. Petiole 0.3–2 cm long. Leaf blade 4.2–28.4 × 1.4–9.2 cm, 5-plinerved, with the innermost pair of veins usually diverging from the midvein upt to 5 cm cm above the base in opposite to alternate fashion, elliptic, the base gradually tapering to decurrent on the petiole, the apex acuminate to long acuminate, margin entire to undulate, adaxial surface sparsely pulverulent to glabrous, the abaxial surface moderately and deciduously scurfy-pulverulent with short dendritic hairs evident mostly on the veins. Inflorescence a terminal panicle 4.4–11.2 cm long, branching above the base, accessory branches absent, brown scurfy on the branches; bracteoles 0.3–0.75 × 0.2–0.5 mm, subulate, persistent, fused and forming a nodal collar or ridge. Pedicel 0.5–2 mm. Flowers 5 merous, not calyptrate. Flower buds 3–3.75 × 1.3–1.6 mm; hypanthium 1.25–1.75 × 1.25–1.75 mm, campanulate, deciduously scurfy-pulverulent, calyx tube 0.2–0.3 mm long, calyx lobes depressed-triangular to undulate, 0.5 mm long but concealed and barely exceeded by the exterior subulate teeth, androecial fringe present and beset with glandular hairs. Petals 2.75–3.25 × 0.75–1 mm, translucent white to white with or usually without pinkish tinges towards the base, linear-oblong, spreading to reflexed at anthesis, glabrous the apex narrowly rounded to acute. Stamens 10, 2.25–2.75 mm, radially arranged around the style, the filaments 1.25–1.5 mm long, with a geniculation near the apex, white, anthers 1–1.25 0.25–0.4 mm, oblong, yellow, laterally compressed, the pore ca. 0.1 mm wide, somewhat dorsally inclined. Ovary 5 locular, 3/4 to 4/5 inferior, glabrous apically. Style 3.5–4.25 mm long, curving towards the apex, vertical distance from the anther pore to the stigma 1.5–2 mm, horizontal distance absent or up to 1 mm, stigma truncate, ca. 0.35 mm wide. Berry 3–4 × 3–4 mm, purple-black. Seeds 0.35–0.5 mm, pyramidate, smooth with verruculose angles.

**Figure 147.**
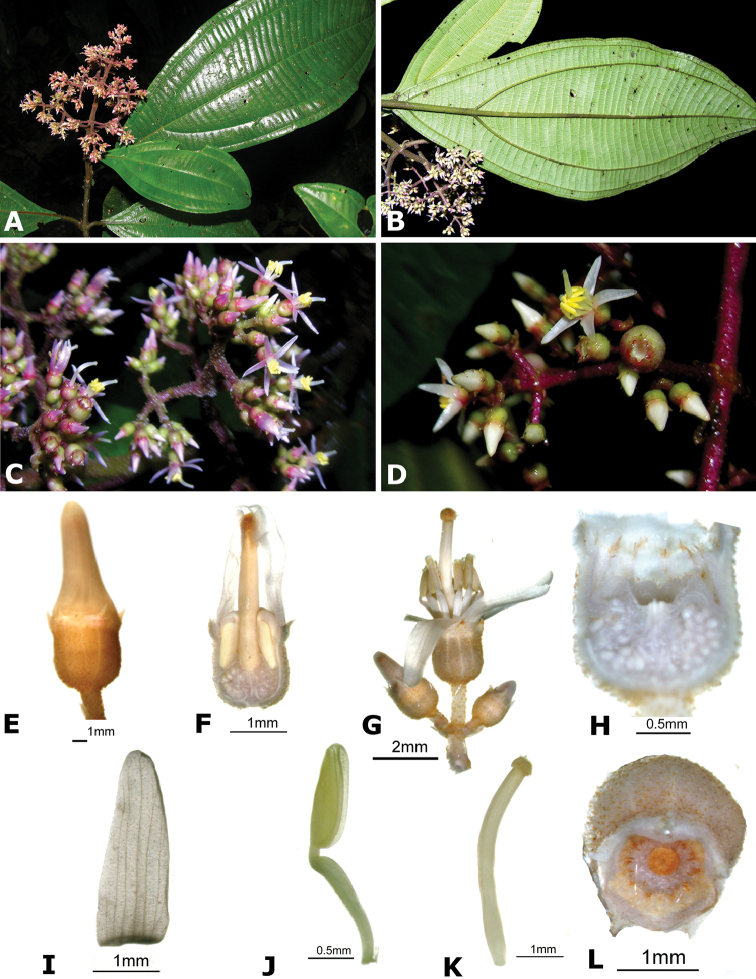
*Conostegia consimilis*. **A** Habit **B** Leaf abaxial surface **C** Inflorescence **D** Close up of flowers **E** Flower bud **F** Longitudinal section of a floral bud **G** Apex of inflorescence branch with flower at anthesis **H** Longitudinal section of the hypanthium. Note androecial fringe **I** Petal **J** Stamen **K** Style **L** View of a maturing fruit from above. Note androecial fringe. Photos **A–C** of specimen vouchered *R. Kriebel 5323* **D** from *R. Kriebel 5644*, and **E–L** from *R. Kriebel 5726*.

#### Distribution

**Figure 148.**
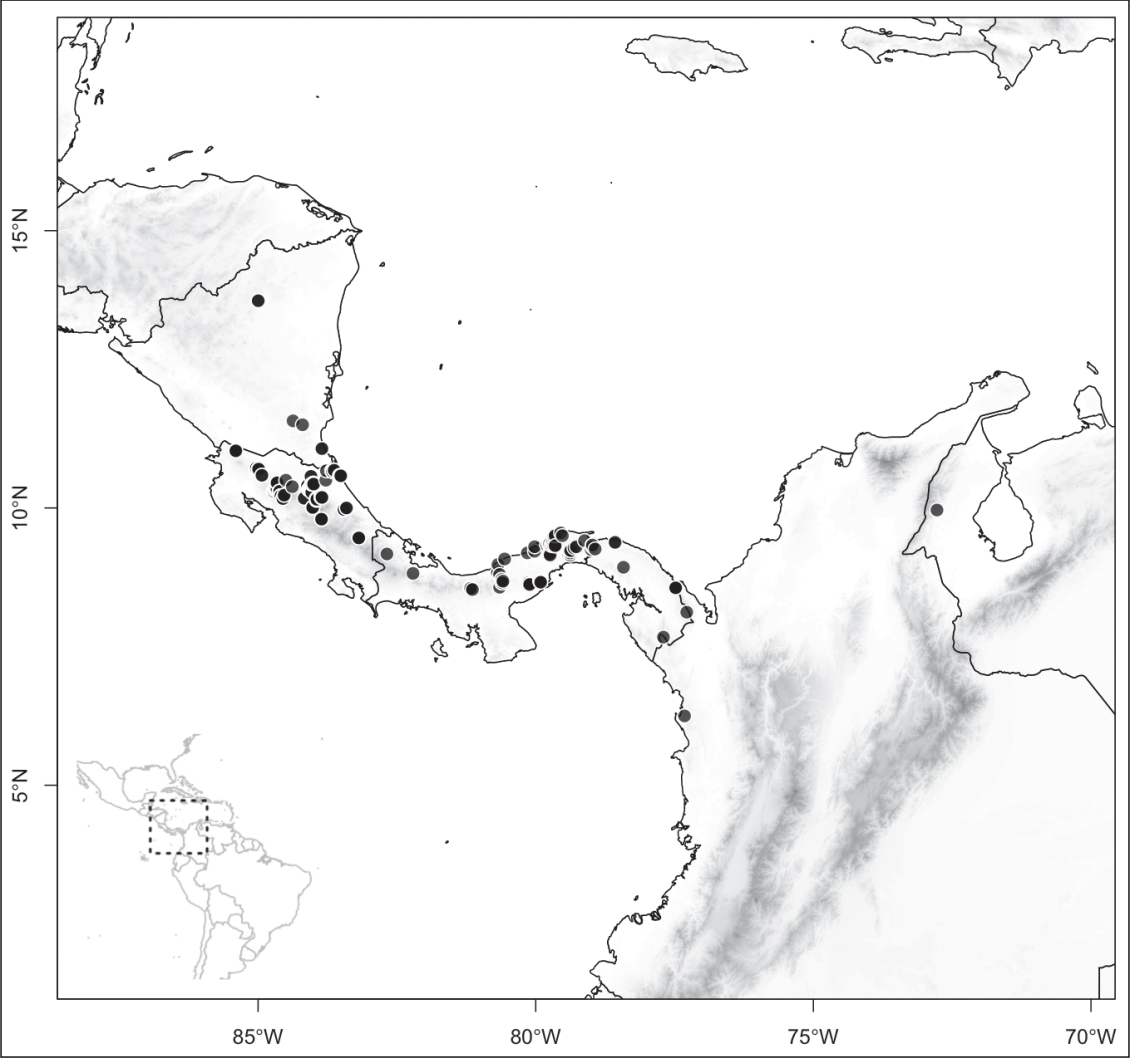
Distribution of *Conostegia consimilis*.

(Fig. 148). Nicaragua, Costa Rica, Panama and reaching the north Pacific part of Colombia, also an outlying population in Sierra Perijá in Venezuela, from sea level to 2000 m elevation.

*Conostegia consimilis* is a common species especially in lowlands and middle elevation in Costa Rica and Panama. It can be recognized by its large leaves with decurrent bases and strongly plinerved venation. Its flowers are quite small and its petals are quite narrow and somewhat acute at the apex which prompted in its original description in the genus *Leandra*. Other useful characters are its yellow anthers, exserted styles, and angulate seeds with verruculose angles. In general, *Conostegia consimilis* shares its rusty indument and petal shape with its close relatives which include *Conostegia iteophylla*, *Conostegia jefensis*, *Conostegia peltata*, and *Conostegia trichosantha*. Of the latter taxa, it is most similar to *Conostegia iteophylla* and the common morphotype of *Conostegia jefensis*. From *Conostegia iteophylla* it can be distinguished by its much larger leaves, and from *Conostegia jefensis* by its white petals (vs. magenta). The drawing in the protologue (Almeda 2000) evidences the exserted style which is common in section *Geniculatae*.

#### Specimens examined.

**COLOMBIA** (fide Wurdack). **Chocó**: S of ridge of Cerro Mecana, Juncosa 1837 (MO).

**NICARAGUA. Zelaya**: SW flank of Cerro Hormiguero, Grijalva 440 (CAS).

**COSTA RICA. Alajuela**: Caribbean slope between San Lorenzo and Los Angeles de San Ramón, above the río San Lorenzo, Burger and Antonio 11175 (F, CR, NY); Guatuso, P. N. Volcán Tenorio, El Pilón, orillas de sendero Los Misterios del Tenorio, Chaves 868 (INB, NY); San Carlos, La Marina, bosque vecino a fuente de Aguas termales en Río Rafael, Jiménez 2326 (CR, NY); San Carlos, Fortuna, R. B. Arenal Mundo Aventura, Rodríguez 8999 (INB, NY). **Heredia**: Sarapiquí, El Ceibo, Kriebel et al. 4814 (INB, NY). **Limón**: P.N. Braulio Carrillo, Sector Quebrada Gonzalez, sendero Las Palmas, Kriebel 1416, 5323 (INB, NY); Pococí, Finca del bosque lluvioso propiedad del INBio, Rodriguez and Vargas 5150 (INB, MO, NY).

**PANAMA. Coclé**: forest slopes above El Copé of the abandoned road leading to the Continental Divide, Almeda et al. 6402 (CAS, NY). **Colón**: Santa Rita Ridge road 8.3 road miles east of Transisthmian highway, along trail north of road, McPherson 7474 (MO, NY). **Darién**: Primary forest along headwater of Río Turquesa ca 2 km air distance from Continental Divide in vicinity of upper gold mining camp of Tyler Kittredge, Croat 27174 (MO, NY). **Panamá**: a lo largo de camino a Cerro Campana, Correa et al. 1027 (MO, NY); Cerro Jefe, Dwyer 9415 (MO, NY). **Veraguas**: Southern shore of Ensenada Santa Cruz, northern tip of Coiba Island, Foster 1632 (MO, NY); P. N. Santa Fé, aproximadamente de 3 a 6 km pasando la Escuela Agrícola Alto de Piedra, Kriebel and Burke 5726 (NY, PMA).

**VENEZUELA** (fide Schnell). **Zulia**: Caño Helena, Sierra Perijá, Delascio and Benkowski 3197 (US).

### Conostegia dissitiflora

Taxon classificationPlantaeMyrtalesMelastomataceae

(Almeda) Kriebel comb. nov.

urn:lsid:ipni.org:names:77156250-1

Fig. 149

Conostegia dissitiflora (Almeda) Kriebel. Basionym: Miconia dissitiflora Almeda, Proc. Calif. Acad. Sci, series 4, 46(5): 146, f. 5. 1989. Type: Costa Rica. Puntarenas: above Golfito along road to television tower, elev. 50–500 m, 16 Jul 1977, F. Almeda, Wilbur, R. and T. Daniel 3093 (holotype: CAS!, isotypes: BM, BR, CR!, DUKE, F!, MO!, NY!, US!).

#### Description.

Sparingly branched shrub 1–3 m tall with terete and glabrous internodes; the distal branches moderately to sparingly stellate-furfuraceous; the nodal line present. Leaves of a pair somewhat unequal in size, sessile and clasping or with petioles 1–9 mm long. Leaf blade 4–22 × 1.3–10 cm, 5–7 plinerved, with the innermost pair of veins diverging 0.4–3.5 cm above the blade base in sub opposite or alternate fashion, elliptic to elliptic ovate, the base rounded to subcordate and sometimes oblique, the apex acuminate, the margin undulate-dentate to subentire, adaxially glabrous, abaxially glabrous or with some stellate hairs on the elevated primary veins. Inflorescence a terminal, erect or reflexed divaricately branched, paniculiform dichasium, 6.4–20.5 cm long, branching at or above the base, accessory branches absent, branches glabrescent, thin and wiry, bracteoles paired, sessile and fused into a short nodal collar forming an elevated internode ridge, 0.5 mm long, lance triangular to subulate, persistent. Pedicels 1.5–2.5 mm long. Flowers 5 merous, calyx in bud not calyptrate but closed in bud and crowned by an apiculum, rupturing irregularly at anthesis into 2–5 hyaline lobes 1–1.5 mm long. Flower buds 1.75–2.25 × 1.5–1.75 mm, hypanthium 1.5–1.85 × 1.5–1.65 mm, campanulate, glabrous to stellate puberulent, calyx tube ca. 0.5 mm long, the calyx teeth subulate, 0.5 mm long. Petals 3.25–3.75 × 1.5–2 mm, translucent white, oblong-lanceolate, reflexed at anthesis, the apex acute to retuse, glabrous. Stamens 10, alternately unequal with the larger stamens inserted on the torus opposite the petals and the small ones opposite the calyx lobes, radially arranged around the style, the filaments 1.5–2 mm, with a geniculation near the apex, white, anthers 1.5–2 × 0.5 mm, linear-oblong, yellow, laterally compressed, the pore 0.15 mm, somewhat dorsally inclined. Ovary 5-locular, inferior, minutely puberulent at the apex. Style 2.5–3 mm, straight, vertical distance from the anther to the stigma 1.25–1.5 mm, horizontal distance absent, stigma truncate, 0.3–0.5 mm wide. Berry 3–4 × 3–4 mm, purple black. Seeds 0.36–0.7 mm, mostly ovoid, with densely muricate or verrucose testa.

**Figure 149.**
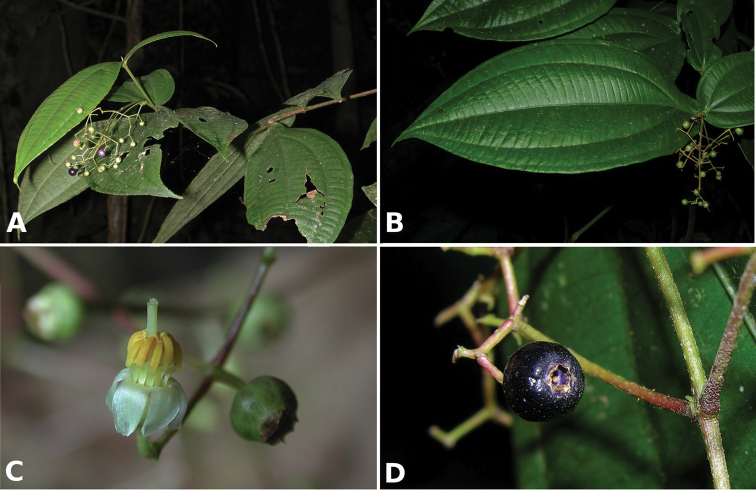
*Conostegia dissitiflora*. **A** Habit **B** Leaf adaxial surface **C** Close up of a flower **D** Berry. Photos of **A** and **C** of specimen vouchered *R. Kriebel 5070*, and **B, D** vouchered *R. Kriebel 5378*.

#### Distribution

**Figure 150.**
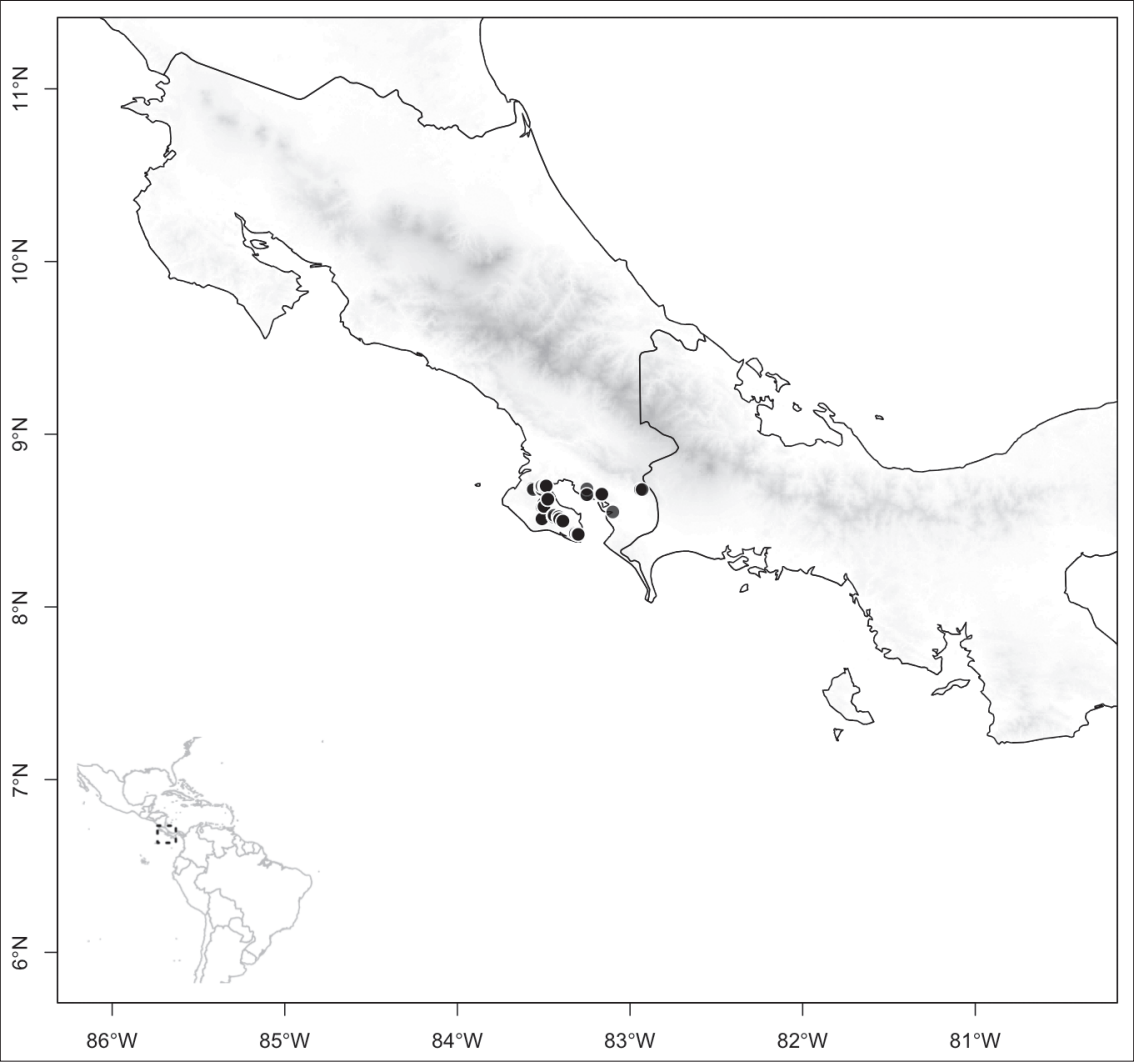
Distribution of *Conostegia dissitiflora*.

(Fig. 150). Endemic to south Pacific Costa Rica, mainly in the Golfo Dulce region and the Osa Peninsula but also towards to mountains on the road from San Vito de Coto Brus to Ciudad Neily, from sea level to 660 m elevation.

*Conostegia dissitiflora* is a distinctive species on the basis of its overall glabrous, plinerved, sessile leaves, and 5-merous flowers with an irregularly rupturing calyx. Almeda (1989a) discussed an unusual feature of this species which is the presence of “anisomorphic” stamens, with the larger set of stamens positioned opposite the petals. The three flowering populations observed had flowers with exserted styles as is common in section *Geniculatae*. Interestingly, when Almeda (1989a) described this species, he compared it to *Conostegia cinnamomea* which it falls very close to in the molecular phylogeny. The two share almost complete glabrosity and plinerved venation. Almeda (1989a) discussed similar problems in placing *Conostegia dissitiflora* in Cogniaux’s sections as he encountered placing *Conostegia calocoma*. The problems arose because staminal characters including anther shape and lack of stamen appendages suggested section *Amblyarrhena*, but a fused calyx suggested section *Laceraria*.

#### Specimens examined.

**COSTA RICA. Puntarenas**: Sierpe, Reserva Forestal Golfo Dulce, Rincón, cerca de la desembocadura del Río Rincón, Aguilar 11413 (NY); R. F. Golfo Dulce, Península de Osa, Río Tigre, Quebrada Pizote, Azofeifa 783 (INB, NY); R. F. Golfo Dulce, Península de Osa, Puerto Jiménez, Río Nuevo, Kriebel et al. 5070 (INB, NY); Península de Osa, Guadalupe La Tarde, Kriebel et al. 5378 (INB).

### Conostegia dissitinervia

Taxon classificationPlantaeMyrtalesMelastomataceae

(Kriebel, Almeda & A. Estrada) Kriebel comb. nov.

urn:lsid:ipni.org:names:77156251-1

Fig. 151

Conostegia dissitinervia (Kriebel, Almeda & A. Estrada) Kriebel. Basionym: Miconia dissitinervia Kriebel, Almeda & A. Estrada, Proc. Calif. Acad. Sci, series 4, 56(37): 678, f. 2A–C, f. 3A–G. 2005. Type: Costa Rica. San José: Turrubares, San Juan de Mata, Lajas, área no protegida, 942'20"N 8435'13"W, 600 m, 26 November 2001, A. Estrada, Chacón, R., & A. Ruiz, et al. 3101 (holotype: CR!, isotype: CAS!, INB!, MO).

#### Description.

Small tree 2–5 m tall with somewhat angled stems that become terete and which are densely covered with lightly orange stellate hairs; the nodal line the nodal line evident. Leaves of a pair equal to unequal in length. Petiole 0.8–3.8 cm. Leaves at a node equal to unequal in size. Leaf blade 10–44.1 × 5–17.2 cm, 3–5 plinerved, with the inner pairs of subparallel veins arising up to about 8 cm above the base in opposite to alternate fashion, elliptic to elliptic ovate, the base acute to decurrent on the petiole, the apex acuminate to long-acuminate, the margin entire to inconspicuously crenulate, the adaxial surface glabrous when mature,the abaxial surface whitish from the entire cover of stellate trichomes. Inflorescence a terminal erect panicle 7.8–13.2 cm long branching above the base, accessory branches present, the axis covered with stellate hairs, bracteoles 1–2 × 0.25–0.5 mm, linear, caducous. Pedicel 0.25–0.5 mm long. Flowers 5-merous, calyx in bud not calyptrate but closed in bud and crowned by an apiculum, rupturing irregularly at anthesis into 2–5 hyaline lobes 0.75–1.25 mm long, buds 2.5–4 × 1.4–2.3 mm, the hypanthium urceolate, 1.75–2.25 × 1.75–2 mm, covered by stellate hairs, calyx tube 0.25 mm long, the calyx teeth narrowly triangular, 0.15–0.35 mm long, torus glabrous. Petals 1.5–2 × 1.25–1.75 mm, translucent white, oblong to ovate, reflexed at anthesis, the apex rounded to emarginate, papillose adaxially. Stamens 10, 2.5–3.5 mm long, radially arranged around the style, the filaments 1.5–2 mm long, with a geniculation near the apex, white, anthers 1.25–1.75 × 0.25–0.75 mm, linear-oblong, yellow, laterally compressed, apiculate at the apex, the pore ca. 0.15 mm, ventrally inclined. Ovary 5-locular, inferior. Style 4–5.25 mm long, straight to slightly gradually bent, vertical distance from the anther pore to the stigma 1.25–2.75 mm, horizontal distance absent, stigma punctiform, 0.35–0.5 mm wide. Berry 4–5 × 4–5 mm, purple black. Seeds 0.3–0.5 mm, pyramidate, the testa muriculate to papillate.

**Figure 151.**
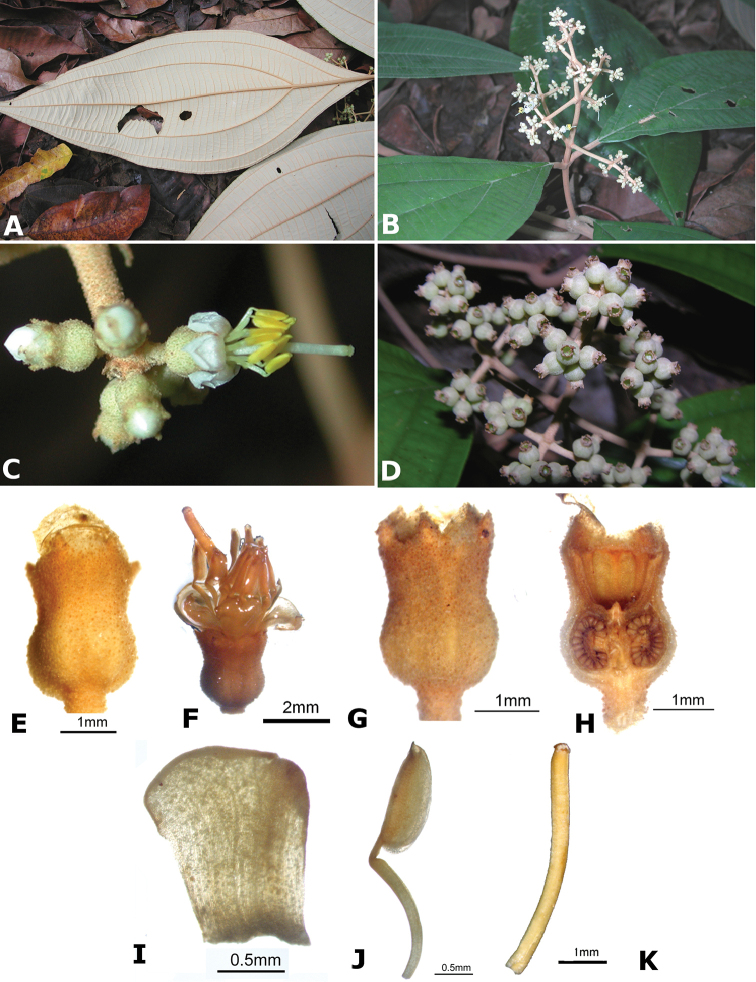
*Conostegia dissitinervia*. **A** Leaf abaxial surface. Inflorescence **C** Close up of flower **D** Infructescence **E** Flower bud with irregularly rupturing calyx **F** Pickled flower **G** External view of a hypanthium at anthesis with all parts removed **H** Internal view of a hypanthium at anthesis with all parts removed **I** Petal **J** Stamen **K** Style. Photos of **A–C** of specimen vouchered *R. Kriebel 5046*, and **D–K** of specimen vouchered *R. Kriebel 5377*.

#### Distribution

**Figure 152.**
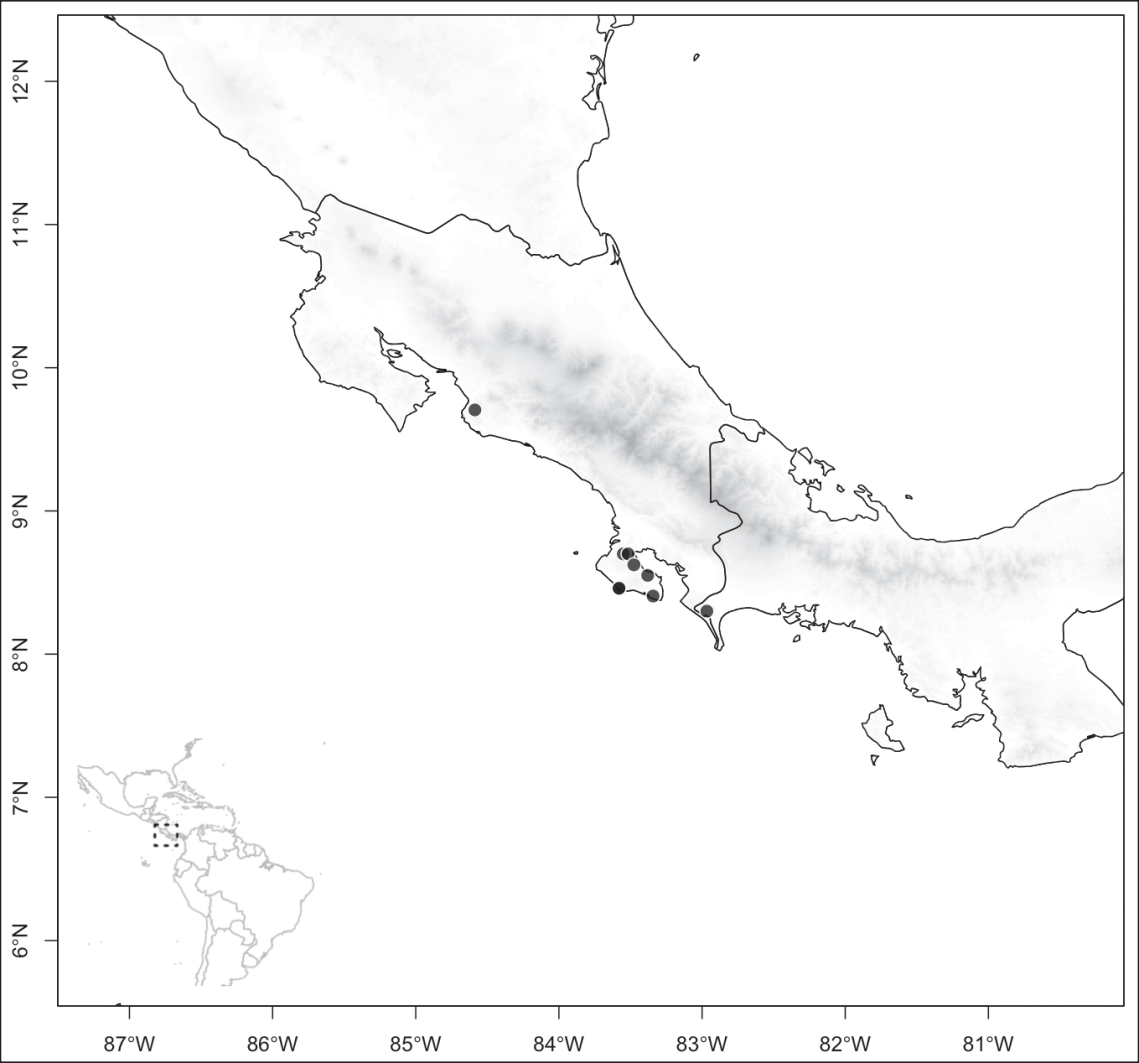
Distribution of *Conostegia dissitinervia*.

(Fig. 152). Restricted to central and south Pacific slope of Costa Rica and barely getting into Panama, 0–600 m in elevation.

*Conostegia dissitinervia* can be recognized by its large leaves with acute to decurrent leaf bases that are abaxially covered with stellate trichomes and are strongly plinerved. In addition, the flowers have a fused calyx that ruptures irregularly and this species tends to have a very exserted style as many other species in section *Geniculatae*. The acute anther apex is distinctive. This species has been confused with *Miconia argentea* in herbaria because of the similar colored abaxial leaf surface as a result of the leaf indument. They are easily distinguished because *Miconia argentea* has nerved leaf venation whereas *Conostegia dissitinervia* is strongly plinerved. In the molecular phylogeny this species forms a well supported sister pair with *Conostegia centrosperma* which it resembles. See the discussion under the latter species for the differences between the two.

#### Specimens examined.

**COSTA RICA. Puntarenas**: Península de Osa, about 5 km west of Rincón de Osa, Burger and Liesner 7253 (NY); Península de Osa, Cerca del Río Piro, Kriebel et al. 5046 (INB, NY); Península de Osa, Guadalupe La Tarde, Kriebel et al. 5377 (INB, NY).

### Conostegia ecuadorensis

Taxon classificationPlantaeMyrtalesMelastomataceae

(Gleason) Kriebel comb. nov.

urn:lsid:ipni.org:names:77156252-1

Conostegia ecuadorensis (Gleason) Kriebel. Basionym: Clidemia ecuadorensis Gleason, Bull. Torrey Bot. Club 66(6): 418. 1939. Type: Ecuador. Esmeraldas: Parroquia de Concepción, Playa Rica, alt. 105 m, 10 December 1936, Y. Mexía 8431 (holotype: NY!, isotypes: BM, CAS!, F!, K, S, US!).

#### Description.

Shrub to small tree 2–3.5 m tall with terete, caducously furfurate-lepidote branches; the nodal line not evident. Leaves of a pair somewhat unequal in size. Petiole 0.4–1.3 cm. Leaf blade 11–14 × 2.5–5.2 cm, 3–5 plinerved with the innermost pair of lateral veins arising about 1–2 cm above the base and usually diverging from each other at their point of origin, elliptic, cuneate, apex caudate-acuminate, the margin entire, pocket like domatia present at the base abaxially on both pairs of lateral veins, adaxially glabrous, abaxially glabrous or caducously lepidote. Inflorescence an cyme axillary cyme 1.8–3.2 cm long, divaricately branched form the base with slender branches, accessory branches absent, the rachis inconspicuously furfurate-lepidote, bracteoles 2.2–5 cm long, triangular to subulate and fused basally to forma a peristent, shallow amplexicaul collar, 1–1.5 × 0.5–1 mm, persistent. Flowers sessile or subsessile, 4 (-5) merous, not calyptrate nor with the calyx lobes fused in bud, the hypanthium 2.25–2.75 × 1.75–2.25 mm, glabrous, calyx lobes broadly triangular ovate, 0.5–1 mm long, calyx teeth subulate, 0.5 mm long. Immature petals 1.5 × 1.2 mm, triangular ovate, posture not seen live at anthesis, glabrous, apically broadly acute. Stamens not studied, reportedly 8 (-10) in number. Ovary 4 (-5) locular, inferior. Style ca. 5 mm long, not observed in good flower at anthesis. Berry 4–5 × 4–5 mm, dark purple to black. Seeds 0.4–0.6 mm long, more or less triangular in outline, the testa muriculate.

#### Distribution

**Figure 153.**
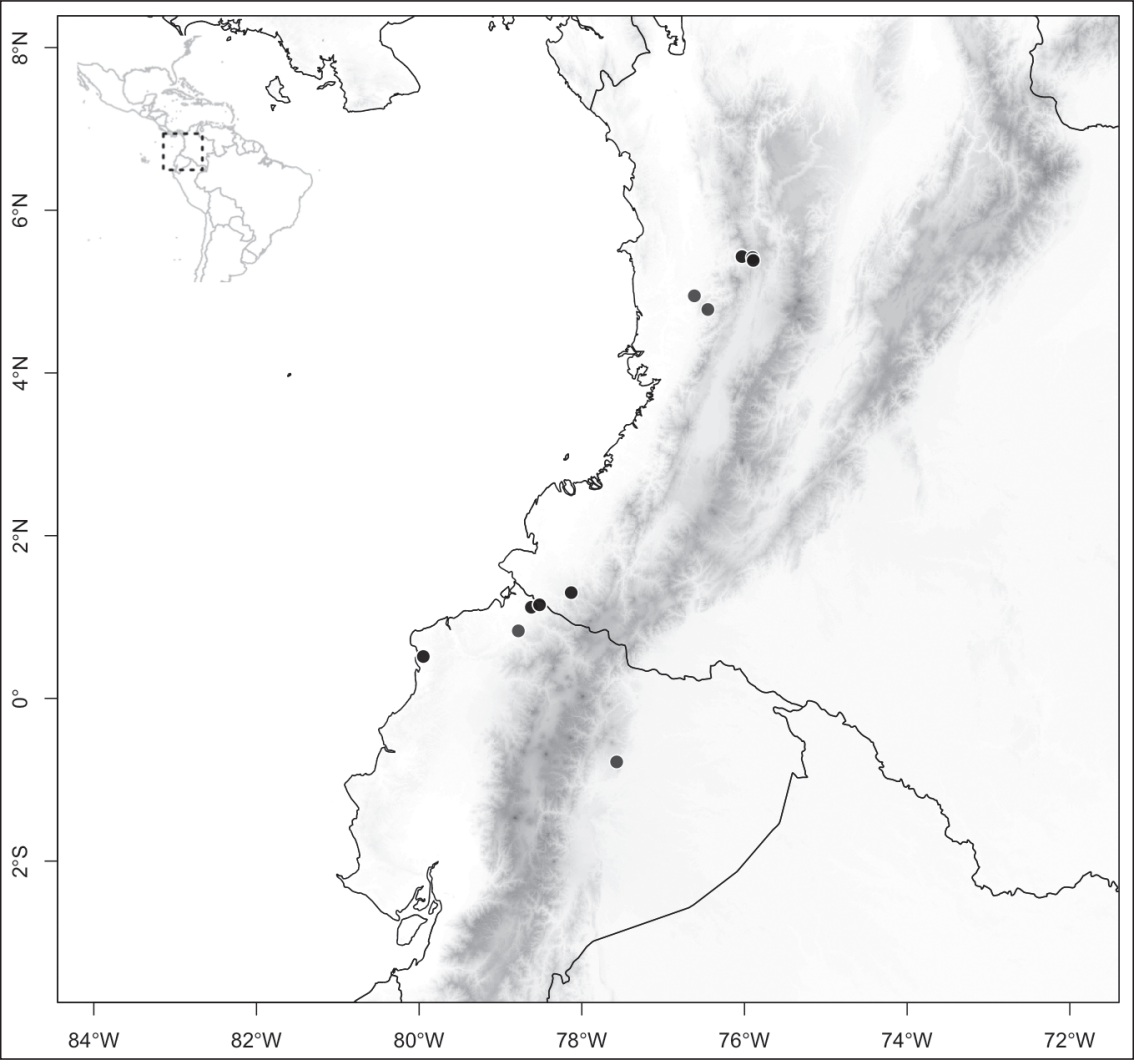
Distribution of *Conostegia ecuadorensis*.

(Fig. 153). On the Pacific slope of Colombia and north western Ecuador with a population on the eastern side of the Andes in Ecuador, 100–2000 m in elevation.

*Conostegia ecuadorensis* can be recognized by its mostly glabrous vegetative parts, its caudate leaf apex forming a long drip tip, the presence four pocket domatia at the base of the leaf on the abaxial side where each of the major lateral veins arises, and its 4-merous flowers on divaricately branched slender inflorescences branches. Gleason described *Conostegia ecuadorensis* in the same year that he described *Conostegia ombrophila* (Gleason 1939a,b). These two species are quite similar in their almost glabrous vegetative parts with inconspicuous lepidote hairs, presence of leaf domatia, and caudate leaf apices, so it is surprising he did not comment on the similarities between the two. In fact, they can be hard to separate, with *Conostegia ecuadorensis* having four domatia and a more southern distribution, and *Conostegia ombrophila* none or usually two domatia and a more southern Central American distribution. Instead, Gleason (1939b) compared *Conostegia ecuadorensis* to *Clidemia japurensis* and *Clidemia naevula* (now considered a synonym of the *Clidemia japurensis*) of section *Staphidium* (where he placed, *Conostegia ecuadorensis*), two glandular pubescent species that we know now, are distantly related from *Conostegia ecuadorensis* based on molecular phylogenies. On the other hand, he did not place *Conostegia ombrophila* in any section or compare to any other species, stating that its position in *Clidemia* was uncertain.

#### Specimens examined.

**COLOMBIA. Chocó**: Vereda Llanadas, Ladera Norte del Cerro Torrá, Fila al Oeste, Forero et al. 1977 (MO, NY). **Risaralda**: Mistrató, corregimiento de Jeguadas, Betancur et al. 3202 (MO, NY). ECUADOR. NAPO: Carretera Sumaco-Galeras 2km N of Rio Papauco, Stern and Tepe 328 (CAS, NY).

### Conostegia foreroi

Taxon classificationPlantaeMyrtalesMelastomataceae

(Wurdack) Kriebel comb. nov.

urn:lsid:ipni.org:names:77156253-1

Fig. 154

Conostegia foreroi (Wurdack) Kriebel. Basionym: Clidemia foreroi Wurdack, Phytologia 64(4): 300–301. 1988. Type: Colombia. Chocó: Alrededores de San José del Palmar, cerro SO de la población, 1370 m, 1 September 1976, E. Forero & R. Jaramillo 2455 (holotype: COL!; isotypes: MO!, US!).

#### Description.

Shrub 1–1.5 m tall with terete branches that have a sparse to dense covering of smooth spreading hairs (2–3.5 mm) intermixed with inconspicuous, early deciduous, asperous headed underlain by a moderate to dense understory of stellulate-furfuraceous or short asperous headed hairs but that become glabrate at maturity; the nodal line absent. Leaves at a node equal to somewhat unequal in size. Petiole 0.5–1.5 cm. Leaf blade 6–10 × 2.5–4 cm, 5-nerved to slightly plinerved, elliptic, the apex acute to acuminate, the margin entire to crenulate, adaxially moderately strigose to subhirsute with hairs mostly 1–2 mm long, abaxially moderately hirsute with a mixture of smooth hairs (1–2.5 mm long) and minute glandular hairs essentially restricted to the primary and higher order veins. Inflorescence a pseudolateral modified dichasium 3–5 cm long divaricately branched at the base and appearing axillary on older nodes, accessory branches absent, rachis sparingly setulose, bracteoles lanceolate to ovate, 1.5–3 × 1–1.5 mm, paired at each node and persistent, setose in between the bracteoles. Pedicels 2–3 mm long. Flower buds 2 × 1.5 mm, the hypanthium campanulate, ca. 1.5–2 mm long, densely covered with spreading smooth hairs 1–2 mm long and a sparse understory of sessile stellulate furfuraceous hairs. Flowers 5-merous, calyx not calyptrate but closed in bud and crowned by an apiculum 0.5 mm long and rupturing irregularly at anthesis into 3–5 hyaline, persistent lobes 0.5–1 mm long, external calyx teeth setiform, 0.5–1 mm long, the torus glandular puberulent. Petals 2.8–3.4 × 1.3–1.5 mm, translucent white, oblong to oblong ovate, spreading at anthesis, rounded apically. Stamens 10, 2.7–3.8 mm long, radially arranged around the style, the filament 1.5–2 mm with a geniculation near the apex, translucent white, anthers 1.2–1.6 × 0.35–0.55 mm, yellow, oblong and not compressed, the connective thickened, the pore 0.1 mm wide, slightly dorsally inclined. Ovary 5 locular, 2/3 inferior, apex glandular-puberulent lobulate collar. Style 4–4.5 mm long, straight to slightly bending, vertical distance from the anther to the stigma ca. 1.25–1.5 mm, horizontal distance absent; stigma capitellate, 0.3–0.5 mm wide. Berry and seeds not seen.

**Figure 154.**
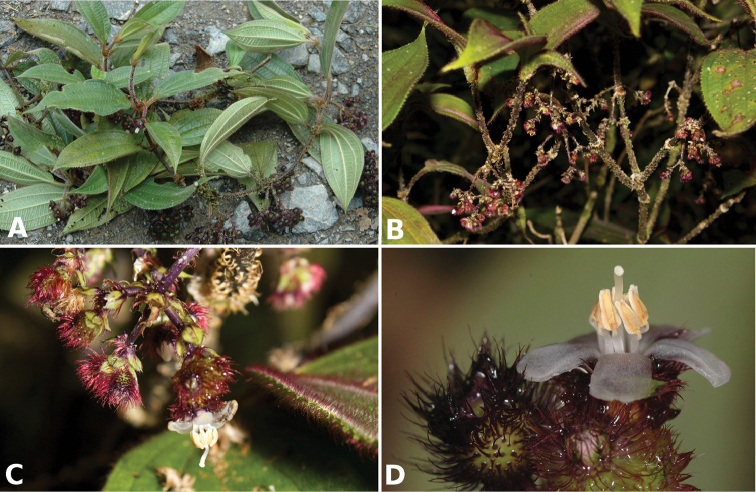
*Conostegia foreroi*. **A** Habit **B** Habit with detail of multiple pseudolateral inflorescences **C** Close of an inflorescence showing multiple foliaceous bracteoles **D** Close of a flower. Photographs **A, D** by Frank Almeda from voucher *F. Almeda 10336*, photographs **B, C** by Paola Pedraza-Peñaloza from voucher *Pedraza-Peñaloza et al. 1923*.

#### Distribution

**Figure 155.**
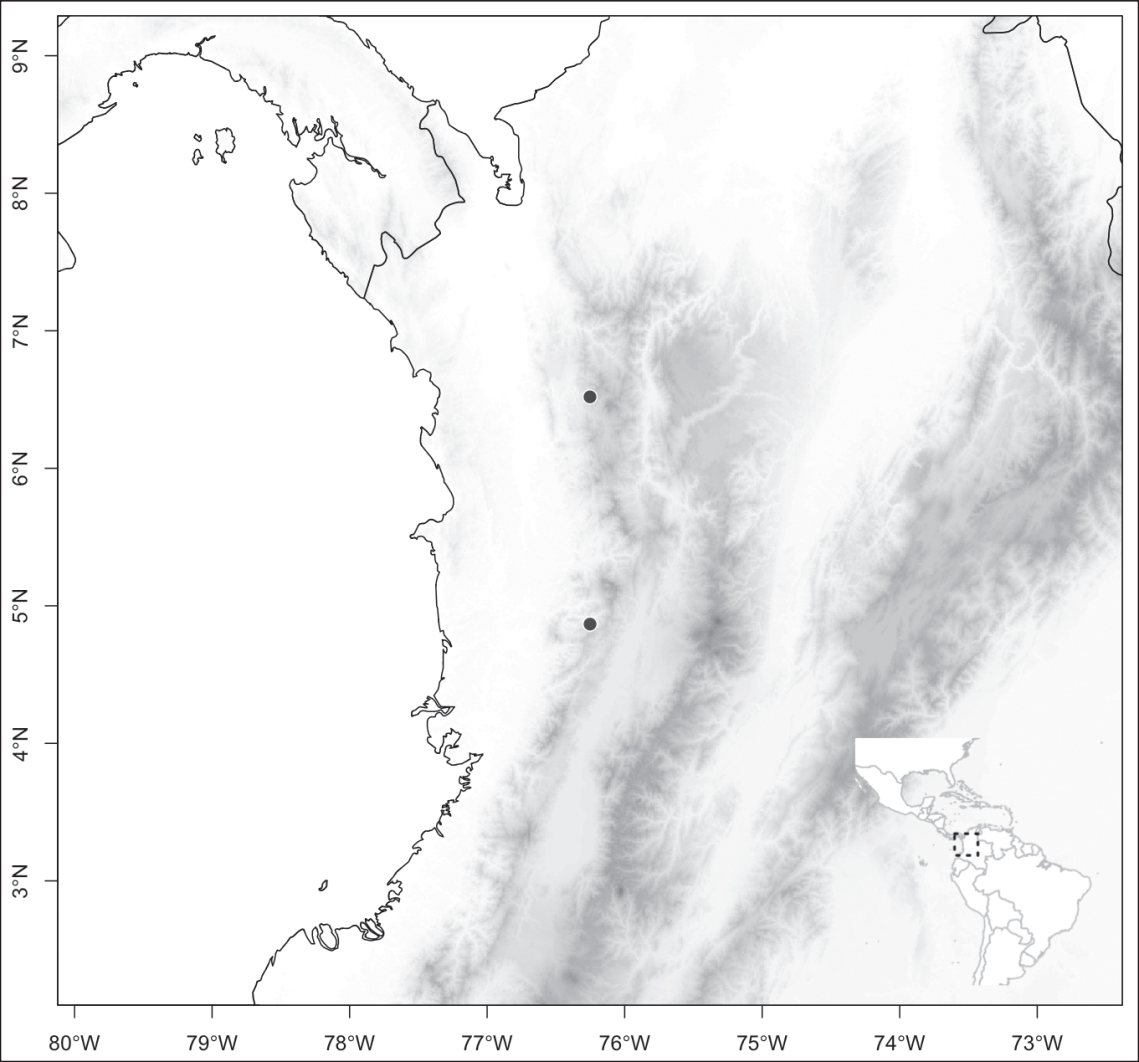
Distribution of *Conostegia foreroi*.

(Fig. 155). Endemic to the the departments of Antioquia and Chocó in Colombia, 1300–1400 m in elevation.

*Conostegia foreroi* is a very uncommon species characterized by its dense pubescence on stems, leaves, inflorescences and hypanthia. As Wurdack (1988) mentioned in the protologue, it resembles *Clidemia costaricensis* and *Clidemia petiolaris*. The latter two species thus also resemble each other but none of the three are closely related based on the molecular phylogeny, evidencing the possible multiple evolutions of shrubby and densely pubescent species. Among the differences between *Conostegia foreroi* that help distinguish it from *Clidemia costaricensis* and *Clidemia petiolaris* are the flattened setae on the nodes, smaller flowers, a sparsely glandular pubescent ovary apex and stamens that have a stronger geniculation. Within section *Geniculatae*, *Conostegia foreroi* appears to resemble more *Clidemia hammelii*. The latter species is overall less pubescent and has pocket domatia at the base of the leaf on the abaxial surface. This species appears to also converges with *Conostegia trichosantha* in the accumulation of water on the external pubescence of the floral buds. The paratype *Lozano and Díaz 3201* (US) notes extreme herbivory on the plant like in other species of section *Geniculatae* such as *Conostegia calocoma*.

#### Specimens examined.

**COLOMBIA. Antioquia**: Urrao, Corregimiento La Encarnación, vereda Calles, P.N. Natural Las Orquídeas, Pedraza-Peñaloza et al. 1923 (NY).

### Conostegia fraterna

Taxon classificationPlantaeMyrtalesMelastomataceae

(Gleason) Kriebel comb. nov.

urn:lsid:ipni.org:names:77156254-1

Fig. 156

Conostegia fraterna (Gleason) Kriebel. Basionym: Clidemia fraterna Gleason, Brittonia 2(4): 323. 1937. Type: Costa Rica. San José: collected near El General, July 1936, A. Skutch 2687 (holotype: NY!, isotype: GH!, K!, MICH!, S!, US!).

#### Description.

Shrub 1–3 m tall with terete branches densely covered with a stellulate lepidote indument; the nodal line not evident. Leaves at each pair equal to unequal in size. Petiole 0.5–1.5 cm. Leaf blade 5–17 × 2.9–6.7 cm, 5-plinerved, with the innermost pair of veins diverging from the midvein 1–5 cm above the base in alternate or opposite fashion, elliptic, the margin entire, the base decurrent on the petiole, the apex acuminate, adaxially glabrous, abaxially deciduously stellulate lepidote but appearing glabrous. Inflorescence an openly branched pseudolateral cyme 2.5–5 cm long borne on both leafy and defoliated nodes, branching at or above the base into pedunculate mostly 3-flowered glomerules, accessory branches absent, rachis inconspicuously stellulate lepidote, paired bracteoles subtending each glomerule ovate, 2–3 × 2 mm, persistent and fused at the base, paired bracteoles subtending each flower elliptic-ovate, 2–2.5 × 1.5 mm, persistent. Flower buds 2.5–4 × 2.5–2.75 mm. Flowers 5-merous, sessile, calyx not calyptrate but fused in bud, rupturing at anthesis into 3–5 ovate hyaline lobes, 0.5–1 × 1 mm, external calyx teeth triangular-subulate, 0.25–0.5 mm long; the hypanthium broadly campanulate, 2–2.5 × 2–2.5 mm long, densely stellate tomentose. Petals 3.5–4.6 × 1.5–2.8 mm, white, oblong-obovate, rounded apically, reflexed at anthesis. Stamens 10, 2.5–3.5 mm long, radially arranged around the style, the filament 1.4–2 mm without an evident geniculation, translucent white, anthers 1.2–1.5 × 0.5–0.75 mm, elliptic-oblong, laterally compressed, yellow, the pore ca. 0.15 mm wide, dorsally inclined. Ovary 5-locular, totally inferior, apex flat and glabrous except for s few minute glands. Style ca. 5.7–5.9 mm long, straight to slightly bending, vertical distance from the anther to the stigma 1.9–2.15 mm, horizontal distance absent, stigma truncate, 0.25–0.37 mm wide. Berry 3.5 × 3.5 mm when dry, purple-black. Seeds ca. 0.5 mm long, pyramidal, the testa asperulate or undulate.

**Figure 156.**
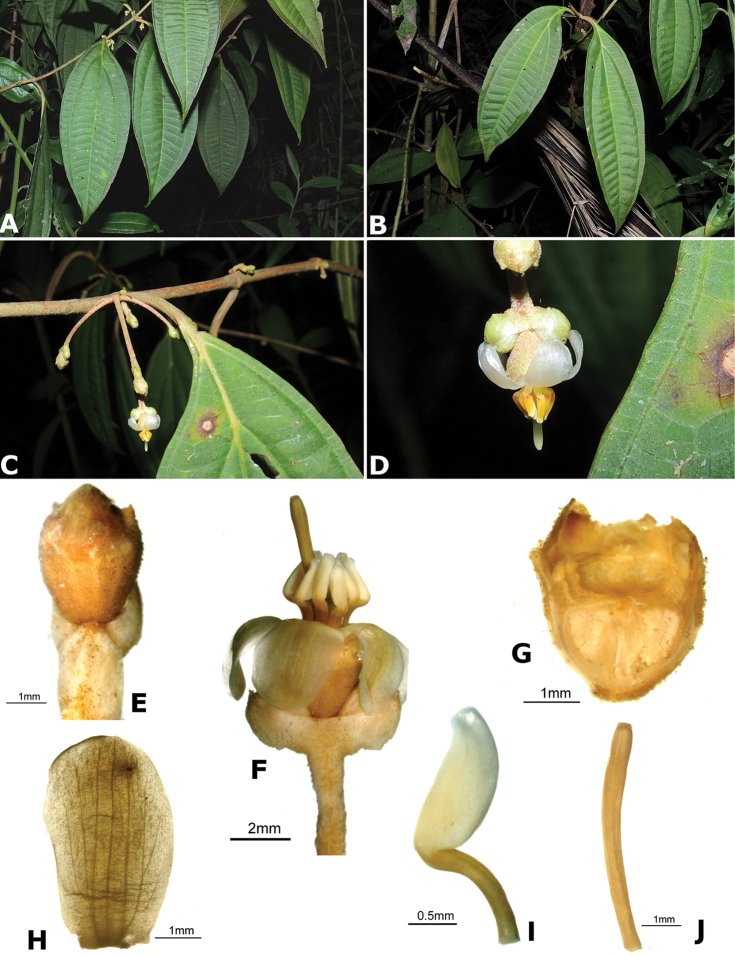
*Conostegia fraterna*. **A** Habit **B** Leaf adaxial surface **C** Inflorescence **D** Close up of flower **E** Close up of flower bud **F** Pickled flower **G** Longitudinal section of a flower at anthesis with all parts removed **H** Petal **I** Stamen **J** Style. Photos of specimen vouchered *R. Kriebel 5774*.

#### Distribution

**Figure 157.**
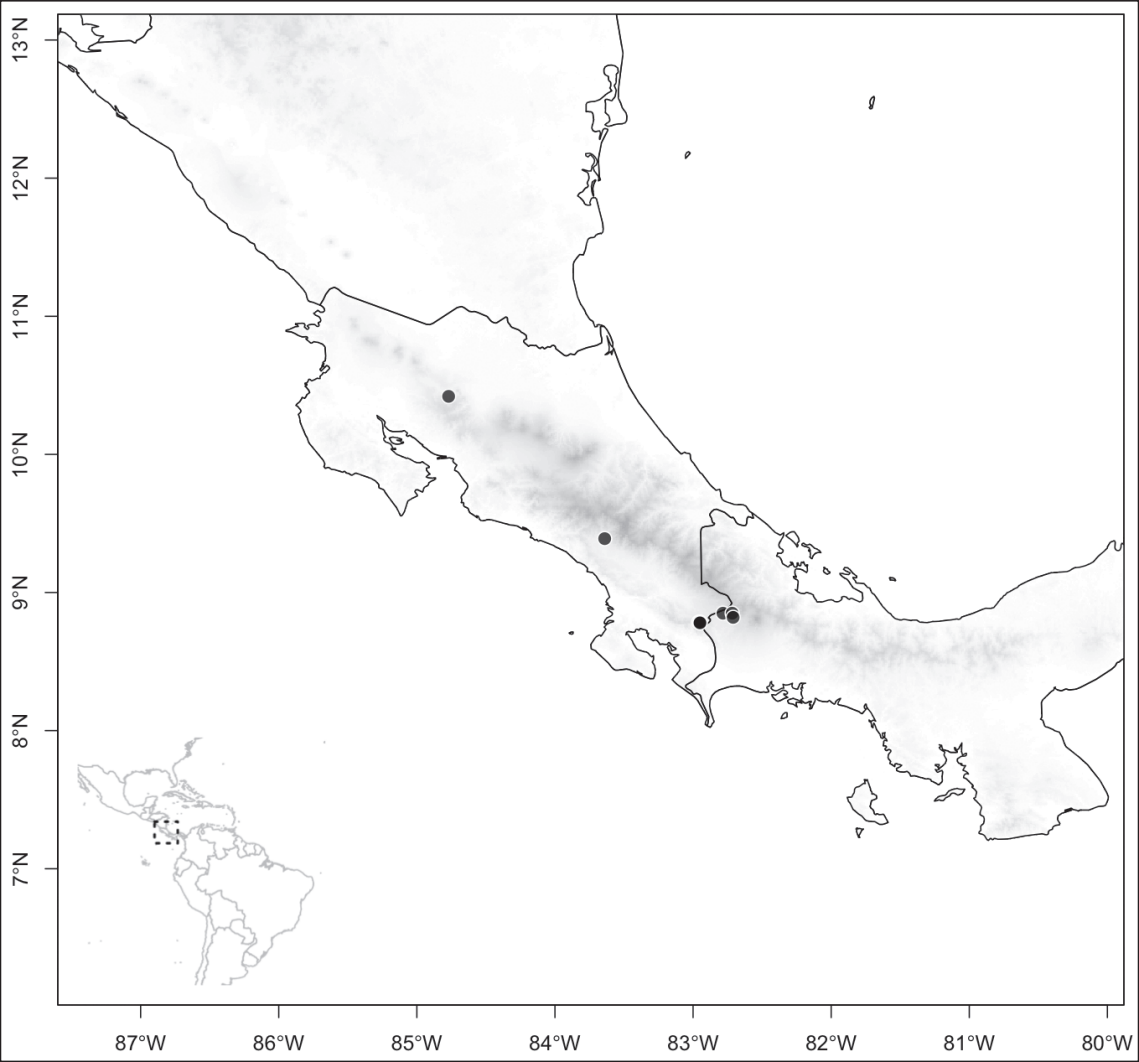
Distribution of *Conostegia fraterna*.

(Fig. 157). On the foothills of the Pacific slope of Talamanca Cordillera in Valle del General and San Vito de Coto Brus and barely reaching neighboring Panama, 950–1300 m in elevation. One outlying population collected on the south side of Lake Arenal in northern Costa Rica.

*Conostegia fraterna* is a distinctive species that can be identified on the basis of its short petiolate, narrow, plinerved leaves, and unusual inflorescence which consists of pedunculate mostly 3-flowered glomerules. This kind of inflorescence is not seen in any other species of *Conostegia*. The sessile flowers with fused buds that rupture at anthesis, herkogamous flowers, and broad anthers are also helpful to place it in section *Geniculatae*. *Conostegia fraterna* did not fall as sister to any species with support in the recent molecular phylogeny. Nevertheless, it fell in a clade that includes similar looking species such as *Conostegia cinnamomea*, *Conostegia dissitiflora* and *Conostegia grayumii*. All these species are glabrous or just barely pubescent and plinerved like *Conostegia fraterna*. Gleason (1937) compared this species to *Clidemia conglomerata* and *Clidemia involucrata*. Of the latter two species, he believed *Clidemia involucrata* to be the closest relative to *Conostegia fraterna*. *Clidemia involucrata* differs in its glandular pubescence, ciliate-serrulate margins and 3-locular ovary. Based on the molecular phylogeny available (Goldenberg et al. 2008), we know they are not closely related at all. A seed micrograph of this species is provided in Ocampo and Almeda (2013).

#### Specimens examined.

**COSTA RICA. Guanacaste**: Río Negro ford on south side of Lake Arenal, 10 km NNE of Santa Elena, Haber et al. 4808 (INB, MO). **Puntarenas**: E. B. Las Cruces, Kriebel 5774 (INB). **PANAMA. Chiriquí**: along road between Volcán and Río Sereno, 13.7 mi W of Volcán, Croat 66335 (CAS, MO); road from Volcán to Río Sereno, 16.0 km from Río Sereno, Folsom 4053 (CAS, MO).

### Conostegia friedmaniorum

Taxon classificationPlantaeMyrtalesMelastomataceae

(Almeda & Umaña) Kriebel comb. nov.

urn:lsid:ipni.org:names:77156255-1

Fig. 158

Conostegia friedmaniorum (Almeda & Umaña) Kriebel. Basionym: Miconia friedmaniorum Almeda & Umaña, Novon 3(1): 5, f. 1. 1993. Type: Costa Rica. Alajuela: Upala, Colonia Libertad, subiendo hasta el Llano Aguacatales, 10 48'25"N, 85 17'50"W, 1500 m, 28 April 1988, G. Herrera 1900 (holotype: CR!, isotype: BR!, CAS!, F!, MEXU!, MO!, PMA, SAC, USJ).

#### Description.

Shrub to small tree 2–5 m tall with somewhat tetragonal and ridged stems in newer branches that are densely covered with inconspicuously stalked reddish-orange asperous-headed hairs usually also with some simple multicellular hairs on the distal internodes; the nodal line nodal line present. Leaves of a pair equal to somewhat unequal in length. Petioles 0.5–4.8 cm. Leaf blades 7.5–18.6 × 3.4–7.7 cm, 5-plinerved, with the innermost pair of veins arising up to about 4 cm above the base in opposite or frequently in strongly alternate fashion, elliptic to elliptic-ovate, the base obtuse to oblique, the apex acuminate,the margin entire to inconspicuously denticulate, the adaxial surface mostly glabrous, moderately to sparsely covered with a mixture of inconspicuously asperous headed and scalelike multicellular hairs on the secondary and higher order veins abaxially. Inflorescence a mostly deflexed terminal modified cyme branching at the base, 3.6–9.8 cm long, accessory branches absent, rachis covered with stalked asperous headed hairs, linear oblong to triangular, bracteoles 1.5–4 mm, persistent. Pedicel 1–1.75 mm long. Flowers 5-merous, calyx in bud not calyptrate but closed in bud and crowned by an apiculum, rupturing irregularly at anthesis into 4–5 hyaline irregular lobes 0.3–1.5 mm long, flower buds 3–4 × 2–2.5 mm, the hypanthium 1.5–2.5 × 1.75–2.25 mm, campanulate, covered with orangish asperous headed hairs; exterior calyx teeth conspicuous, triangular, 0.5–0.6 mm long, torus glabrous. Petals 3–3.5 × 0.9–1.25 mm, translucent pink, linear-oblong, reflexed at anthesis, glabrous, the apex acute. Stamens 10, 2.2–3 mm, radially arranged around the style, the filaments 1–1.75 mm, geniculate near the apex, white, anthers 1–1.25 × 0.25–0.75 mm, linear-oblong, yellow, laterally compressed, the pore 0.1–0.14 mm wide, terminal and slightly dorsally inclined. Ovary 5 locular, 3/4 inferior, slightly fluted and with glandular hairs on the apex. Style 5.25–5.5 mm long, slightly to strongly bending, vertical distance from anther pore to stigma 1.75–2.25 mm, horizontal distance absent or up to1.3 mm, stigma truncate and somewhat dilated, 0.25–35 mm wide. Berry 3–4 × 3–4 mm, red turning purple black when mature. Seeds 0.39–0.6 mm, angular pyramidate to somewhat cescent shaped in profile, the testa smooth.

**Figure 158.**
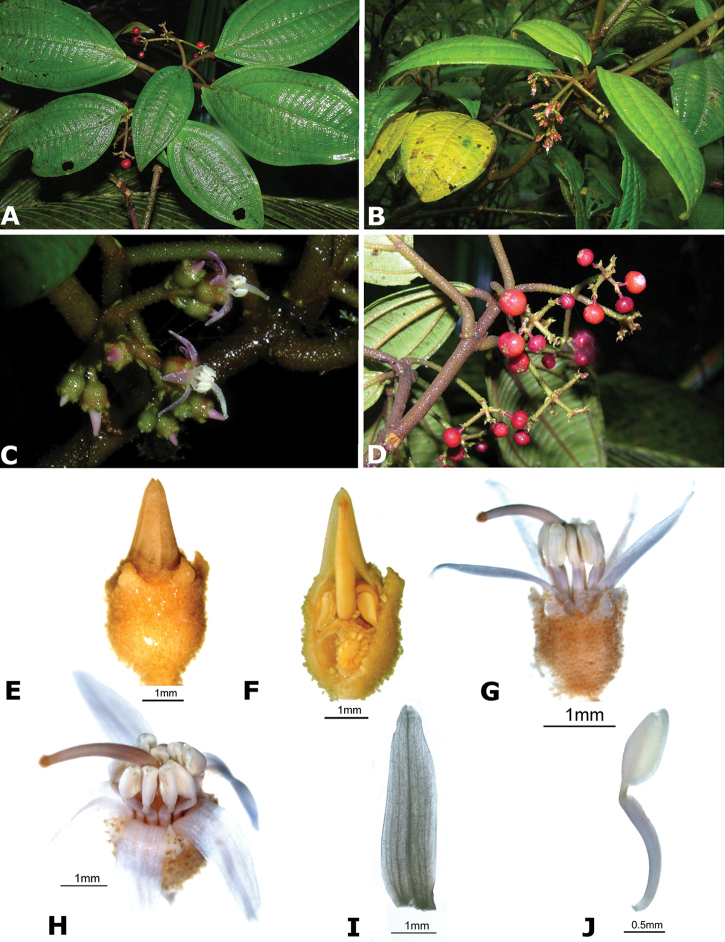
*Conostegia friedmaniorum*. **A** Habit. Note asymmetric leaf base **B** Habit showing deflexed inflorescence **C** Close up of flower **D** Infructescence **E** Flower bud **F** Longitudinal section of a flower bud **G–H** Pickled flowers **I** Petal **J** Stamen. Photos of **A, D** of specimen vouchered *R. Kriebel 5497*, and **B, D** *R. Kriebel 5641*.

#### Distribution

**Figure 159.**
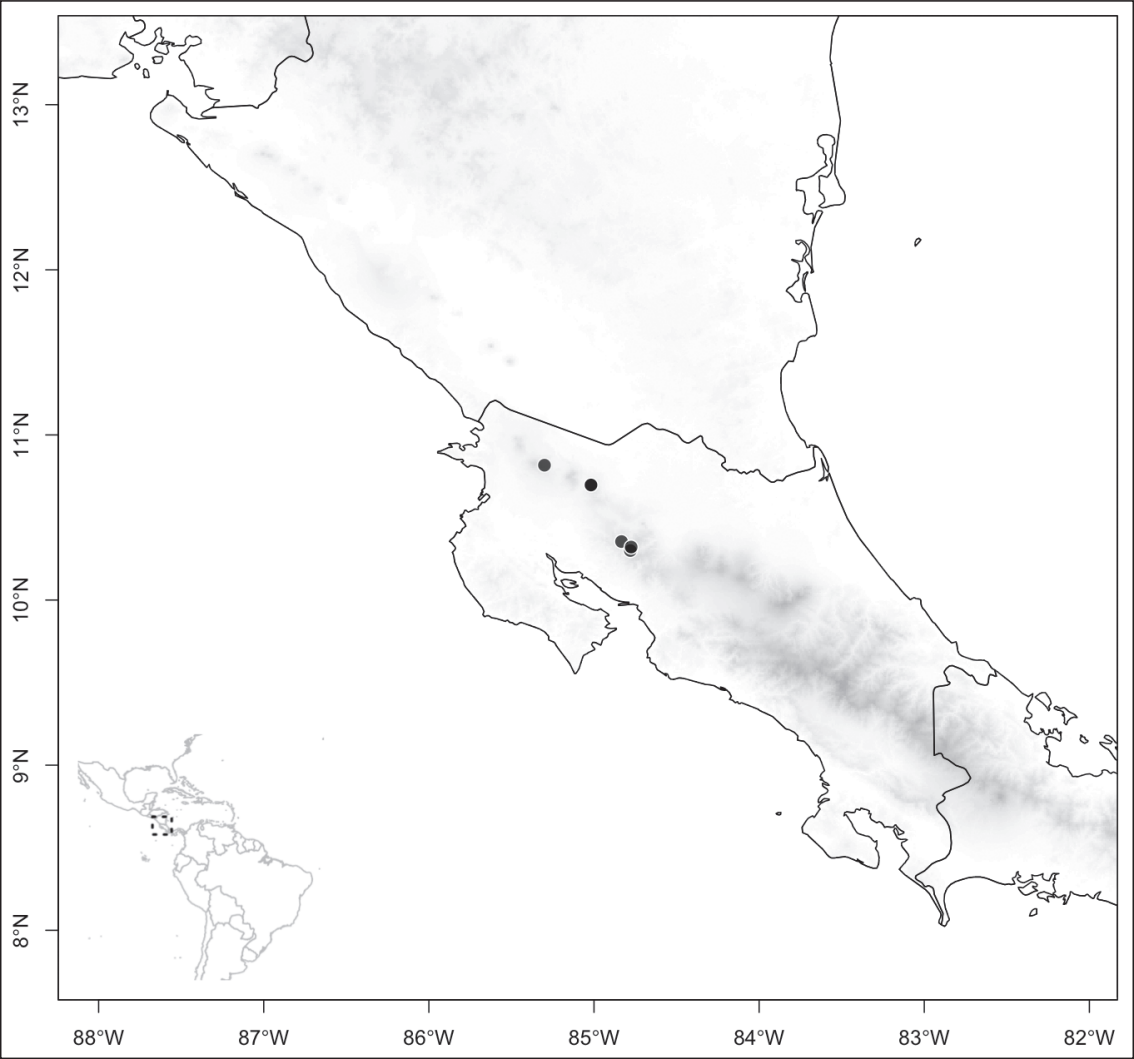
Distribution of *Conostegia friedmaniorum*.

(Fig. 159). Endemic to the Caribbean slope of the Cordillera de Tilarán and Volcán Tenorio National Park in Costa Rica, 1250–1700 m in elevation.

*Conostegia friedmaniorum* can be recognized by its rusty pubescence, leaves which are strongly plinerved with the veins frequently arising in asymmetric fashion, deflexed inflorescences and linear-oblong petals. Vegetatively it resembles another Costa Rican endemic, *Conostegia pendula*, as well as the Panamanian endemics *Conostegia galdamesiae* and *Conostegia papillopetala*. From *Conostegia pendula* it can be distinguished because the latter has lanate indument on the stems which is absent in *Conostegia friedmaniorum*. From *Conostegia galdamesiae* and *Conostegia papillopetala* it can be distinguished by its deflexed inflorescence and linear-oblong petals.

#### Specimens examined.

**COSTA RICA. Alajuela**: Monteverde Reserve, Cerro Negro, continental divide with Atlantic Slope exposure, Bello 3242 (CAS); San Ramón, Valle del Río Peñas Blancas, R. B. Monteverde, Bello 5155 (CAS); P. N. Volcán Tenorio, Estación El Pilón, sendero hacia Cerro Montezuma, Kriebel and Nicholas 5497 (INB, NY); loc. cit., Kriebel et al. 5641 (INB); Border of Alajuela, Guanacaste, and Puntarenas Provinces, Cordillera de Tilarán, sendero El Valle, Almeda and Anderson 5416 (CAS); Border of Puntarenas Alajuela border, Cordillera de Tilarán, Monteverde Cloud Forest Reserve, sendero Brillante along the Continental Divide, Almeda and Daniel 7074 (CAS).

### Conostegia fulvostellata

Taxon classificationPlantaeMyrtalesMelastomataceae

(L. O. Williams) Kriebel comb. nov.

urn:lsid:ipni.org:names:77156256-1

Conostegia fulvostellata (L. O. Williams) Kriebel. Basionym: Miconia fulvostellata, Fieldiana, Bot. 29:571.1963. Type: Guatemala. Huehuetenango: Cerro Chiblac, between Finca San Rafael and Ixcan, Sierra de los Cuchumatanes, alt. 1200–2000 m, 22 July 1942, J. Steyermark 49143a (F fide Almeda (2009)).

#### Description.

Shrubs to small trees 2.5–15 m tall with somewhat flattened cauline internodes that covered by a densely ferrugineus stellate indument; the nodal line inconspicuous. Leaves of a pair equal to somewhat unequal in length. Petioles 1–5 cm long. Leaf blade 7–14 × 2–6 cm, 3–5-plinerved, with the innermost pair of veins diverging in from the midvein 0.3–2 cm above the base in opposite or alternate fashion, elliptic to elliptic lanceolate, the base acute to obtuse and sometimes asymmetric, the apex acuminate, the margin undulate-denticulate, the adaxial surface glabrous, the abaxial surface densely ferrugineus stellate indumentum. Inflorescence a terminal panicle 7–12 cm long, branching at or above the base, accessory branches absent or present, the axes covered with reddish stellate hairs; bracteoles 0.5–1 × 0.25 mm, linear-oblong, deciduous. Flowers 4–5 merous, not calyptrate nor the sepals fused in bud and rupturing, hypanthium 1.6–2.3 × 1.5–2 mm, campanulate, covered with stellate hairs, calyx tube 0.2 mm long, calyx lobes undulate, ca. 0.2–0.5 mm long but almost undifferentiated, calyx teeth barely evident. Petals 2–3 × 1.5–2 mm, white, oblong-obovate, not seen live at anthesis, glabrous or papillose, if papillose with conspicuous papillae at the base, the apex rounded to emarginate. Stamens 8–10, 4–5 mm long, radially arranged around the style, the filaments 2–2.5 mm, with a geniculation near the apex, anthers 2–2.5 × 0.5–0.7 mm, linear-oblong, yellow, laterally compressed, dorsally thickened, the pore ca. 0.2 mm wide, ventrally inclined. Ovary 4–5-locular, 1/4 inferior, the apex densely stellate puberulent, lacking a collar around the style base. Style ca. 5–6 mm long, mostly straight, vertical distance from the anther to the stigma ca. 1.5–2 mm, the stigma capitate, ca. 0.8–0.9 mm wide. Berry 4–5 × 4–5 mm, purple black. Seeds 0.65–0.8 mm long, Seeds pyramidate, rounded-angulate and obscurely puncticulate, 1.5 mm long (fide Almeda 2009).

#### Distribution

**Figure 160.**
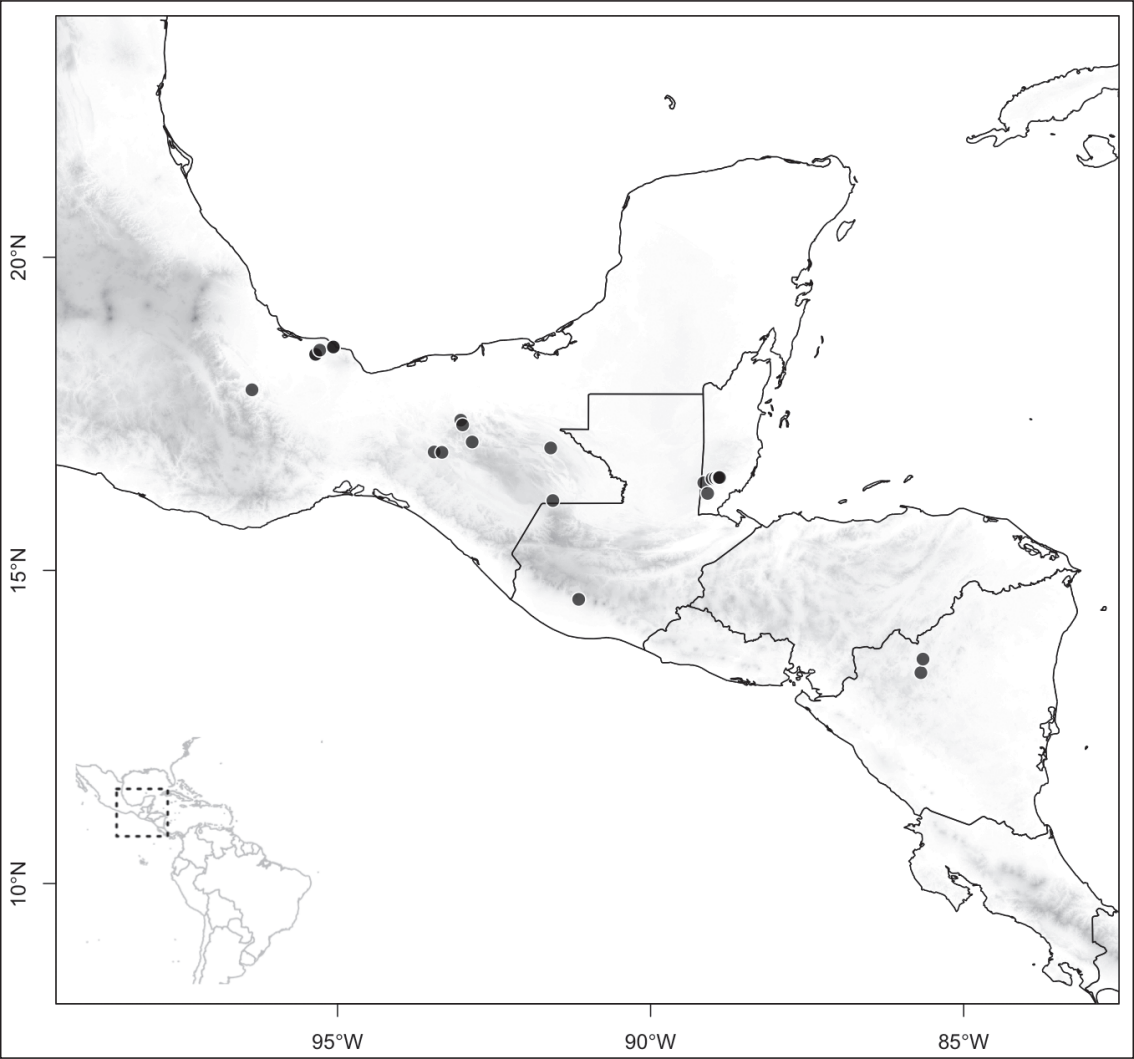
Distribution of *Conostegia fulvostellata*.

(Fig. 160). Mexico, Belize, Guatemala and Nicaragua, at 200–1700 m in elevation.

Almeda (2009) notes significant variation within *Conostegia fulvostellata*. In particular, populations from Veracruz, Mexico, have four merous flowers and glabrous petals. Specimens form the rest of its range have five merous flowers with papillose petals. More specimens are needed to better establish the differences noted by Almeda. Almeda (1989a) compared *Conostegia fulvostellata* in the protologue of *Miconia ibarrae* and noted differences in pubescence and notably in style curvature and seed characteristics. *Conostegia fulvostellata* has a dense indument in abaxial leaf surface reminiscent of *Conostegia oligocephala*. With the latter *Conostegia fulvostellata* also shares an overall geographic range. *Conostegia fulvostellata* differs from *Conostegia oligocephala* in its small calyx teeth, four locular ovary and usually papillose petals (vs. evident calyx teeth, five locular ovary and glabrous petals in *Conostegia oligocephala*).

#### Specimens examined.

**MEXICO. Chiapas**: 13km N of Berriozabal near Pozo Turipache and Finca El Suspiro, Breedlove 26340 (MO, NY); 18–20 km north of Ocozocoautla along the road to Mal Paso, Ocozocoautla de Espinosa, Breedlove 28141 (MO, NY). **Veracruz**: cerca de 2 km al S. de Tapalapan, Santiago Tuxtla, Beaman 6043 (MO, NY); Carretera Veracruz-Los Tuxtlas cerca al Cerro El Vigía, Pennington and Sarukhan 9121 (NY); Coatzacoalcos, isthmus of Tehuantepec, Smith 1003 (MO, NY).

### Conostegia galdamesiae

Taxon classificationPlantaeMyrtalesMelastomataceae

(Kriebel & Almeda) Kriebel comb. nov.

urn:lsid:ipni.org:names:77156257-1

Fig. 161

Conostegia galdamesiae (Kriebel & Almeda) Kriebel. Basionym: Miconia galdamesiae, Phytotaxa 134 (1): 28. 2013. Type: Panama. Chiriquí: Reserva Forestal de Fortuna, sendero atras de la estación del Smithsonian (STRI), 1162 m, 8.734583 N, -82.240083 W, 19 September 2011, R. Kriebel & J. Burke 5736 (holotype: NY!, isotypes: INB!, PMA!).

#### Description.

Small trees 2.5–7 m tall with young stems orange-brown from the copious indument of asperous-headed hairs, nodal line barely evident. Petioles 0.8–4 cm. Leaf blades 4.5–20 × 2–10.5 cm, 3–5-plinerved, diverging from the midvein 0.5–6 cm above the base usually asymmetrically, elliptic-ovate to ovate, base obtuse to acute and usually oblique, apex acuminate, the margin denticulate, adaxially glabrous except for asperous-headed hairs on the main veins towards the base, somewhat thin and dark green when alive, abaxially densely pubescent on tertiary and higher order veins with asperous-headed orange-brown hairs and glabrous to glabrescent on the actual surface. Inflorescences terminal, lax dichasia branched at or near the base of the inflorescence, 3.7–7 cm long, copiously covered with orange-brown asperous-headed hairs, bracts to 8 mm long, linear oblong, bracteoles 0.5–1 mm long, linear, less pubescent than rest of inflorescence rachis, drying pinkish, flowers clustered at the end of the inflorescence branches. Pedicels essentially absent. Hypanthia campanulate 1.25–1.75 × 1–1.5 mm, densely covered with asperous-headed hairs. Flowers 5-merous. Calyx fused in bud, shortly apiculate and less pubescent than the hypanthium, rupturing at anthesis into irregular, broadly rounded hyaline lobes 0.25–0.75 mm long and 0.5–0.75 mm wide at the base, the exterior calyx teeth 0.25–0.5 mm long, linear oblong, the calyx tube 0.25–0.5 mm long. Petals 1.5–2 × 1–1.5 mm, ovate, white, smooth, reflexed at anthesis, emarginate. Stamens 10, 3–3.5 mm long, radially arranged around the style; filaments 1.5–2 mm long, geniculate near the apex, translucent white; anthers 1–1.5 × 0.35–0.65 mm, linear-oblong, somewhat laterally compressed, cream yellow, pores 0.1–0.15 mm wide, truncate to somewhat ventrally inclined. Ovaries 5-locular, half inferior, apex elevated into a low papillose collar. Styles 4.5–4.75 mm long, straight to very slightly curved, distance between the anther apex and the stigma ca. 1 mm; stigmas truncate to capitellate, ca. 0.5 mm wide. Berries described as green-red on one label (McPherson 8410, CAS) but drying purple, 1.7–1.9 × 2.0–2.2 mm when dry; seeds ovoid and angled, 0.4–0.5 × 0.3–0.4 mm, orange-brown, lateral symmetrical plane ovate to triangular, the highest point toward the chalazal side, antiraphal symmetrical plane ovate-triangular and inconspicuously verruculose on the angles, raphal zone narrowly triangular and extending the length of the seed, expanded into an appendage that covers about 30% of the seed length. Chromosome number: n = 17 (reported as Miconia aff. friedmaniorum in Almeda (2013)).

**Figure 161.**
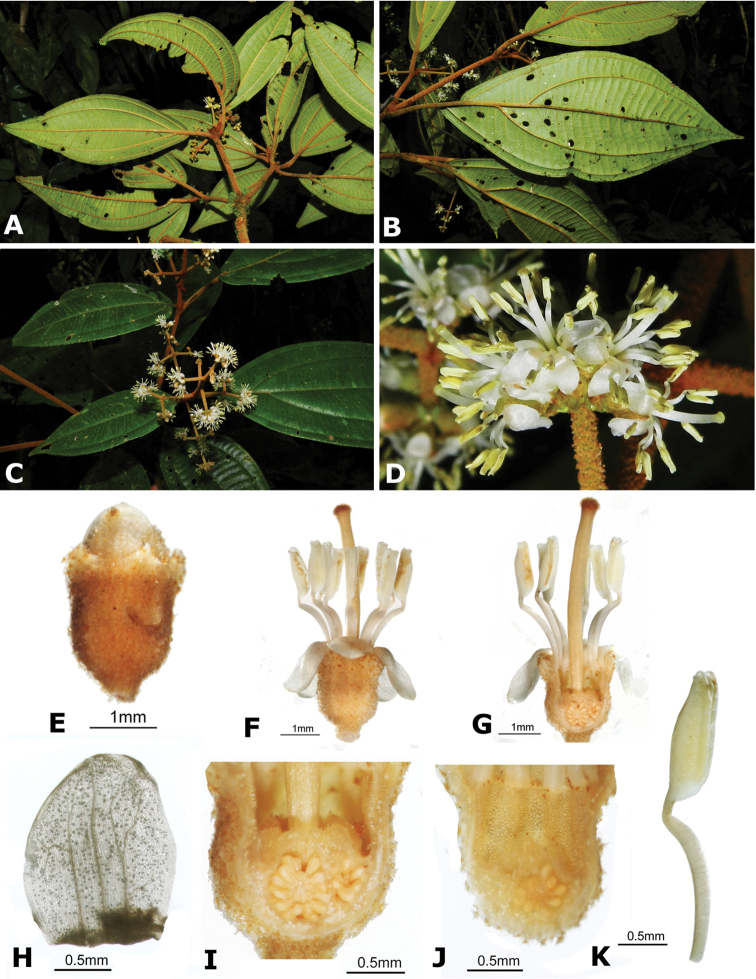
*Conostegia galdamesiae*. **A** Habit **B** Leaf abaxial surface. Note asymmetric leaf venation **C** Inflorescence **D** Close up of the flowers **E** Flower bud. Note closed sepals with apiculum **F** Pickled flower **G** Longitudinal section of a pickled flower **H** Petal **I** Close up of the longitudinal section of the flower showing the ovary apex **J** Close up of the longitudinal section of the flower showing the inner hypanthium wall **K** Stamen. Photos of specimen vouchered *R. Kriebel 5736*.

#### Distribution

**Figure 162.**
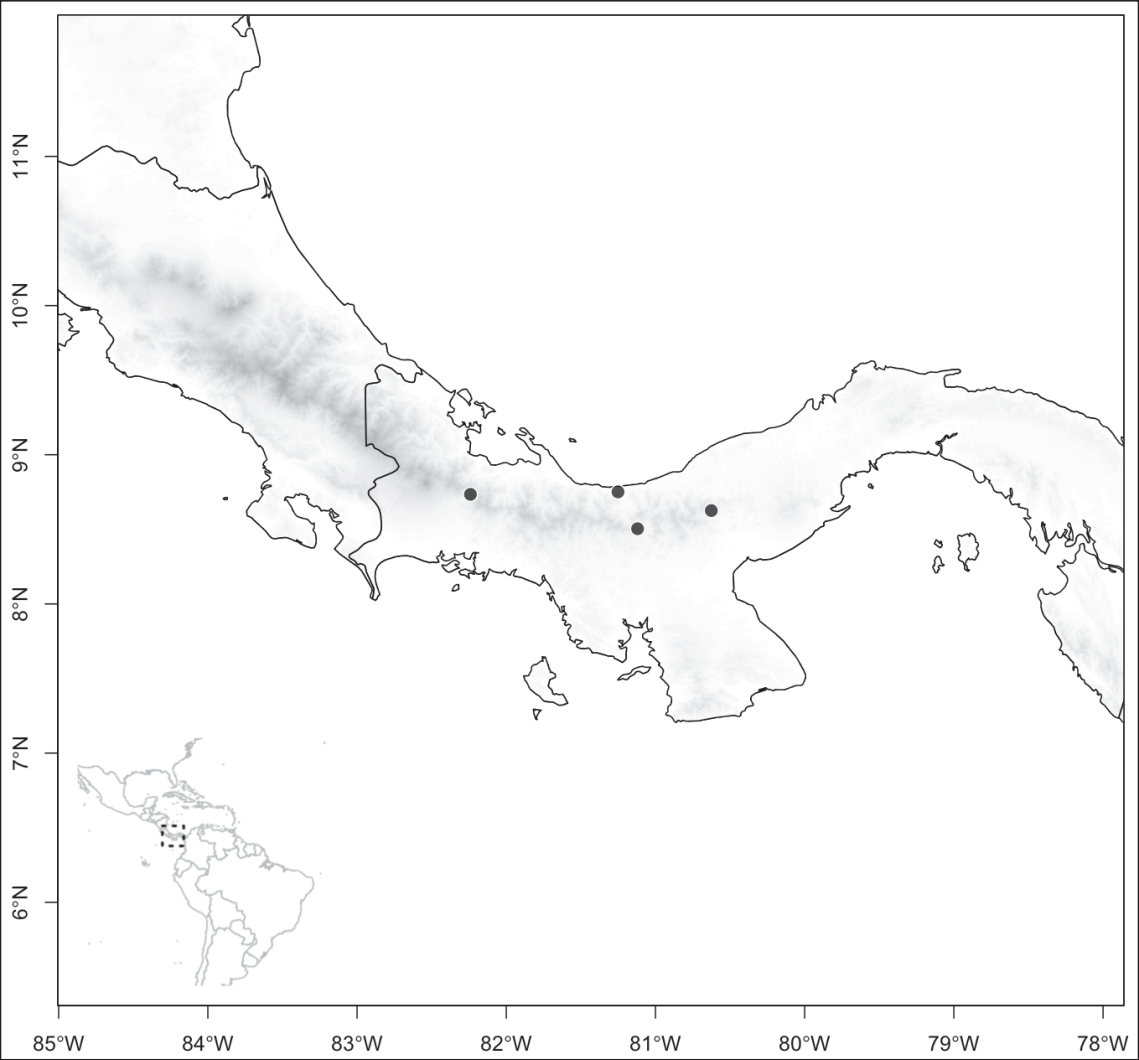
Distribution of *Conostegia galdamesiae*.

(Fig. 162). Endemic to cloud forests in the Panamanian provinces of Bocas del Toro, Coclé and Veraguas, 860–1350 m.

*Conostegia galdamesiae* is a Panamanian cloud forest endemic that can be recognized on the basis of its reddish indument, broad leaves with extremely plinerved leaf veins which are frequently asymmetric, and inflorescences with clustered, sessile flowers that have a fused calyx in bud. It is similar to *Conostegia brenesiana*, *Conostegia friedmaniroum*, and *Conostegia papillopetala*. From all of these species *Conostegia galdamesiae* can be recognized by its inflorescences with the flowers clustered at the end of the branches. From *Conostegia brenesiana* it can be further distinguished by its evident indument on branch apices and veins on the abaxial leaf surface. *Conostegia brenesiana* also has a unique broad anther pore. From *Conostegia friedmaniroum* it can be distinguished because the latter has deflexed inflorescences and linear-oblong petals. Lastly from *Conostegia papillopetala* it can be distinguished because the latter has narrower, somewhat bullate leaves, and papillose pink petals.

#### Specimens examined.

**PANAMA. Bocas del Toro**: Fortuna Dam Area, along continental divide trail bordering Chiriquí Province, Almeda et al. 6059 (BM, BR, CAS, GH, MEXU, MO, NY, PMA, P, US); Fortuna Dam region, near trail along continental divide, McPherson 8410 (CAS, MO, PMA); Vicinity of Fortuna Dam, along continental divide trail west of highway, McPherson 11636 (CAS, MO, PMA). **Coclé**: Cerro Tigrero, Mendieta 17–466 (CAS, PMA). **Veraguas**: Trail to Reserva Biológica Serrania de Tute and the summit of Cerro Tute about 0.7 km beyond the Escuela Agricola Rio Piedra just outside Santa Fe, Almeda et al. 7618 (CAS, INB, MO, NY, PMA, US); Vicinity of Escuela Agricola, Alto de Piedra near Santa Fe. 3 mi beyond the fork in the road near the school, toward Atlantic slope along trail to top of Cerro Tute, Antonio 3501 (CAS, MO, PMA); puente sobre rio Los Valles, cerca de la hidrolectrica Estrella Los Valles, Correa et al. 4341 (CAS, PMA); Cerro Tute ridge up from former Escuela Agricola, Santa Fe, Hamilton and Dressler 3073 (CAS, EAP, INB, MEXU, MO, PMA).

### Conostegia grayumii

Taxon classificationPlantaeMyrtalesMelastomataceae

(Almeda) Kriebel comb. nov.

urn:lsid:ipni.org:names:77156258-1

Fig. 163

Conostegia grayumii (Almeda) Kriebel. Miconia grayumii Almeda, Proc. Calif. Acad. Sci, series 4, 46(9): 209. 1989. Type: Costa Rica. Heredia: Finca La Selva, Field Station of the Organization for Tropical Studies on the Río Puerto Viejo on its junction with the Río Sarapiqui, elev. 100 m, 22 October 1982, T. McDowell 576 (holotype CAS!, isotype DUKE, MO!).

#### Description.

Shrub to small tree 1–5 m tall with subquadrate to terete stems which are sparsely to moderately ferrugineous scurfy-pulverulent to glabrous; the nodal line present but inconspicuous. Leaves of a pair somewhat unequal in size. Petioles 0.2–2.1 cm. Leaf blades 3.4–13 × 1.1–3.7 cm, 3-plinerved, with the inner pairs of subparallel veins arising up to 2 cm above the base opposite or generally alternate fashion, narrowly elliptic, the base broadly acute to obtuse and typically asymmetrical, the apex acuminate, the margin undulate denticulate to entire, glabrous above when mature, abaxially glabrous or scurfy pulverulent or glandular-pulverulent. Inflorescence a terminal and deflexed paniculiform cyme 2.4–5.7 cm long, sometimes becoming pseudolateral because of axillary bud elongation, accessory branches absent, rachis thin and glabrous, bracts and bracteoles to 3 mm long, lance-triangular to subulate, persistent and forming a nodal collar. Pedicel 2–2.5 mm. Flowers 5-merous, not calyptrate, nor the sepals fused in bud, floral buds ca. 1.5–2.8 × 1 mm long, the hypanthium 1–2 × 1–1.75 mm, globose, sparingly and deciduously scurfy-puverulent, the calyx lobes rounded-deltoid, hyaline and glabrous, ca. 0.5 mm long, exterior calyx teeth subulate, 0.25–0.5 mm long, torus glabrous. Petals 2–2.75 × 0.75–1.25 mm, white, obovate-oblong, spreading an anthesis, rounded to emarginated apically, papillose adaxially, otherwise glabrous. Stamens 10, 1.5–2.5 cm long, radially arranged around the style, filaments 1–1.5 mm, with a geniculation near the apex, white, anthers 0.75–1.25 × 0.25–0.5 mm, oblong, white to yellow, the pore ca. 0.1 mm wide, retuse to somewhat dorsally inclined. Ovary 5 locular, 3/4 inferior, glabrous. Style 3–4.5 mm long, gradually curving, vertical distance from the anther pore to the stigma 1.35–1.65 mm, stigma punctiform to truncate, ca. 0.29 mm wide. Berry 3–5 × 3–5 mm, purple black. Seeds 0.3–1 mm, angulate-pyramidate, smooth with verruculose angles.

**Figure 163.**
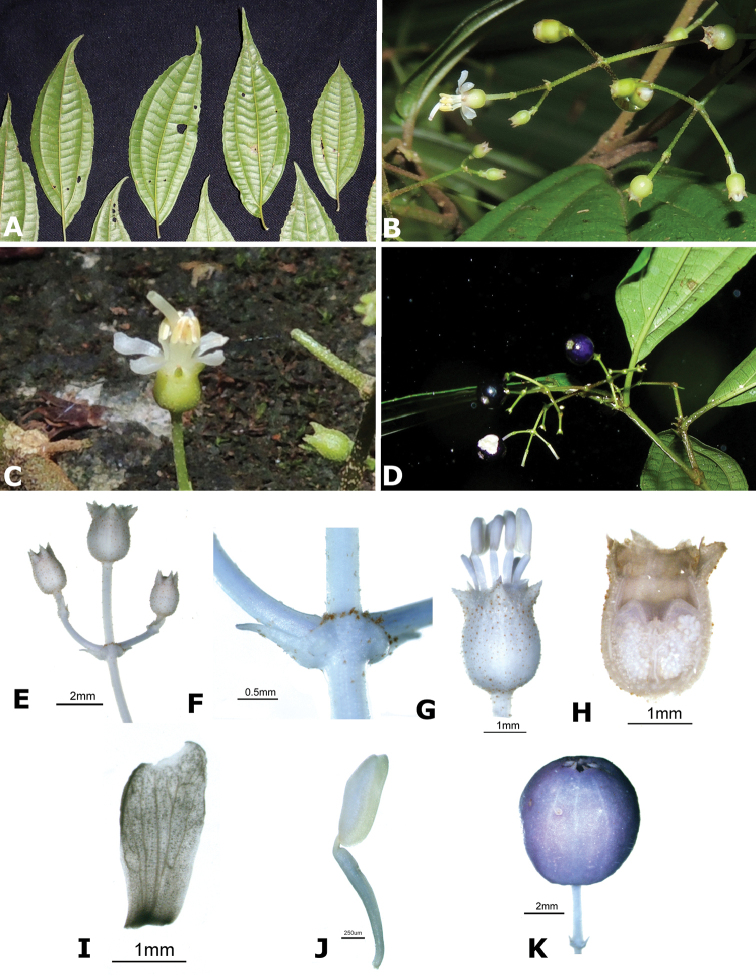
*Conostegia grayumii*. **A** Abaxial surface of several leaves. Note consistency of asymmetric venation **B** Inflorescence **C** Close up the flower **D** Infructescence **E** Apex of an inflorescence branch **F** Close up of bracteoles **G** Pickled flower at anthesis with the style removed **H** Longitudinal section of a flower with all parts removed **I** Petal **J** Stamen **K** Berry. Photos of specimen vouchered *R. Kriebel 5807*.

#### Distribution

**Figure 164.**
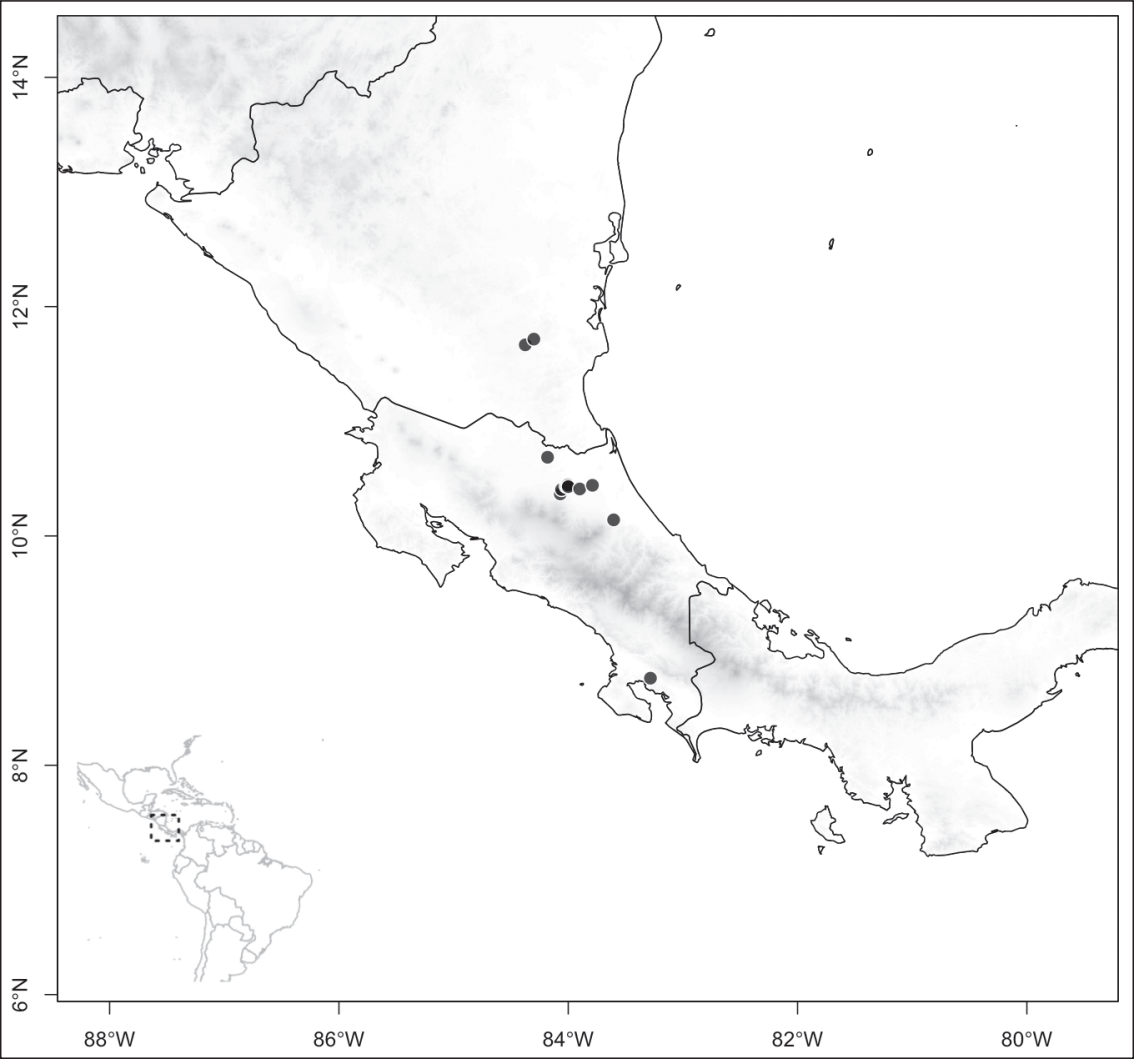
Distribution of *Conostegia grayumii*.

(Fig. 164). South eastern Nicaragua to the Caribbean lowlands of Costa Rica and one outlying population in the Osa Peninsula of Costa Rica on the Pacific side, 0–300 m in elevation.

*Conostegia grayumii* is a small tree restricted to lowland rain forests which can be recognized by its glabrosity, small plinerved and asymmetric leaves, and small flowers on wiry inflorescences. This species was identified before its recognition as a distinct taxon as *Conostegia brenesiana* (as *Miconia brenesii*). Almeda (1989b) noticed important differences between both such as the fact that *Conostegia brenesiana* has noticeably broader anther pores and lacks seed testa ornamentation present in *Conostegia grayumii*. Lastly, Almeda (1989b) noted the difference in habitat occupied by both taxa. *Conostegia grayumii* is restricted to the lowlands whereas *Conostegia brenesiana* is mostly restricted to middle elevation cloud forests. The molecular phylogeny shed further light on the relationships of these taxa. In fact, they did not form a sister species pair. *Conostegia grayumii* appears more closely related to *Conostegia cinnamomea*, *Conostegia dissitiflora*, *Conostegia fraterna*, and *Conostegia povedae* than to *Conostegia brenesiana*.

#### Specimens examined.

**COSTA RICA. Alajuela**: Laguna de Lagarto Lodge, sendero El Tucán, Solano and Hernández 1440 (INB, NY). **Heredia**: Finca La Selva, Puerto Viejo de Sarapiquí, Loop trail near SW trail, Grayum 1834 (NY); Chilamate, Hotel Selva Verde, Kriebel 2783 (INB); Finca La Selva, al lado de la Estación del Río, Kriebel 5222 (INB); Finca La Selva, the OTS Field Station on the Rio Puerto Viejo just E of its junction with the Río Sarapiquí, on bluff above the El Salto Stream at Rafael’s Point, Wilbur 66335 (NY). **Limón**: Pococí, Estacion Biológica La Suerte, Primavera, Kriebel 1605 (INB, MO); Siquirres, alrededores de la finca de Erick Berlin, Santamaría and González 4584 (INB, NY). **Puntarenas**: P.N. Piedras Blancas, Serranías de Golfito, Sendero frente los Altos, Fletes 641 (INB, MO).

### Conostegia hammelii

Taxon classificationPlantaeMyrtalesMelastomataceae

(Almeda) Kriebel comb. nov.

urn:lsid:ipni.org:names:77156259-1

Fig. 165

Conostegia hammelii (Almeda) Kriebel. Basionym: Clidemia hammelii, Proc. Calif. Acad. Sci, ser. 4, 46(5): 140. 1989. Type: Costa Rica. Heredia: Finca La Selva. OTS (Organization for Tropical Studies) Field Station on Río Puerto Viejo just E of its junction with Río Sarapiquí, slopes along Q. El Salto, 2900 m, elev. about 100 m, 2 September 1980, B. Hammel 9682 (holotype: CAS!, isotype: DUKE, MICH!, MO!).

#### Description.

Shrub 1.5–3.25 m tall with slightly tetragonal branches at the apex that have a sparse to dense covering of smooth spreading hairs (2–3.5 mm) intermixed with inconspicuous, early deciduous, asperous headed hairs that are underlain by a moderate to dense understory of stellulate-furfuraceous or short asperous headed hairs but becoming glabrate at maturity; the nodal line absent. Leaves at each node equal to somewhat unequal in size. Petiole 0.5–4 cm. Leaf blade 7.4–29 × 3.7–14.5 cm, 5–7 nerved or if 5–7 plinerved, with the innermost pair of primary veins diverging from the midvein up to 2 cm above the base in opposite or alternate fashion, elliptic, the apex long acuminate, the margin entire to crenulate, adaxially moderately strigose to subhirsute with hairs mostly 1–2 mm long, abaxially moderately hirsute with a mixture of smooth hairs (1–2.5 mm long) and minute glandular hairs essentially restricted to the primary and higher order veins and with pocket mite domatia at the base. Inflorescence a pseudolateral modified dichasium 2.1–6 cm long divaricately branched at the base and appearing axillary on older nodes, accessory branches absent, bracteoles 1.5–3.5 × 0.5 mm, paired at a node and persistent, sometimes fused forming a nodal collar, lanceolate to subulate. Pedicels 1–2.5 mm long. Flower buds 2–2.5 × 1.9–2.4 mm. Flowers 5 merous, calyx not calyptrate but closed in bud and crowned by an apiculum 0.5 mm long and rupturing irregularly at anthesis into 3–5 hyaline, persistent lobes 0.5 mm long, 2–2.5 × 1.9–2.4 mm, ca. 0.25 mm, external calyx teeth triangular-subulate, 0.5–1 mm long, the hypanthium campanulate, 0.5–2 mm long, moderately to sparsely covered with spreading smooth hairs 0.5–2 mm long and a sparse understory of sessile stellulate furfuraceous hairs, the torus not evidently glandular puberulent but the inner hypanthium wall sparsely so. Petals 3.5–4 × 1.5–2 mm, white or translucent white, oblong, rounded apically, spreading at anthesis. Stamens 10, 2.5–4 mm long, radially arranged around the style, the filament 1.5–2.5 mm with a geniculation near the apex, translucent white, anthers 1–1.75 × 0.35–0.85 mm, oblong and laterally compressed, yellow, the pore 0.15 mm wide, somewhat dorsally inclined. Ovary 5 locular, totally inferior, apex fluted and elevated into a glandular-puberulent lobulate collar. Style 5–6 mm long, straight to slightly bending, vertical distance from the anther to the stigma 1.25–1.75 mm, horizontal distance absent; stigma truncate, 0.3–0.5 mm wide. Berry 6–7 × 5–6 mm, purple-black. Seeds 0.5–0.7, galeiform to deltoid, irregularly angulate with a densely papillate testa and a lateral flattened or somewhat convex raphe.

**Figure 165.**
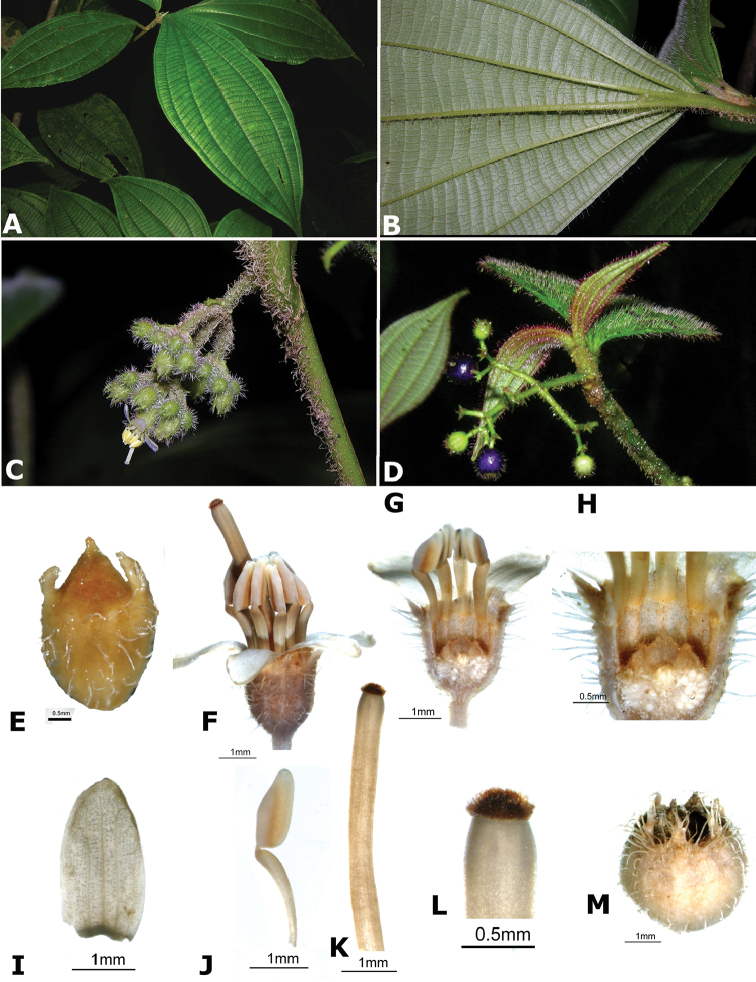
*Conostegia hammelii*. **A** Leaf adaxial surface **B** Leaf base abaxial surface. Note pocket domatia **C** Inflorescence **D** Infructescence **E** Flower bud showing fused calyx with apiculum **F** Pickled flower **G** Longitudinal section of a flower with the style removed **H** Ovary apex **I** Petal **J** Stamen **K** Style **L** Stigma **M** Immature berry. Photos **A–C** of specimen vouchered *R. Kriebel 5539* **D** of specimen vouchered *R. Kriebel 5317* **E–M** vouchered *R. Kriebel 5737*.

#### Distribution

**Figure 166.**
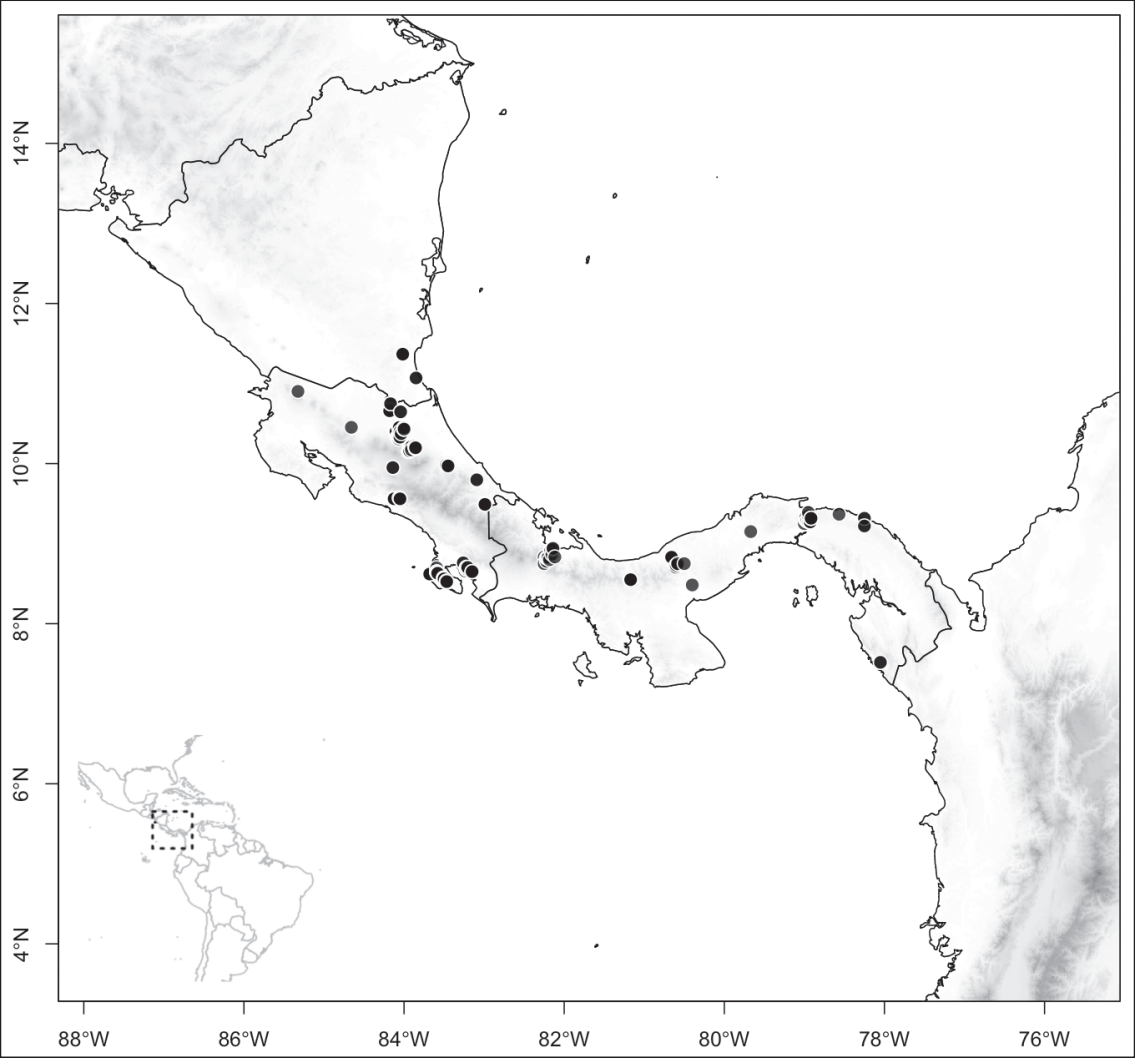
Distribution of *Conostegia hammelii*.

(Fig. 166). Nicaragua, Costa Rica, Panama and reaching Colombia, 0–950 m in elevation.

*Conostegia hammelii* is easy to distinguish by the presence of pocket domatia on the base of the abaxial leaf surface, its pseudolateral inflorescences branched at the base, sepals that are fused in bud into an apiculum, ovary apex with a glandular-puberulent lobulate collar and papillose seed testa. Almeda (1989a) noted that some specimens have stamens that are somewhat unequal in length. This phenomenon is only present in *Conostegia dissitiflora* within *Conostegia*. I have not noted this staminal length difference in my collections in spirit from Costa Rica and Panama of this species. In the molecular phylogeny, *Clidemia hammelii* forms a relatively well supported clade with *Conostegia allenii*, *Conostegia ecuadorensis*, and *Conostegia ombrophila*. This clade shares the pseudolateral inflorescences as well as the papillose seed testa. It is also noteworthy that three species of this clade, namely *Conostegia ecuadorensis*, *Clidemia hammelii*, and *Conostegia ombrophila* are the only ones in section *Geniculatae* to have domatia. Almeda (1989a) reported on the presence of mites of the genus *Ololaelaps* found in the domatia of this species.

#### Specimens examined.

**COSTARICA. Alajuela**: Boca Tapada, Laguna del Lagarto Lodge, Solano and Santamaría 1709 (INB, NY). **Limón**: P.N. Braulio Carrillo, Sector Quebrada Gonzalez, sendero Las Palmas, Kriebel 1420, 5317 (INB, NY); R.B. Barbilla, Sendero Valle Escondido, Mora 440 (INB, NY). **Puntarenas**: Golfito, Esquinas, Estacion Biológica La Gamba, Kriebel 1018 (INB).

**PANAMA. Bocas del Toro**: above Chiriquí Grande on a side road about 10 road miles below the Continental Divide about 2.5 miles east on that road, Almeda et al. 6332 (CAS, NY); 15 km mas alla de la division continental en la calle hacia Chiriqui Grande, despues del poblado de Mali 4 km hacia el este, Kriebel and Burke 5737 (NY, PMA). **Coclé**: area between La Junta and Limón, 5 hours walk north of Alto Calvario, Folsom 5864 (MO, NY). **Colón**: 9 miles south of Portobello, Croat 11372 (NY). **San Blás**: El Llano Carti Road, 17.5 km from Interamerican Highway, de Nevers and Herrera 4009 (MO, NY).

### Conostegia henripittieri

Taxon classificationPlantaeMyrtalesMelastomataceae

Kriebel nom. nov.

urn:lsid:ipni.org:names:77156260-1

Fig. 167

Conostegia henripittieri Kriebel.Based on: Clidemia pittieri Gleason, Bull. Torrey Bot. Club 68: 252. 1941. Type: Panama. Chiriquí: Humid forest around Los Siguas Camp, southern slope of Cerro de la Horqueta, alt. about 1700 m, 17–19 March 1911, H. Pittier 3177 (holotype: NY!, isotype: F!, NY!, US!).

#### Description.

Shrubs 0.5–2.5 m tall (with one report to 8 m) with slightly tetragonal, ridged and glabrous stems; the nodal line nodal line present but inconspicuous. Leaves of a pair very unequal in size. Petiole 0.1–0.2 cm long. Leaf blades 2.5–16.1 × 2–7.8 cm, 3–5 nerved, ovate-lanceolate to oblong-ovate, the base cordate, the apex acuminate, the margin entire, glabrous on both surfaces. Inflorescence a pseudolateral laxly branched and few-flowered pseudolateral dichasium 4.7–6.5 cm long, branching above the base, accessory branches absent, the rachis glabrous, the branches slender, bracteoles 0.75–2 × 0.1–0.75 mm, elliptic to oblong or narrowly oblong-obovate, persistent and forming a low nodal collar. Pedicels 1–2 mm. Flowers 5 or 6 merous, not calyptrate, nor sepals fused in bud. Floral buds ca. 3–4 × 1.5–2 mm, hypanthium campanulate to urceolate, glabrous, calyx lobes depressed triangular to rounded-undulate, 0.6–0.75 × 0.75 mm, calyx teeth broadly triangular, 0.5 × 0.75 mm. Petals 3–4 × 3–4 mm, pink, broadly obovate to subrotund, rounded to emarginated apically, glabrous. Stamens 10 or 12, 2.25–3 mm long, the filament 1.25–2, apparently lacking a geniculation near the apex, anthers 1 × 0.6–0.8 mm, yellow, laterally compressed, connective thickened, the pore 0.18–0.2 mm wide, terminal and somewhat dorsally inclined. Ovary 5–6 locular, inferior, apically glabrous and flat, with five or six callose thickenings around the style base. Style not seen. Berry 4–6 × 4–6 mm, purple black. Seeds 0.45–0.6 mm long, triangular in sideview, the testa angulate and smooth.

**Figure 167.**
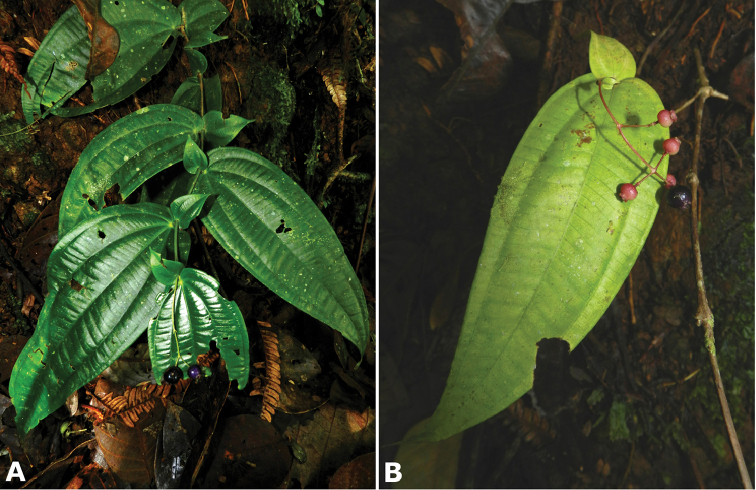
*Conostegia henripittieri*. **A** Habit **B** Infructescence. Photos of specimen vouchered *R. Kriebel and J. Burke 5757*.

#### Distribution

**Figure 168.**
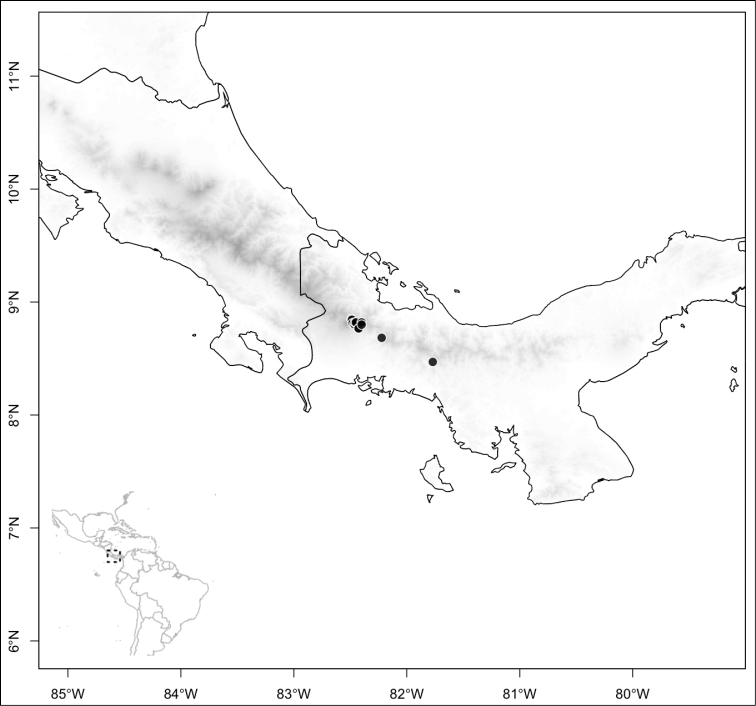
Distribution of *Conostegia henripittieri*.

(Fig. 168). Endemic to western Panama at 1300–2000 m.

The epithet *henripittieri* was chosen for this species because *pittieri* is preempted by *Conostegia pittieri* Cogn. ex T. Durand. *Conostegia henripitteri* is unmistakeable because of its glabrous, subsessile, anisophyllous leaves. Although I was not able to see flowering material of this species, the isotype at F shows the evidently exserted style that is almost universal in section *Geniculatae*.

#### Specimens examined.

**PANAMA. Chiriquí**: disturbed cloud forest at Monte Rey above Boquete, Croat 15658, 15693 (NY); above Boquete near Parque Nacional Volcán Barú, along Rio Caldera in Alto Chiquero, Penneys and Olmos 1740 (FLAS, NY); Boquete, Finca Lérida, Quiroz 800 (NY); Finca Lérida to Peña Blanca, Woodson and Schery 289 (NY); Cerro Horqueta, von Hagen and von Hagen 2039 (NY); vicinity of Bajo Mona and Quebrada Chiquero, Woodson and Schery 562 (NY).

### Conostegia incurva

Taxon classificationPlantaeMyrtalesMelastomataceae

(Gleason) Kriebel comb. nov.

urn:lsid:ipni.org:names:77156261-1

Fig. 169

Conostegia incurva (Gleason) Kriebe. Basionym: Miconia incurva Gleason, Bull. Torrey Bot. Club 65: 580. 1938. Type: Costa Rica. Vara Blanca de Sarapiquí, north of Central Cordillera, alt. 1500–1750 m, July-Sept 1937, A. Skutch 3273 (holotype: NY!, isotypes: MO!, US!).
Miconia austin-smithii Standl. and L.O. Williams, Brittonia 15(1): 25–26, f. 1. 1963. Type: Costa Rica. Alajuela: 5 km s of Zarcero, cantón de Alfaro Ruíz, 17 June 1941, A. Smith 2804 (holotype: F!).

#### Description.

Trees 3–10 m tall with rounded-quadrate stems that are densely covered with a ferrugineous stellate indument; the nodal line not evident from the consipucous indument. Leaves of a pair equal to unequal in length. Petiole 0.5–2.8 cm. Leaves of a pair equal to somewhat unequal in length; leaf blade 4.3–18 × 1.9–5.9 cm, 5-plinerved, with the innermost pair of veins diverging from the midvein ca. 1–2 cm above the base in opposite or alternate fashion, elliptic to oblong-elliptic, the base acute to obtuse, the apex acuminate, the margin entire, the adaxial surface glabrous, the abaxial surface covered with a ferrugineous stellate indument. Inflorescence a terminal paniculiform dichasium, 2.5–7 cm long, accessory branches absent, rachis densely covered with a ferrugineous stellate indument; bracts and bracteoles triangular subulate, 1.5–3 × 0.5–1 mm, persistent. Flowers 5-merous, sessile or subsessile at anthesis, not calyptrate, nor the sepals fused in bud, floral buds ca. 8–9 mm, the calyx truncate and forming a flange ca. 1.5 mm long, exterior calyx teeth conspicuous, linear and more or less hooked, 2–7 mm long, torus glabrous. Petals 0.6–1.1 × 0.6–0.8 mm, white, broadly obovate, asymmetrical apparently spreading an anthesis, glabrous, emargiante apically. Stamens 10, 6.5–7 mm long, radially arranged around the style, the filament 3–3.25 mm, with a geniculation near the apex of the filament, white, anthers 3.25–3.75 × 0.75–1 mm, oblong, yellow, laterally compressed, the connective thickened, the pore ca. 0.15 mm wide, ventrally inclined. Ovary 5 locular, 4/5 inferior, glabrous and elevated into a collar around the style base. Style 8–8.5 mm long, vertical distance from the anther pore to the stigma ca. 1–2 mm, horizontal distance absent, the stigma capitate, ca. 1 mm wide. Berry 6–10 × 6–10 mm, reportedly light red. Seeds ca. 0.5 mm long, triangular to angulate-ovoid, the testa muriculate.

**Figure 169.**
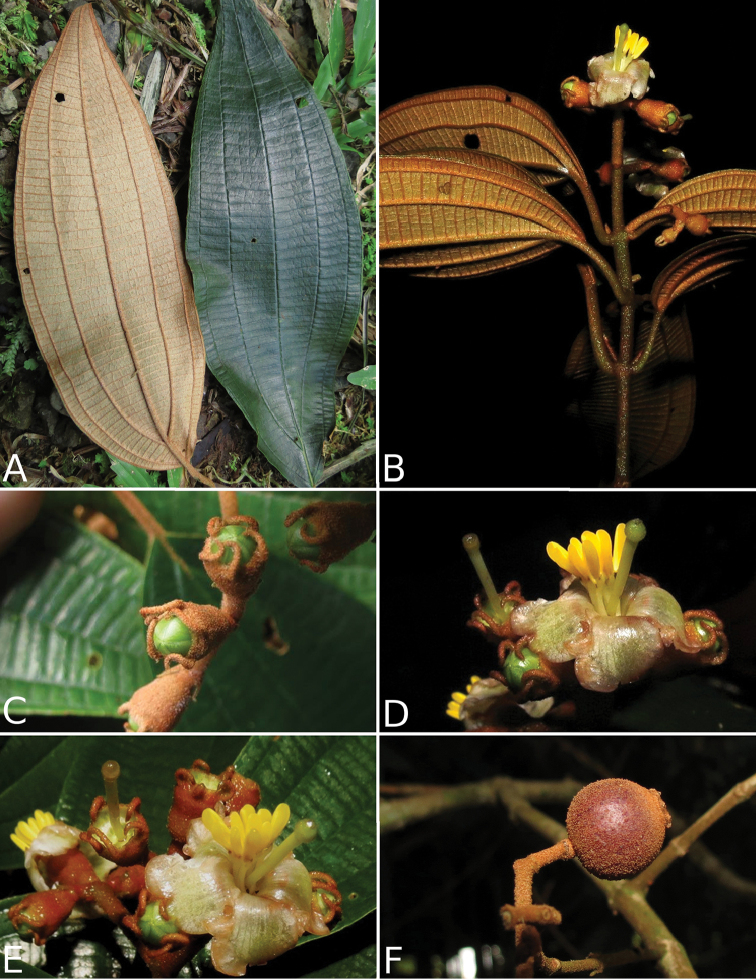
*Conostegia incurva*. **A** Abaxial and adaxial leaf surfaces **B** Inflorescence **C** Flower buds **D** Close up of flower **E** Close up of flowers **F** Fruit. Photographs by Ronny Josué Morales Mesén.

#### Distribution

**Figure 170.**
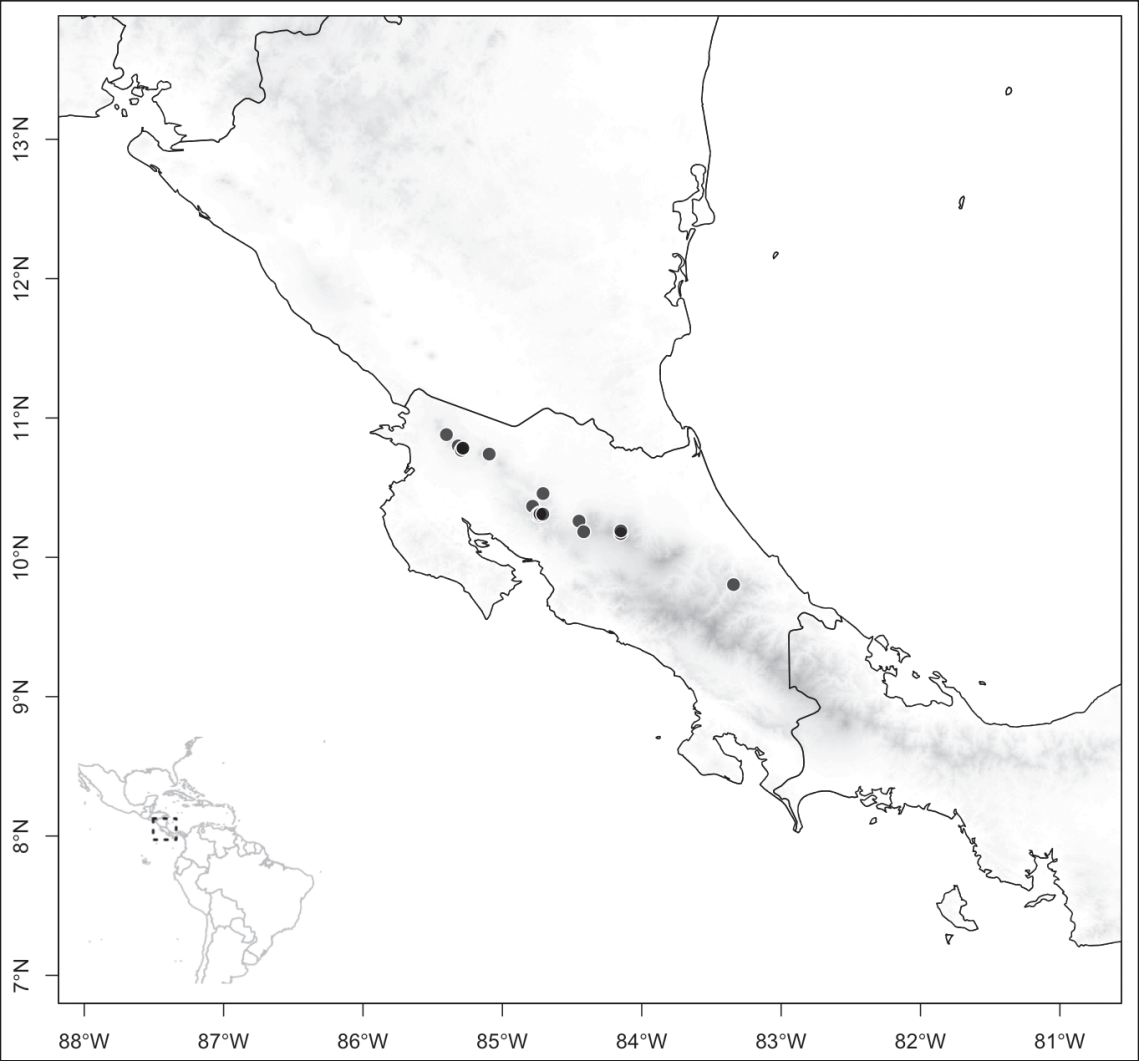
Distribution of *Conostegia incurva*.

(Fig. 170). Endemic to the Pacific slope of the Tilarán, Central and Talamanca Cordilleras in Costa Rica, 500–1500 in elevation.

*Conostegia incurva* is a rare species that can be recognized by the ferrugineous indument that covers the abaxial leaf surface, the strongly plinerved leaves with frequently asymmetrical primary veins, and large flowers with linear and evident calyx teeth. It resembles *Conostegia schlimii*, but the latter lacks the linear calyx teeth. Interestingly, *Conostegia incurva* forms a strongly supported species pair with the northern Central American endemic *Conostegia oligocephala* in the molecular phylogeny. The latter has much smaller flowers and more whitish indument on the leaves abaxially. The specimen *Herrera 627* (CAS, CR) mentions the style separate from the stamens like most other species in section *Geniculatae*.

#### Specimens examined.

**COSTA RICA. Alajuela**: along dirt road that turns into Finca Los Ensayos off Highway 15 ca. 7.5 miles N of Zarcero, Croat 43463 (CAS); Upala, C.B. Guanacaste-Rincón de la Vieja, Sector la Campana, Sendero sobre margen del Río Colón, Espinoza 1566 (INB, MO); Río Peñas Blancas, Finca Novo, Haber and Bello 7346 (CAS, INB); Z.P. Miravalles, 3 km West of Bijagua, Ridges above Rio Zapote, Penneys and Haber 615 (INB, MO); San Luis de Zarcero, Smith 625 (CR, NY); Cordillera Central, near San Juan de Laja about 15 km north of Zarcero, Williams et al. 29000 (NY). **Guanacaste**: P. N. Rincón de la Vieja, siguiendo el canal desde la Hacienda Santa María hasta Quebrada Provisión, Herrera 627 (CAS, CR). Heredia: Montaña Azul de Varablanca, Jiménez 3444 (CR, NY). **Limón**: Cordillera de Talamanca, headwaters of Quebrada Kakebeta, below divide between Río Xikiari and Río Boyei, Grayum 10955 (CAS, CR, INB).

### Conostegia iteophylla

Taxon classificationPlantaeMyrtalesMelastomataceae

(Almeda) Kriebel comb. nov.

urn:lsid:ipni.org:names:77156262-1

Conostegia iteophylla (Almeda) Kriebel. Basionym: Miconia iteophylla Almeda, Proc. Calif. Acad. Sci. 46(9): 214. 1989. Type: Panama. Coclé: along Río San Juan below its junction with Río Tife, elev. 1200 ft (366 m), 11 June 1978, B. Hammel 3393 (holotype: CAS!, isotype: MO!).

#### Description.

Shrubs 0.4–1.5 m tall with sub quadrate to terete branches moderately covered with a brown scurfy-pulverulent indument; the nodal line nodal line present. Leaves of a pair equal to somewhat unequal in size. Petiole 2.5–1.1 cm. Leaf blade 1.7–9.5 × 0.4–1.7 cm, 3-plinerved, with the innermost pair of veins diverging from the midvein 0.2–0.7 mm above the base in opposite or sub opposite fashion, narrowly elliptic, the base acute to attenuate, the apex acuminate, the margin entire, deciduously pulverulent adaxially when young but soon becoming glabrous, the abaxial surface deciduously scurfy pulverulent on the veins. Inflorescence a terminal paniculate cyme 2–7 cm long, branching above the base, accessory branches absent, rachis brown scurfy, bracteoles 0.5–3.5 × 0.25–0.5 mm, linear-oblong to triangular, sessile and persistent, paired and fused laterally into a short nodal collar. Pedicel 1–2 mm. Flowers 5-merous, calyx in bud not calyptrate nor fused in bud, hypanthium 1–1.5 mm, campanulate, deciduously scurfy pulverulent, calyx tube 0.25 mm long, the lobes barely discernible as triangular undulations, calyx teeth subulate ca. 0.25 mm long. Petals 2–2.5 × 0.6–0.8 mm, white, elliptic oblong, their posture not observed at anthesis, glabrous and entire, narrowly rounded to acute apically. Stamens 10, 2.5–3 mm long, apparently radially arranged around the style, the filament 1.25–1.5 mm, the geniculation near the apex of the filament apparently present, white, anthers 1.25–1.5 × 0.25–0.5 mm, linear-oblong, yellow, laterally compressed, the connective thickened dorsally, the pore ca. 0.1 mm wide, dorsally inclined. Ovary 5 locular, 2/3 inferior, the apex fluted and glandular puberulent. Style 3.5–4 mm long, distance from the anther pore to the stigma ca. 1 mm, the stigma capitate, ca. 0.3 mm wide. Berry 2–4 × 3–4 mm, red turning purple black. Seeds ca. 0.5 mm long, irregularly triangular to angulate-ovoid, the testa smooth and polished on the convex face.

#### Distribution.

**Figure 171.**
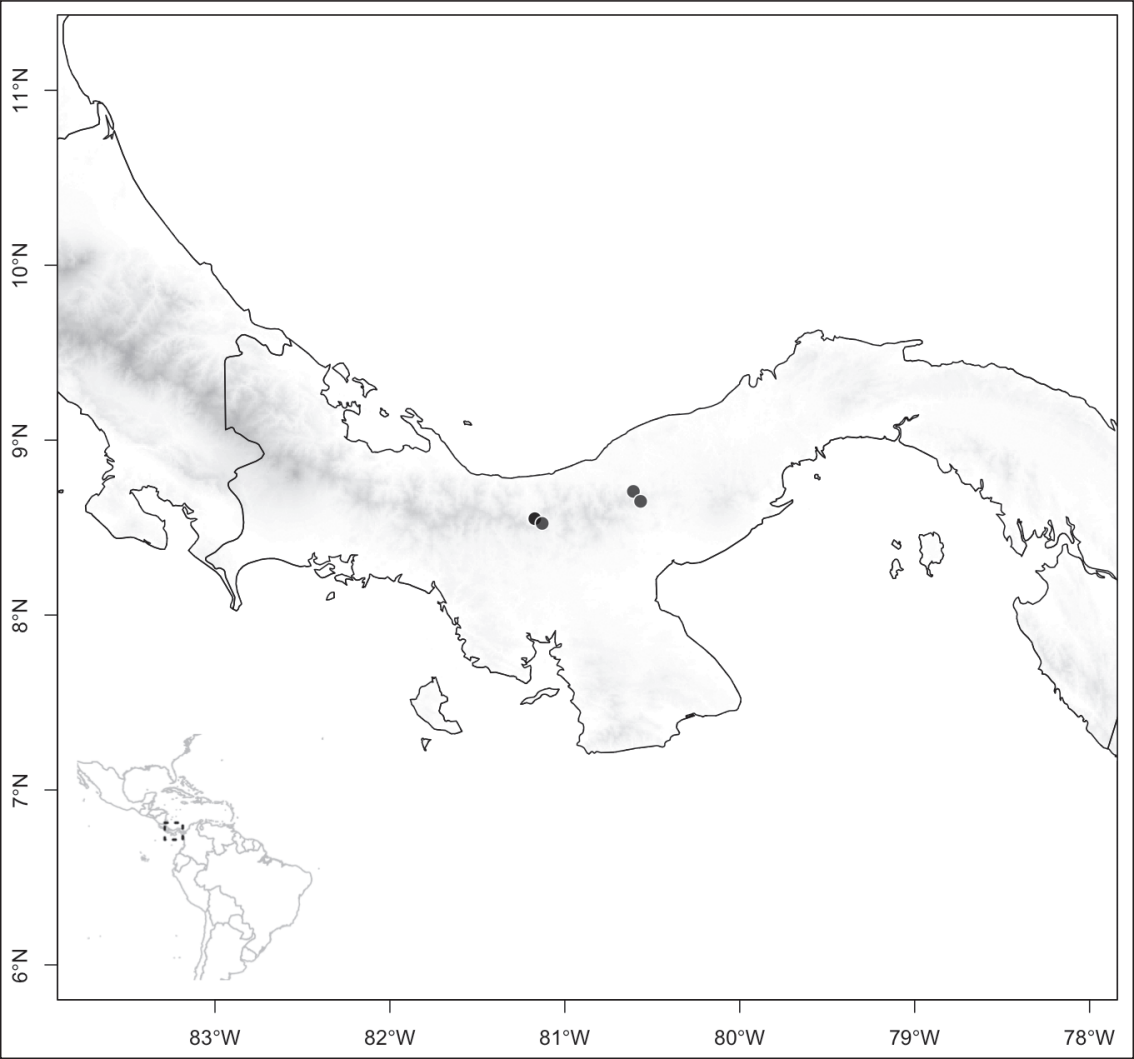
Distribution of *Conostegia iteophylla*.

Endemic to Panama, where it grows near moving water (fide Almeda), 200–700 m. Fig. 171.

When Almeda (1989a) described *Miconia iteophylla* he noted the strong similarity between it and *Miconia ligulata* (here combined to *Conostegia consimilis*). Indeed it appears to be a narrow leaf *Conostegia consimilis*. Unfortunately I have not been able to place *Miconia iteophylla* in the molecular phylogeny and test this possible close relationship. A line drawing of this species is available in the protologue (Almeda 1989a). For further dicussion of the differences between *Conostegia iteophylla* and *Conostegia consimilis* see the discussion under the latter.

#### Specimens examined.

**PANAMA. Bocas del Toro** (fide Almeda): upper Río San Pedro, Gordon 59Db (MO). **Coclé**: trail from Caño Sucio to Cerro Tife on the Atlanti slope, Antonio 3669 (CAS); Caribbean side of divide at El Copé, Hamilton and Davidse 2628 (CAS).

### Conostegia jefensis

Taxon classificationPlantaeMyrtalesMelastomataceae

(Almeda) Kriebel comb. nov.

urn:lsid:ipni.org:names:77156263-1

Fig. 172

Conostegia jefensis (Almeda) Kriebel. Basionym: Miconia jefensis Almeda, Proc. Calif. Acad. Sci. 52(4): 43. 2000. Type: Panama. Cerro Jefe, along summit road and along trail into the Chagres Valley, ca. 900 m, 19 February 1988, F. Almeda, Daniel, T. & G. McPherson 5826 (holotype: CAS!; isotypes: MO!, NY!, PMA!).

#### Description.

Small tree 2–4 m tall with somewhat tetragonal and ridged stems in newer branches that are moderately to densely brown scurfy with stalked stellulate hairs; the nodal line present but inconspicuous. Petiole 0.3–4 cm. Leaf blade 17–43 × 8–14 cm, 5(-7) plinerved, with the innermost pair of veins usually diverging from the midvein 2.5–6 cm above the base in opposite to alternate fashion, elliptic, the base gradually tapering to decurrent on the petiole or less commonly rounded to slightly cordate, the apex acuminate to long acuminate, the margin entire to undulate, the adaxial surface sparsely scurfy-pulverulent to glabrous, the abaxial surface moderately and deciduously scurfy-pulverulent on the secondary and higher order veins. Inflorescence a terminal panicle 4.4–11.2 cm long branched at the base, accessory branches absent, brown scurfy on the branches, subulate, bracteoles 0.3–0.75 × 0.2–0.5 mm, persistent, fused and forming a nodal collar or ridge. Pedicel 0.5–2 mm. 1.25–1.75 × 1.25–1.75 mm, hypanthium campanulate, not constricted, deciduously scurfy-pulverulent. Flowers 5-merous, not calyptrate, flower buds ca. 0.3–0.5 × 0.1–0.2 mm, calyx tube 0.2–0.3 mm long, calyx lobes depressed-triangular to undulate, 0.5 mm long but concealed and barely exceeded by the exterior subulate teeth, androecial fringe present and beset with glandular hairs. Petals 2.75–3.25 × 0.75–1 mm, magenta, linear-oblong, spreading an anthesis, the apex acute, glabrous, spreading an anthesis, the margin entire. Stamens 10, 2.25–2.75 mm, radially arranged around the style, the filaments 1.25–1.5 mm long, with a geniculation near the apex, white, anthers 1–1.25 0.25–0.4 mm, oblong, yellow, the pore ca. 0.1 mm wide, somewhat dorsally inclined. Ovary 5 locular, 3/4 to 4/5 inferior, glabrous apically. Style 3.5–4.25 mm long, curving towards the apex, distance from the anther pore to the stigma 1–1.5 mm, stigma truncate, ca. 0.35 mm wide. Berry 3–4 × 3–4 mm, purple-black. Seeds 0.35–0.5 mm, pyramidate, smooth with verruculose angles.

**Figure 172.**
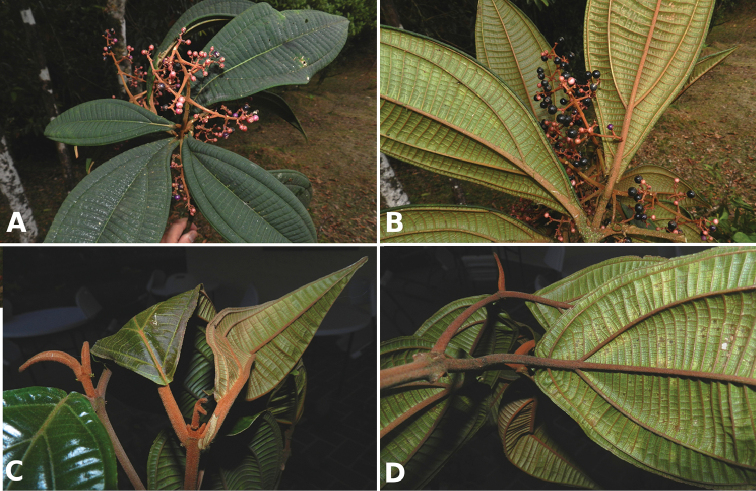
*Conostegia jefensis*. **A** Branch with infructescence **B** Abaxial leaf surface **C** Apex of a branch. Note orange pubescence **D** Base of the abaxial leaf surface **A–B** from specimen vouchered *R. Kriebel and J. Burke 5659* **C–D** from specimen vouchered *R. Kriebel and J. Burke 5680*.

#### Distribution

**Figure 173.**
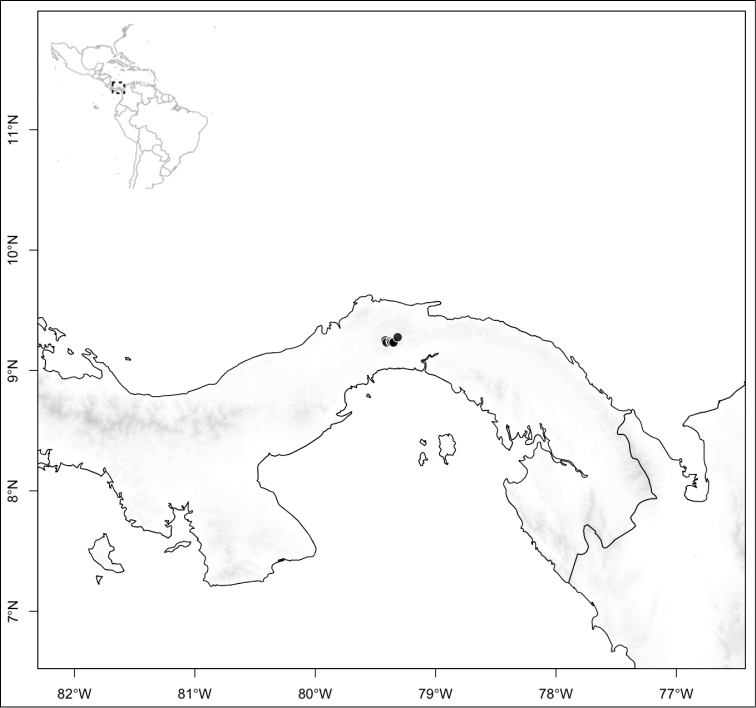
Distribution of *Conostegia jefensis*.

(Fig. 173). Endemic to Cerro Jefe in Panama from 750–1000 m elevation.

*Conostegia jefensis* can be recognized by its reddish scurfy pubescence, strongly plinerved leaves and magenta petals. This is a puzzling species because it includes two morphotypes endemic to Cerro Jefe. The common morphotype resembles *Conostegia consimilis* in the decurrent leaf base but differs from it in its coarse habit, somewhat coriaceous leaves and magenta petals. The second morphotype which corresponds to the morphology of the holotype is apparently much less common and does not have the decurrent leaf base but rather a rounded to cordate leaf base. This morphotype also has the typical magenta petals of the other morphotype. Unexpectedly, the two morphotypes did not form a clade in the molecular phylogeny. The holotype matching morphotype formed a strong sister relationship to another Cerro Jefe endemic, *Conostegia peltata*. Lack of resolution and more samples of each morphotype are needed at this time to further confirm the possibility that they represent different taxa.

#### Specimens examined.

**PANAMA. Panamá**: Una milla después de la Eneida, Región de Cerro Jefe, Correa and Dressler 946 (MO, NY, PMA); P. N. Chagres, sendero hacia Cerro Jefe que sale de la urbanización Altos de Cerro Azul, Kriebel and Burke 5659, 5669 (PMA, NY).

### Conostegia oligocephala

Taxon classificationPlantaeMyrtalesMelastomataceae

(Donn. Sm.) Kriebel comb. nov.

urn:lsid:ipni.org:names:77156264-1

Fig. 174

Conostegia oligocephala (Donn. Sm.) Kriebel. Basionym: Miconia oligocephala Donn. Sm., Bot. Gaz. 46: 111. 1908. Lectotype (designated here): Guatemala: Alta Verapaz: Cobán, alt. 1550 m (US), von Tuerckheim II-1781 (US!). Additional syntype: H. von Tuerckheim 8686 (US).

#### Description.

Shrubs to small trees 2–12 m tall with somewhat flattened cauline internodes that are densely covered with a whitish and ferrugineaous stellate indumentum; the nodal line not evident from the indument. Leaves of a pair equal to unequal in length.Petioles 0.5–3 cm. Leaf blade 3.5–14.2 × 1.5–5 cm, 5-plinerved, with the innermost pair of veins diverging in from the midvein 0.5–2 cm above the base in opposite or usually alternate fashion, elliptic, the base acute to obtuse, the apex acuminate, the margin denticulate to subentire, the adaxial surface glabrous, the abaxial surface densely covered with white stellate trichomes in between the veins and white intermixed with ferrugineus trichomes on the main veins. Inflorescence a terminal panicle 3.6–8 cm long, branching at or above the base, accessory branches absent, the axes covered with stellate hairs; bracteoles 2–5 × 0.5–3 mm, elliptic to linear-oblong, persistent. Flowers sessile or subsessile, 5 merous, not calyptrate or with the sepals fused in bud and rupturing, hypanthium 2.75–3.25 × 2.5–3 mm, campanulate, covered with stellate hairs, calyx tube 0.5 mm long, calyx lobes rounded-triangular to oblong, 1.5 × 1.5–2 mm, oblong, 1–1.5 mm long. Petals 3.5–4 × 3–4.5 mm, white, obovate, spreading, glabrous the apex rounded to emarginate. Stamens 10, 5–6 mm long, radially arranged around the style, the filaments 2.5–3 mm, with a geniculation near the apex, anthers 2.5–3 mm, linear-oblong, yellow, laterally compressed, the pore ca. 0.2 mm wide, ventrally inclined. Ovary 5-locular, half inferior, densely sericeous with trichomes surrounding and overtopping the stylar scar. Style ca. 5–7 mm long, straight but somewhat bent in some specimens, vertical distance from the anther to the stigma ca. 2.5-3 mm, the stigma punctiform, ca. 0.4-0.5 mm wide. Berry 4–5 × 4–5 mm, purple black. Seeds 0.65-0.8 mm long, angulate-ovoid, the testa smooth.

**Figure 174.**
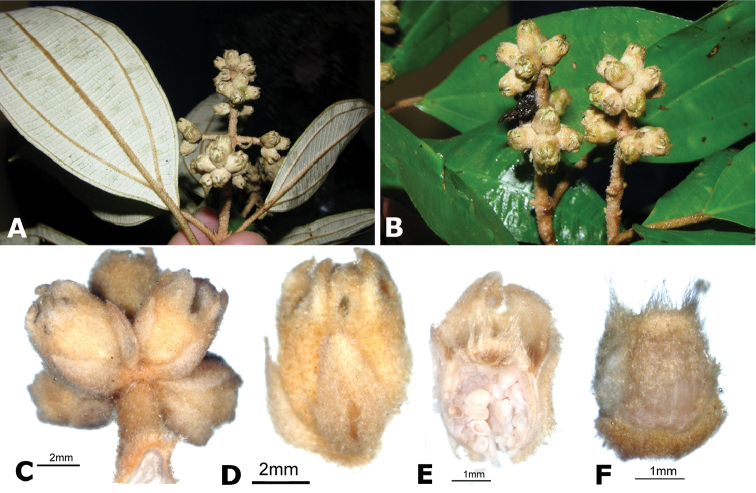
*Conostegia oligocephala*. **A** Branch apex showing leaf abaxial surface and infructescence **B** Infructescence **C** Close up of an infructescence showing sessile flowers subtended by bracteoles **D** Close up of a fruiting hypanthium **E** Longitudinal section of a fruiting hypanthium showing setose rim around the style scar **F** Close up of the ovary with the most of the hypanthium removed. Photos of specimen vouchered *R. Kriebel 5575*.

#### Distribution

**Figure 175.**
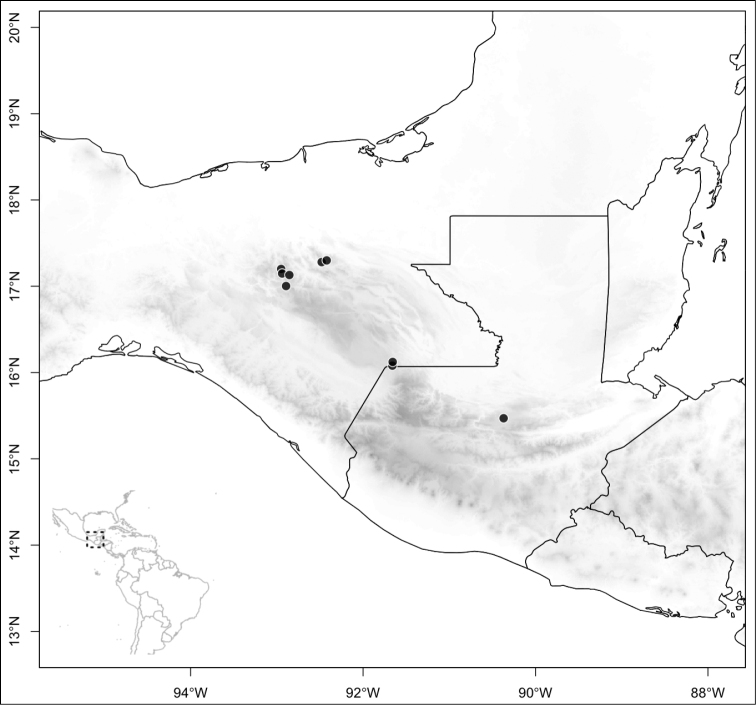
Distribution of *Conostegia oligocephala*.

(Fig. 175). Mexico and Guatemala at 1300-1950 m in elevation.

*Conostegia oligocephala* can be recognized by its leaf abaxial surface which is obscured by whitish and ferrugineaous trichomes, sessile flowers subtended by persistent bracteoles, non calyptrate calyx, and linear calyx teeth. This species has been confused with *Conostegia plumosa* and *Conostegia xalapensis* but can be distinguished from them by the lack of a calyptrate calyx and presence of evident calyx teeth. It is also similar to another non-calyptrate specie, *Conostegia fulvostellata*. See the latter species for differences between the two. The molecular phylogeny places *Conostegia oligocephala* as sister to *Conostegia incurva* with strong support. The two share a similar leaf indument and calyx teeth but are easy to separate based on inflorescence and flower size.

#### Specimens examined.

**GUATEMALA. Alta Verapaz**: Intersección de camino de terracería y Quebrada Sacsae, a lo largo del río, Kriebel et al. 5575 (NY, USCG).

**MEXICO. Chiapas**: steep wooded slope on the bank of the Río Hondo 4 miles north of Jitotol on the road to Pueblo Nuevo Solistahuacán, Breedlove 10134 (CAS); Lago de Monte Bello, 25 miles east of La Trinitaria, Breedlove 9712, 14981 (CAS); 10 km north of Jitotol near Río Hondo, Breedlove and Davidse 55149 (CAS); along Highway 195 between Ixtapa and Pichucalco 8 miles NW of Pueblo Nuevo Solistahuacán, Croat 47770 (CAS); at Clinica Yerba Buena, 2 km northwest of Pueblo Nuevo Solistahuacán, Raven and Breedlove 19919 (CAS); Colonia Kokijaz, Municipio Tila, Shilom Ton 6079 (CAS); on the slopes below Hy. 195 in the visinity of Clinica Yerba Buena, Thorne and Latrop 41087 (CAS).

### Conostegia ombrophila

Taxon classificationPlantaeMyrtalesMelastomataceae

(Gleason) Kriebel comb. nov.

urn:lsid:ipni.org:names:77156265-1

Fig. 176

Conostegia ombrophila (Gleason) Kriebel. Basionym: Clidemia ombrophila Gleason, Brittonia 3(2): 138-139. 1939. Type: Panama: Foothills of Garagará, Sambú basin, southern Darien, alt. 30-500 m, February 1912, H. Pittier 5610 (holotype: US!, F!).

#### Description.

Shrub to small tree 1.5–3.5 m tall with terete, caducously furfurate-lepidote branches; the nodal line not evident. Petiole 0.2–2.1 cm. Leaves of a pair equal to unequal in size. Leaf blades 4.5–17.7 × 1–5.8 cm, 3–5-plinerved, with the innermost pair of primary veins arising about 1–2 cm above the base and usually diverging from each other at their point of origin, elliptic, cuneate, apex caudate-acuminate, the margin entire, pocket like domatia absent or usually present at the base abaxially where the innermost pair of veins divere from the midvein, adaxially glabrous, abaxially glabrous or caducously lepidote. Inflorescence a pseudolateral paniculiform cyme divaricately branching from the base with slender branches, 2.2–5 cm long, triangular to subulate and fused basally to forma a persistent, shallow amplexicaul collar, 1–1.5 × 0.5–1 mm, persistent. Flowers sessile or subsessile, 4-merous, not calyptrate or evidently fused in bud, flower buds 3–4 × 1.5–2 mm, the hypanthium 2.25–2.75 × 1.25–1.75 mm, subglobose, calyx lobes broadly triangular ovate, 0.5–1 mm long, calyx teeth subulate, 0.5 mm long. Petals 4.75–5 × 2.25–2.75 mm, translucent white, elliptic to oblong, spreading to reflexed, glabrous, rounded to emarginate. Stamens 8, 2.5–4 mm long, erect and radially arranged around the style, the filament 1.5–2.6 mm with a geniculation near the apex, translucent white, anthers 1–1.5 × 0.5–1 mm, elliptic-ovate and laterally compressed, yellow, the pore truncate to dorsally inclined, ca. 0.19 mm wide. Ovary 4-locular, inferior, glabrous. Style 6.5–7 mm, straight to slightly curving, vertical distance of the anthers from the stigma 2.25–2.75 mm, horizontal distance absent, stigma punctiform, 0.35–0.5 mm wide. Berry 4–5 × 4–5 mm, dark purple to black. Seeds 0.4–0.9 mm long, ruminate to tuberculate, smooth.

**Figure 176.**
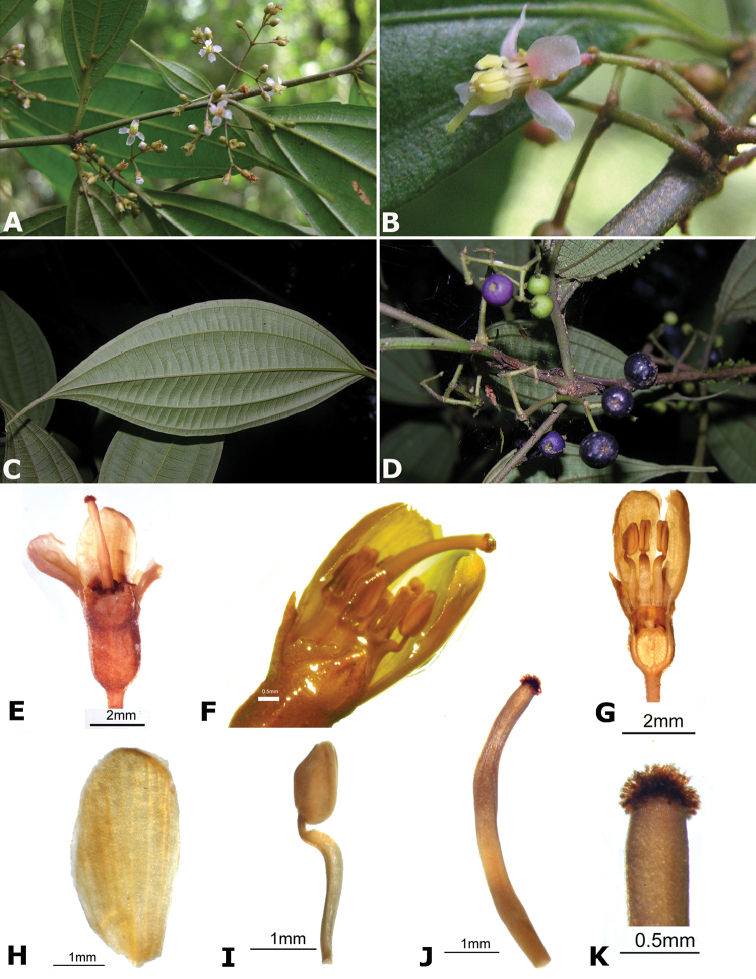
*Conostegia ombrophila*. **A** Flowering branch. Note marsupiform domatia **B** Flower **C** Leaf abaxial surface **D** Fruits **E** Flower at early anthesis with stamens still inflexed **F** Pickled flower at anthesis **G** Longitudinal section of a flower at anthesis with the style removed **H** Petal **I** Stamen **J** Style **K** Stigma. Photos **A–B, E–K** of specimen vouchered *R. Kriebel 3120*, and **C–D** from specimen vouchered *R. Kriebel 5396*.

#### Distribution

**Figure 177.**
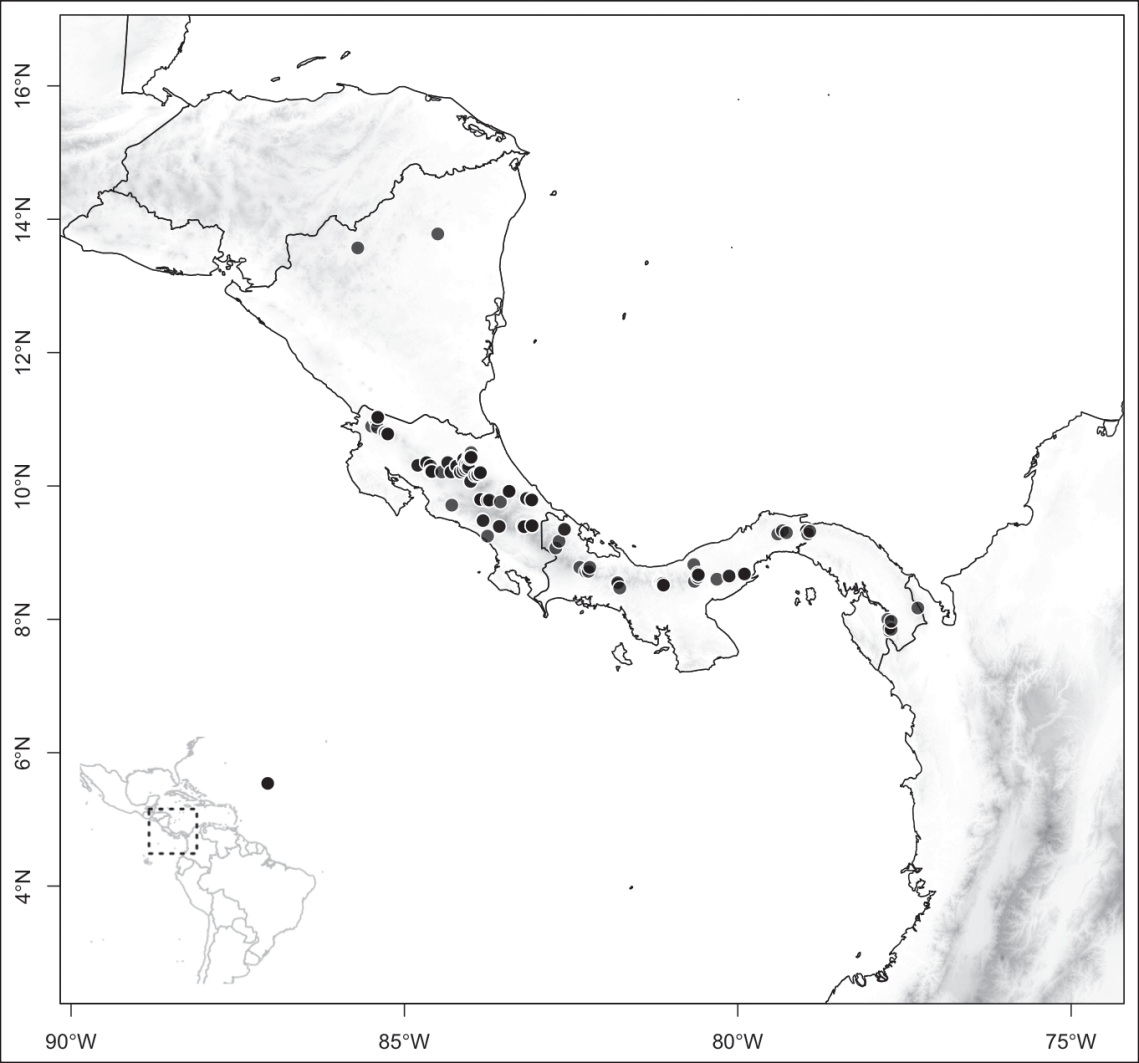
Distribution of *Conostegia ombrophila*.

(Fig. 177). Nicaragua, Costa Rica (including Cocos Island) and Panama, at 0–1800 m.

*Conostegia ombrophila* can be recognized by its almost entire glabrosity, relatively small plinerved leaves with caudate apices and pocket domatia usually present at the base of the lamina abaxially. It also has pseudolateral, divaricately branched and wiry inflorescences with persistent bracteoles fused at the base to form a small collar. Its anthers are laterally compressed and their shape is unusual in that they are somewhat ovate and conspicuously flatted at an angle towards the apex. *Conostegia ombrophila* in general is a very uniform species morphologically but two morphotypes of the species are recognizable. The common morphotype occurs more commonly at middle elevation cloud forests and has leaf domatia at the base of the blade abaxially. The lowland morphotype which occurs for example at La Selva Biological Station in Costa Rica does not tend to have domatia. Suprisingly, the two morphotypes did not form a sister pair in the molecular phylogeny. Further morphological studies should be made and more populations collected for genetic analyses. The non domatia bearing morphotype can also resemble *Conostegia grayumii* in places where they are sympatric like in the Sarapiquí area in Costa Rica. The latter can be distinguished by the more glabrous and smaller leaves of *Conostegia grayumii* as well as the five merous flowers and seeds with tubercles on the edges in the latter. In the lowlands, it is not uncommon to find several fruit with galls.

#### Specimens examined.

**COSTA RICA. Cartago**: Paraíso, Kiri Lodge, sendero a la catarata, Kriebel et al. 5468 (INB, NY); loc. cit, Kriebel and Solano 3120 (INB, NY); Pejibaye, R. V. S. La Marta, Kriebel and Soto 4887 (INB, NY); Turrialba, P.N. Barbilla, Sector Quebrada San Miguel, Camino a Moravia de Chirripó, Mora and Rojas 1519 (CAS, CR, INB, NY); Turrialba, El Humo, Albergue El Copal, Santamaría and González 6407 (INB, NY). **Guanacaste**: Cordillera de Tilarán, Monteverde Cloud Forest Reserve along the sendero El Valle, Almeda and Anderson 5441 (CAS, CR, NY). **Heredia**: Sarapiquí, La Tirimbina, Kriebel and González 5396 (INB, NY). **Limón**: Talamanca, P.N. La Amistad, Bratsi, sendero Transtalamanca, entre Ujarrás y San José Cabécar, alrededores del Río Coén, Rodríguez et al. 10911 (INB, NY); R. B. Bosque Lluvioso, Sendero derecho, Vargas, Blanco and Umaña 3528 (INB, NY). San José: P. N. Braulio Carrillo, cerca del tú nel, Kriebel 4896 (INB, NY); Dota, estribaciones sureste del Cerro Lira, Martén and Herrera 745 (CR, NY).

**NICARAGUA. Zelaya**: along new road to Mina Nueva America (leading more or less westward from 14.3 km N of El Empalme on main road to Rosita), ca. 7.7 km from main road, Stevens and Krukoff 12706 (CAS).

**PANAMA. Bocas del Toro**: on Chiriqui trail E slope of La Zorra to Divide, Kirkbride and Duke 828 (NY). **Coclé**: Along river leading up mountain to Alto Calvario and trout stream from La Junta near Limon, Folsom 5886 (MO, NY). **Panama**: Cerro Campana, McDaniel 6885 (NY). **Veraguas**: 5 km w of Santa Fé on road past Escuela Agrícola Alto Piedra on Pacific side of the divide, Croat 22987 (MO, NY); Primary forest on Caribbean slope above Rio Primero Brazo 5 mi nw of Santa Fé, Croat 23207 (MO, NY); Valley of Río Dos Bocas along road between Escuela Agrícola Alto Piedra and Calovebora15.6 km northwest of Santa Fé, Croat 27626 (MO, NY); Along the Santa Fé to Colevebora road beyond Escuela Agrícola Alto Piedra along first mayor stream ca. 3 mi from the fork in the road at the school, Croat 49008 (MO, NY).

### Conostegia osaensis

Taxon classificationPlantaeMyrtalesMelastomataceae

(Aguilar, Kriebel & Almeda) Kriebel comb. nov.

urn:lsid:ipni.org:names:77156266-1

Fig. 178

Conostegia osaensis (Aguilar, Kriebel & Almeda) Kriebel. Basionym: Miconia osaensis Aguilar, Kriebel and Almeda, Proc. Calif. Acad. Sci. Series 4, 59(10): 490, f. 1A–J, 2A–D. 2008. Type: Costa Rica. Puntarenas: Cantón de Osa, Reserva Forestal Golfo Dulce, entrada a Chocuaco, por la casa de Moncho, 200–350 m, 28 May 1997, R. Aguilar 5145 (holotype: INB!; isotypes: CAS!, CR!, MO!, USJ).

#### Description.

Trees 9–25 m tall with the uppermost flattened and two sided branchlets completely covered with a stellulate-lepidote indumenta; the nodal line present. Leaves of a pair equal to subequal in size. Petioles 1.5–4.5 cm long. Leaf blades 7.5–21 × 3–7 cm, 5-plinerved, with the innermost pair of primary veins diverging ca. 0.5–1.5 cm above the blade base in opposite, alternate or subalternate fashion, elliptic to elliptic-oblong or slightly elliptic-lanceolate, the base acute, the apex acute to acuminate, the margin entire to inconspicuously crenulate-denticulate, the adaxial surface glabrous and inconspicuously glandular puncticulate, the abaxial surface completely covered by peltate scales. Inflorescence a terminal panicle 10.8–15 cm long branching above the base, accessory branches present, reddish stellulate-lepidote indument throughout; bracts and bracteoles 1–1.5 × 0.25–0.5 mm, triangular, persistent. Pedicels 0.25–0.5 mm. Flowers 5-merous, calyx calyptrate, flower buds 3–4.5 × 2–2.75 mm, elliptic-ovate, calyx teeth, the base rounded, the apex acute, not constricted, the hypanthial and calyptrate portions differentiated in texture, color and indument different, the calyptra being white or translucent, very thin and more sparsely pubescent than the hypanthium, the latter campanulate to urceolate, 2.5–3 × 2.25–2.75 mm, densely covered with peltate scales that grade into stellate hairs, the torus also beset with scales. Petals 5–8 × 3–6 mm, white, obtriangular, spreading at anthesis, emarginate at the apex, glabrous. Stamens 10, Stamens ca. 5–8 mm long, androecium slightly bilaterally symmetric, the filaments 2.5–4.5 mm, white, 3.25–3.75 × 1–1.5 m, oblong, yellow, laterally compressed, the connective dorsally thickened, the pore ca. 0.2 mm wide, slightly ventrally inclined. Ovary 5-locular, inferior, apically glabrous and slightly ribbed, forming a low collar around the style base, with pronounced elevated lines. Style ca. 5 mm long, straight and abruptly curved near the apex, vertical distance from the anther to the stigma ca. 0–0.5 mm, stigma punctiform to truncate, ca. 0.8 mm wide. Berry ca. 6 × 6 mm, purple black. Seeds 1.3–1.9 mm long, broadly pyramidate, rounded to bluntly angled on the convex face, the testa smooth.

**Figure 178.**
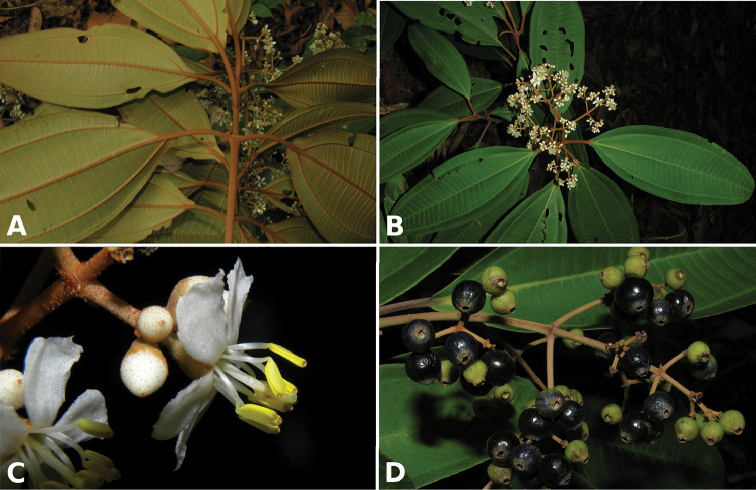
*Conostegia osaensis*. **A** Habit and detail of leaf abaxial surface **B** Flowering branch **C** Close up of the flower **D** Infructescence. Photographs taken Reinaldo Aguilar and vouchered *R. Aguilar 10200*.

#### Distribution

**Figure 179.**
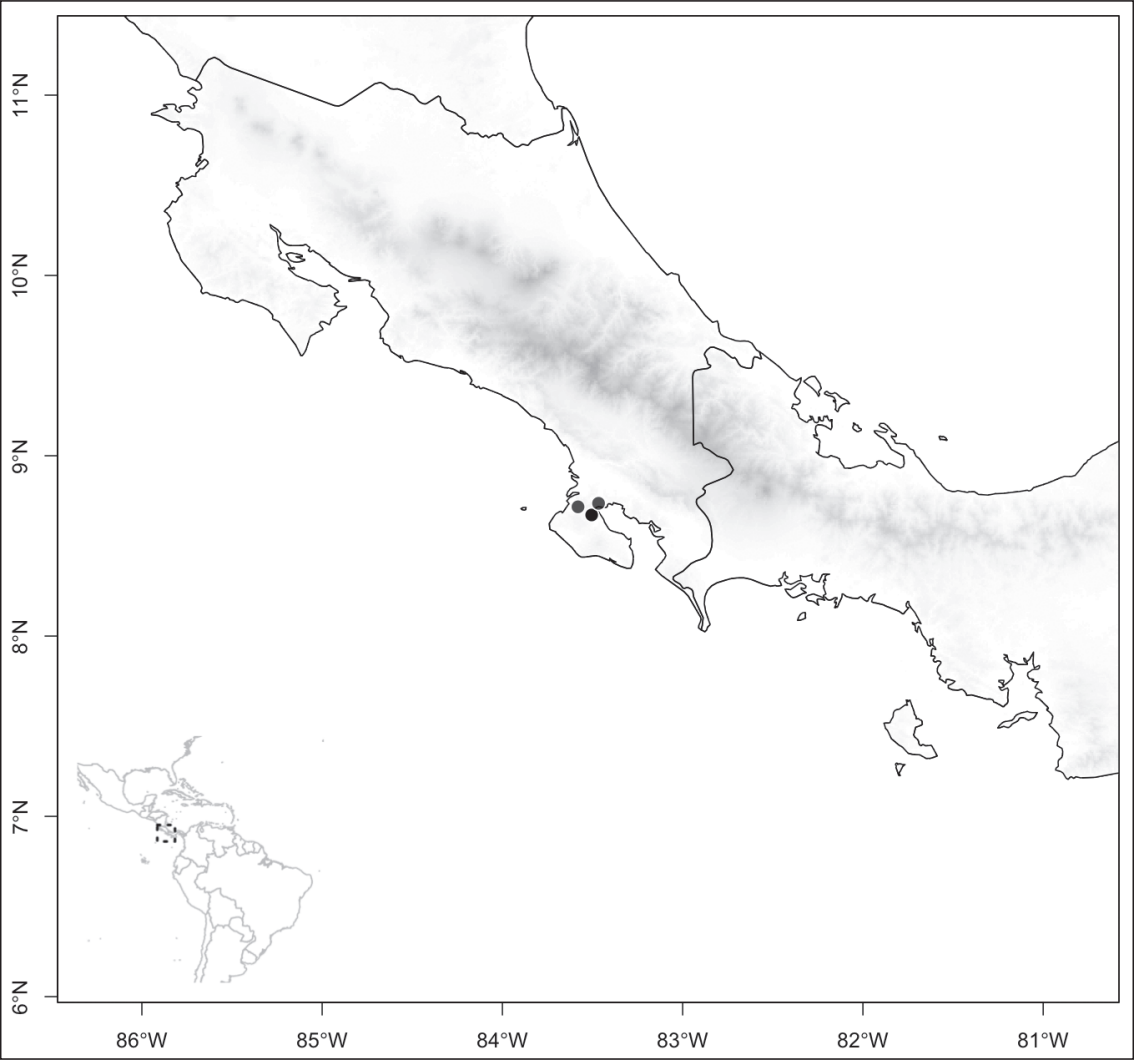
Distribution of *Conostegia osaensis*.

(Fig. 179). Endemic to the Osa Peninsula in southern Costa Rica, 40–200 m in elevation.

*Conostegia osaensis* is endemic to the Osa Peninsula of Costa Rica and quite distinctive as it tends to be a large tree up to about 25 m tall. In addition, the leaf abaxial surface is covered with a stellate lepidote indument and the flowers are calyptrate, not pleiostemonous, and have a short style. In the molecular phylogeny *Conostegia osaensis* falls sister to the clade comprised of *Conostegia plumosa*, *Conostegia speciosa*, *Conostegia subcrustulata*, and *Conostegia xalapensis*. With these taxa *Conostegia osaensis* shares the calyptrate calyx and the short style. *Conostegia osaensis* has the largest seeds of any species in *Conostegia*.

#### Specimens examined.

**COSTA RICA. Puntarenas**: Rincón, cerca de Banegas, 1 km al este del centro del pueblo de Banegas, Estación Biológica Los Charcos, sendero Dendrobates, Aguilar 10200, 10228 (INB); Mogos, Bahía Chal, entrada a Chocuaco a 35 km de Chacarita, finca de Carlos Rojas, Aguilar 6208 (INB).

### Conostegia papillopetala

Taxon classificationPlantaeMyrtalesMelastomataceae

(Kriebel & Almeda) Kriebel comb. nov.

urn:lsid:ipni.org:names:77156267-1

Fig. 180

Conostegia papillopetala (Kriebel & Almeda) Kriebel. Basionym: Miconia papillopetala, Phytotaxa 134 (1): 32. 2013. Type: Panama. Veraguas: Parque Nacional Santa Fé. Sendero a la cima del Cerro Mariposa, 960 m, N 08.50412, W 081.11999, 16 September 2011, R. Kriebel & J. Burke 5718 (holotype: NY!, isotypes: INB!, PMA!).

#### Description.

Shrubs 1–2.5 m tall with young stems copiously covered with pinoid hairs intermixed with asperous-headed hairs that are both golden-orange in color, nodal line not evident and concealed by the copious indument. Petioles 0.1–1 cm. Leaves subisophyllous to anisophyllous; blades 3.5–16 × 1.5–8.5 cm, 3–5-plinerved, diverging from the midvein 0.5–3 cm above the blade base usually asymmetrically, elliptic, base obtuse to rounded and sometimes oblique, apex acuminate, the margin denticulate, adaxially glabrous except for short and long pinoid hairs on the main veins towards the base, somewhat bullate and dark green when alive, abaxially densely pubescent on tertiary and higher order veins with pinoid golden-orange hairs. Inflorescences terminal, lax dichasia branched from the base, (4-)7–10 cm long, copiously covered with golden-orange pinoid hairs intermixed with asperous-headed hairs; bracts to 8 mm long, linear oblong; bracteoles 0.5–1 mm long, lanceolate, less pubescent than the rest of inflorescence rachis, drying pinkish. Pedicels ca. 0.5 mm. Hypanthia campanulate 1.25–2 × 1–1.25 mm, densely covered with asperous-headed that appear somewhat stellate. Flowers 5-merous. Calyx fused in bud, shortly apiculate and less pubescent than the hypanthium, rupturing at anthesis into irregular, broadly rounded hyaline lobes 0.25–0.75 mm long and 0.5–0.75 mm wide at the base, the exterior calyx teeth 0.25–0.5 mm long, linear oblong, the calyx tube 0.25–0.5 mm long. Petals 1.25–2 × 2.5–3 mm, ovate, pink, papillose abaxially, reflexed at anthesis, emarginate. Stamens 10, 3–3.5 mm long, radially arranged around the style; filaments 1.5–2 mm long, geniculate near the apex, translucent white; anthers 1.25–1.75 × 0.4–0.6 mm, linear-oblong, somewhat laterally compressed, cream yellow, pores 0.1–0.15 mm, truncate to somewhat ventrally inclined. Ovaries 5-locular, half inferior, the apex elevated into a low papillose collar. Styles 4.5–4.75 mm long, slightly curved, distance between the anther apex and the stigma 1–1.5 mm; stigmas truncate to capitellate, 0.4 mm wide. Berries pink when immature and turning purple at maturity, 3.3–4.5 × 3.5–4.5 mm; seeds ovoid and angled, 0.4–0.5 × 0.2–0.3 mm, orange-brown, lateral symmetrical plane ovate to triangular, the highest point toward the chalazal side, antiraphal symmetrical plane ovate-triangular and inconspicuously verruculose on the angles, raphal zone narrowly triangular and extending the length of the seed, expanded into an appendage that covers about 30% of the seed length.

**Figure 180.**
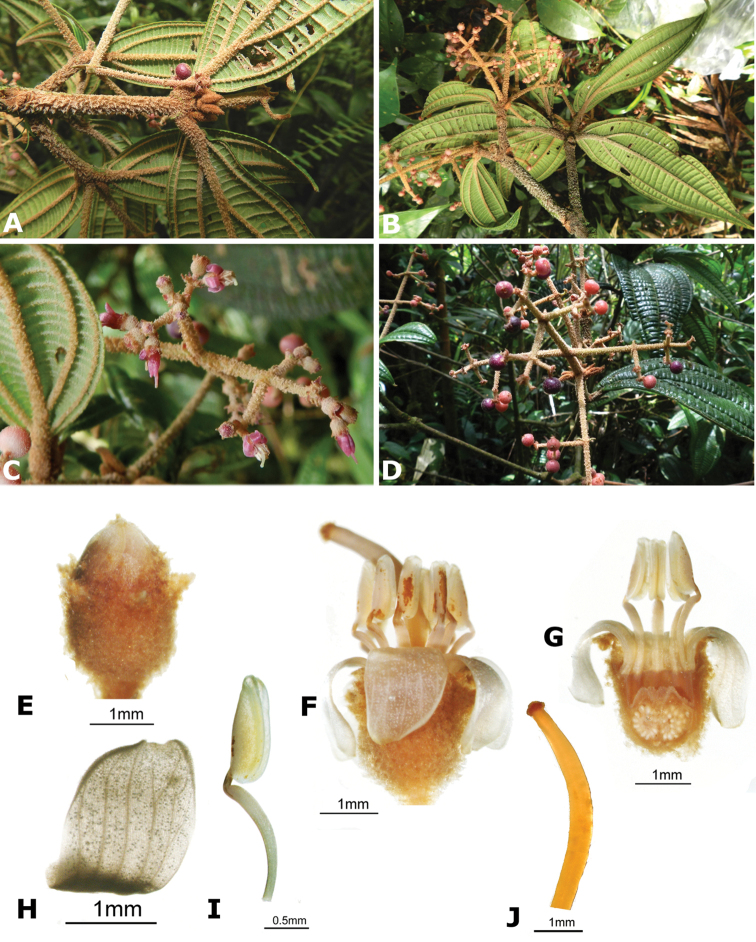
*Conostegia papillopetala*. **A** Stem apex **B** Branch **C** Inflorescence **D** Infructescence **E** Flower bud with closed hyaline calyx and apiculum **F** Pickled flower **G** Longitudinal cut of flower with style removed **H** Petal **I** Stamen. Photos of specimen vouchered *R. Kriebel and J. Burke 5718*.

#### Distribution

**Figure 181.**
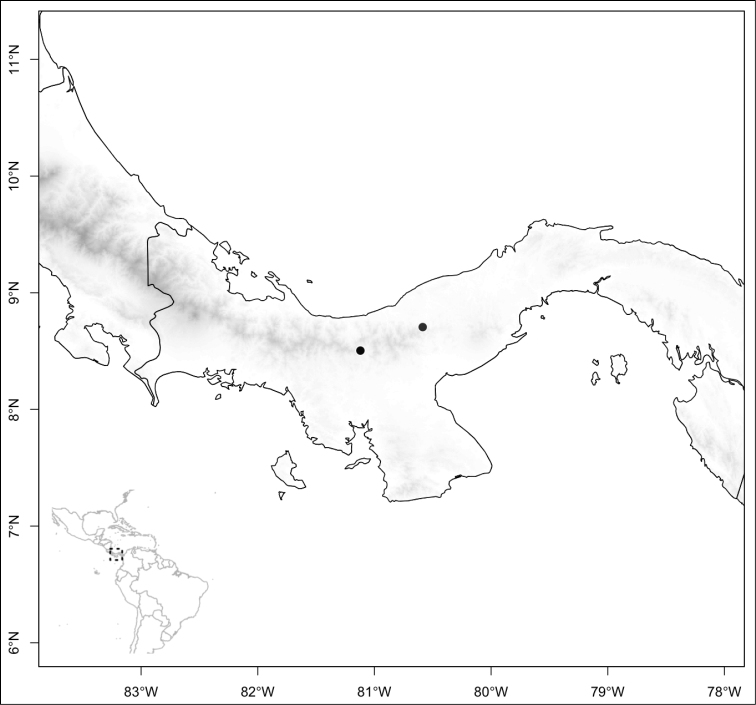
Distribution of *Conostegia papillopetala*.

(Fig. 181). Endemic to cloud forests in the provinces of Coclé and Veraguas, Panamá, at 750–1400 m elevation.

*Conostegia papillopetala* can be recognized by its indument of long asperous headed hairs, short petiolate, somewhat bullate, narrow and plinerved leaves, small, herkogamous flowers with pink papillose petals and seeds with tubercles on the edges. It is similar and closely related to *Conostegia friedmaniorum*, *Conostegia galdamesiae*, and *Conostegia pendula*. See the discussion section under these species for difference with *Conostegia papillopetala*.

#### Specimens examined.

**PANAMA. Coclé**: Atlantic slope near the continental divide along lumbering road N of El Copé, 9.4 km above El Copé, Croat 44624 (CAS, PMA); Atlantic slope near the continental divide along lumbering road N of El Copé, 2.2 km N of lumber sawmill, Croat 44668 (CAS, MO, PMA); Lumber camp at Alto Calvario, 7 km N of El Copé, 900 m, Folsom 1276 (CAS, INB, MEXU, MO, PMA); Near continental divide along lumbering road, 2.3 km beyond sawmill above El Copé, Hammel 990, 1054 (CAS, MO, PMA). **Veraguas**: Trail on ridge summit of Cerro Tute, Cordillera de Tute, 1 km past Escuela Agricola Altos de Piedra, W of Santa Fe, Knapp and Sytsma 2654 (BM, CAS, MO, PMA); Santa Fe, Altos de Piedra, trail leading up Cerro Mariposa (=Cerro Arizona) about 2 km past the Escuela Agricola, ca. 3 km from summit, Penneys and Blanco 1707 (CAS, FLAS, NY, PMA, US).

### Conostegia peltata

Taxon classificationPlantaeMyrtalesMelastomataceae

(Almeda) Kriebel comb. nov.

urn:lsid:ipni.org:names:77156268-1

Fig. 182

Conostegia peltata (Almeda) Kriebel. Basionym: Miconia peltata Almeda, Proc. Calif. Acad. Sci. Series 4, 46(9): 217, f. 4. 1989. Type: Panama. Panamá: near Cerro Jefe, along road towards Alto Pacora, forested slopes, ca. 850 m, ca. 915'N, 7930'W, 27 December 1985, *G. McPherson 7882* (holotype CAS!, isotype MEXU!, MO!, PMA!, US!).

#### Description.

Small trees 3–6 m tall with compressed branches when young that are covered with reddish pinoid hairs; the nodal line absent or obscured by indument. Leaves of a pair somewhat unequal to very unequal in size. Petiole 1–3.5 cm. Leaf blade 5–15 × 2.5–8.5 cm, 5–7 nerved, elliptic-ovate to ovate, the base peltate, the apex acuminate, margin inconspicuously undulate-denticulate to entire, the adaxial surface glabrous, the abaxial surface sparingly beset with spreading pinoid hairs and an inconspicuous appressed glandular puberulence mostly on the veins. Inflorescence a terminal multi flowered paniculiform dichasium 2–4 cm long, branching at the base, accessory branches absent, the rachis reddish covered with reddish pinoid hairs; bracteoles 0.25–0.5 × 0.25 mm, subulate, persistent. Pedicels ca. 1 mm long. Flowers 5-merous, neither calyptrate nor fused in bud, flower buds 1.9–2.15 × 1.25–1.5 mm, the hypanthium 1.5–2 × 1.5–2 mm, campanulate to subglobose, moderately to sparsely covered with stipitate-stellate or short reddish pinoid hairs, calyx tube ca. 0.25 mm long, calyx lobes triangular, 0.25 mm long, calyx teeth triangular, 0.25 mm long, the torus puberulent. Petals 2.5–3 × 1–1.25 mm, reddish-pink, oblong-elliptic, glabrous, spreading at anthesis, apically rounded almost acute. Stamens 10, 2.5–3 mm long, radially arranged around the style, the filaments 1.5–2 mm, with a geniculation near the apex, white, anthers 1–1.5 × 0.25–5 mm, oblong, pale yellow, slightly laterally compressed, the pore ca. 0.15 mm wide, somewhat dorsally inclined. Ovary 5 locular, 2/3 inferior, the apex fluted and sparingly glandular puberulent. Style 4.25–4.75 mm, curving downward gradually, vertical distance from the anther to the stigma 1.25–1.75 mm, horizontal distance a 1.5 mm, the stigma truncate, ca. 0.3 mm wide. Berry 3–4 × 3–4 mm, purple black. Seeds ca. 0.5 mm long, irregularly angulate pyramidate, smooth with polished angles on the convex face.

**Figure 182.**
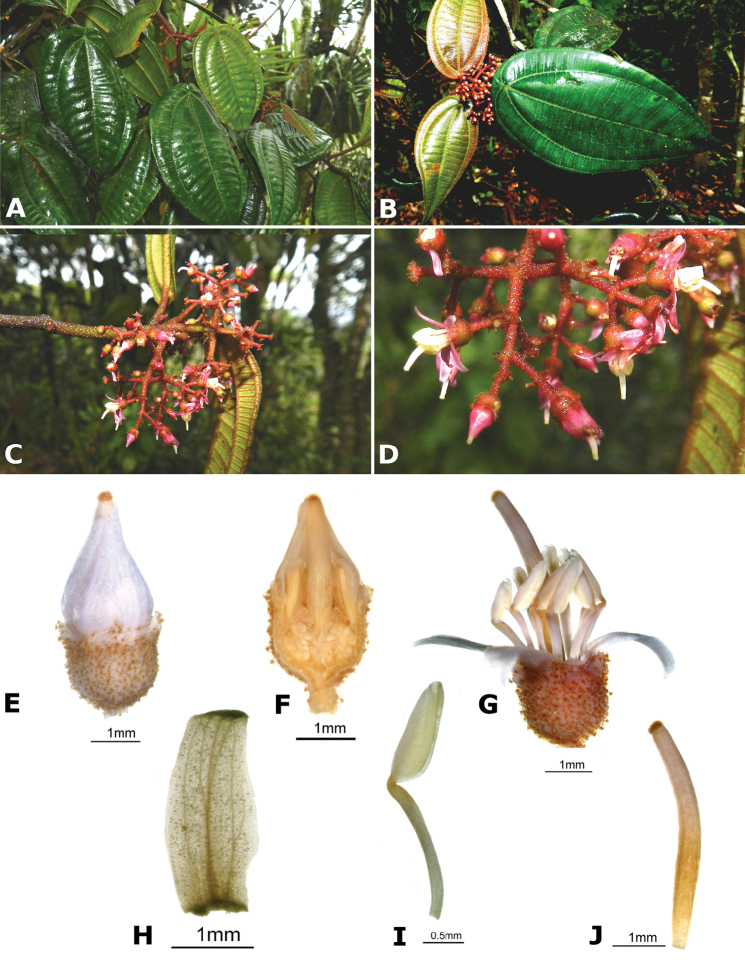
*Conostegia peltata*. **A** Branch **B** Leaf adaxial surface (notice peltate leaf base) **C** Inflorescence **D** Close up of the inflorescence **E** Flower bud **F** Longitudinal section of flower bud **G** Flower at anthesis **H** Petal **I** Stamen. Photos of specimen vouchered *R.Kriebel and J. Burke 5658*.

#### Distribution

**Figure 183.**
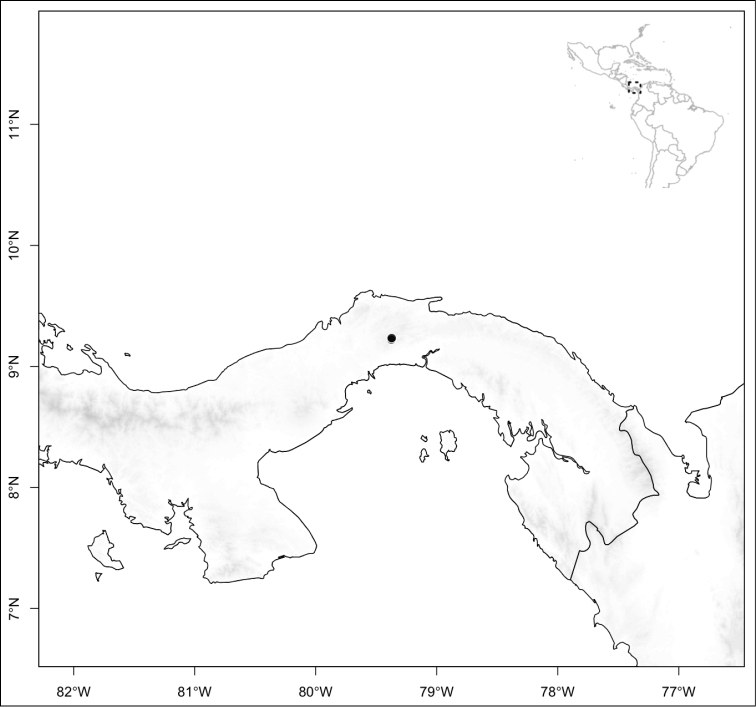
Distribution of *Conostegia peltata*.

(Fig. 183). Endemic to Cerro Jefe in Panama, 850–1000 m in elevation.

*Conostegia peltata* is a Cerro Jefe endemic quite distinctive because of its peltate leaves. It is closely related to another Cerro Jefe endemic, *Conostegia jefensis*, as well as to *Conostegia consimilis* and *Conostegia trichosantha* and shares the mostly linear-oblong petals with these species. With these particular petals, this clade is convergent with other taxa in section *Geniculatae* like *Conostegia friedmaniorum*.

#### Specimens examined.

**PANAMA. Panamá**: Cerro Jefe, Aranda 185 (CAS); P. N. Chagres, Cerro Jefe, subiendo por calle principal hacia la cima, Kriebel and Burke 5658 (NY, PMA); Near Cerro Jefe along road towards Alto Pacora, McPherson 7882 (CAS).

### Conostegia pendula

Taxon classificationPlantaeMyrtalesMelastomataceae

(Umaña & Almeda) Kriebel comb. nov.

urn:lsid:ipni.org:names:77156269-1

Conostegia pendula (Umaña & Almeda) Kriebel. Basionym: Miconia pendula Umaña & Almeda, Novon 3(1): 8, f. 2. 1993. Type: Costa Rica. Cartago: Refugio Nacional de Vida Silvestre Tapantí, orilla de Sendero Los Palmitos, 1300–1400 m, 0944'00” N, 8347'00"W, 2 August 1990, G. Umaña, Kennedy, H., Nilson, V., & R. Chacón 391 (holotype: CR!, isotypes: BM, BR, CAS!, COL, F!, MEXU!, MO!, NY!, PMA, US!, USJ).

#### Description.

Shrub to small tree 1.5–5 m tall with somewhat tetragonal stems in newer branches densely covered with a lanate indument of variable dendritic trichomes; the nodal line present. Petioles 1–7 cm. Leaves at a node equal or somewhat unequal at a node. Leaf blades 6.5–28 × 3.5–13.5 cm, 7-plinerved, with the inner pairs of innermost veins arising 1–5 cm above the base mostly in alternate fashion, elliptic to elliptic-ovate, the base obtuse to asymmetrical, the apex acuminate, the margin entire to inconspicuously denticulate, the adaxial surface strigose or lightly lanate to glabrous, lanate on the primary veins abaxially, and with furfuraceous hairs on higher order veins. Inflorescence a pendant modified cyme branching well above the base, 4–12 cm long, the flowers clustered at the end of the branches, accessory branches absent, rachis lanate, bracteoles 0.2–0.3 mm, persistent. Pedicel 0.5–2.5 mm. Flower buds 3–4 × 2–3 mm. Flowers 5-merous, calyx not calyptrate but closed in bud and crowned by an apiculum, rupturing irregularly at anthesis into 5 hyaline irregular lobes 0.25–0.5 mm long, exterior calyx teeth narrowly triangular, 0.9–1.3 mm long, torus glabrous; the hypanthium 2–3 × 1–2 mm, campanulate. Petals 2.9–3.5 × 1 mm, pale pink, linear-oblong, not observed at anthesis, glabrous, the apex obtuse to rounded. Stamens 10, ca. 3–4 mm, radially arranged around the style, the filaments ca. 2 mm long, geniculate near the apex, white, anthers 1–1.5 × 0.35–0.5 mm, linear-oblong, somewhat recurved, yellow, the connective thickened dorsally, the pore 0.1 mm wide, slightly dorsally inclined. Ovary 5 locular, 2/3 inferior, slightly fluted and with glandular hairs on the apex. Style reportedly 2.5–5 mm long, not observed at anthesis but notes on the type suggest it is exserted, stigma truncate and somewhat dilated. Berry 4–5 × 4–5 mm, pink turning purple black when mature. Seeds 0.4–0.6 mm long, angular pyramidate to somewhat cescent shaped in profile, the testa smooth.

#### Distribution

**Figure 184.**
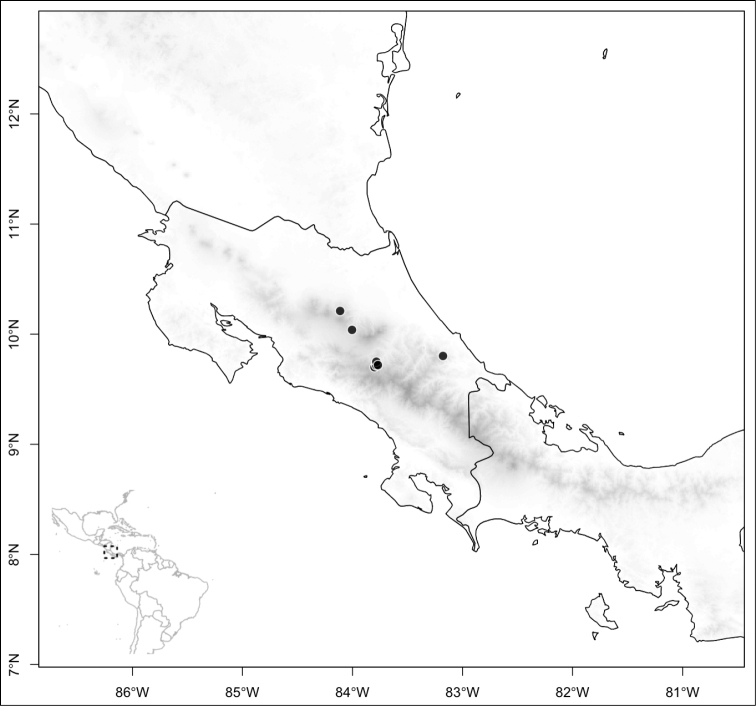
Distribution of *Conostegia pendula*.

(Fig. 184). Endemic to cloud forests of the Caribbean slope of the Central and Talamanca Cordilleras of Costa Rica, 1200–1800 m in elevation.

The description of this species is for the most part based on the original one by Almeda and Umaña Dodero (1993). Few recent collections have been made since its description but as Almeda and Umaña Dodero (1993) predicted, the species has been collected in the Cordillera de Talamanca in Fila Matama, Limón. The notes on the type specimen describe the anthers around the style which suggests the style is exserted as in its close relative like *Conostegia friedmaniorum*. Almeda and Umaña Dodero (1993) also noted the filament geniculation in this species, something not commonly done. They mention strong herbivory on these plants as is frequently noted in species of section *Geniculatae*. The label on *Almeda et al. 7245* (CAS) records a pleasant acidic flavor of the berries of this species.

#### Specimens examined.

**COSTA RICA. Cartago**: Refugio Nacional de Vida Silvestre Tapantí, sendero Palmito about 6 km from main gate to refuge, Almeda et al. 7245 (CAS, CR); Tapantí Reserve ca 1 km S of jct of Quebrada Salto and Río Grande de Orosí, Croat and Grayum 68255 (CAS); in forest along creek debouching into Río Grande de Orosi, from the east, ca. 1 km upstream from confluence of Quebrada Salta, Tapantí, Grayum and Sleeper 3486 (CAS, CR); P. N. Tapantí, sendero Palmito, Umaña, Valerio and Chacón 378 (CAS, CR). **Heredia**: Cantón de Barva, La Legua, Finca Montreal, ridge between headwaters of Río Volcán and Río San Fernando, Boyle et al. 1168 (CAS, CR, INB). **Limón**: P. N. La Amistad, Fila Matama, cerca de 11 km SW del pueblo de Aguas Zarcas, Santamaría et al. 6672 (INB, NY).

### Conostegia plumosa

Taxon classificationPlantaeMyrtalesMelastomataceae

L.O. Williams, Fieldiana

Conostegia plumosa L.O. Williams, Fieldiana, Bot. 29: 562. 1963. Type: British Honduras [= Belize]. Middlesex: 200 ft elev, 10 July, 1929, W. Schipp 232 (holotype: F!, isotypes: A, CAS!, GH, MO!, US!).

#### Description.

Trees 2.7–15 m tall with flattened branches which soon become terete and are covered with stellate and dendritic hairs in younger parts; the nodal line inconspicuous. Leaves of a pair equal to somewhat unequal in length. Petiole 0.9–4.5 cm. Leaf blade 6–21 × 2–6 cm, 3–5 plinerved, with innermost pair of veins diverging from the midvein above the base in opposite or alternate fashion, elliptic to elliptic ovate, the base acute to attenuate, the apex acute to acuminate, the margin serrulate, the adaxial surface stellate pubescent when young and glabrous with age, the abaxial surface tan to white color from the stellate and plumose hairs. Inflorescence a terminal panicle 3.3–12 cm long, the rachis covered with stellate and plumose hairs, linear, the bracteoles 2–8 mm long, deciduous. Pedicel absent or to 0.5 mm. Flowers 6 merous, calyptrate. Floral buds 4–6 × 2.4–3.25 mm, ovoid, the base rounded, the apex obtuse, slightly constricted in the middle, with six linear appendages ca. 2.5 mm long appressed to the top of the calyptra. Hypanthium 2.5–3 × 3–3.25 mm, with stellate and plumose hairs. Petals 4–6 × 1.5–2 mm, white, spathulate, spreading, glabrous. Stamens 12(-13), 4.25–5.5 mm long, the filament 2–2.5 mm long, white, the anthers 2.25–2.75 × 0.5–0.75 mm, linear-oblong and somewhat recirved, yellow, the pore 0.1–0.2 mm in diameter, subterminal. Ovary 6 locular, inferior, the apex glabrous and forming an irregular collar around the style. Style 3.5–6 mm, apparently straight, distance between the anthers and stigma ca. 0–1 mm, stigma truncate, 0.35–0.65 mm wide. Berry 5–6 × 5–6 mm, purple-black. Seeds 0.65–1.2 mm, obliquely pyramidal, the testa angulate and smooth.

#### Distribution

**Figure 185.**
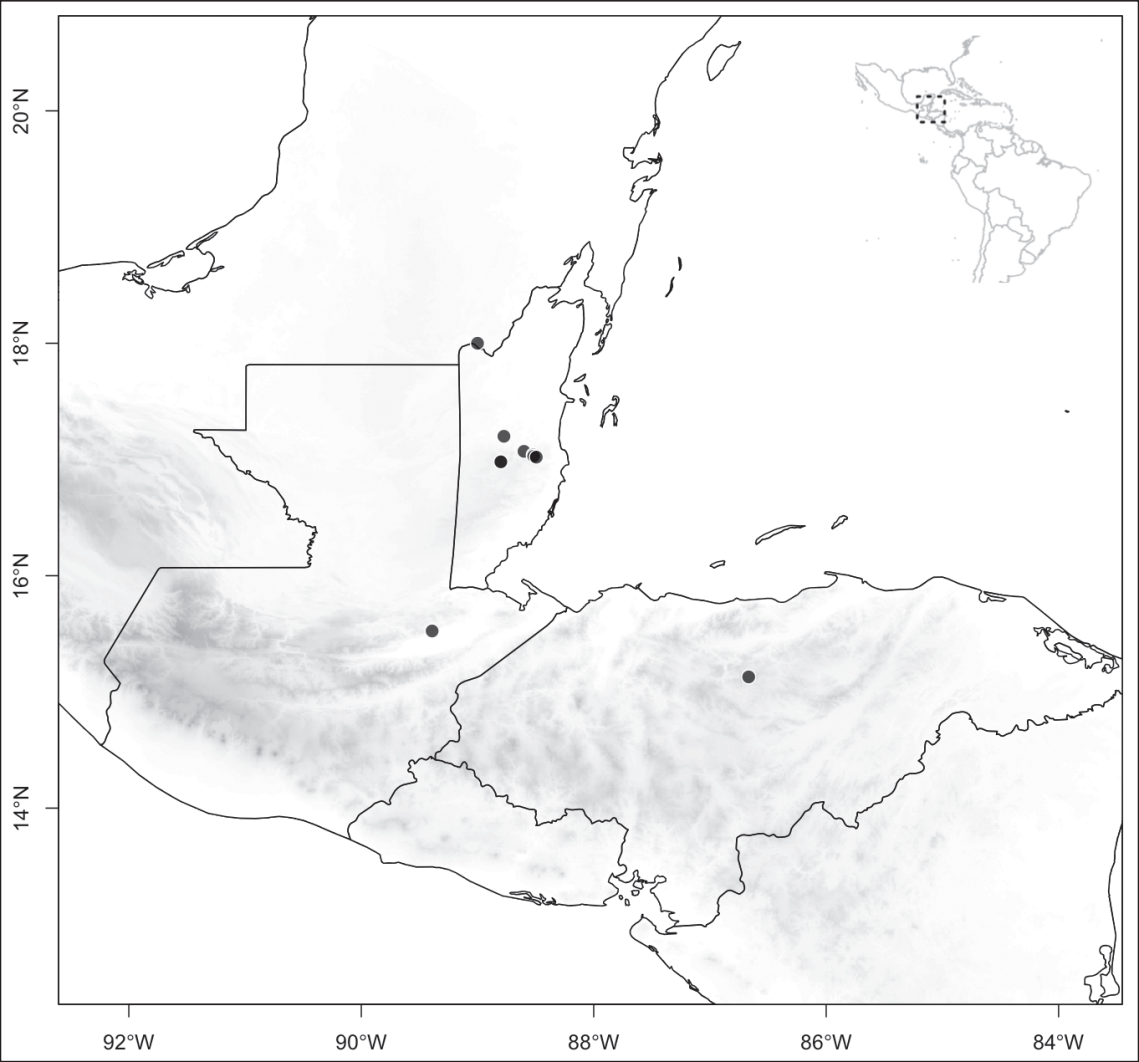
Distribution of *Conostegia plumosa*.

(Fig. 185). Belize, Guatemala, Honduras and in Chiapas in Mexico, at 50–1000 m in elevation.

*Conostegia plumosa* is a rare species and one of the few in the genus to have the abaxial leaf surface tan colored from the dense indument covering it. This characteristic leaf underside is shared with *Conostegia dissitinervia*, *Conostegia centrosperma*, *Conostegia incurva*, *Conostegia oligocephala* and *Conostegia xalapensis*. In particular, *Conostegia plumosa* has been confused with *Conostegia oligocephala* and *Conostegia xalapensis*, both of which occur in the same general area of northern Central America. The other species mentioned are endemic to southern Central America. *Conostegia plumosa* shares the calyptrate calyx and short style with *Conostegia xalapensis* but not with *Conostegia oligocephala* which lacks the calyptrate calyx and has an exserted style. *Conostegia plumosa* can be distinguished from *Conostegia xalapensis* on the basis of its evident calyptra appendices which are absent in *Conostegia xalapensis*.

#### Specimens examined.

**MEXICO** (fide Schnell). **Chiapas**: km 33 S of SE on the road to Mal Paso, near Tabasco border, Roe et al. 1371 (US).

**BELIZE. Cayo**: Road to Caracol at Guacamallo Bridge, Balick 3104 (NY); along Hummingbird Highway at mile 28.5, Dwyer 11208 (MO, NY); Humming Bird Highway, Gentle 8388 (NY); Mountain Pine Ridge, San Agustin, Lundell 6608, 6770 (NY). **Stann Creek**: beyond Middlesex, Gentle 2758 (NY); 17 miles section Stann Creek Valley, Gentle 9258 (NY).

**GUATEMALA. Izabal**: slopes WNW of (above) El Estor, along margin of open pit nickel mine, Stevens and Martinez 25247 (NY).

**HONDURAS. Olancho**: Montaña La Bellota 20 kms al N.O. de Campamento, Molina 13436 (NY).

### Conostegia povedae

Taxon classificationPlantaeMyrtalesMelastomataceae

(Kriebel & Oviedo) Kriebel comb. nov.

urn:lsid:ipni.org:names:77156270-1

Fig. 186

Conostegia povedae (Kriebel & Oviedo) Kriebel. Basionym: Miconia povedae Kriebel & Oviedo, Phytotaxa 126 (1): 57. 2013. Type: Costa Rica. Puntarenas; Estación Biológica Las Cruces. En el Sendero Gamboa, 1157 m, 5 September 2008, 8.789333N, -82.970250W, F. Oviedo-Brenes 231 (holotype HLDG!, isotype CR!).

#### Description.

Shrub to small tree to 2.5–4 m tall. Young branches tomentose with golden to orange indument consisting of dendritic to stellate trichomes. The interpetiolar line inconspicuous and covered by indument as in the stems. Leaves sessile or subsessile, somewhat anisophyllous. Leaf blades 9–24 × 5–9 cm, 3–5-plinerved, with the innermost pair of veins diverging from the midvein 1.5–7 cm above the base generally in asymmetric fashion, elliptic to obovate, the base attenuate, apex acuminate, the margin subentire to denticulate, adaxially glabrous, abaxially moderately stellate pubescent on tertiary and higher order veins. Inflorescence a terminal and deflexed panicle with dichasial branches, 5–10 cm long, with about 3–7 sessile flowers clustered at the end of the branches; bracts 2, 3–10 mm long, elliptic to ovate, persistent; bracteoles 2, ovate, ca. 1–2 × 0.5–1.25 mm, persistent. Flower buds 4–5 mm long, with the petals forming an acuminate cone when mature. Hypanthium narrowly campanulate, sparsely stellate. Flowers 5(-6) merous, calyx not calyptrate but with the calyx lobes fused and irregularly rupturing in their early stages, the calyx tube 0.3–0.5 mm, the teeth 0.25–0.4 mm long, linear-oblong. Petals 4.5–5.5 × 2–2.5 mm, white, spreading at anthesis, elliptic to elliptic-ovate, apically acute, glabrous. Stamens 10(12), 4–5 mm long, radially arranged around the style, the filament 2.25–2.75 mm long with a geniculation near the apex, white, anthers 1.5–2 × 0.6–0.8 mm, elliptic, somewhat laterally compressed, lacking an appendage or collar at the anther filament junction, yellow, pore ca. 0.15 mm wide, terminating the truncate apex. Ovary 5-locular, half superior, the apex beset with small brown glandular trichomes and elevated into a collar ca. 0.25 mm high around the style. Style ca. 6.4 mm long, straight to slightly curving, the stigma truncate, ca. 0.38 wide, the distance between the anther apex and the stigma ca. 1.5 mm, stigma truncate to capitellate, ca. 0.3 mm wide. Berry and seeds not seen.

**Figure 186.**
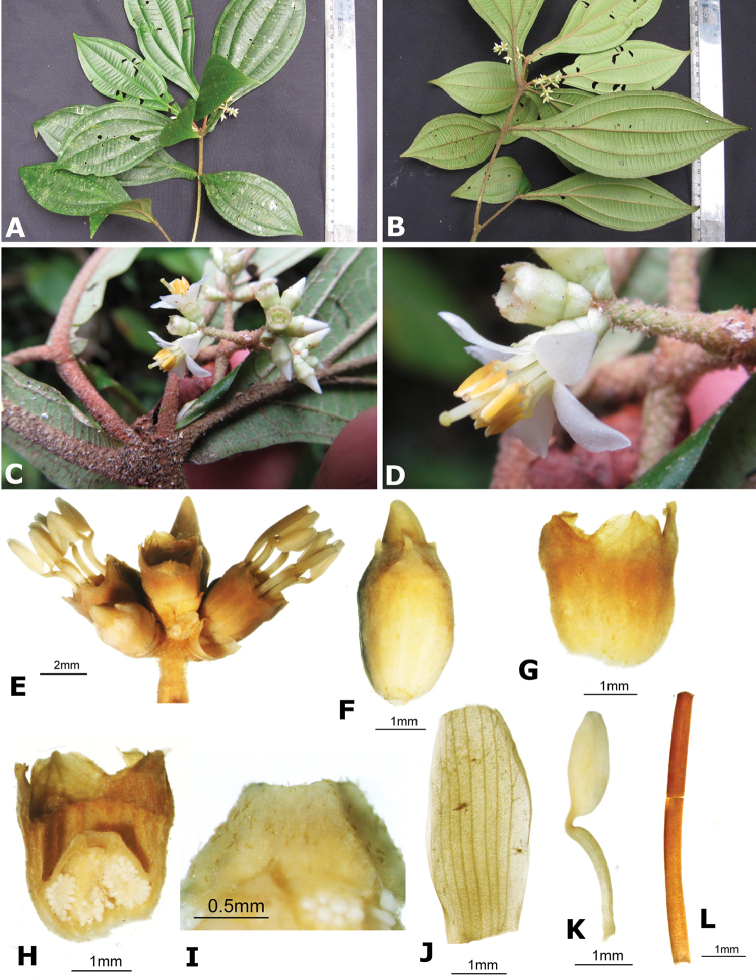
*Conostegia povedae*. **A** Fertile branch and adaxial leaf surfaces **B** Abaxial leaf surfaces **C** Inflorescence showing clustered sessile flowers **D** Close up of the flower **E** Flower cluster terminating an inflorescence branch **F** Flower bud **G** View of the hypanthium from outside, note the acute calyx teeth on the rounded calyx lobes **H** Longitudinal cut of a flower with petals, stamens, and style removed **I** Ovary apex with minute glands **J** Petal **K** Lateral view of the stamen **L** Style. Photographs of the type by Federico Oviedo.

#### Distribution

**Figure 187.**
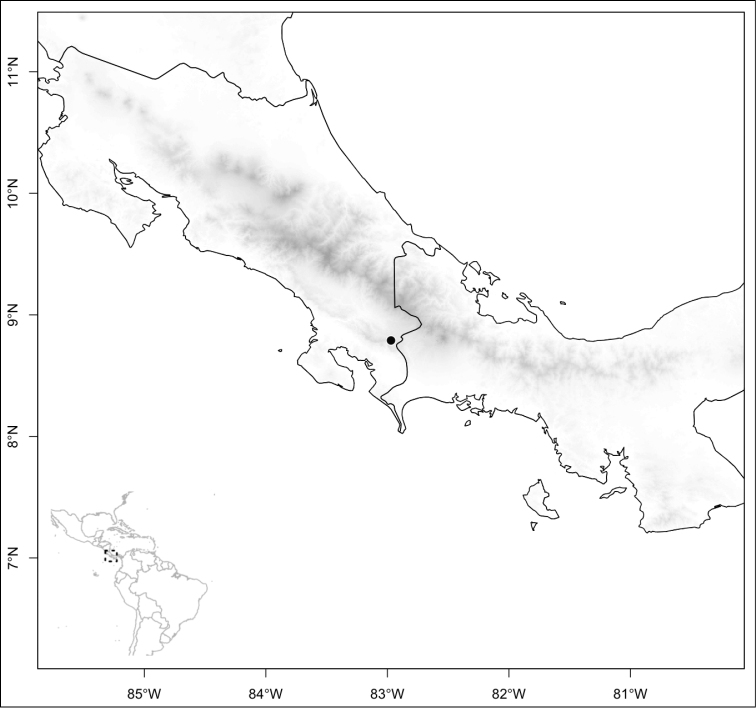
Distribution of *Conostegia povedae*.

(Fig. 187). Endemic to the remaining primary forest in San Vito de Coto Brus, Puntarenas, Costa Rica at 1100–1200 m elevation.

*Conostegia povedae* is a very narrow endemic easy to recognize because of its rusty indument, sessile leaves with attenuate leaf bases and which are strongly plinerved, and sessile flowers in clusters that when in bud have a fused calyx that ruptures irregularly. In their evidently yellow anthers, exserted styles, and geniculate filaments, *Conostegia povedae* shows the typical floral morphology present in section *Geniculatae*.

#### Specimens examined.

**COSTA RICA. Puntarenas**: Estación Biológica Las Cruces, en el Sendero Gamboa, Oviedo-Brenes and Zahawi 1215 (HLDG); en el sendero Melissa, bosque maduro bastante denso, Oviedo-Brenes 1908 (HLDG).

### Conostegia schlimii

Taxon classificationPlantaeMyrtalesMelastomataceae

(Triana) Kriebel comb. nov.

urn:lsid:ipni.org:names:77156271-1

Fig. 188

Conostegia schlimii (Triana) Kriebel. Basionym: Miconia schlimii Triana, Trans. Linn. Soc. London 28(1): 102. 1872. Lectotype (designated here): Colombia. Nouvelle Grenade: Santa Marta, 1852, L. Schlim 903 (lectotype: BM!, isolectotypes K!).
Acinodendron schlimii (Triana) Kuntze, Revis. Gen. Pl. 2: 952. 1891. Type: Colombia. Nouvelle Grenade: Santa Marta, 1852, L. Schlim 903 (lectotype: BM!, isolectotypes: K!).
Conostegia dolichostylis Donn. Sm, Bot. Gaz. 42(4): 294. 1906. Type: Costa Rica. Puntarenas: in silvis ad Buenos Aires, Feb 1892, A. Tonduz 4943 (holotype: US!).

#### Description.

Shrubs to trees 1.5–8(-14) m tall with flattened stems in newer branches that are densely covered with ferrugineous stellate hairs often intermixed with plumose hairs; the nodal line inconspicuous. Leaves of a pair equal to unequal in length. Petiole 1–4.8 cm. Leaf blades 5–25.8 × 2–10.1 cm, 3–5(-7)-plinerved, with the innermost pair of primary veins diverging from the midvein 0.8–4.5 cm above the base in opposite or commonly alternate fashion, narrowly elliptic-lanceolate to elliptic-ovate, the base acute to obtuse but sometimes varying to asymmetrical, the apex acuminate, the margin obscurely to conspicuously undulate-denticulate, the adaxial surface glabrous or with few trichomes on the main veins, the abaxial surface covered with ferrugineous stellate hairs often intermixed with plumose hairs on the primary veins. Inflorescence a terminal few flowered panicle 2–5.2 cm long branching at or above the base, accessory branches absent, the rachis with a ferrugineus indument like the young stems, bracteoles 1–3 mm, linear, deciduous. Pedicel 0.5–2 mm. Floral buds 6.5–7.5 × 4–5 mm. Flowers 5-merous, calyx not calyptrate or fused in bud, stellulate within, the hypanthium 3.75–4.25 × 5.25–5.5 mm, slightly constricted at the torus especially when going into fruit, ferrugineus, calyx lobes flange-like, 1–1.5 mm long, calyx teeth small and tuberculate, barely exceeding the calyx lobes. Petals 9–14 × 8–10 mm, white, obovate, spreading at anthesis, rounded-emarginate at the apex, glabrous on both surfaces. Stamens 10, 9.5–10.5 mm, radially arranged in the middle of the flower, the filaments 4.75–5.25 mm, white, anthers 4.5–5 × 1.25–1.75 mm, oblong, yellow, the pore 0.15–0.2 mm, ventrally inclined. Ovary 5-locular, inferior, the apex shallowly fluted and glabrous. Style 12–14.5 mm, straight or strongly bent, vertical distance between the anthers and stigma ca. 1.5–3 mm, horizontal distance 0–3 mm, stigma capitate, ca. 1 mm wide. Berry 8–10 × 8–10 mm, purple. Seeds 0.45–0.7 mm long, deltoid, smooth and angulate.

**Figure 188.**
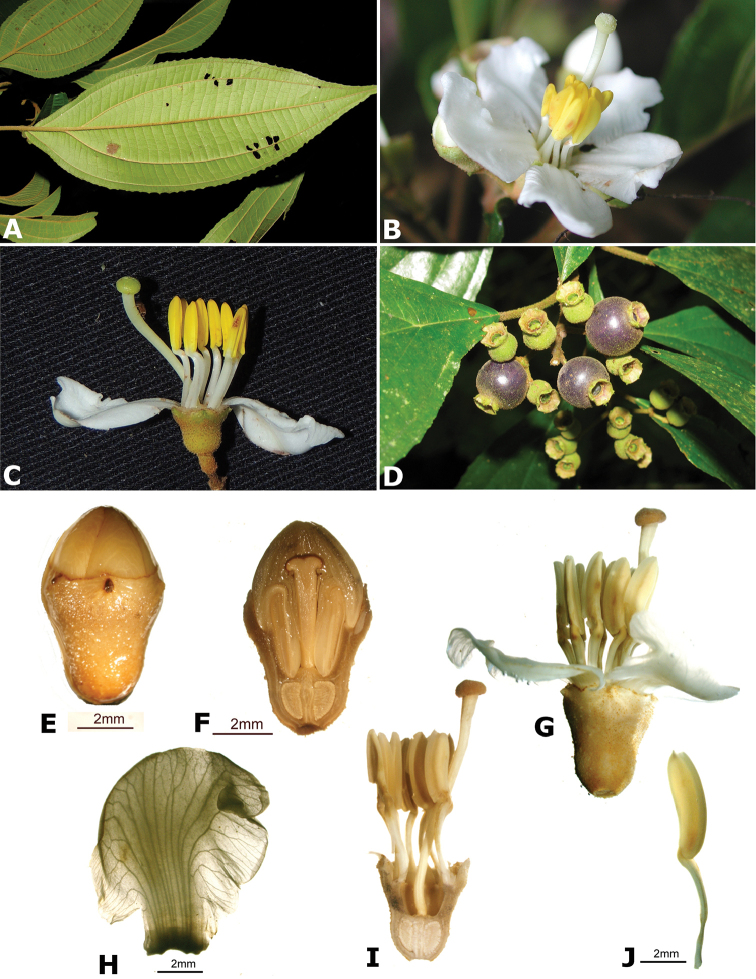
*Conostegia schlimii*. **A** Leaf abaxial surface **B–C** Flower **D** Infructescence **E** Flower bud **F** Longitudinal section of a flower bud **G** Pickled flower **H** Petal **I** Longitudinal section of a flower. Note inferior ovary **J** Stamen. Photo **A** taken by Reinaldo Aguilar and vouchered *R. Aguilar 10854* **C, E–J** of specimen vouchered *R. Kriebel et al 5614* **B** from *R. Kriebel et al 5095*, and **D** from *R. Kriebel et al 5329*.

#### Distribution

**Figure 189.**
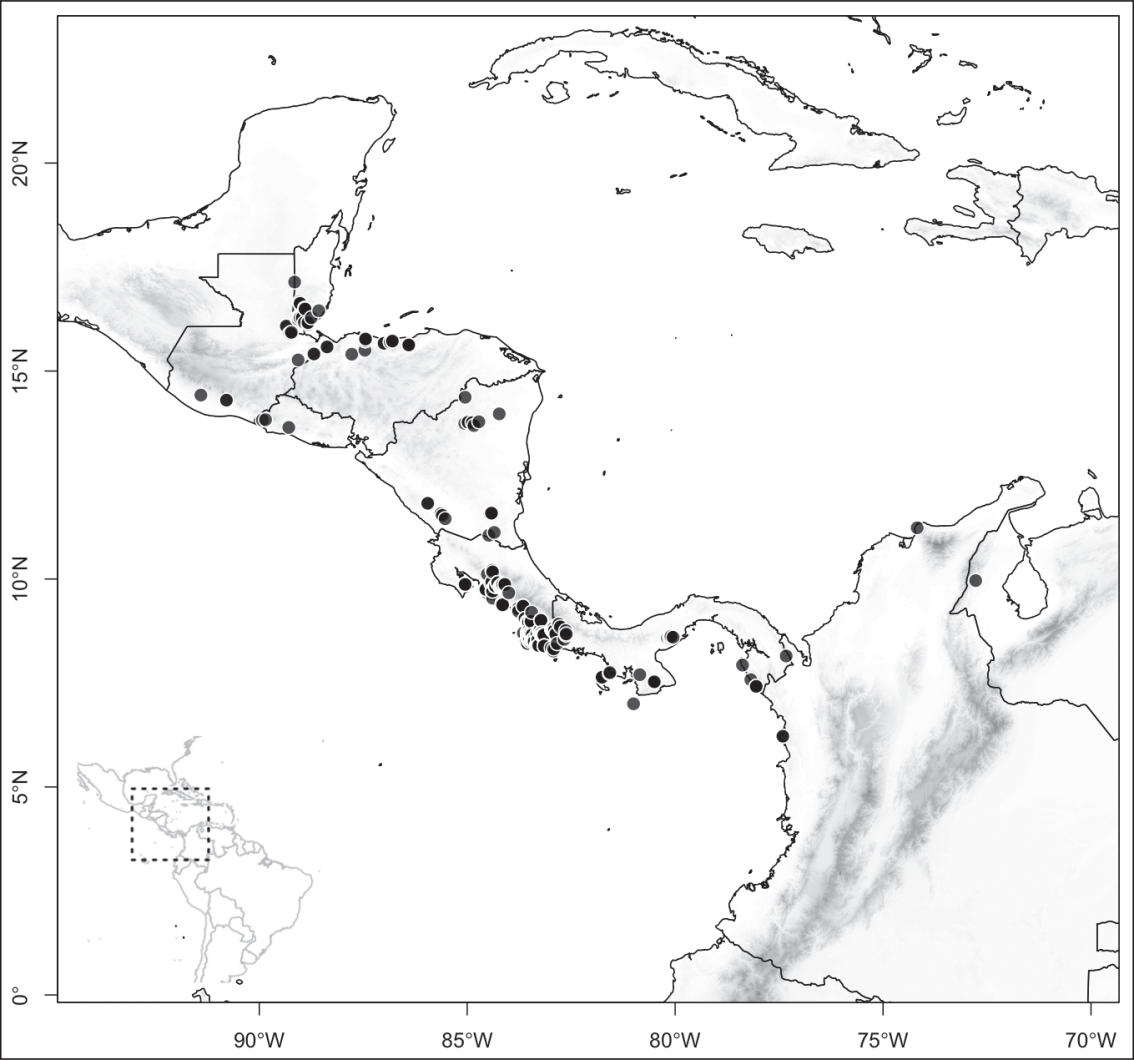
Distribution of *Conostegia schlimii*.

(Fig. 189). Belize, El Salvador, Guatemala, Honduras, Costa Rica, Nicaragua, Panama, Colombia, and northern Venezuela at 0–1200(-2000) m in elevation.

*Conostegia schlimii* is one of the most common species of *Conostegia* and an easy one to recognize. It has a conspicuous rusty indument on branch apices, the leaves are evidently plinerved and frequently with asymmetrically arising primary veins, as well as an undulate-denticulate margin. The flowers are quite large and have distinctive basally clawed petals, large bright yellow anthers and a capitate stigma. It is one of the largest flowered species in the genus and one of the few large flowered species not to be pleiostemonous. Its berries are also quite large. Flowers of this species have been observed being buzzed by *Melipona costaricensis* in the Osa Peninsula of Costa Rica. For such a common species in the Pacific slope of Costa Rica as well as being present towards the Caribbean slope in Nicaragua, Honduras, Guatemala and Belize, it is suprising it is absent in the Caribbean slope of Costa Rica. Based on morphology, *Conostegia schlimii* is hard to confuse with any other taxon. Perhaps the one that most closely resembles *Conostegia schlimii*, based on floral size and general morphology is *Conostegia incurva*. The latter differs in its leaf abaxial surface being covered with indument and most noticeably in its claw like, conspicuous, calyx teeth.

#### Specimens examined.

**COSTA RICA. Alajuela**: San Gerardo de San Ramón, Brenes 1903 (CR, NY). Bella Vista De Zarcero, Smith 149 (CR, MO, NY). **Cartago**: Turrialba, Reventazón, DeWolf 236 (NY). **Puntarenas**: Distrito Sierpe, R. F. Golfo Dulce, Los Charcos 1 km. al este del centro del pueblo Banegas, Estación Biológica Los Charcos de Osa, Aguilar 10854 (NY); Estación Biológica Las Cruces, road between the station and San Vito, Boyle and Michelangeli 6228 (NY); Steep forested slopes above Golfito along the trail to the television tower, Burger and Matta 4743 (NY); R. N. V. S. Golfito, Martén 775 (CR, NY); in forest on hills near Volcán de Buenos Aires, Williams 19251 (CR, NY). **San José**: P.N. Carara Hills at SW part of Montañas de Jamaica ca. 2.5 km NE of Bijagual de Turrubares, Carara Reserve, Grayum et sl. 5462 (MO, NY); Zona Protectora La Cangreja, forests along Río Negro ca. 1.5 km E of Santa Rosa de Puriscal, Grayum 8327 (NY); Acosta, Salvaje, Cerros de Escazú, Kriebel and Cordero 1316 (NY); Camino entre Puriscal y P. N. La Cangreja, Kriebel 5329 (INB, NY). Perez Zeledón, Vicinity of El General, Skutch 4320, 4867 (MO, NY).

**BELIZE. Cayo**: 4 miles south of Grand de Oro on road to La Flor, Dwyer 10904 (NY). **Toledo**: Columbia Forest Station near entrance, Dwyer 11090 (MO, NY); in broken bridge, Criquetrosa, Punta Gorda-San Antonio Road, Gentle 4805 (NY); BARC farm about 1 km up the Columbia River from San Pedro, Houck 3971 (NY).

**EL SALVADOR. Ahuachapán**: Vicinity of Ahuachapán, Standley 20270 (NY). **La Libertad**: Vicinity of Santa Tecla, Standley 23072 (NY).

**GUATEMALA. Escuintla**: Concepción, Smith 2212 (MO, NY); Along or near Río Michatoya southeast of Escuintla, Standley 89081 (NY). **Izabal**: Sierra Caral, Quebrada atravesada por el sendero al noreste de la casa de investigadores, hacia la Finca Bonanza, Kriebel et al. 5614 (USCG, NY); Vicinity of Quiriguá, Standley 24560 (NY). Suchitepéquez: Rio Sis, Smith 2655 (NY).

**HONDURAS. Atlántida**: along Tela River between Peñas Gordas and Tela, Molina and Molina 25664 (MO, NY). **La Ceiba**: in forest slopes of Mt. Cangrejal, Yuncker 8780 (MO, NY). YORO: alrededores de El Progreso, Molina 6796 (NY).

**NICARAGUA. Granada**: Volcán Mombacho, por las faldas del lado E, camino de Las Delicias, Moreno 24275 (CAS). **Rivas**: Isla Ometepe, Volcán Concepción, cafetales ”La Flor”, Robleto 1015 (CAS). Zelaya: Forest bridge over Río Almacén 8 km SW of Nueva Guinea, Nee and Vega 27935 (NY).

**PANAMA. Chiriquí**: Progreso, Cooper and Slater 297 (NY); Roadside from Paso Canoas to Canos Gordas 17 mi from Paso Canoas, Liesner 225 (NY); between Concepcion and El Volcán, White 312 (NY). **Coclé**: Between Las Margaritas and El Valle, Woodson, Allen and Seibert 1241 (NY); Hills south of El Valle de Anton, Allen 2496 (NY). **Darién**: SE of Punta Guayabo Grande, Antonio and Hahn 4382 (NY). **Los Santos**: Loma Prieta, Cerro Grande, Lewis et al. 2205 (MO, NY). **Veraguas**: Isla Coiba, Playa Rosario, Galdames et al. 2583 (MO, NY).

**COLOMBIA. Chocó**: vicinity of Bahía Solano tropical wet forest near sea level cli?s along coast, Gentry and Fallen 17188 (NY). **Santa Marta**: near Cacagualito, Santa Marta, Smith 3 (NY).

**VENEZUELA** (fide Almeda)**. Zulia**: Sierra Perijá, Steyermark 105793 (US).

### Conostegia shattuckii

Taxon classificationPlantaeMyrtalesMelastomataceae

(Standl.) Kriebel comb. nov.

urn:lsid:ipni.org:names:77156272-1

Fig. 190

Conostegia shattuckii (Standl.) Kriebel. Basionym: Miconia shattuckii Standl., Contr. Arnold. Arbor. 5: 119, pl. 16. 1933. Type. Panamá: Barro Colorado Island, Canal Zone, 24 October 1931, O. Shattuck 335 (holotype: F!, isotype: MO!).

#### Description.

Shrubs 1–4 m tall with terete stems that are moderately to densely covered with rusty barbellate or plumose hairs and moderately underlain with minute oblong glands; the nodal line not evident from the copious indument. Leaves of a pair mostly equal in length. Petiole 0.5–3 cm. Leaf blades 9.5–40 × 7–20, 5–7 nerved, elliptic-ovate to ovate, base cordate, apex acuminate, the margin undulate-denticulate, adaxially with simple hairs on the veins when young but becoming glabrous with age, abaxially with a mixture of simple, barbellate and minute glandular hairs. Inflorescence a terminal panicle 6–15 cm long, accessory branches absent, rachis copiously covered with rusty barbellate hairs, bracteoles 0.5–1 mm long, subulate, persistent. Pedicels about 1.5–2.5 mm. Hypanthium campanulate 2–2.25 × 1.25–1.75 mm, sparingly covered with barbellate, plumose and glandular hairs, the torus and inner hypanthium walls glandular puberulent. Flowers 5-merous, calyx not calyptrate nor fused in bud, the undulate apiculate lobes 0.25–0.5 mm long, the exterior calyx teeth tuberculate, 0.25 mm long. Petals 2.5–5 × 1.5–3 mm, ovate-oblong, translucent white, glabrous, not observed at anthesis but petals apparently closing after anthesis, emarginate. Stamens 10, 3.75–4.5 mm long, radially arranged around the style, the filament 1.75–2 mm long with a geniculation near the apex, translucent white, anthers 2–2.25 × 0.4–0.6 mm, linear-oblong, somewhat laterally compressed, cream yellow, the pore ca. 0.17 mm, truncate. Ovary 5-locular, 3/4 inferior, the apex more or less flat and glandular puberulent. Style 6–6.5 mm long, glandular puberulent at the base, straight but gently curving, vertical distance between the anther apex and the stigma 1.25–1.75 mm, horizontal distance absent, stigma truncate, 0.4–0.55 mm wide. Berry pink when immature and turning purple black at maturity, 5–6 × 5–6 mm. Seeds broadly deltoid in outline, smooth and rounded-angulate, the raphe expanded and sunken, 0.5–0.8 mm long.

**Figure 190.**
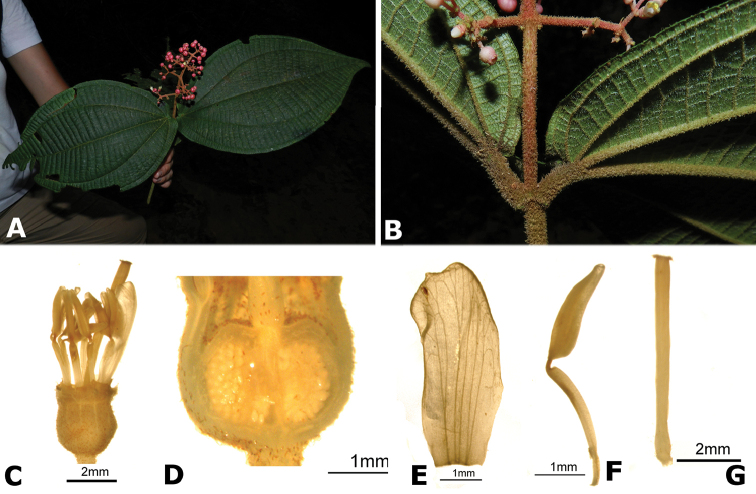
*Conostegia shattuckii*. **A** Habit **B** Close up of internode **C** Pickled flower **D** Longitudinal section of hypanthium showing brown glands on the apex of the ovary **E** Petal **F** Stamen. Photos of specimen vouchered *R. Kriebel and J. Burke 5578*.

#### Distribution

**Figure 191.**
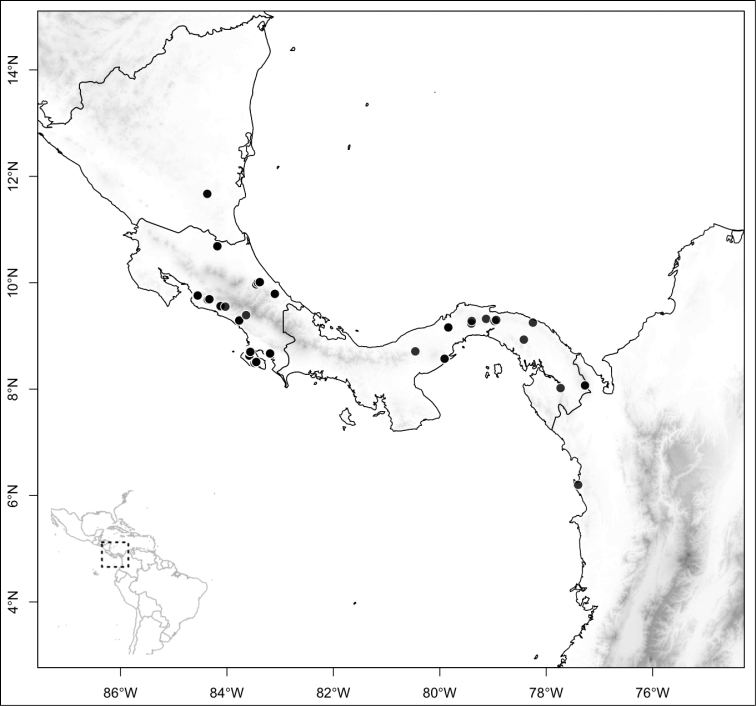
Distribution of *Conostegia shattuckii*.

(Fig. 191). Nicaragua, Costa Rica, Panama and Colombia, 0–950 m in elevation.

*Conostegia shattuckii* is readily recognized on the basis of its nerved leaves with a cordate base, and its small flowers with yellow anthers and exserted styles. Among species of section *Geniculatae*, *Conostegia shattuckii* is one of the few that is not plinerved. The style measurement given here is much longer here than in Flora Mesoamericana (Almeda, 2009). This is perhaps due to fresh versus dry measured material.

#### Specimens examined.

**NICARAGUA. Zelaya**: Nueva Guinea, Colonia Yolaina, Arachistain 3075 (CAS).

**COSTA RICA. Alajuela**: ca. 7 km NE de Boca Tapada, Laguna del Lagarto Lodge, Hammel 20345 (INB). **Limón**: 200 mts. aguas abajo de la confluencia de Quebrada Canabral con Río Barbilla, margen derecha, siguiendo el curso de la Quebrada Camagre, Herrera 2293 (INB). **Puntarenas**: Rancho Quemado, Tierra de Conservacion, Península de Osa, Aguilar 1971 (INB).

**PANAMA. Panamá**: Parque Nacional Chagres, Sendero El Mono, adentro de la urbanización Altos de Cerro Azul, Kriebel and Burke 5688 (NY).

**COLOMBIA. Chocó**: Municipio Bahía Solano/Ciudad Mutis, corregimiento Ciudad Mutis, Quebrada Seca, Almeda et al. 10468 (CAS).

### Conostegia speciosa

Taxon classificationPlantaeMyrtalesMelastomataceae

Naudin

Fig. 192

Conostegia speciosa Naudin, Ann. Sc. Nat. Bot. ser. 3 16: 109. 1850. Type: Panama. E. Duchassaing s.n. (lectotype: BR!, designated here; isolectotypes: GOET, ?F [putative fragment of P specimen; however, Almeda did not find syntypical material at P, pers. comm. 1995 in Schnell (1996)]). Other syntype:—Nova Granata, Goudot s.n. (not seen).

#### Description.

Shrubs to small trees 1.2–7 m tall with terete to slightly rectangular stems that are moderately to densely covered with stellate and stipitate stellate hairs, the stipe ca. 0.5–1.75 mm; rarely some simple hairs intermixed; the nodal line present and covered by indument. Leaves of a pair equal to unequal in length. Petioles 0.4–4.5 cm. Leaf blades 8.6–22.3 × 4–11.1 cm, 5–7 plinerved, with the innermost pair of primary veins diverging from the mid vein 1–3 cm above the base in mostly opposite to sub opposite fashion, broadly elliptic to ovate, or oblong-ovate, the base acute broadly rounded, the apex attenuate to long acuminate, the margin denticulate or unevenly dentate. Inflorescence a terminal panicle small and closed but expanding as fruit mature, 2.3–17.9 cm long, accessory branches present, bracts linear, to 1.5 cm long, deciduous, the rachis purple pubescent, linear, bracteoles 2–3 mm, early deciduous. Pedicels to 3 mm long. Flowers (5-)6–7 merous, ovoid, calyptrate. Floral buds 5–9 × 2.5–5.5, rounded at the base, the apex subacute, not constricted in the middle; the hypanthium 3.5–4 × 3–3.5 mm, purple pubescent with sessile and stipitate stellate trichomes. Petals 5–7.25 × 4–5 mm, pink, lavender or almost white, ovate to obovate, apically emarginate, glabrous. Stamens (10-)12–14(-15), 4–6.5 mm long, slightly bilaterally arranged, the filaments 2–3.5 mm, white, anthers 2–3 × 0.75 mm, linear or oblong, straight or slightly curved, yellow, laterally compressed, the pore 0.1–0.2 mm wide, ventrally subterminal. Ovary (5-)6(-8) locular, inferior, apically glabrous and not forming an evident collar around the style. Style 2.5–4 mm long, strongly curved below the stigma, the distance of the anther pore to the stigma -1.75 – -0.75; stigma capitate, 1–1.25 mm wide. Berry 7–10 × 7–10 mm, dark purple. Seeds 0.9–1.3 mm, triangular and flat or slightly curved, the testa glossy.

**Figure 192.**
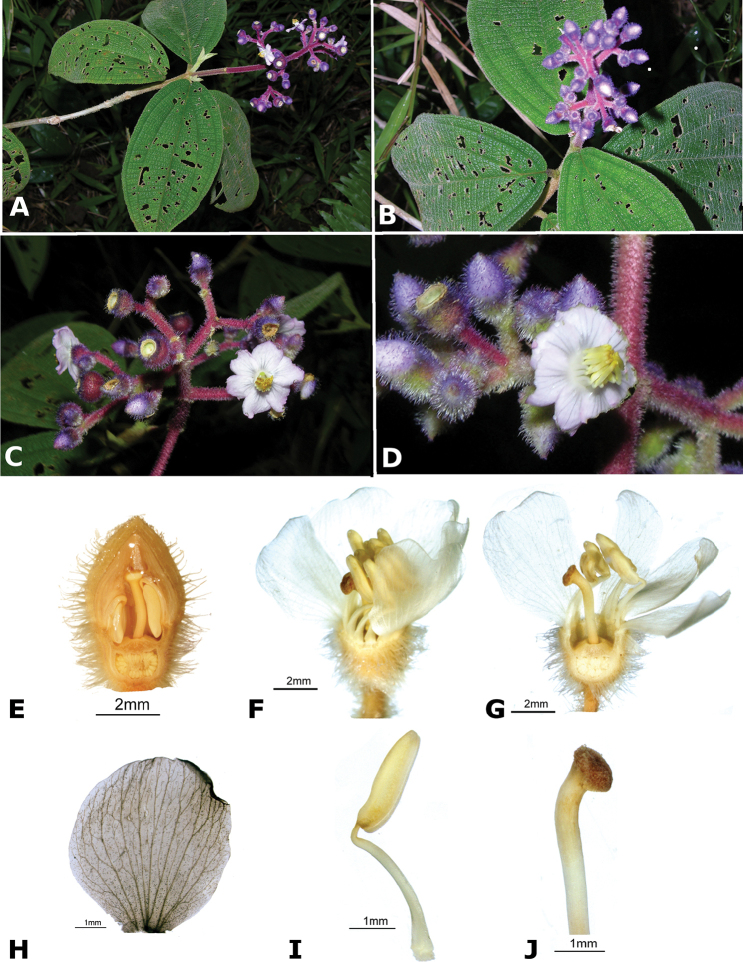
*Conostegia speciosa*. **A** Habit **B** Close up of internode **C** Pickled flower **D** Longitudinal section of hypanthium showing brown glands on the apex of the ovary **E** Petal **F** Stamen. Photos **A–D** of specimen vouchered *R. Kriebel 5489* **E–J** vouchered *R. Kriebel 5677*.

#### Distribution

**Figure 193.**
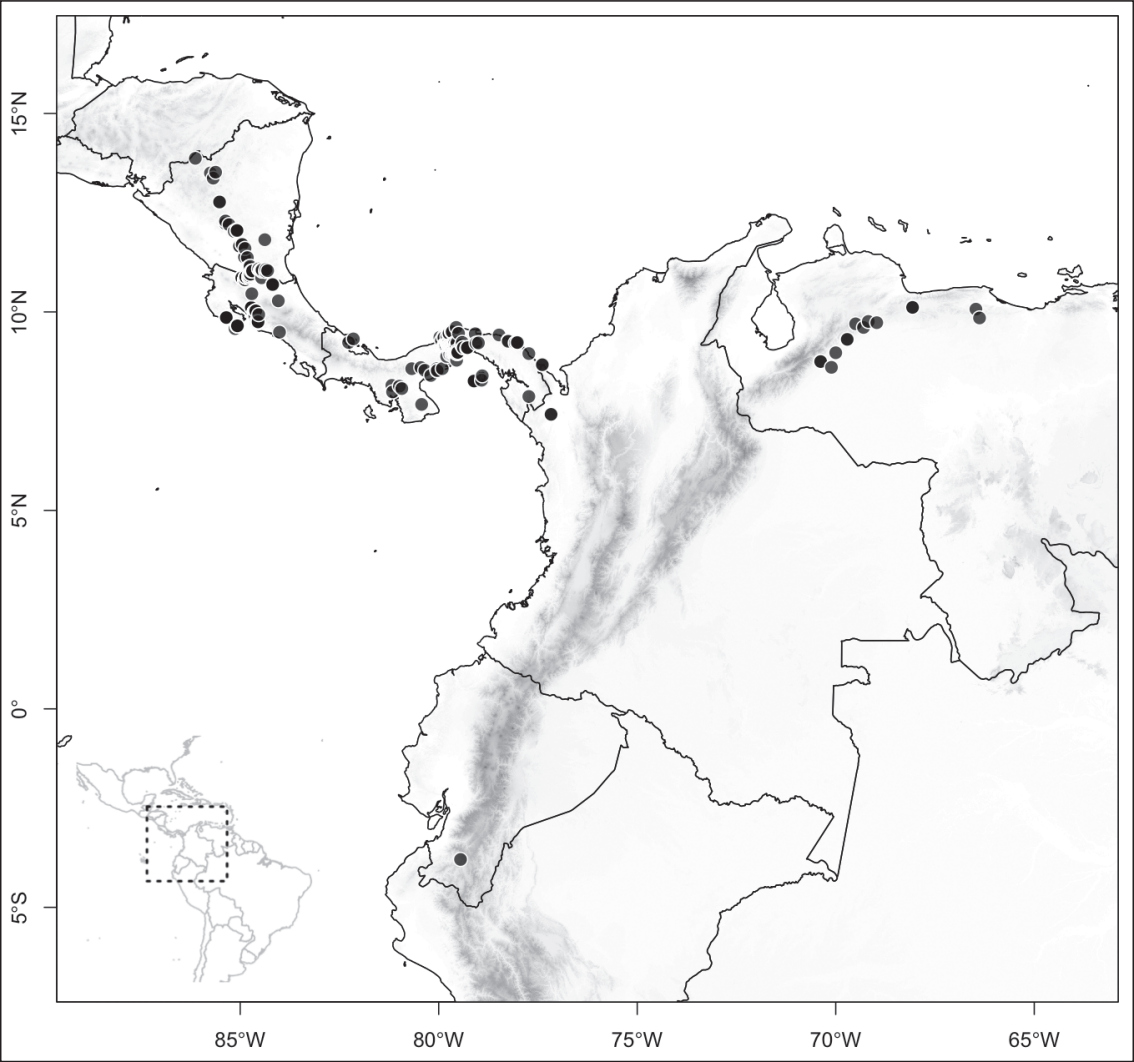
Distribution of *Conostegia speciosa*.

(Fig. 193). Occurring in disjunct populations in Nicaragua, Costa Rica, Panama, Santa Marta in Colombia, Loja in Ecuador, and in north-central Venezuela, 0–1000 m in elevation.

*Conostegia speciosa* is a distinctive species based on its dense hirsute indument of stipitate stellate and simple trichomes. Also, the indument on its inflorescences tends to be bright purple. Its style is short like that of its close relatives *Conostegia subcrustulata* and *Conostegia xalapensis* and like the latter taxa lacks a stele inside the style. Other species with short styles which are restricted to section *Conostegia* have a stele inside the style. Schnell (1996) argued that *Conostegia speciosa* was the most abundant weed of the genus in Central Panama in habitats that would probably be occupied by *Conostegia icosandra*, *Conostegia polyandra*, *Conostegia subcrustulata*, or *Conostegia xalapensis*. For this reason, Schnell (1996) suggested they might be ecological replacements of each other. Of all these taxa, *Conostegia speciosa* is the one that inhabits the driest areas. Schnell (1996) also reported possible hybrids between *Conostegia speciosa* and *Conostegia xalapensis* form the Canal Zone in Panama and from Santa Marta, Colombia, and tested their pollen viability. He found that the purported hybrids in fact had a high percentage of non viable pollen grains. Lastly Schnell (1996) suggested some specimens show introgression from *Conostegia subcrustulata*.

#### Specimens examined.

**NICARAGUA. Rio San Juan**: San Carlos, Atwood 5328 (MO, NY); San Bartolo, Robbins and Seymour 6173 (NY); San Carlos, Smith 1893 (NY).

**COSTA RICA. Alajuela**: Vicinity of Los Chiles, Río Frío, Holm and Iltis 768 (NY); Guatuso, La Garroba (Sector Buenavista), Finca La Garroba (pinéra parche grande), Kriebel et al. 5489 (NY). **Puntarenas**: About 4–5 km N of Miramar on C.R. number 144, Almeda, Wilbur and Daniel 2863 (CR, MO, NY).

**PANAMA. Canal Zone**: vicinity of Gamboa, Allen 1970 (NY); Pipeline Road within 5 mi. of Gamboa gate, D’Arcy and D’Arcy 6014 (NY); Corozal Cemetery, Dwyer 2584 (NY); Madden Forest Las Cruces Trail, Gentry 1379 (MO, NY); Pipeline road near Río Agua Salud, Kennedy 1808 (MO, NY); Ancon Hill, Killip 3011 (NY); Parque Nacional Chagres, al lado del camino hacia sendero El Mono, Kriebel and Burke 5677 (PMA, NY); Las Sabanas, Pittier 6709 (NY); Vicinity of Madden Dam near Río Chagres, Seibert 551 (NY); Along road K-9, Smith and Smith 3266 (NY); Fort Clayton on road C-15 at tower, Tyson 1784 (NY). **Coclé**: Llano Bonito north of Las Margaritas, Seibert 532 (NY); Vicinity of Miraflores, White 137 (NY). **Panamá**: Vicinity of Pacora, Allen 1118 (NY); Vicinity of Río Pacora, savanas along Panama National Highway east of Panama City, Bartlett and Lasser 16468 (MO, NY); Barro Colorado Island, Woodson and Schery 972 (NY); Isla Taboga, Woodson, Allen and Seibert 1524 (NY); thickets and forests near Arraiján, Woodson et al. 1361 (NY). **San Jose Island**: San José Island, Pearl Archipelago, Along Canyon Road, Erlanson 567 (NY); Perlas Archipelago, Gulf of Panama (about 55 miles SSE of Balboa), M-Area road, San Jose Island, Perlas Archipelago, Gulf of Panama (about 55 miles SSE of Balboa), Johnston 446 (NY); Junction of Loops and Canyon Road, Johnston 1166 (MO, NY). **Veraguas**: Río de Jesus, Dwyer 1317 (NY).

**COLOMBIA. Santa Marta**: near Cacagualito, Smith 5 (NY).

**ECUADOR. Loja**: Horsetrail NE of Recinto El Prado on road Portovelo-Loja, Harling 27098 (MO, NY).

**VENEZUELA. Barinas**: 15 km from Barinas along road to Barinitas, Breteler 4212 (MO, NY); caserío El Pescado, río Yuca, Dtto. Obispos, Stergios 8642 (NY). Carabobo: alrededores de Tucuyito, Aristeguieta 2368 (NY); alrededores de Tocuyito, distrito Valencia, Saer deHeguert 836 (NY). **Lara**: Presa Yacambú, Orilla Izquierda del Río Yacambú, 500 m aguas abajo del tapón de la presa, Michelangeli and Gallagher 738 (NY). **Miranda**: Carretera Parque Nacional de Guatopo-Altagracia de Orituco, Aristeguieta 6383 (NY). **Portuguesa**: Caserío Villa Rosa 20 km al este de Biscucuy, Aymard et al. 3657 (NY); a lo largo de la carretera Gavilán-Mijagual-Mesa de Bucaral 15 km al S-SE de Biscucuy, Aymard, Cuello and Desantiago 3708 (NY); Agua Sucia 4-8 km on road to Las Panelas, 10 km from junction with Guanare-Barinas road, Hahn and Grifo 4927 (NY); Hacienda Los Caminos, 10 km al N de Araure en la vía Camburito, Potreros al Oeste del río de los pozos al N del campamento La Llanada, Michelangeli, Davalos and Cibois 810 (NY); Municipio Guanare, río Las Marías, camino hacia San José de Montaña, Stergios and Ortega 2940 (NY); along woodland stream between Las Palmas and Paraíso de Chabasquen, Steyermark and Rabe 97417 (NY). **Zulia**: cuenca del río Guasare, No 1. (La Yolanda), entre el Destacamento y “La Piscina” (Caño Rechazado) en la Hacienda Doná Clara (5 km al NE del Destacamento), Bunting et al. 12740 (NY).

### Conostegia subcrustulata

Taxon classificationPlantaeMyrtalesMelastomataceae

(Beurl.) Triana

Fig. 194

Conostegia subcrustulata (Beurl.) Triana, Trans. Linn. Soc. 28: 98. 1071. Miconia subcrustulata Beurl., Prim. Fl. Portob. 130. 1856. Type: Panama. Porto Bello in montibus, April 1826, Billberg s.n. (lectotype: S!, designated here).
Conostegia purpurea Grisebach, Bonplandia 6: 6. 1858. Type: Panama. in ripas fluminis, Chagres, E. Duchassaing s.n. (holotype: GOET!).

#### Description.

Shrub to small tree 1–5 m tall with tetragonal stems at first but soon becoming terete that are tomentulose, covered with small stellate hairs and sometimes also more developed somewhat branched or roughened trichomes; the nodal line present but slight and obscured by indument. Leaves of a pair equal to somewhat unequal in length. Petioles 0.5–12 cm. Leaf blades 5–20.7 × 3.1–12 cm, 5–9 plinerved, with the innermost pair of primary veins diverging from the mid vein 0.5–3 cm above the base in mostly opposite to sub opposite fashion, ovate, the base rounded to cordate, the apex acute or short acuminate, the margin serrate and short ciliate, the adaxial surface sparsely hirsute to glabrous,the abaxial surface tomentulose covered with small stellate hairs and sometimes with also more developed somewhat branched or roughened trichomes intermixed. Inflorescence a terminal panicle 6.7–30 cm long branching above the base, accessory branches present, the rachis covered with stellate hairs, bracteoles to 1.5 mm, linear, deciduous. The pedicels 0.7–2 mm. Flowers 5(-6) merous, calyptrate. Floral buds 3–6 × 2–3.5 mm, pyriform, the base rounded, the apex acute to apiculate and with teeth at the tip, constricted at the middle, hypanthial and calyptrate portions well differentiated, the hypanthium 2–3.5 × 2–3 mm, glabrescent, with stellate trichomes. Petals 4–5 × 2.5–3.25 mm, pink, narrowly obovate, spreading, glabrous, apically acute. Stamens 10 (-12), 4–5 mm, somewhat zygomorphic, the filament 2–2.75 mm, white, geniculate near the apex, anthers 1.8–2.5 × 0.5–1 mm, linear to elliptic, yellow, laterally compressed, the pore 0.2–0.3 mm wide, ventrally inclined. Ovary (4-)5(-6) locular, inferior, apically glabrous and not forming an evident collar around the style. The style 4–4.5 mm, straight but abruptly bent just below the stigma, vertical distance of the anther pore to the stigma -1 – -0.25 mm, stigma clavate to capitellate, 0.65–1 mm wide. Berry 4–6 × 4–6 mm, at first pink but turning dark purple to black. Seeds 1–1.5 mm long, narrowly wedge shaped, glossy on one side and smooth on the other.

**Figure 194.**
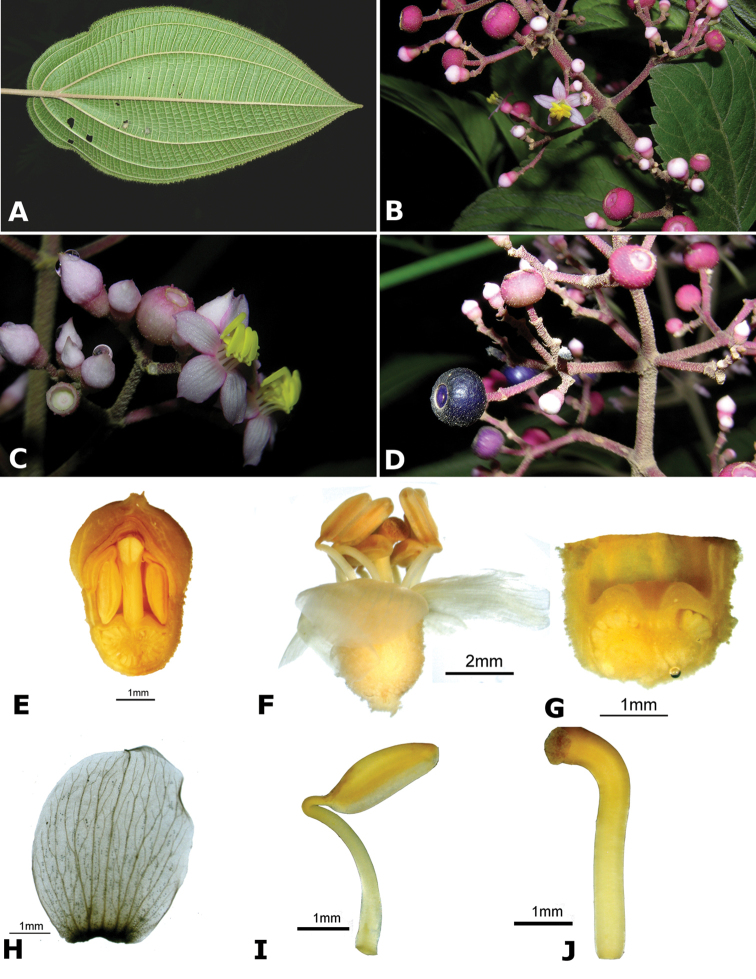
*Conostegia subcrustulata*. **A** Leaf abaxial surface **B** Inflorescence **C** Close up of flower **D** Infructescence **E** Longitudinal section of flower bud **F** Pickled flower **G** Longitudinal section of a flower at anthesis with stamens, style and petals removed **H** Petal **I** Stamen **J** Style. Photos of specimen vouchered *R. Kriebel 5333*.

#### Distribution.

**Figure 195.**
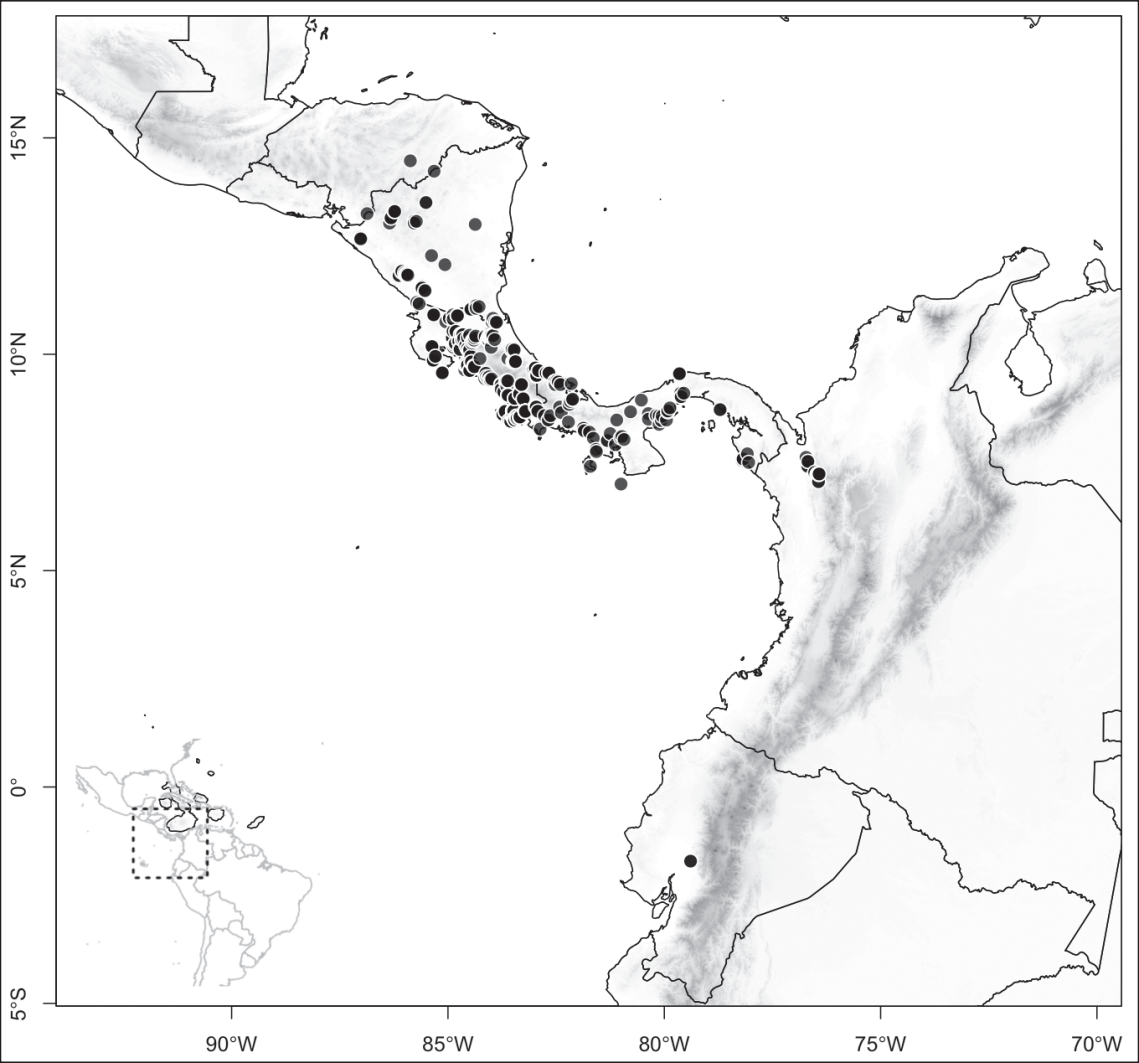
Distribution of *Conostegia subcrustulata*.

Nicaragua, Costa Rica, Panama, Colombia, and a locality in central Ecuador, mostly at low elevations but occasionally to 1500 m in elevation. Fig. 195.

*Conostegia subcrustulata* is a weedy species frequently found in open areas and on roadsides. It is easy to recognize because of its membranaceous, broad leaves with serrate and ciliate margins, and whitish stellate trichomes on twigs and inflorescences. The latter are generally pink, as are the hypanthia and calyptras. The calyptra in this species tends to have small appendage or calyx teeth at the apex. The calyptra is quite thick but lacks sclereids. The filaments are evidently geniculate which agrees with its phylogenetic placement in the *Geniculatae* clade. In the Osa Peninsula, Costa Rica, flowers of this species were found to be quite variable. In particular, the positioning of the stamens with respect to the style varies almost flower to flower. The short style slightly bent apically might contribute to this apparent intrafloral interference.

Schnell reports on observation by Dent-Acosta who at La Selva observed a species of *Melipona* as the most common visitor of *Conostegia subcrustulata* flowers between 8:30 and 10:30 am. Dent-Acosta also reported on pollination of all flowers but fruit set of a couple of berries a day per inflorescence. Lastly, Dent-Acosta noted that ovaries have about 200 ovules and stigmas received more than 200 grains but only up to seventy seeds were produced in each one. Schnell (1996) suggests this is either a case of a self-incompatibility mechanism or self limitation of seed and fruit development. He further suggests it might be an example of fruit and or seed abortion. I have also observed *Melipona costaricensis* as a common visitor of *Conostegia subcrustulata* at Los Charcos, Osa Peninsula, Costa Rica.

Hybrids with *Conostegia xalapensis* were reported by Schnell (1996) from El Valle, Coclé, Panama. Schnell tested the viability of these potential hybrids and found very low viability suggesting they were indeed hybrids. I have collected a specimen which appears to be a hybrid with *Conostegia xalapensis* in Sarapiquí, Costa Rica. The specimen looks like *Conostegia subcrustulata* but has an abaxial leaf surface more like *Conostegia xalapensis* in its heavy indument. Schnell also reports possible hybridization with *Conostegia speciosa* from two Costa Rican specimens, but the testing of pollen viability resulted in normally filled grains.

Schnell (1996) reported the findings of Ellison et al. (1993) and colleagues who studied the germination of seedling ecology. They reported that the seeds handled dry storage and had about a 50% germination rate.

#### Specimens examined.

**HONDURAS (fide Schnell). Olancho**: between Poncaya and Río del Incendio, Blackmore and Heath 2078.

**NICARAGUA. Estelí**: common on moist Estelí river bank 5 kms from Estelí town, Molina 23012 (MO, NY). **Granada**: Catarina, Baker 2223 (MO, NY). Matagalpa: Cut-over hills about 15 km. northeast of Matagalpa along Río Las Cañas, Williams et al. 27545 (MO, NY).

**COSTA RICA. Alajuela**: La Palma de San Ramón, Brenes 5752 (CR, NY); San Pedro de San Ramón, Brenes 5083, 22291 (NY); en la orilla oriental del Río San Carlos, en jabillos, Jiménez 1795 (CR, NY); Ciudad Quesada, Quebrada El Palo y Las Nubes, Morales 134500 (INB, NY). Villa Quesada San Carlos, Smith 3 (NY); 3.5 km west of Fortuna, 2.5 km northwest of New Volcán Arenal along its sloping base, Taylor and Taylor 11696 (NY). Cartago: roadside near Pavones, Lent 21 (CR, NY). **Guanacaste**: along Guacimal-Monteverde road ca. 16 km from Santa Elena, Penneys and Blanco 1794 (FLAS, NY). **Heredia**: Finca La Selva, the OTS Field Station on the Río Puerto Viejo just east of its junction with the Río Sarapiquí, Sendero El Atajo cerca del Guayabal, Chacón 565 (NY). Finca San Bosco al otro lado del Río Sarapiquí al ES de Puerto Viejo, Jiménez 4149 (MO, NY); Finca La Selva, the OTS Field Station on the Río Puerto Viejo just E of its junction with the Río Sarapiquí, McDowell 131 (F, MO, NY); Sarapiquí. CECAFOR, Santamaria et al. 5650 (INB, NY). **Limón**: slopes above Río Pacuare, between Jicotea and the Río Pacuare along the road from Turrialba to Moravia, Burger and Ramirez 1966 (CR, NY); Shiroles, Talamanca, Tonduz 9345 (NY); above the Río Pacuare about 15 kms west of Moravia, Williams 19497 (NY). **Puntarenas**: Golfito, R.F. Golfo Dulce, Península de Osa, junto al Río Rincón, camino a la estación Los Patos, Acosta et al. 1188 (INB, NY); about 4–5 km N of Miramar on CR 144, Almeda et al. 2866 (CR, NY); Cultivated on the property of Mr. Robert Wilson about 5–6 km south of San Vito, in garden originally from Villa Neilly at 50 m alt, Burger 4596 (NY); Pérez Zeledón, Tinamaste, Finca de los suizos, Estrada 1523 (CR, NY); Cantón de Osa, Camino a Rancho Quemado, del cruce 1 km, Hurtado 115 (CR, INB, MO, NY). **San José**: Camino entre Puriscal y P. N. La Cangreja, Kriebel 5333 (INB, NY); Turrubares, Montelimar, 1 km E de la Escuela, Murillo et al. 85 (INB, NY). Perez Zeledón, Vicinity of El General, Skutch 2503, 5156 (MO, NY); lower slopes of Cordillera de Talamanca along Río Hermoso, Finca El Quizarrá, Williams 28422 (NY).

**PANAMA. Bocas del Toro**: Region of Almirante, Cooper 341 (NY); 10–15 miles inland (S) from mouth of Changuinola River, Lewis et al. 973 (NY); Near Lago de Fortuna, ca. km 80 from Chiriquí on road to Chiriquí Grande, Penneys 1727 (FLAS, NY); Vecinity of Nievecita, Woodson, Allen and Seibert 1874 (MO, NY). **Canal Zone**: Road C-16 northwest of Pedro Miguel, Croat 12254 (MO, NY); **Chiriquí**: Cerro Galera Chorcha, Vcty. Gualaca, Allen 5064 (NY); Río Chiriquí to Remedios, Woodson et al. 1183 (MO, NY). **Coclé**: Vicinity of El Valle, Allen 1152 (NY); Vicinity of San Felix, Allen 3653 (NY); 6.5 km from La Pintada on road to Toabré, Penneys and Blanco 1674 (FLAS, NY); El Valle de Antón and vicinity, Seibert 484 (NY); between Las Margaritas and El Valle, Woodson, Allen and Seibert 1740 (MO, NY). **Darién**: Headwaters of Rio Chico, Allen 4602 (NY); Puerto St. Dorotea, Dwyer 2240 (MO, NY). Veraguas: Hills west of Soná, Allen 1031 (MO, NY).

**COLOMBIA. Antioquia**: en los alrededores de Villa Arteaga, Araque and Barklay 723 (NY); entre los ríos Guapá y León, Ruiz Landa, Rivera and Barklay 379 (NY); Carretera Mutatá-Pavarando, antes del puente sobre el Río Sucio, Fonnegra et al. 1670 (MO, NY); Guapá. 53 km south of Turbo, Haught 4596 (NY); 3 km WSW of Mutatá along road to Pavarandogrande, Zarucchi et al. 5046 (MO, NY).

**ECUADOR. Los Rios**: Hacienda Clementina, Harling 71, 161 (MO, NY).

### Conostegia subpeltata

Taxon classificationPlantaeMyrtalesMelastomataceae

(Kriebel & Almeda) Kriebel comb. nov.

urn:lsid:ipni.org:names:77156273-1

Fig. 196

Conostegia subpeltata (Kriebel & Almeda) Kriebel. Basionym: Clidemia subpeltata Kriebel & Almeda, Brittonia 61(3): 214, f. 2E, F, 6A–I. 2009. Type: Costa Rica. Cartago: Cantón de Paraíso, Parque Nacional Tapantí, al lado de quebrada en la salida del Sendero Arboles Caídos, 948'18"N, 8357'12"W, 1200 m, 8 August 2003, R. Kriebel & D. Solano 3643 (holotype: INB!, isotypes: CAS!, CR!, MO!, NY!, US!).

#### Description.

Shrubs to small trees with adventicious roots 1.5–2 (reportedly to 5) m tall with quadrisulcate young nodes that become terete with age and that are moderately to copiously covered with pinoid hairs intermixed or replaced with stellate and asperous hairs; the nodal line not evident. Leaves of a pair equal to somewhat unequal in length. Petiole 0.7–3.1 cm. Leaf blades 5.75–16.5 × 2.8–8.9 cm, 3–5-plinerved, diverging from the midvein 0.8–1.4 cm above the blade base in opposite to alternate fashion, elliptic to elliptic-ovate, base rounded to subcordate and generally slightly peltate, apex acuminate, margin subentire to denticulate or crenulate, adaxially glabrous, abaxially stellate pubescent on tertiary and higher order veins. Inflorescences laxly branched dichasia in the axils of the upper leaves or lateral, usually arising on branchlets below the leaves and paired mainly at defoliated nodes, 1.5–4 cm long, branching above the base, accessory branches absent, rachis moderately to copiously covered with pinoid hairs intermixed or replaced with stellate and asperous hairs, bracteoles 0.5–1 mm long, subulate. Pedicels 1–2 mm long. Floral buds 4 × mm. Flowers 4 (-5) merous, calyx not calyptrate but fused in bud, shortly apiculate and rupturing into 4 irregular, broadly rounded hyaline lobes 0.25–0.75 mm long and 0.5–0.75 mm wide at the base, the exterior calyx teeth 0.25–0.5 mm long, linear oblong, hypanthium 2–2.75 × 1.25–2 mm, narrowly campanulate to urceolate, sparsely stellate. Petals 2.5–3.5 × 2.5–3 mm, translucent white, ovate to orbicular, glabrous m, reflexed at anthesis, emarginated and strongly asymmetrical. Stamens 8, 3–3.75 mm long, radially arranged around the style, the filament 1.5–2 mm long with a geniculation near the apex, translucent white, anthers 1.25–1.75 × 0.5–0.75 mm, linear-oblong, laterally compressed, yellow, the pore 0.1–0.15 mm, truncate to somewhat ventrally inclined. Ovary 4-locular, inferior, the apex elevated into glabrous collar around the style base. Style 5–5.75 mm long, straight to slightly curving, vertical distance between the anther apex and the stigma 1.9–2.25 mm, horizontal distance absent, stigma truncate to capitellate, ca. 0.3 mm wide. Berry ca. 4–5 × 4–5 mm. Seeds 0.4–0.6 mm, triangular, regulate or muriculate.

**Figure 196.**
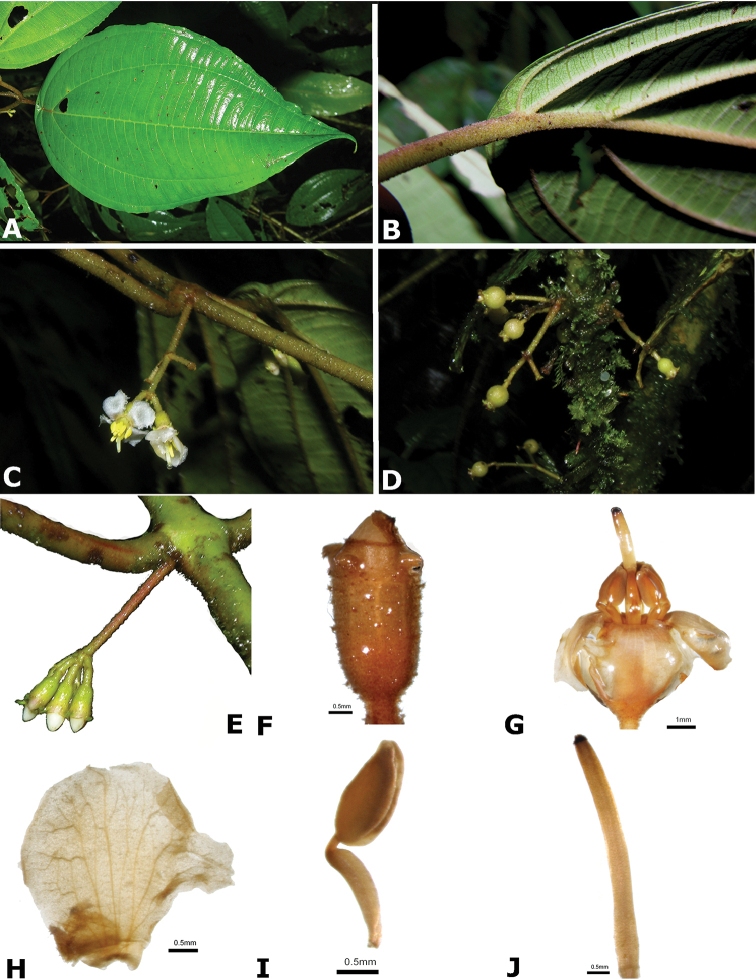
*Conostegia subpeltata*. **A** Leaf adaxial surface **B** Close up of peltate leaf base **C** Inflorescence **F** Infructescence **E** Inflorescence with floral buds **F** Floral bud. Note rupturing calyx **G** Pickled flower **H** Petal **I** Stamen **J** Style. Photos of specimen vouchered *R. Kriebel 5347*.

#### Distribution

**Figure 197.**
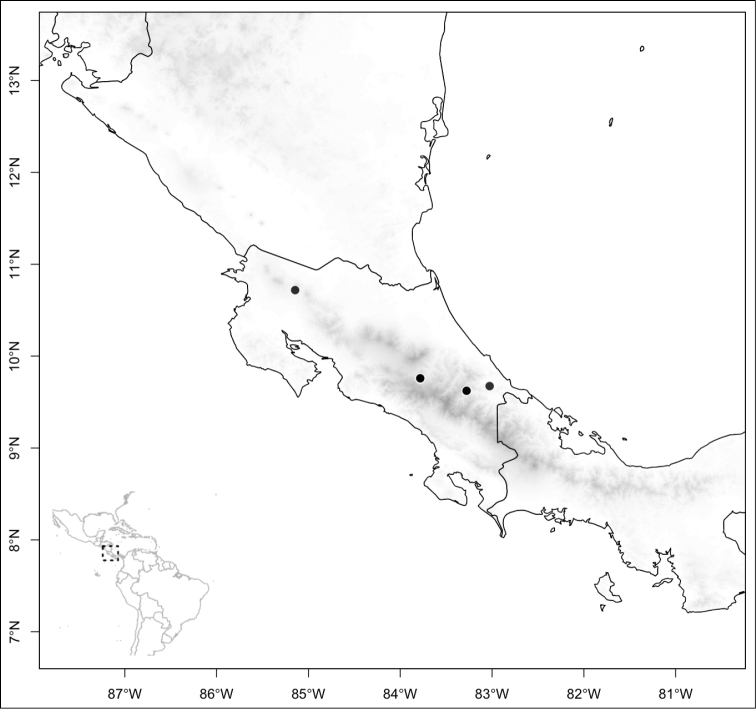
Distribution of *Conostegia subpeltata*.

(Fig. 197). Endemic to Costa Rica where it is known from the Caribbean slope of the Guanacaste and Talamanca mountain ranges at (100-)900–1200 m.

*Conostegia subpeltata* is a very distinctive species because of its slightly peltate leaves, well defined stellate trichomes on the leaves and hypanthia, axillary inflorescences, and 4-merous flowers with a calyx that is fused in bud and ruptures irregularly into unequal lobes. The flowers of this species resemble the common morphology on section *Geniculatae* with bright yellow anthers and exserted styles. This species is known from very few specimens but surprisingly these few specimens come from two mountain ranges, one in Volcan Miravalles in Guanacaste and the other from the Talamanca mountain range (Tapanti National Park and Hitoy Cerere Biological Reserve). *Conostegia subpeltata* forms a well supported species pair with *Conostegia colliculosa* in the molecular phylogeny. This species pair is in turn sister to *Conostegia calocoma*. *Conostegia subpeltata* can be easily distinguished from both of these close relatives by the subpeltate leaves and axillary inflorescences. It shares the 4-merous flowers with *Conostegia calocoma*.

#### Specimens examined.

**COSTA RICA. Cartago**: Cantón de Paraíso, P. N. Tapantí, al lado de quebrada en la salida del Sendero Arboles Caídos, Kriebel et al. 1389 (INB, NY); loc. cit, Kriebel 5347 (INB); near bridge over Rio Aquiares 1/2 km above Santa Cruz, Lent 659 (F). **Guanacaste**: Bagaces, Volcan Miravalles, estación Cabro Muco, Azofeifa 76 (INB, MO). **Limón**: Rainforest slopes of Cerro Skopte west of Río Siori and Río Coén about 7 km beyond Coroma, Almeda and Daniel 7040 (CAS, CR); bosque primario, between Camp 3 and point 11B close to a stream ca. 1km NW of Laguna Dabagri, Bridgewater 4183 (INB, NY); Cuenca del Río Estrella, R. B. Hitoy Cerere, sendero en alrededores de la estación, Rodríguez et al. 4921 (CR, INB, MO).

### Conostegia trichosantha

Taxon classificationPlantaeMyrtalesMelastomataceae

(Almeda) Kriebel comb. nov.

urn:lsid:ipni.org:names:77156274-1

Fig. 198

Conostegia trichosantha (Almeda) Kriebel. Basionym: Clidemia trichosantha Almeda, Proc. Calif. Acad. Sci, Series 4, 43(17): 274, f. 2. 1984. Type: Panama. Coclé: sawmill above El Cope, in forest along stream E of sawmill on the Atlantic drainage, elev. 2300 ft (701 m), 27 July 1978, B. Hammel 4133 (holotype: CAS!, isotype: MO).

#### Description.

Shrub 1–2.5 m tall with terete stems that are moderately to densely covered with smooth e spreading hairs mostly 1–3 mm long; the nodal line inconspicuous and not evident from the indumenta. Leaves of a pair equal or usually unequal in size. Petiole 0.1–2.2 cm. Large leaf blade 5–14.5 × 2.5–6 cm, 5–7 plinerved with the innermost pair of primary veins diverging 0.6–2.2 cm above the blade base and in alternate fashion, elliptic, acute to obtuse, rounded or oblique, acuminate, the margin conspicuously serrate denticulate, adaxially moderately to sparely strigose to hirtellous, abaxially hirsute. Inflorescence a pseudolateral, modified and usually deflexed dichasium with flowers borne in pedunculate terminal glomerules 1.2–3 cm, branched above the base, accessory branches absent, the rachis densely hirsute, lanceolate to naviculiform, 1.5–3 × 0.5–1.5 mm. Flower buds 1.75–2.5 × 1.25–1.75 mm, more or less ovoid, copiously covered with smooth spreading trichomes. Flowers 5 merous, not calyptrate, the hypanthium 1.75–2.2 × 1.5 mm wide, calyx lobes broadly deltoid, 1 × 1.5 mm long, calyx teeth setiform, 1–2 mm long. Petals 4–4.5 × 1.5–2 mm, translucent white, elliptic-lanceolate, spreading to somewhat reflexed, apex acute, glabrous. Stamens 10, radially arranged around the style, the filaments 2–2.5 mm, with a geniculation near the apex, translucent white, anthers 1.25–1.75 × 0.5–0.75 mm, linear-oblong, yellow, somewhat laterally compressed, the pore ca. 0.15 mm wide, truncate. Ovary 5-locular, 2/3 inferior, apically mostly glabrous but with some scattered sessile glands and elevated into a low lobulate collar around the style. Style 5–6.5 mm, gently curving, vertical distance from the anther apex to the stigma 2–2.5 mm, horizontal distance ca. 0.5 mm, stigma punctiform and conspicuously papillose, 0.3–0.4 mm wide. Berry 4–6 × 4–5.5 mm, dark purple; seeds 0.3–0.5 mm, cuneate to triangular, smooth with verruculose angles.

**Figure 198.**
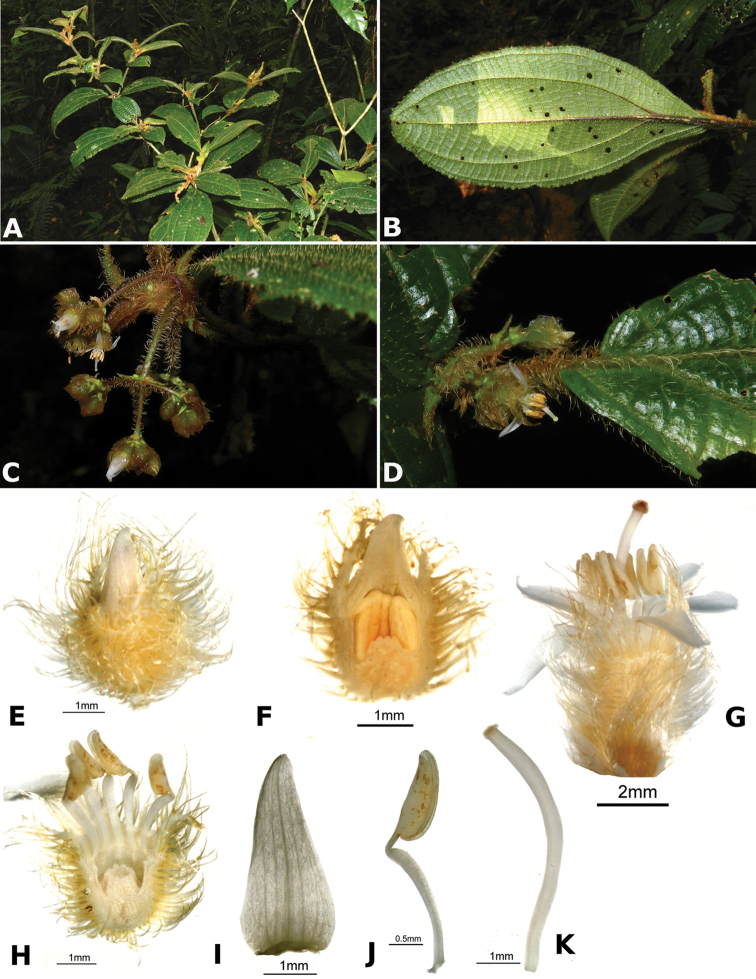
*Conostegia trichosantha*. **A** Habit **B** Leaf abaxial surface **C** Inflorescence **D** Close up the flower **E** Floral bud **F** Longitudinal section of a flower bud with the style removed **G** Pickled flower **H** Longitudinal section of a flower at anthesis with the style removed **I** Petal **J** Stamen **K** Style. Photos of specimen vouchered *R. Kriebel 5693*.

#### Distribution

**Figure 199.**
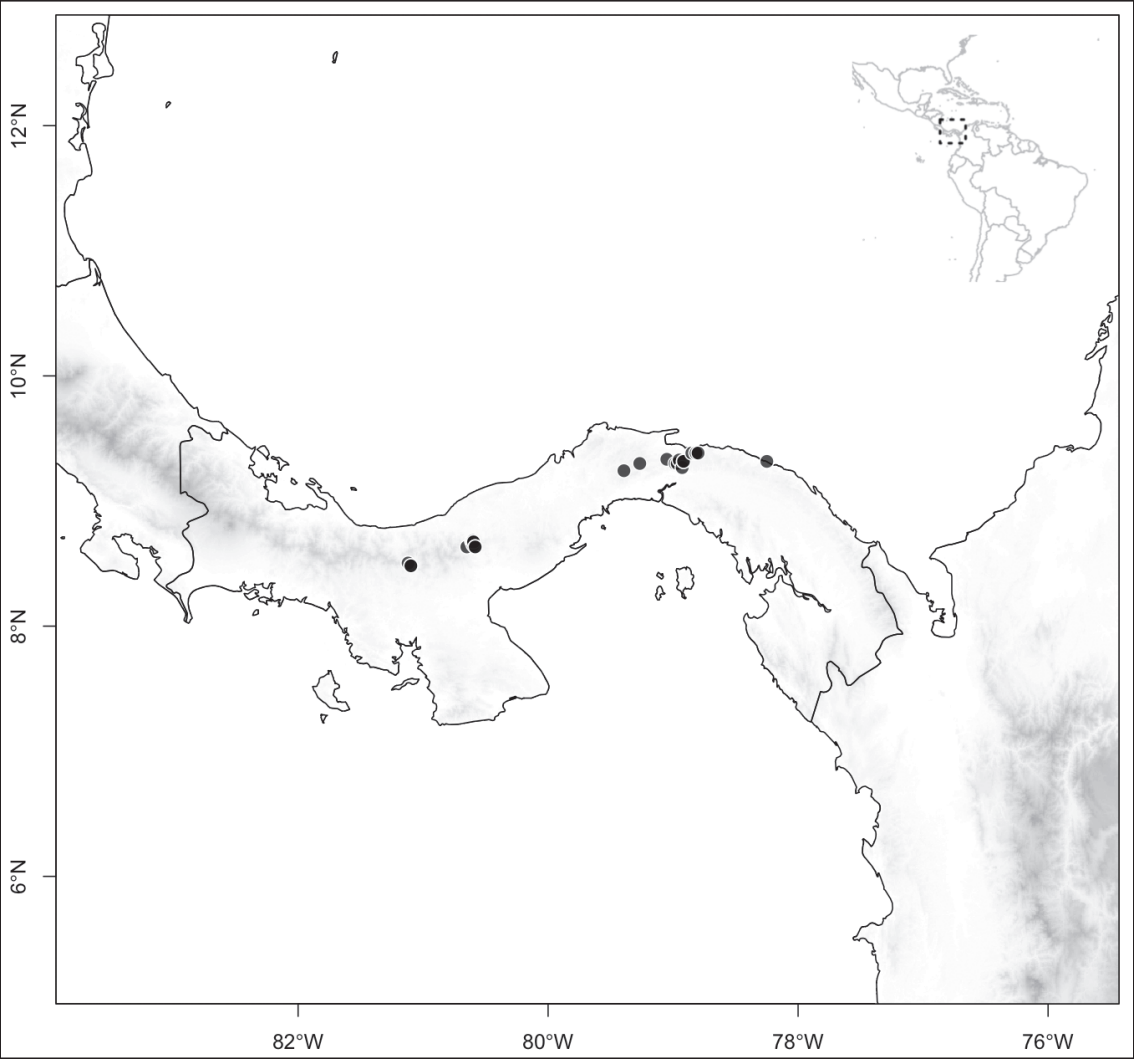
Distribution of *Conostegia trichosantha*.

(Fig. 199). Endemic to Panama where it grows mostly in cloud forests, 250–1250 m in elevation.

*Conostegia trichosantha* is a very distinctive species because of its dense indument of smooth hairs, small leaves with very plinerved and usually asymmetric venation, pseudolateral, short inflorescences, 5-merous flowers with narrow and acute white petals and very exserted styles. It is perhaps not surprising that in the molecular phylogeny, *Conostegia trichosantha* appears as closely related to other Panamanian endemics such as *Conostegia jefensis* and *Conostegia peltata* as well as with the more widespread *Conostegia consimilis*. With these taxa it shares narrow petals and with most of them angulate seeds with verruculose angles. This species accumulates water in the hypanthium indument.

#### Specimens examined.

**PANAMA. Coclé**: about 7–10.5 km beyond El Cope in Omar Torrijos National Park, along end of the rocky trail to Río Blanco and Limon beyond Alto Calvario, Almeda et al. 7653 (CAS); 7 kms north of El Copé, area around Rivera Sawmill, called Alto Calvario, New Works, Folsom et al. 7093 (CAS). **Panamá**: forested slopes along El Llano Cartí road 12 km from Panamerican Highway, D’Arcy 10617 (CAS); loc. cit., Sytsma and Anderson 4493 (CAS); Comarca de San Blas, Nusagandi, along Continental Divide on the El Llano Carti Road, de Nevers and Pérez 3577 (CAS); Nacional Chagres, Cerro Jefe, Road leading NE from TV towers, Penneys and Blanco 1697 (FLAS, NY). **Veraguas**: along trail to summit of Cerro Tute above the Escuela Agrícola Alto de Piedra near Santa Fé, Almeda et al. 6491 (CAS); P. N. Santa Fé, sendero a la cima del Cerro Mariposa, Kriebel and Burke 5693 (PMA, NY).

### Conostegia xalapensis

Taxon classificationPlantaeMyrtalesMelastomataceae

(Bonpl.) DC

Figs 200 , 201

Conostegia xalapensis (Bonpl.) DC, Prodr. 3: 175. 1828. Melastoma xalapense Bonpl., Melast. 126, t. 54. 1806–1816. Type: Mexico. Jalisco: Rio Papagayo, no date, A. Bonpland s.n. (holotype: P!).
Miconia xalapensis (Bonpl.) M. Gómez, Anal. Hist. Nat. Madrid 23: 69. 1894.
Miconia rostrata (Bertol.) Triana, Trans. Linn. Soc. London 28: 131. 1872. Basionym: Melastoma rostratum Bertol., Novi Comment. Acad. Sci. Inst. Bononiensis 4: 417. 1840. Type: Guatemala. Antigua: J. Velsquez s.n. (holotype: BOLO).
Miconia umbilicata (Bertol.) Triana, Trans. Linn. Soc. London 28: 131. 1872. Basionym: Melastoma umbilicatum Bertol., Novi Comment. Acad. Sci. Inst. Bononiensis 4: 416. 1840. Type: Guatemala. Escuintla: J. Velsquez s.n. (holotype: BOLO).
Conostegia acutidentata Rich, Ess. Flo Cuba 558. 1845. Type: Cuba. Vuelta de Abajo, Cuba, J. Valenzuela. I have not seen this specimen. Schnell (1996) states that the description of the leaves, “leaves little room for doubt” it is this species.
Conostegia lanceolata Cogn, DC. Monog. Phan. 7: 708. 1891. Type: Costa Rica. San Jose: Salitral de Desamparados, May 1889, H. Pittier 1144 (lectotype BR!, designated here; isolectotypes LE, M). The specimen at M has the name H. Pittier scratched off leaving Tonduz as the colector. Other syntypes: Costa Rica: San Jose, Wendland 639 (BR, GOET) and 1092 (GOET); Aguacate, Oersted 2836 (holotype: C).
Conostegia lanceolata var. subtrinervia Cogn., Bull. Soc. Roy. Bot. Belgique 30: 253. 1892. Type: Costa Rica. San José: H. Pittier 1144 (holotype: BR!).
Conostegia minutiflora Rose, Contr. U.S. Natl. Herb. 8(4): 327, t. 71. 1905. Type: Mexico. Oaxaca: Plunia, 17 March 1895, E. W. Nelson 2493 (holotype: US!; isotype: GH!).
Conostegia viridis Cogn. ex Donn. Sm, Bot. Gaz. 20: 286. 1895. Type: Guatemala. Retalhuleu: San Felipe, April 1892, J. Smith 2650 (holotype: BR!; isotypes: K!, M!, US!).
Conostegia minutiflora Rose, Contr. U.S. Nat. Herb. 8: 327. 1905. Type: Mexico. Oaxaca: Plunia, 17 March 1895, E. Nelson 2493a (holotype: US!, isotype: GH!).

#### Description.

Shrubs or trees 0.5–12 m tall with apically flattened stems which become terete with age and which are covered by a dense tomentum of sessile stellate trichomes; the nodal line present but sometimes invisible from the indument. Leaves of a pair equal to slightly unequal in length. Petioles 0.5–5.1 cm long. Leaf blade 3–18.6 × 0.9–8.8 cm, 3–5 plinerved, with the innermost pair of primary veins diverging from the midvein 0.3–1.5 cm above the base in opposite to alternate fashion, ovate-oblong, or narrowly to broadly ovate, the base acute to subcordate, the apex acute to acuminate, the margin dentate or denticulate, the adaxial surface glabrous, the abaxial surface white, grey, brown or reddish from the dense tomentum of sessile stellate trichomes. Inflorescence a terminal panicle 2.3–20 cm long, branched at or above the base, accessory branches absent or present, the rachis covered by sessile stellate trichomes, the bracteoles 0.5–3.25 mm, linear, deciduous or persistent. Pedicel absent or to 1.25 mm. the hypanthium 2–4 × 2–3.5 mm, covered by stellate hairs. Flowers (4-)5(-7) merous, pyriform, calyptrate, floral buds 3–8.75 × 1.75–5 mm, the base rounded, the apex acute to apiculate and sometimes with inconspicuous calyx teeth looking appendages at the top of the calyptra, scarcely constricted below the middle. Petals 3–8 × 1.5–3.25 mm, white or pink, obovate, spreading at anthesis, glabrous, rounded to emarginated apically. Stamens (9-)10(-15), 4–7 mm, androecium slightly zygomorphic, the filaments 2.2–4.25 mm, white, the filament geniculation just below the thecae, anthers 1.8–3.25 × 0.5–1.25 mm, elliptic to oblong, yellow, laterally compressed, the pore 0.1–0.2 mm, slightly ventrally inclined. Ovary (4-)5(-6) locular, inferior, apically glabrous and forming a small collar around the style base. Style 3–6.25 mm, bent away from anthers just below the stigma, vertical distance from the anther to the stigma ca. 0 mm, horizontal distance absent, the stigma punctiform to slightly expanded, 0.5–1 mm wide. Berry 4–7 × 4–7 mm, dark purple to black. Seeds 1–1.4 mm long, broadly pyramidal or rounded, the testa smooth.

**Figure 200.**
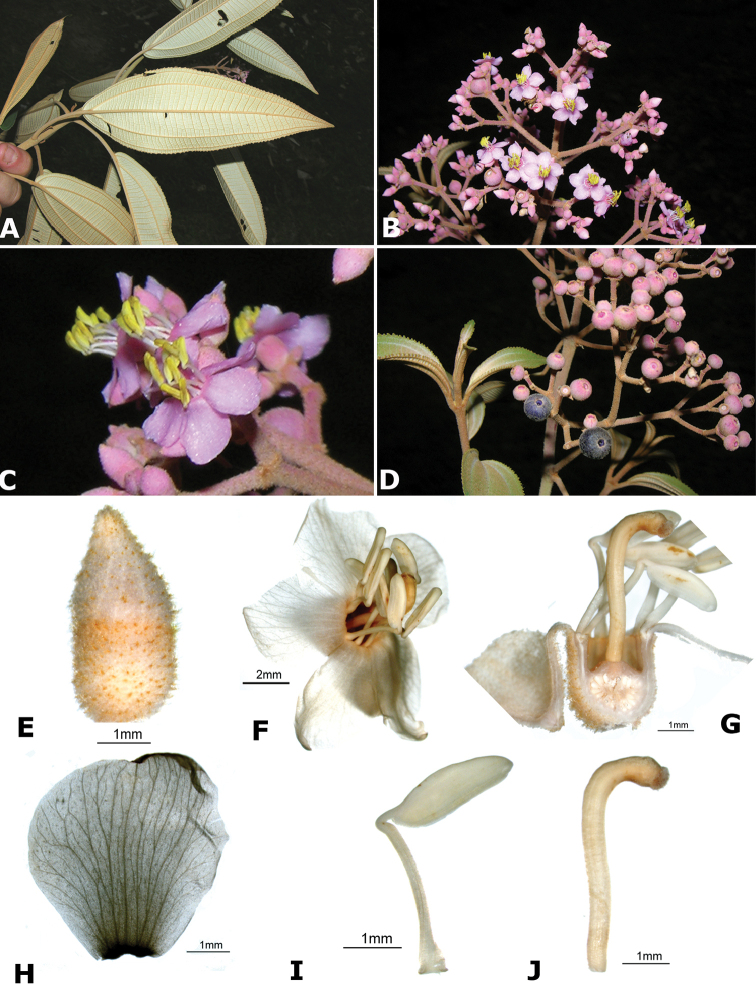
*Conostegia xalapensis*. **A** Leaf abaxial surface **B** Inflorescence **C** Close up of flowers **D** Infructescence **E** Flower bud. Pickled flower **G** Longitudinal section of a flower **H** Petal I Stamen **J** Style. Photos of specimen vouchered *R. Kriebel 5619*.

**Figure 201.**
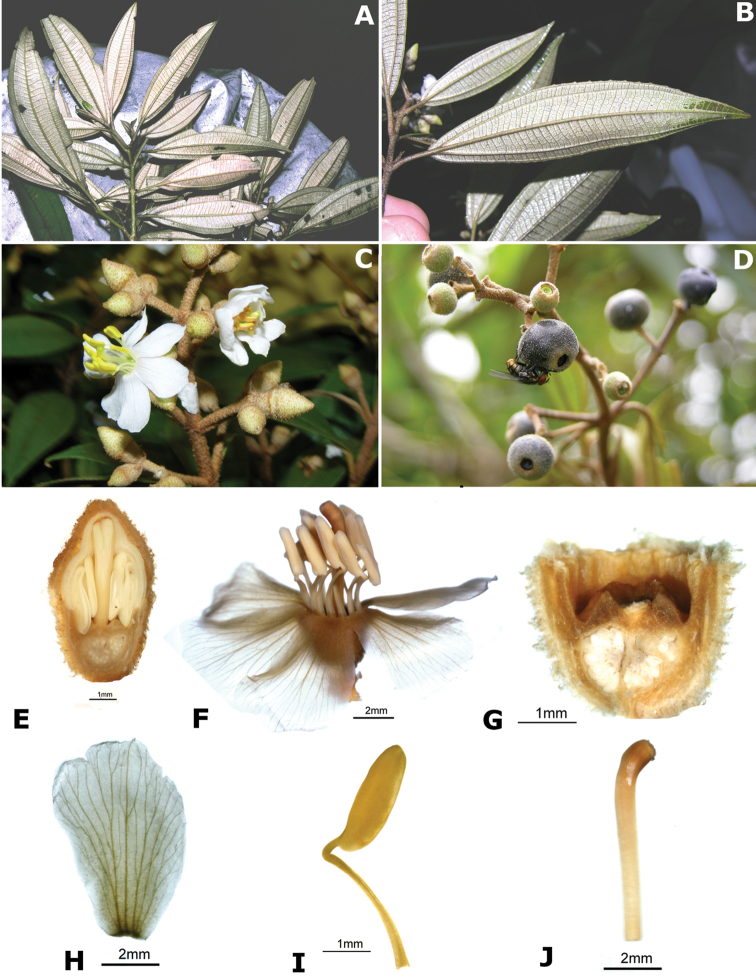
*Conostegia xalapensis*. **A** Habit. Leaf abaxial surface. Inflorescence. Infructescence **E** Longitudinal section of a flower bud **F** Pickled flower **G** Longitudinal section of a flower at anthesis with stamens, style and petals removed **H** Petal **I** Stamen **J** Style. Photos of specimen vouchered *R. Kriebel 5629*.

#### Distribution

**Figure 202.**
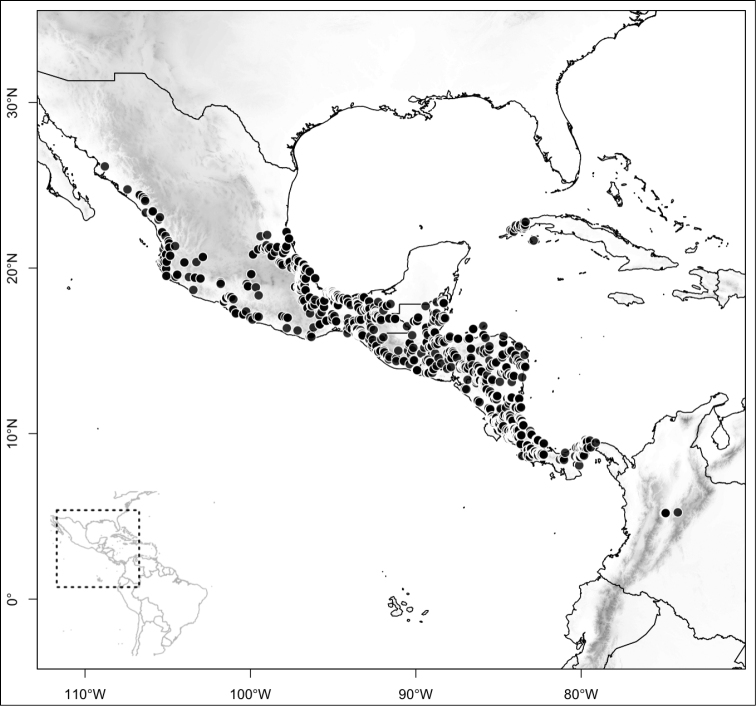
Distribution of *Conostegia xalapensis*.

(Fig. 202). From Mexico south to Panama and reaching central and northern Colombia, also in Cuba, from sea level to 2880 m in elevation.

*Conostegia xalapensis* has one of the widest distribution of any species in the genus and is by far the most commonly collected species. It is a weedy species and is probably expanding its range in the present. With this wide distributional range also comes morphological variation. Schnell (1996) characterized several morphotypes within *Conostegia xalapensis*. Of these, I have observed two of them which are here illustrated (Figs 201, 202). The specimen *Kriebel 5619* (NY) from Sierra Caral, Izabal, Guatemala which is a low elevation rainforest locality, has diplostemonous flowers with small pink petals. This morphotype matches *Conostegia viridis*, a species Cogniaux recognized. The specimen *Kriebel 5629* (INB) from higher elevations in the Cerros de Escazú, San José, Costa Rica, has pleiostemonous flowers with larger white petals and matches another species recognized by Cogniaux, *Conostegia lanceolata*. These extremes look different but there are many populations that seem to bridge their extreme morphologies. Further genetic work is needed to test if some of these populations, which have been given other names, in fact, correspond to distinct species. The accumulation of aluminum by this species has been demonstrated experimentally (González-Santana et al. 2012).

#### Specimens examined.

**CUBA. Oriente**: ladera sur de Cajálbana, 15 kms de La Palma hacia Mil Cumbres, Berazaín, Baker and Reeves 71858 (NY); Vicinity of Herradura, Britton et al. 6362 (NY); on Guane road, Britton, Britton and Gager 7207 (NY); San Isidro, Britton, Wilson and Leon 13948 (NY); El Pinar (pine wood) not far from San Diego de los Barrios, León 4257 (NY); Pan de Guajaibón rail from village of San Juan de Sagua and base of Pan de Guajaibón, Clark et al. 10592 (NY); Highway Luis Lazo towards Cabeza, between Valle Isabel Maria and Mestanza (ca. 9 air km northwest of Pinar del Rio), Clark 11983 (NY); Lomas de Candelaria, Earle 1618 (NY); Sierra de los Organos, San Claudio, Pinar de Lexhuza, Ekman 10545 (NY); junto a Soroa 4-5 km al norte sobre serpentinas, Fernández and Morales 10752 (MO, NY); Municipio San Cristobal cerca de Cinco Pesos, Greuter, Rankin and Silva 25800 (NY); Mantua to Arroyos, Shafer 11240 (MO, NY); Los Palacios to Herradura, Shafer 11722 (MO, NY); Los Palacios to San Pablo de Las Yeguas, Shafer 11930 (MO, NY).

**MEXICO. Chiapas**: 13 km north of Berriozábal near Pozo Turipache and Finca El Suspiro, Municipio de Berriozábal, Breedlove 20225 (MO, NY); Tabil Nuk’um 2 km al norte de tierra templada monte bajo la cabecera, Gonzalez 638 (NY); along the road from El Boque to Simojovel, Municipio de Simojovel de Allende, Ton 3071 (NY). **Guerrero**: Manchón, Mina, Hinton 9256 (NY); Mpio. Mochitlán, camino a la torre de microondas “El Fresno”, 2.5–3.5 km al E de Cajeles, Koch, Fryxell and Altman 87524 (NY). **Michoacán**: municipio de La Huacana MEX 120, 19 km north of La Huacana and 2.5 km southwest of Los Sabinos, Steinmann and Porter 5361 (NY). **Nayarit**: Roadside along highway between Tepic and Santa Cruz 2.4 mi. NE jct. Hwy. to San Blas and 8.9 mi. SW of Jalcocotan, Daniel 2037 (NY); ca. 12 miles west of Tepic 6.5 miles east of Jalcocotán, McVaugh 18845 (NY); Ruiz, Mexia 995 (MO, NY). **Oaxaca**: Dto. Yautepec, Mpio. San Carlos Yautepec, a 7 km al S de San Miguel Chongo, Martínez, Elorsa and Perret 32118 (NY); Distrito de Juchitán, Mpio. de San Juan Guichicovi, 2 km. al sur de Piedra Blanca (13 km. al Norte de Matías Romero), Perina 3236 (NY). Collected along Hwy 185, 8.3 mi. north of Matias Romero, Trott et al. 260 (MO, NY). **Puebla**: Carretera Guayabal, San Jose Acateno, Zola 110 (NY). **San Luis Potosí**: Mountains along the gravel road to Jalpan 2 miles generally west of Xilitla, King 4267 (NY); Mountain canyons, Hacienda de Tamasopo, Pringle 3976 (MO, NY). **Sinaloa**: Mesa Malqueson, Cerro Colorado, Gentry 5166 (MO, NY); San Agustín, Ortega 4002 (NY); along the dirt road from Rosario to Plomosas about 3.5 miles east of La Rastra and 0.7 mile up the grade from the river crossing, about 33 miles east of Mexico Highway 15 at Chilillos this about 1 mile south of Rosario, Reveal and Harley 4047 (MO, NY). **Tabasco**: Acahual del modulo, Ejido Lázaro Cárdenas, Cowan 2035 (MO, NY). Ejido Francisco Villa 8 km al S de Francisco Rueda, municipio Humanguillo, Fernández 1424 (NY); Municipio Teapa along bank of Río Azufre on trail between Campamento San Joaquín, Chiapas and Teapa, Gilly and Hernández 215 (NY). **Tamaulipas**: Vicinity of Gómez Fárias, Paler 278 (MO, NY). Veracruz: 1.5 km despues de la estación de Chavarrillo rumbo a Palmar, Municipio Emiliano Zapata, Castillo and Tapia 753 (MO, NY); 1 km al S de Palmillas Municipio, Puente Nacional, Castillo and Medina 4251 (NY); 3 km SW of Campamento La Laguna, Mun. Hidalgotitlan, Nee 30021 (NY); Rancho de la secretaria de salubridad y asistencia (antes rancho 3 pasos), Municipio E. Zapata, Ortega 672 (NY); above saddle on s.e. ridge of San Martin Tuxtla s.e. of road between Catemaco and Sontecomapan, Ward and McVaugh 7917 (NY).

**BELIZE. Belize**: Butcher Burns Road off Western Highway about Mile 21, Dwyer 12447 (MO, NY). **Cayo**: at Millonario on road past Augustin on way to Cuevas, Croat 23510 (MO, NY); Mt. Pine Ridge, Vicinity of Blancaneaux Lodge, Savanna. 17 mi. S of Georgeville, Liesner and Dwyer 1614 (MO, NY). **Stann Creek**: Cockscomb Basin, O? Southern Highway, Road between Jaguar Preserve and Maya Ctr, Balick et al. 2699 (MO, NY); Dangriga, near town along Melinda Road, Balick 3020 (NY); Silkgrass creek, Gentle 8175 (NY). **Toledo**: of highway from Dangriga to Punta Gorda, Road to Laguna Village, Balick et al. 3582 (MO, NY).

**EL SALVADOR. San Salvador**: Calderón 106 (NY). **Sonsonate**: Hacienda Las Tablas in the Balsam range, Tucker 1332 (NY).

**GUATEMALA. Alta Verapaz**: Low hills along National route 7 W about 3 miles west of San Cristóbal Verapaz, King 3339 (NY); near Cobán, Standley 69318 (NY). Escuintla: Escuintla, Smith 2215 (NY). **Izabal**: Sierra Caral, quebrada atravesada por el sendero al noreste de la casa de investigadores, hacia la Finca Bonanza, Kriebel et al. 5619 (NY). **Quetzaltenango**: Colomba, Skutch 1281 (NY). **Petén**: La Libertad, Lundell 2186, 3208 (NY); San Francisco 15 km SSW Flores, Savannen entlang der Strasse in Richtung San Vincente das ist ca. 3 km nach der Strassen-Abzweigung in San Francisco, Wallnofer and Frisch 5800 (NY). Retalhuleu: San Felipe, Smith 2649 (MO, NY). **Santa Rosa**: Santa Rosa, Heyde 3330 (NY). **Sololá**: pine woods bordering Río Bravo in vicinity of Finca Mocá south facing slopes of Volcán Atitlán, Steyermark 47962 (NY).

**HONDURAS. Atlántida**: vicinity of La Ceiba, Yuncker 8026 (MO, NY). **Colón**: Road to Castilla 0.2 miles east Trujillo, Saunders 168 (NY); 1.8 mi strip on the north bank of río Guaimoreto between old bridge and opening of Laguna Guaimoreto 4.5 mi NE of Trujillo on oald road to Castilla, Saunders 766 (NY). **Comayagua**: Pinewoods around Escuela Nacional de Ciencias Forestales, Siguatepeque, Burch 6081 (MO, NY); Montaña La Choca, Cordillera Comayagua, cerca de Coyocutena, Molina 7115 (NY). **Cortés**: Tulian 5 km O de Pto. Cortes, Ruiz 163 (NY). **Copán**: cerca de Dulce Nombre, Molina 11774 (NY). **Cortés**: Montana San Idalfonso entre Banaderos y Cusuco, Molina 11438 (MO, NY). **Distrito Central**: Escuela Panamericana de Agricultura Forest Reserve, 5 km east of Zamorano on road to Tegucigalpa, Balick et al. 1745 (NY). **El Paraíso**: 17 km E of El Zamorano, Davidse and Pohl 2138 (NY) ; Yuscarán, Ochoa 40 (NY). Francisco Morazán: Ojojona 24 km SO de Tegucigalpa, Avila 156 (NY); Linderos km 92 carretera a Olancho, Chévez 186 (NY); Valle de Angeles, quebrada Sabana Redonda 2 km del empalme, Cristoff 130 (NY). El Hatillo 10 kms NE de Tegucigalpa, Figueroa 136 (NY). **Gracias a Dios**: La Mosquitia, alrededores de Mocoron 60 km al SO de Puerto Lempira, Torres 137 (NY). **Guaimaca**: Trail from Finca Sansón to Cerro Sansón, Carlson 3201 (NY). Lempira: 2 kms. from Erandique, Molina 24046 (NY). Olancho: Alrededores de Campamento, Gomez 17 (NY); bosque de Montaña La Bellota en Cordillera Almendares 8 kms de Campamento, Molina 8451 (NY); Arroyadas del río Wampú, Dulce nombre de Culmí, río Wampusito, Nelson and Clewell 387 (NY). **Santa Bárbara**: Near El Mochito, Davidse and Pohl 2211 (NY). **Tela**: Lancetilla Exp. Station, Mallery 5016 (NY). Near town of Roatan on the Island of Roatan, Harmon and Dwyer 3921 (NY). **Yoró**: Subirana Valley, von Hagen and von Hagen 1050 (NY).

**NICARAGUA. Chontales**: 4 km N of Cuapa, Nee 28306 (MO, NY); Vicinity of Finca San Pedro de Oluma on NE flanks of Cerro Oluma 4 km N of Cuapa, Nee 28335 (NY); Sierra Amerrisque, Cerro Los Andes 10 km E of Juigalpa, Neill 7295 (NY). Jinotega: San Rafael Norte, Neill 201 (NY). **Managua**: 27 km S of Managua along Highway 8 (road to Masachapa), Davidse and Pohl 2380 (MO, NY); Casa Colorada and vicinity south of Managua, Maxon, Harvey and Valentine 7433 (NY). **Matagalpa**: Cordillera Dariense, La Fundadora, Hall and Bockus 7941 (NY); area between Disparate de Potter and Aranjuez Cordillera Central de Nicaragua, Williams et al. 23672 (MO, NY); Tuma Grande about 8 km east of Matagalpa, Williams 27563 (MO, NY); Departamento de Bluefields, Finca Santa Rosa, ca. 2.5 km ENE of Rama and vicinity of Rio Escondido, Proctor et al. 27331 (NY); Comarca del Cabo, Waspan, Davidse and Pohl 2325 (MO, NY); Vicinity of Waspam, Cabo Gracias a Dios, Bunting and Licht 497 (NY). **San Juan del Norte**: (Greytown), Smith 93 (MO, NY). **Zelaya**: estimated 10 km north of Bluefields, Harmon 5090 (NY); Río Chiquito a 5-7 km al N de Atlanta en Caño dos Oros, Río Punta Gorda Costa del Atlántico, Tellez et al. 4931 (NY).

**COSTA RICA. Alajuela**: Colinas de San Pedro de San Ramón, Brenes 5275, 6452 (NY); Vicinity of Los Chiles, Río Frío, Holm and Iltis 702 (NY); San Luis de Zarcero cantón de Alfaro Ruiz, Smith 1526 (NY). **Cartago**: 5 km above Tobosí, along road to Frailes, Lent 1153 (NY); Between Puente Negro (over Río Agua Caliente) and Río Sombrero at Muñeco, Utley and Utley 2990 (NY); Turrialba, Tonduz 8350 (NY); about 10 km SW of Navarro on a winding trail or about 10 km. S of Cartago in a straight line, Wilbur and Stone 10560 (MO, NY); La Carpintera above Tres Ríos, Williams 19695 (NY); Forest area near Pavones, about 15 kms east of Turrialba, Williams 19700 (NY). Guanacaste: Cacao camino a Cerro Pedregal y su cumbre, Soto 1772 (INB, NY). **Heredia**: south slope of Volcán Barba above San José de la Montaña, Hatheway 1493 (NY); Cantón de San Isidro, Junto a la carretera Braulio Carrillo, 2 km antes del peaje, Jiménez 1116 (CR, NY); Lagunas aledañas al Río San Juan, Solano and Hernández 1472 (INB, NY). **Limón**: aprox. 200 yards inland from the coastline ca. 8 km S of Limon on the road to Cahuita, Almeda et al. 3232 (CR, MO, NY); Vicinity of U.S. Department of Agriculture Rubber Experiment Station, Los Diamantes on Río Sta. Clara (1.6 km E of Guapiles), Holm and Iltis 412 (MO, NY); Puerto Viejo, R.V.S. Gandoca-Manzanillo, Punta Mona, Kriebel 426 (INB); P. N. Tortuguero, Lumer 1355 (NY); densely shrubby banks on the Moin River ca 0.25 mi from its mouth, 5 mi N of Puerto Limón, Morley 784 (NY); Puerto Viejo, Manzanillo, hacia la punta, Vargas 2471 (INB, NY). **Puntarenas**: Monteverde, on property of A. Hoage at edge of woods, Lumer 1046 (NY); Road between Santa Elena and Monteverde, Lumer 1311 (CR, NY); Biolley, Sabanas Esperanza, sabana inferior, Rodríguez and Santamaría 9939 (INB, NY). **San José**: Vicinity of Altos Tablazo about 7 km west of Tablón and SE of Higuito, Almeda et al. 2830 (NY); Tablazo above San Lorenzo de Tres Ríos, Barringer and Christenson 3285 (CR, MO, NY); Lado de Vargas, Tabarcia, Mora, Jiménez 659 (CR, NY); Cerros de Escazú, Kriebel 5629 (INB); Z.P. Cerros de Escazú, Suarez, Cuenca del Río Suarez, carretera al Cedral por cuesta de Piedra, Morales, Lépiz and Ramírez 1751 (INB, NY); Acosta, Bajo Pérez, 4 km de Teruel, Morales 10539 (INB, NY); Perez Zeledón, Vicinity of El General, Skutch 4175, 2582 (MO, NY).

**EL SALVADOR. Ahuachapán**: Vicinity of Ahuachapán, Standley 20302 (NY). **Chalatenango**: beside trail from San Ignacio to Las Pailas, west side of Los Esemiles, Tucker 1207 (NY).

**PANAMA. Canal Zone**: Barro Colorado Island, West side of Orchid Island, Croat 12281 (MO, NY); Militar Reserva Fuerte Sherman from Pina to 3 mi northeast of Pina, Liesner 1371 (MO, NY); Barro Colorado Island, Woodworth and Vestal 678 (NY). **Chiriquí**: along road between Boquete and Cerro Horqueta, Dwyer 13729 (MO, NY); Río Chiriquí Viejo Valley, near El Volcan, White 210 (MO, NY). Coclé: El Valle de Antón, North Hills, Allen 3546 (NY); roadside between Barrigon and entrance to Parque Nacional Omar Torrijos, Penneys and Blanco 1758 (NY). **Panamá**: Hills above Campana, Allen 1693 (NY); Upper slopes of Cerro Campana within the boundary of the national park administered by RENARE, LeDoux 2574 (MO, NY); SE slopes of Cerro Trinidad, Kirkbride and Duke 1669 (MO, NY). Colón: Road to Santa Rita, Busey and Mahler 333 (NY); 2 miles south of Portobello along river, Croat 11414 (NY).

**COLOMBIA. Caldas**: cerca a Victoria (región próxima a Mariquita), Uribe and Pérez 2714 (NY). **Cundinamarca**: Pacho-Paime Highway, Haught 6055 (NY). **Tolima**: cerca a “La Parroquia”, Uribe 2764 (NY). **Santa Marta**: Cacagualito, Smith 6 (NY).

### Excluded taxa or uncertain names

*Conostegia acuminata* Steud., Flora 27(2): 722. 1844. This is the basionym for *Miconia acuminata* (Steud.) Naudin, Ann. Sci. Nat., Bot., Ser. 3 16: 244 (1850). The latter is the currently accepted name (Goldenberg et al. 2013).

*Conostegia cornifolia* (Desr.) Ser. ex DC., Prodr. 3: 175. 1828. Based on *Melastoma cornifolium* Desr., Encycl. Méth. Bot. 4: 51 (1797). This name was treated in Cogniaux’s (1891) monograph as a synonym of *Miconia cinnamomifolia* (Jacq.) Triana, an illegitimate name i.e. a later homonym of *Miconia cinnamomifolia* (DC.) Naudin. The currently accepted name is *Miconia cornifolia* (Desr.) Naudin (Goldenberg et al. 2013).

*Conostegia cucullata* D. Don ex DC., Prodr. 3: 176. 1828. Based on *Melastoma cucullata* Pavon Msc. ex D. Don. This name appears in Cogniaux’s (1891) monograph as a synonym of *Calyptrella cucullata* Triana, but in Triana’s (1872) monograph there is no description. The first valid publication of the name *Calyptrella cucullata* would seem to be in Cogn., Mart. Fl. Bras. 14(4): 44. 1886. This name is currently treated as a synonym of *Graffenrieda cucullata* (Triana ex Cogn.) L.O.Williams, Fieldiana, Bot. 29: 563 (1963).

*Conostegia discolor* DC., Prodr. 3: 174. 1828. This name was treated in Cogniaux’s (1891) monograph as a synonym of *Charianthus coccineus* (Rich.) D. Don, and the latter is currently considered as a synonym of *Charianthus alpinus* (Sw.) R.A. Howard (Penneys and Judd 2005).

*Conostegia excelsa* Pittier, J. Wash. Acad. Sci. 14: 450. 1924. This name is a synonym of *Meriania macrophylla* (Benth.) Triana (Almeda 1993).

*Conostegia glabra* (G. Forst.) D. Don ex DC. Prodr. 3: 176. 1828. Based on *Melastoma glabrum* G. Forst., Prodr. : 34 (1786). The currently accepted name for this species is *Astronidium glabrum* (G.Forst.) Markgr., Notizbl. Bot. Gart. Berlin-Dahlem 12: 50 (1934).

*Conostegia gloriosa* Macfad., Fl. Jamaica 2: 68. 1850. Invalid name and possibly a taxonomic synonym of *Conostegia procera* (Sw.) D. Don ex DC.

*Conostegia holosericea* D. Don ex DC., Prodr. 3: 176. 1828. Nomen nudum.

*Conostegia inusitata* Wurdack, Phytologia 16: 170. 1968. Based on preliminary nrETS a relative of this species, the undescribed *Florbella wurdackii*, is more closely related to a clade of species of *Miconia* from Peru.

Conostegia lanceolata f. grandifolia Cogn, Prim. Fl. C.R. 1: 156. 1892 (fide Stafleu and Cowan 1976, p. 709). Nomen nudum.

*Conostegia lutescens* (Vahl) Ser. ex DC., Prodr. 3: 175. 1828. Based on *Melastoma lutescens* Vahl, Eclog. Amer. 3: 17 (1807). This name is a synonym of *Miconia cornifolia* (Desr.) Naudin (Goldenberg et al. 2013).

*Conostegia mexicana* (Bonpl.) Ser. ex DC is a synonym of *Miconia mexicana* (Bonpl.) Naudin (Goldenberg et al. 2013) as verified by the description and plate provided by Bonpland in his description of *Melastoma mexicanum* Bonpl. The description of *Conostegia mexicana* Cogn. matches that of *Conostegia icosandra*, as does the lectotype Galeotti 2963 (BR) where it was placed in synonymy in this work. The specimen Galeotti 2963 was cited by Cogniaux (1891) under *Miconia mexicana* (Bonpl.) Naudin possibly by mistake.

*Conostegia mutisii* (Bonpl.) Ser. ex DC., Prodr. 3: 174. 1828. Based on *Melastoma mutisii* Bonpl., Monogr. Melast., 136. 1816. This currently recognized name for this species is *Meriania mutisii* (Bonpl.) Humberto Mend. & Fern-Alonso.

*Conostegia myriasporoides* Triana. Trans. Linn. Soc. London 28(1): 99. 1872. Schnell (1996) suggested to move this species to *Cyphostyla* Gleason, but this genus is now a synonym of *Allomaieta*. Both of these genera belong to a different tribe than *Conostegia*, the capsular fruited Cyphostyleae.

*Conostegia orbeliana* Almeda, Proc. Cal. Acad. Sci. 46: 333. 1990. The identity of this species is hard to confirm. It was put in the synonymy of *Conostegia volcanalis* by Schnell, a species otherwise restricted to mostly north of Nicaragua. As recognized by Schnell (1996), the Panamanian specimens described as *Conostegia orbeliana* differ in their smaller leaves and petioles, larger flowers and different flowering time, details of the pubescence, and anther morphology. It seems that enough differences are found between these populations to cast doubt as if they represent the same species. The problem arises in that in the southern Central American mountains other similar species to these also grow. For example, *Conostegia oerstediana* and *Conostegia macrantha*. Thus it seems reasonable to wait for more material of what has been described as *Conostegia orbeliana* to confirm their differences.

*Conostegia parviflora* (Aubl.) DC., Prodr. 3: 175. 1828. Based on *Melastoma parviflorum* Aubl., Hist. Pl. Guiane 1: 433; t. 171 (1775). This name is currently treated as a taxonomic synonym of *Miconia prasina* (Sw.) DC. (Goldenberg et al. 2013).

*Conostegia quadrangularis* Steudel, Nomencl. ed. II 1: 405. 1841. Nomen nudum.

*Conostegia semicrenata* (Richard in Bonpl.) Ser. ex DC., Prodr. 3: 175. 1828. Based on *Melastoma semicrenatum* Richard in Bonpl., Monogr. Melast. : 69; t. 31. (1809). This name is a synonym of *Miconia cornifolia* (Desr.) Naudin (Goldenberg et al. 2013).

*Conostegia sub-hirsuta* Gomez, Anal. Hist. Nat. Madrid 23: 69. 1894. No specimen was cited in the publication.

*Conostegiatunicata (Bonpl.) Ser. DC., Prodr. 3: 175. 1828.* Based on *Melastoma semicrenatum* Richard in Bonpl., Monogr. Melast. : 69; t. 31. (1809). This name is a synonym of *Miconia tunicata* (Bonpl.) Naudin (Goldenberg et al. 2013).

Conostegia xalapensis f. canescens Cogn. ex Donn. Sm, Enum. Pl. Guat. 2: 21. 1891. Nomen nudum.

Conostegia xalapensis f. parvifolia Cogn. ex Donn. Sm, Enum. Pl. Guat. 3: 28. 1893. Nomen nudum.

Conostegia viridis var. angustifolia Cogn. ex Donn. Sm, Bot. Gaz. 20: 286. 1895. Nomen nudum.

Melastoma icosandrum var. punctulatum Swartz ex Wikstrom, Vet. akad. Stockholm Handl. 1827, St. I: 65. 1827. Type:—*O. Swartz s.n.* (not seen).

Melastoma icosandrum var. farinulentum Swartz ex Wikstrom, Vet. akad. Stockholm Handl. 1827, St. I: 65. 1827. Type:—*O. Swartz s.n.* (not seen).

## Supplementary Material

XML Treatment for Conostegia

XML Treatment for Conostegia sect. Conostegia

XML Treatment for Conostegia arborea

XML Treatment for Conostegia balbisiana

XML Treatment for Conostegia bernoulliana

XML Treatment for Conostegia bigibbosa

XML Treatment for Conostegia bracteata

XML Treatment for Conostegia brenesii

XML Treatment for Conostegia caelestis

XML Treatment for Conostegia chiriquensis

XML Treatment for Conostegia cuatrecasii

XML Treatment for Conostegia fragrantissima

XML Treatment for Conostegia hirtella

XML Treatment for Conostegia icosandra

XML Treatment for Conostegia jaliscana

XML Treatment for Conostegia lindenii

XML Treatment for Conostegia macrantha

XML Treatment for Conostegia micrantha

XML Treatment for Conostegia montana

XML Treatment for Conostegia muriculata

XML Treatment for Conostegia oerstediana

XML Treatment for Conostegia pittieri

XML Treatment for Conostegia procera

XML Treatment for Conostegia pyxidata

XML Treatment for Conostegia rhodopetala

XML Treatment for Conostegia rufescens

XML Treatment for Conostegia setifera

XML Treatment for Conostegia setosa

XML Treatment for Conostegia superba

XML Treatment for Conostegia volcanalis

XML Treatment for Conostegia vulcanicola

XML Treatment for Conostegia sect. Australis

XML Treatment for Conostegia apiculata

XML Treatment for Conostegia attenuata

XML Treatment for Conostegia centronioides

XML Treatment for Conostegia dentata

XML Treatment for Conostegia extinctoria

XML Treatment for Conostegia lancifolia

XML Treatment for Conostegia lasiopoda

XML Treatment for Conostegia monteleagreana

XML Treatment for Conostegia ortizae

XML Treatment for Conostegia polyandra

XML Treatment for Conostegia rubiginosa

XML Treatment for Conostegia tenuifolia

XML Treatment for Conostegia sect. Geniculatae

XML Treatment for Conostegia allenii

XML Treatment for Conostegia brenesiana

XML Treatment for Conostegia calocoma

XML Treatment for Conostegia centrosperma

XML Treatment for Conostegia cinnamomea

XML Treatment for Conostegia colliculosa

XML Treatment for Conostegia consimilis

XML Treatment for Conostegia dissitiflora

XML Treatment for Conostegia dissitinervia

XML Treatment for Conostegia ecuadorensis

XML Treatment for Conostegia foreroi

XML Treatment for Conostegia fraterna

XML Treatment for Conostegia friedmaniorum

XML Treatment for Conostegia fulvostellata

XML Treatment for Conostegia galdamesiae

XML Treatment for Conostegia grayumii

XML Treatment for Conostegia hammelii

XML Treatment for Conostegia henripittieri

XML Treatment for Conostegia incurva

XML Treatment for Conostegia iteophylla

XML Treatment for Conostegia jefensis

XML Treatment for Conostegia oligocephala

XML Treatment for Conostegia ombrophila

XML Treatment for Conostegia osaensis

XML Treatment for Conostegia papillopetala

XML Treatment for Conostegia peltata

XML Treatment for Conostegia pendula

XML Treatment for Conostegia plumosa

XML Treatment for Conostegia povedae

XML Treatment for Conostegia schlimii

XML Treatment for Conostegia shattuckii

XML Treatment for Conostegia speciosa

XML Treatment for Conostegia subcrustulata

XML Treatment for Conostegia subpeltata

XML Treatment for Conostegia trichosantha

XML Treatment for Conostegia xalapensis
